# It's All in Your Mind: Determining Germ Cell Fate by Neuronal IRE-1 in *C. elegans*

**DOI:** 10.1371/journal.pgen.1004747

**Published:** 2014-10-23

**Authors:** Mor Levi-Ferber, Yehuda Salzberg, Modi Safra, Anat Haviv-Chesner, Hannes E. Bülow, Sivan Henis-Korenblit

**Affiliations:** 1 The Mina and Everard Goodman Faculty of Life Sciences, Bar-Ilan University, Ramat-Gan, Israel; 2 Department of Genetics, Albert Einstein College of Medicine of Yeshiva University, Bronx, New York, New York, United States of America; University of Wisconsin, Howard Hughes Medical Institute, United States of America

## Abstract

The *C. elegans* germline is pluripotent and mitotic, similar to self-renewing mammalian tissues. Apoptosis is triggered as part of the normal oogenesis program, and is increased in response to various stresses. Here, we examined the effect of endoplasmic reticulum (ER) stress on apoptosis in the *C. elegans* germline. We demonstrate that pharmacological or genetic induction of ER stress enhances germline apoptosis. This process is mediated by the ER stress response sensor IRE-1, but is independent of its canonical downstream target XBP-1. We further demonstrate that *ire-1*-dependent apoptosis in the germline requires both CEP-1/p53 and the same canonical apoptotic genes as DNA damage-induced germline apoptosis. Strikingly, we find that activation of *ire-1*, specifically in the ASI neurons, but not in germ cells, is sufficient to induce apoptosis in the germline. This implies that ER stress related germline apoptosis can be determined at the organism level, and is a result of active IRE-1 signaling in neurons. Altogether, our findings uncover *ire-1* as a novel cell non-autonomous regulator of germ cell apoptosis, linking ER homeostasis in sensory neurons and germ cell fate.

## Author Summary

Cells in the *C. elegans* germline undergo programmed cell death as part of the normal developmental program and in response to various stresses. Here, we discovered that more germ cells undergo programmed cell death under stress conditions associated with the accumulation of misfolded proteins in the endoplasmic reticulum, a cellular organelle responsible for protein folding and trafficking. Surprisingly, we found that germ cell death is a consequence of stress in neurons rather than in the germ cells themselves. This implies that germ cell death under ER stress conditions is regulated at the organismal level and implicates signaling between tissues.

## Introduction

Apoptosis, also known as programed cell death (PCD), is a highly conserved fundamental cellular process that provides a self-elimination mechanism for the removal of unwanted cells. PCD is critical for organ development, tissue remodeling, cellular homeostasis and elimination of abnormal and damaged cells [1], [2]. The apoptotic machinery that actually executes cell death is intrinsic to all cells and can be activated in response to extracellular or intracellular cues. These are thought to be mediated by cell death receptors or by cytotoxic stress respectively [3].

In *C. elegans*, 131 somatic cells invariably undergo apoptosis during hermaphrodite development [4], [5]. In contrast, in the adult *C. elegans*, only germ cells undergo apoptotic cell death. These cell deaths can be either physiological or stress-induced [6], [7]. So far, stress-induced germ cell apoptosis has been associated with DNA damage, pathogens, oxidative stress, osmotic stress, heat shock and starvation [7]–[9]. These apoptotic events are restricted to germ cells at the pachytene stage which are located in the loop region of the gonad [6], where oogenesis transition normally occurs [10]. The physiological germ cell apoptosis pathway acts during oogenesis and is thought to act either as a part of a quality control process, preferentially removing unfit germ cells from the gonad, or as a resource re-allocation factor important for maintaining oocyte quality or simply as a gonad homeostatic pathway removing excess germ cells [6], [11], [12]. Both somatic and germ cell apoptosis rely on the highly conserved core apoptotic machinery comprised of the Caspase-3 homolog *ced-3*, the Apaf-1 homolog *ced-4* and the anti-apoptotic Bcl-2 homolog *ced-9* [6], [13]–[16].

All germ cell apoptosis, physiological and stress-induced, relies on the core apoptotic machinery [7]–[9]. However, different upstream genes activate the core apoptotic machinery in the germline in response to different stresses. For example, DNA damage-induced germ cell apoptosis involves the proteins EGL-1, CED-13, and the DNA damage response protein p53 homolog CEP-1 [9], [17]–[19]. In contrast, oxidative, osmotic, heat shock and starvation stresses induce germ cell apoptosis through a CEP-1 and EGL-1 independent pathway and rely on the MEK-1 and SEK-1 MAPKs instead [7].

The endoplasmic reticulum (ER) fulfills many essential cellular functions, including a role in the secretory pathway, in lipid metabolism and in calcium sequestration. Accordingly, ER homeostasis is essential for proper cellular function [20]. A specialized, conserved cellular stress response, called the unfolded protein response (UPR), is in charge of detecting ER stress and adjusting the capacity of the ER to restore ER homeostasis. In *C. elegans*, as in humans, three proteins located at the ER membrane sense ER stress and activate the UPR: the ribonuclease inositol-requiring protein-1 (IRE-1), the PERK kinase homolog PEK-1 and the activating transcription factor-6 (ATF-6) [21]. Of the three, IRE-1 is the major and most highly conserved ER stress sensor. In response to ER stress, IRE-1 activates the ER stress-related transcription factor XBP-1, which induces the transcription of genes that help restore ER homeostasis [22]–[24]. Accordingly, *ire-1* and *xbp-1* deficiencies perturb ER homeostasis [25], [26].

Although IRE-1 typically protects cells, upon excessive and prolonged ER stress, IRE-1 can also trigger cell death, usually in the form of apoptosis [27], [28]. For example, IRE-1 can lead to activation of the cell death machinery via JNK and caspase activation [29], [30] or by mediating decay of critical ER-localized mRNAs through the RIDD pathway, tipping the balance in favor of apoptosis [31]. These functions of IRE-1 are independent of XBP-1 [29]–[33].

Highly proliferating cells with a high protein and lipid biosynthetic load are thought to rely on ER function to a greater extent than other cells. This together with the general sensitivity of the germline to cellular stresses prompted us to investigate the effects of ER stress on germ cell fate. Strikingly, we discovered that ER stress does not simply kill the germ cells by not meeting their biosynthetic demands. Instead, we found that ER stress initiates a signaling cascade in neurons that regulates germ cell survival non-autonomously. Thus, our findings reveal that germ cell sensitivity to ER stress conditions can be regulated at an organismal level and can be uncoupled from germ cell stress.

## Results

### Disruption of ER homeostasis induces germ cell apoptosis

**Figure 1:**
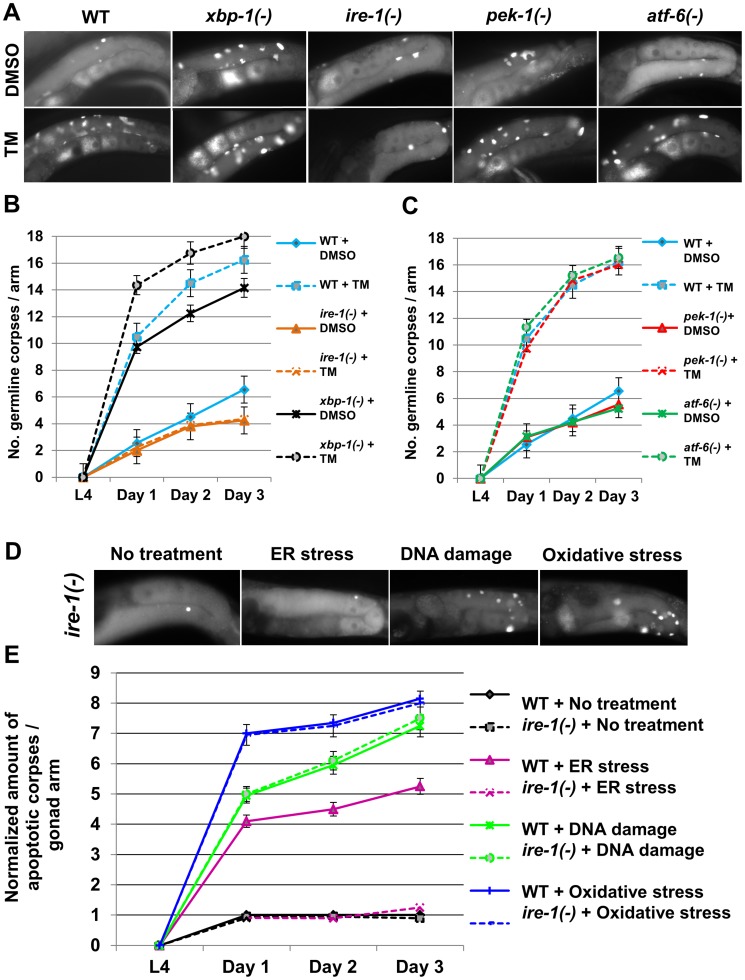
*ire-1* is specifically required for ER stress induced germ cell apoptosis. (**A–C**) Animals of the indicated genotypes were treated with DMSO (solid lines) or with 25 µg/ml tunicamycin (TM, dashed lines) from day-0 (L4). Germ cell corpses were identified by SYTO12 staining. (A) Representative fluorescence micrographs (400-fold magnification) of SYTO12-stained germ cell corpses of day-2 animals. (B–C) Time course experiment presenting average number of apoptotic corpses per gonad arm, from day-0 (L4) to day-3 of adulthood, 160 animals were analyzed per genotype. Error bars mark SEM. (**D–E**) Wild-type (WT) or *ire-1(−)* animals were treated with control RNAi, *tfg-1* RNAi (which induces ER stress by abrogating protein export from the ER), *rad-51* RNAi (to induce DNA damage) or with 10 mM paraquat (to induce oxidative stress). (D) Representative fluorescence micrographs (400-fold magnification) of SYTO12-stained germ cell corpses of control or control or stressed *ire-1(−)* day-2 mutants. (E) The normalized amount of apoptotic corpses per gonad arm from day-0 (L4) to day-3 of adulthood is presented. The average amount of SYTO12-labeled apoptotic corpses per gonad arm was normalized to the average number of mitotic germ cells in each of the indicated genotypes. 120 animals per treatment were analyzed. Error bars mark SEM.

To investigate whether ER stress induces apoptosis in the *C. elegans* germline, we first assessed the number of apoptotic corpses in the gonads of animals treated with tunicamycin, a chemical ER stress inducer which blocks N-linked glycosylation. Apoptotic corpses in the gonad were identified by staining with the vital dye SYTO12 and by their discrete cellularization within the germline syncytium. We found that tunicamycin treatment increased the number of apoptotic germ cells present in wild-type gonads by approximately 3 fold compared to control DMSO treatment from day-1 to day-3 of adulthood (P<0.001, **Figure 1A–B**).

If indeed the increased number of germline corpses in tunicamycin-treated animals is a consequence of ER stress, then additional manipulations that disrupt ER homeostasis should also increase germ cell apoptosis. *tfg-1* encodes a protein that directly interacts with SEC-16 to control COPII subunit accumulation at ER exit sites and is required for the vesicular export of cargo from the ER [34]. We hypothesized that ER homeostasis would be disrupted in *tfg-1*-deficient animals. To examine the effect of *tfg-1* deficiency on ER homeostasis, we assessed the effect of *tfg-1* RNAi treatment on the levels of the ER stress response reporter *Phsp-4::gfp* [22]. *tfg-1* RNAi efficacy was confirmed by the reduction in the animals' body size compared to control RNAi treated animals [35]. We found that treatment with *tfg-1* RNAi specifically activated the ER stress response, as it increased the level of the ER stress response reporter without increasing the expression of oxidative stress response, heat shock response or mitochondrial stress response reporters (**Figure S1**).

**Figure 2:**
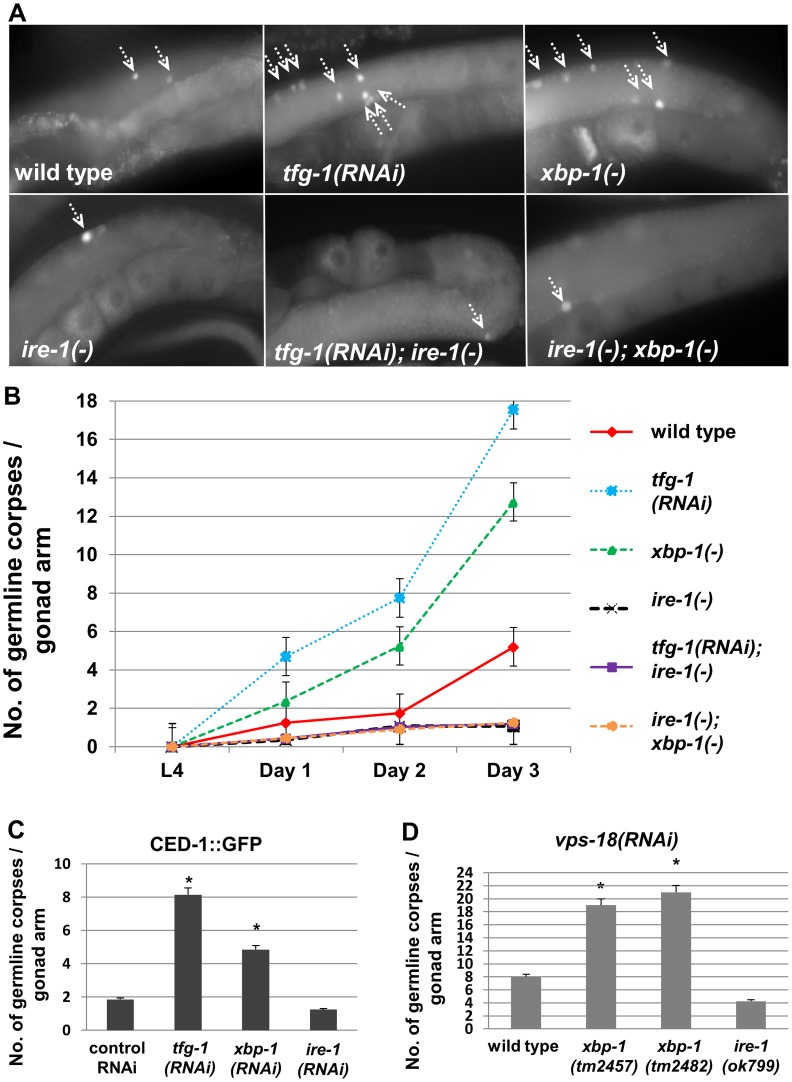
Genetically-induced ER stress increases germ cell apoptosis in an *ire-1*-dependent manner. (A–B) Animals of the indicated genotypes were treated with control or *tfg-1* RNAi. Germ cell corpses were identified by SYTO12 staining. (**A**) Representative fluorescence micrographs (400-fold magnification) of SYTO12-stained germ cell corpses in day-2 adults. Top panels present gonads of wild-type, *xbp-1(tm2457)* or *tfg-1* RNAi treated animals. Bottom panels present gonads of the corresponding genotypes in an *ire-1(ok799)* background. Arrows point at SYTO12-stained germ cell corpses. (**B**) Time course analysis of cell corpses in the adult gonads of the indicated genotypes. SYTO12-labeled cell corpses were scored at L4, day 1, day 2 and day 3 of adulthood. Each point represents the mean number of cell corpses scored in 30 gonads. Error bars indicate SEM. (**C**) Animals expressing CED-1::GFP were treated with control, *tfg-1, xbp-1* or *ire-1* RNAi and analyzed. Bar graph shows average number of apoptotic corpses per gonad arm in the indicated genotypes identified by their engulfment by CED-1::GFP labeled gonadal sheath cells. Asterisk indicates a significant increase in germline apoptosis compared to control RNAi-treated animals (Student's t-test values of P<0.001). (**D**) Bar graph shows average number of SYTO12-labeled apoptotic corpses per gonad arm in day-2 *vps-18* RNAi-treated animals (n = 40 gonads per genotype). Asterisk indicates a significant increase in germline apoptosis compared to wild-type animals treated with *vps-18* RNAi) Student's t-test values of P<0.001).

In terms of germ cell apoptosis, we observed that *tfg-1* RNAi consistently increased the number of apoptotic germ cells in the gonad by approximately 4 fold from day-1 to day-3 of adulthood compared to wild-type animals (P<0.001, **Figure 2A,B**). A similar 4 fold increase in germ cell apoptosis was observed by scoring germ cell engulfment by neighboring cells that expressed GFP-labeled CED-1, a transmembrane receptor that mediates cell corpse engulfment in *C. elegans* [36] (**Figure 2C**). *tfg-1* RNAi treatment did not increase the number of SYTO12-labeled cells in the gonads of apoptosis-defective *ced-3(n1286)* mutants, confirming that the dye specifically labels apoptotic cells (**Figure S2**). Together, these results indicate that conditions that disrupt ER homeostasis, including tunicamycin treatment or blocking secretory traffic from the ER, increase apoptosis frequency in the gonad compared to non-stressed animals.

### ER stress-induced germ cell apoptosis is mediated by the apoptotic machinery implicated in DNA damage-induced germ cell apoptosis

**Figure 3:**
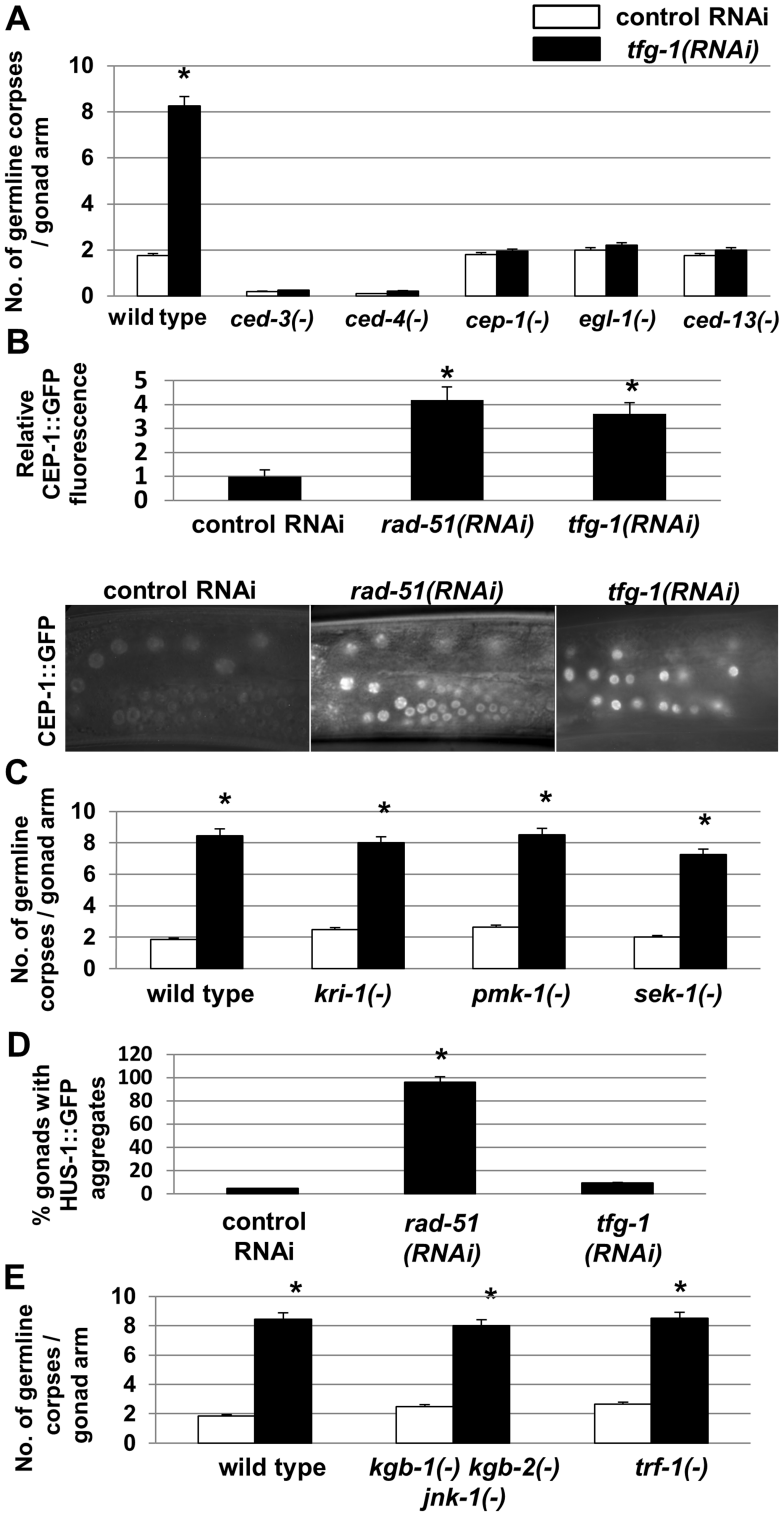
ER stress-induced germ cell apoptosis is mediated by the same apoptotic machinery implicated in DNA damage-induced apoptosis. (**A,C,E**) Bar graphs present average number +/−SEM of SYTO12-labeled apoptotic corpses per gonad arm in day-2 animals of the indicated genotypes treated with control RNAi (white bars) or *tfg-1* RNAi (black bars). At least 50 gonads per genotype were scored. Asterisks mark Student's t-test values of P<0.001 between *tfg-1* RNAi and control RNAi. (**B**) Mean fluorescence and representative fluorescence micrographs (400-fold magnification) of CEP-1::GFP expression in the germ cells of CEP-1::GFP transgenic animals treated with control, *rad-51* or *tfg-1* RNAi. Each bar graph presents the average fluorescence of 100 germ cells from 10 gonads. *rad-51* RNAi treatment served as a positive control for DNA damage-induced expression of CEP-1::GFP. Asterisks mark Student's t-test values of P<0.001 compared to control RNAi treatment. (**D**) Bar graph presents percentage of dissected gonads of HUS-1::GFP transgenic animals treated with control, *rad-51* or *tfg-1* RNAi, in which HUS-1::GFP aggregates were detected. 50–60 transgenic gonads were analyzed. *rad-51* RNAi treatment served as a positive control for DNA damage-induced HUS-1::GFP aggregation. Asterisks mark Student's t-test values of P<0.001 compared to control RNAi treatment. Error bars present SEM.

We next asked which apoptotic machinery is implicated in ER stress-induced germ cell apoptosis. To this end, we examined mutants deficient in core-apoptotic genes as well as mutants deficient in genes specifically implicated in germ cell apoptosis. This array of apoptosis-related mutants was treated with control or *tfg-1* RNAi, and germ cell apoptosis was scored by SYTO12 labeling. As expected, we found that the core apoptosis machinery genes *ced-3* and *ced-4* [6], [13], [15], [16] were required for germ cell apoptosis in response to ER stress (**Figure 3A**). Importantly, the *cep-1*, *egl-1* and *ced-13* genes, previously implicated in DNA damage-induced apoptosis [9], [17]–[19], were also found to be completely essential for germ cell apoptosis in response to *tfg-1* RNAi treatment (**Figure 3A**). Accordingly, the levels of a CEP-1::GFP translational fusion transgene driven by the *cep-1* promoter were increased within the germ cells of *tfg-1* RNAi-treated animals (P<0.001, **Figure 3B**). In contrast, *pmk-1* and *sek-1*, previously implicated in oxidative stress-induced and pathogen-induced germ cell apoptosis respectively [7], [8], were dispensable for germ cell apoptosis in response to *tfg-1* RNAi treatment (**Figure 3C**). Thus, the genetic analysis clearly implicated the apoptotic machinery that mediates DNA damage-induced germ cell apoptosis in ER stress-induced germ cell apoptosis as well.

The strong changes in CEP-1 levels observed in *tfg-1* RNAi-treated animals suggest that ER stress controls CEP-1 activation within the germ cells. One possible explanation for the involvement of genes implicated in DNA damage-induced germ cell apoptosis is that ER stress indirectly damages DNA, which in turn leads to CEP-1 activation and DNA damage-induced germ cell apoptosis. However, whereas a previous study implicated the intestinal *kri-1* gene in non-autonomous regulation of ionizing-radiation induced germ cell apoptosis [37], we found that *tfg-1* RNAi treatment efficiently induced germ cell apoptosis in *kri-1*-deficient animals (Figure 3C). This genetic uncoupling between the requirements for ionizing-radiation induced germ cell apoptosis and ER stress-induced germ cell apoptosis suggest that ER stress does not simply induce DNA damage which in turn leads to germ cell apoptosis.

To further substantiate this conclusion, we examined directly whether the DNA damage response is activated in the germ cells of ER stressed- animals treated with *tfg-1* RNAi. To this end, we followed the nuclear aggregation of HUS-1::GFP, which encodes a DNA damage checkpoint protein that relocalizes to distinct nuclear foci upon induction of DNA damage [38]. Whereas nuclear HUS-1::GFP aggregates were clearly observed in the germ cells of DNA-damaged *rad-51* RNAi treated animals (P<0.001 compared to control RNAi), HUS-1::GFP aggregates were not detected in *tfg-1* RNAi treated animals (P = 0.78 compared to control RNAi, **Figure 3D**). Thus, ER stress activates CEP-1/p53 to induce germ cell apoptosis without generating DNA damage.

### Conditions that disrupt ER homeostasis induce germ cell apoptosis *via* the ER stress sensor *ire-1*

We next asked whether any of the canonical ER stress sensing genes was implicated in ER stress-induced germ cell apoptosis. To this end, we examined mutants deficient in the ER stress-response sensor genes that comprise the UPR: *ire-1, pek-1* or *atf-6*. This array of mutants was treated with DMSO or with tunicamycin and germ cell apoptosis was scored by SYTO12 labeling. We found that similarly to its effect in wild-type animals, tunicamycin treatment increased the number of germline corpses in *atf-6* and *pek-1*-deficient animals by approximately 3 fold from day-1 to day-3 of adulthood (P<0.001, **Figure 1A,C**). In contrast, ER stress induced by tunicamycin treatment failed to increase germ cell apoptosis in *ire-1* mutants (**Figure 1A,B**). Therefore, the insensitivity of the germline to tunicamycin is unique to *ire-1*-deficient animals and not seen in animals deficient in other UPR sensors.

*ire-1* mutants are abnormal in terms of their gonad anatomy and their reproductive capacity: *ire-1* mutants have approximately 2 fold less progeny and 2 fold less mitotic germ cells within their proliferative zones compared to *ire-1(+)* wild-type animals (P<0.001, **Figures S3A,D**). Thus, we wondered whether these abnormalities affected the ability of their germ cells to undergo apoptosis in general or whether they were specifically defective in their ability to undergo apoptosis in response to ER stress.

First we assessed germline apoptosis in *ire-1(−)* mutants under normal growth conditions, from day-0 (L4) to day-3 of adulthood. At all timepoints, we detected approximately half the amount of germline corpses in *ire-1(−)* gonads compared to *ire-1(+)* wild-type gonads, as assessed by SYTO12 labeling and by the CED-1::GFP engulfment marker (**Figure 2A–C**). The low levels of germ cell apoptosis persisted in *ire-1* mutants treated with *vps-18* RNAi (**Figure 2D**), a treatment that impairs germ cell corpse clearance [39]. However, normalization of the number of apoptotic corpses to the number of mitotic germ cells resulted in comparable levels of germ cell corpses in *ire-1* mutants and in non-stressed wild-type animals (P = 0.12, **Figure S3C**). This indicates that in spite of their reproductive abnormalities, physiological germ cell apoptosis in *ire-1(−)* and *ire-1(+)* animals is comparable.

Next, we assessed stress-induced germline apoptosis in *ire-1* mutants. We found that although manipulations that disrupt ER homeostasis fail to increase germ cell apoptosis in *ire-1* mutants (**Figure 1B,1D–E**), DNA damage and oxidative stress conditions did increase germ cell apoptosis in *ire-1* mutants (P<0.001 compared to non-stressed *ire-1* mutants, **Figure 1D–E**). This resulted in a similar level of germline apoptosis as in stressed wild-type animals upon normalization of the number of apoptotic corpses to the number of mitotic germ cells (P = 0.44 for DNA damage and P = 0.42 for oxidative stress, **Figure 1D–E**). Thus, in spite of the reproductive abnormalities of *ire-1* mutants, the germ cells of these mutants undergo stress-induced apoptosis similarly to wild-type animals, however not in response to ER stress. The inability of *ire-1* mutants to increase germline apoptosis specifically in response to perturbations in ER homeostasis suggests that IRE-1 may be a critical mediator of ER stress-induced germ cell apoptosis.

### IRE-1 function in ER stress-induced germ cell apoptosis is independent of its canonical downstream target XBP-1

The most established mode of action of IRE-1 under ER stress conditions is *via* the activation of the UPR-related transcription factor XBP-1 [22]–[24]. Therefore, if IRE-1 enabled ER stress-induced germ cell apoptosis *via* its downstream target *xbp-1*, then the number of germline corpses detected in *xbp-1*(−) mutants should remain low under ER stress conditions, similarly to *ire-1*(−) mutants.

In order to test this, we first examined germ cell apoptosis in *xbp-1(tm2457)* null mutants. Surprisingly, in contrast to *ire-1(−)* mutants, we consistently detected increased germ cell apoptosis in *xbp-1*(−) gonads compared to wild-type gonads under normal growth conditions. A 2.5 fold increase in the number of germline corpses in *xbp-1(−)* mutants was detected by SYTO12 labeling of gonads from day-1 to day-3 of adulthood compared to wild-type animals (P<0.001, **Figure 2A–B**). A similar observation was apparent by using the CED-1::GFP engulfment marker (**Figure 2C**). The 2.5 fold increase in the number of germ cell corpses was still apparent in engulfment defective *vps-18* RNAi-treated animals (**Figure 2D**) and upon normalization to the number of mitotic germ cells located in the proliferative zone (**Figure S3C**). Thus, in contrast to *ire-1* mutants and wild-type animals, *xbp-1* mutants exhibit a high basal level of germ cell apoptosis.

We hypothesized that the increase in germline apoptosis in *xbp-1* mutants may be due to perturbed ER homeostasis in these animals [25], [26], If so, then it should be mediated *via ire-1*, similarly to other ER stress conditions that induce germ cell apoptosis. Accordingly, we found that in an *ire-1(−)* background, the *xbp-1* mutation did not increase germ cell apoptosis. This observation was consistent along different time points spanning from day-0 (L4) to day-3 of adulthood (**Figure 2A–B**). This also persisted upon normalization to the number of mitotic germ cells located in the proliferative zone (**Figure S3C**). Interestingly, the amount of mitotic germ cells of *xbp-1; ire-1* double mutants was similar to that of *xbp-1* single mutants (P = 0.072, **Figure S3A**), whose germ cells were responsive to ER stress-induced apoptosis (**Figure S3B–C**). This indicates that the reduced amount of mitotic cells in the gonad of *ire-1* mutants can be uncoupled from the inability of their germ cells to undergo ER stress-associated apoptosis.

The finding that *xbp-1* deficiency per se promotes *ire-1*-dependent ER stress-induced germ cell apoptosis suggests that *xbp-1* is dispensable for increasing germ cell apoptosis in response to ER stress. Consistent with this, we found that tunicamycin treatment further increased germ cell apoptosis in *xbp-1* mutants (P<0.001, **Figure 1A–B**). Altogether, these results lend further support to the notion that *ire-1* is a critical signaling molecule in mediating ER stress-induced germline apoptosis, whereas its' downstream canonical target *xbp-1* is not. Furthermore, since ER function is compromised both in *ire-1* and in *xbp-1* deficient mutants [25], [26], the differential ability to induce germ cell apoptosis in these mutants suggests that germ cell apoptosis may be the result of active IRE-1 signaling, rather than simply a consequence of ER dysfunction.

In mammalian cells, activation of IRE1 can cell-autonomously activate JNK *via* the adaptor protein TRAF. Consequently, IRE1-mediated activation of JNK initiates proapoptotic signaling, independently of XBP1 [29]. Thus, we examined whether the *C. elegans* homologs of TRAF and JNK proteins were required for *ire-1*/ER stress-induced apoptosis in *C. elegans*, which is also independent of *xbp-1*. To this end, *trf-1* mutants or mutants deficient in all three *C. elegans* JNK homologs were treated with control or *tfg-1* RNAi. We found that *tfg-1* RNAi increased germ cell apoptosis independently of the *trf-1* and the JNK-like genes (**Figure 3E**). Thus, since ER stress can effectively induce germ cell apoptosis in the absence of *xbp-1, trf-1* and JNK homologs, the signaling mediated by IRE-1, in this case, must be executed by an alternative *xbp-1*-independent output of IRE-1.

### ER stress in the ASI sensory neurons regulates germ cell apoptosis cell non-autonomously

**Figure 4:**
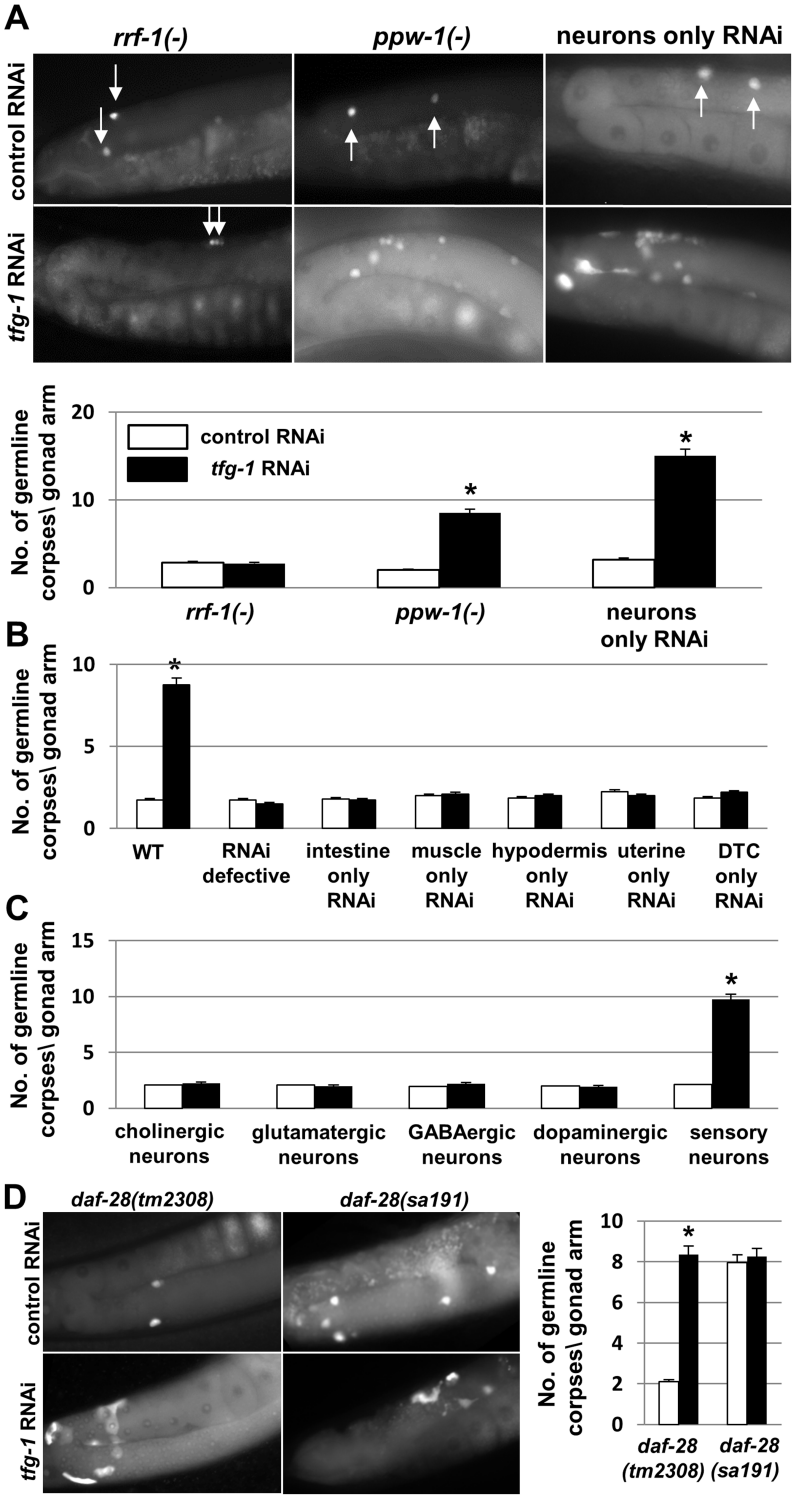
ER stress specifically in sensory neurons is sufficient to induce germline apoptosis. (**A**) Bar graph and representative fluorescence micrographs (400-fold magnification) of SYTO12-stained germ cell corpses in day-2 adults. Arrows point at SYTO12 stained germ cell corpses. Bar graph presents average number +/−SEM of apoptotic corpses per gonad arm in the indicated genotypes (n = 60 per genotype). Asterisks mark Student's t-test values of P<0.001 of *tfg-1* RNAi treated animals compared to the corresponding control RNAi treated animals. Note that in animals in which RNAi functions only in the neurons, *tfg-1* RNAi increased the amount of germ cell corpses to a greater extent than in *ppw-1* mutants or wild-type animals. This may be due to more efficient *tfg-1* RNAi uptake in the neurons of these animals, whose neurons over-express SID-1 [76] (**B,C**) Bar graph presents average number +/− SEM of SYTO-12-labeled germ cell corpses of day-2 adults of the indicated genotypes (n = 60 gonads per genotype). Asterisks mark Student's t-test values of P<0.001 of *tfg-1* RNAi treated animals (black bars) compared to the corresponding control RNAi treated animals (white bars). See materials and methods for strains details. (**D**) Representative fluorescence micrographs (400-fold magnification) and bar graph of SYTO12-stained germ cell corpses in day-2 *daf-28* mutant strains treated with control RNAi (white bars) or *tfg-1* RNAi (black bars). Bar graph presents average number +/− SEM of apoptotic corpses per gonad arm in the indicated genotypes (n = 70 per genotype). *tm2308* is a deletion mutation in the *daf-28* gene. *sa191* is a point mutation that interferes with the posttranslational processing of DAF-28. Asterisks mark Student's t-test values of P<0.001 of *tfg-1* RNAi treated animals compared control RNAi treated animals.

Next, we examined whether ER stress triggers programmed cell death autonomously within the germ cells, or non-autonomously from the soma. To test this, we used *tfg-1* RNAi to induce ER stress specifically in the germline or in the soma. To induce ER stress primarily in the germ cells, mutants in the *rrf-1* gene, encoding an RNA-directed RNA polymerase (RdRP) homolog required for most somatic RNAi but not for germline RNAi [40], were treated with *tfg-1* RNAi. No increase in the amount of germline corpses was observed as a result of *tfg-1* RNAi treatment in *rrf-1* mutants (P = 0.19, **Figure 4A**). To induce ER stress specifically in the soma, mutants in the *ppw-1* gene, which is required for efficient RNAi in the germline [41], were treated with *tfg-1* RNAi. This resulted in a 4.5 fold increase in the amount of apoptotic corpses in the gonads (P<0.001, **Figure 4A**). Thus, ER stress in the soma, rather than in the germ cells, is sufficient for the induction of germ cell apoptosis.

Does germ cell apoptosis occur upon disruption of ER homeostasis in the entire soma or does it occur in response to ER stress in a particular part of the soma? To answer this, ER stress was induced locally in specific somatic tissues. This was achieved by treating animals expressing functional RNAi machinery only in specific tissues with *tfg-1* RNAi and assessing germ cell apoptosis in these animals. We found that *tfg-1* RNAi treatment did not increase germ cell apoptosis in animals which respond to RNAi only in the intestine, in the muscle, in the hypodermis, in the uterine or in the distal tip cells (P>0.1 in each one of these strains, **Figure 4B**). In contrast, *tfg-1* RNAi treatment increased germ cell apoptosis by approximately 7 fold in animals which respond to RNAi specifically in the neurons (P<0.001, **Figure 4A**).

Next, we examined whether ER stress-induced germline apoptosis is under pan-neuronal control or under the control of specific neurons. To this end, we introduced ER stress-inducing *tfg-1* RNAi into animals expressing functional RNAi machinery specifically in the cholinergic, glutamatergic, GABAergic, dopaminergic or in a subset of sensory neurons. Importantly, we found that *tfg-1* RNAi treatment increased germ cell apoptosis only in animals whose sensory neurons responded to RNAi (**Figure 4C**).

Among the sensory neurons whose exposure to ER stress increased germline apoptosis were the ASI neurons, which have been previously implicated in the regulation of germ cell proliferation and maturation [42]. Hence, we examined whether ER stress in the ASI sensory neurons alone is sufficient for the induction of germ cell apoptosis in the gonad. To this end, we first assessed germline apoptosis in *daf-28(sa191)* mutants, which produce a toxic insulin peptide that activates the UPR specifically in the ASI neurons [43]. We found that germ cell apoptosis in the gonads of *daf-28(sa191)* mutants was increased by approximately 4 fold compared to wild-type animals (P<0.001, **Figure 4D**). Importantly, germ cell apoptosis was not increased in a *daf-28(tm2308)* null strain, which is deficient in *daf-28* and does not produce the toxic insulin peptide which induces ER stress (P = 0.15, **Figure 4D**). *tfg-1* RNAi treatment of the two *daf-28* mutant strains increased the number of germline corpses in *daf-28(tm2308)* null strain (P<0.001), but did not further increase germline apoptosis in the *daf-28(sa191)* strain (P = 0.09, **Figure 4D**). *tfg-1* RNAi treatment did not alter ASI overall morphology as assessed by the expression pattern of a GFP reporter driven by an ASI-specific promoter (**Figure S4A**). Together, these findings suggest that expression of the toxic form of DAF-28 and *tfg-1*-deficiency increase germ cell apoptosis by similar means; most likely by causing ER stress and activating the UPR in the ASI neurons.

### Activation of IRE-1 in ASI neurons is sufficient to induce germ cell apoptosis

We have demonstrated that in the absence of the ER stress sensor *ire-1*, ER stress does not increase germline apoptosis. We further demonstrated that ER stress in the ASI sensory neurons is sufficient to induce germ cell apoptosis. Thus, we next examined whether it is also sufficient to express *ire-1* in the soma, and specifically in the ASI neurons, to restore germ cell apoptosis in response to ER stress.

**Figure 5:**
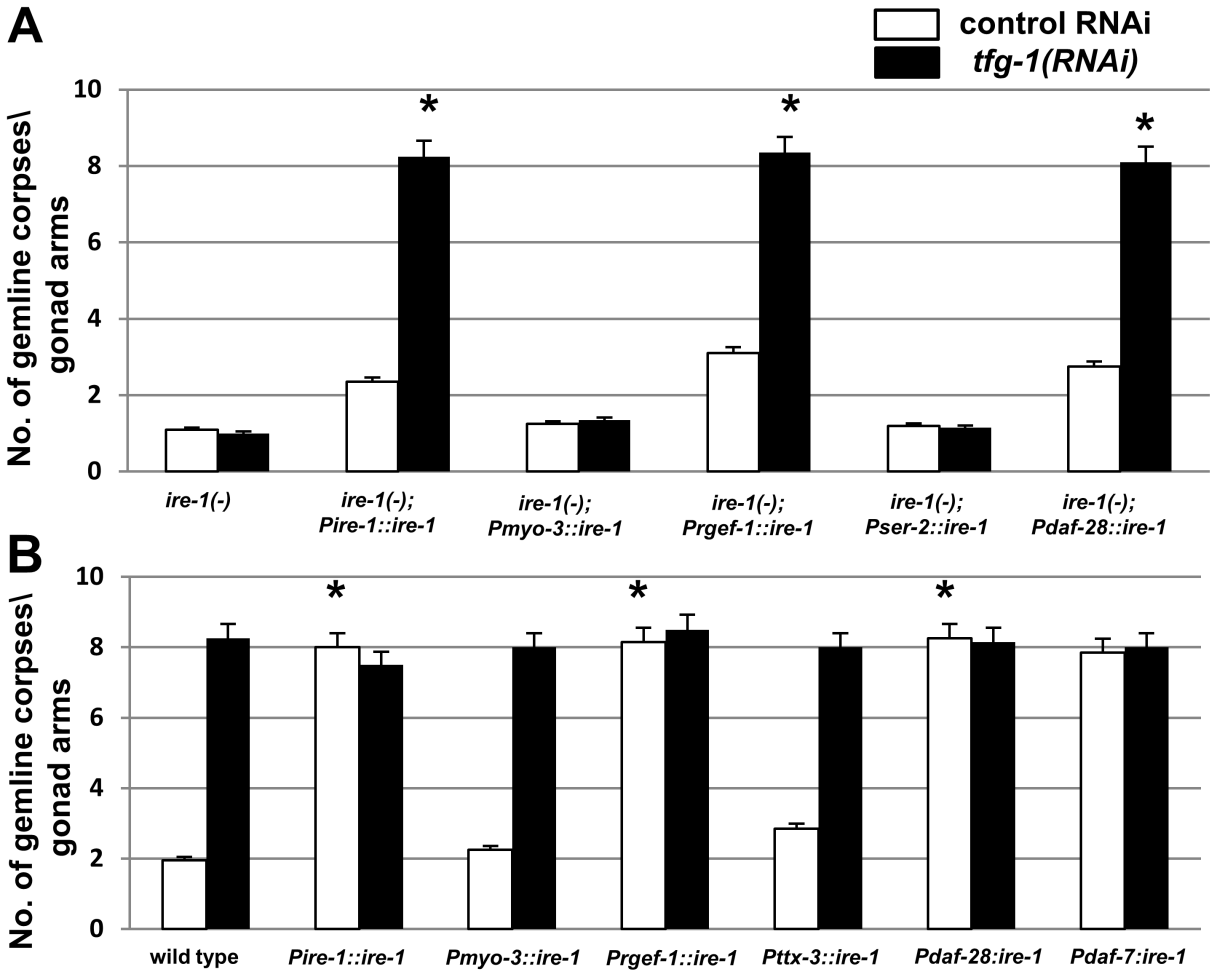
Over-expression of *ire-1* in the ASI pair of amphid neurons is sufficient to induce germline apoptosis independently of ER stress. (**A–B**) Bar graph presents average number of apoptotic corpses per gonad arm in day-2 *ire-1(−)* or *ire-1(+)* animals expressing an *ire-1* transgene driven by the indicated promoters (n = 100 gonads per genotype). Animals were treated with control RNAi (white bars) or with *tfg-1* RNAi (black bars). Asterisks mark Student's t-test values of P<0.001 of each strain treated with *tfg-1* RNAi compared to its' control RNAi treatment. Error bars indicate SEM. (**A**) Germ cell apoptosis in *ire-1(−)* mutants expressing an *ire-1* rescuing transgene in different tissues is presented. The *ire-1* rescuing transgene was expressed in somatic cells (*Pire-1::ire-1*), in neurons (*Prgef-1::ire-1*, *Pdaf-28::ire-1* and *Pser-2::ire-1*, which drive expression in all neurons, in the ASI/ASJ neurons or in the PVD and OLL neurons respectively) and in muscle cells (*Pmyo-3::ire-1*). (**B**) Germ cell apoptosis in *ire-1(+)* animals overexpressing an *ire-1* transgene in different tissues is presented. The *ire-1* transgene was expressed in somatic cells (*Pire-1::ire-1*), in neurons [driven by the promoters of *rgef-1* (pan-neuronal expression), *daf-28* (expressed in the ASI/ASJ neurons), *daf-7* (expressed only in the ASI neurons) and *ttx-3* (expressed in the AIY interneurons)] and in muscle (*Pmyo-3::ire-1*).

To this end, we restored *ire-1* expression in the entire soma, pan-neuronally or specifically in the ASI/ASJ neurons of *ire-1(−)* mutants. This was achieved using multi-copy *ire-1* transgenes under *ire-1, rgef-1* and *daf-28* promoters respectively. Since the expression of multi-copy transgenes is normally suppressed in germ cells [44], and due to the specificity of their promoters, these transgenes restore *ire-1* expression within different parts of the soma but not in the germline. We found that expression of each of these *ire-1* transgenes completely restored the increase in germline apoptosis in response to treatment with *tfg-1* RNAi (P<0.001, compare white and black bars within each strain in **Figure 5A**). Similarly, we restored *ire-1* expression in muscle cells and in the PVD and OLL neurons using multi-copy *ire-1* transgenes under *myo-3* and *ser-2* promoters respectively. No increase in germline apoptosis in response to *tfg-1* RNAi treatment was apparent in these two transgenic lines compared to control RNAi treatment (P>0.1, **Figure 5A**). The fact that not all *ire-1* transgenes induced apoptosis supports the notion that *ire-1*-induced germline apoptosis is not the result of leaky expression of the transgenes in other tissues. Altogether, this implies that not all tissues and not all neurons are involved in the regulation of this process.

Next, we asked whether increasing IRE-1 levels to a greater extent may be sufficient for inducing germline apoptosis even in the absence of ER stress. To this end, we overexpressed *ire-1* transgenes in various tissues or cells of *ire-1(+)* wild-type animals. This was achieved by using multi-copy *ire-1* transgenes under *ire-1, rgef-1*, *daf-28* and *daf-7* promoters. This is consistent with the interpretation that some activation of IRE-1 is achieved merely by its over-expression, as has been previously observed in yeast and in mammalian cells [45], [46]. We found that this artificial activation of IRE-1 in the soma, pan-neuronally or specifically in the ASI/ASJ neurons of *ire-1(+)* animals was sufficient to induce high levels of germ cell apoptosis (P<0.001, compare white bars of transgenic animals to that of wild-type animals in **Figure 5B**). No increase in germ-cell apoptosis was observed upon overexpression of an *ire-1* transgene in muscle cells or in the AIY neurons in *ire-1(+)* animals (P>0.1, **Figure 5B**). These findings support the claim that the rescuing activity of the *ire-1* transgenes stems from their expression in specific neurons.

*tfg-1* RNAi treatment of *ire-1(+)* animals over-expressing the *ire-1* transgenes in the soma, in the neurons and specifically in the ASI/ASJ neurons did not further increase germline apoptosis (P>0.5, compare white and black bars within the strains, **Figure 5B**). This suggests that IRE-1 overexpression and *tfg-1*-deficiency increase germ cell apoptosis by similar means, *i.e.* by activating IRE-1. Taken together, our data demonstrate that activation of *ire-1* specifically in the ASI neurons, either by ER stress in the ASI neurons or by IRE-1 overexpression, can non-autonomously regulate germ cell apoptosis. Furthermore, since over-expression of transgenic IRE-1 is sufficient for its artificial activation in a manner that is independent of ER stress, this further suggests that active IRE-1 signaling in the ASI neurons per se, rather than neuronal ER stress or ER dysfunction, is the cause of germ cell apoptosis.

## Discussion

Understanding the molecular events that regulate the life-death decision of cells is of fundamental importance in cell biology research, cell development, cancer biology and disease biology [47]. In this study, we gained new and fascinating insights into the complex coupling between ER stress in the nerve system and germ cell apoptosis.

We report for the first time that germ cells undergo apoptosis in response to ER stress. We find that activation of the ER stress response gene *ire-1* is required and sufficient to induce germ cell apoptosis in response to several ER stress-inducing conditions. Strikingly, we find that germ cell fate is regulated non-autonomously by ER stress and/or through IRE-1 activation specifically in the ASI neurons. This implies that ER homeostasis and UPR signaling in the germ cells themselves is not a factor in determining their fate, ruling out the possibility that these apoptotic events are part of a quality control process that removes “stress-damaged” germ cells from the gonad [6], [11], [12]. Furthermore, this assigns a central neuroendocrine role for the ASI neuron pair in coupling between stress sensing and the onset of germ cell apoptosis. This is in addition to other central physiological processes in *C. elegans*, such as dauer formation [48], [49] and longevity [50], [51], that are also controlled by the sensory ASI neuron pair. Interestingly, another pair of sensory neurons, the ASJ neurons, has been previously implicated in the protection of germ cells from apoptosis under hypoxic conditions [52]. Thus, depending on the stress condition, different neurons can shift germ cell fate from survival to death or vice versa.

How might IRE-1 activation in the ASI neurons dictate germ cell survival or death? One possibility is that defects associated with *ire-1* deficiency and/or *ire-1* activation indirectly abrogate the communication between the neurons and the gonad. However, several lines of evidence undermine this hypothesis: (1) We find that ER stress-induced germ cell apoptosis proceeds normally in animals with a severely defective nervous system (**Figure S4B,C**). This implies that germline apoptosis does not result from a generic neuronal defect. (2) *ire-1* deficiency is associated with germline abnormalities which include a significant reduction in the number of mitotic germ cells and in reduced progeny number. However, these gonad-related defects do not confer generic resistance to stress-induced apoptosis as the germ cells of *ire-1* mutants do undergo apoptosis in response to a variety of stresses. Furthermore, a mutation in *xbp-1*, which improved the reproductive abnormalities of *ire-1* mutants, did not restore responsiveness to ER stress induced germ cell apoptosis in *ire-1; xbp-1* double mutants, thus uncoupling the two. (3) Whereas the comparison of germ cell apoptosis in *ire-1* and wild-type animals may be confined by the basal discrepancy of their reproductive systems, this concern does not exist in the analysis of *ire-1* overexpressing strains, whose gonad appears to be normal (P>0.1 for *Pire-1::ire-1* and *Pdaf-7::ire-1* compared to wild-type animals **Figure S3A,D**). Similarly, this concern does not exist in the intra-strain comparisons of germline apoptosis within the *ire-1(−)* strain under control and stress conditions.

If *ire-1* misregulation in the ASI neurons does not indirectly abrogate the communication between the neurons and the gonad, how might it dictate germ cell survival or death? IRE-1 is a dual-activity enzyme, bearing both kinase and endoribonuclease activities and a propensity to self-aggregate at the ER membrane in response to ER stress. The most characterized mode of action of IRE-1 is the activation of its downstream transcription factor XBP-1 [53]. Significantly less characterized are XBP-1 independent targets of IRE-1, that include activation of the cell death machinery *via* JNK/TRAF signaling and degradation of ER-localized mRNAs that encode secreted and membrane proteins in a process called RIDD [29], [30], [32], [54]–[58]. Since we find that ER stress can effectively induce germ cell apoptosis in the absence of *xbp-1, trf-1* and JNK homologs, the signaling mediated by IRE-1 in this case may be executed by the RIDD pathway or *via* a novel, yet undescribed, *xbp-1*-independent output of IRE-1.

**Figure 6:**
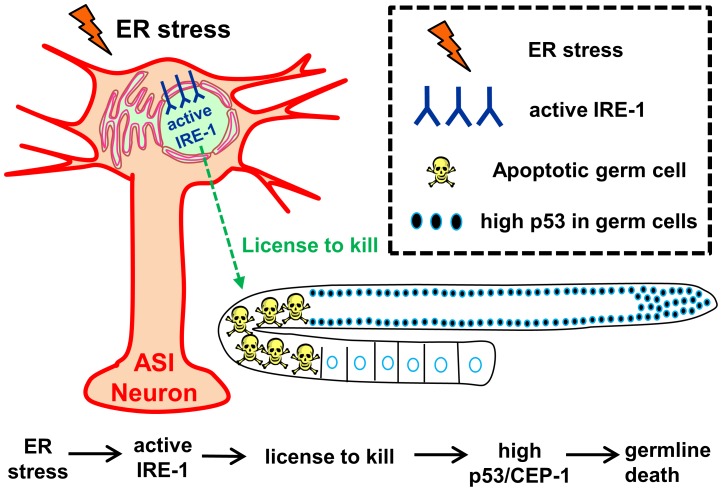
Model – Activation of IRE-1 in the ASI neurons induces germ cell apoptosis. Our data demonstrate that activation of IRE-1 in ASI neurons (by ER stress or simply by its overexpression) is sufficient and required for ER stress-related induction of germline apoptosis. We propose that activation of IRE-1 in the ASI neurons initiates a signaling cascade that “licenses” germ cell apoptosis. This, in turn, increases CEP-1 levels in the germ cells, which triggers germ cell apoptosis using the same genes previously associated with DNA damage induced apoptosis.

We propose that activation of IRE-1 in the neurons (either as a result of ER stress or merely by its over-expression) actively regulates the production of a germ cell regulatory signal. In principle this may be a germ cell proapoptotic signal produced by the neurons upon IRE-1 activation. Alternatively, this may be a germ cell anti-apoptotic signal that is down-regulated by IRE-1 upon its activation. This ASI-regulated signal, whose identity and nature remain to be elucidated, propagates in the animal and affects the gonad where it acts upstream to the p53 homolog *cep-1*, activating the same apoptotic machinery in the germ cells as the DNA damage response, without inducing DNA damage in the germ cells (Figure 6). This indicates the existence of a new pathway that can activate CEP-1 independently of DNA damage upon activation of neuronal IRE-1.

Interestingly, in adult animals, exposure to ER stress or activation of IRE-1 in the soma induce apoptosis only in germ cells, as we did not detect any apoptotic corpses outside of the gonad of these animals. This is in contrast to the developing embryo, where exposure to ER stress can induce apoptosis in the soma [35], [59]. We propose that as the organism completes development, its ability to respond or execute programmed cell death upon exposure to ER stress is maintained in mitotic germ cells while being selectively abrogated in the post-mitotic soma, as has been demonstrated for their ability to execute apoptosis in response to DNA-damage [60]. This resistance of the soma is important in terms of survival of the animal as it prevents cell death of somatic tissues that lack stem cell pools and regenerative capacity, while allowing cell death of immortal germline cells at times of stress.

What could be the advantage in diluting the germ cell pool when neurons “feel” ER-stressed (*i.e.* when IRE-1 is activated naturally by ER stress or artificially by overexpression)? Recent studies demonstrate a tight inverse correlation between germ-cell proliferation and the maintenance of somatic proteostasis and longevity [61]–[65]. This inverse correlation is thought to be due to a limitation of resources shared by the germline and the soma and due to altered metabolic and cellular repair mechanisms in the soma that are enabled upon germ cell loss. Previous studies implicated the nervous system in systemic and hierarchical control of cellular stress responses elsewhere in the soma to maintain organismal homeostasis [66]–[72]. Our data further imply that neurons also have the ability to communicate with the germ cells to promote their death in response to stress in the ER. This, in turn, may orchestrate a proteostasis switch in the soma at the expense of a replenishable germ cell pool in times of stress. This adds a new layer of complexity to our understanding of how protein homeostasis is regulated and coordinated across tissues in multicellular organisms.

## Materials and Methods

### Cell corpse assay

For single time-point experiments, the number of apoptotic germ cells was scored in day-2 animals stained with SYTO12 (Molecular Probes) as previously described [6]. For time course experiments, the number of SYTO12-labeled apoptotic corpses per gonad arm was scored in animals from day-0 (L4) to day-3 of adulthood. Where indicated, the average number of apoptotic corpses was normalized to the number of mitotic germ cells within the proliferative zone of the gonads, determined by section analysis of DAPI-stained gonads.

### Oxidative stress treatment

Day-1 adult animals were placed in 200 µl of M9 (control) or 10 mM paraquat (oxidative stress) for 1.5 h at 20°C. After the incubation period, 1 ml of M9 was added to dilute the paraquat. Animals were then transferred to eppendorfs with SYTO12 staining for 4.5 hrs. Animals were allowed to recover on plates for 40 min. Finally, the animals were mounted and observed under the microscope to determine cell corpse numbers.

### Gonad dissection and DAPI staining

Gonads of day-1 adults were dissected, fixed, and stained with DAPI as previously described [10].

### RNA interference

Bacteria expressing dsRNA were cultured overnight in LB containing tetracycline and ampicillin. Bacteria were seeded on NGM plates containing IPTG and carbenicillin. RNAi clone identity was verified by sequencing. Eggs were placed on plates and synchronized from day-0 (L4).The efficacy of the *tfg-1* RNAi was confirmed by the animals' reduced body size [35].

### Fluorescence microscopy and quantification

Animals were anaesthetized on 2% agarose pads containing 2 mM levamisol.

Images were taken with a CCD digital camera using a Nikon 90i fluorescence microscope. For each trial, exposure time was calibrated to minimize the number of saturated pixels and was kept constant through the experiment. The NIS element software was used to quantify mean fluorescence intensity as measured by intensity of each pixel in the selected area.

### Statistical analysis

Error bars represent the standard error of the mean (SEM) of at least 3 independent experiments. P values were calculated using the unpaired Student's t test.

### Strains and transgenic lines

The following lines were used in this study: N2, CF2012: *pek-1(ok275) X*, CF2988: *atf-6(ok551) X*, CF2473: *ire-1(ok799) II*, CF3208: *xbp-1(tm2457) III*, SHK62: *ire-1(ok799) II; xbp-1(tm2457) III*, MD701: *Plim-7 ced-1::gfp V*, *xbp-1(tm2482) III*, CF2185: *ced-3(n1289) IV*, MT2547: *ced-4(n1162) III*, TJ1: *cep-1(gk138) I*, MT8735: *egl-1(n1084n3082) V*, FX536: *ced-13(tm536) X*, WS1433: *hus-1(op241) I; unc-119(ed3) III; opIs34*, CF2052: *kri-1(ok1251) I*, KU25: *pmk-1(km25) IV*, AU1: *sek-1(ag1) X*, CF3030: *kgb-1(um3) kgb-2(gk361) jnk-1(gk7) IV*, NS2937: *trf-1(nr2014) III*, CF2260: *zcIs4[Phsp-4::gfp] V*, CL2166: *Pgst-4::gfp(dvls19) V*, CF1553: muIs84 [(pAD76) *Psod-3::gfp*+rol-6], SJ4100: *Phsp-6::gfp(zcIs13) V*, CL2070: *Phsp-16.2::gfp(dvls70) V*, SHK57: *xbp-1(tm2457) III; ced-3(n1286) IV*, SHK189: *zcIs4[Phsp-4::gfp] V; Pire-1::ire-1*, NL2098: *rrf-1(pk1417) I*, NL2550: *ppw-1(pk2505) I*, SHK185: *ire-1(ok799) II; Prgef-1::ire-1*, SHK4: *Pire-1::ire-1*, SHK182: *Prgef-1::ire-1*, BB22 *rde-4(ne299) III; adr-2(gv42) III*, TG12: *cep-1(lg12501) I; unc-119(ed4) III*; gtIs1 [CEP-1::GFP+unc-119(+)], SHK8: *Pmyo-3::ire-1*, SHK14: *Pmyo-3::ire-1; ire-1(ok799) II*, *Pser-2::ire-1; ire-1(ok799) II*, SHK15: *Pdaf-28::ire-1; ire-1(ok799) II*, SHK6: *Pdaf-28::ire-1*, SHK237: *Pttx-3::ire-1; Pdaf-7::gfp*, SHK234: *Pdaf-7::ire-1; Pdaf-7::gfp*, *daf-28(tm2308) V*, CF2638: *daf-28(sa191) V*, VB1605: *svls69[Pdaf-28::daf-28::gfp]*, SHK11: *ire-1(ok799) II*; *svls69[Pdaf-28::daf-28::gfp]*, SHK27: *ire-1(ok799) II*; *Pire-1::ire-1; svls69[Pdaf-28::daf-28::gfp]*, SHK60: *unc-13(e51) I*, SK7: *unc-64(e246) III; unc-31(e928) IV* and MT6308: *eat-4 (ky5) III*.

The following strains were used for tissue-specific RNAi experiments: TU3401: *sid-1(pk3321) V; Punc-119::sid-1* (neuron only RNAi), VP303: *rde-1(ne213) V; Pnhx-2::rde-1* (intestine only RNAi), WM118: *rde-1(ne300) V; Pmyo-3::rde-1*(muscle only RNAi), NR222: *rde-1(ne219) V; Plin-26::rde-1* (hypodermis only RNAi), NK640: *rrf-3(pk1426)II; rde-1(ne219) V; Pfos-1A::rde-1* (uterine only RNAi),JK4143: *rde-1(ne219) V*; *Plag-2::rde-1::gfp* (distal tip cell only RNAi).

The following strains were used for neuron-specific RNAi treatments as previously described [73]: XE1581: *wpSi10 II [unc-17p::rde-1::SL2::sid-1+Cbr-unc-119(+)]; eri-1(mg366) IV; rde-1(ne219) V; lin-15B(n744) X* - Cholinergic neuron-specific RNAi strain. XE1375: *wpIs36 I [unc-47p::mCherry]; wpSi1 II [unc-47p::rde-1::SL2::sid-1+Cbr-unc-119(+)]; eri-1(mg366) IV; rde-1(ne219) V; lin-15B(n744) X* - GABAergic neuron-specific RNAi strain. XE1582: *wpSi11 II [eat-4p::rde-1::SL2::sid-1+Cbr-unc-119(+)] II.; eri-1(mg366) IV; rde-1(ne219) V; lin-15B(n744) X* - Glutamatergic neuron-specific RNAi strain. XE1474: *wpSi6 II [dat-1p::rde-1::SL2::sid-1+Cbr-unc-119(+)] II; eri-1(mg366) IV; rde-1(ne219) V; lin-15B(n744) X* - Dopaminergic neuron-specific RNAi strain, SHK231: *sid-1(pk3321) V; Pche-12::sid-1(+); rol-6(su1006)*.

### Plasmid construction

*Prgef-1::ire-1 - ire-1* cDNA was cloned under the 3.5 kb *rgef-1* (*F25B3.3*) promoter and injected at 5 ng/µl with *Pmyo-3::mCherry* at 50 ng/µl.

*Pire-1::ire-1 - ire-1* cDNA was cloned under the 4.5 kb *ire-1 (C41C4.4)* promoter in the L3691 vector and injected at 25 ng/µl with *rol-6* at 100 ng/µl. *Pttx-3::ire-1* - was created by cloning the *ire-1* cDNA into a *Pttx-3* vector [74] using KpnI/SphI.

*Pdaf-7::gfp* and *Pdaf-7::ire-1 - daf-7* promoter fragment [49] was cloned into pPD95.75 (gift from A. Fire, Carnegie Institute) using SphI/XbaI to create *daf-7p::gfp* transcriptional fusion. The *gfp* fragment was then replaced by *ire-1* cDNA using XmaI/AflII to make *daf-7p::ire-1*. *daf-7p::ire-1* or *ttx-3::ire-1* were injected at 10 ng/µl with *daf-7p::gfp* and *pRF4 (rol-6)* at 20 ng/µl each.

*ser-2prom-3::ire-1* - was created by cloning *ire-1* cDNA under *ser-2prom-3* fragment [75] using XmaI/AflII. *ser-2prom-3::ire-1* was injected at 10 ng/µl with *ttx-3::mCherry* at 40 ng/µl.

## Supporting Information

Figure S1 *tfg-1* deficiency specifically activates the ER stress response. Representative fluorescence micrographs (100-fold magnification) of adult transgenic animals expressing a GFP reporter fused to promoters, whose activity is induced in response to a variety of cellular stresses. Animals were treated with control RNAi or with *tfg-1* RNAi. Bar graph presents relative fluorescence +/−SEM of the indicated genotypes (n = 50 animals per genotype). *Phsp-4::gfp* is induced in response to ER stress, *Pgst-4::gfp* and *Psod-3::gfp* are induced in response to oxidative stress, *Phsp-6::gfp* is induced in response to mitochondrial stress and *Phsp-16.2::gfp* is induced in response to heat shock. Asterisk marks Student's t-test value of P<0.001 compared to control RNAi. White Arrows point at *Phsp-4::gfp* induction in the spermatheca. No increase in the levels of any of the cellular stress reporters was seen upon treatment with *tfg-1* RNAi except for the *Phsp-4::gfp* ER stress response reporter.
(TIF)

Figure S2 ER stress does not increase germ cell apoptosis in *ced-3* deficient animals. Representative fluorescence micrographs (400-fold magnification) of SYTO12-stained germ cell corpses in day-2 adults. Arrows point at SYTO12-labeled germ cell corpses. ER stress was induced by inactivation of the UPR gene *xbp-1* or by blocking protein export from the ER by inactivation of *tfg-1* (UPR is constitutively activated in *tfg-1*-deficient animals, see **Figure S1**). No germ cell corpses were detected in either *ced-3(n1286), xbp-1(tm2457); ced-3(n1286)* or *tfg-1(RNAi); ced-3(n1286)* backgrounds. Bar graph shows average number +/−SEM of apoptotic corpses per gonad arm (n = 40 per genotype). Asterisk marks Student's t-test values of P<0.001 compared to *ced-3*-deficient animals.
(TIF)

Figure S3 Effects of different *ire-1* expression levels on the reproductive system. (**A**) Bar graph presents amount of mitotic germ cells per gonad arm scored in DAPI-stained dissected gonads from day-1 adults of the indicated genotypes (n = 50 gonads per genotype). (**B**) Bar graph presents the average number of apoptotic corpses per gonad arm as scored in SYTO12-stained day-2 adults (n = 50 animals per genotype). Note that the *xbp-1* mutation did not significantly increase the levels of apoptotic corpses in the gonads of *ire-1(ok799)* mutants (P = 0.29). Note that *xbp-1* mutants and *xbp-1; ire-1* double mutants have similar amounts of mitotic germ cells (P = 0.072, see panel A). (**C**) Bar graph presents the fold change in the normalized amount of apoptotic corpses per gonad arm compared to wild-type animals. The amount of apoptotic corpses (presented in B) was normalized to the average number of mitotic germ cells in each of the indicated genotypes (presented in A). Asterisk marks Student's t-test values of P<0.001. (**D**) Bar graph presents average progeny number scored in 15 animals per genotype. Asterisk marks Student's t-test value of P<0.001 compared to wild-type animals. Error bars represent SEM. All animals in panel D contained a *daf-28::gfp* transgene in their background.
(TIF)

Figure S4 Uncoupling general neuronal dysfunction and the responsiveness to ER stress. (**A**) Representative fluorescence micrographs (400-fold magnification) of GFP-expressing ASI neurons driven by the *daf-7* promoter. The overall pattern of the ASI neurons was similar in control RNAi and the *tfg-1* RNAi treated animals. (**B–C**) Bar graph and representative fluorescence micrographs (400-fold magnification) of germline corpses in *unc-13(e51)*, *unc-64(e246) unc-31(e928)* and *eat-4 (ky5)* day-2 mutants are presented. The average number of apoptotic corpses per gonad arm was scored by SYTO12 staining (n = 40 animals per genotype). Note that although these strains have a severely defective nervous system, they display normal basal levels of germline apoptosis, which increase in response to ER stress. These results uncouple general neuronal dysfunction and the responsiveness to ER stress-induced germline apoptosis.
(TIF)
