# Correction: It’s All in Your Mind: Determining Germ Cell Fate by Neuronal IRE-1 in *C*. *elegans*

**DOI:** 10.1371/journal.pgen.1011061

**Published:** 2023-11-30

**Authors:** Mor Levi-Ferber, Yehuda Salzberg, Modi Safra, Anat Haviv-Chesner, Hannes E. Bülow, Sivan Henis-Korenblit

After this article [1] was published, concerns were raised about Figs 2, 4, S1, S2, and S4. Specifically:

- The control RNAi/*eat-4(-)* panel in S4C Fig is duplicated as the control RNAi/*daf-28(tm2308)* panel in Fig 4D.
- The right side of the *tfg-1 (RNAi)*/*Phsp-16*.*2*::*gfp* panel in S1 Fig is duplicated as the left side of the control RNAi/*Phsp-16*.*2*::*gfp* panel in S1 Fig.
- The wild type panel in Fig 2A is duplicated as the wild type panel in S2 Fig.

In response to queries about the experiments in Figs 4D, S1, and S4C in [1], the corresponding author stated that the control RNAi/*eat-4(-)* panel in S4C Fig was duplicated in error and the control RNAi/*daf-28(tm2308)* panel in Fig 4D is incorrect. They also stated that the *tfg-1 (RNAi)*/*Phsp-16*.*2*::*gfp* panel in S1 Fig was duplicated in error and the control RNAi/*Phsp-16*.*2*::*gfp* panel in S1 Fig is incorrect.

In response to queries about the experiments in Figs 2 and S2 in [1], the corresponding author stated that the images for Figs 2A and S2 (which controls for Fig 2A) were done in parallel, hence the common image.

The corresponding author provided the below updated versions of Figs 2A, 4D, S1, S2 and S4B and S4C generated from three recent biological replicate experiments. They also provided the underlying data for the replicate experiments used to generate the updated Figs 2A, 4D, S1, S2 and S4B and S4C in Files S1–S6. Due to the generation of new data from the replicate experiments, the corresponding author provided updates to the number of animals examined and the statistical values in the legends for the updated Figs 2, 4, S1, S2 and S4.

In the text, sentences four to six in paragraph four of the ER stress in the ASI sensory neurons regulates germ cell apoptosis cell non-autonomously section have been updated, with new P values, to ‘We found that germ cell apoptosis in the gonads of *daf-28(sa191)* mutants was increased by approximately 4 fold compared to wild-type animals (Fig 4D). Importantly, germ cell apoptosis was not increased in a *daf-28(tm2308)* null strain, which is deficient in *daf-28* and does not produce the toxic insulin peptide which induces ER stress (Fig 4D). *tfg-1* RNAi treatment of the two *daf-28* mutant strains increased the number of germline corpses in *daf-28(tm2308)* null strain (P<0.05), but did not further increase germline apoptosis in the *daf-28(sa191)* strain (P=0.961, Fig 4D).’

An expert Academic Editor reviewed the updated Figures and underlying data for the replicate experiments and stated that the conclusions in [1] are still supported.

The corresponding author stated that, with the exception of some data underlying S1 Fig, the primary data underlying this article [1] are no longer available. At the time of the article’s submission, PLOS policy required that underlying data should be available without restriction, but *PLOS Genetics* did not yet, as it now does, strictly require publication or deposition of primary data.

**Fig 2:**
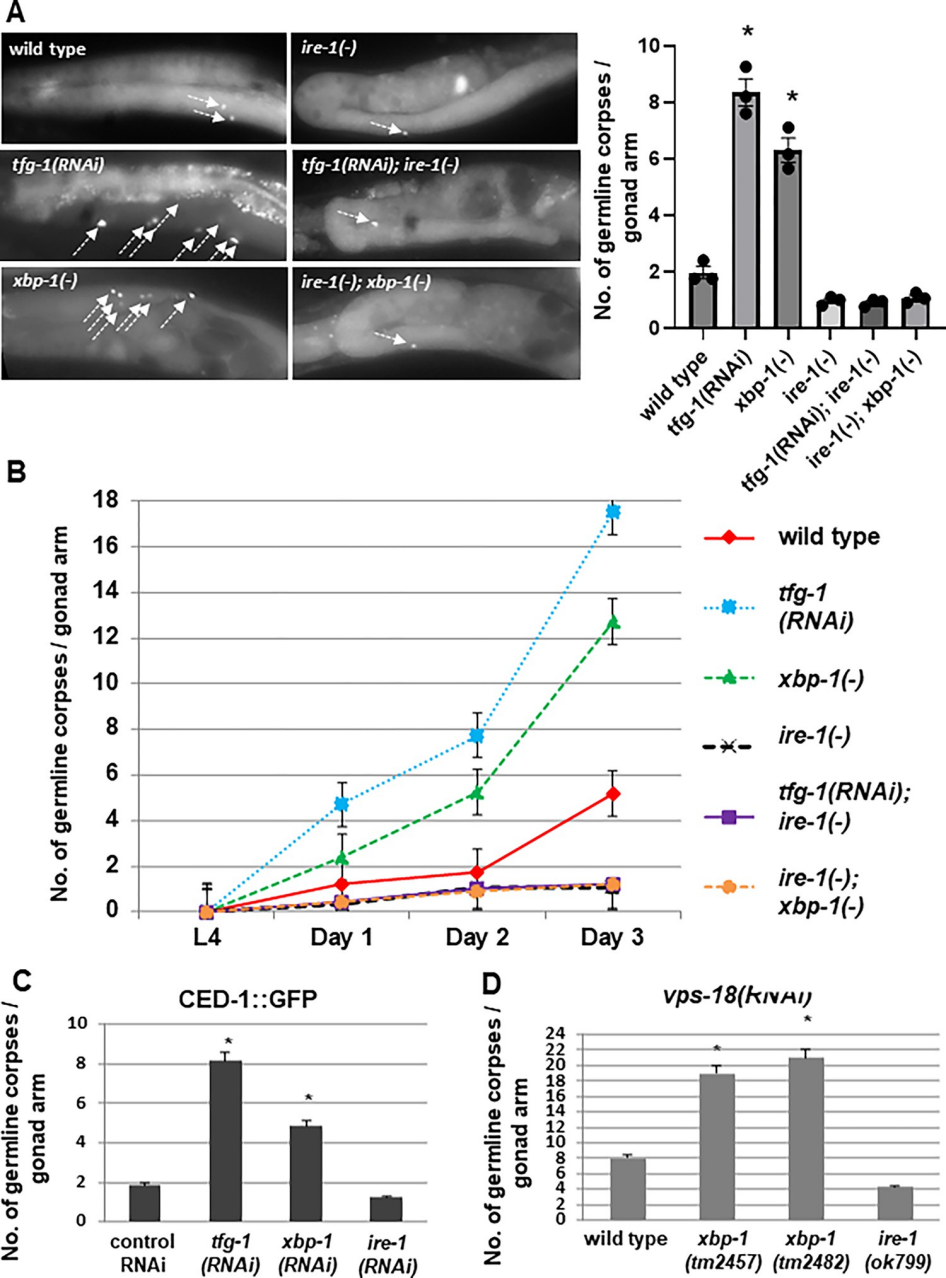
Genetically-induced ER stress increases germ cell apoptosis in an *ire-1*-dependent manner. (A-B) Animals of the indicated genotypes were treated with control or *tfg-1* RNAi. Germ cell corpses were identified by SYTO12 staining. **(A)** Representative fluorescence micrographs (200-fold magnification) of SYTO12-stained germ cell corpses in day-2 adults. Left panels present gonads of wild-type, *xbp-1(tm2457)* or *tfg-1* RNAi treated animals. Right panels present gonads of the corresponding genotypes in an *ire-1(ok799)* background. Arrows point at SYTO12-stained germ cell corpses. Bar graph shows average number of SYTO12-labeled apoptotic corpses per gonad arm in day-2 animals (n = 60 gonads per genotype). Asterisk indicates a significant increase in germline apoptosis compared to wild-type animals) One-way ANOVA followed by Tukey’s multiple comparisons test of P<0.05). **(B)** Time course analysis of cell corpses in the adult gonads of the indicated genotypes. SYTO12-labeled cell corpses were scored at L4, day 1, day 2 and day 3 of adulthood. Each point represents the mean number of cell corpses scored in 30 gonads. Error bars indicate SEM. **(C)** Animals expressing CED-1::GFP were treated with control, *tfg-1*, *xbp-1* or *ire-1* RNAi and analyzed. Bar graph shows average number of apoptotic corpses per gonad arm in the indicated genotypes identified by their engulfment by CED-1::GFP labeled gonadal sheath cells. Asterisk indicates a significant increase in germline apoptosis compared to control RNAi-treated animals (Student’s t-test values of P<0.001). **(D)** Bar graph shows average number of SYTO12-labeled apoptotic corpses per gonad arm in day-2 *vps-18* RNAi-treated animals (n = 40 gonads per genotype). Asterisk indicates a significant increase in germline apoptosis compared to wild-type animals treated with *vps-18* RNAi) Student’s t-test values of P<0.001).

**Fig 4:**
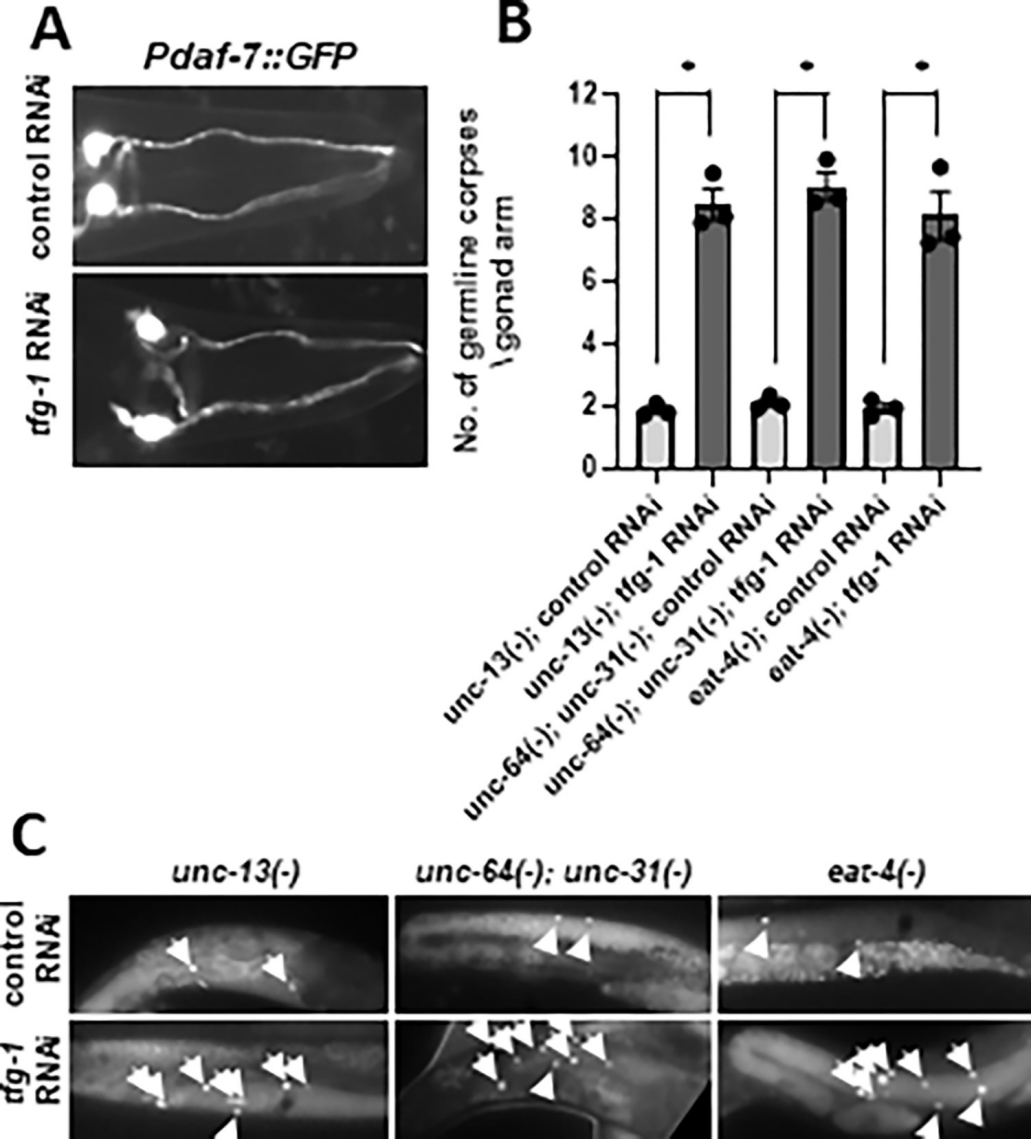
ER stress specifically in sensory neurons is sufficient to induce germline apoptosis. **(A)** Bar graph and representative fluorescence micrographs (400-fold magnification) of SYTO12-stained germ cell corpses in day-2 adults. Arrows point at SYTO12 stained germ cell corpses. Bar graph presents average number +/-SEM of apoptotic corpses per gonad arm in the indicated genotypes (n = 60 per genotype). Asterisks mark Student’s t-test values of P<0.001 of *tfg-1* RNAi treated animals compared to the corresponding control RNAi treated animals. Note that in animals in which RNAi functions only in the neurons, *tfg-1* RNAi increased the amount of germ cell corpses to a greater extent than in *ppw-1* mutants or wild-type animals. This may be due to more efficient *tfg-1* RNAi uptake in the neurons of these animals, whose neurons over-express SID-1 [76] **(B,C)** Bar graph presents average number +/- SEM of SYTO-12-labeled germ cell corpses of day-2 adults of the indicated genotypes (n = 60 gonads per genotype). Asterisks mark Student’s t-test values of P<0.001 of *tfg-1* RNAi treated animals (black bars) compared to the corresponding control RNAi treated animals (white bars). See materials and methods for strains details. **(D)** Representative fluorescence micrographs (200-fold magnification) and bar graph of SYTO12-stained germ cell corpses in day-2 *daf-28* mutant strains treated with control RNAi or *tfg-1* RNAi. Bar graph presents average number +/- SEM of apoptotic corpses per gonad arm in the indicated genotypes (n = 60 per genotype). *tm2308* is a deletion mutation in the *daf-28* gene. *sa191* is a point mutation that interferes with the posttranslational processing of DAF-28. Asterisk marks a significant increase in germline apoptosis (One-way ANOVA followed by Šidák multiple comparisons test of P<0.05).

## Supporting information

S1 File Combined underlying data for the updated Figs 2A, 4D, S2, S4B and S4C. This file contains all the scoring data of the revised SYTO12 staining experiments. Each of the three biological replicates for Figs 2A, 4D, S2 and S4B–S4C were done in parallel in all strains. Hence, the wild type animals in Fig 2A and in S2 Fig are the same. In most cases animals were scored by live imaging without accompanied image acquisition. Representative images are provided. Consecutive images may image the same gonad. The scoring of apoptotic corpses was performed per gonad, not per image.
(ZIP)

S2 File Underlying data for the updated Fig 2A. (ZIP)

S3 File Underlying data for the updated Fig 4D. (ZIP)

S4 File Underlying data for the updated S1 Fig. Since images include multiple worms, images were independently measured several times to achieve measurements for distinct worms in one shared image. Only animals whose body was fully imaged (at least from front to back intestine) were measured. Exposure levels were maintained constant per worm strain.
(ZIP)

S5 File Underlying data for the updated S2 Fig. (ZIP)

S6 File Underlying data for the updated S4B and S4C Fig. (ZIP)

S1 Fig *tfg-1* deficiency specifically activates the ER stress response. Representative fluorescence micrographs (100-fold magnification) of adult transgenic animals expressing a GFP reporter fused to promoters, whose activity is induced in response to a variety of cellular stresses. Animals were treated with control RNAi or with *tfg-1* RNAi. Bar graph presents relative fluorescence +/-SEM of the indicated genotypes (n = 75 animals per genotype). *Phsp-4*::*gfp* is induced in response to ER stress, *Pgst-4*::*gfp* and *Psod-3*::*gfp* are induced in response to oxidative stress, *Phsp-6*::*gfp* is induced in response to mitochondrial stress and *Phsp-16*.*2*::*gfp* is induced in response to heat shock. Asterisk marks One sample-test value of P<0.05 compared to control RNAi. No increase in the levels of any of the cellular stress reporters was seen upon treatment with *tfg-1* RNAi except for the *Phsp-4*::*gfp* ER stress response reporter.
(TIF)

S2 Fig ER stress does not increase germ cell apoptosis in *ced-3* deficient animals. Representative fluorescence micrographs (200-fold magnification) of SYTO12-stained germ cell corpses in day-2 adults. Arrows point at SYTO12-labeled germ cell corpses. ER stress was induced by inactivation of the UPR gene *xbp-1* or by blocking protein export from the ER by inactivation of *tfg-1* (UPR is constitutively activated in *tfg-1*-deficient animals, see **S1 Fig**). No germ cell corpses were detected in either *ced-3(n1286)*, *xbp-1(tm2457); ced-3(n1286)* or *tfg-1(RNAi); ced-3(n1286)* backgrounds. Bar graph shows average number +/-SEM of apoptotic corpses per gonad arm (n = 60 per genotype). Asterisk marks One-way ANOVA followed by Tukey’s multiple comparisons test values of P<0.05 compared to *ced-3*-deficient animals.
(TIF)

S4 Fig Uncoupling general neuronal dysfunction and the responsiveness to ER stress. **(A)** Representative fluorescence micrographs (400-fold magnification) of GFP-expressing ASI neurons driven by the *daf-7* promoter. The overall pattern of the ASI neurons was similar in control RNAi and the *tfg-1* RNAi treated animals. **(B-C)** Bar graph and representative fluorescence micrographs (200-fold magnification) of germline corpses in *unc-13(e51)*, *unc-64(e246) unc-31(e928)* and *eat-4 (ky5)* day-2 mutants are presented. The average number of apoptotic corpses per gonad arm was scored by SYTO12 staining (n = 60 animals per genotype). Asterisk marks One-way ANOVA followed by Šidák multiple comparisons test values of P<0.05. Error bars represent SEM +/-. Note that although these strains have a severely defective nervous system, they display normal basal levels of germline apoptosis, which increase in response to ER stress. These results uncouple general neuronal dysfunction and the responsiveness to ER stress-induced germline apoptosis.
(TIF)
