# Supplementary figures and images for: It's All in Your Mind: Determining Germ Cell Fate by Neuronal IRE-1 in C. elegans
Source: PLoS Genet. 2014 Oct 23;10(10):e1004747. doi: 10.1371/journal.pgen.1004747 (PMC4207656; doi:10.1371/journal.pgen.1004747)

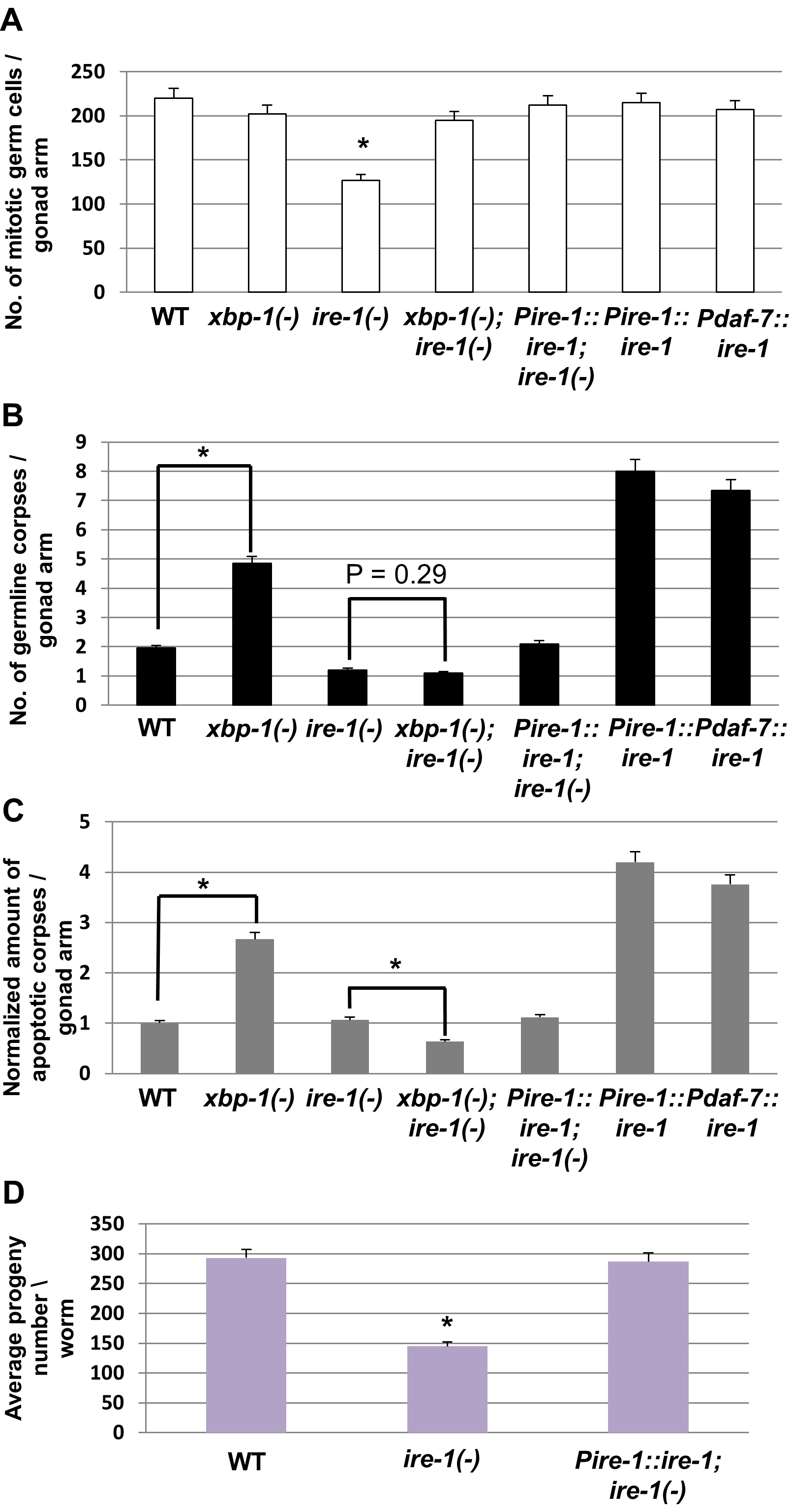

Supplement: Figure S3 — Effects of different ire-1 expression levels on the reproductive system. (A) Bar graph presents amount of mitotic germ cells per gonad arm scored in DAPI-stained dissected gonads from day-1 adults of the indicated genotypes (n = 50 gonads per genotype). (B) Bar graph presents the average number of apoptotic corpses per gonad arm as scored in SYTO12-stained day-2 adults (n = 50 animals per genotype). Note that the xbp-1 mutation did not significantly increase the levels of apoptotic corpses in the gonads of ire-1(ok799) mutants (P = 0.29). Note that xbp-1 mutants and xbp-1; ire-1 double mutants have similar amounts of mitotic germ cells (P = 0.072, see panel A). (C) Bar graph presents the fold change in the normalized amount of apoptotic corpses per gonad arm compared to wild-type animals. The amount of apoptotic corpses (presented in B) was normalized to the average number of mitotic germ cells in each of the indicated genotypes (presented in A). Asterisk marks Student's t-test values of P<0.001. (D) Bar graph presents average progeny number scored in 15 animals per genotype. Asterisk marks Student's t-test value of P<0.001 compared to wild-type animals. Error bars represent SEM. All animals in panel D contained a daf-28::gfp transgene in their background. (TIF) [file pgen.1004747.s003.tif]
