# Supplementary figures and images for: Correction: It’s All in Your Mind: Determining Germ Cell Fate by Neuronal IRE-1 in C. elegans (part 2 of 7)
Source: PLoS Genet. 2023 Nov 30;19(11):e1011061. doi: 10.1371/journal.pgen.1011061 (PMC10688620; doi:10.1371/journal.pgen.1011061)

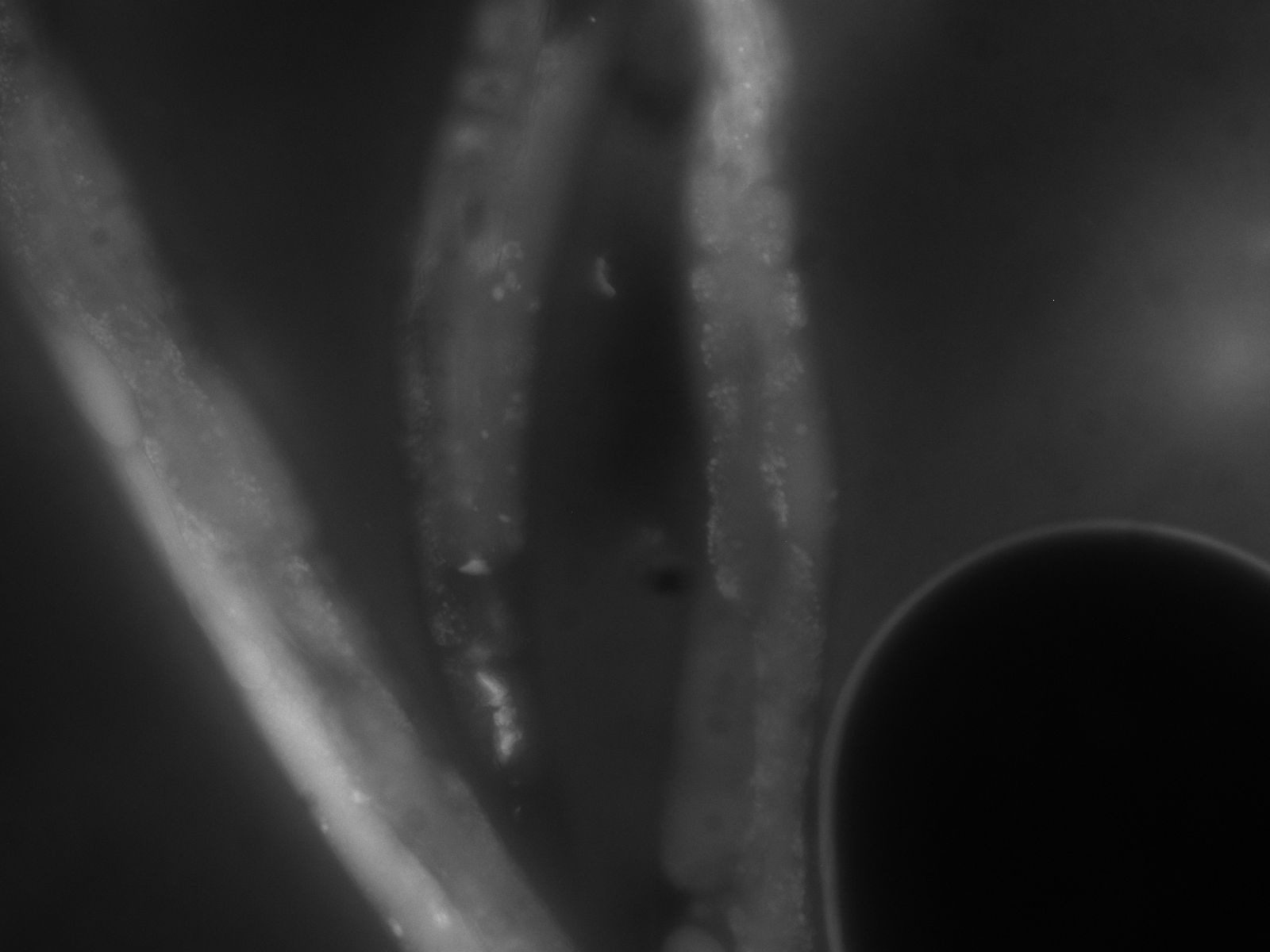

Supplement: S1 File — This file contains all the scoring data of the revised SYTO12 staining experiments. Each of the three biological replicates for Figs 2A, 4D, S2 and S4B–S4C were done in parallel in all strains. Hence, the wild type animals in Fig 2A and in S2 Fig are the same. In most cases animals were scored by live imaging without accompanied image acquisition. Representative images are provided. Consecutive images may image the same gonad. The scoring of apoptotic corpses was performed per gonad, not per image. (ZIP) [file pgen.1011061.s001.zip › SYTO staining experiment united/syto12 staining - 1_rep - 14.5.23 - JPEG/daf-28_tm2308+ tfg-1211.jpg]

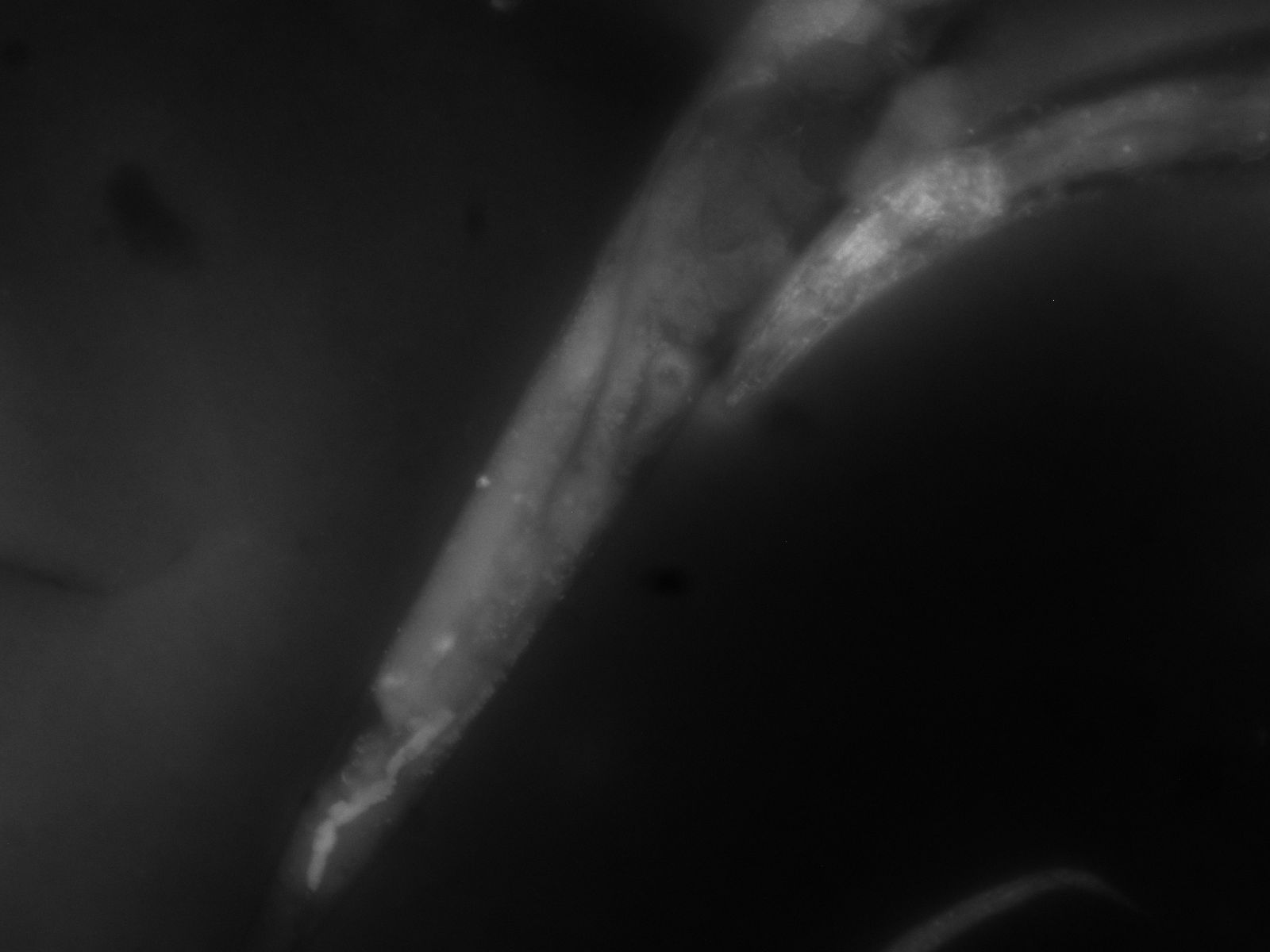

Supplement: S1 File — This file contains all the scoring data of the revised SYTO12 staining experiments. Each of the three biological replicates for Figs 2A, 4D, S2 and S4B–S4C were done in parallel in all strains. Hence, the wild type animals in Fig 2A and in S2 Fig are the same. In most cases animals were scored by live imaging without accompanied image acquisition. Representative images are provided. Consecutive images may image the same gonad. The scoring of apoptotic corpses was performed per gonad, not per image. (ZIP) [file pgen.1011061.s001.zip › SYTO staining experiment united/syto12 staining - 1_rep - 14.5.23 - JPEG/daf-28_tm2308+ tfg-1212.jpg]

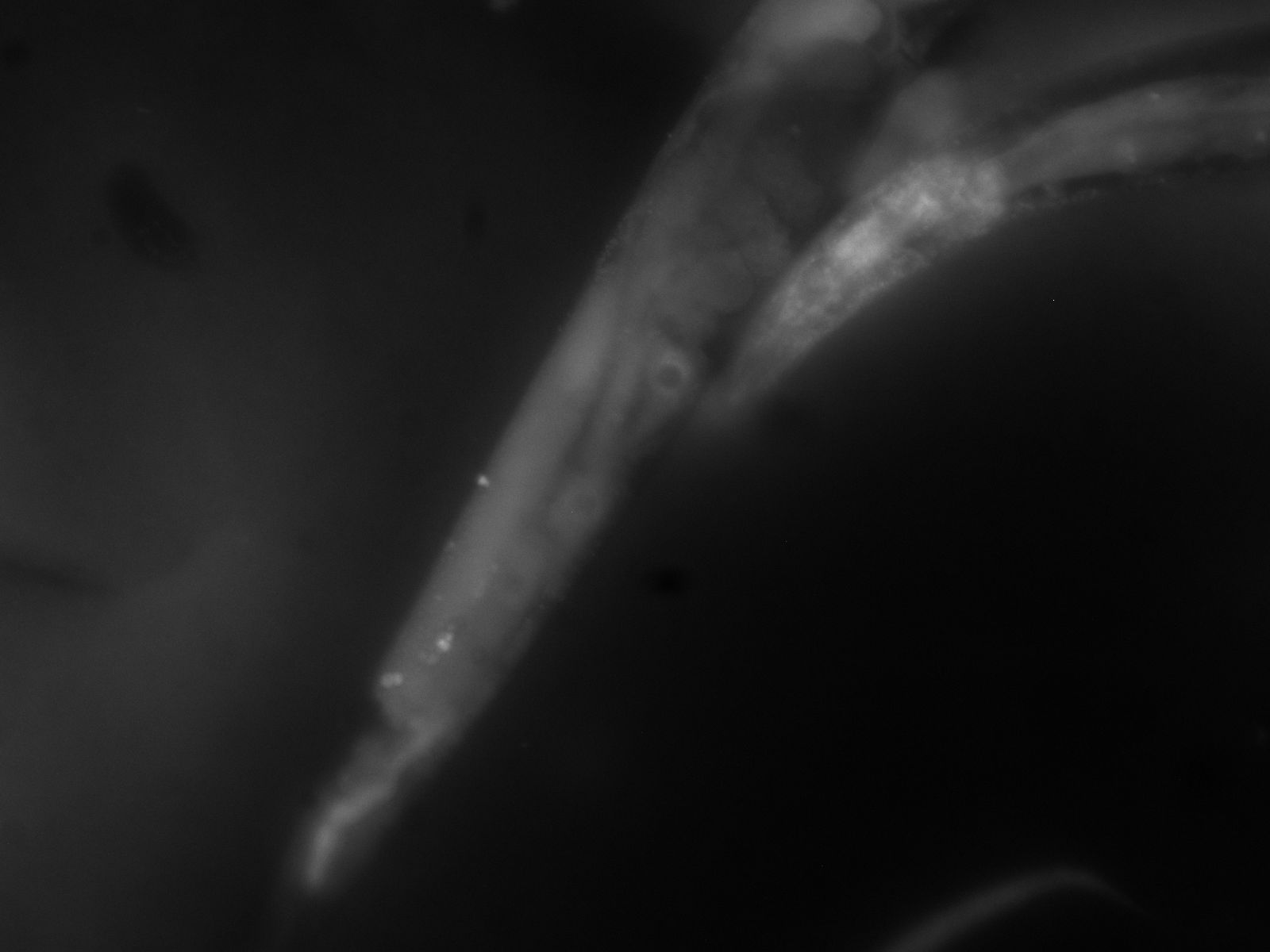

Supplement: S1 File — This file contains all the scoring data of the revised SYTO12 staining experiments. Each of the three biological replicates for Figs 2A, 4D, S2 and S4B–S4C were done in parallel in all strains. Hence, the wild type animals in Fig 2A and in S2 Fig are the same. In most cases animals were scored by live imaging without accompanied image acquisition. Representative images are provided. Consecutive images may image the same gonad. The scoring of apoptotic corpses was performed per gonad, not per image. (ZIP) [file pgen.1011061.s001.zip › SYTO staining experiment united/syto12 staining - 1_rep - 14.5.23 - JPEG/daf-28_tm2308+ tfg-1213.jpg]

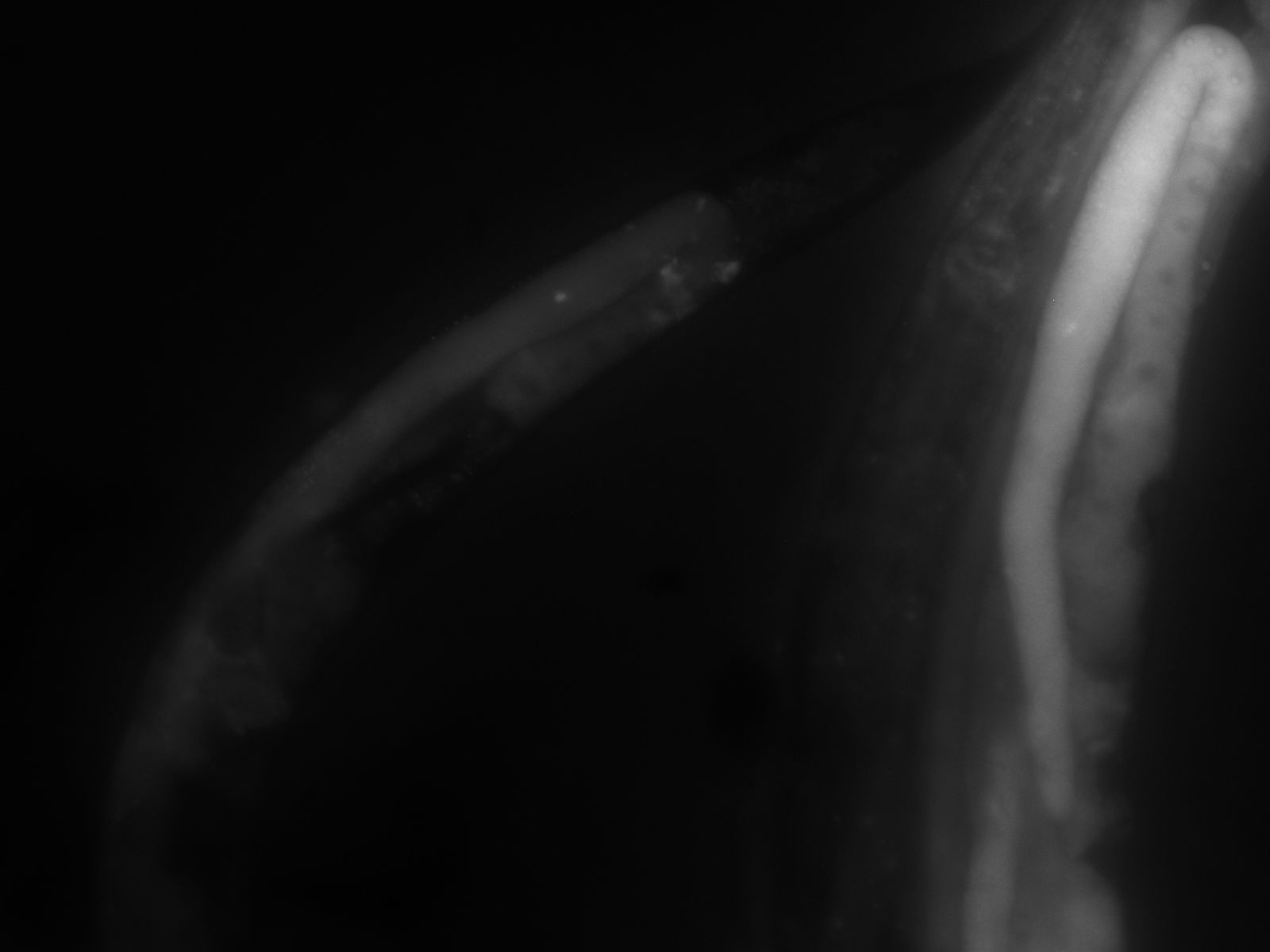

Supplement: S1 File — This file contains all the scoring data of the revised SYTO12 staining experiments. Each of the three biological replicates for Figs 2A, 4D, S2 and S4B–S4C were done in parallel in all strains. Hence, the wild type animals in Fig 2A and in S2 Fig are the same. In most cases animals were scored by live imaging without accompanied image acquisition. Representative images are provided. Consecutive images may image the same gonad. The scoring of apoptotic corpses was performed per gonad, not per image. (ZIP) [file pgen.1011061.s001.zip › SYTO staining experiment united/syto12 staining - 1_rep - 14.5.23 - JPEG/daf-28_tm2308+ tfg-1214.jpg]

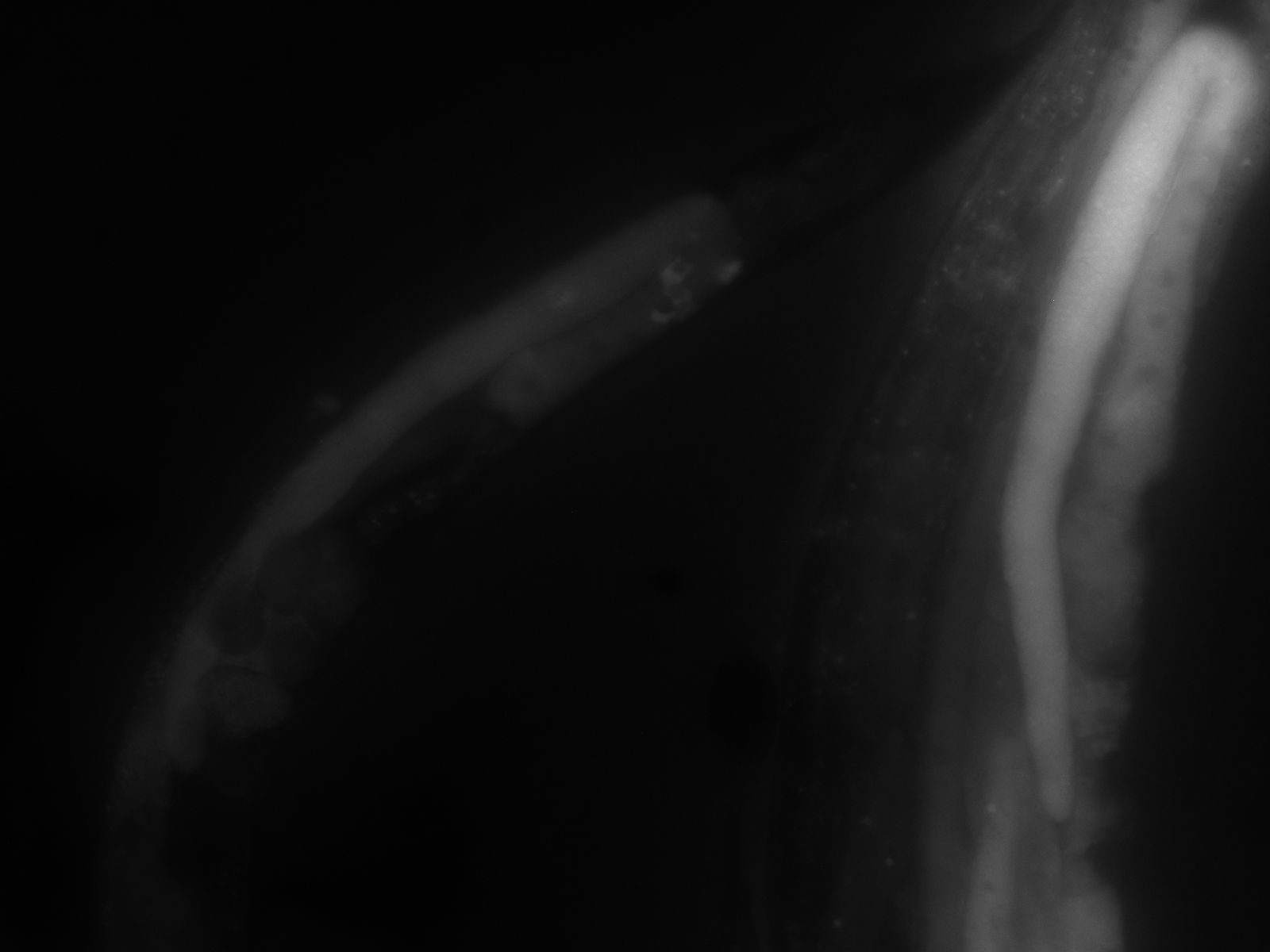

Supplement: S1 File — This file contains all the scoring data of the revised SYTO12 staining experiments. Each of the three biological replicates for Figs 2A, 4D, S2 and S4B–S4C were done in parallel in all strains. Hence, the wild type animals in Fig 2A and in S2 Fig are the same. In most cases animals were scored by live imaging without accompanied image acquisition. Representative images are provided. Consecutive images may image the same gonad. The scoring of apoptotic corpses was performed per gonad, not per image. (ZIP) [file pgen.1011061.s001.zip › SYTO staining experiment united/syto12 staining - 1_rep - 14.5.23 - JPEG/daf-28_tm2308+ tfg-1215.jpg]

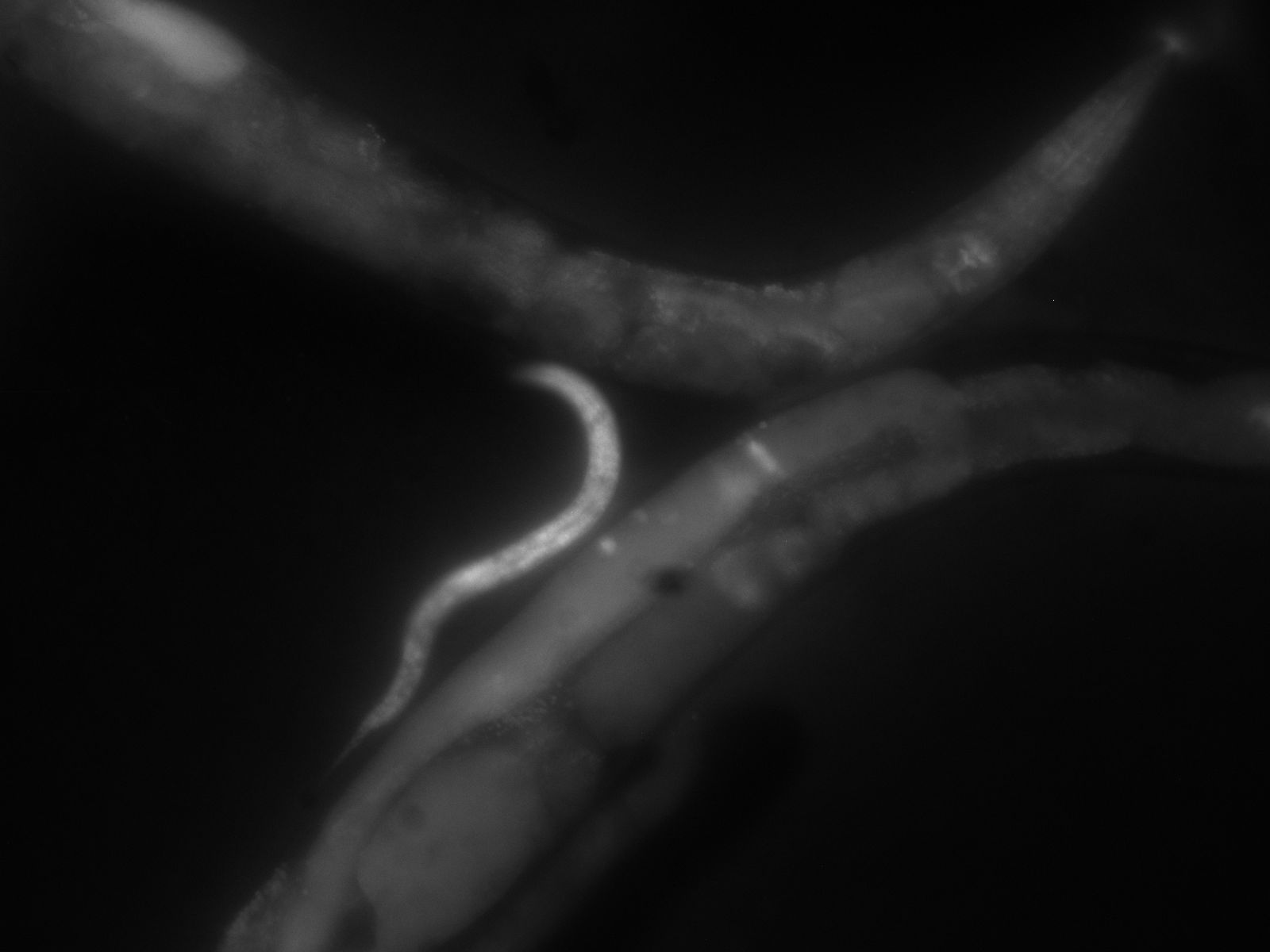

Supplement: S1 File — This file contains all the scoring data of the revised SYTO12 staining experiments. Each of the three biological replicates for Figs 2A, 4D, S2 and S4B–S4C were done in parallel in all strains. Hence, the wild type animals in Fig 2A and in S2 Fig are the same. In most cases animals were scored by live imaging without accompanied image acquisition. Representative images are provided. Consecutive images may image the same gonad. The scoring of apoptotic corpses was performed per gonad, not per image. (ZIP) [file pgen.1011061.s001.zip › SYTO staining experiment united/syto12 staining - 1_rep - 14.5.23 - JPEG/daf-28_tm2308+ tfg-1216.jpg]

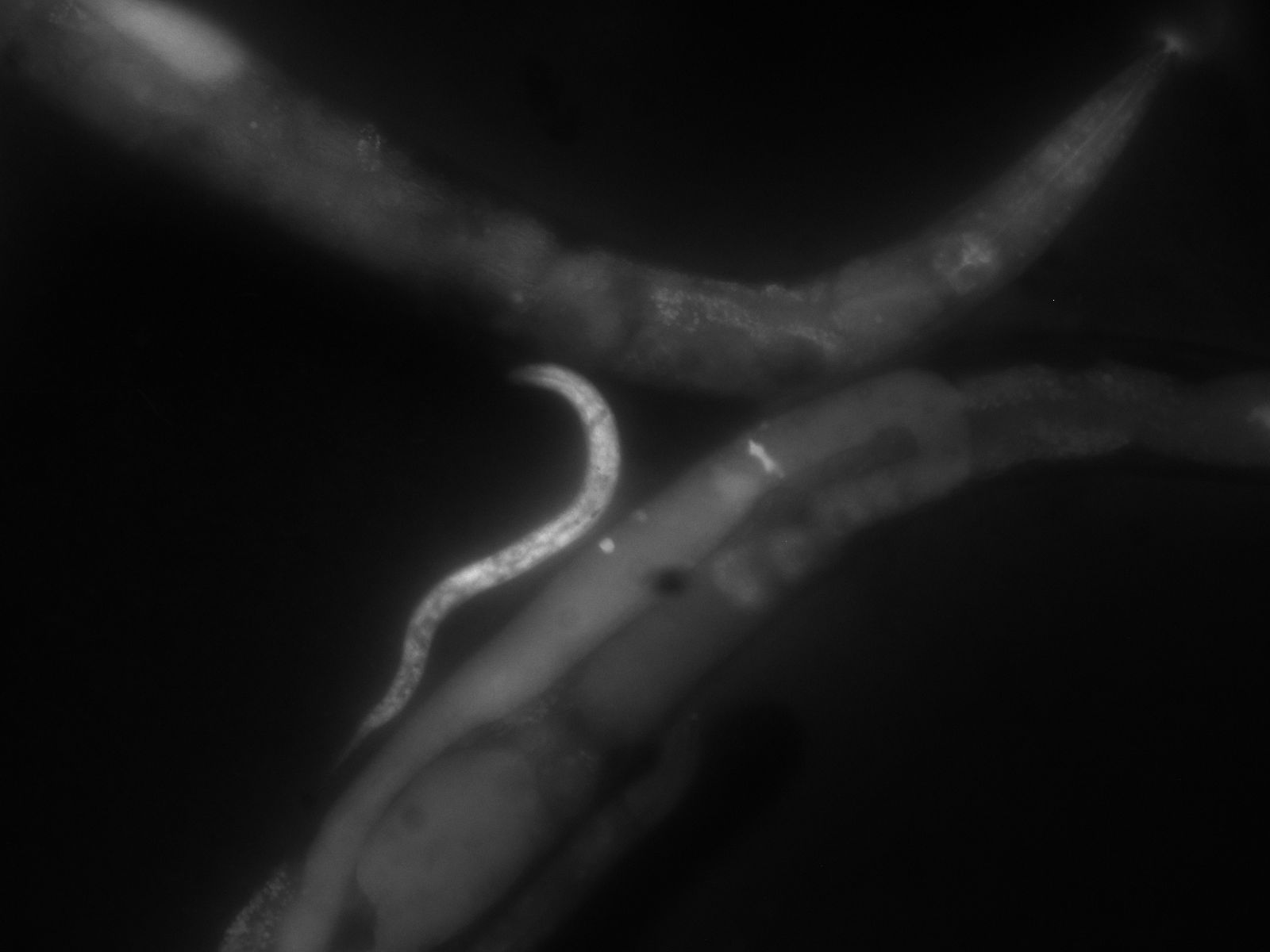

Supplement: S1 File — This file contains all the scoring data of the revised SYTO12 staining experiments. Each of the three biological replicates for Figs 2A, 4D, S2 and S4B–S4C were done in parallel in all strains. Hence, the wild type animals in Fig 2A and in S2 Fig are the same. In most cases animals were scored by live imaging without accompanied image acquisition. Representative images are provided. Consecutive images may image the same gonad. The scoring of apoptotic corpses was performed per gonad, not per image. (ZIP) [file pgen.1011061.s001.zip › SYTO staining experiment united/syto12 staining - 1_rep - 14.5.23 - JPEG/daf-28_tm2308+ tfg-1217.jpg]

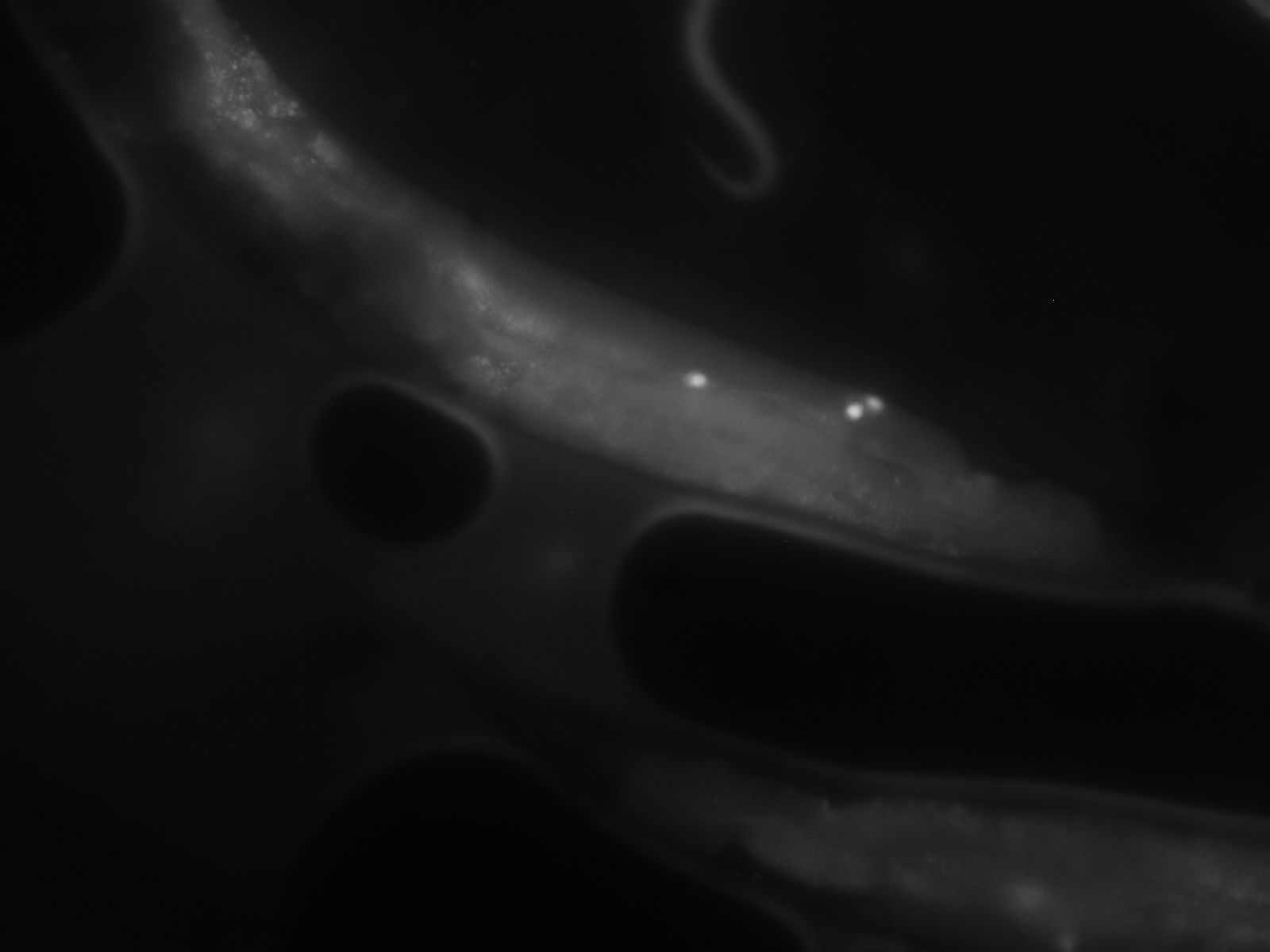

Supplement: S1 File — This file contains all the scoring data of the revised SYTO12 staining experiments. Each of the three biological replicates for Figs 2A, 4D, S2 and S4B–S4C were done in parallel in all strains. Hence, the wild type animals in Fig 2A and in S2 Fig are the same. In most cases animals were scored by live imaging without accompanied image acquisition. Representative images are provided. Consecutive images may image the same gonad. The scoring of apoptotic corpses was performed per gonad, not per image. (ZIP) [file pgen.1011061.s001.zip › SYTO staining experiment united/syto12 staining - 1_rep - 14.5.23 - JPEG/eat-4+pad12251.jpg]

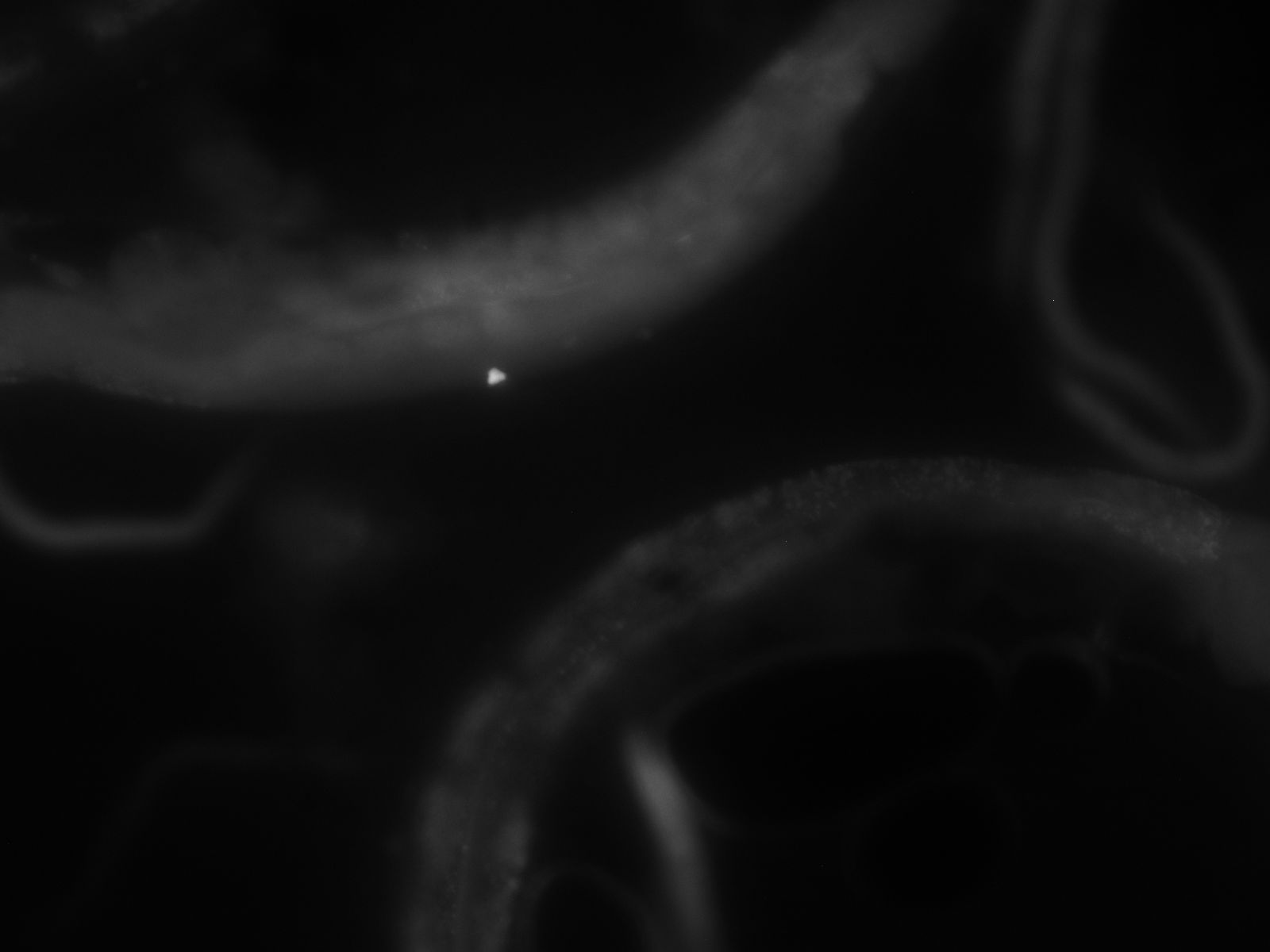

Supplement: S1 File — This file contains all the scoring data of the revised SYTO12 staining experiments. Each of the three biological replicates for Figs 2A, 4D, S2 and S4B–S4C were done in parallel in all strains. Hence, the wild type animals in Fig 2A and in S2 Fig are the same. In most cases animals were scored by live imaging without accompanied image acquisition. Representative images are provided. Consecutive images may image the same gonad. The scoring of apoptotic corpses was performed per gonad, not per image. (ZIP) [file pgen.1011061.s001.zip › SYTO staining experiment united/syto12 staining - 1_rep - 14.5.23 - JPEG/eat-4+pad12252.jpg]

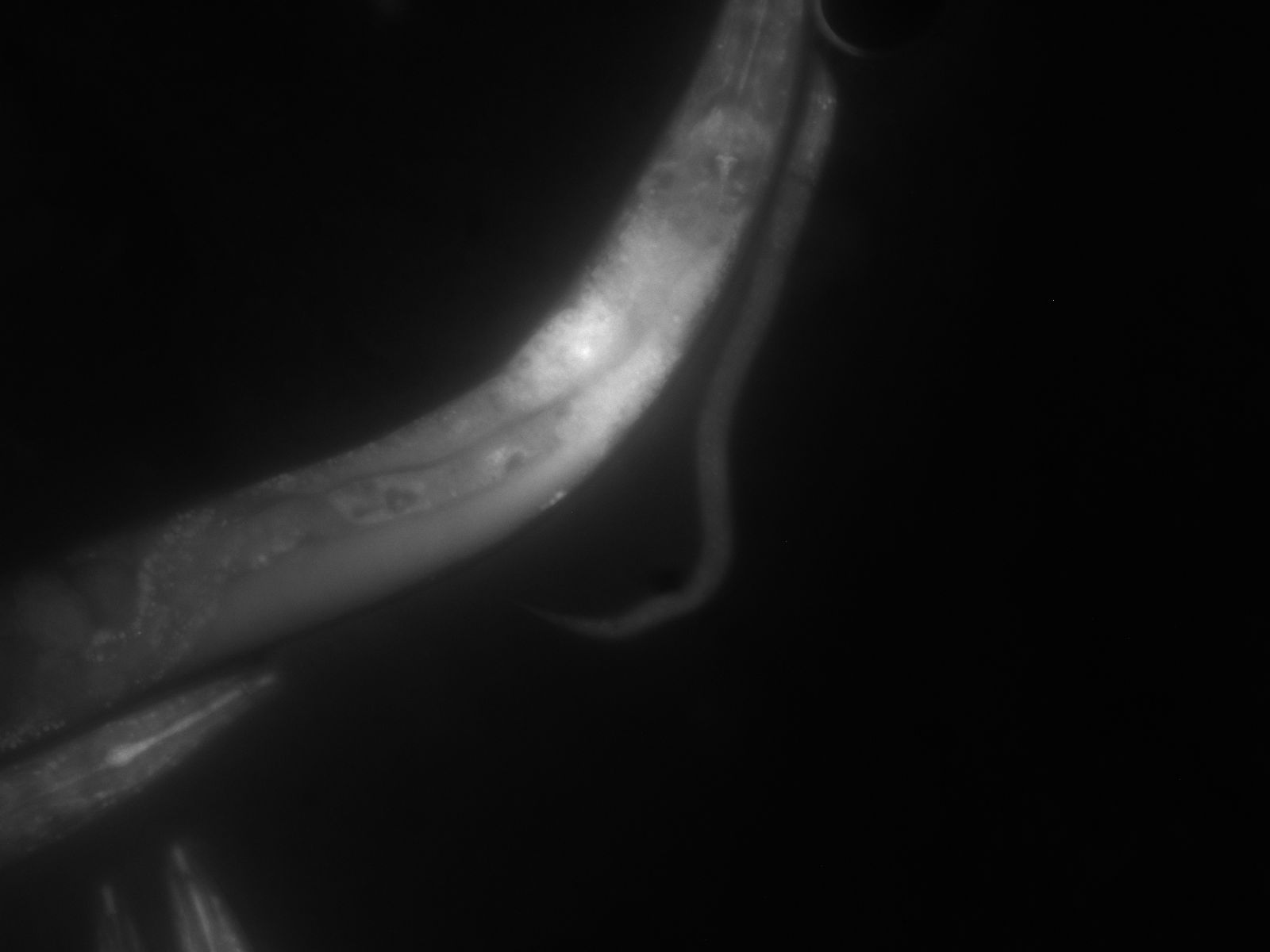

Supplement: S1 File — This file contains all the scoring data of the revised SYTO12 staining experiments. Each of the three biological replicates for Figs 2A, 4D, S2 and S4B–S4C were done in parallel in all strains. Hence, the wild type animals in Fig 2A and in S2 Fig are the same. In most cases animals were scored by live imaging without accompanied image acquisition. Representative images are provided. Consecutive images may image the same gonad. The scoring of apoptotic corpses was performed per gonad, not per image. (ZIP) [file pgen.1011061.s001.zip › SYTO staining experiment united/syto12 staining - 1_rep - 14.5.23 - JPEG/eat-4+pad12253.jpg]

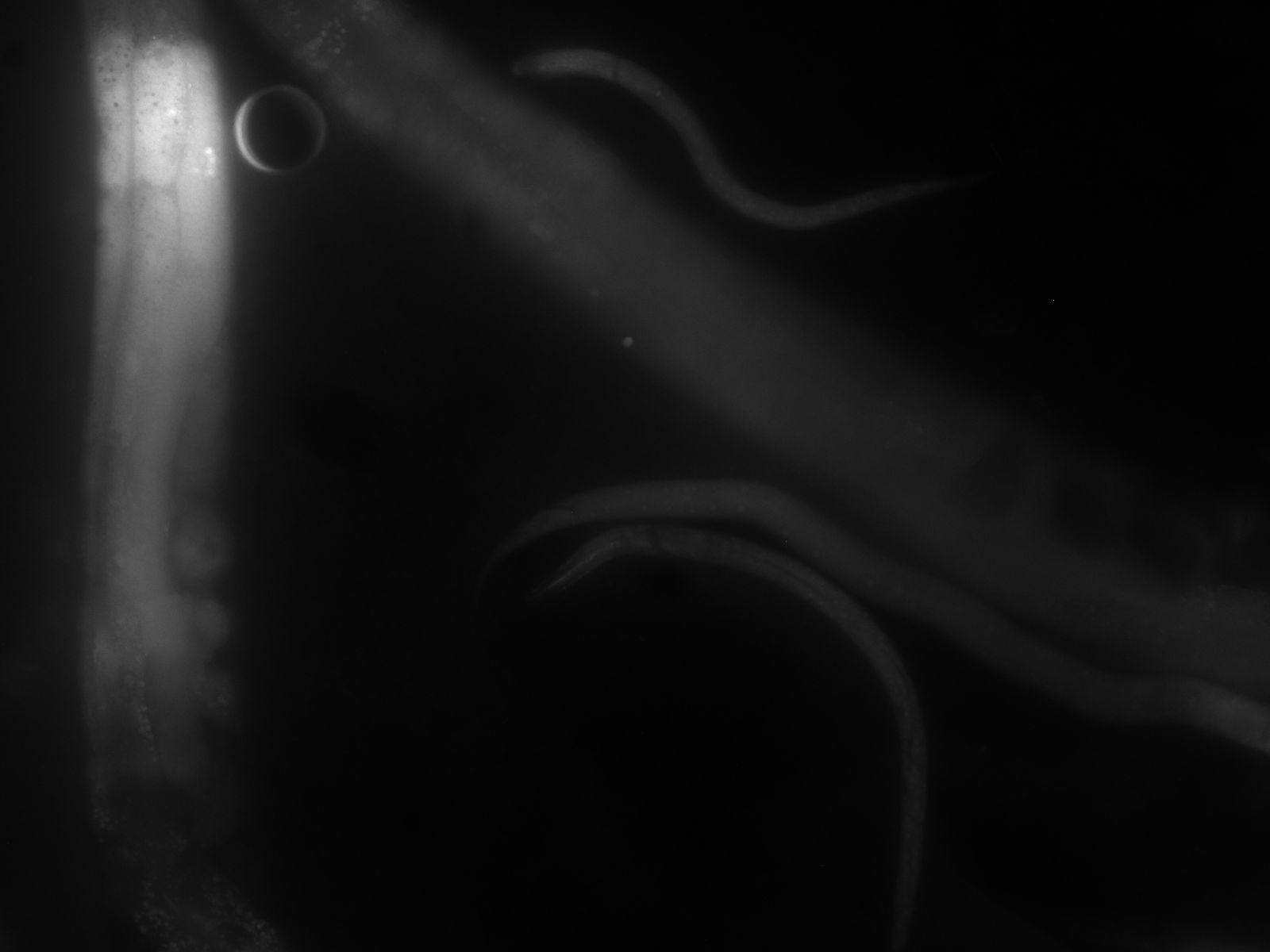

Supplement: S1 File — This file contains all the scoring data of the revised SYTO12 staining experiments. Each of the three biological replicates for Figs 2A, 4D, S2 and S4B–S4C were done in parallel in all strains. Hence, the wild type animals in Fig 2A and in S2 Fig are the same. In most cases animals were scored by live imaging without accompanied image acquisition. Representative images are provided. Consecutive images may image the same gonad. The scoring of apoptotic corpses was performed per gonad, not per image. (ZIP) [file pgen.1011061.s001.zip › SYTO staining experiment united/syto12 staining - 1_rep - 14.5.23 - JPEG/eat-4+pad12254.jpg]

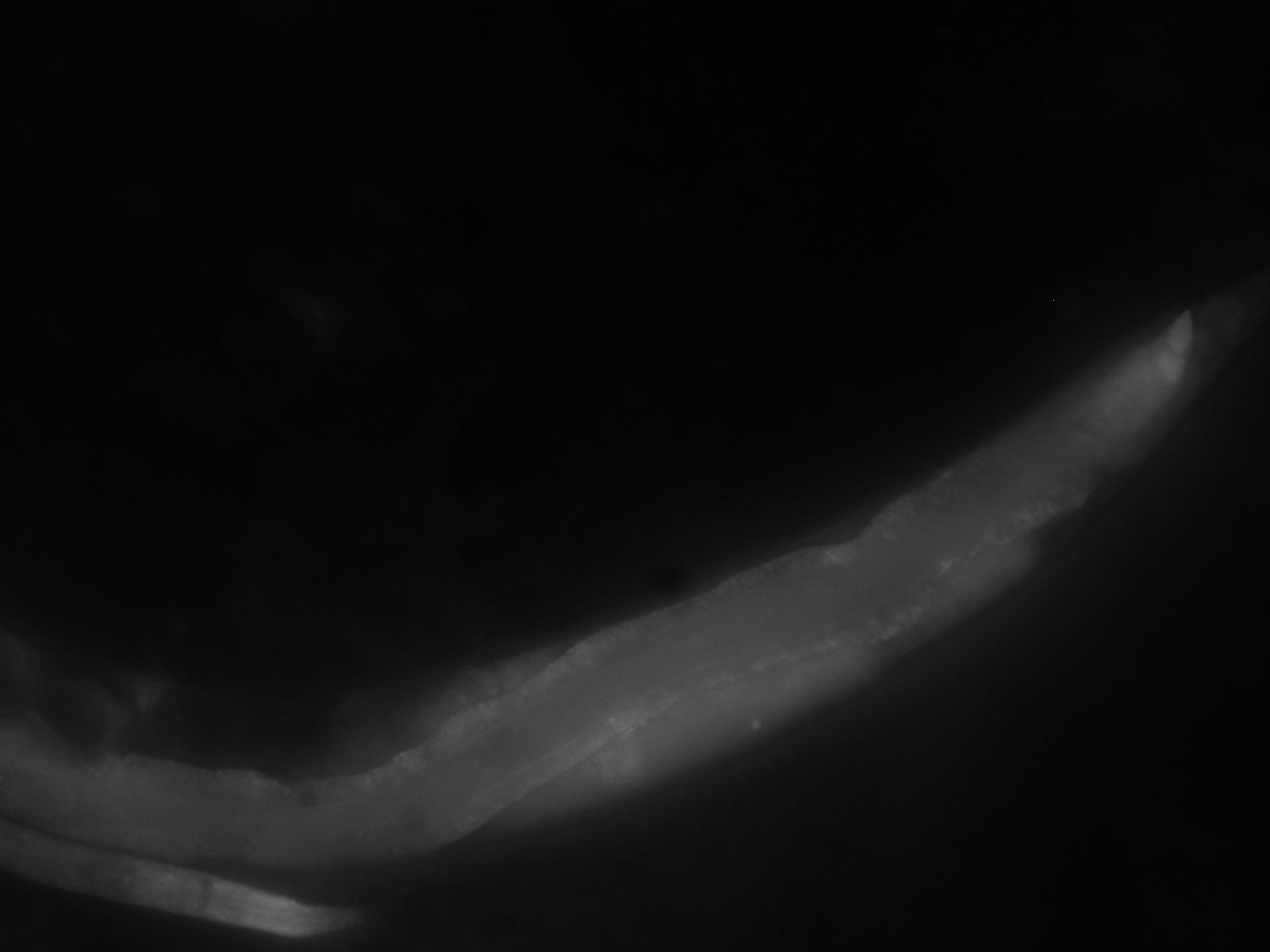

Supplement: S1 File — This file contains all the scoring data of the revised SYTO12 staining experiments. Each of the three biological replicates for Figs 2A, 4D, S2 and S4B–S4C were done in parallel in all strains. Hence, the wild type animals in Fig 2A and in S2 Fig are the same. In most cases animals were scored by live imaging without accompanied image acquisition. Representative images are provided. Consecutive images may image the same gonad. The scoring of apoptotic corpses was performed per gonad, not per image. (ZIP) [file pgen.1011061.s001.zip › SYTO staining experiment united/syto12 staining - 1_rep - 14.5.23 - JPEG/eat-4+pad12255.jpg]

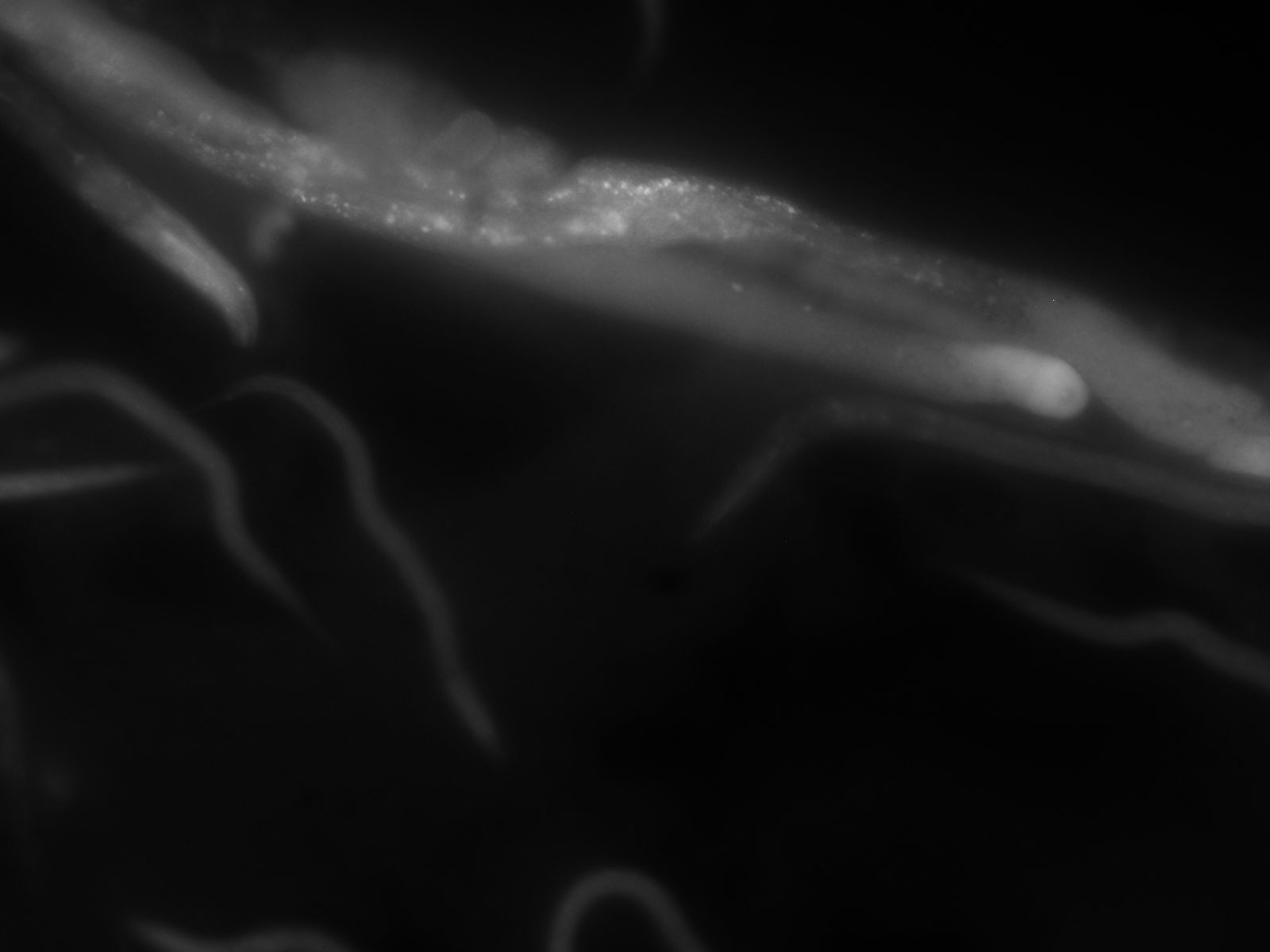

Supplement: S1 File — This file contains all the scoring data of the revised SYTO12 staining experiments. Each of the three biological replicates for Figs 2A, 4D, S2 and S4B–S4C were done in parallel in all strains. Hence, the wild type animals in Fig 2A and in S2 Fig are the same. In most cases animals were scored by live imaging without accompanied image acquisition. Representative images are provided. Consecutive images may image the same gonad. The scoring of apoptotic corpses was performed per gonad, not per image. (ZIP) [file pgen.1011061.s001.zip › SYTO staining experiment united/syto12 staining - 1_rep - 14.5.23 - JPEG/eat-4+pad12256.jpg]

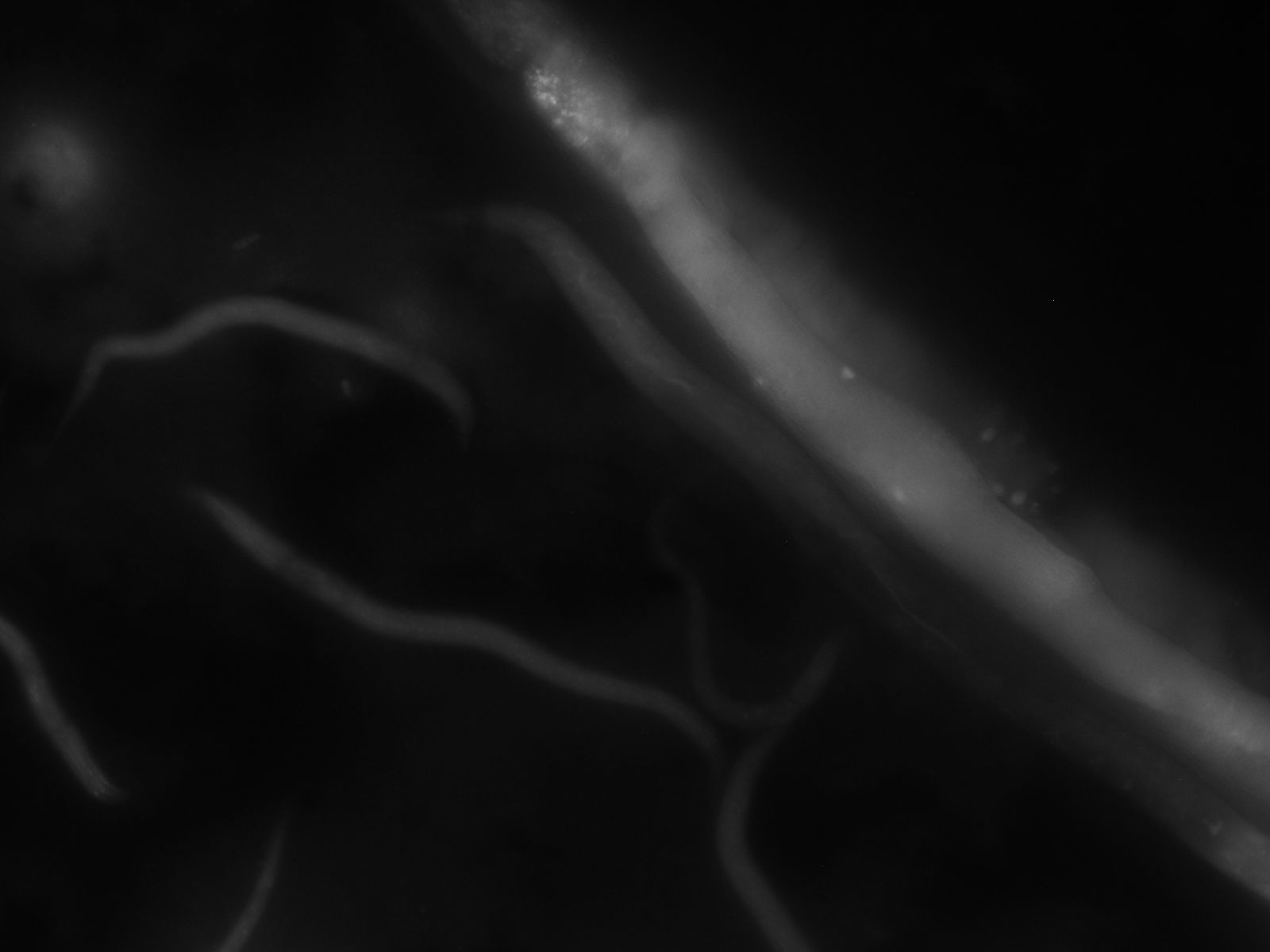

Supplement: S1 File — This file contains all the scoring data of the revised SYTO12 staining experiments. Each of the three biological replicates for Figs 2A, 4D, S2 and S4B–S4C were done in parallel in all strains. Hence, the wild type animals in Fig 2A and in S2 Fig are the same. In most cases animals were scored by live imaging without accompanied image acquisition. Representative images are provided. Consecutive images may image the same gonad. The scoring of apoptotic corpses was performed per gonad, not per image. (ZIP) [file pgen.1011061.s001.zip › SYTO staining experiment united/syto12 staining - 1_rep - 14.5.23 - JPEG/eat-4+pad12257.jpg]

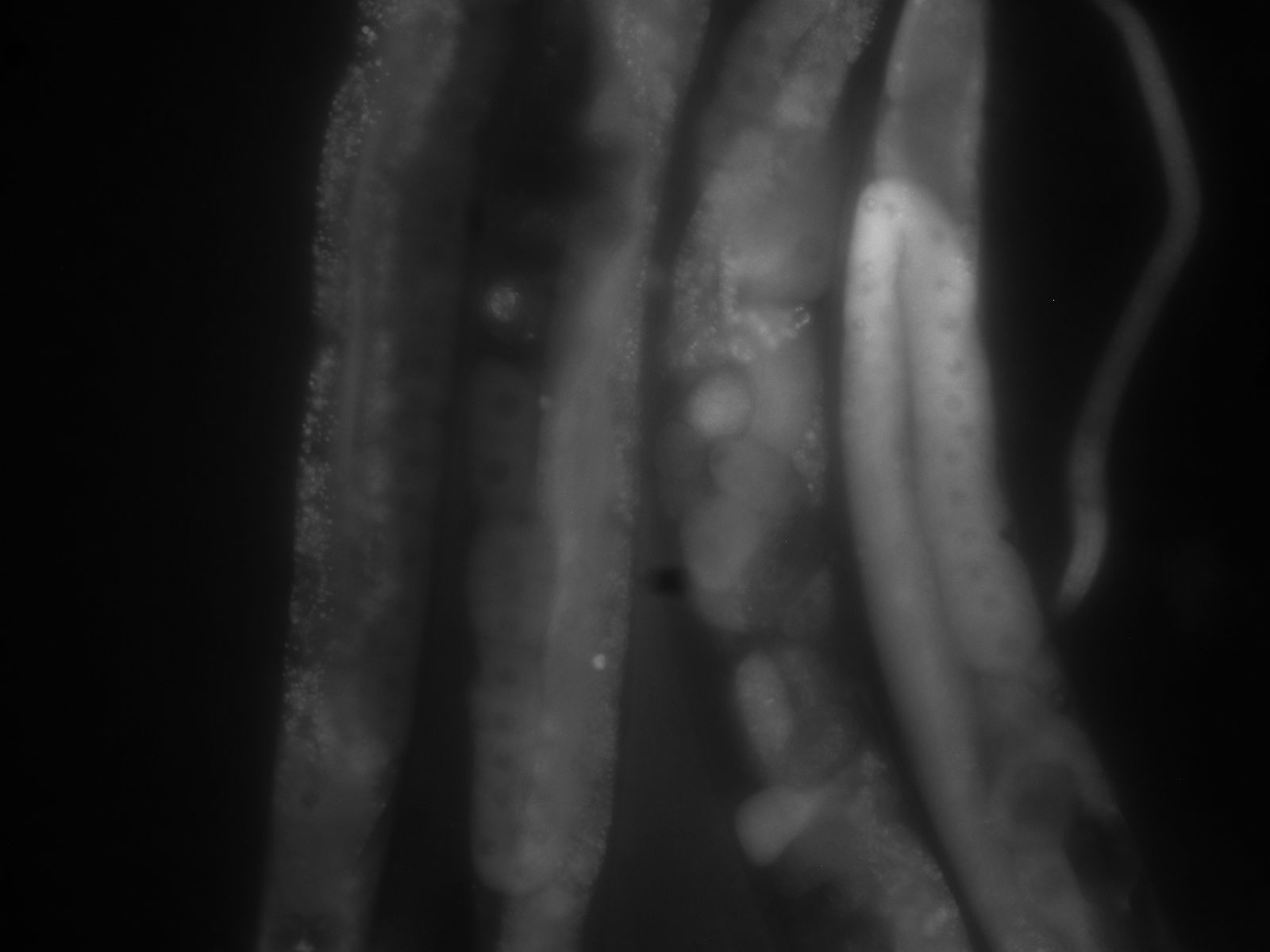

Supplement: S1 File — This file contains all the scoring data of the revised SYTO12 staining experiments. Each of the three biological replicates for Figs 2A, 4D, S2 and S4B–S4C were done in parallel in all strains. Hence, the wild type animals in Fig 2A and in S2 Fig are the same. In most cases animals were scored by live imaging without accompanied image acquisition. Representative images are provided. Consecutive images may image the same gonad. The scoring of apoptotic corpses was performed per gonad, not per image. (ZIP) [file pgen.1011061.s001.zip › SYTO staining experiment united/syto12 staining - 1_rep - 14.5.23 - JPEG/eat-4+pad12258.jpg]

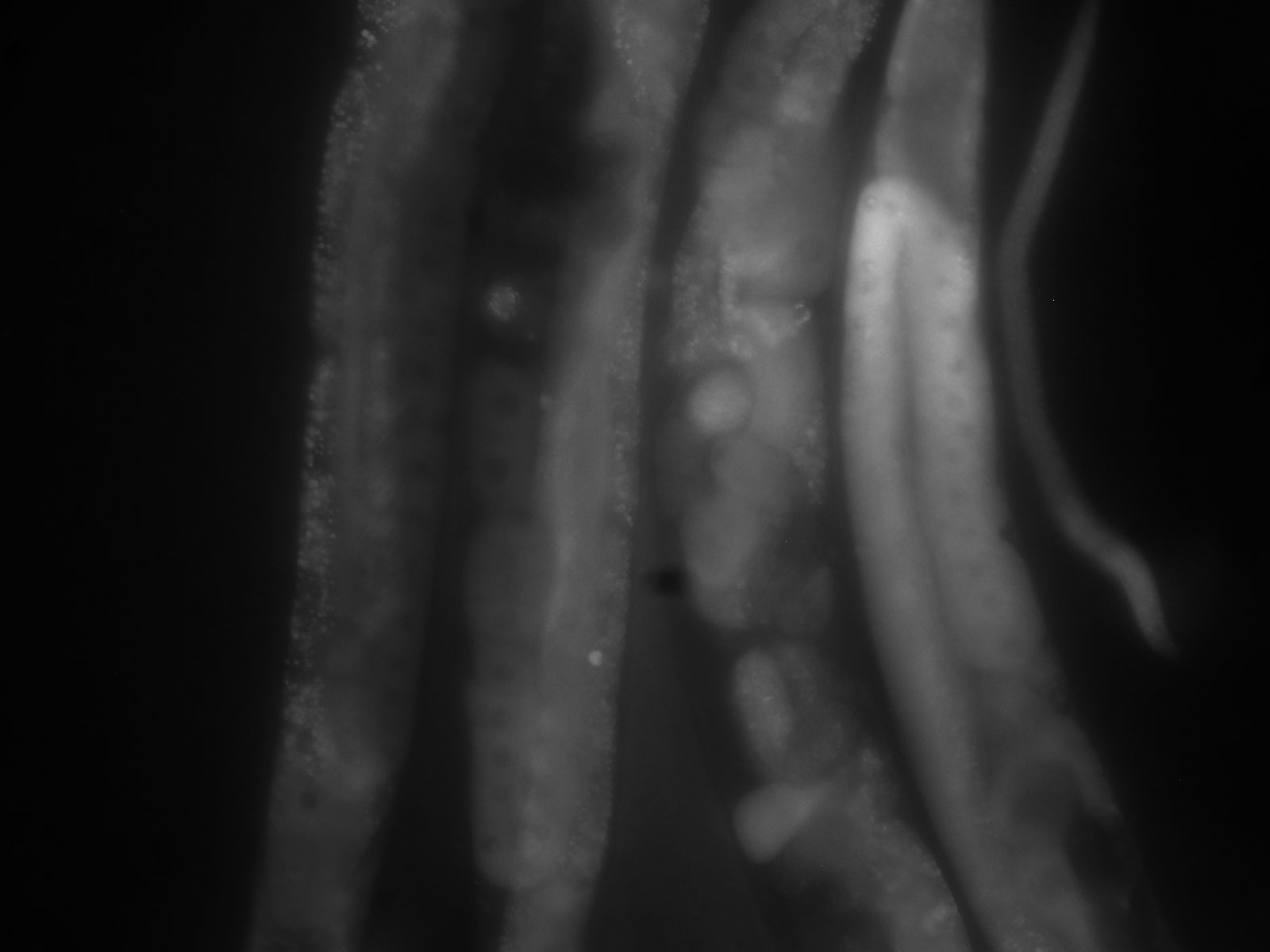

Supplement: S1 File — This file contains all the scoring data of the revised SYTO12 staining experiments. Each of the three biological replicates for Figs 2A, 4D, S2 and S4B–S4C were done in parallel in all strains. Hence, the wild type animals in Fig 2A and in S2 Fig are the same. In most cases animals were scored by live imaging without accompanied image acquisition. Representative images are provided. Consecutive images may image the same gonad. The scoring of apoptotic corpses was performed per gonad, not per image. (ZIP) [file pgen.1011061.s001.zip › SYTO staining experiment united/syto12 staining - 1_rep - 14.5.23 - JPEG/eat-4+pad12259.jpg]

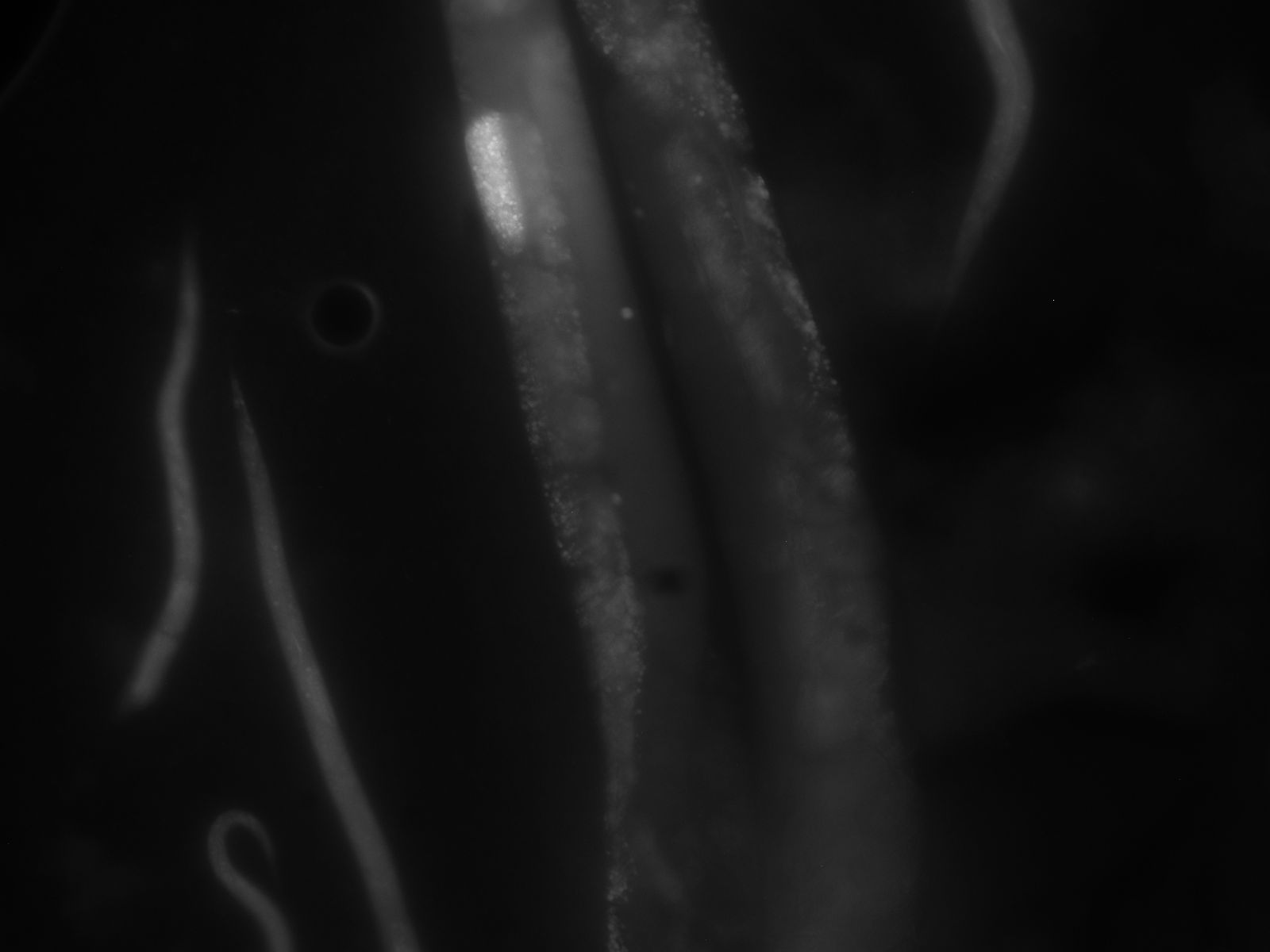

Supplement: S1 File — This file contains all the scoring data of the revised SYTO12 staining experiments. Each of the three biological replicates for Figs 2A, 4D, S2 and S4B–S4C were done in parallel in all strains. Hence, the wild type animals in Fig 2A and in S2 Fig are the same. In most cases animals were scored by live imaging without accompanied image acquisition. Representative images are provided. Consecutive images may image the same gonad. The scoring of apoptotic corpses was performed per gonad, not per image. (ZIP) [file pgen.1011061.s001.zip › SYTO staining experiment united/syto12 staining - 1_rep - 14.5.23 - JPEG/eat-4+pad12260.jpg]

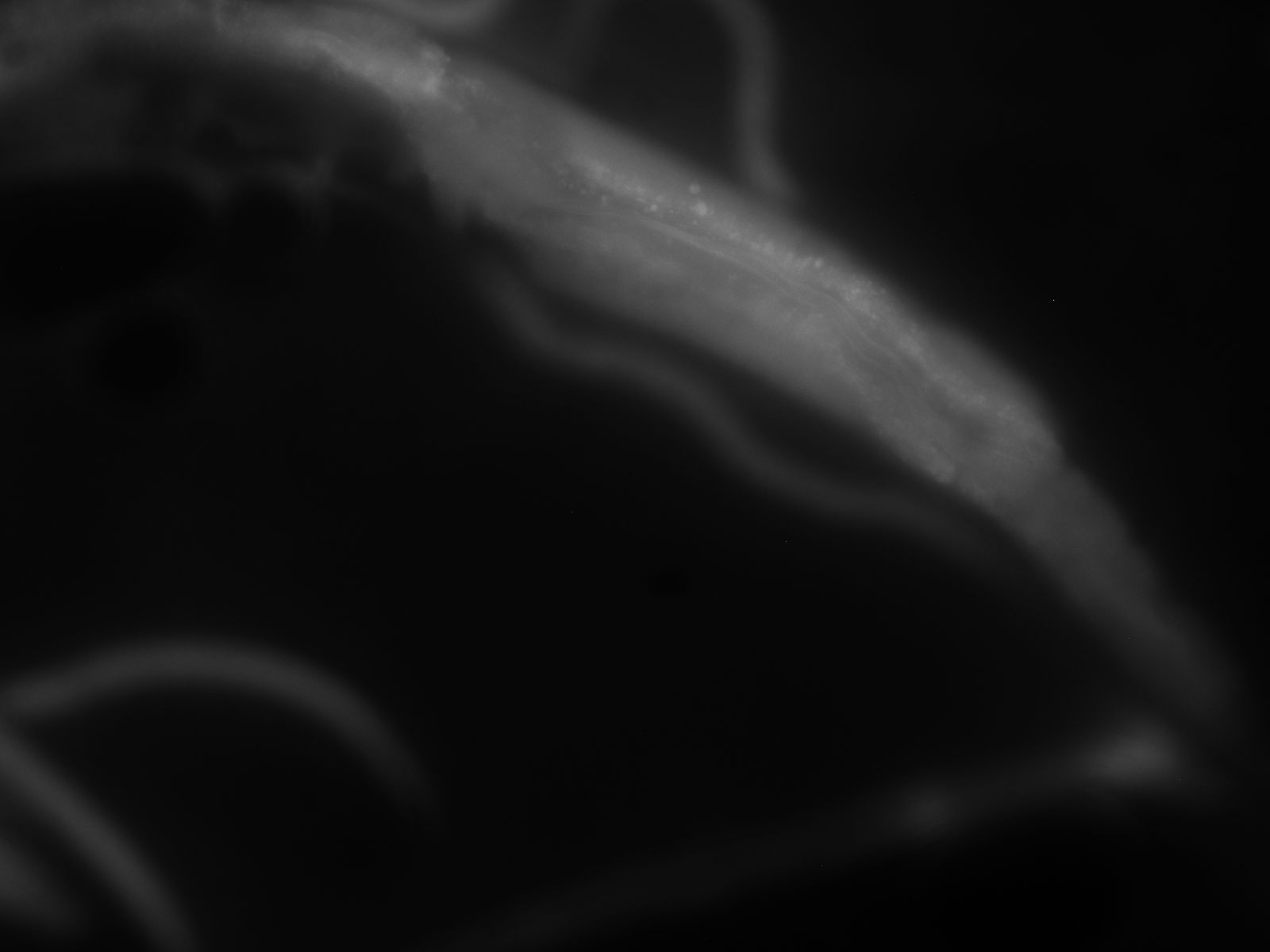

Supplement: S1 File — This file contains all the scoring data of the revised SYTO12 staining experiments. Each of the three biological replicates for Figs 2A, 4D, S2 and S4B–S4C were done in parallel in all strains. Hence, the wild type animals in Fig 2A and in S2 Fig are the same. In most cases animals were scored by live imaging without accompanied image acquisition. Representative images are provided. Consecutive images may image the same gonad. The scoring of apoptotic corpses was performed per gonad, not per image. (ZIP) [file pgen.1011061.s001.zip › SYTO staining experiment united/syto12 staining - 1_rep - 14.5.23 - JPEG/eat-4+pad12261.jpg]

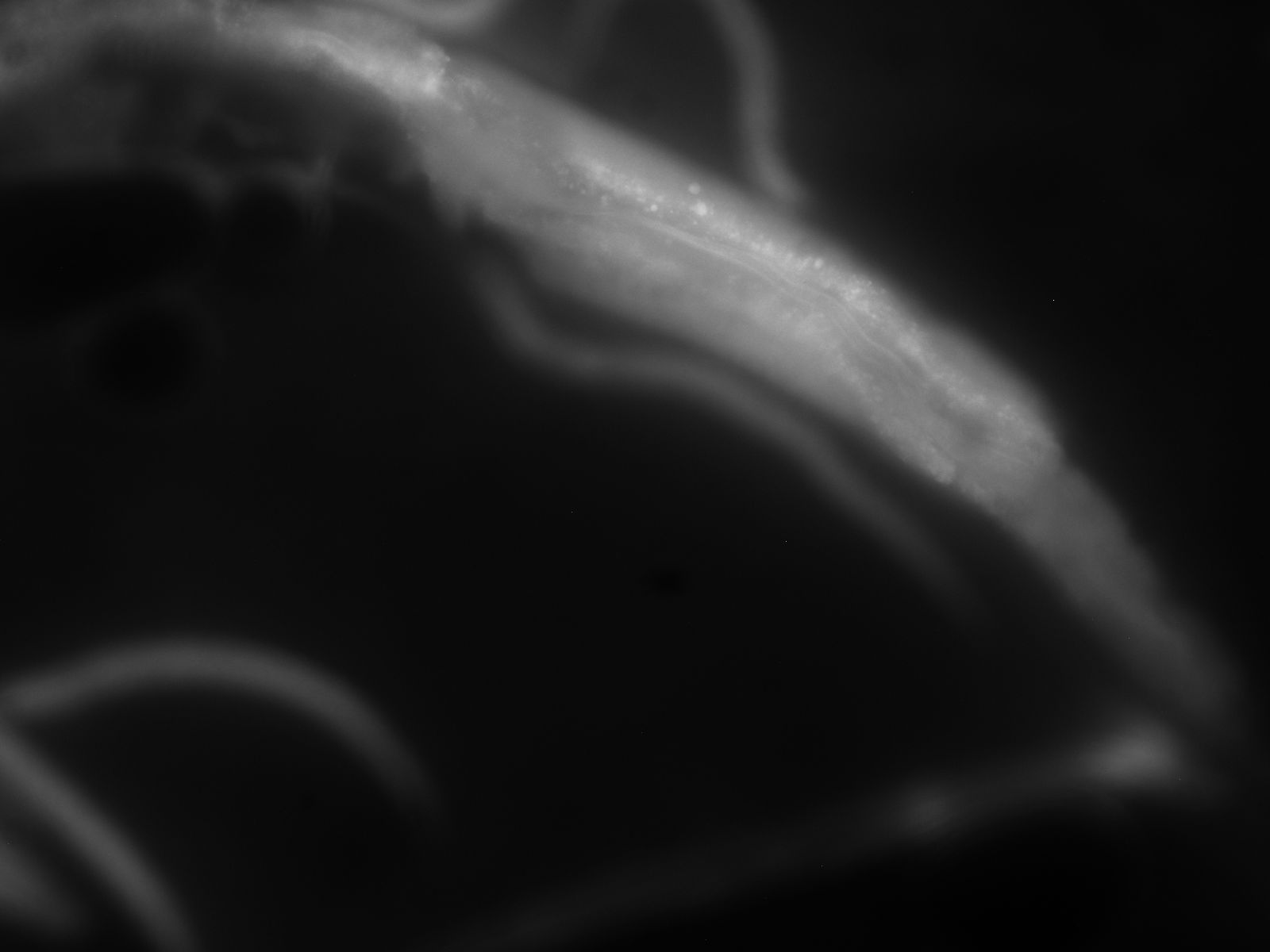

Supplement: S1 File — This file contains all the scoring data of the revised SYTO12 staining experiments. Each of the three biological replicates for Figs 2A, 4D, S2 and S4B–S4C were done in parallel in all strains. Hence, the wild type animals in Fig 2A and in S2 Fig are the same. In most cases animals were scored by live imaging without accompanied image acquisition. Representative images are provided. Consecutive images may image the same gonad. The scoring of apoptotic corpses was performed per gonad, not per image. (ZIP) [file pgen.1011061.s001.zip › SYTO staining experiment united/syto12 staining - 1_rep - 14.5.23 - JPEG/eat-4+pad12262.jpg]

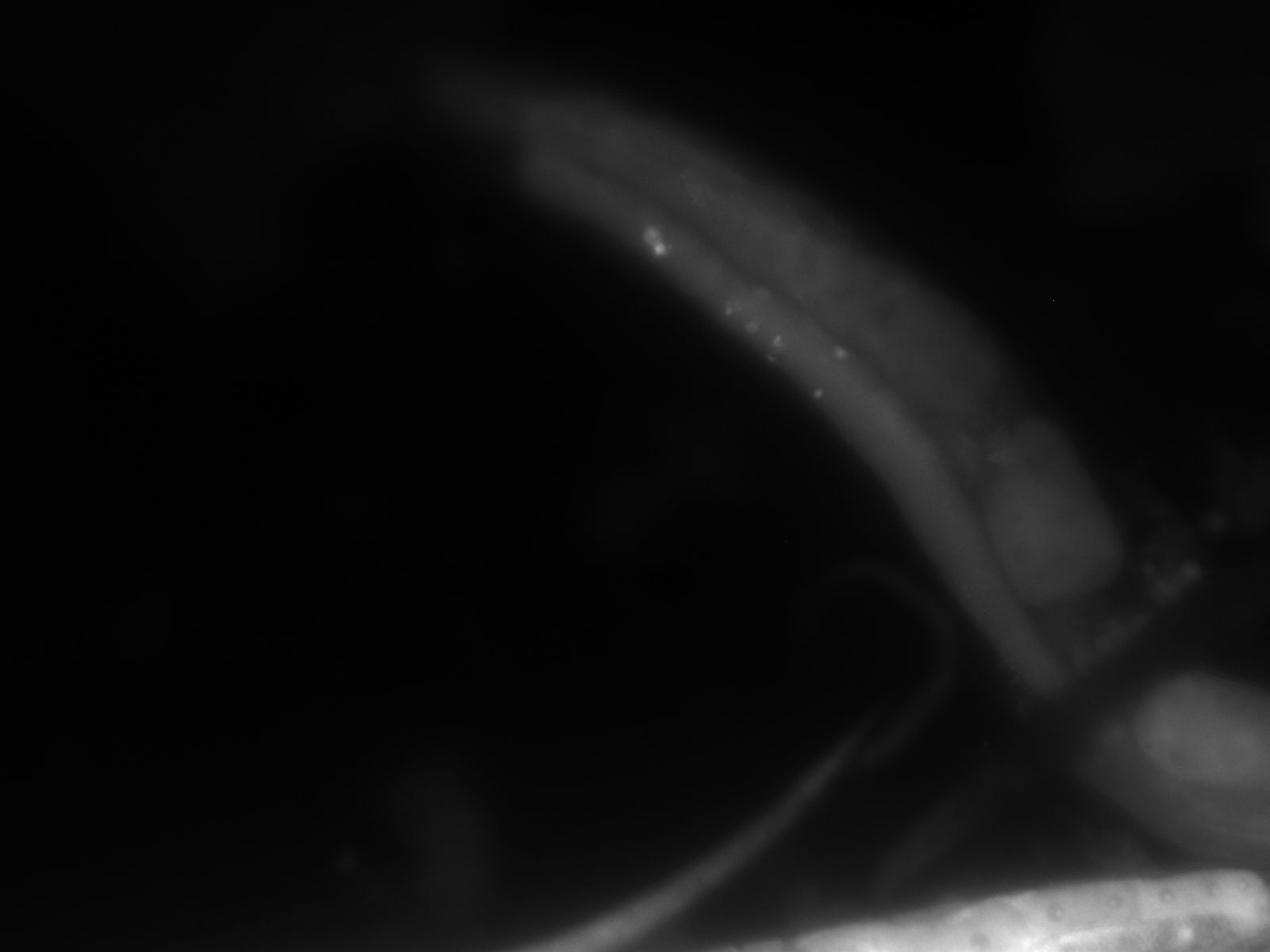

Supplement: S1 File — This file contains all the scoring data of the revised SYTO12 staining experiments. Each of the three biological replicates for Figs 2A, 4D, S2 and S4B–S4C were done in parallel in all strains. Hence, the wild type animals in Fig 2A and in S2 Fig are the same. In most cases animals were scored by live imaging without accompanied image acquisition. Representative images are provided. Consecutive images may image the same gonad. The scoring of apoptotic corpses was performed per gonad, not per image. (ZIP) [file pgen.1011061.s001.zip › SYTO staining experiment united/syto12 staining - 1_rep - 14.5.23 - JPEG/eat-4+tfg-1263.jpg]

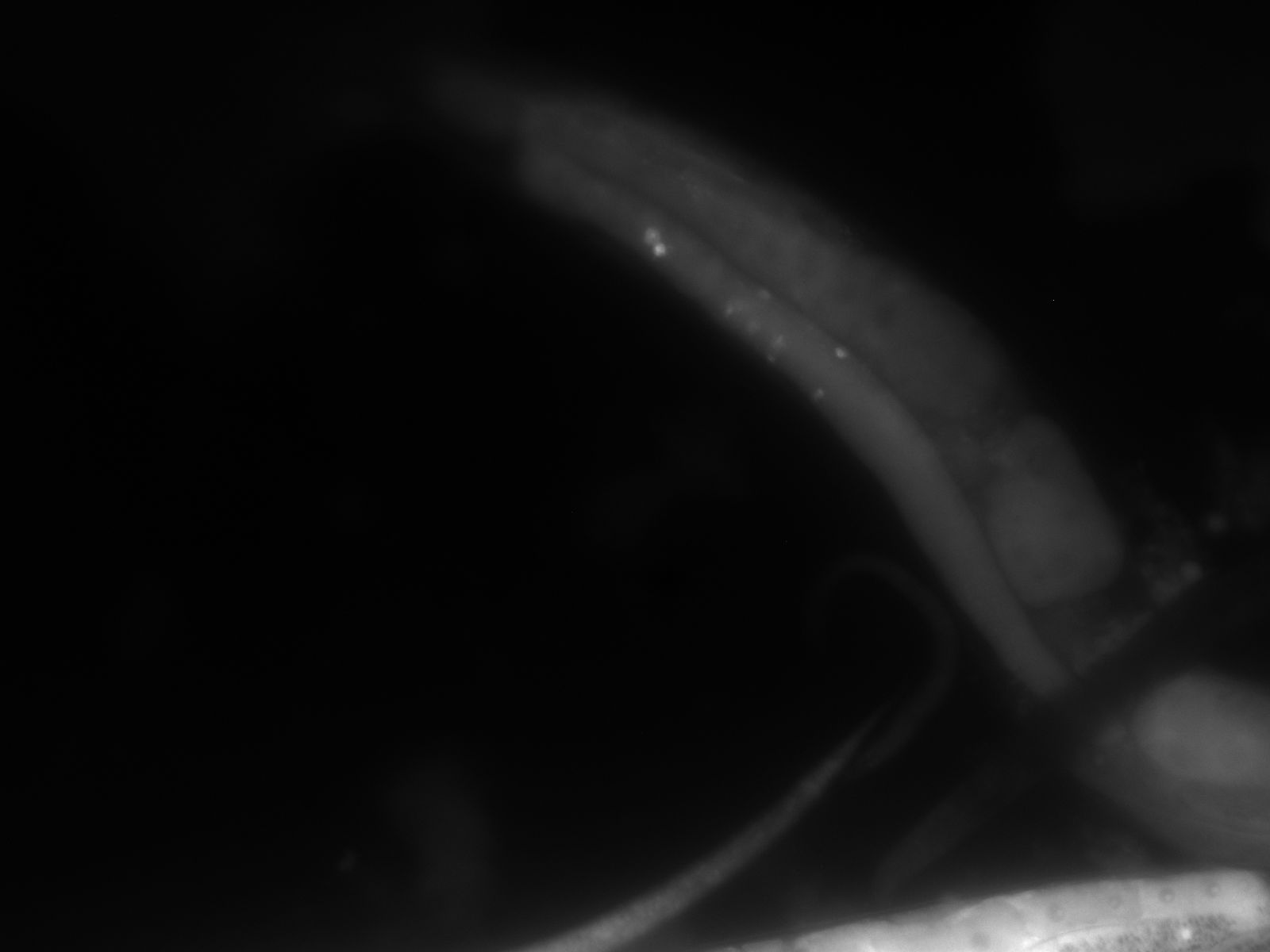

Supplement: S1 File — This file contains all the scoring data of the revised SYTO12 staining experiments. Each of the three biological replicates for Figs 2A, 4D, S2 and S4B–S4C were done in parallel in all strains. Hence, the wild type animals in Fig 2A and in S2 Fig are the same. In most cases animals were scored by live imaging without accompanied image acquisition. Representative images are provided. Consecutive images may image the same gonad. The scoring of apoptotic corpses was performed per gonad, not per image. (ZIP) [file pgen.1011061.s001.zip › SYTO staining experiment united/syto12 staining - 1_rep - 14.5.23 - JPEG/eat-4+tfg-1264.jpg]

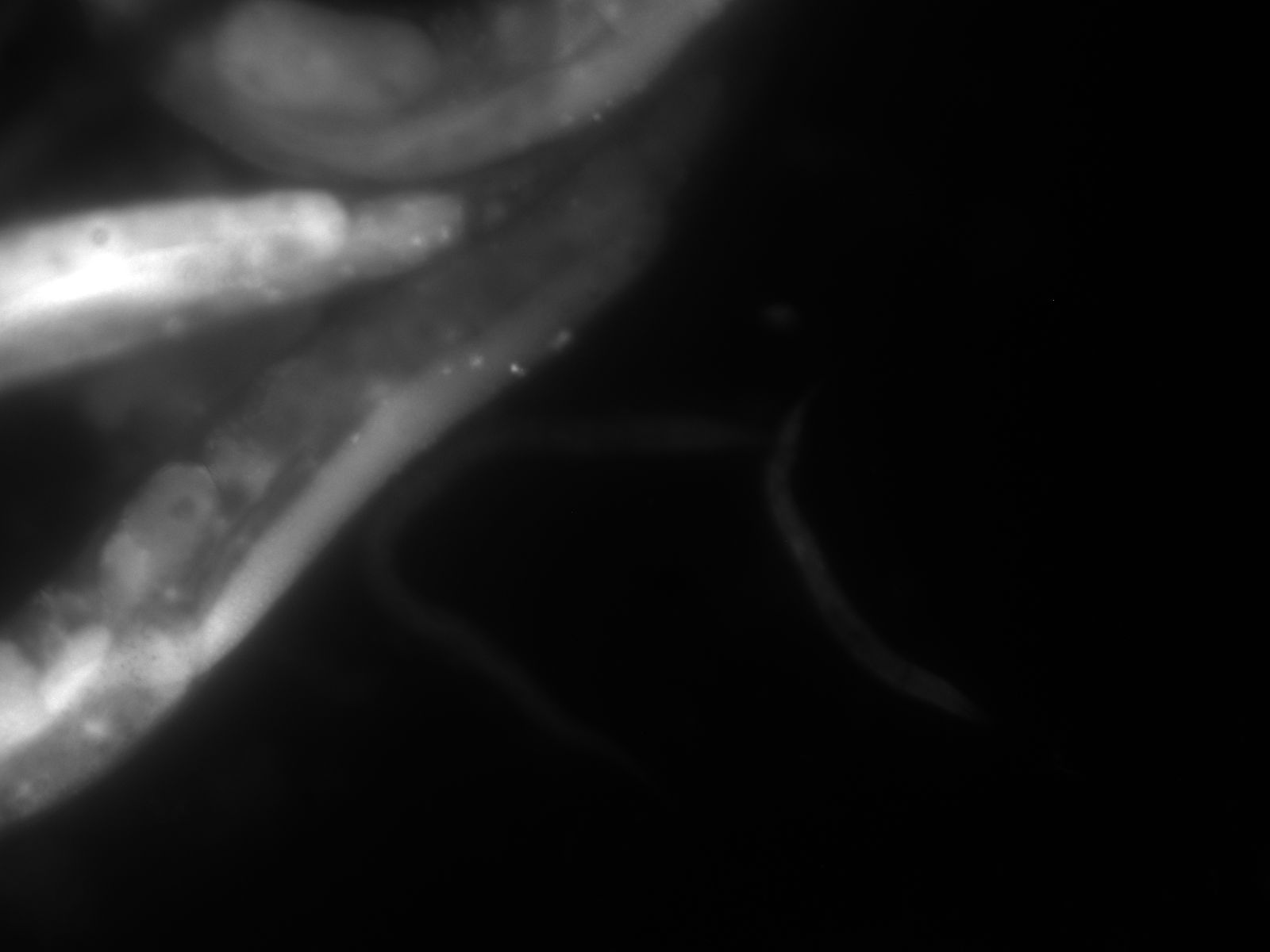

Supplement: S1 File — This file contains all the scoring data of the revised SYTO12 staining experiments. Each of the three biological replicates for Figs 2A, 4D, S2 and S4B–S4C were done in parallel in all strains. Hence, the wild type animals in Fig 2A and in S2 Fig are the same. In most cases animals were scored by live imaging without accompanied image acquisition. Representative images are provided. Consecutive images may image the same gonad. The scoring of apoptotic corpses was performed per gonad, not per image. (ZIP) [file pgen.1011061.s001.zip › SYTO staining experiment united/syto12 staining - 1_rep - 14.5.23 - JPEG/eat-4+tfg-1265.jpg]

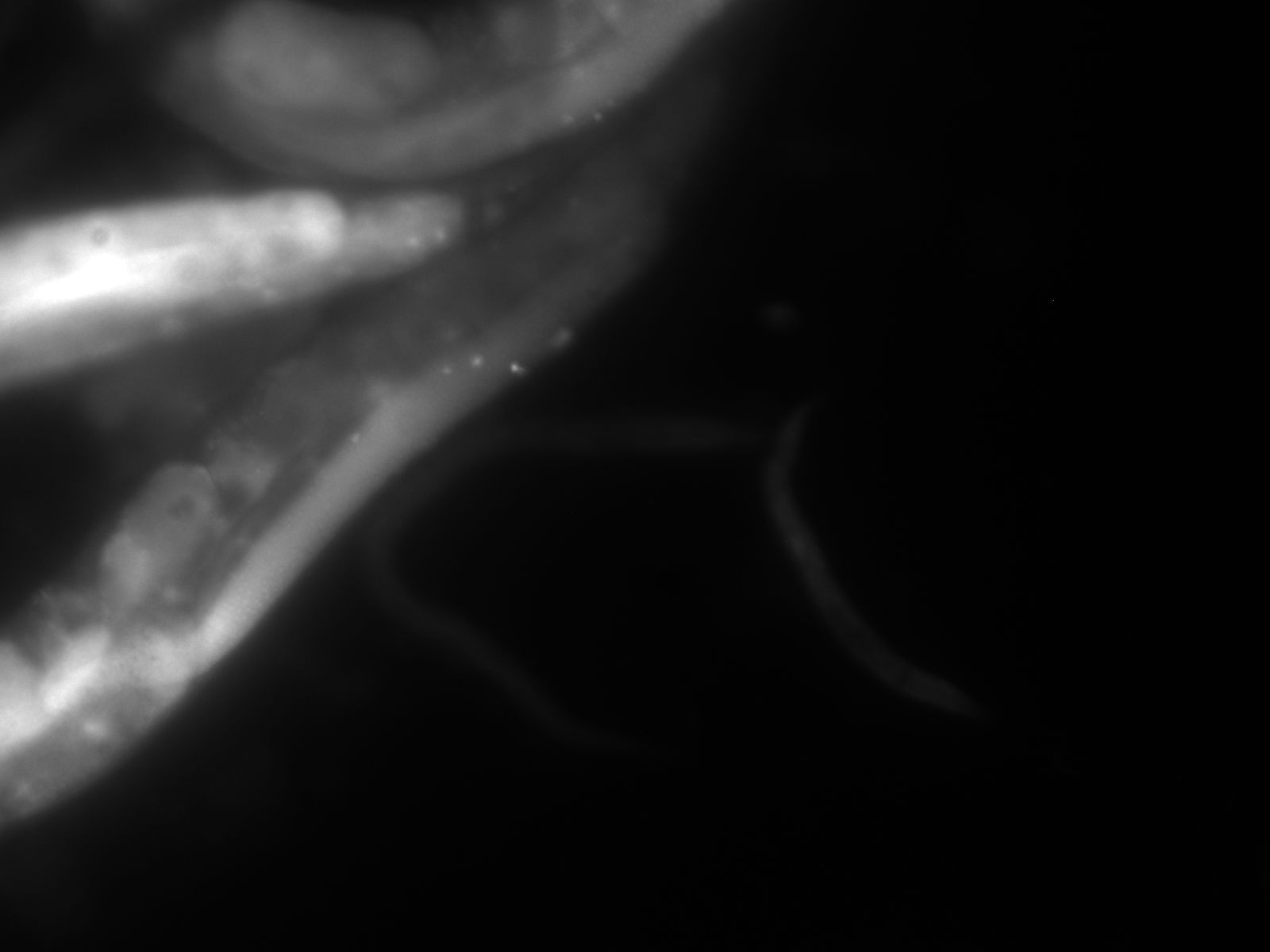

Supplement: S1 File — This file contains all the scoring data of the revised SYTO12 staining experiments. Each of the three biological replicates for Figs 2A, 4D, S2 and S4B–S4C were done in parallel in all strains. Hence, the wild type animals in Fig 2A and in S2 Fig are the same. In most cases animals were scored by live imaging without accompanied image acquisition. Representative images are provided. Consecutive images may image the same gonad. The scoring of apoptotic corpses was performed per gonad, not per image. (ZIP) [file pgen.1011061.s001.zip › SYTO staining experiment united/syto12 staining - 1_rep - 14.5.23 - JPEG/eat-4+tfg-1266.jpg]

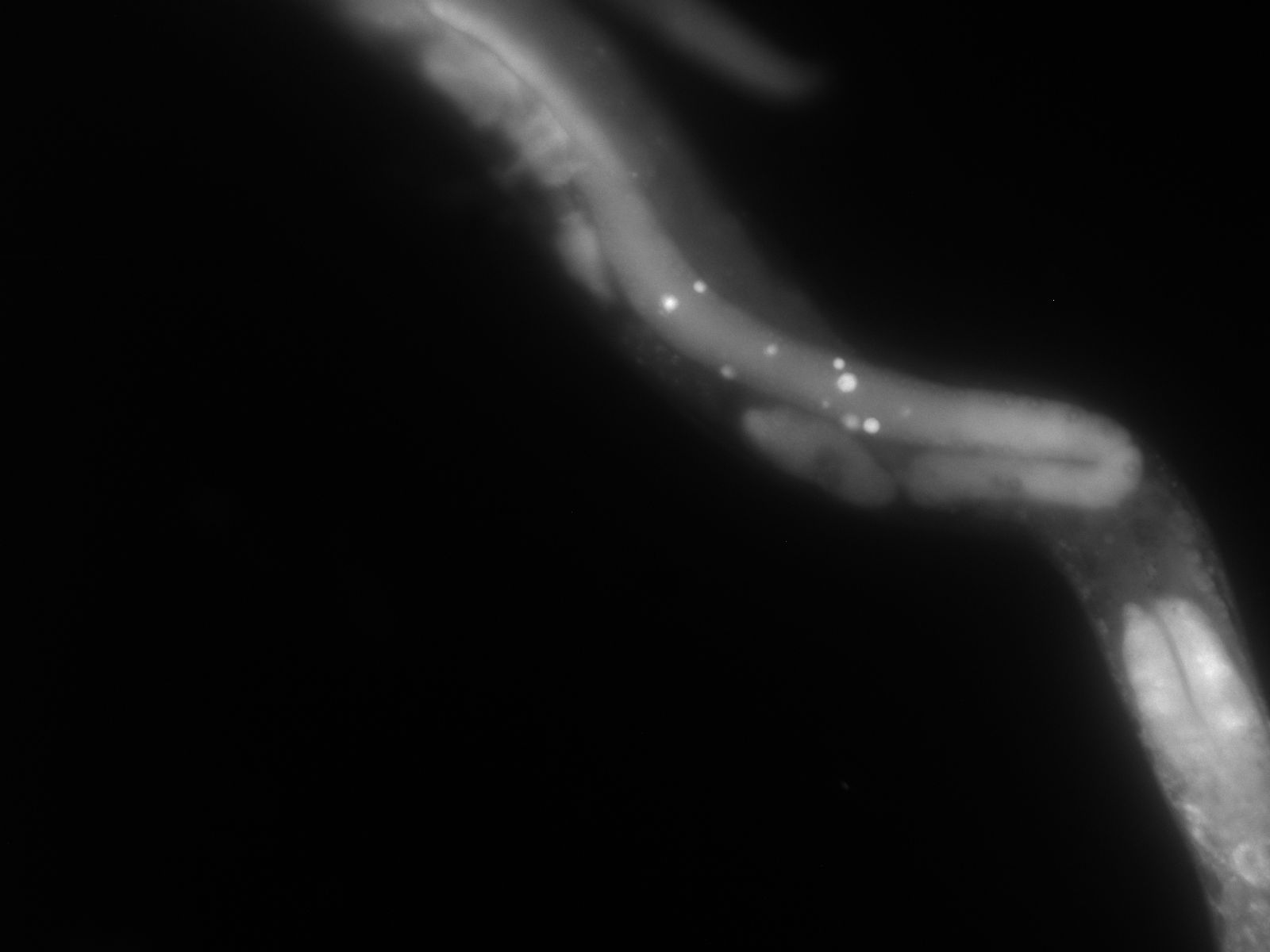

Supplement: S1 File — This file contains all the scoring data of the revised SYTO12 staining experiments. Each of the three biological replicates for Figs 2A, 4D, S2 and S4B–S4C were done in parallel in all strains. Hence, the wild type animals in Fig 2A and in S2 Fig are the same. In most cases animals were scored by live imaging without accompanied image acquisition. Representative images are provided. Consecutive images may image the same gonad. The scoring of apoptotic corpses was performed per gonad, not per image. (ZIP) [file pgen.1011061.s001.zip › SYTO staining experiment united/syto12 staining - 1_rep - 14.5.23 - JPEG/eat-4+tfg-1267.jpg]

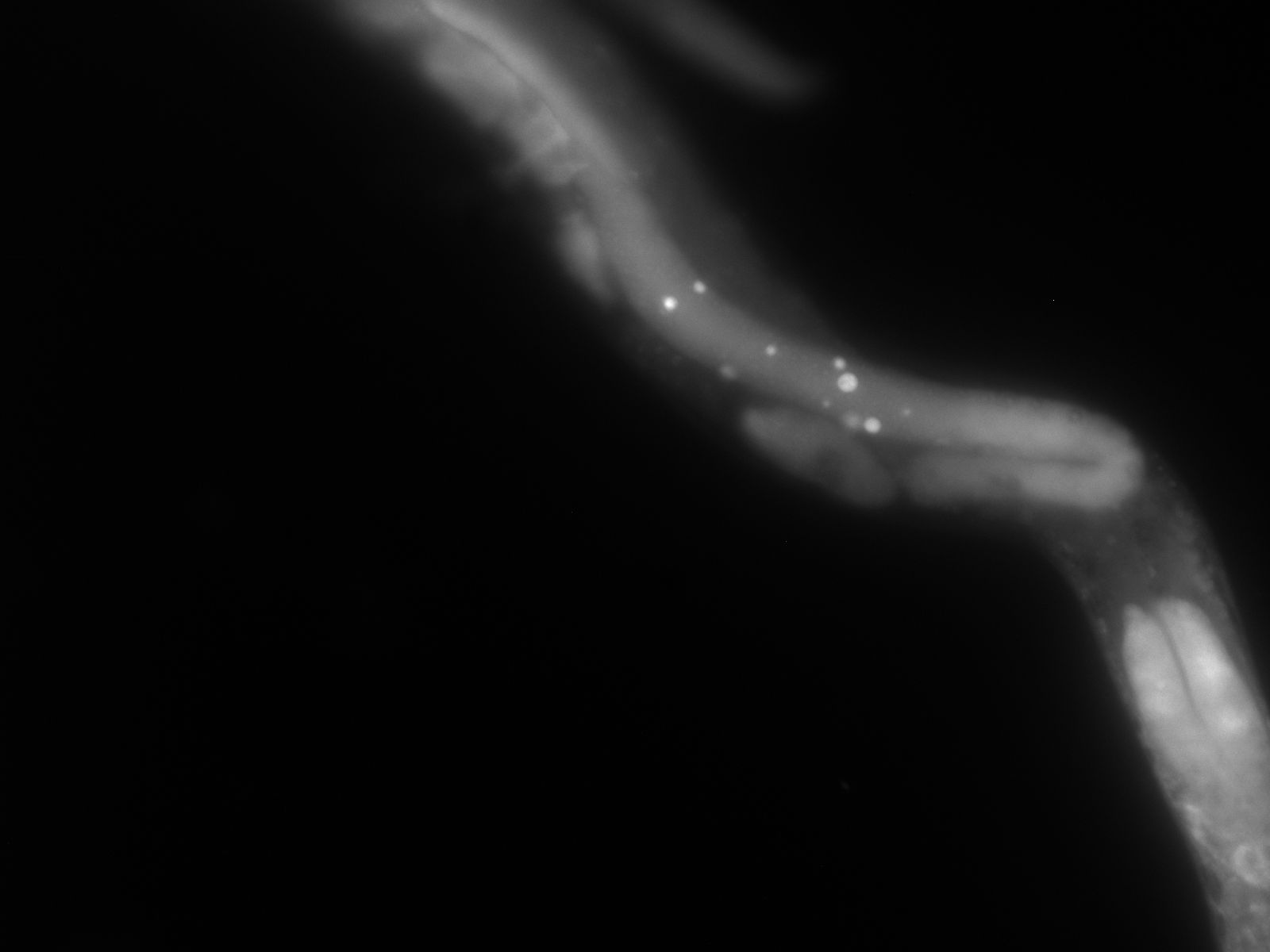

Supplement: S1 File — This file contains all the scoring data of the revised SYTO12 staining experiments. Each of the three biological replicates for Figs 2A, 4D, S2 and S4B–S4C were done in parallel in all strains. Hence, the wild type animals in Fig 2A and in S2 Fig are the same. In most cases animals were scored by live imaging without accompanied image acquisition. Representative images are provided. Consecutive images may image the same gonad. The scoring of apoptotic corpses was performed per gonad, not per image. (ZIP) [file pgen.1011061.s001.zip › SYTO staining experiment united/syto12 staining - 1_rep - 14.5.23 - JPEG/eat-4+tfg-1268.jpg]

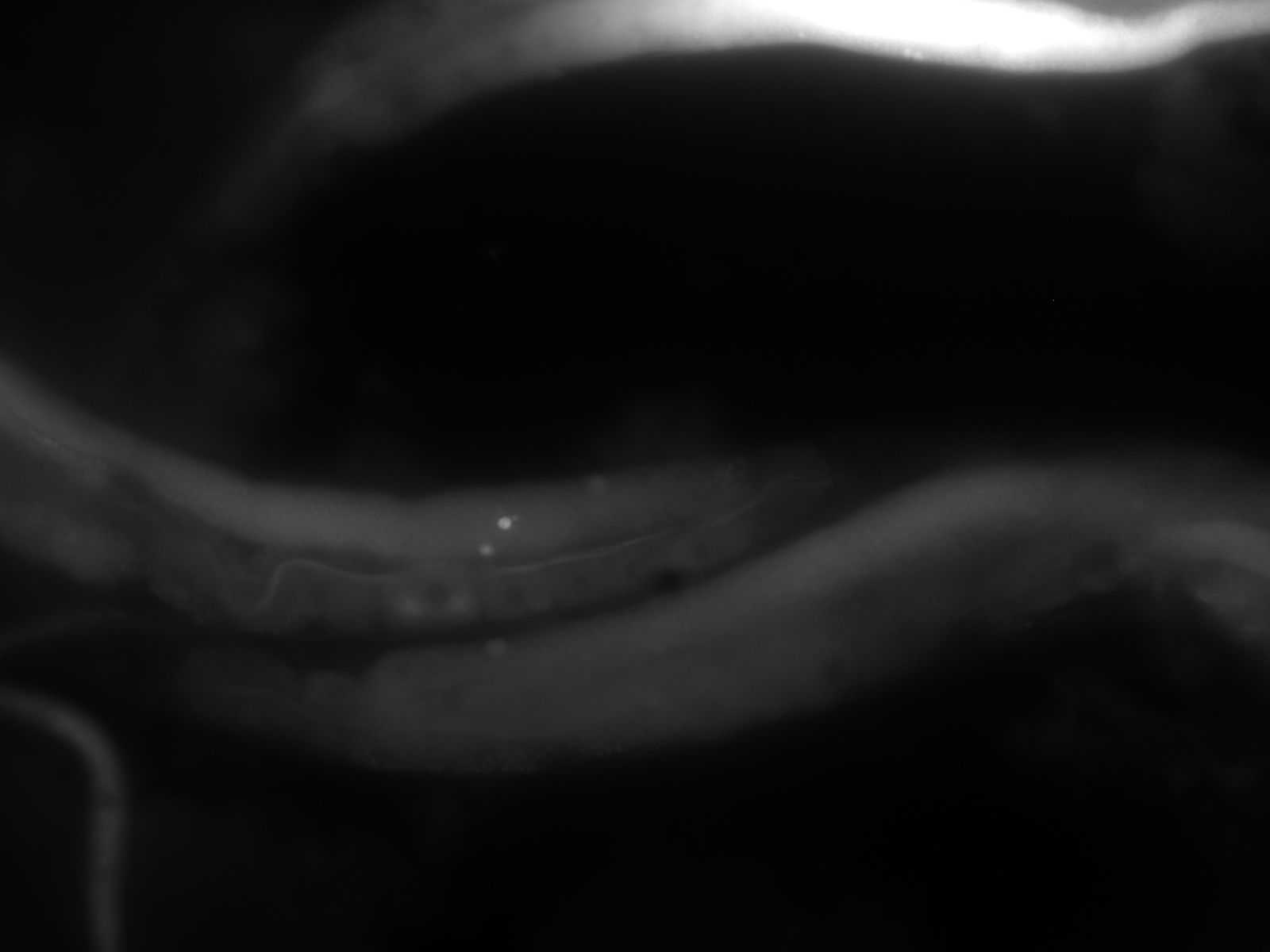

Supplement: S1 File — This file contains all the scoring data of the revised SYTO12 staining experiments. Each of the three biological replicates for Figs 2A, 4D, S2 and S4B–S4C were done in parallel in all strains. Hence, the wild type animals in Fig 2A and in S2 Fig are the same. In most cases animals were scored by live imaging without accompanied image acquisition. Representative images are provided. Consecutive images may image the same gonad. The scoring of apoptotic corpses was performed per gonad, not per image. (ZIP) [file pgen.1011061.s001.zip › SYTO staining experiment united/syto12 staining - 1_rep - 14.5.23 - JPEG/eat-4+tfg-1269.jpg]

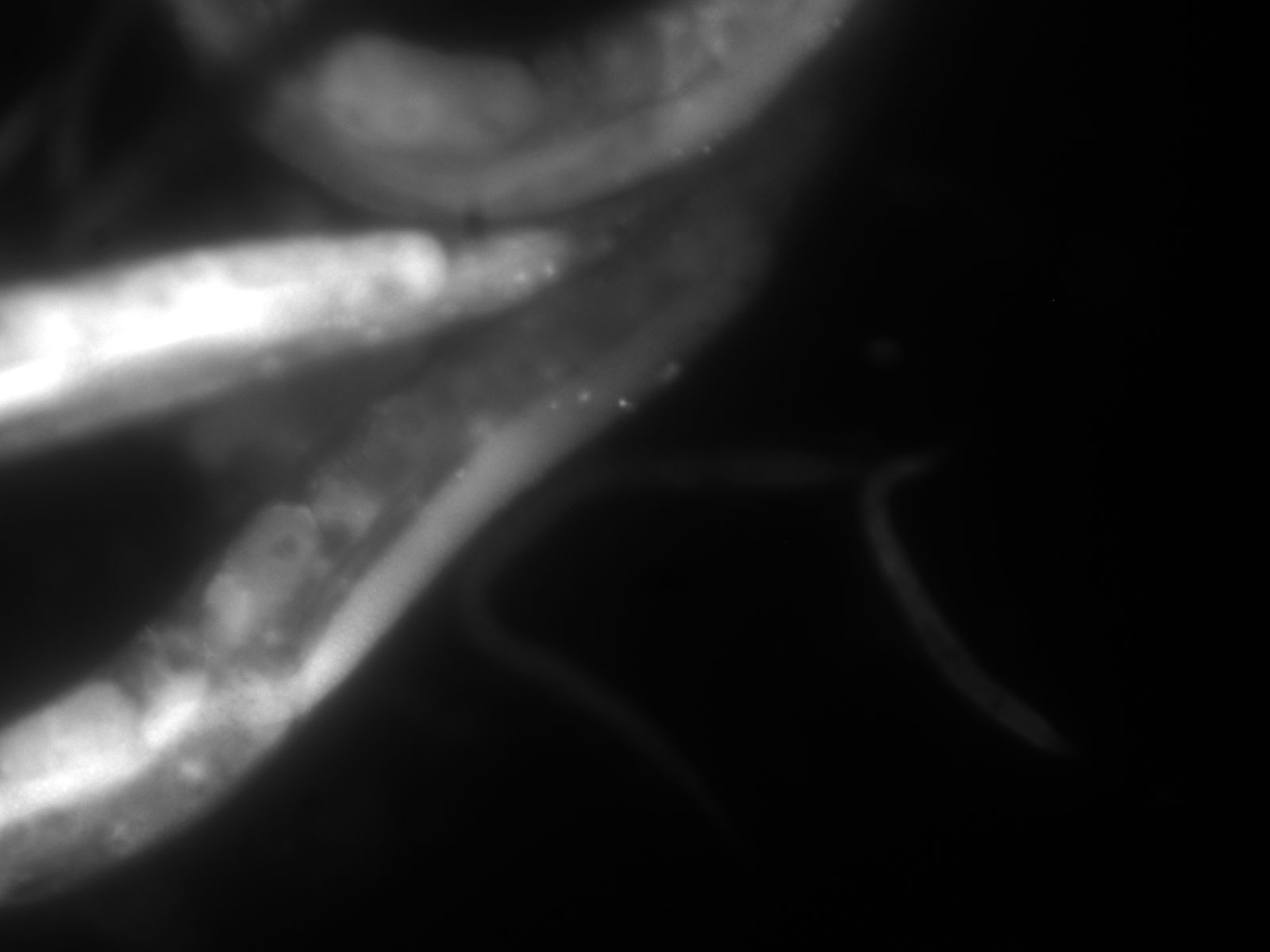

Supplement: S1 File — This file contains all the scoring data of the revised SYTO12 staining experiments. Each of the three biological replicates for Figs 2A, 4D, S2 and S4B–S4C were done in parallel in all strains. Hence, the wild type animals in Fig 2A and in S2 Fig are the same. In most cases animals were scored by live imaging without accompanied image acquisition. Representative images are provided. Consecutive images may image the same gonad. The scoring of apoptotic corpses was performed per gonad, not per image. (ZIP) [file pgen.1011061.s001.zip › SYTO staining experiment united/syto12 staining - 1_rep - 14.5.23 - JPEG/eat-4+tfg-1270.jpg]

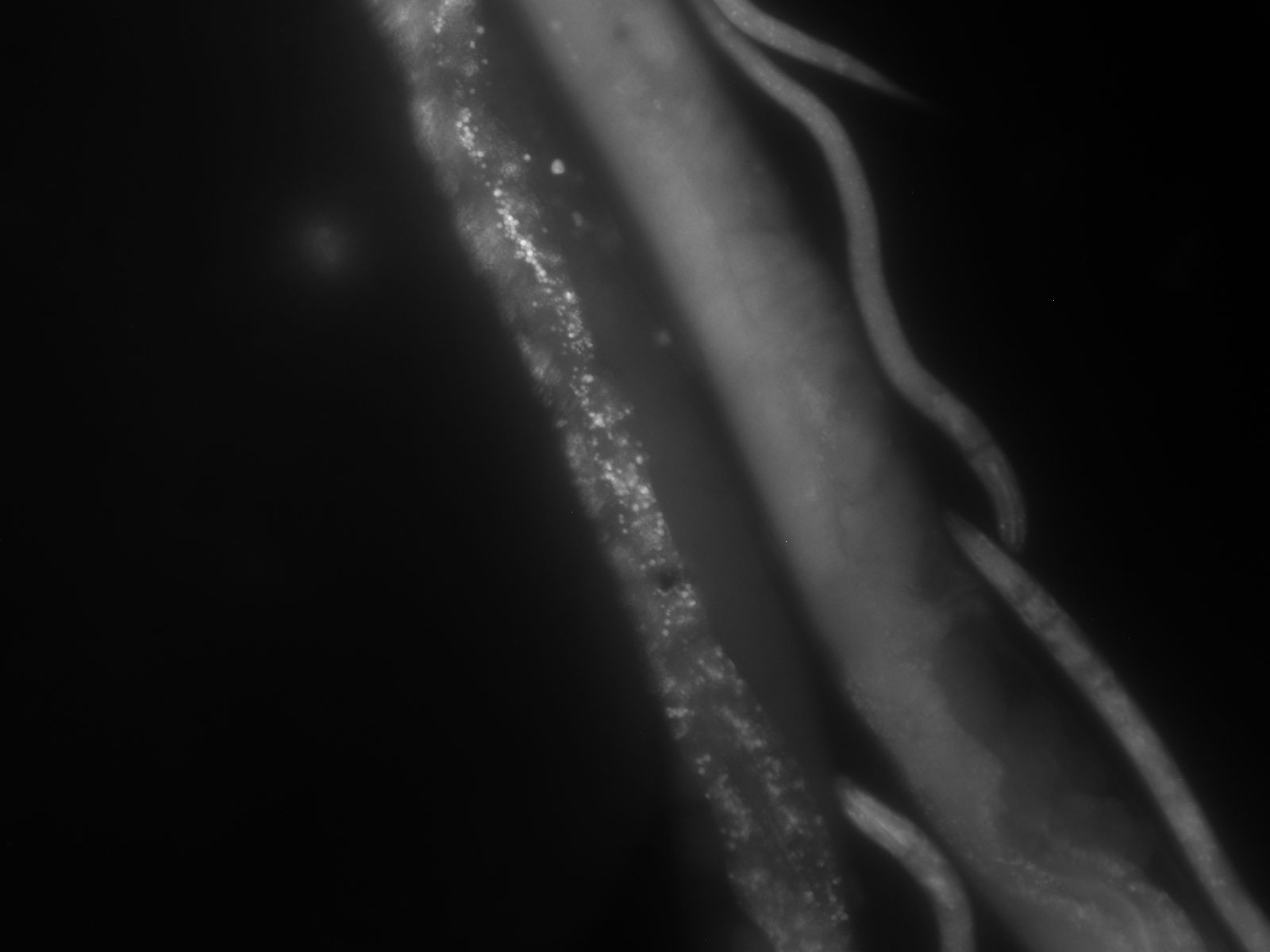

Supplement: S1 File — This file contains all the scoring data of the revised SYTO12 staining experiments. Each of the three biological replicates for Figs 2A, 4D, S2 and S4B–S4C were done in parallel in all strains. Hence, the wild type animals in Fig 2A and in S2 Fig are the same. In most cases animals were scored by live imaging without accompanied image acquisition. Representative images are provided. Consecutive images may image the same gonad. The scoring of apoptotic corpses was performed per gonad, not per image. (ZIP) [file pgen.1011061.s001.zip › SYTO staining experiment united/syto12 staining - 1_rep - 14.5.23 - JPEG/eat-4+tfg-1271.jpg]

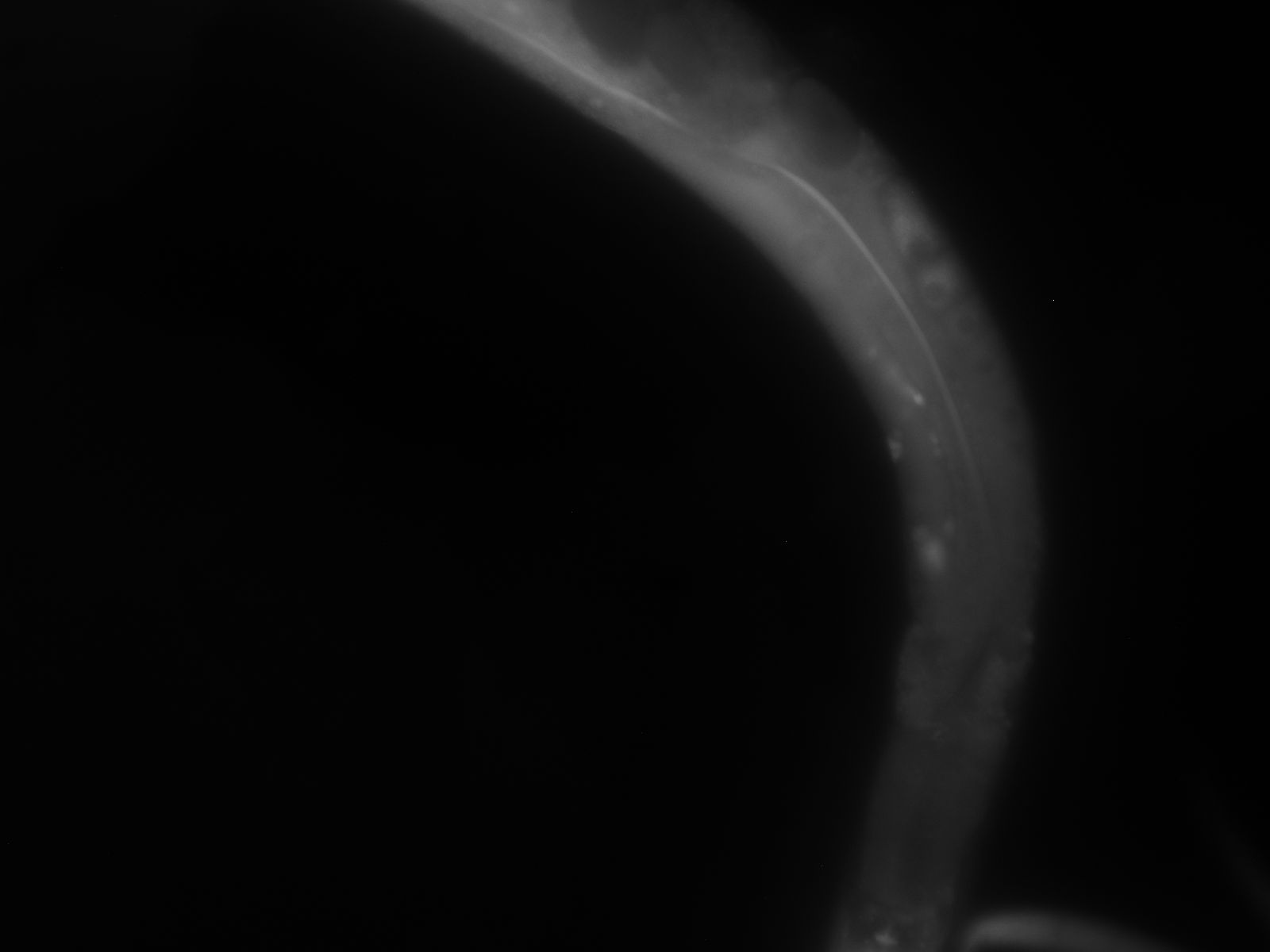

Supplement: S1 File — This file contains all the scoring data of the revised SYTO12 staining experiments. Each of the three biological replicates for Figs 2A, 4D, S2 and S4B–S4C were done in parallel in all strains. Hence, the wild type animals in Fig 2A and in S2 Fig are the same. In most cases animals were scored by live imaging without accompanied image acquisition. Representative images are provided. Consecutive images may image the same gonad. The scoring of apoptotic corpses was performed per gonad, not per image. (ZIP) [file pgen.1011061.s001.zip › SYTO staining experiment united/syto12 staining - 1_rep - 14.5.23 - JPEG/eat-4+tfg-1272.jpg]

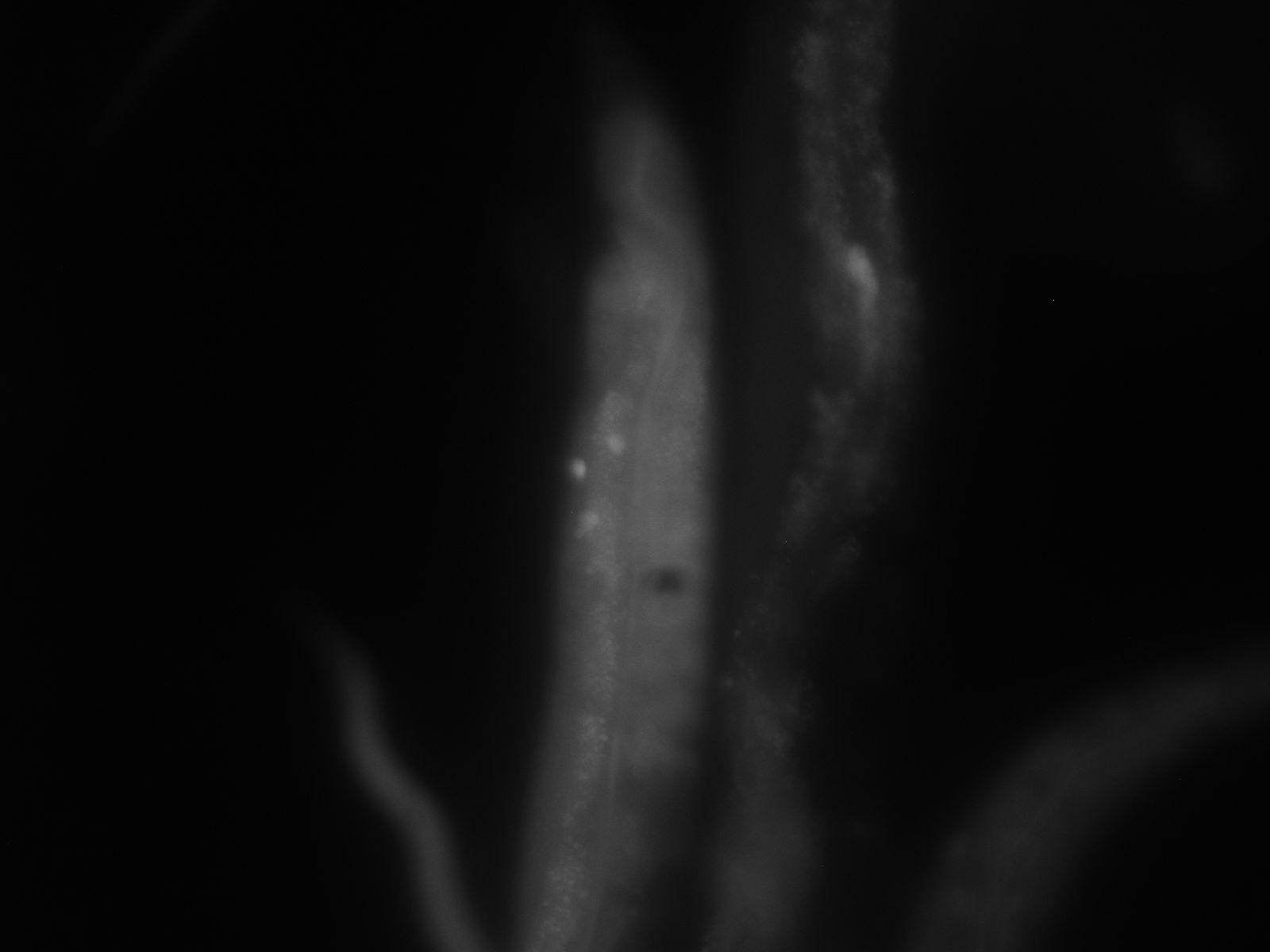

Supplement: S1 File — This file contains all the scoring data of the revised SYTO12 staining experiments. Each of the three biological replicates for Figs 2A, 4D, S2 and S4B–S4C were done in parallel in all strains. Hence, the wild type animals in Fig 2A and in S2 Fig are the same. In most cases animals were scored by live imaging without accompanied image acquisition. Representative images are provided. Consecutive images may image the same gonad. The scoring of apoptotic corpses was performed per gonad, not per image. (ZIP) [file pgen.1011061.s001.zip › SYTO staining experiment united/syto12 staining - 1_rep - 14.5.23 - JPEG/eat-4+tfg-1273.jpg]

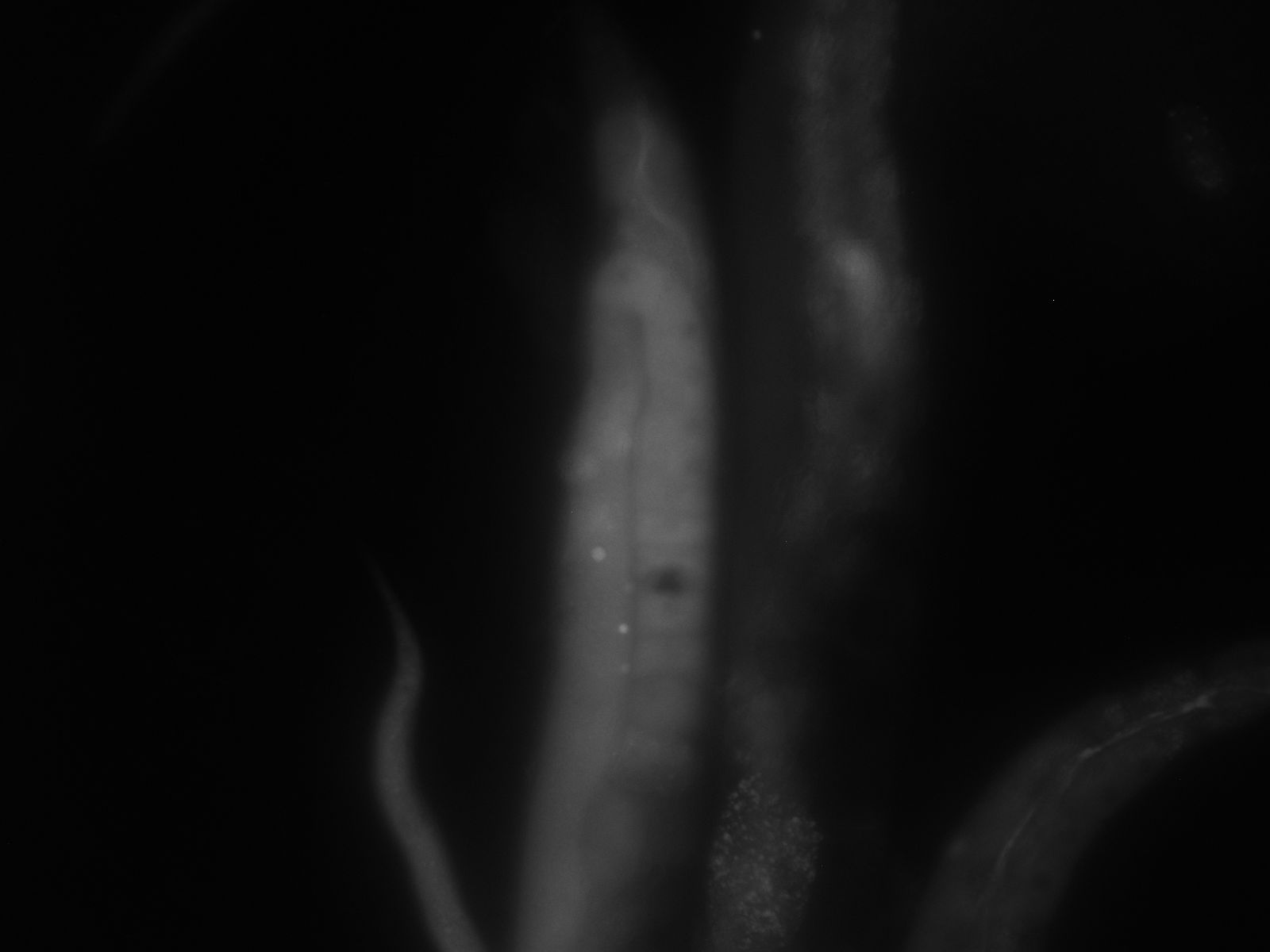

Supplement: S1 File — This file contains all the scoring data of the revised SYTO12 staining experiments. Each of the three biological replicates for Figs 2A, 4D, S2 and S4B–S4C were done in parallel in all strains. Hence, the wild type animals in Fig 2A and in S2 Fig are the same. In most cases animals were scored by live imaging without accompanied image acquisition. Representative images are provided. Consecutive images may image the same gonad. The scoring of apoptotic corpses was performed per gonad, not per image. (ZIP) [file pgen.1011061.s001.zip › SYTO staining experiment united/syto12 staining - 1_rep - 14.5.23 - JPEG/eat-4+tfg-1274.jpg]

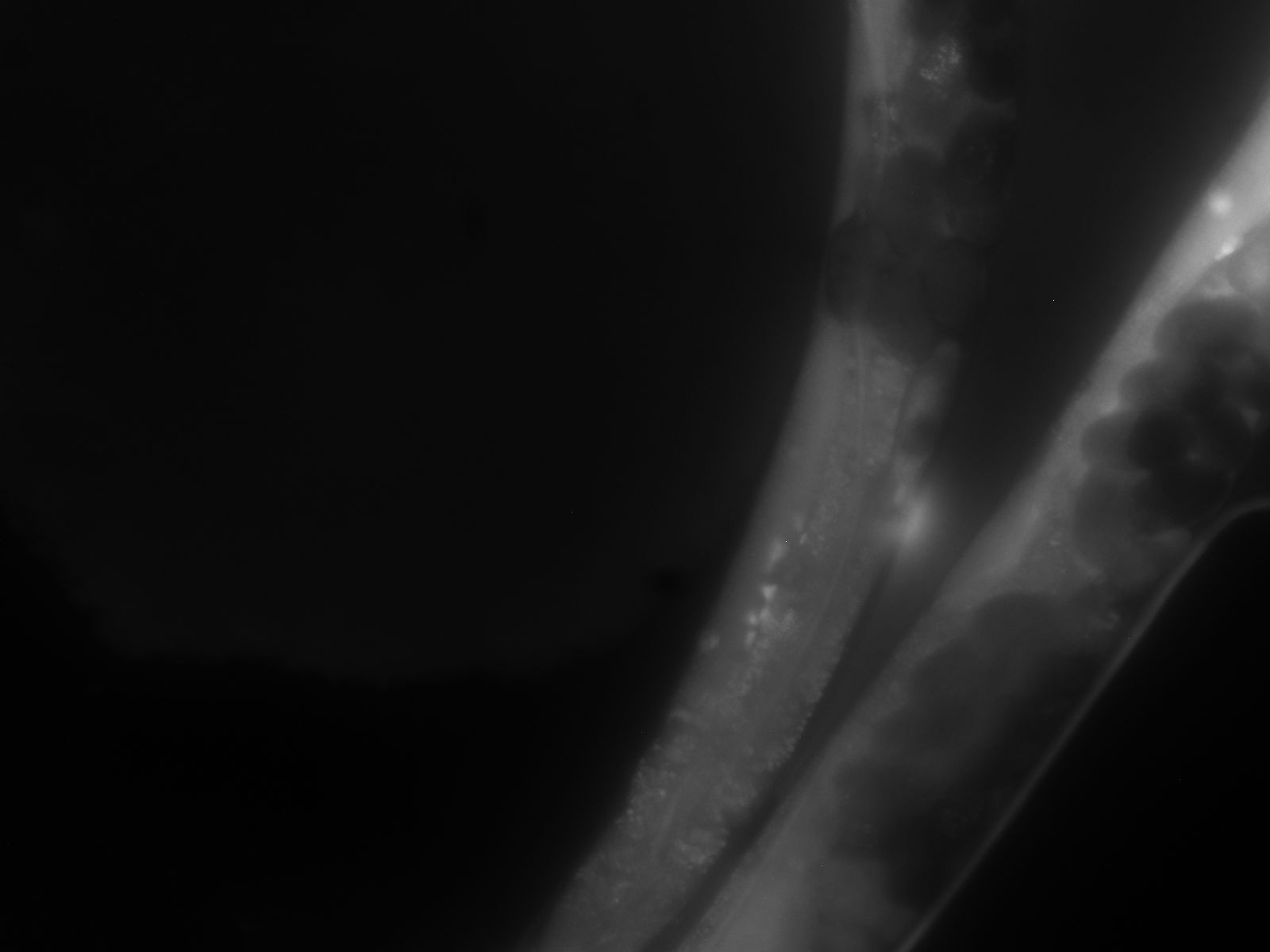

Supplement: S1 File — This file contains all the scoring data of the revised SYTO12 staining experiments. Each of the three biological replicates for Figs 2A, 4D, S2 and S4B–S4C were done in parallel in all strains. Hence, the wild type animals in Fig 2A and in S2 Fig are the same. In most cases animals were scored by live imaging without accompanied image acquisition. Representative images are provided. Consecutive images may image the same gonad. The scoring of apoptotic corpses was performed per gonad, not per image. (ZIP) [file pgen.1011061.s001.zip › SYTO staining experiment united/syto12 staining - 1_rep - 14.5.23 - JPEG/eat-4+tfg-1275.jpg]

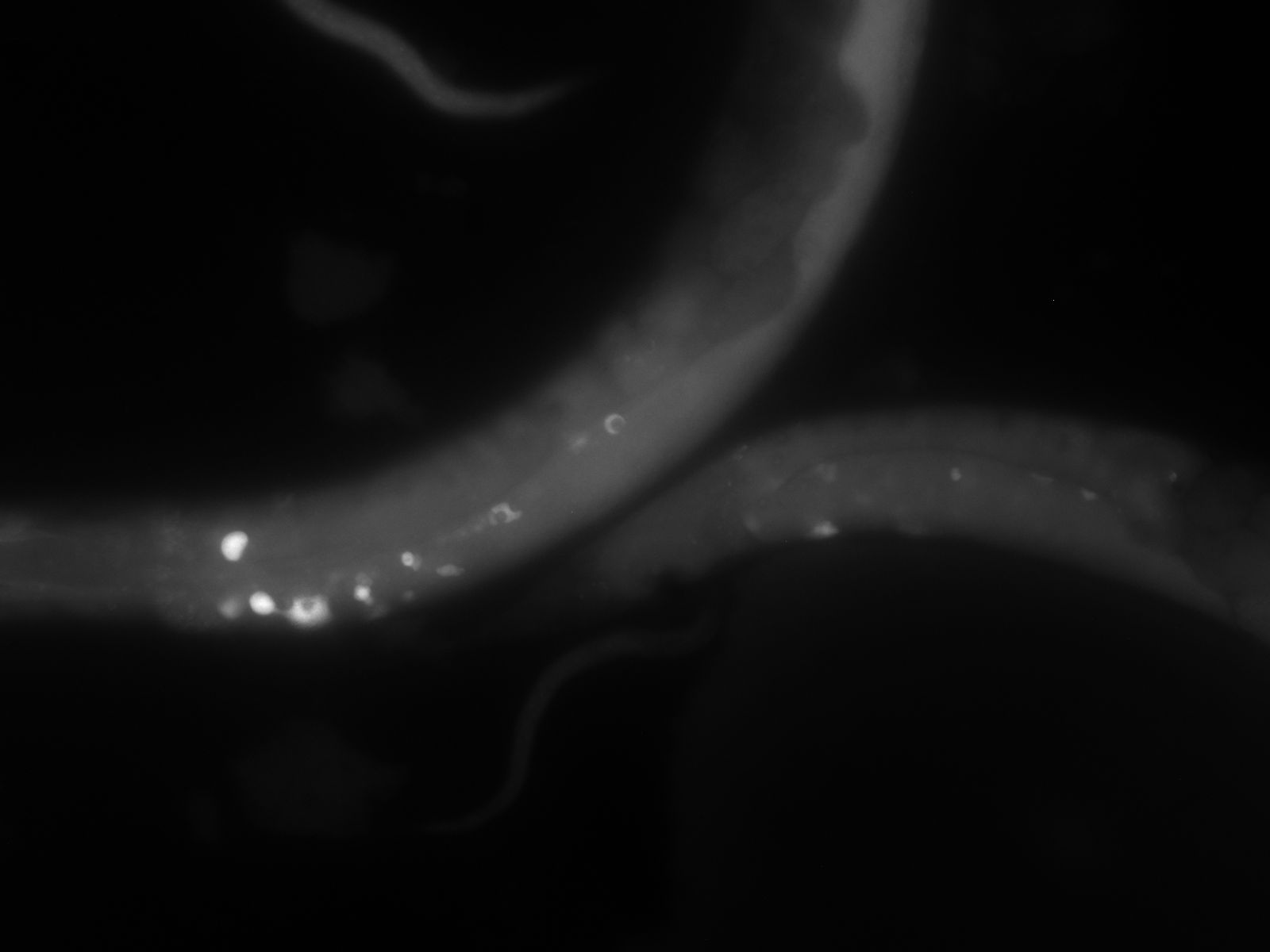

Supplement: S1 File — This file contains all the scoring data of the revised SYTO12 staining experiments. Each of the three biological replicates for Figs 2A, 4D, S2 and S4B–S4C were done in parallel in all strains. Hence, the wild type animals in Fig 2A and in S2 Fig are the same. In most cases animals were scored by live imaging without accompanied image acquisition. Representative images are provided. Consecutive images may image the same gonad. The scoring of apoptotic corpses was performed per gonad, not per image. (ZIP) [file pgen.1011061.s001.zip › SYTO staining experiment united/syto12 staining - 1_rep - 14.5.23 - JPEG/eat-4+tfg-1276.jpg]

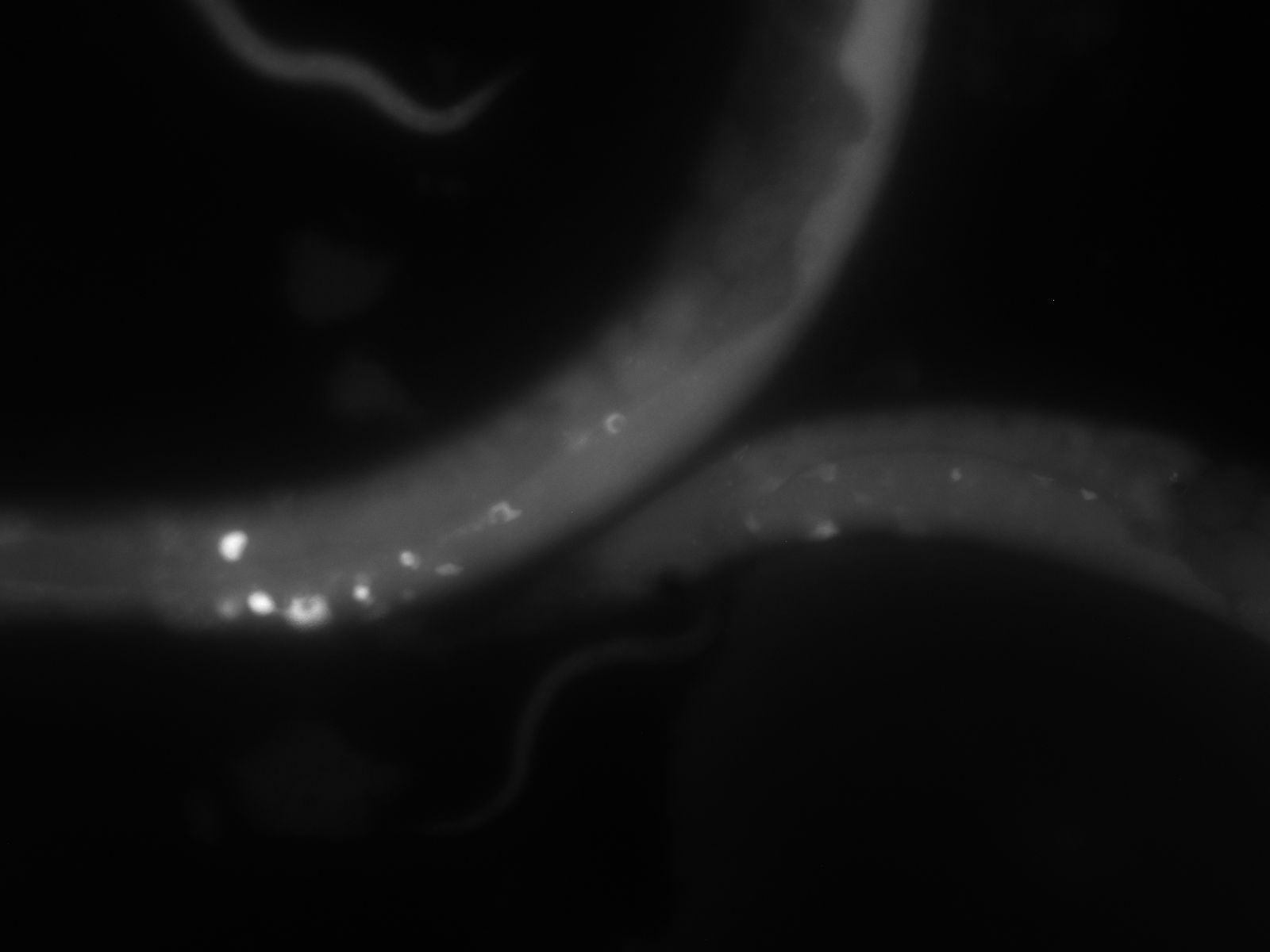

Supplement: S1 File — This file contains all the scoring data of the revised SYTO12 staining experiments. Each of the three biological replicates for Figs 2A, 4D, S2 and S4B–S4C were done in parallel in all strains. Hence, the wild type animals in Fig 2A and in S2 Fig are the same. In most cases animals were scored by live imaging without accompanied image acquisition. Representative images are provided. Consecutive images may image the same gonad. The scoring of apoptotic corpses was performed per gonad, not per image. (ZIP) [file pgen.1011061.s001.zip › SYTO staining experiment united/syto12 staining - 1_rep - 14.5.23 - JPEG/eat-4+tfg-1277.jpg]

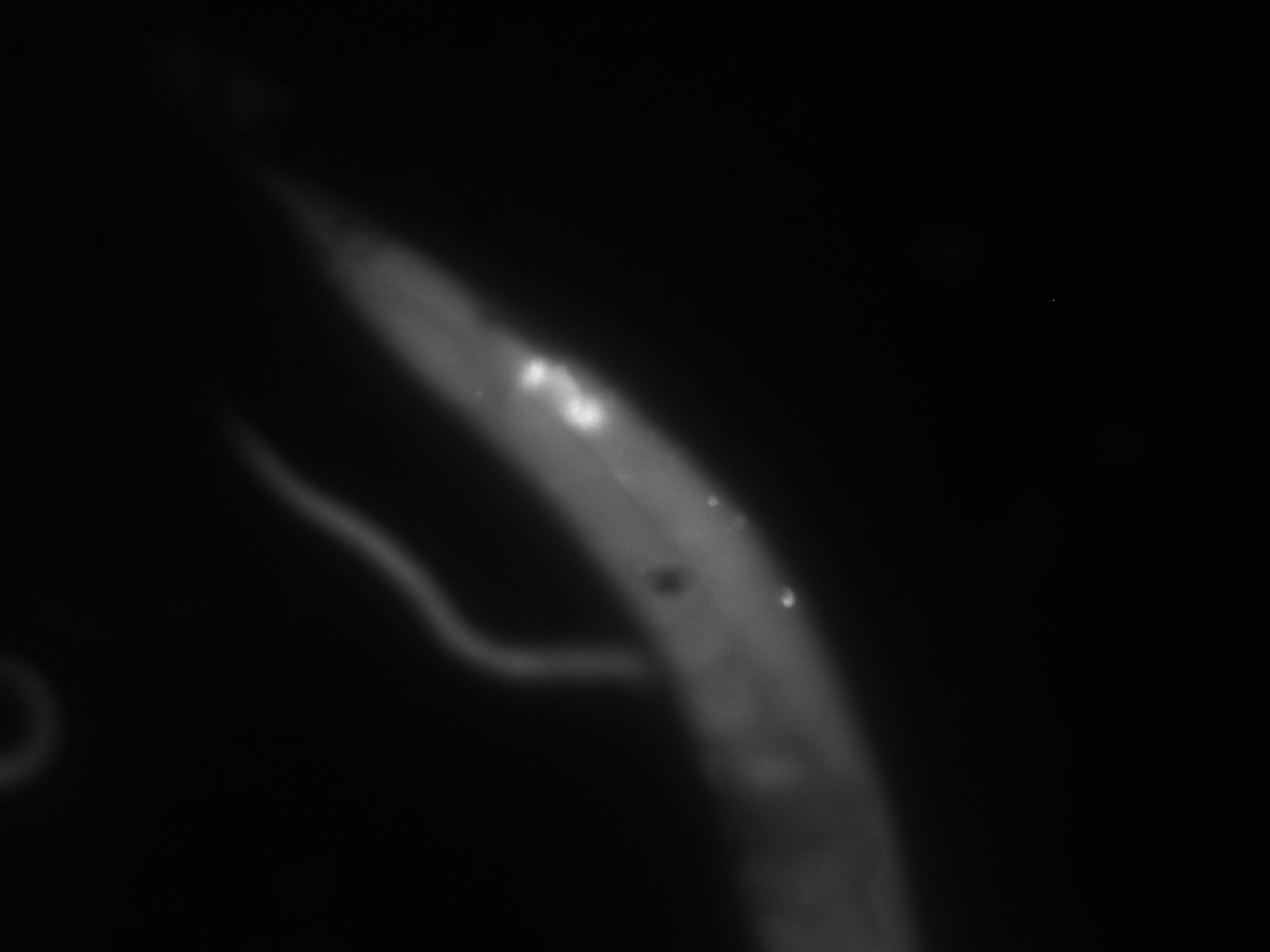

Supplement: S1 File — This file contains all the scoring data of the revised SYTO12 staining experiments. Each of the three biological replicates for Figs 2A, 4D, S2 and S4B–S4C were done in parallel in all strains. Hence, the wild type animals in Fig 2A and in S2 Fig are the same. In most cases animals were scored by live imaging without accompanied image acquisition. Representative images are provided. Consecutive images may image the same gonad. The scoring of apoptotic corpses was performed per gonad, not per image. (ZIP) [file pgen.1011061.s001.zip › SYTO staining experiment united/syto12 staining - 1_rep - 14.5.23 - JPEG/eat-4+tfg-1278.jpg]

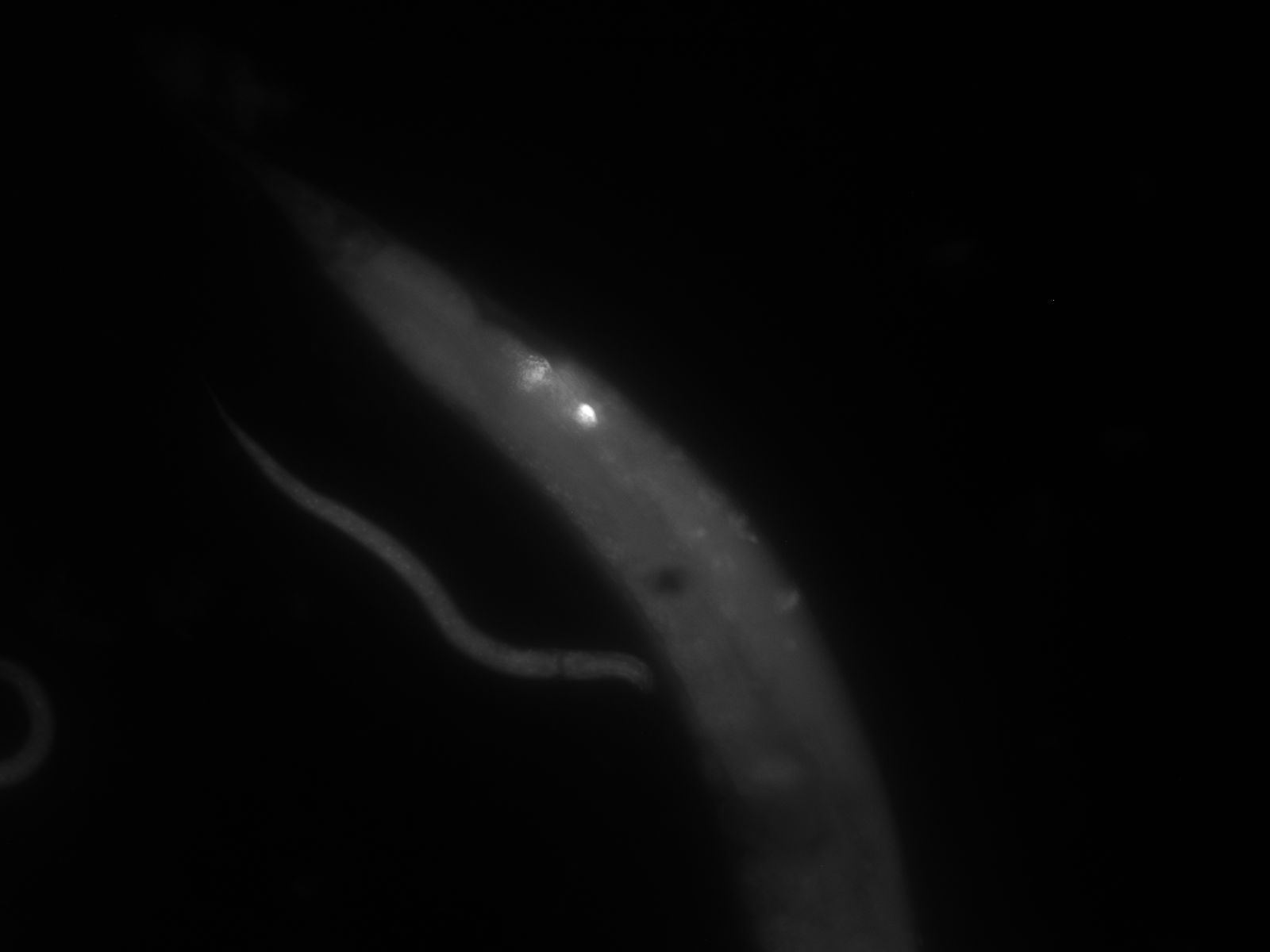

Supplement: S1 File — This file contains all the scoring data of the revised SYTO12 staining experiments. Each of the three biological replicates for Figs 2A, 4D, S2 and S4B–S4C were done in parallel in all strains. Hence, the wild type animals in Fig 2A and in S2 Fig are the same. In most cases animals were scored by live imaging without accompanied image acquisition. Representative images are provided. Consecutive images may image the same gonad. The scoring of apoptotic corpses was performed per gonad, not per image. (ZIP) [file pgen.1011061.s001.zip › SYTO staining experiment united/syto12 staining - 1_rep - 14.5.23 - JPEG/eat-4+tfg-1279.jpg]

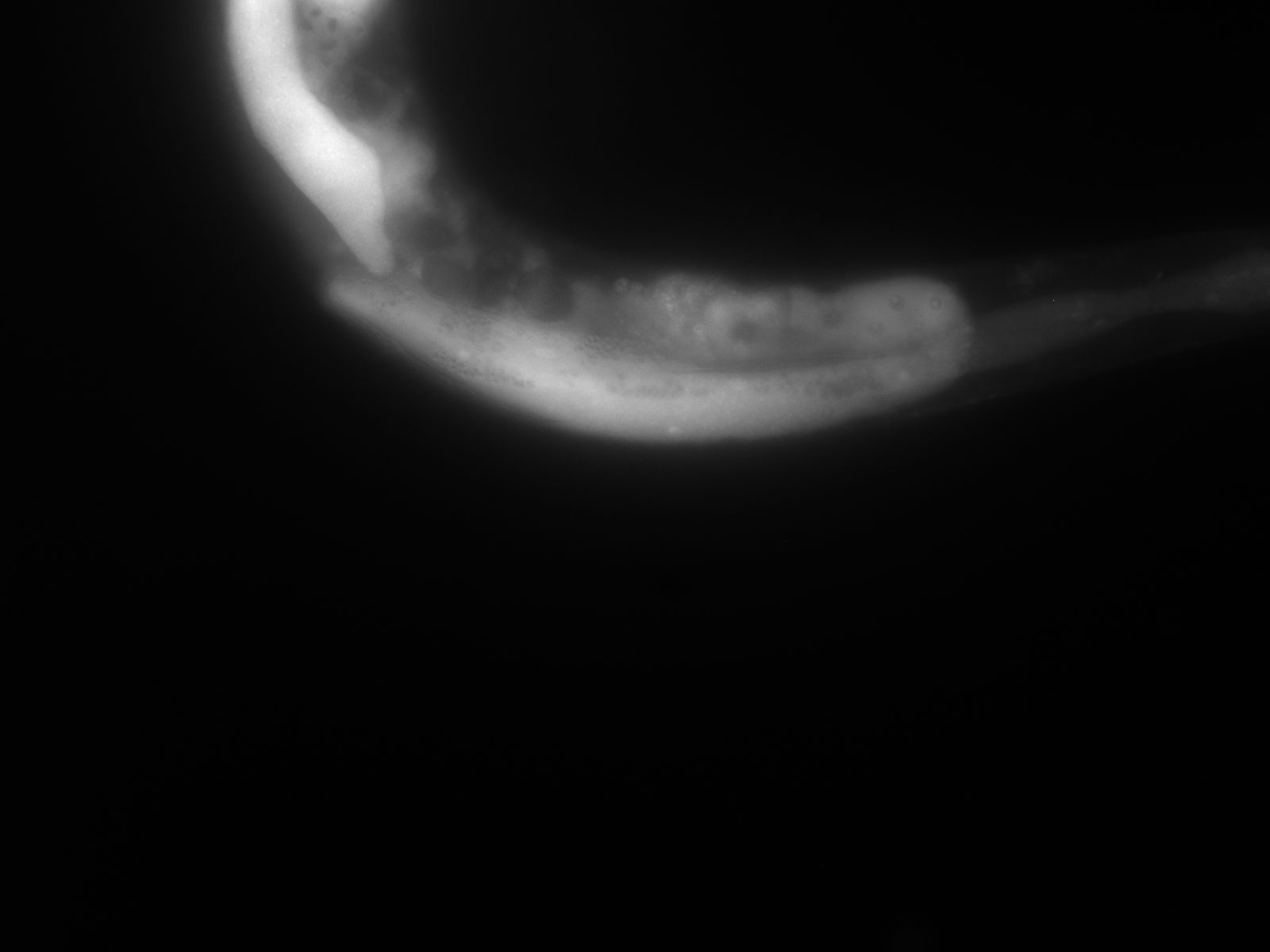

Supplement: S1 File — This file contains all the scoring data of the revised SYTO12 staining experiments. Each of the three biological replicates for Figs 2A, 4D, S2 and S4B–S4C were done in parallel in all strains. Hence, the wild type animals in Fig 2A and in S2 Fig are the same. In most cases animals were scored by live imaging without accompanied image acquisition. Representative images are provided. Consecutive images may image the same gonad. The scoring of apoptotic corpses was performed per gonad, not per image. (ZIP) [file pgen.1011061.s001.zip › SYTO staining experiment united/syto12 staining - 1_rep - 14.5.23 - JPEG/ire-1+pad12118.jpg]

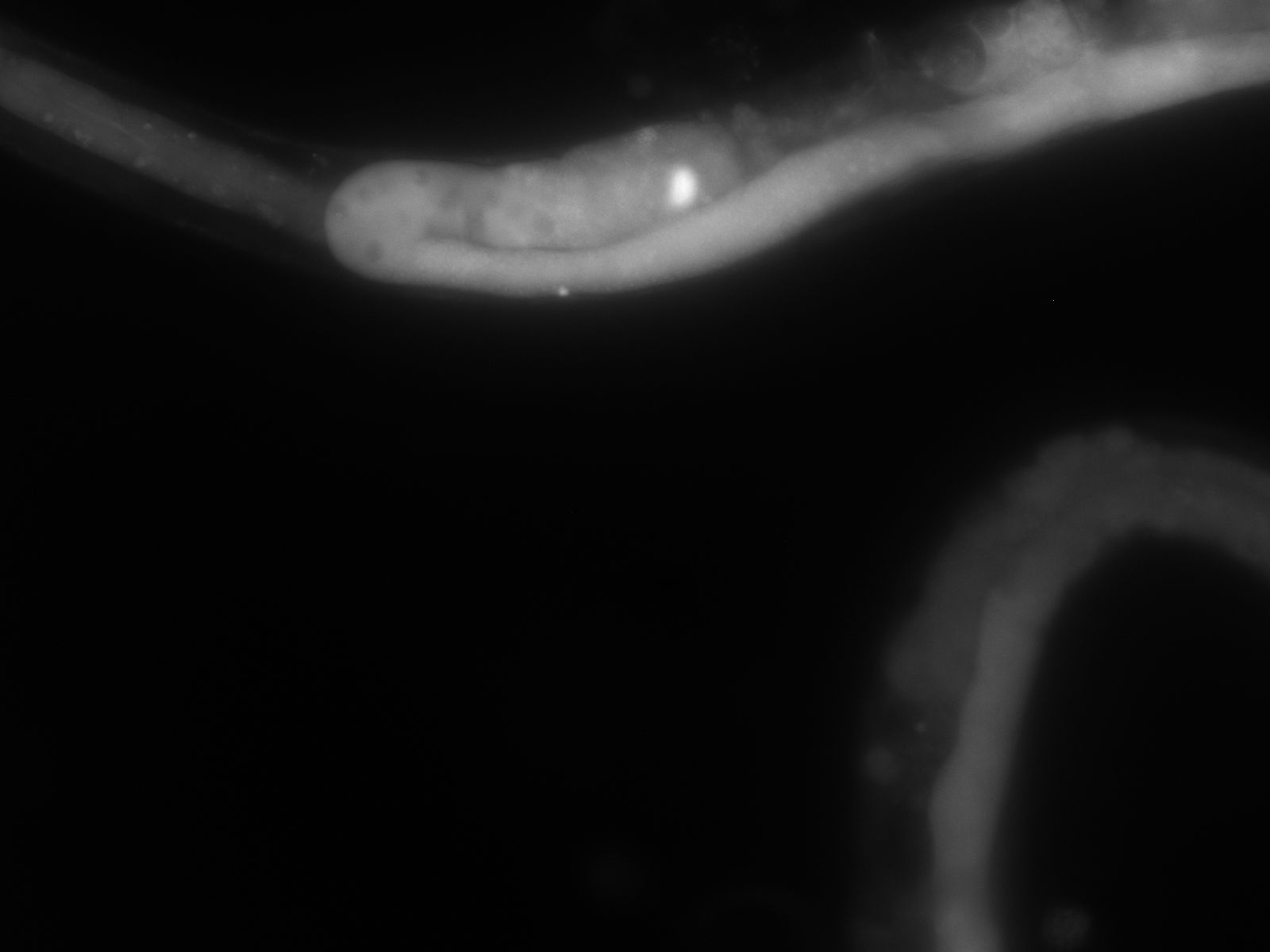

Supplement: S1 File — This file contains all the scoring data of the revised SYTO12 staining experiments. Each of the three biological replicates for Figs 2A, 4D, S2 and S4B–S4C were done in parallel in all strains. Hence, the wild type animals in Fig 2A and in S2 Fig are the same. In most cases animals were scored by live imaging without accompanied image acquisition. Representative images are provided. Consecutive images may image the same gonad. The scoring of apoptotic corpses was performed per gonad, not per image. (ZIP) [file pgen.1011061.s001.zip › SYTO staining experiment united/syto12 staining - 1_rep - 14.5.23 - JPEG/ire-1+pad12119.jpg]

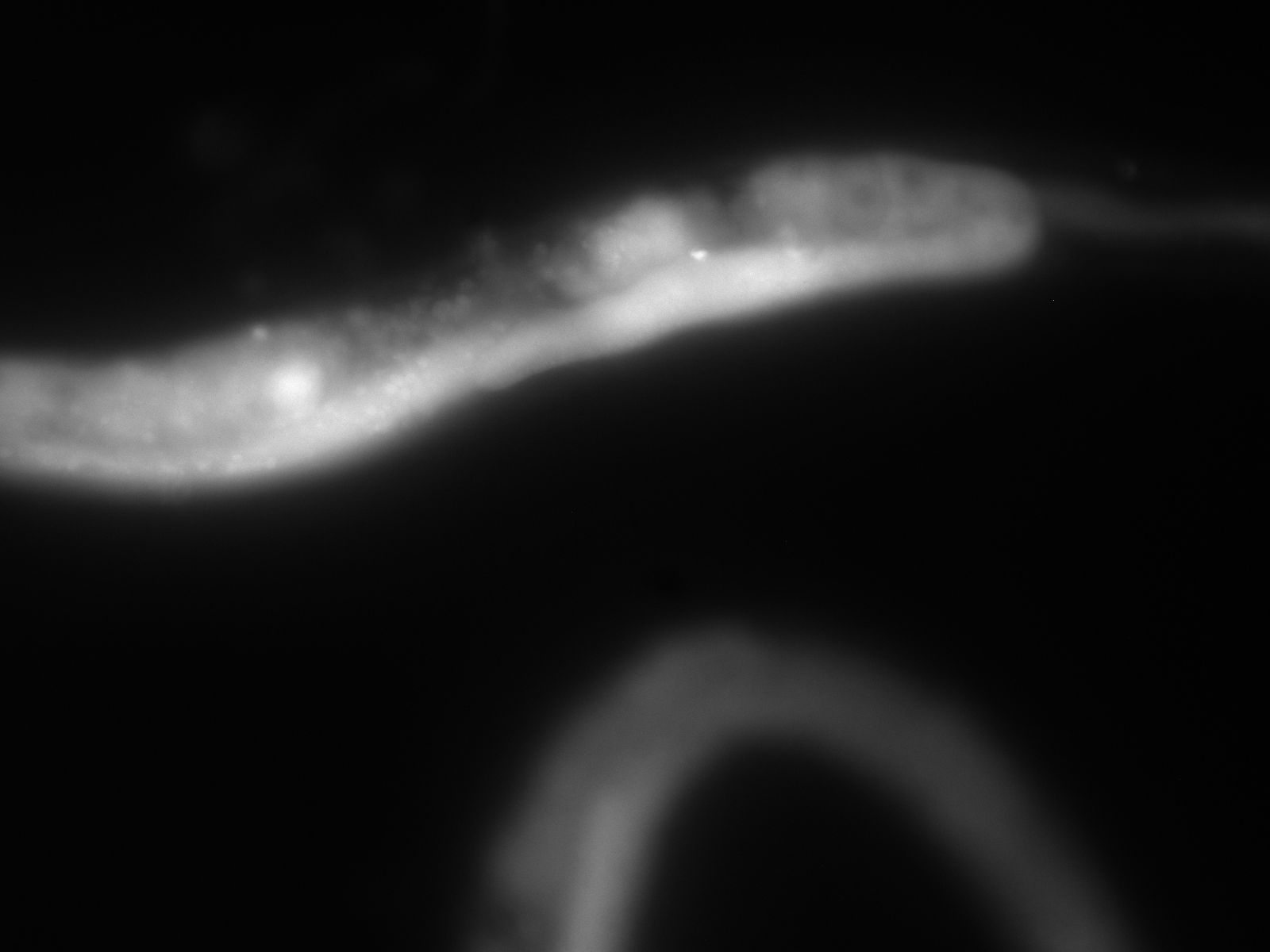

Supplement: S1 File — This file contains all the scoring data of the revised SYTO12 staining experiments. Each of the three biological replicates for Figs 2A, 4D, S2 and S4B–S4C were done in parallel in all strains. Hence, the wild type animals in Fig 2A and in S2 Fig are the same. In most cases animals were scored by live imaging without accompanied image acquisition. Representative images are provided. Consecutive images may image the same gonad. The scoring of apoptotic corpses was performed per gonad, not per image. (ZIP) [file pgen.1011061.s001.zip › SYTO staining experiment united/syto12 staining - 1_rep - 14.5.23 - JPEG/ire-1+pad12120.jpg]

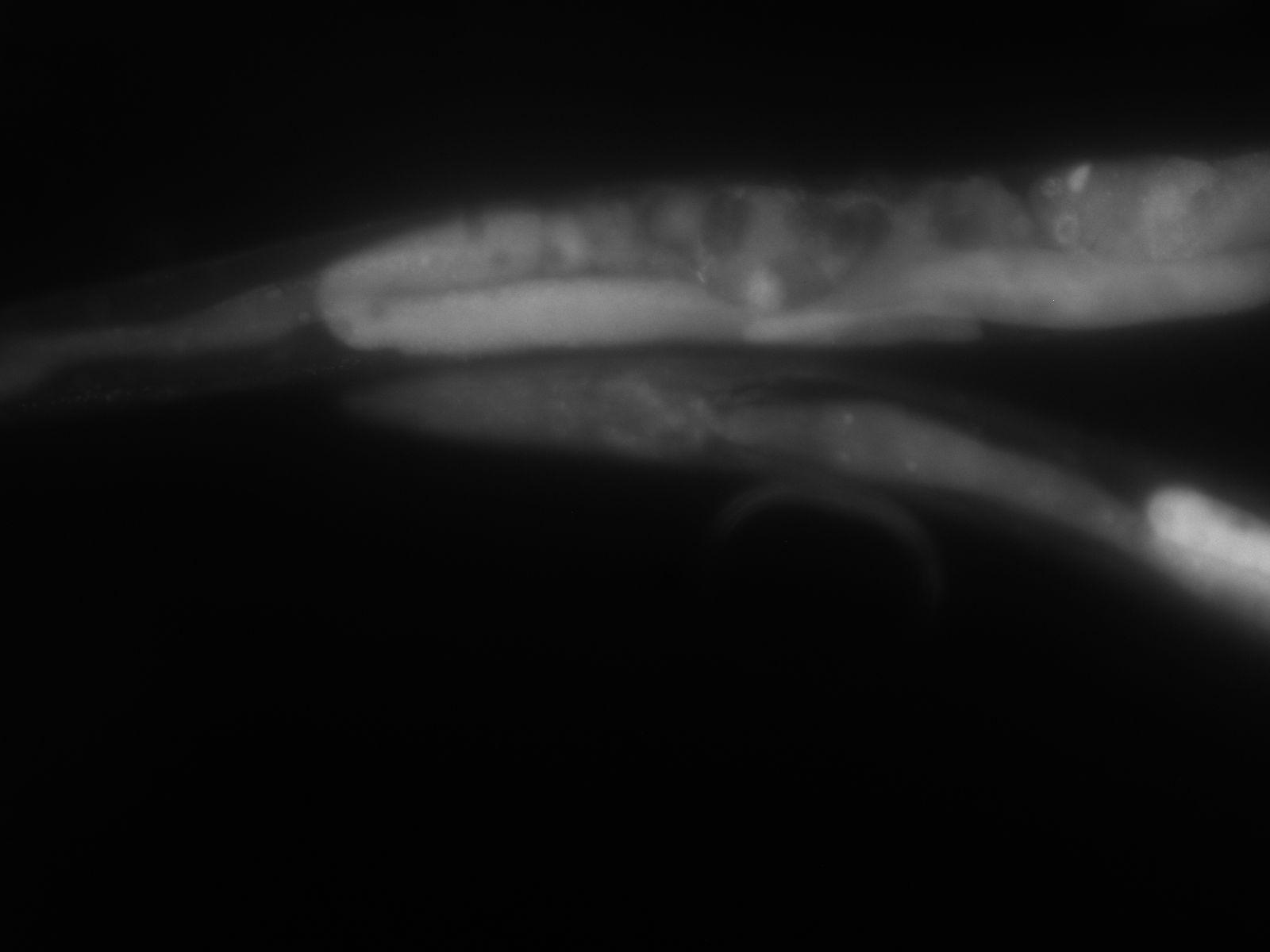

Supplement: S1 File — This file contains all the scoring data of the revised SYTO12 staining experiments. Each of the three biological replicates for Figs 2A, 4D, S2 and S4B–S4C were done in parallel in all strains. Hence, the wild type animals in Fig 2A and in S2 Fig are the same. In most cases animals were scored by live imaging without accompanied image acquisition. Representative images are provided. Consecutive images may image the same gonad. The scoring of apoptotic corpses was performed per gonad, not per image. (ZIP) [file pgen.1011061.s001.zip › SYTO staining experiment united/syto12 staining - 1_rep - 14.5.23 - JPEG/ire-1+pad12121.jpg]

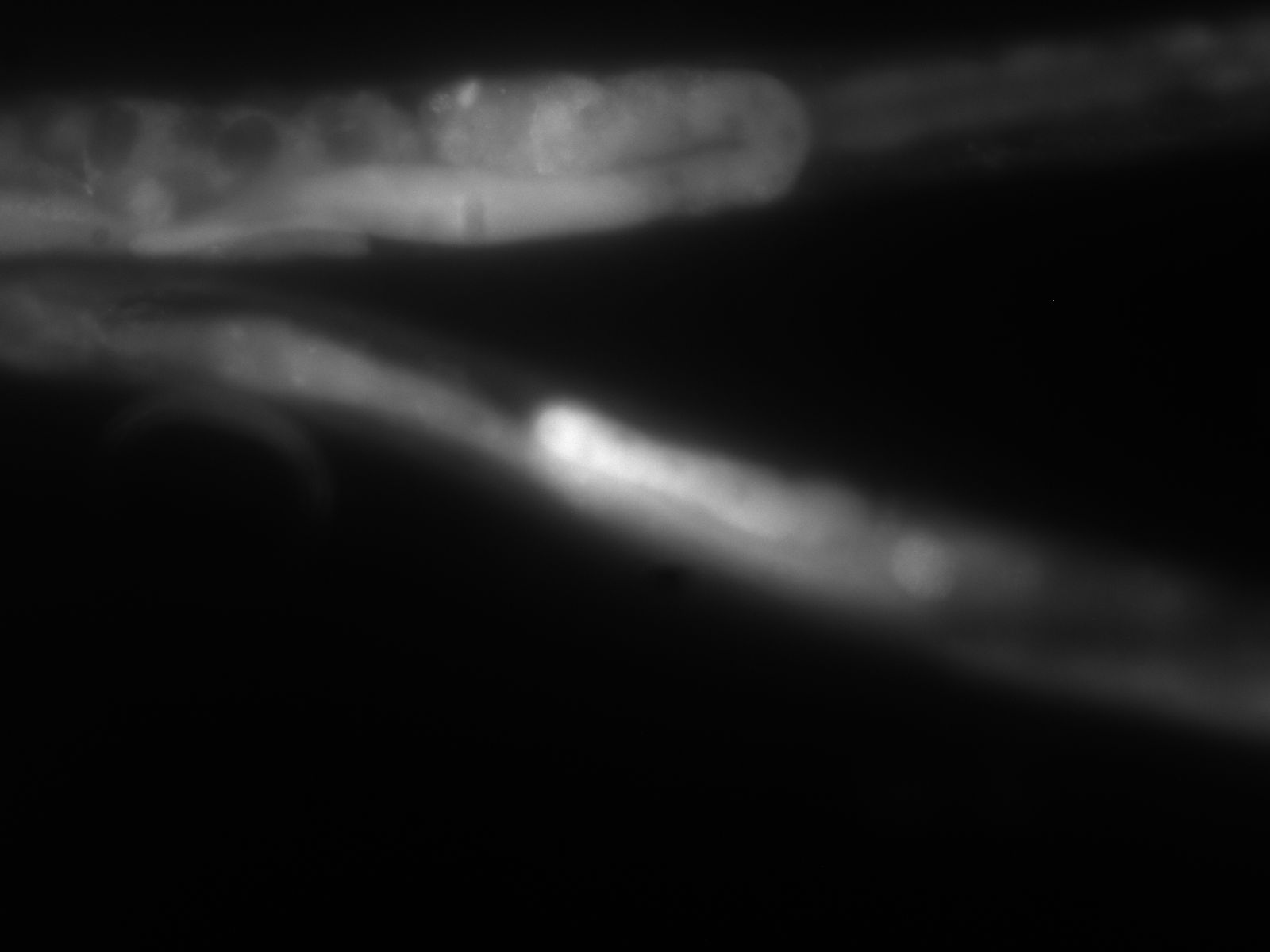

Supplement: S1 File — This file contains all the scoring data of the revised SYTO12 staining experiments. Each of the three biological replicates for Figs 2A, 4D, S2 and S4B–S4C were done in parallel in all strains. Hence, the wild type animals in Fig 2A and in S2 Fig are the same. In most cases animals were scored by live imaging without accompanied image acquisition. Representative images are provided. Consecutive images may image the same gonad. The scoring of apoptotic corpses was performed per gonad, not per image. (ZIP) [file pgen.1011061.s001.zip › SYTO staining experiment united/syto12 staining - 1_rep - 14.5.23 - JPEG/ire-1+pad12122.jpg]

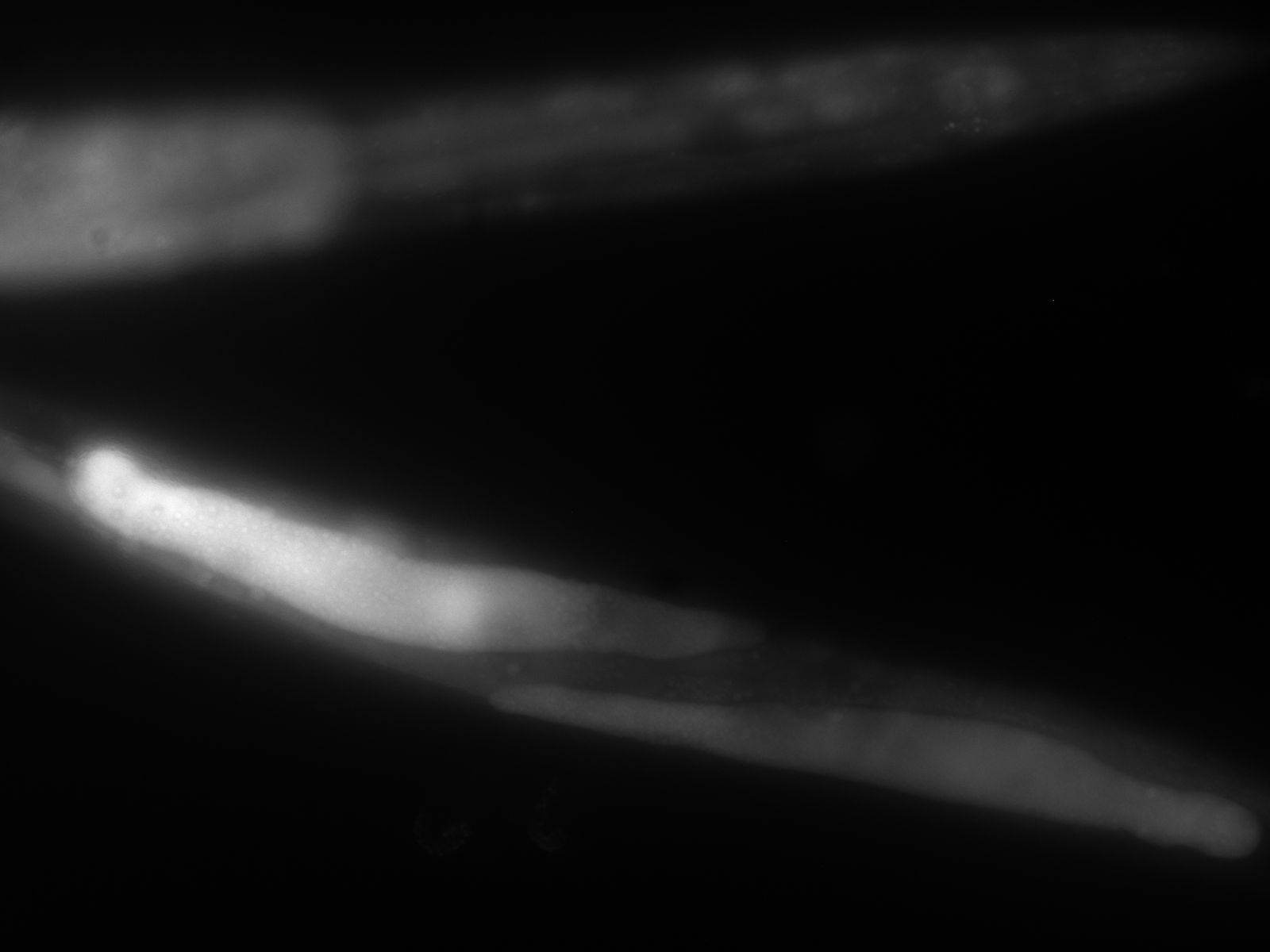

Supplement: S1 File — This file contains all the scoring data of the revised SYTO12 staining experiments. Each of the three biological replicates for Figs 2A, 4D, S2 and S4B–S4C were done in parallel in all strains. Hence, the wild type animals in Fig 2A and in S2 Fig are the same. In most cases animals were scored by live imaging without accompanied image acquisition. Representative images are provided. Consecutive images may image the same gonad. The scoring of apoptotic corpses was performed per gonad, not per image. (ZIP) [file pgen.1011061.s001.zip › SYTO staining experiment united/syto12 staining - 1_rep - 14.5.23 - JPEG/ire-1+pad12123.jpg]

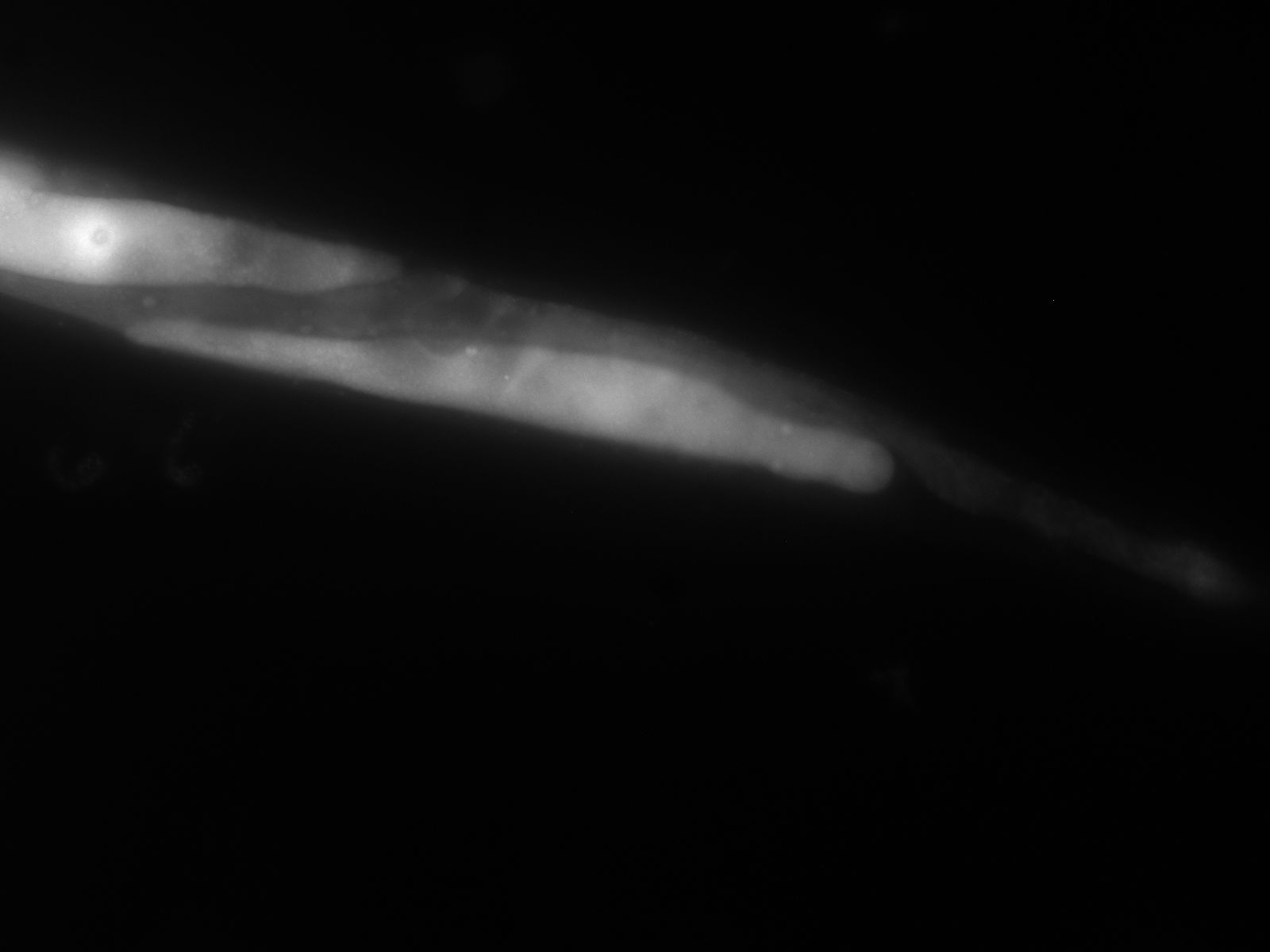

Supplement: S1 File — This file contains all the scoring data of the revised SYTO12 staining experiments. Each of the three biological replicates for Figs 2A, 4D, S2 and S4B–S4C were done in parallel in all strains. Hence, the wild type animals in Fig 2A and in S2 Fig are the same. In most cases animals were scored by live imaging without accompanied image acquisition. Representative images are provided. Consecutive images may image the same gonad. The scoring of apoptotic corpses was performed per gonad, not per image. (ZIP) [file pgen.1011061.s001.zip › SYTO staining experiment united/syto12 staining - 1_rep - 14.5.23 - JPEG/ire-1+pad12124.jpg]

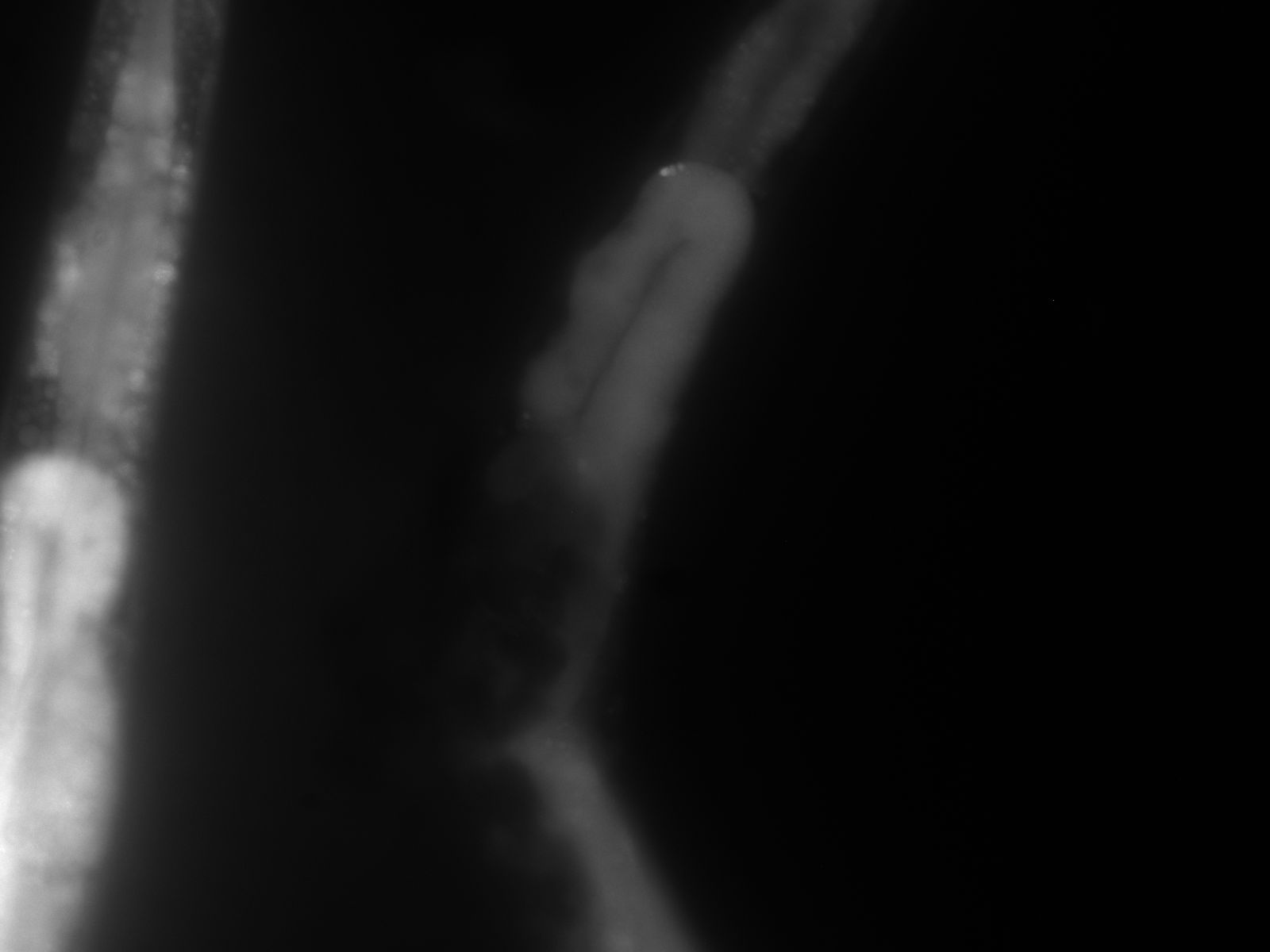

Supplement: S1 File — This file contains all the scoring data of the revised SYTO12 staining experiments. Each of the three biological replicates for Figs 2A, 4D, S2 and S4B–S4C were done in parallel in all strains. Hence, the wild type animals in Fig 2A and in S2 Fig are the same. In most cases animals were scored by live imaging without accompanied image acquisition. Representative images are provided. Consecutive images may image the same gonad. The scoring of apoptotic corpses was performed per gonad, not per image. (ZIP) [file pgen.1011061.s001.zip › SYTO staining experiment united/syto12 staining - 1_rep - 14.5.23 - JPEG/ire-1+pad12125.jpg]

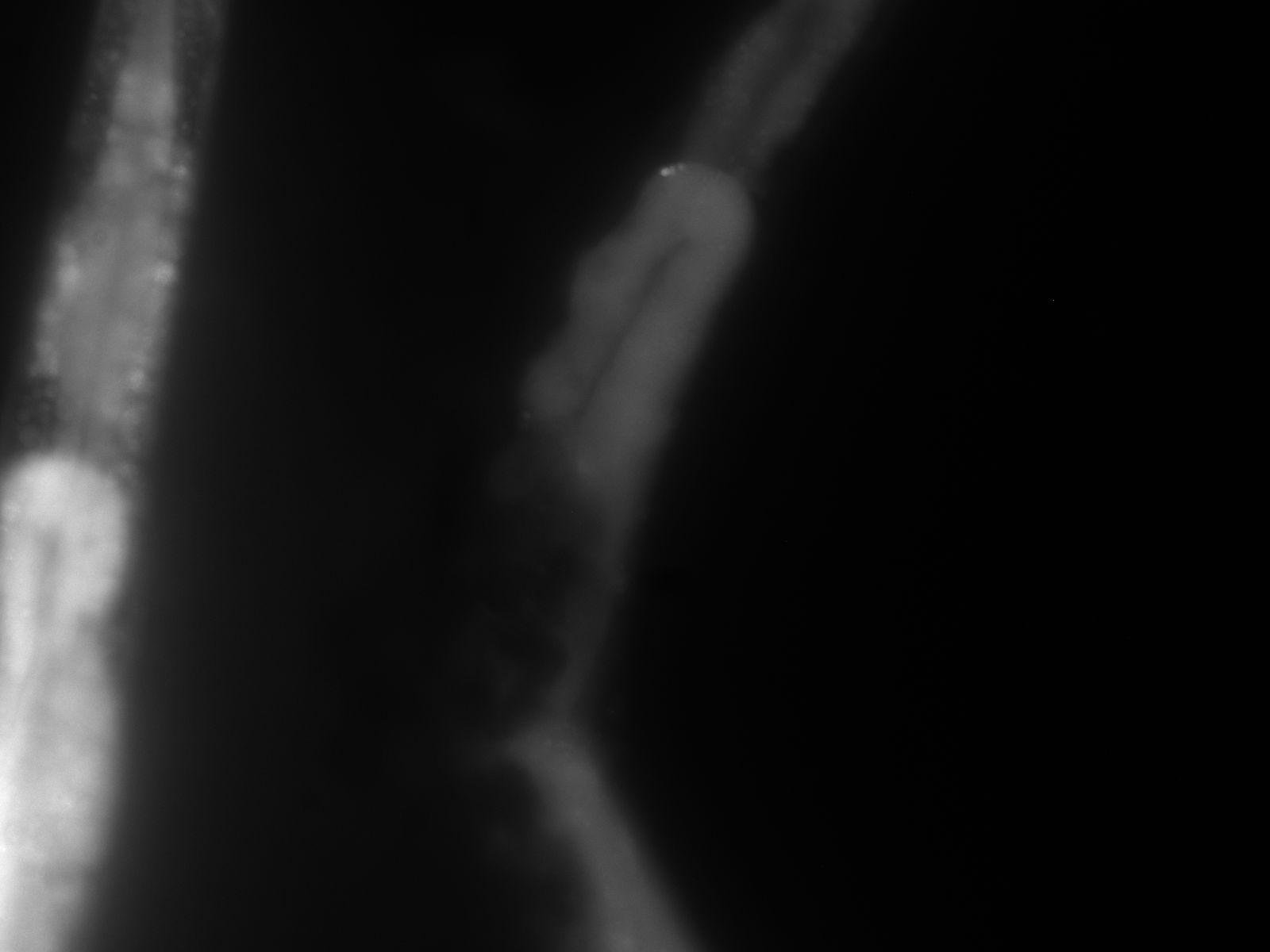

Supplement: S1 File — This file contains all the scoring data of the revised SYTO12 staining experiments. Each of the three biological replicates for Figs 2A, 4D, S2 and S4B–S4C were done in parallel in all strains. Hence, the wild type animals in Fig 2A and in S2 Fig are the same. In most cases animals were scored by live imaging without accompanied image acquisition. Representative images are provided. Consecutive images may image the same gonad. The scoring of apoptotic corpses was performed per gonad, not per image. (ZIP) [file pgen.1011061.s001.zip › SYTO staining experiment united/syto12 staining - 1_rep - 14.5.23 - JPEG/ire-1+pad12126.jpg]

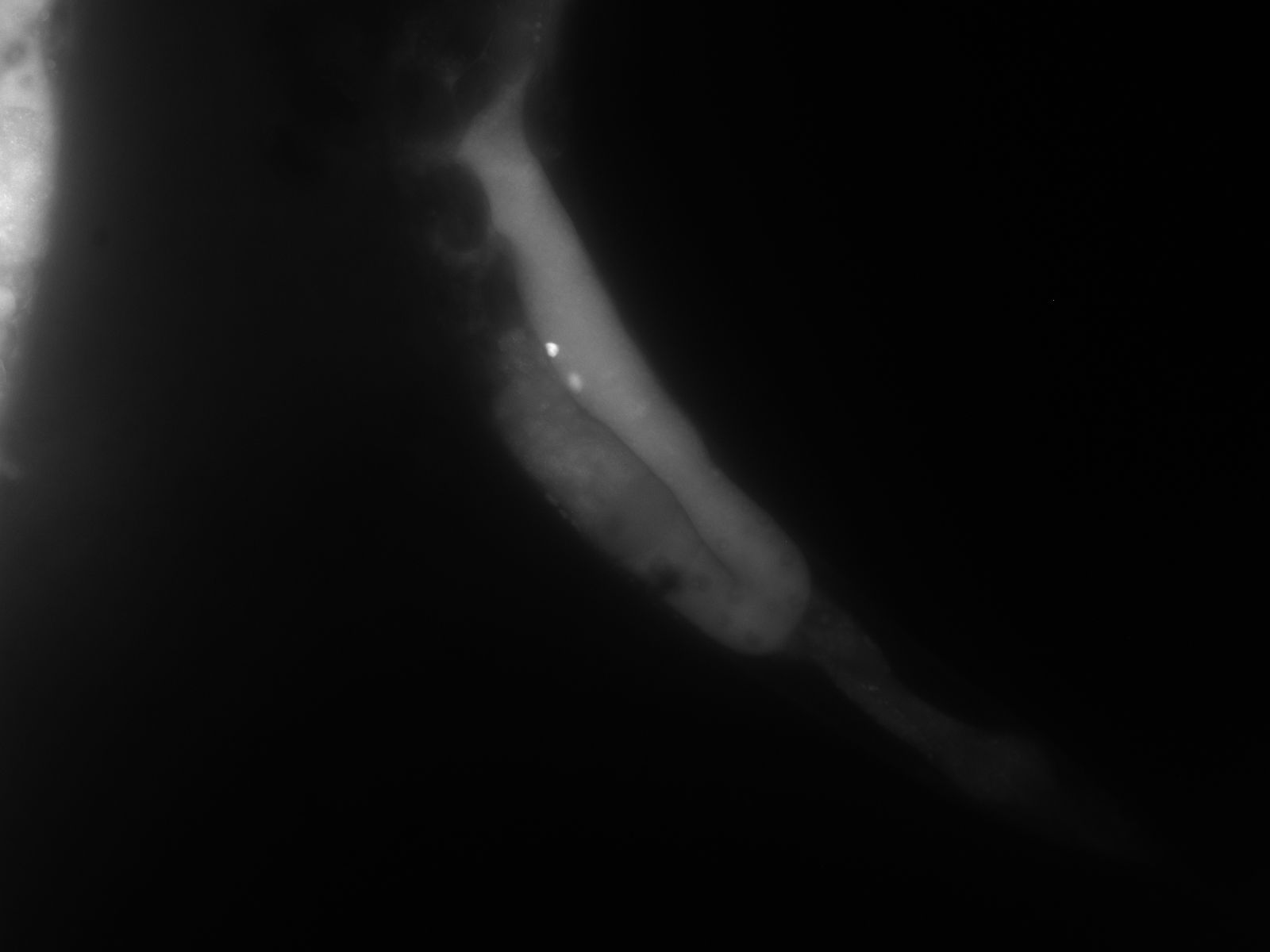

Supplement: S1 File — This file contains all the scoring data of the revised SYTO12 staining experiments. Each of the three biological replicates for Figs 2A, 4D, S2 and S4B–S4C were done in parallel in all strains. Hence, the wild type animals in Fig 2A and in S2 Fig are the same. In most cases animals were scored by live imaging without accompanied image acquisition. Representative images are provided. Consecutive images may image the same gonad. The scoring of apoptotic corpses was performed per gonad, not per image. (ZIP) [file pgen.1011061.s001.zip › SYTO staining experiment united/syto12 staining - 1_rep - 14.5.23 - JPEG/ire-1+pad12127.jpg]

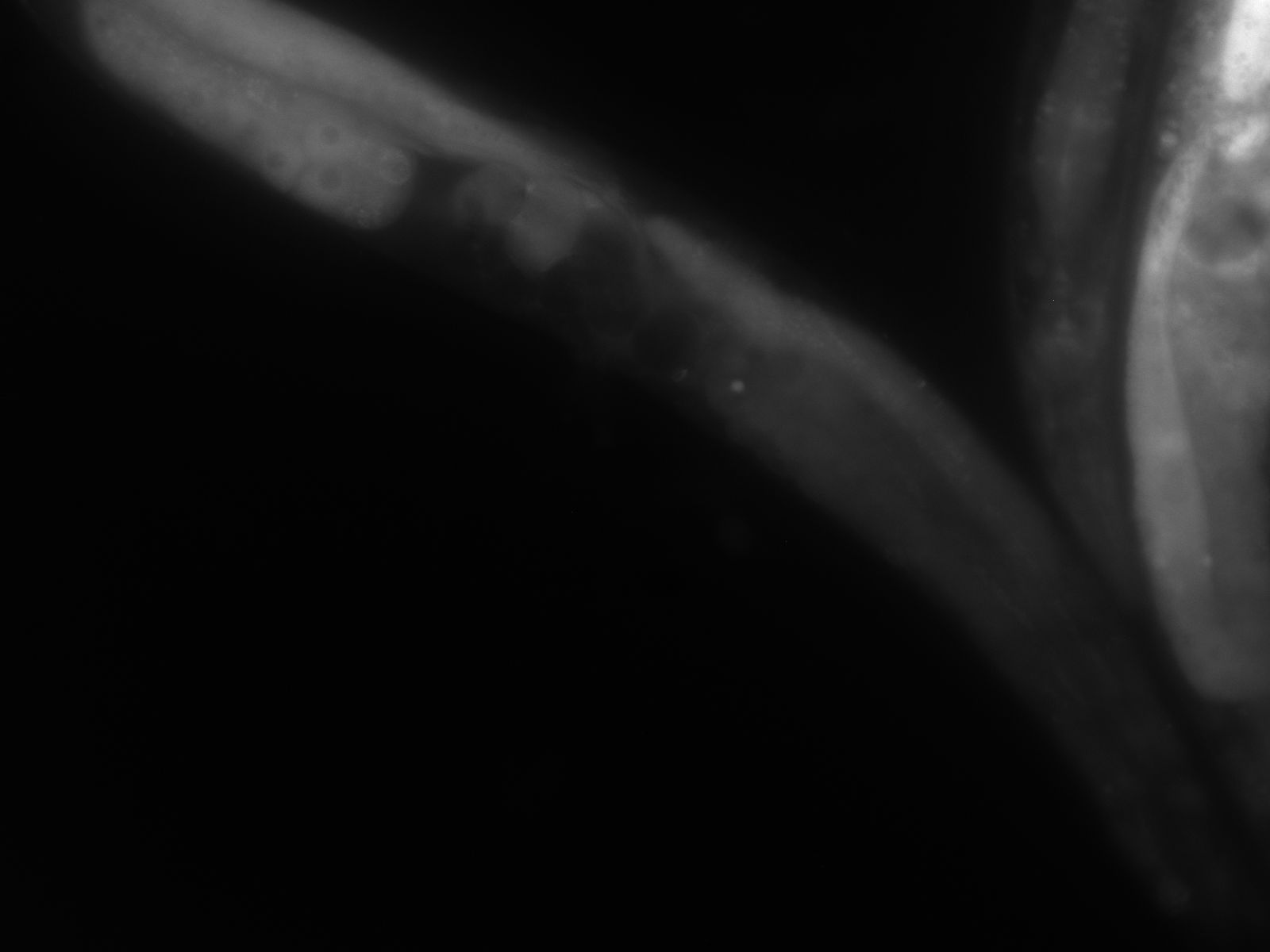

Supplement: S1 File — This file contains all the scoring data of the revised SYTO12 staining experiments. Each of the three biological replicates for Figs 2A, 4D, S2 and S4B–S4C were done in parallel in all strains. Hence, the wild type animals in Fig 2A and in S2 Fig are the same. In most cases animals were scored by live imaging without accompanied image acquisition. Representative images are provided. Consecutive images may image the same gonad. The scoring of apoptotic corpses was performed per gonad, not per image. (ZIP) [file pgen.1011061.s001.zip › SYTO staining experiment united/syto12 staining - 1_rep - 14.5.23 - JPEG/ire-1+pad12128.jpg]

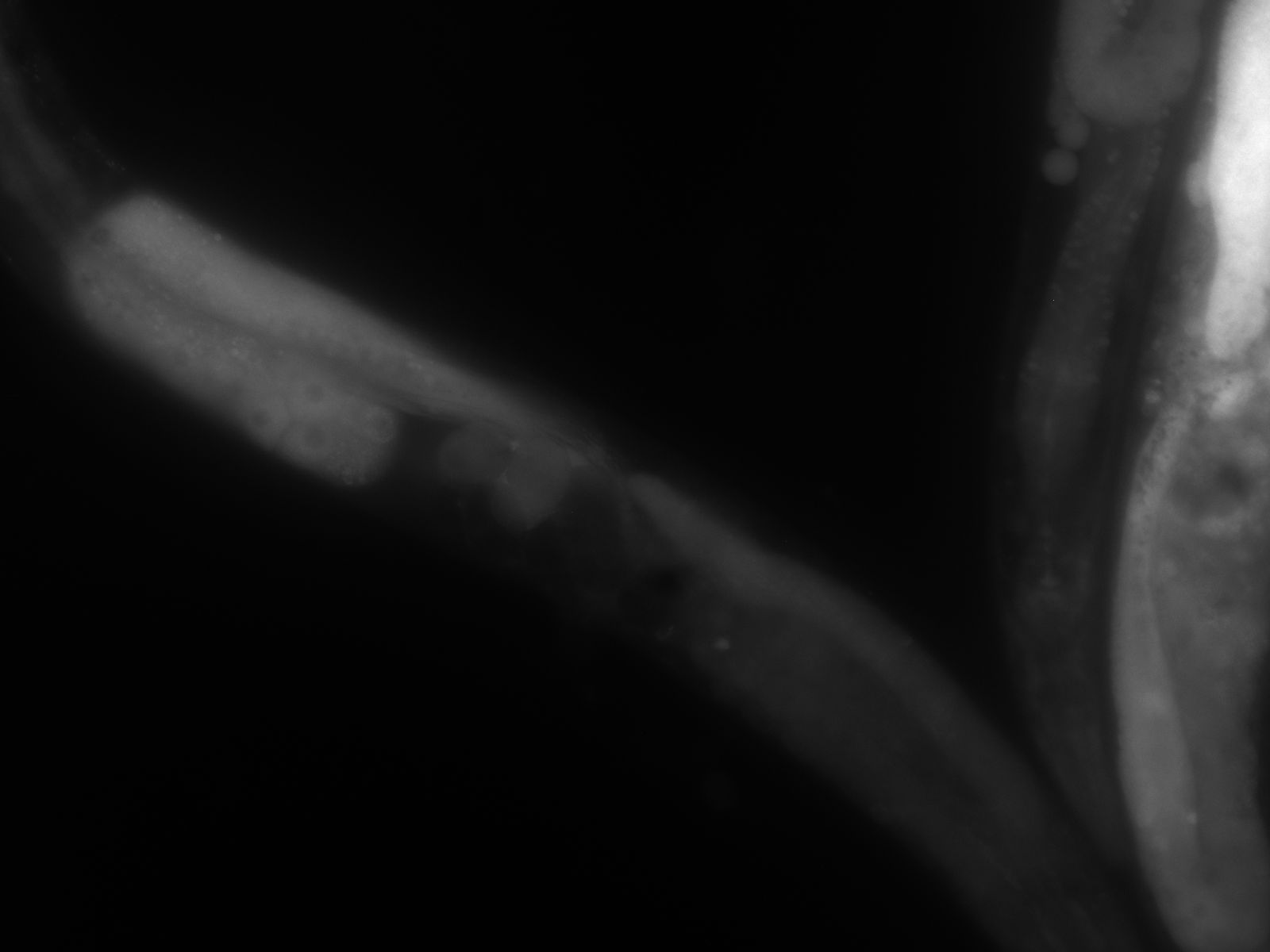

Supplement: S1 File — This file contains all the scoring data of the revised SYTO12 staining experiments. Each of the three biological replicates for Figs 2A, 4D, S2 and S4B–S4C were done in parallel in all strains. Hence, the wild type animals in Fig 2A and in S2 Fig are the same. In most cases animals were scored by live imaging without accompanied image acquisition. Representative images are provided. Consecutive images may image the same gonad. The scoring of apoptotic corpses was performed per gonad, not per image. (ZIP) [file pgen.1011061.s001.zip › SYTO staining experiment united/syto12 staining - 1_rep - 14.5.23 - JPEG/ire-1+pad12129.jpg]

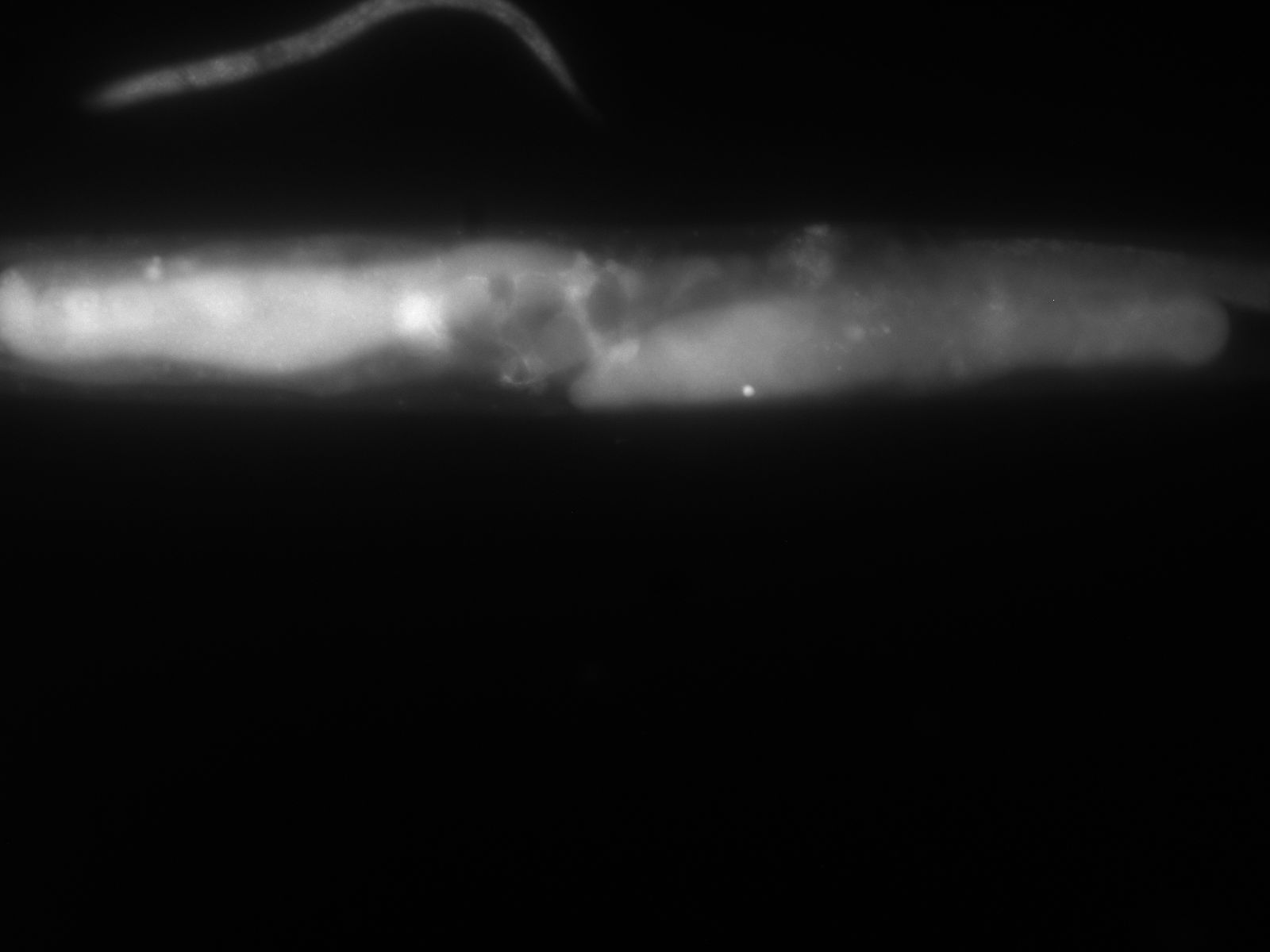

Supplement: S1 File — This file contains all the scoring data of the revised SYTO12 staining experiments. Each of the three biological replicates for Figs 2A, 4D, S2 and S4B–S4C were done in parallel in all strains. Hence, the wild type animals in Fig 2A and in S2 Fig are the same. In most cases animals were scored by live imaging without accompanied image acquisition. Representative images are provided. Consecutive images may image the same gonad. The scoring of apoptotic corpses was performed per gonad, not per image. (ZIP) [file pgen.1011061.s001.zip › SYTO staining experiment united/syto12 staining - 1_rep - 14.5.23 - JPEG/ire-1+pad12130.jpg]

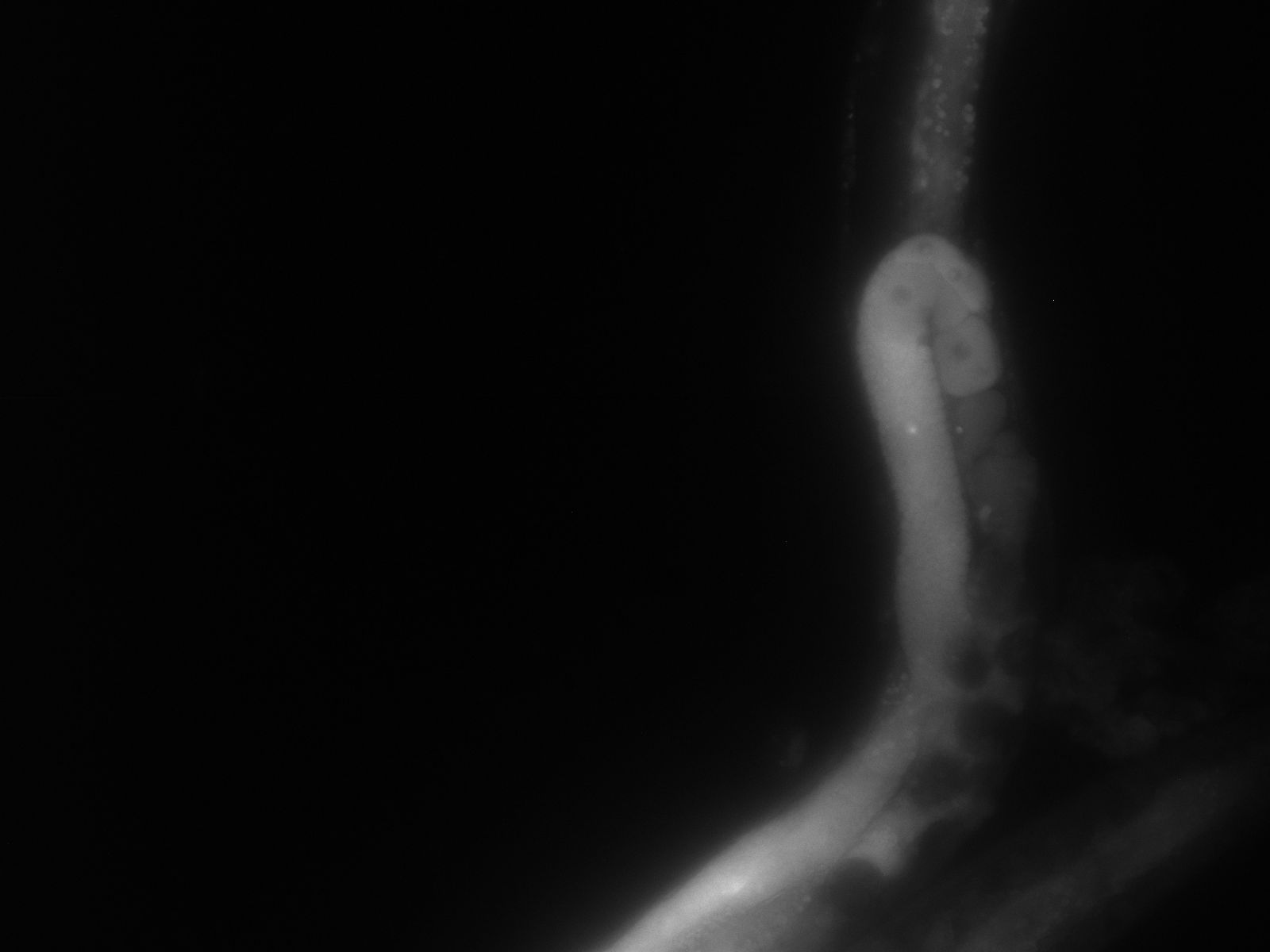

Supplement: S1 File — This file contains all the scoring data of the revised SYTO12 staining experiments. Each of the three biological replicates for Figs 2A, 4D, S2 and S4B–S4C were done in parallel in all strains. Hence, the wild type animals in Fig 2A and in S2 Fig are the same. In most cases animals were scored by live imaging without accompanied image acquisition. Representative images are provided. Consecutive images may image the same gonad. The scoring of apoptotic corpses was performed per gonad, not per image. (ZIP) [file pgen.1011061.s001.zip › SYTO staining experiment united/syto12 staining - 1_rep - 14.5.23 - JPEG/ire-1+pad12131.jpg]

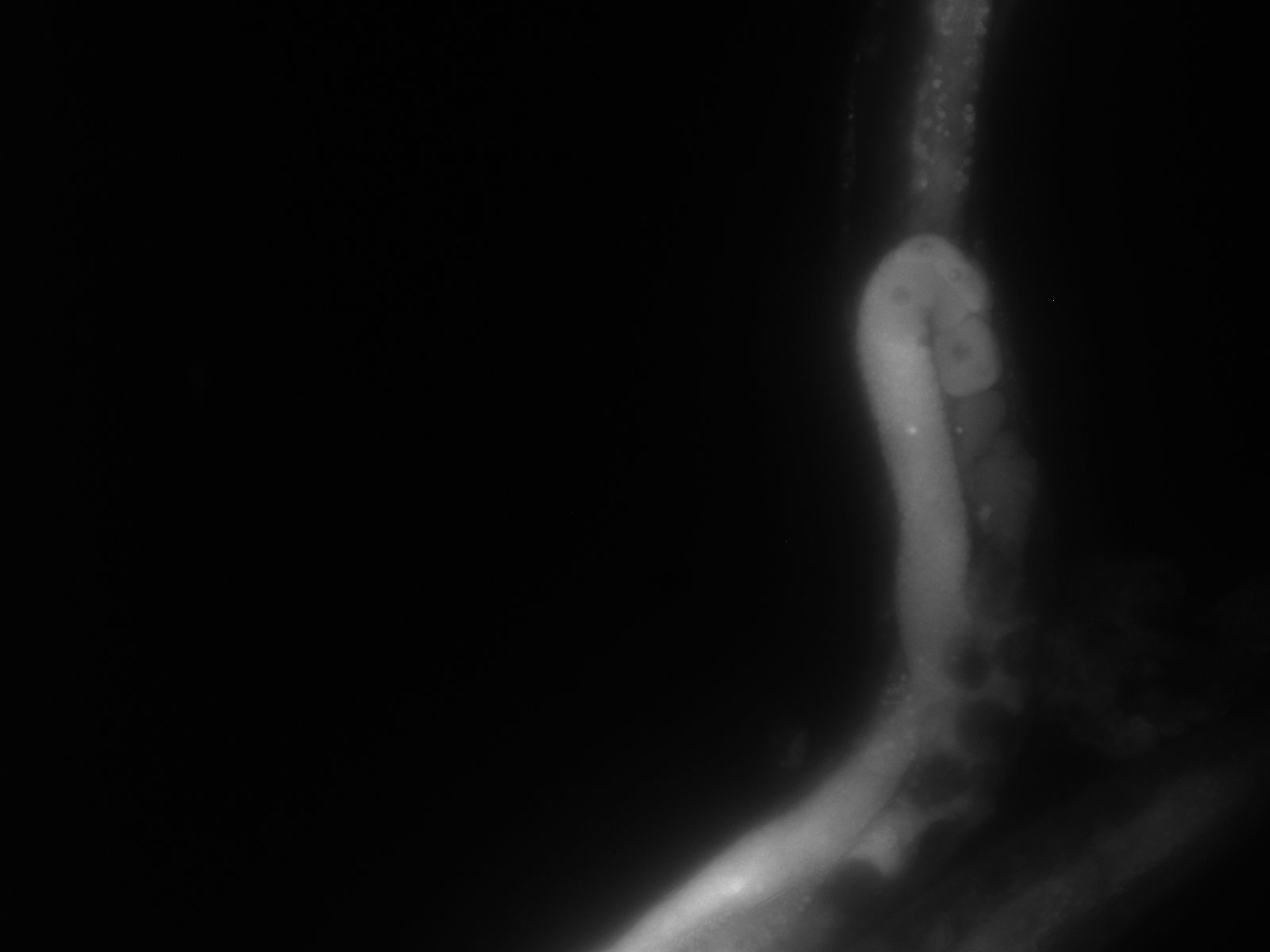

Supplement: S1 File — This file contains all the scoring data of the revised SYTO12 staining experiments. Each of the three biological replicates for Figs 2A, 4D, S2 and S4B–S4C were done in parallel in all strains. Hence, the wild type animals in Fig 2A and in S2 Fig are the same. In most cases animals were scored by live imaging without accompanied image acquisition. Representative images are provided. Consecutive images may image the same gonad. The scoring of apoptotic corpses was performed per gonad, not per image. (ZIP) [file pgen.1011061.s001.zip › SYTO staining experiment united/syto12 staining - 1_rep - 14.5.23 - JPEG/ire-1+pad12132.jpg]

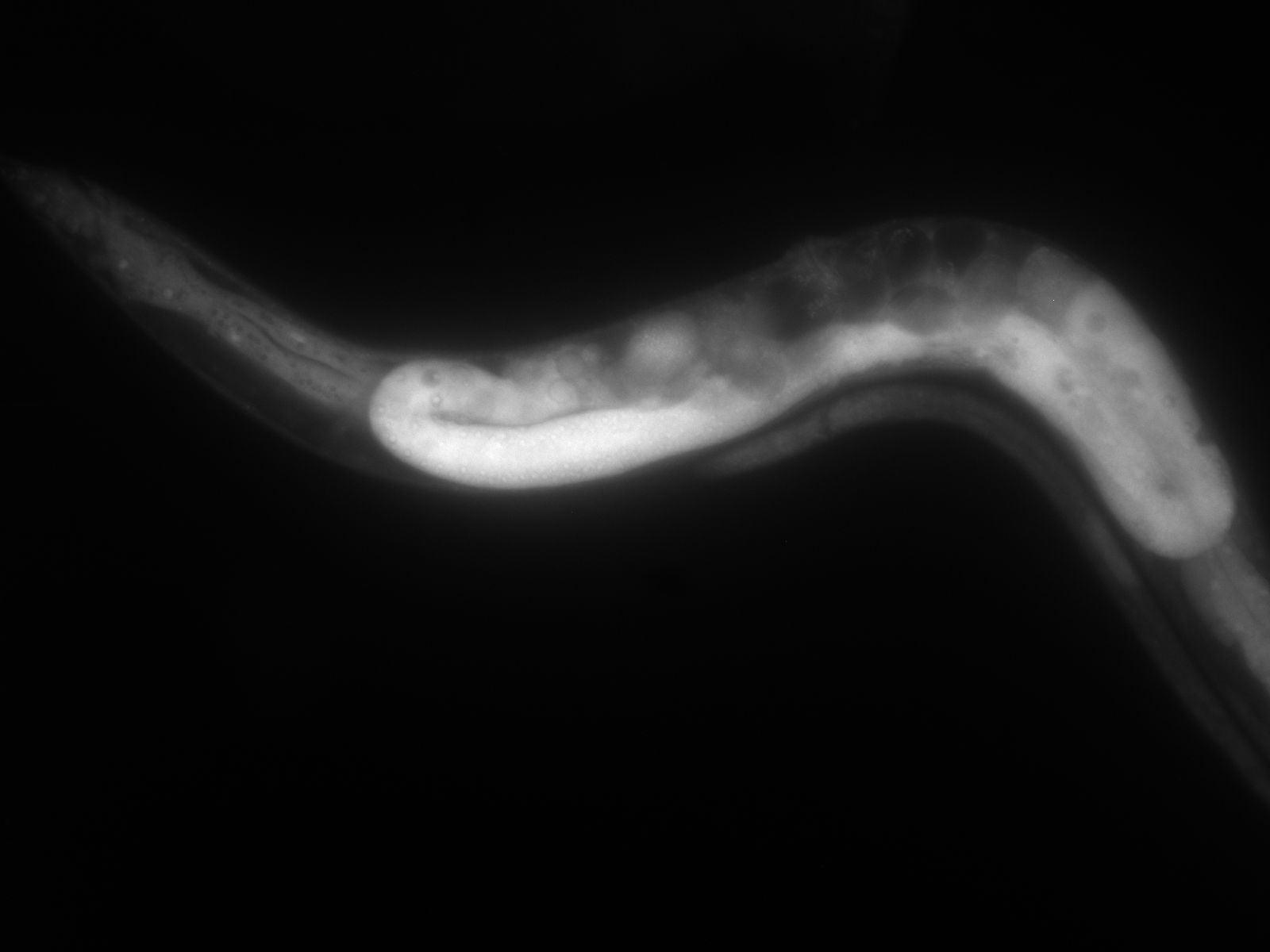

Supplement: S1 File — This file contains all the scoring data of the revised SYTO12 staining experiments. Each of the three biological replicates for Figs 2A, 4D, S2 and S4B–S4C were done in parallel in all strains. Hence, the wild type animals in Fig 2A and in S2 Fig are the same. In most cases animals were scored by live imaging without accompanied image acquisition. Representative images are provided. Consecutive images may image the same gonad. The scoring of apoptotic corpses was performed per gonad, not per image. (ZIP) [file pgen.1011061.s001.zip › SYTO staining experiment united/syto12 staining - 1_rep - 14.5.23 - JPEG/ire-1+tfg1133.jpg]

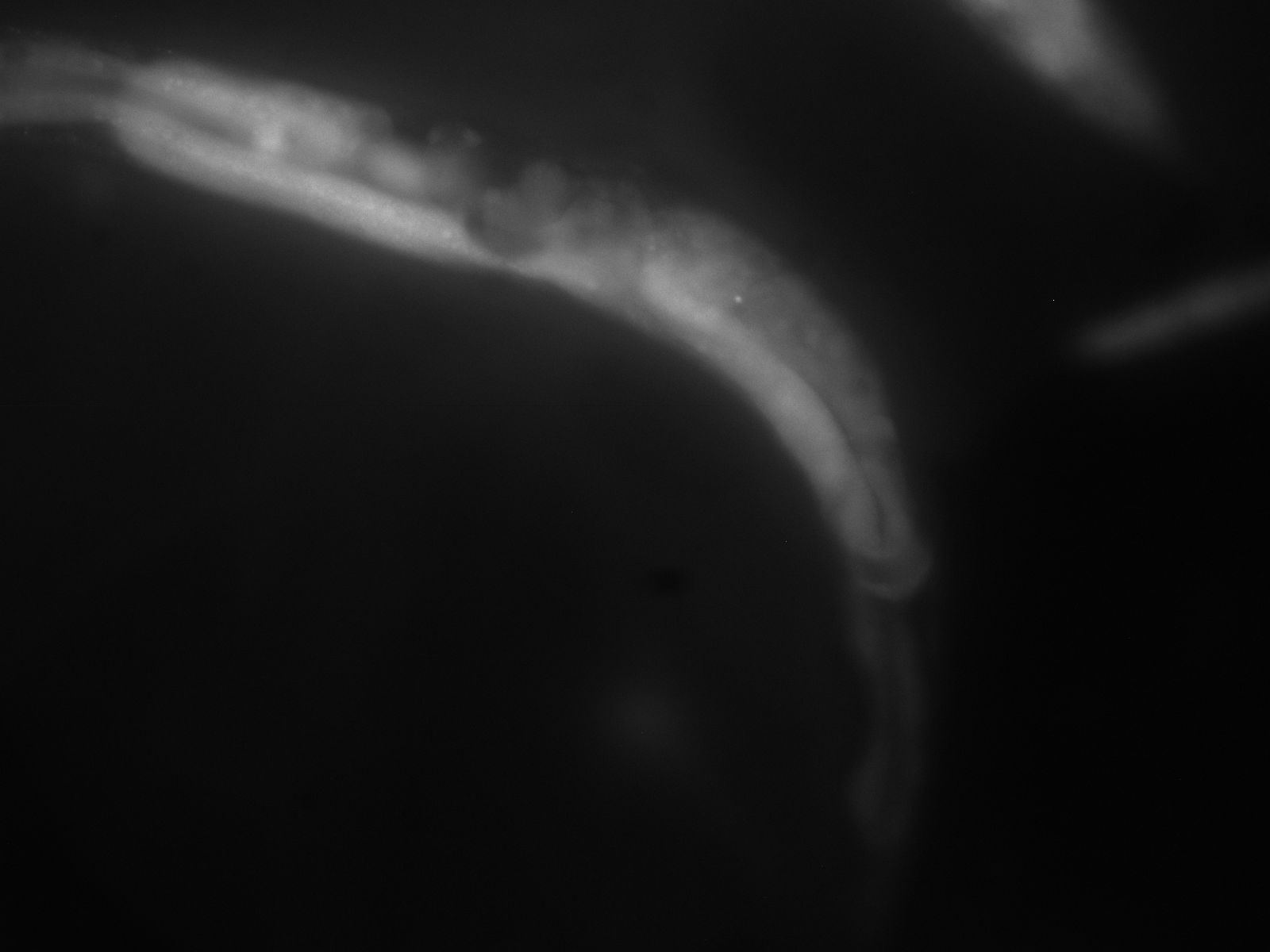

Supplement: S1 File — This file contains all the scoring data of the revised SYTO12 staining experiments. Each of the three biological replicates for Figs 2A, 4D, S2 and S4B–S4C were done in parallel in all strains. Hence, the wild type animals in Fig 2A and in S2 Fig are the same. In most cases animals were scored by live imaging without accompanied image acquisition. Representative images are provided. Consecutive images may image the same gonad. The scoring of apoptotic corpses was performed per gonad, not per image. (ZIP) [file pgen.1011061.s001.zip › SYTO staining experiment united/syto12 staining - 1_rep - 14.5.23 - JPEG/ire-1+tfg1134.jpg]

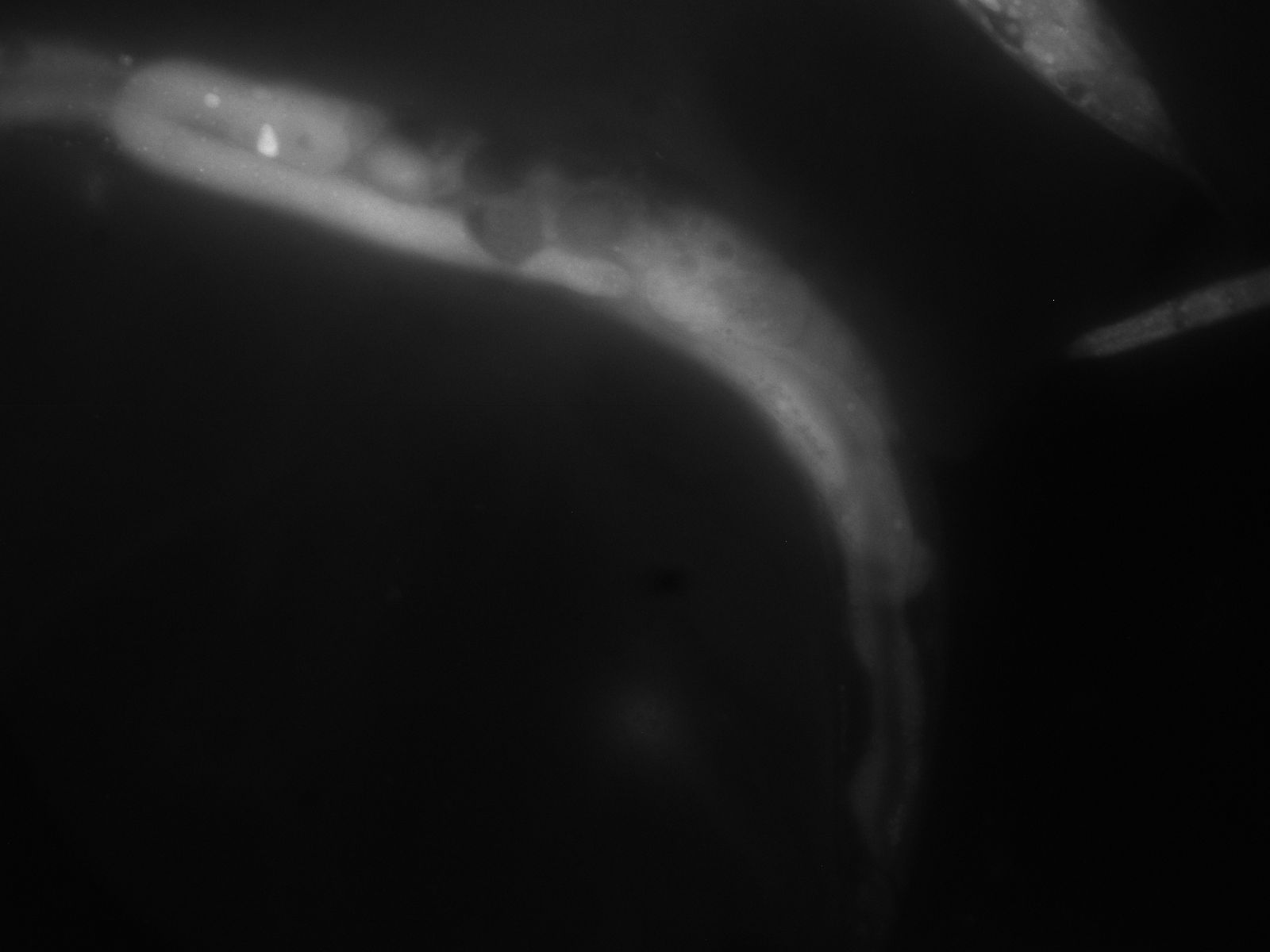

Supplement: S1 File — This file contains all the scoring data of the revised SYTO12 staining experiments. Each of the three biological replicates for Figs 2A, 4D, S2 and S4B–S4C were done in parallel in all strains. Hence, the wild type animals in Fig 2A and in S2 Fig are the same. In most cases animals were scored by live imaging without accompanied image acquisition. Representative images are provided. Consecutive images may image the same gonad. The scoring of apoptotic corpses was performed per gonad, not per image. (ZIP) [file pgen.1011061.s001.zip › SYTO staining experiment united/syto12 staining - 1_rep - 14.5.23 - JPEG/ire-1+tfg1135.jpg]

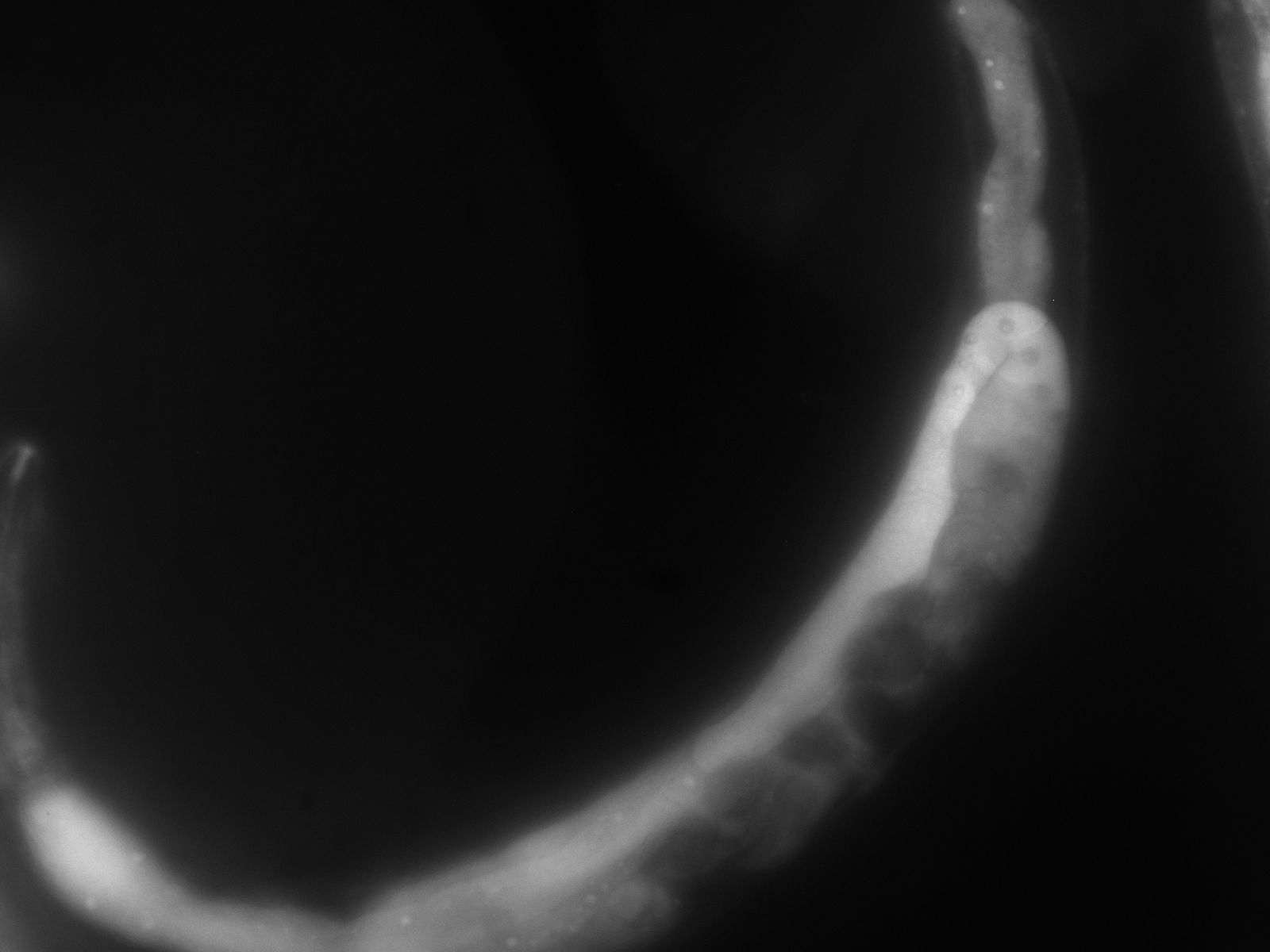

Supplement: S1 File — This file contains all the scoring data of the revised SYTO12 staining experiments. Each of the three biological replicates for Figs 2A, 4D, S2 and S4B–S4C were done in parallel in all strains. Hence, the wild type animals in Fig 2A and in S2 Fig are the same. In most cases animals were scored by live imaging without accompanied image acquisition. Representative images are provided. Consecutive images may image the same gonad. The scoring of apoptotic corpses was performed per gonad, not per image. (ZIP) [file pgen.1011061.s001.zip › SYTO staining experiment united/syto12 staining - 1_rep - 14.5.23 - JPEG/ire-1+tfg1136.jpg]

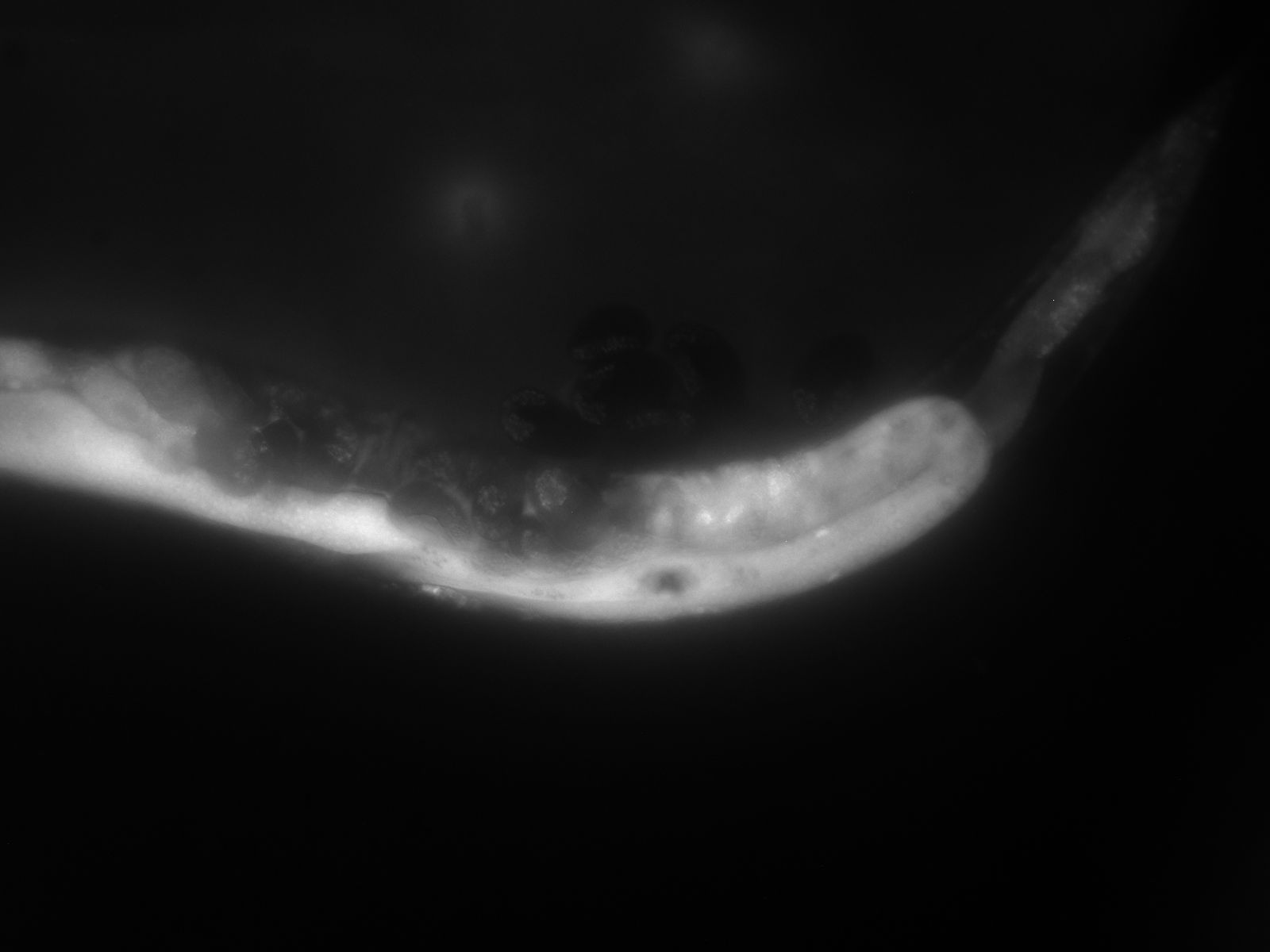

Supplement: S1 File — This file contains all the scoring data of the revised SYTO12 staining experiments. Each of the three biological replicates for Figs 2A, 4D, S2 and S4B–S4C were done in parallel in all strains. Hence, the wild type animals in Fig 2A and in S2 Fig are the same. In most cases animals were scored by live imaging without accompanied image acquisition. Representative images are provided. Consecutive images may image the same gonad. The scoring of apoptotic corpses was performed per gonad, not per image. (ZIP) [file pgen.1011061.s001.zip › SYTO staining experiment united/syto12 staining - 1_rep - 14.5.23 - JPEG/ire-1+tfg1137.jpg]

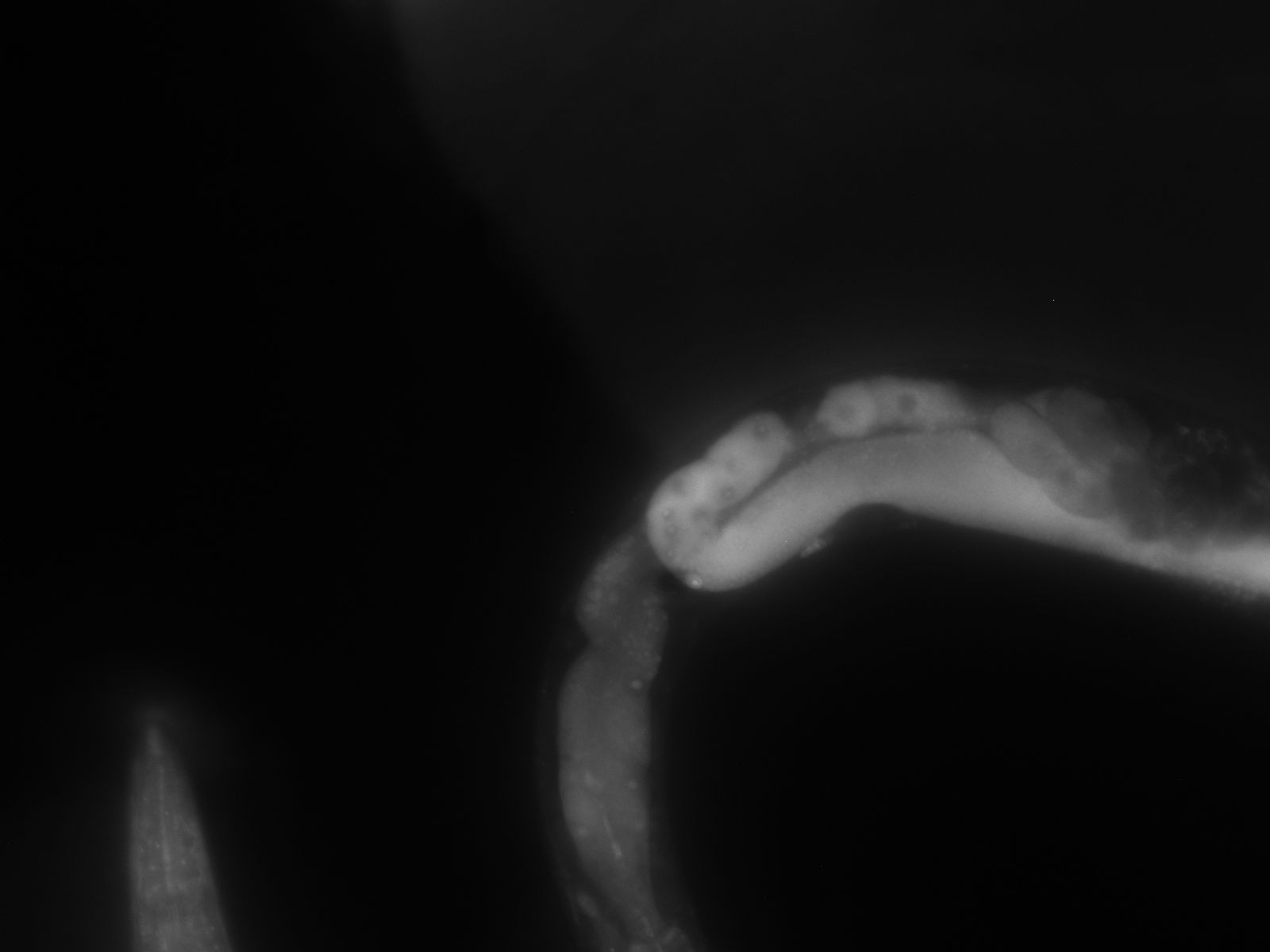

Supplement: S1 File — This file contains all the scoring data of the revised SYTO12 staining experiments. Each of the three biological replicates for Figs 2A, 4D, S2 and S4B–S4C were done in parallel in all strains. Hence, the wild type animals in Fig 2A and in S2 Fig are the same. In most cases animals were scored by live imaging without accompanied image acquisition. Representative images are provided. Consecutive images may image the same gonad. The scoring of apoptotic corpses was performed per gonad, not per image. (ZIP) [file pgen.1011061.s001.zip › SYTO staining experiment united/syto12 staining - 1_rep - 14.5.23 - JPEG/ire-1+tfg1138.jpg]

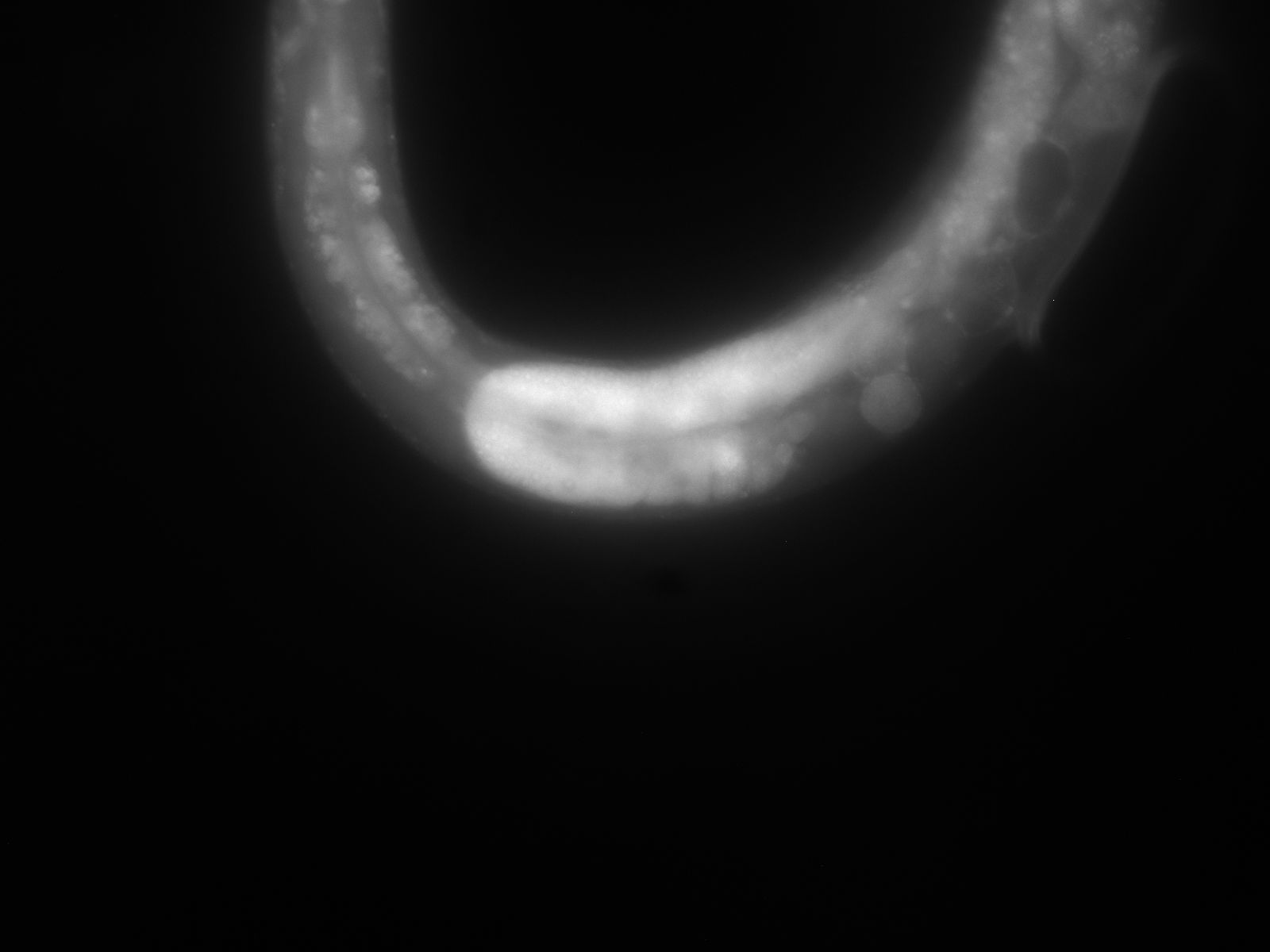

Supplement: S1 File — This file contains all the scoring data of the revised SYTO12 staining experiments. Each of the three biological replicates for Figs 2A, 4D, S2 and S4B–S4C were done in parallel in all strains. Hence, the wild type animals in Fig 2A and in S2 Fig are the same. In most cases animals were scored by live imaging without accompanied image acquisition. Representative images are provided. Consecutive images may image the same gonad. The scoring of apoptotic corpses was performed per gonad, not per image. (ZIP) [file pgen.1011061.s001.zip › SYTO staining experiment united/syto12 staining - 1_rep - 14.5.23 - JPEG/ire-1+tfg1139.jpg]

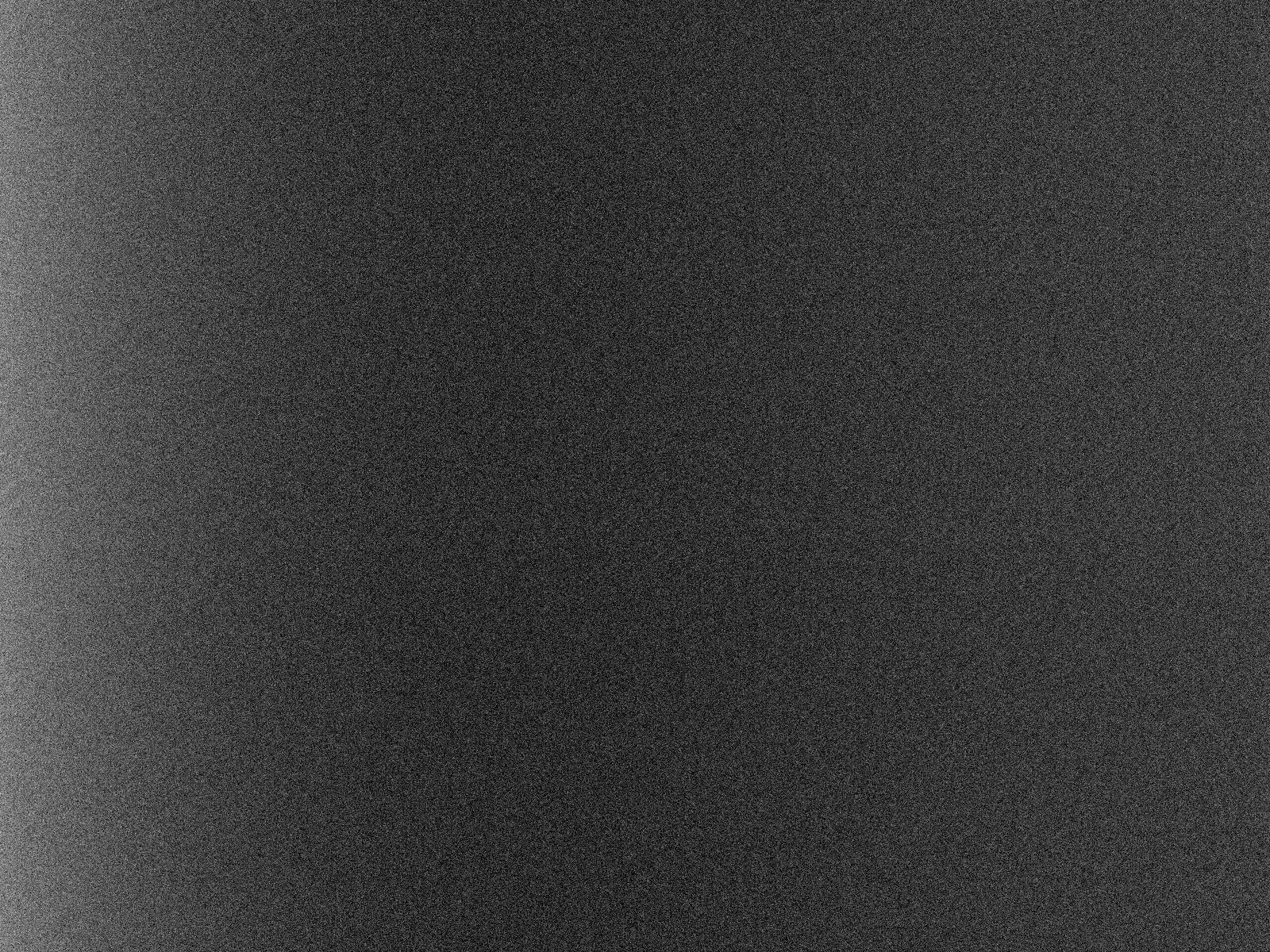

Supplement: S1 File — This file contains all the scoring data of the revised SYTO12 staining experiments. Each of the three biological replicates for Figs 2A, 4D, S2 and S4B–S4C were done in parallel in all strains. Hence, the wild type animals in Fig 2A and in S2 Fig are the same. In most cases animals were scored by live imaging without accompanied image acquisition. Representative images are provided. Consecutive images may image the same gonad. The scoring of apoptotic corpses was performed per gonad, not per image. (ZIP) [file pgen.1011061.s001.zip › SYTO staining experiment united/syto12 staining - 1_rep - 14.5.23 - JPEG/ire-1+tfg1140.jpg]

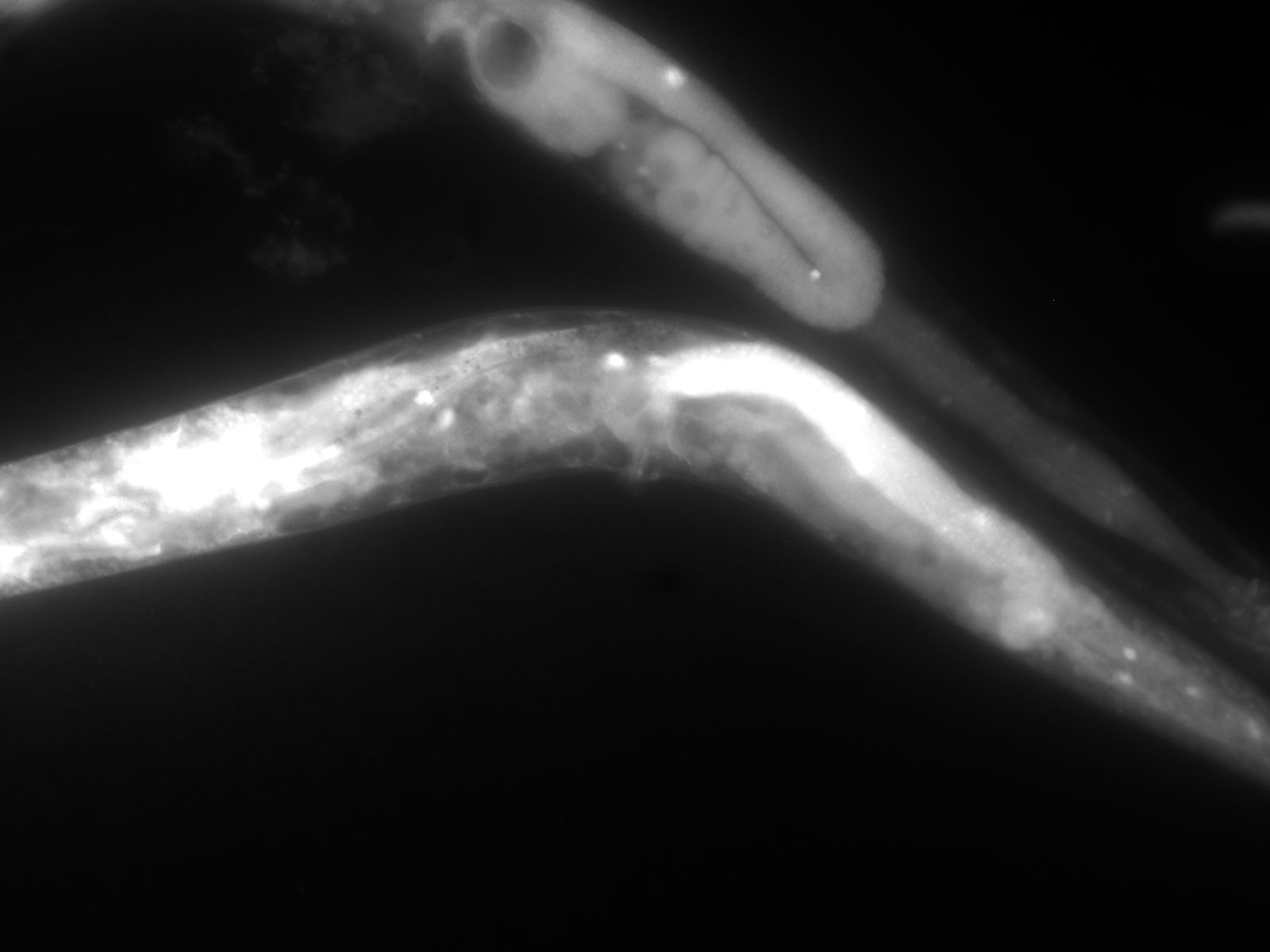

Supplement: S1 File — This file contains all the scoring data of the revised SYTO12 staining experiments. Each of the three biological replicates for Figs 2A, 4D, S2 and S4B–S4C were done in parallel in all strains. Hence, the wild type animals in Fig 2A and in S2 Fig are the same. In most cases animals were scored by live imaging without accompanied image acquisition. Representative images are provided. Consecutive images may image the same gonad. The scoring of apoptotic corpses was performed per gonad, not per image. (ZIP) [file pgen.1011061.s001.zip › SYTO staining experiment united/syto12 staining - 1_rep - 14.5.23 - JPEG/ire-1+tfg1141.jpg]

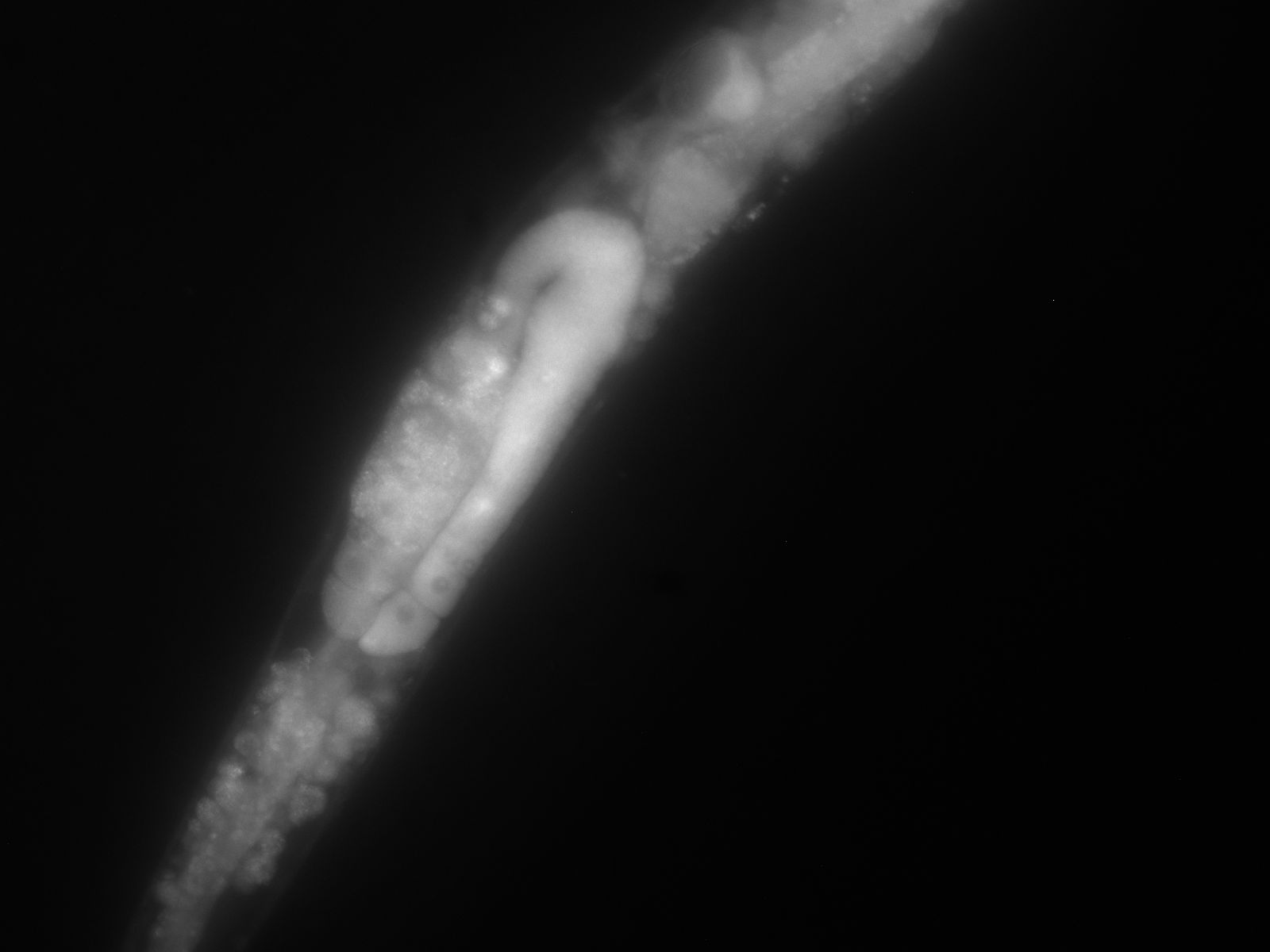

Supplement: S1 File — This file contains all the scoring data of the revised SYTO12 staining experiments. Each of the three biological replicates for Figs 2A, 4D, S2 and S4B–S4C were done in parallel in all strains. Hence, the wild type animals in Fig 2A and in S2 Fig are the same. In most cases animals were scored by live imaging without accompanied image acquisition. Representative images are provided. Consecutive images may image the same gonad. The scoring of apoptotic corpses was performed per gonad, not per image. (ZIP) [file pgen.1011061.s001.zip › SYTO staining experiment united/syto12 staining - 1_rep - 14.5.23 - JPEG/ire-1+tfg1142.jpg]

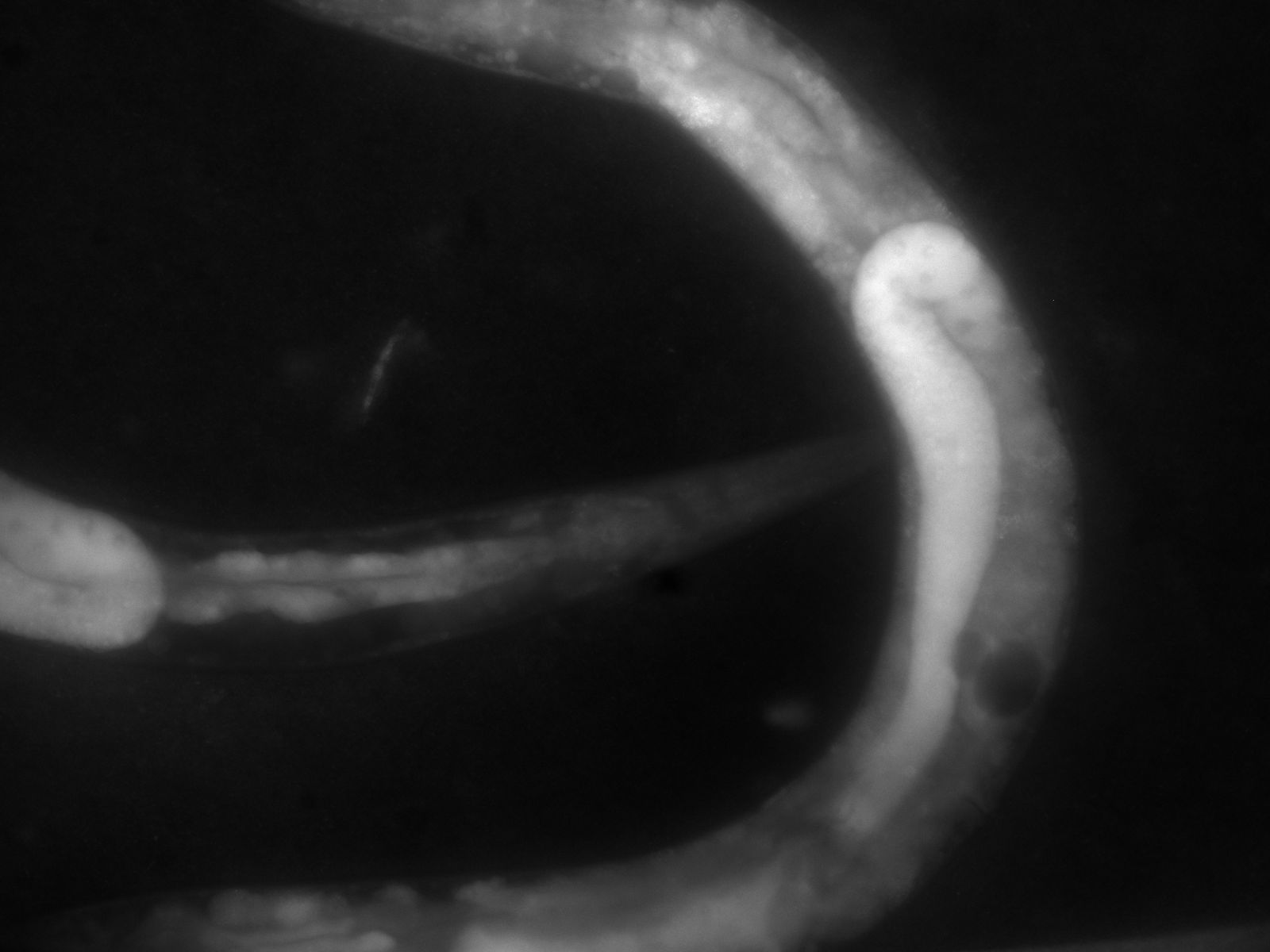

Supplement: S1 File — This file contains all the scoring data of the revised SYTO12 staining experiments. Each of the three biological replicates for Figs 2A, 4D, S2 and S4B–S4C were done in parallel in all strains. Hence, the wild type animals in Fig 2A and in S2 Fig are the same. In most cases animals were scored by live imaging without accompanied image acquisition. Representative images are provided. Consecutive images may image the same gonad. The scoring of apoptotic corpses was performed per gonad, not per image. (ZIP) [file pgen.1011061.s001.zip › SYTO staining experiment united/syto12 staining - 1_rep - 14.5.23 - JPEG/ire-1+tfg1143.jpg]

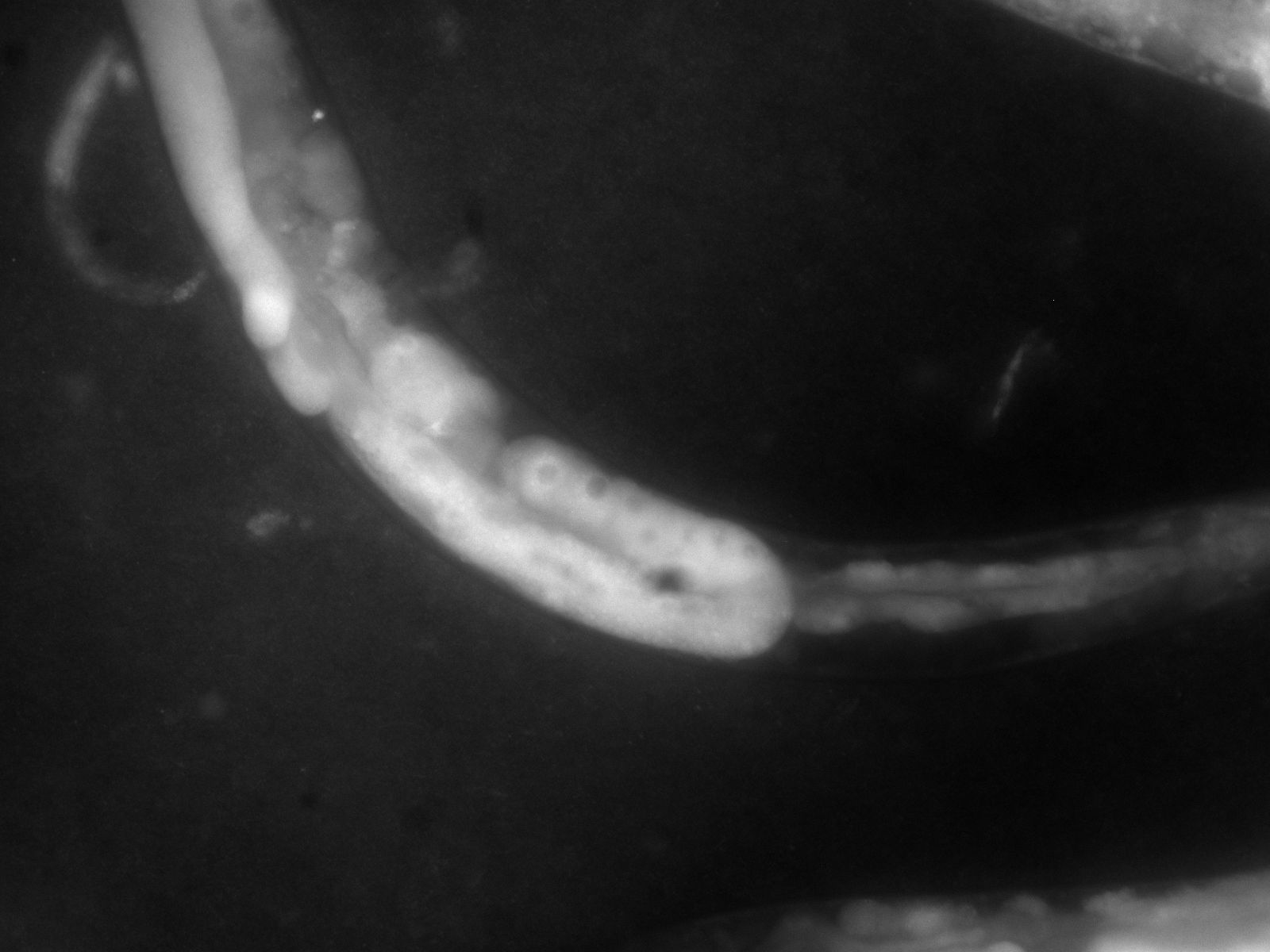

Supplement: S1 File — This file contains all the scoring data of the revised SYTO12 staining experiments. Each of the three biological replicates for Figs 2A, 4D, S2 and S4B–S4C were done in parallel in all strains. Hence, the wild type animals in Fig 2A and in S2 Fig are the same. In most cases animals were scored by live imaging without accompanied image acquisition. Representative images are provided. Consecutive images may image the same gonad. The scoring of apoptotic corpses was performed per gonad, not per image. (ZIP) [file pgen.1011061.s001.zip › SYTO staining experiment united/syto12 staining - 1_rep - 14.5.23 - JPEG/ire-1+tfg1144.jpg]

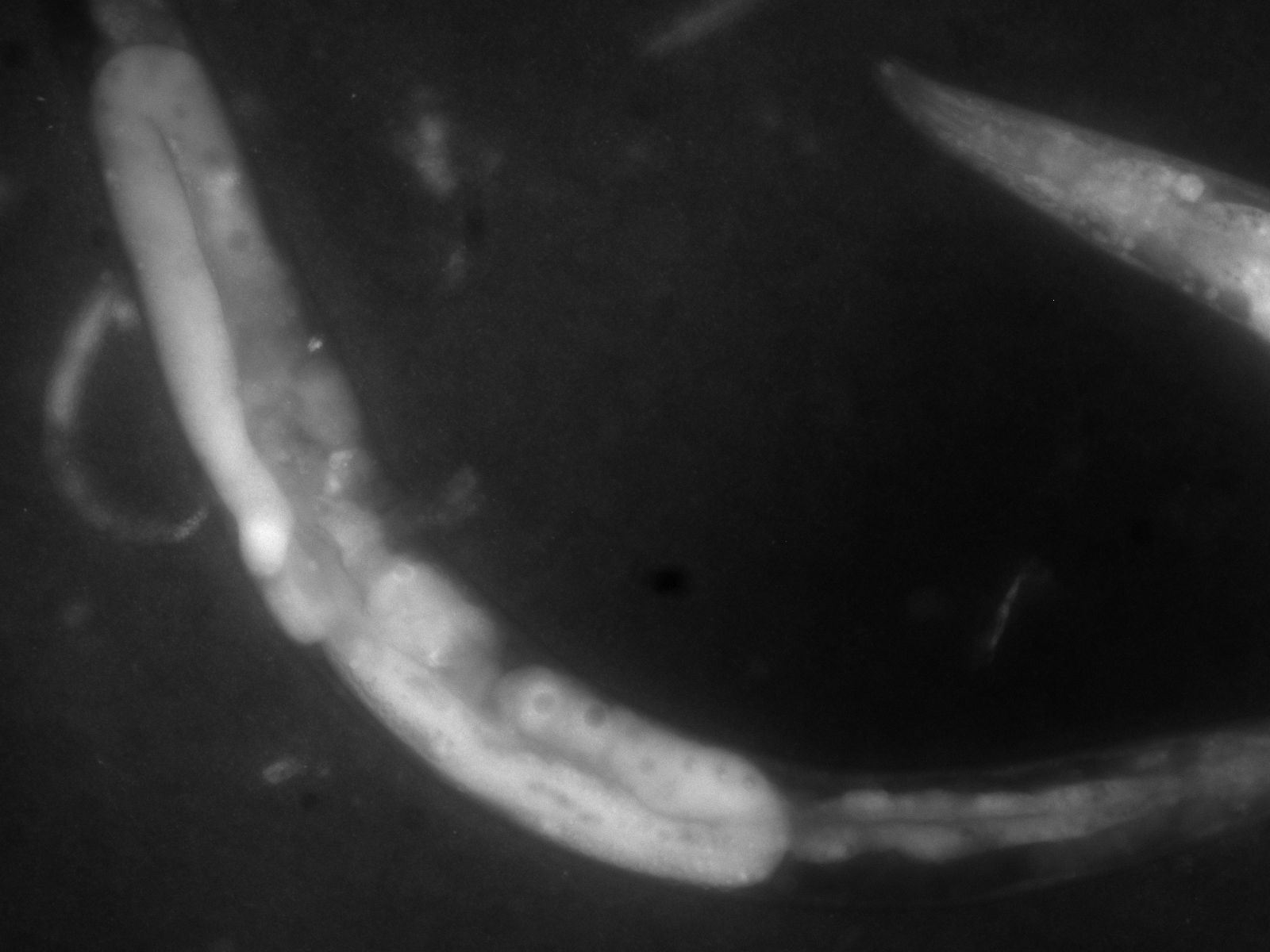

Supplement: S1 File — This file contains all the scoring data of the revised SYTO12 staining experiments. Each of the three biological replicates for Figs 2A, 4D, S2 and S4B–S4C were done in parallel in all strains. Hence, the wild type animals in Fig 2A and in S2 Fig are the same. In most cases animals were scored by live imaging without accompanied image acquisition. Representative images are provided. Consecutive images may image the same gonad. The scoring of apoptotic corpses was performed per gonad, not per image. (ZIP) [file pgen.1011061.s001.zip › SYTO staining experiment united/syto12 staining - 1_rep - 14.5.23 - JPEG/ire-1+tfg1145.jpg]

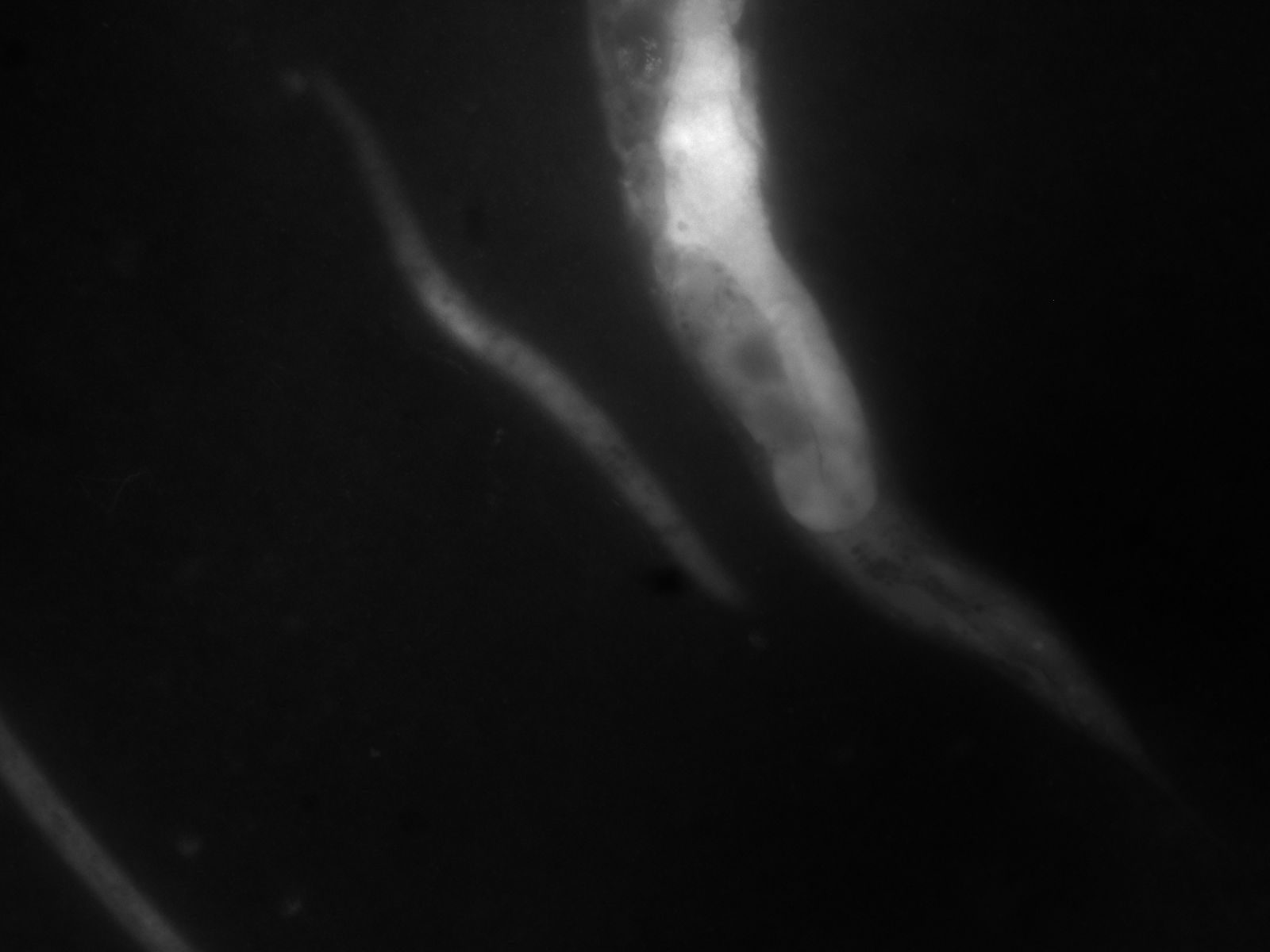

Supplement: S1 File — This file contains all the scoring data of the revised SYTO12 staining experiments. Each of the three biological replicates for Figs 2A, 4D, S2 and S4B–S4C were done in parallel in all strains. Hence, the wild type animals in Fig 2A and in S2 Fig are the same. In most cases animals were scored by live imaging without accompanied image acquisition. Representative images are provided. Consecutive images may image the same gonad. The scoring of apoptotic corpses was performed per gonad, not per image. (ZIP) [file pgen.1011061.s001.zip › SYTO staining experiment united/syto12 staining - 1_rep - 14.5.23 - JPEG/ire-1+tfg1146.jpg]

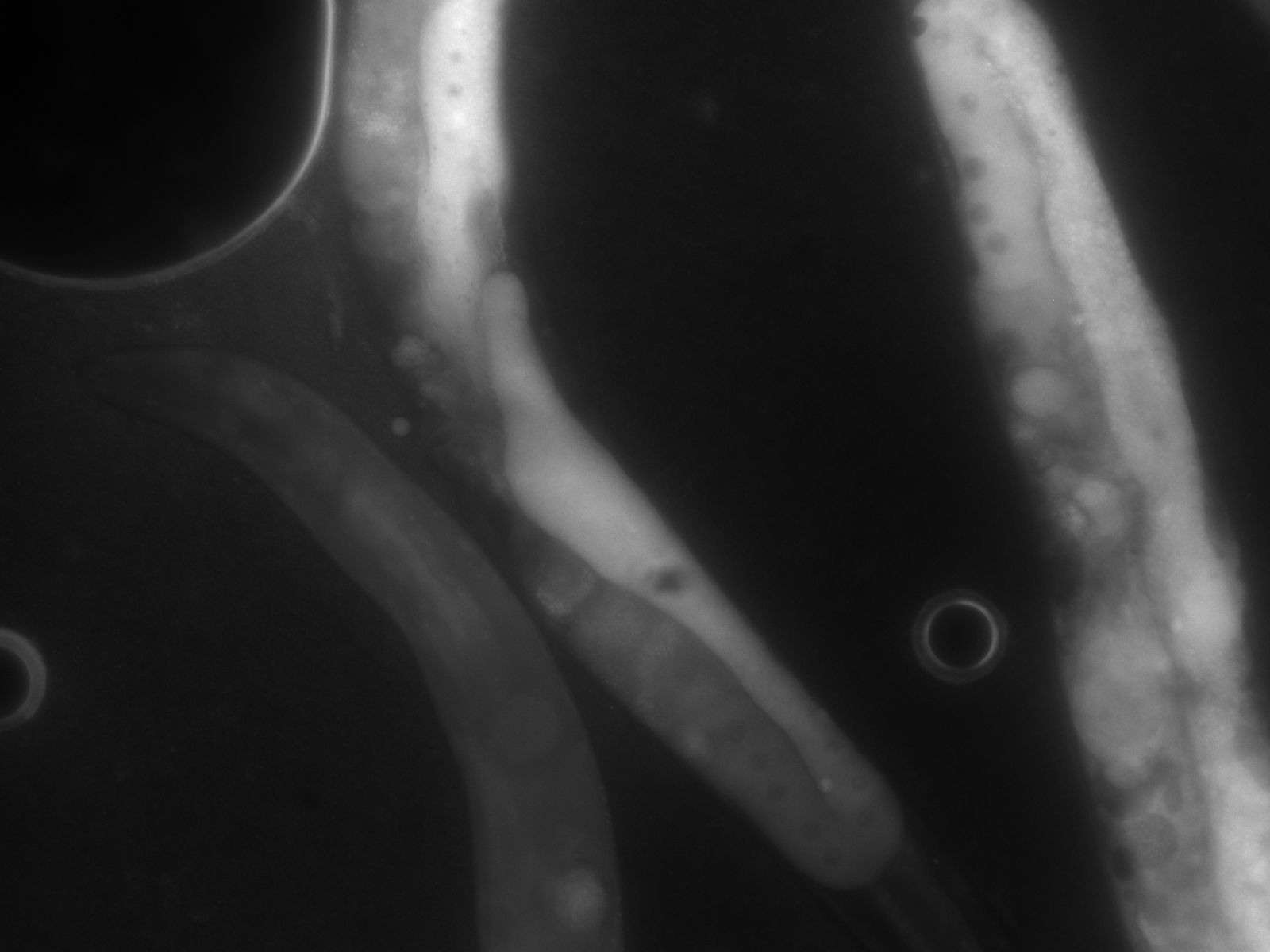

Supplement: S1 File — This file contains all the scoring data of the revised SYTO12 staining experiments. Each of the three biological replicates for Figs 2A, 4D, S2 and S4B–S4C were done in parallel in all strains. Hence, the wild type animals in Fig 2A and in S2 Fig are the same. In most cases animals were scored by live imaging without accompanied image acquisition. Representative images are provided. Consecutive images may image the same gonad. The scoring of apoptotic corpses was performed per gonad, not per image. (ZIP) [file pgen.1011061.s001.zip › SYTO staining experiment united/syto12 staining - 1_rep - 14.5.23 - JPEG/ire-1+tfg1147.jpg]

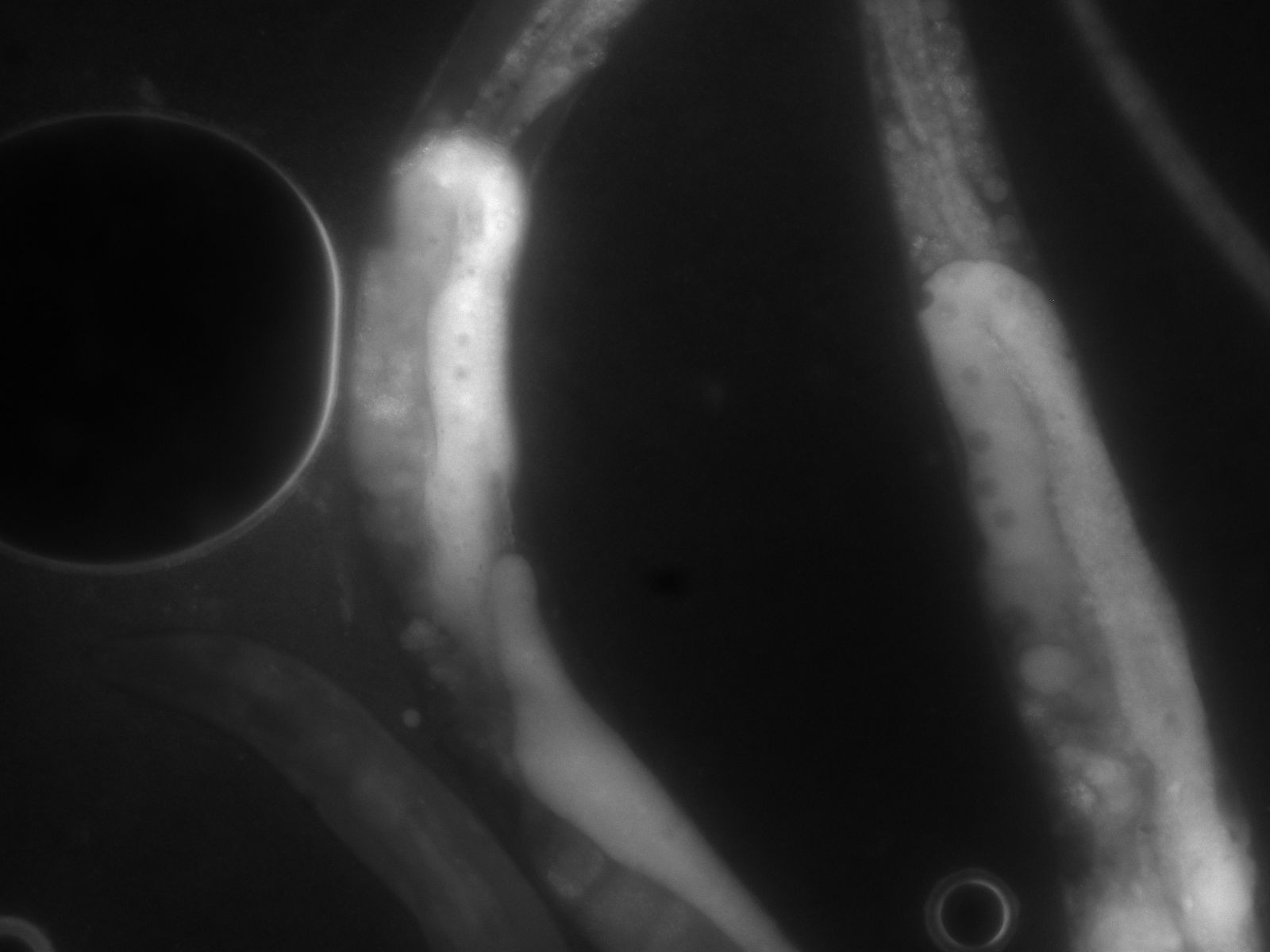

Supplement: S1 File — This file contains all the scoring data of the revised SYTO12 staining experiments. Each of the three biological replicates for Figs 2A, 4D, S2 and S4B–S4C were done in parallel in all strains. Hence, the wild type animals in Fig 2A and in S2 Fig are the same. In most cases animals were scored by live imaging without accompanied image acquisition. Representative images are provided. Consecutive images may image the same gonad. The scoring of apoptotic corpses was performed per gonad, not per image. (ZIP) [file pgen.1011061.s001.zip › SYTO staining experiment united/syto12 staining - 1_rep - 14.5.23 - JPEG/ire-1+tfg1148.jpg]

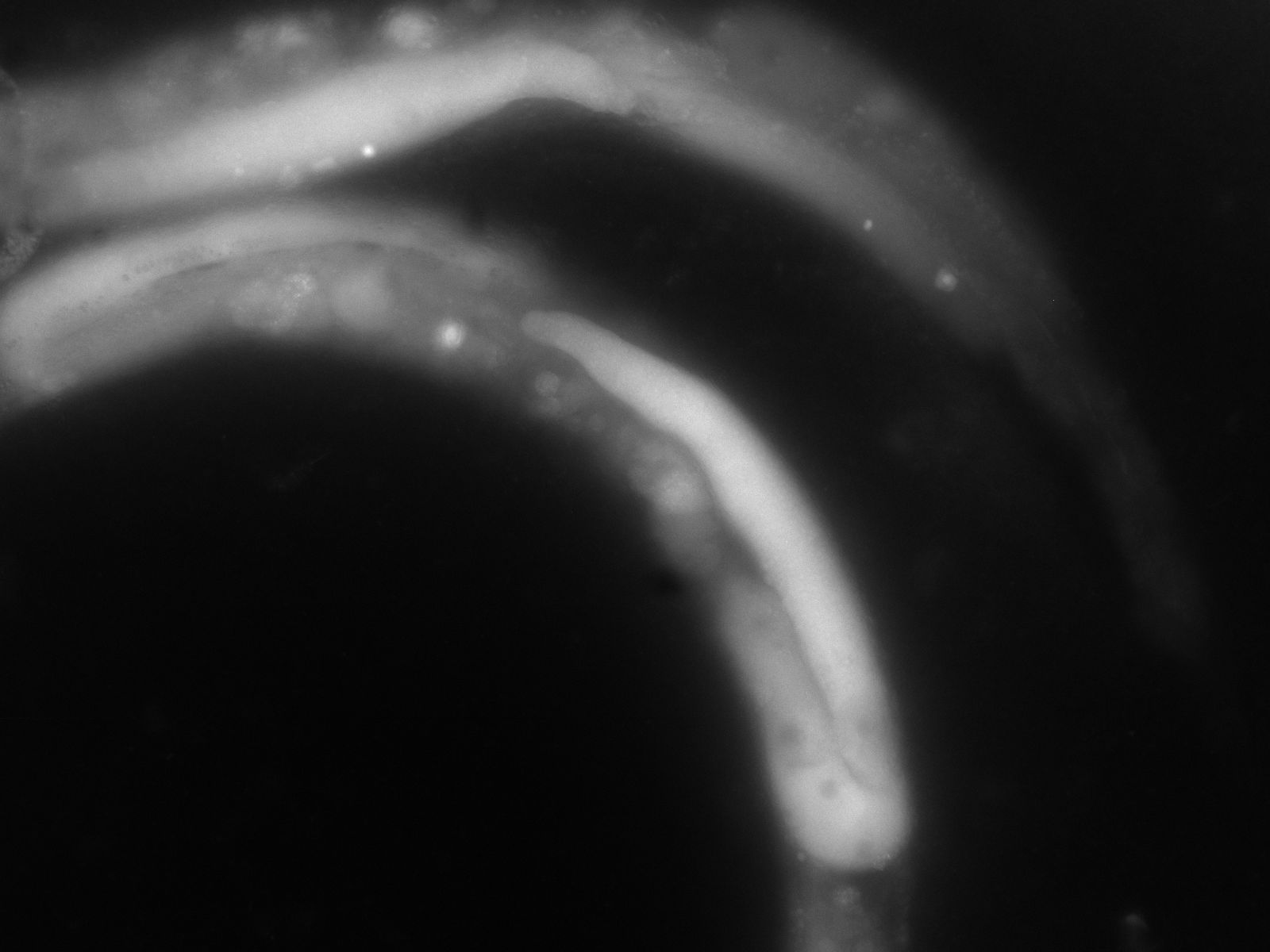

Supplement: S1 File — This file contains all the scoring data of the revised SYTO12 staining experiments. Each of the three biological replicates for Figs 2A, 4D, S2 and S4B–S4C were done in parallel in all strains. Hence, the wild type animals in Fig 2A and in S2 Fig are the same. In most cases animals were scored by live imaging without accompanied image acquisition. Representative images are provided. Consecutive images may image the same gonad. The scoring of apoptotic corpses was performed per gonad, not per image. (ZIP) [file pgen.1011061.s001.zip › SYTO staining experiment united/syto12 staining - 1_rep - 14.5.23 - JPEG/ire-1+tfg1149.jpg]

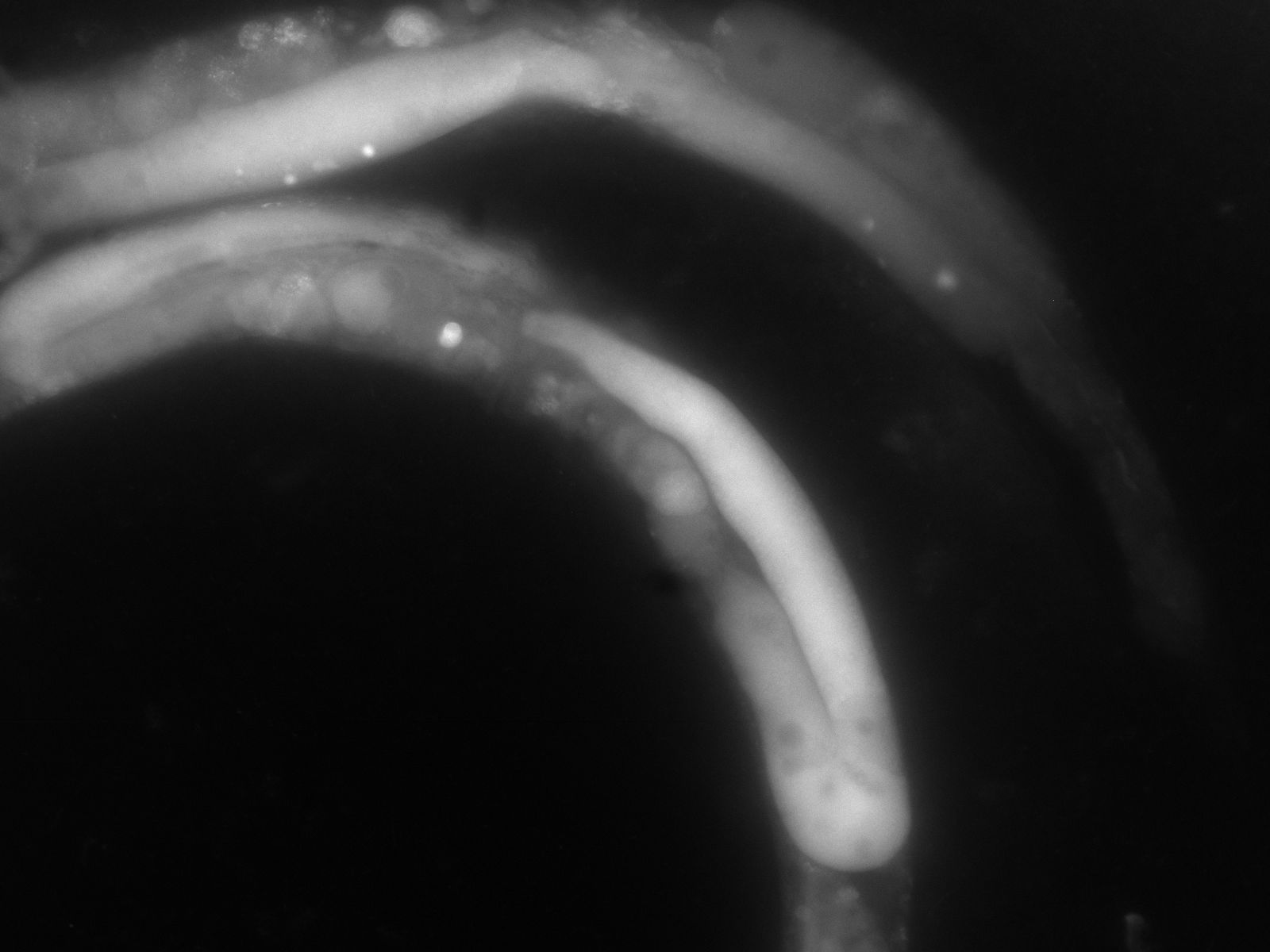

Supplement: S1 File — This file contains all the scoring data of the revised SYTO12 staining experiments. Each of the three biological replicates for Figs 2A, 4D, S2 and S4B–S4C were done in parallel in all strains. Hence, the wild type animals in Fig 2A and in S2 Fig are the same. In most cases animals were scored by live imaging without accompanied image acquisition. Representative images are provided. Consecutive images may image the same gonad. The scoring of apoptotic corpses was performed per gonad, not per image. (ZIP) [file pgen.1011061.s001.zip › SYTO staining experiment united/syto12 staining - 1_rep - 14.5.23 - JPEG/ire-1+tfg1150.jpg]

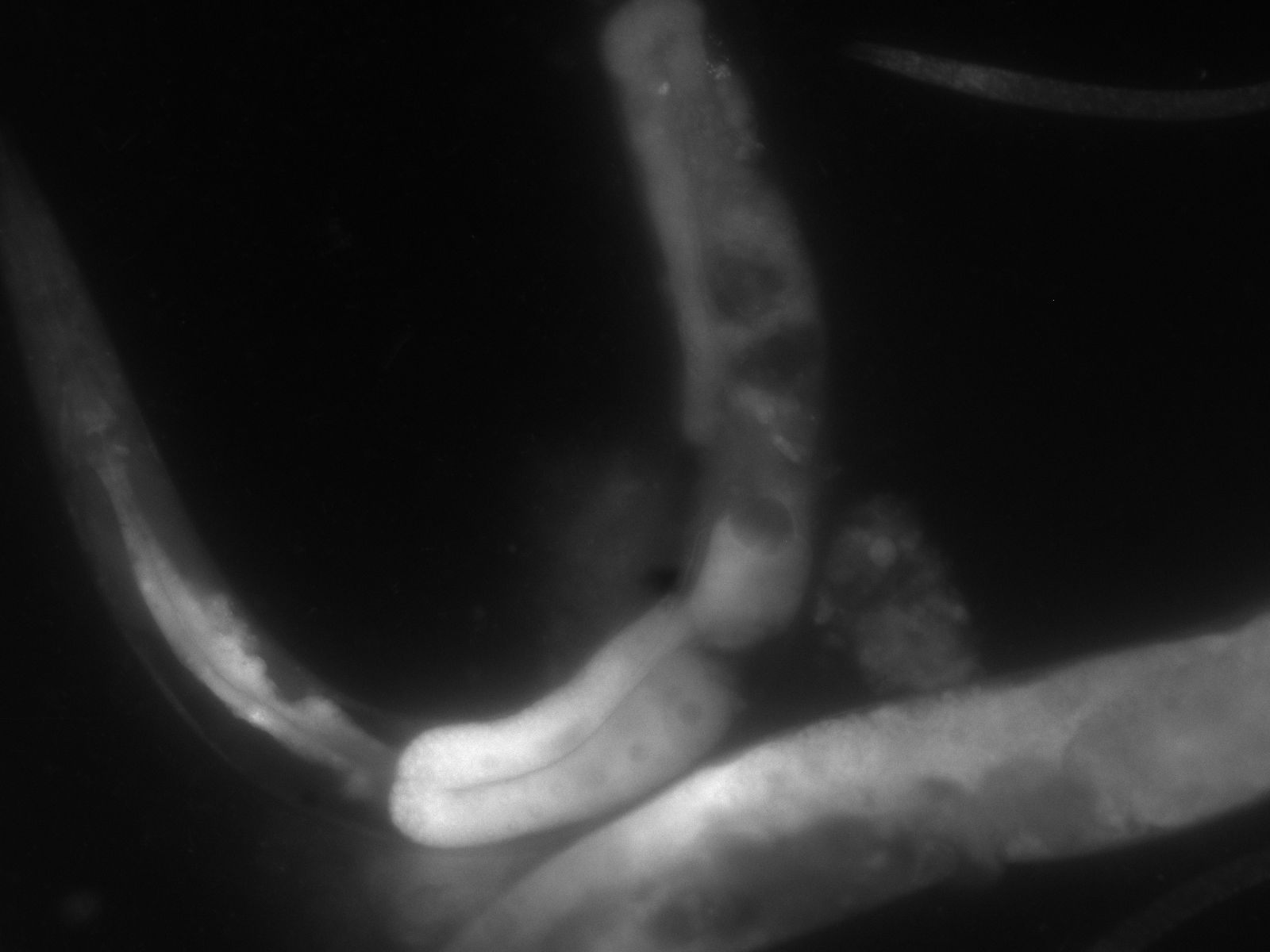

Supplement: S1 File — This file contains all the scoring data of the revised SYTO12 staining experiments. Each of the three biological replicates for Figs 2A, 4D, S2 and S4B–S4C were done in parallel in all strains. Hence, the wild type animals in Fig 2A and in S2 Fig are the same. In most cases animals were scored by live imaging without accompanied image acquisition. Representative images are provided. Consecutive images may image the same gonad. The scoring of apoptotic corpses was performed per gonad, not per image. (ZIP) [file pgen.1011061.s001.zip › SYTO staining experiment united/syto12 staining - 1_rep - 14.5.23 - JPEG/ire-1+tfg1151.jpg]

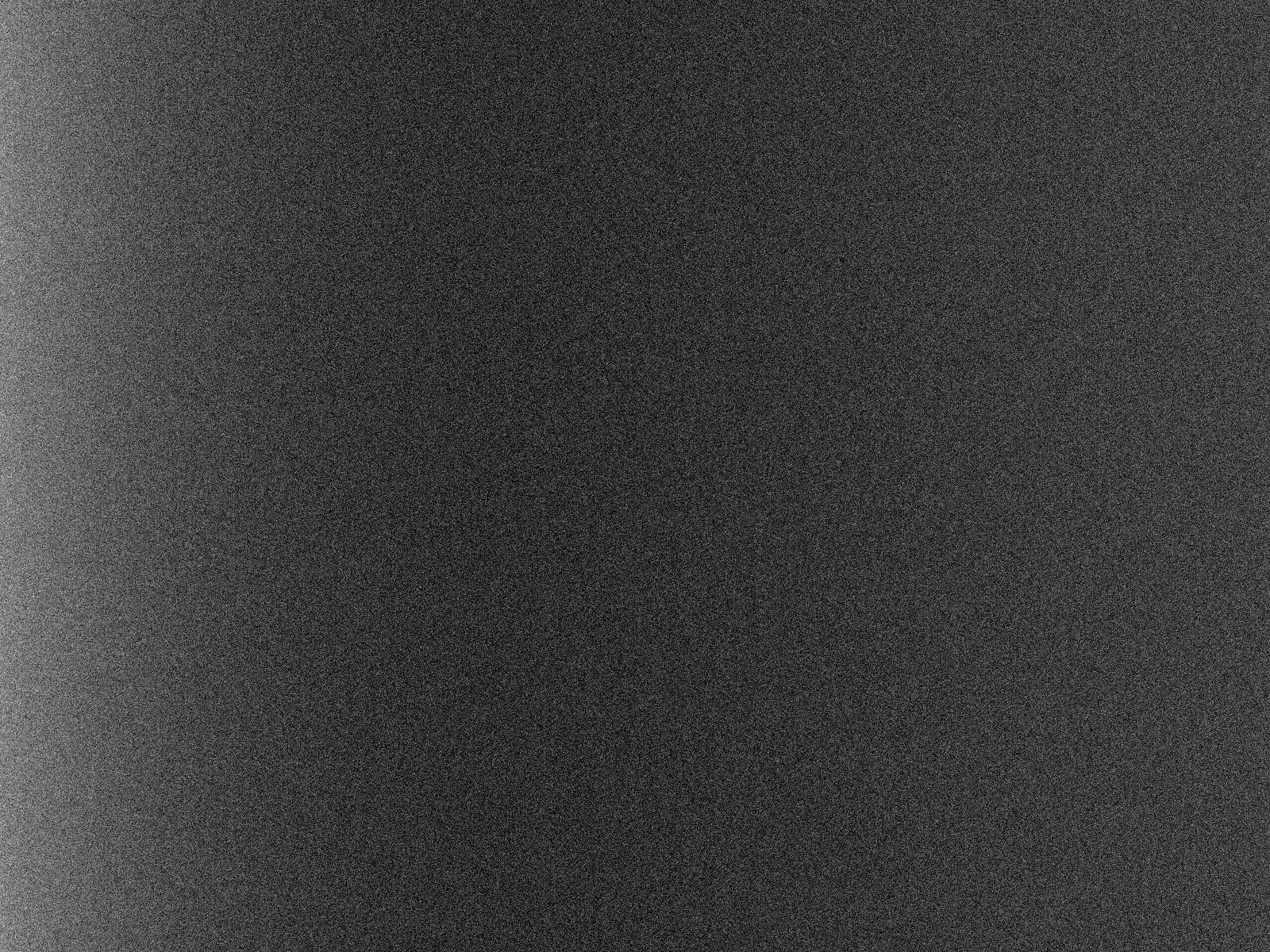

Supplement: S1 File — This file contains all the scoring data of the revised SYTO12 staining experiments. Each of the three biological replicates for Figs 2A, 4D, S2 and S4B–S4C were done in parallel in all strains. Hence, the wild type animals in Fig 2A and in S2 Fig are the same. In most cases animals were scored by live imaging without accompanied image acquisition. Representative images are provided. Consecutive images may image the same gonad. The scoring of apoptotic corpses was performed per gonad, not per image. (ZIP) [file pgen.1011061.s001.zip › SYTO staining experiment united/syto12 staining - 1_rep - 14.5.23 - JPEG/ire-1+tfg1152.jpg]

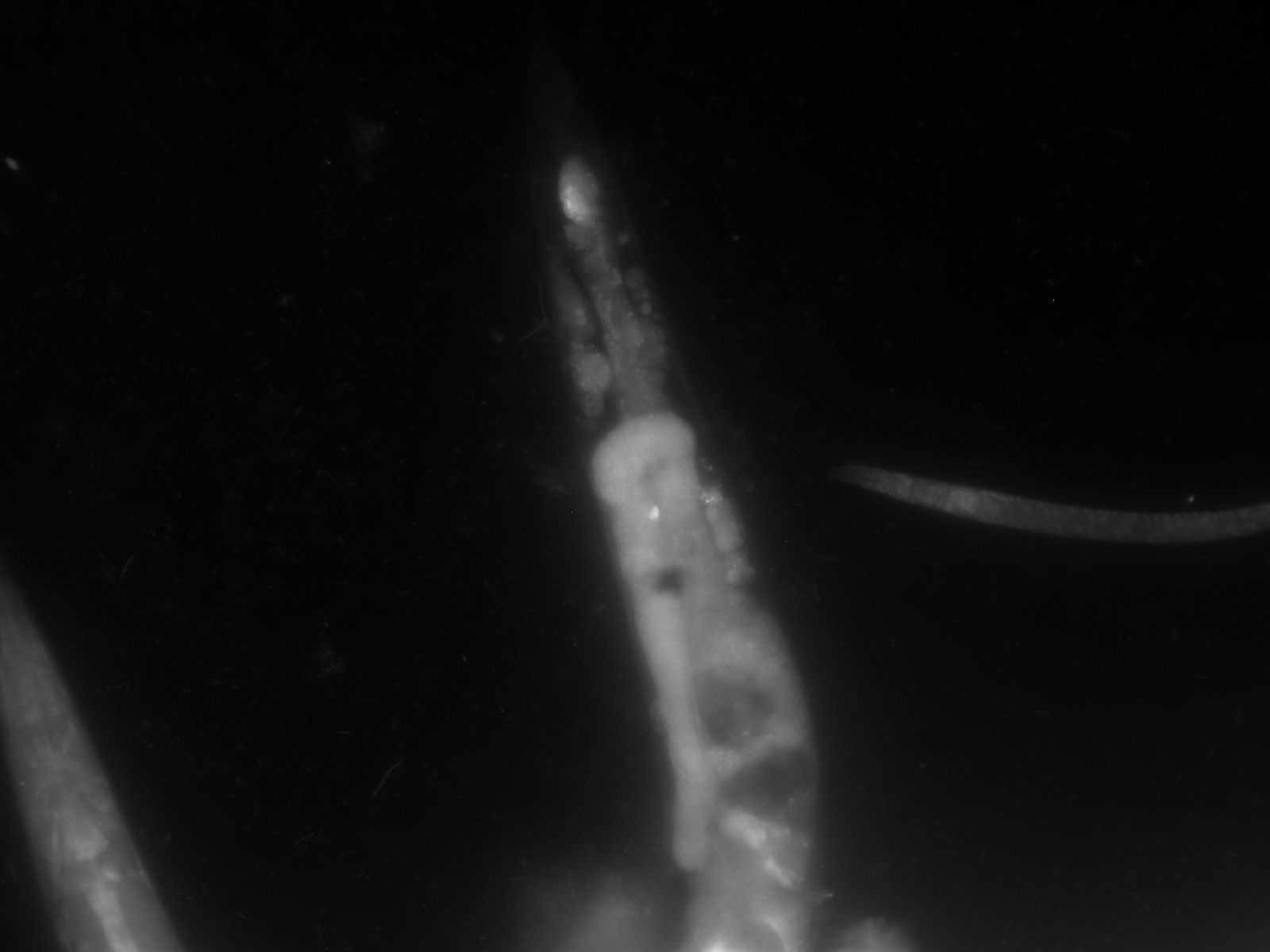

Supplement: S1 File — This file contains all the scoring data of the revised SYTO12 staining experiments. Each of the three biological replicates for Figs 2A, 4D, S2 and S4B–S4C were done in parallel in all strains. Hence, the wild type animals in Fig 2A and in S2 Fig are the same. In most cases animals were scored by live imaging without accompanied image acquisition. Representative images are provided. Consecutive images may image the same gonad. The scoring of apoptotic corpses was performed per gonad, not per image. (ZIP) [file pgen.1011061.s001.zip › SYTO staining experiment united/syto12 staining - 1_rep - 14.5.23 - JPEG/ire-1+tfg1153.jpg]

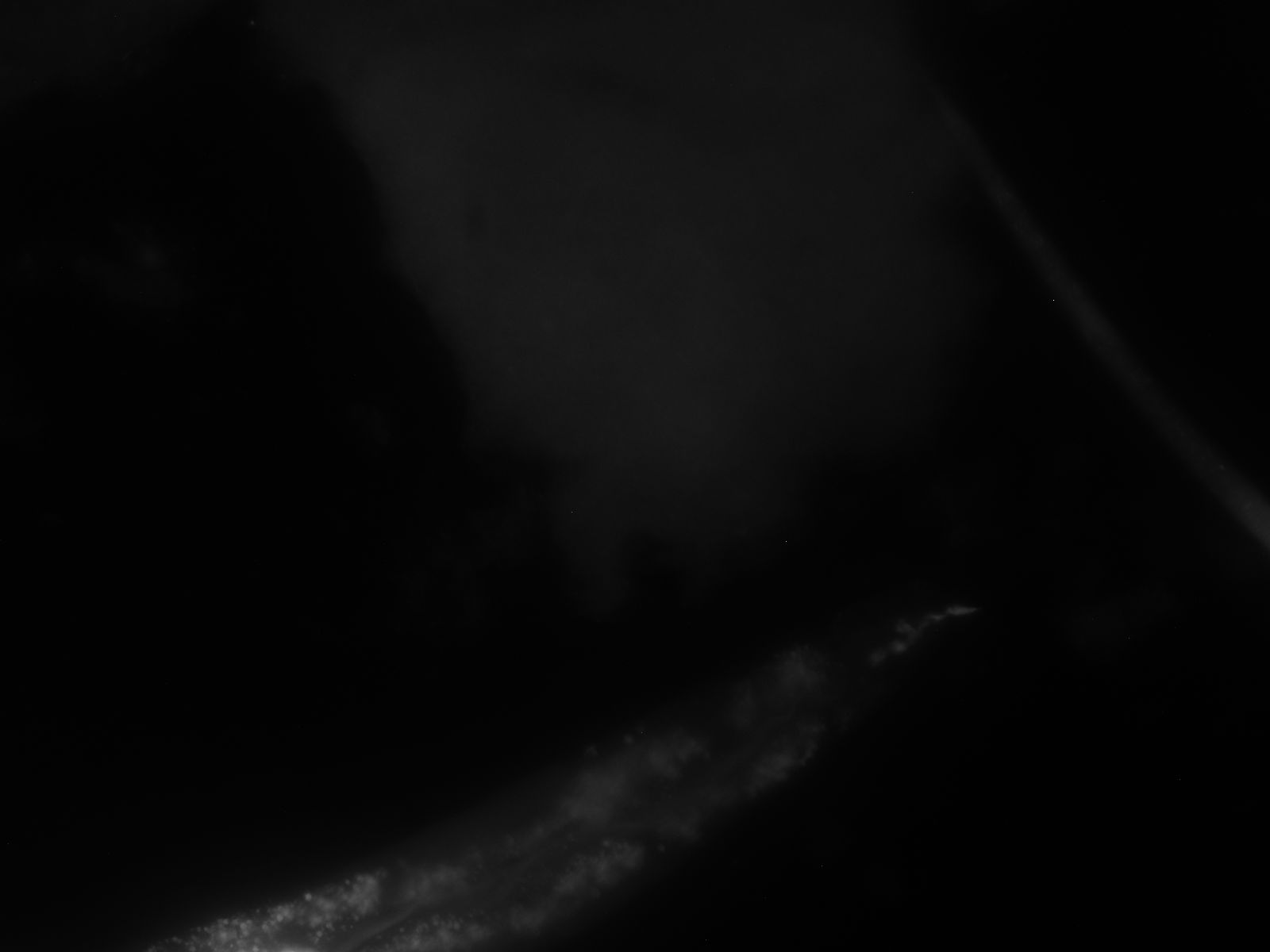

Supplement: S1 File — This file contains all the scoring data of the revised SYTO12 staining experiments. Each of the three biological replicates for Figs 2A, 4D, S2 and S4B–S4C were done in parallel in all strains. Hence, the wild type animals in Fig 2A and in S2 Fig are the same. In most cases animals were scored by live imaging without accompanied image acquisition. Representative images are provided. Consecutive images may image the same gonad. The scoring of apoptotic corpses was performed per gonad, not per image. (ZIP) [file pgen.1011061.s001.zip › SYTO staining experiment united/syto12 staining - 1_rep - 14.5.23 - JPEG/n2+pad1221.jpg]

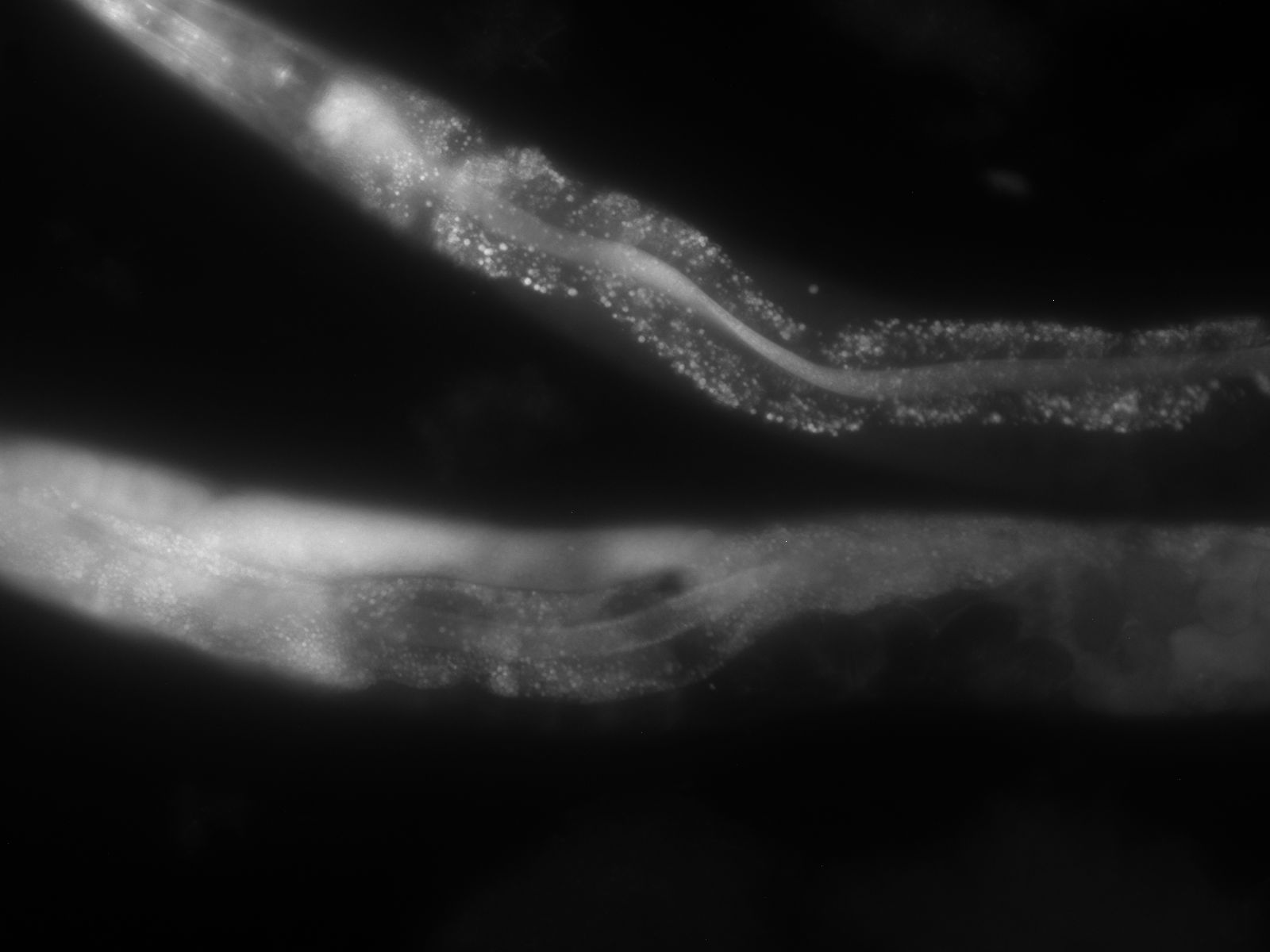

Supplement: S1 File — This file contains all the scoring data of the revised SYTO12 staining experiments. Each of the three biological replicates for Figs 2A, 4D, S2 and S4B–S4C were done in parallel in all strains. Hence, the wild type animals in Fig 2A and in S2 Fig are the same. In most cases animals were scored by live imaging without accompanied image acquisition. Representative images are provided. Consecutive images may image the same gonad. The scoring of apoptotic corpses was performed per gonad, not per image. (ZIP) [file pgen.1011061.s001.zip › SYTO staining experiment united/syto12 staining - 1_rep - 14.5.23 - JPEG/n2+pad1222.jpg]

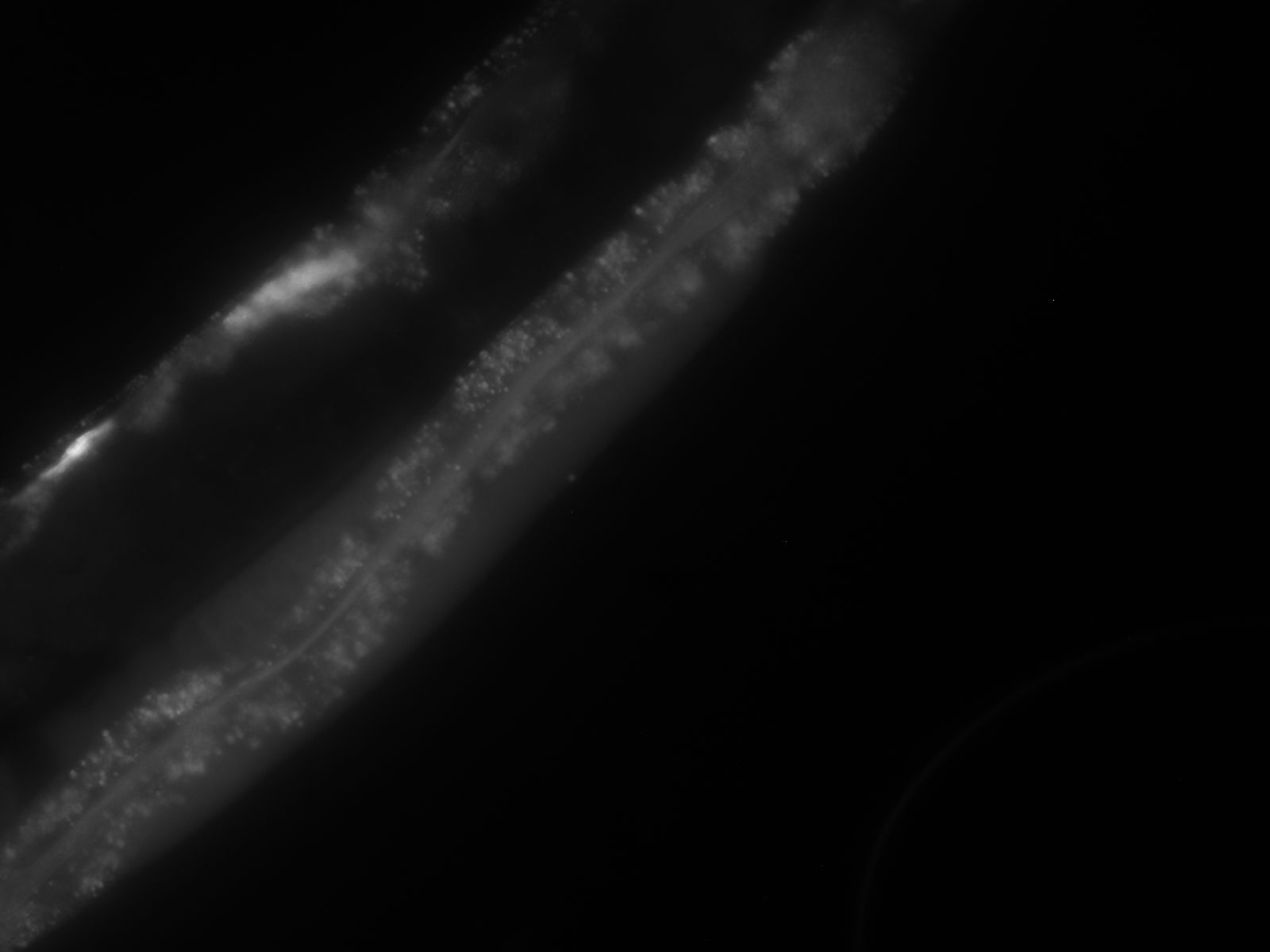

Supplement: S1 File — This file contains all the scoring data of the revised SYTO12 staining experiments. Each of the three biological replicates for Figs 2A, 4D, S2 and S4B–S4C were done in parallel in all strains. Hence, the wild type animals in Fig 2A and in S2 Fig are the same. In most cases animals were scored by live imaging without accompanied image acquisition. Representative images are provided. Consecutive images may image the same gonad. The scoring of apoptotic corpses was performed per gonad, not per image. (ZIP) [file pgen.1011061.s001.zip › SYTO staining experiment united/syto12 staining - 1_rep - 14.5.23 - JPEG/n2+pad1223.jpg]

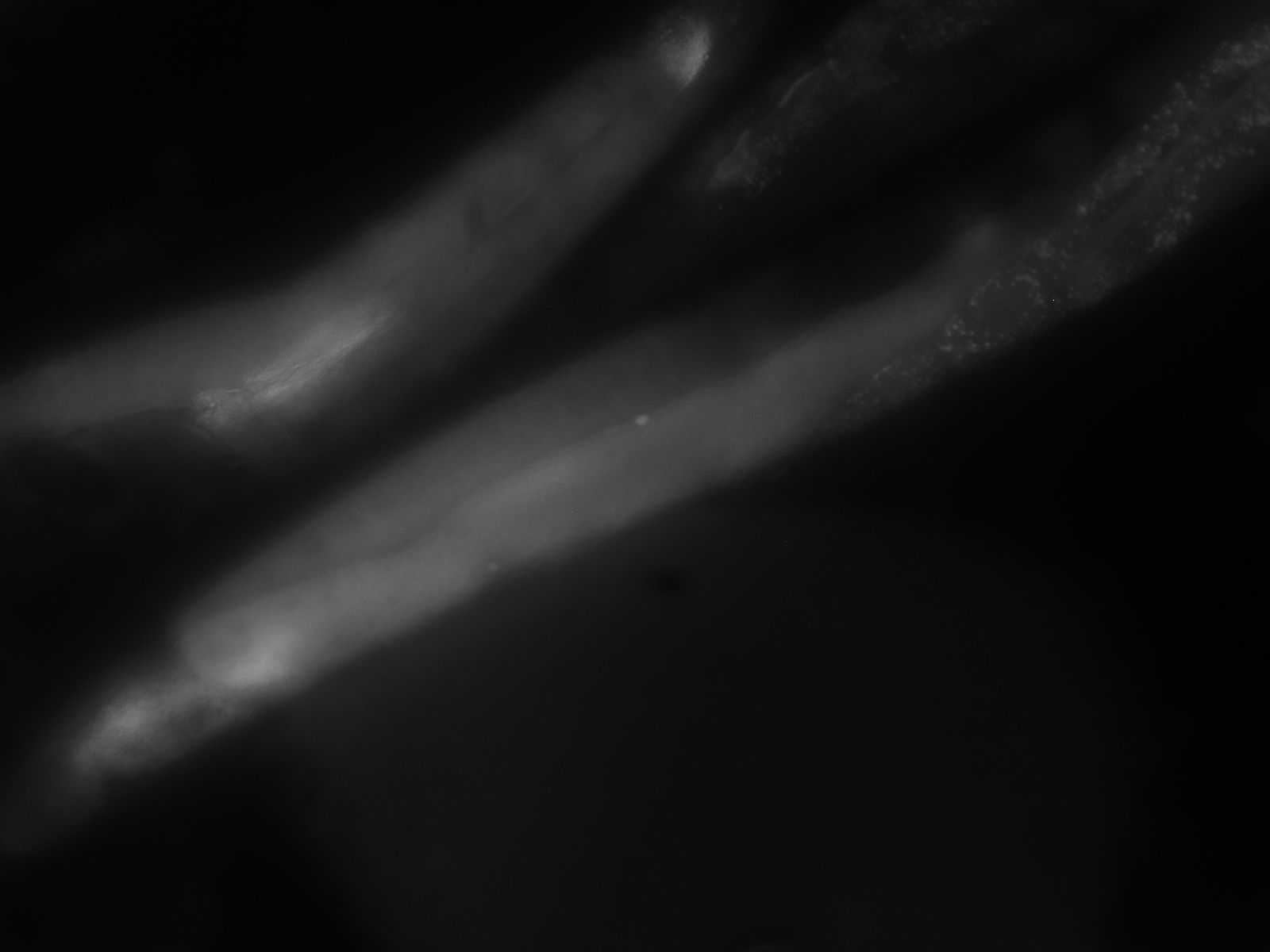

Supplement: S1 File — This file contains all the scoring data of the revised SYTO12 staining experiments. Each of the three biological replicates for Figs 2A, 4D, S2 and S4B–S4C were done in parallel in all strains. Hence, the wild type animals in Fig 2A and in S2 Fig are the same. In most cases animals were scored by live imaging without accompanied image acquisition. Representative images are provided. Consecutive images may image the same gonad. The scoring of apoptotic corpses was performed per gonad, not per image. (ZIP) [file pgen.1011061.s001.zip › SYTO staining experiment united/syto12 staining - 1_rep - 14.5.23 - JPEG/n2+pad1224.jpg]

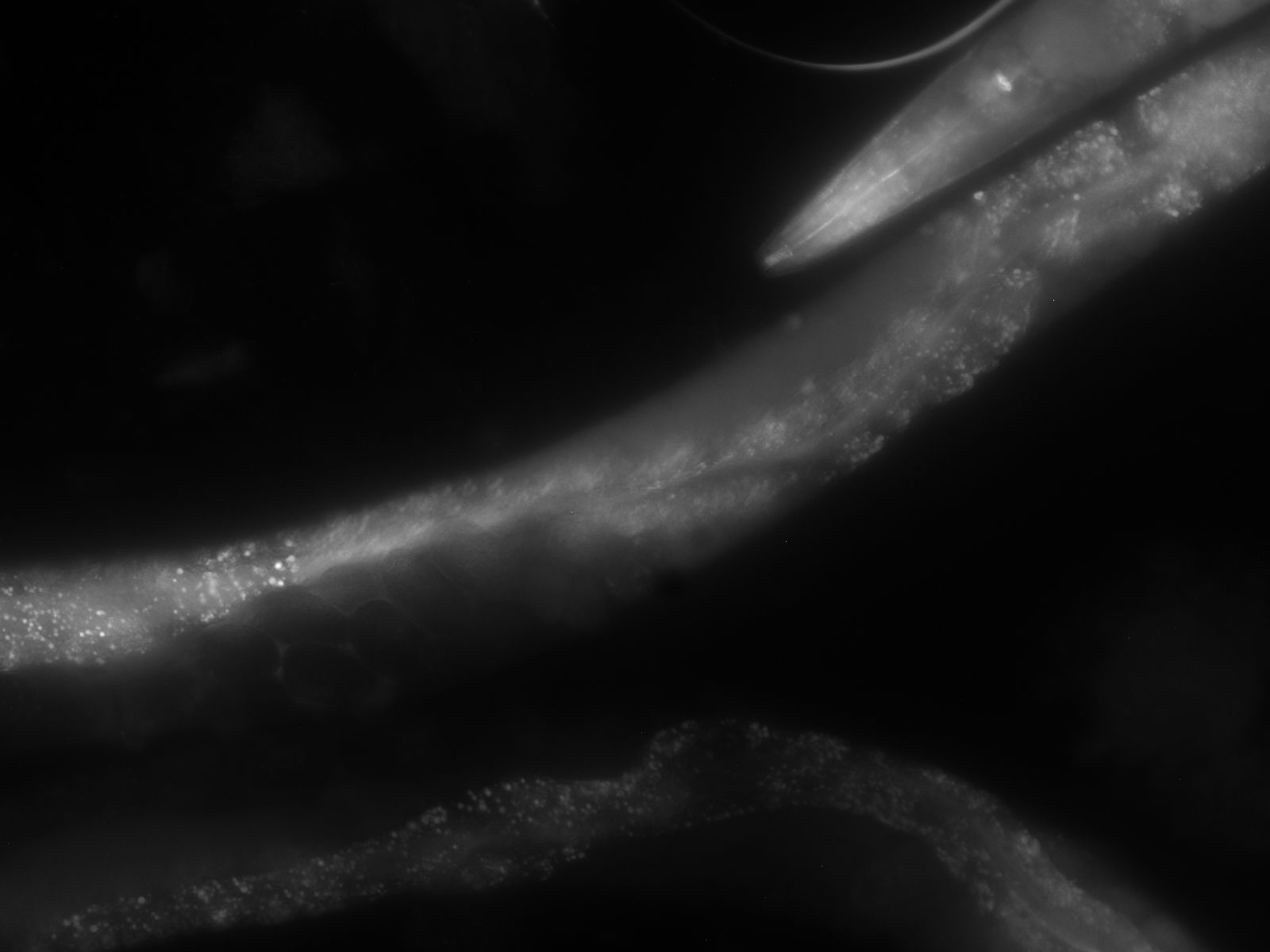

Supplement: S1 File — This file contains all the scoring data of the revised SYTO12 staining experiments. Each of the three biological replicates for Figs 2A, 4D, S2 and S4B–S4C were done in parallel in all strains. Hence, the wild type animals in Fig 2A and in S2 Fig are the same. In most cases animals were scored by live imaging without accompanied image acquisition. Representative images are provided. Consecutive images may image the same gonad. The scoring of apoptotic corpses was performed per gonad, not per image. (ZIP) [file pgen.1011061.s001.zip › SYTO staining experiment united/syto12 staining - 1_rep - 14.5.23 - JPEG/n2+pad1225.jpg]

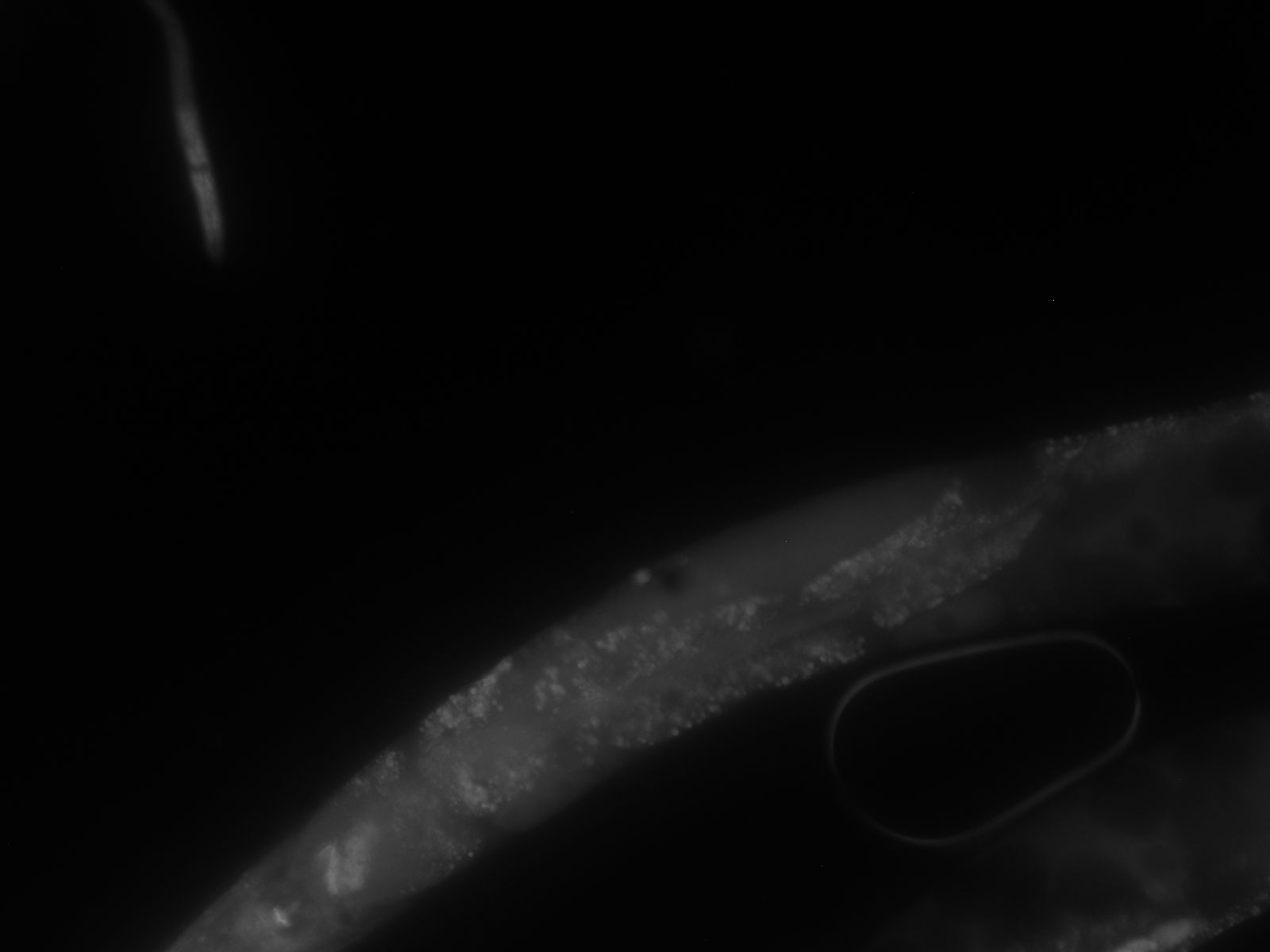

Supplement: S1 File — This file contains all the scoring data of the revised SYTO12 staining experiments. Each of the three biological replicates for Figs 2A, 4D, S2 and S4B–S4C were done in parallel in all strains. Hence, the wild type animals in Fig 2A and in S2 Fig are the same. In most cases animals were scored by live imaging without accompanied image acquisition. Representative images are provided. Consecutive images may image the same gonad. The scoring of apoptotic corpses was performed per gonad, not per image. (ZIP) [file pgen.1011061.s001.zip › SYTO staining experiment united/syto12 staining - 1_rep - 14.5.23 - JPEG/n2+pad1226.jpg]

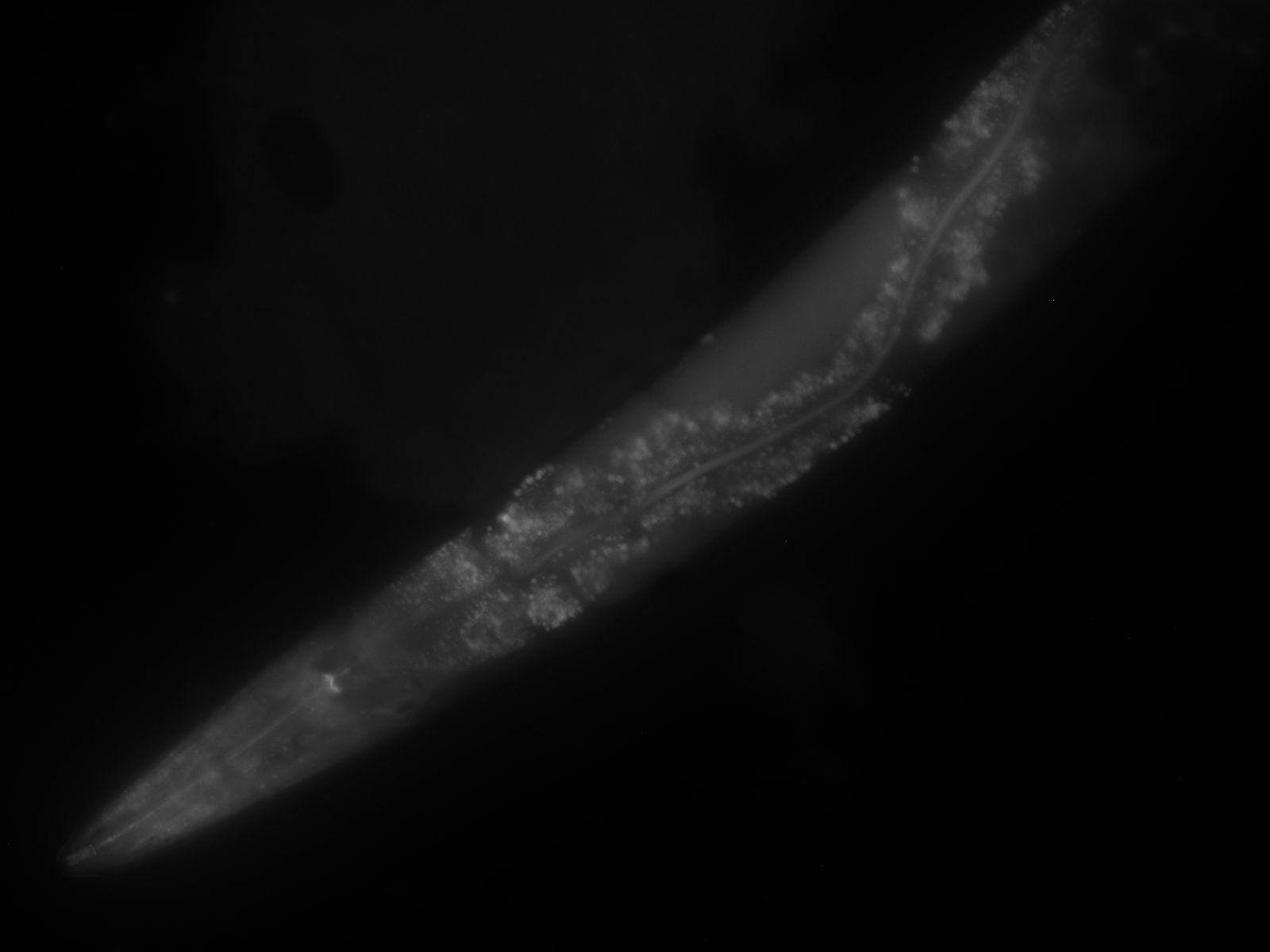

Supplement: S1 File — This file contains all the scoring data of the revised SYTO12 staining experiments. Each of the three biological replicates for Figs 2A, 4D, S2 and S4B–S4C were done in parallel in all strains. Hence, the wild type animals in Fig 2A and in S2 Fig are the same. In most cases animals were scored by live imaging without accompanied image acquisition. Representative images are provided. Consecutive images may image the same gonad. The scoring of apoptotic corpses was performed per gonad, not per image. (ZIP) [file pgen.1011061.s001.zip › SYTO staining experiment united/syto12 staining - 1_rep - 14.5.23 - JPEG/n2+pad1227.jpg]

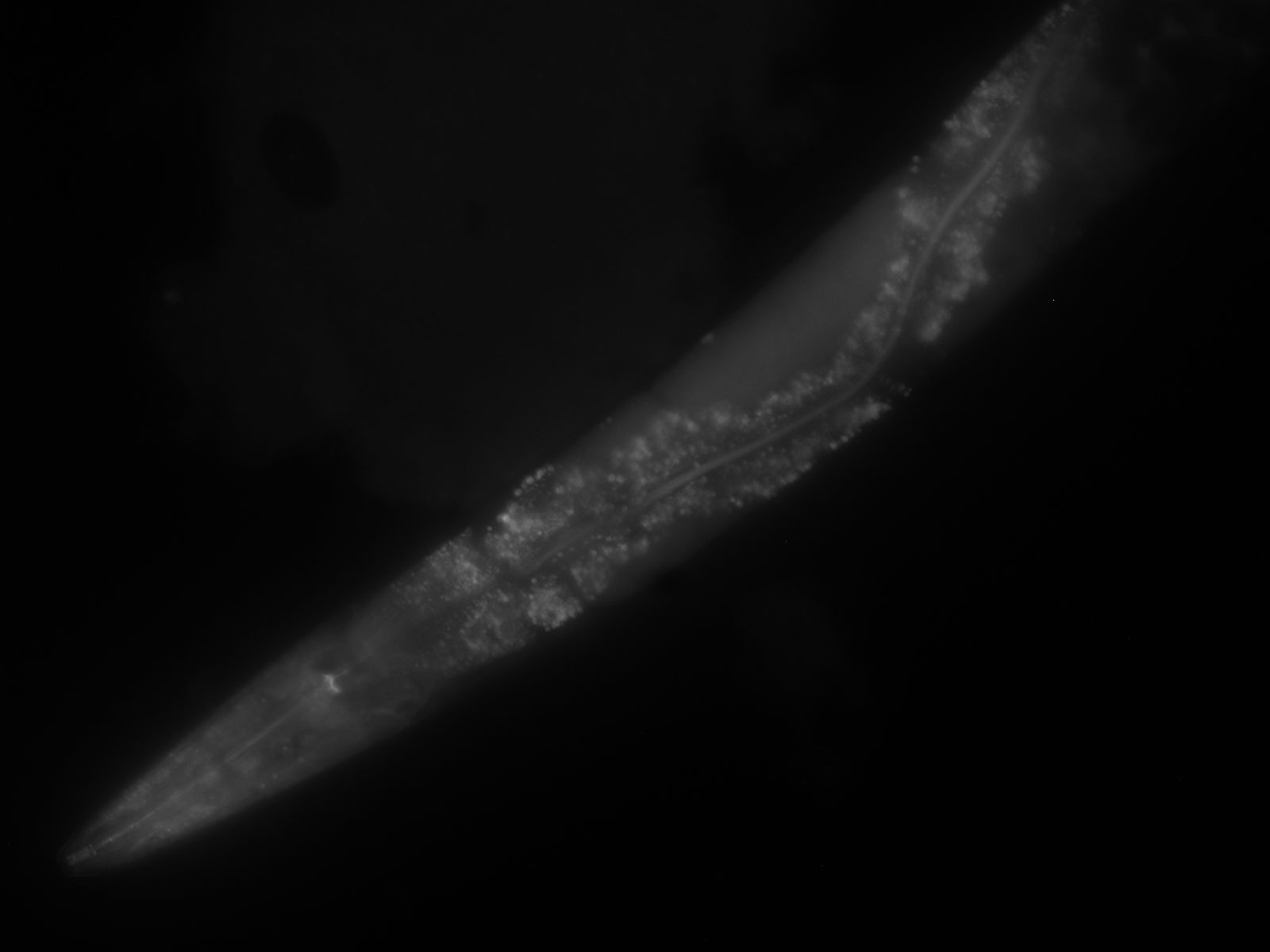

Supplement: S1 File — This file contains all the scoring data of the revised SYTO12 staining experiments. Each of the three biological replicates for Figs 2A, 4D, S2 and S4B–S4C were done in parallel in all strains. Hence, the wild type animals in Fig 2A and in S2 Fig are the same. In most cases animals were scored by live imaging without accompanied image acquisition. Representative images are provided. Consecutive images may image the same gonad. The scoring of apoptotic corpses was performed per gonad, not per image. (ZIP) [file pgen.1011061.s001.zip › SYTO staining experiment united/syto12 staining - 1_rep - 14.5.23 - JPEG/n2+pad1228.jpg]

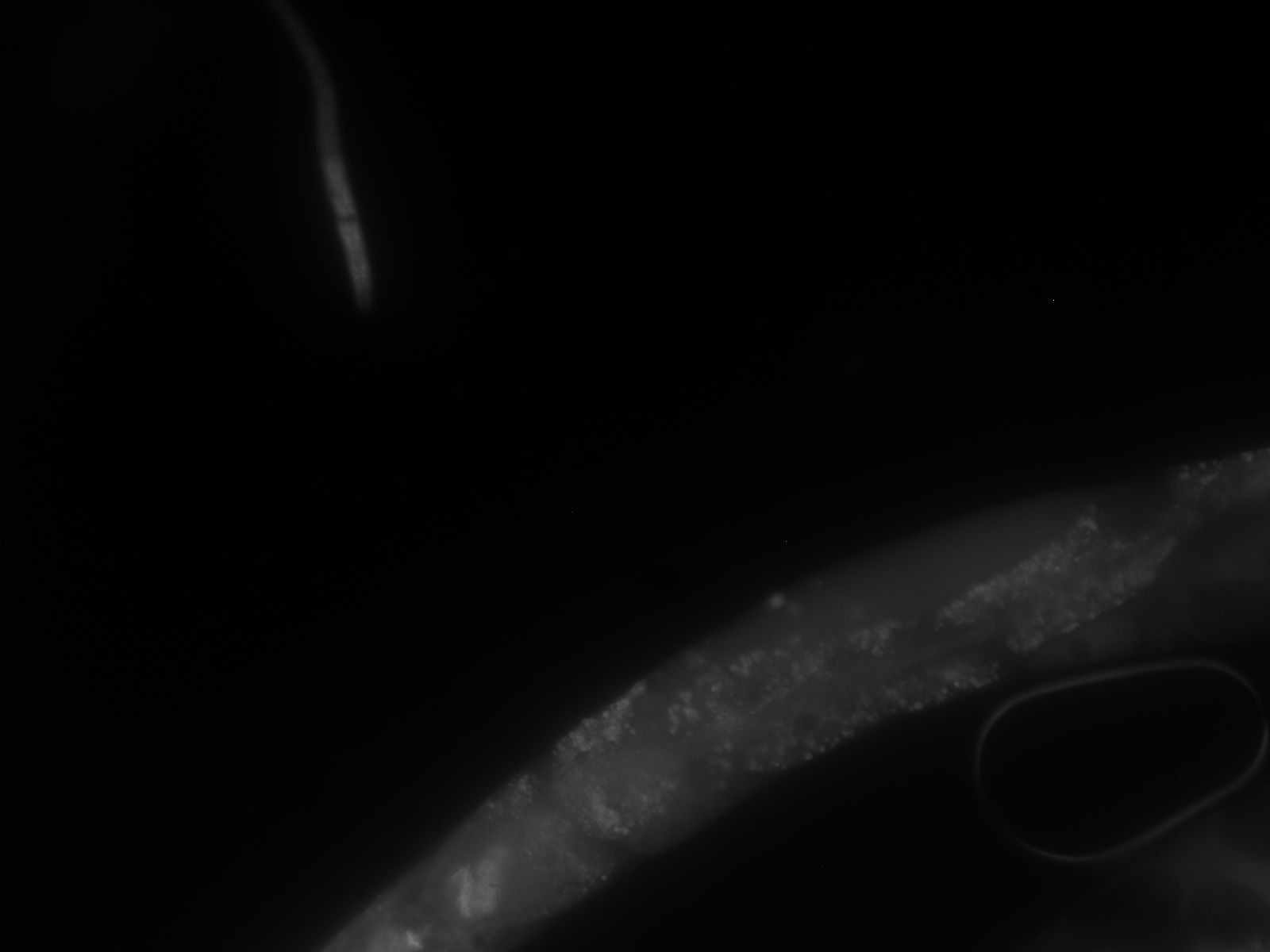

Supplement: S1 File — This file contains all the scoring data of the revised SYTO12 staining experiments. Each of the three biological replicates for Figs 2A, 4D, S2 and S4B–S4C were done in parallel in all strains. Hence, the wild type animals in Fig 2A and in S2 Fig are the same. In most cases animals were scored by live imaging without accompanied image acquisition. Representative images are provided. Consecutive images may image the same gonad. The scoring of apoptotic corpses was performed per gonad, not per image. (ZIP) [file pgen.1011061.s001.zip › SYTO staining experiment united/syto12 staining - 1_rep - 14.5.23 - JPEG/n2+pad1229.jpg]

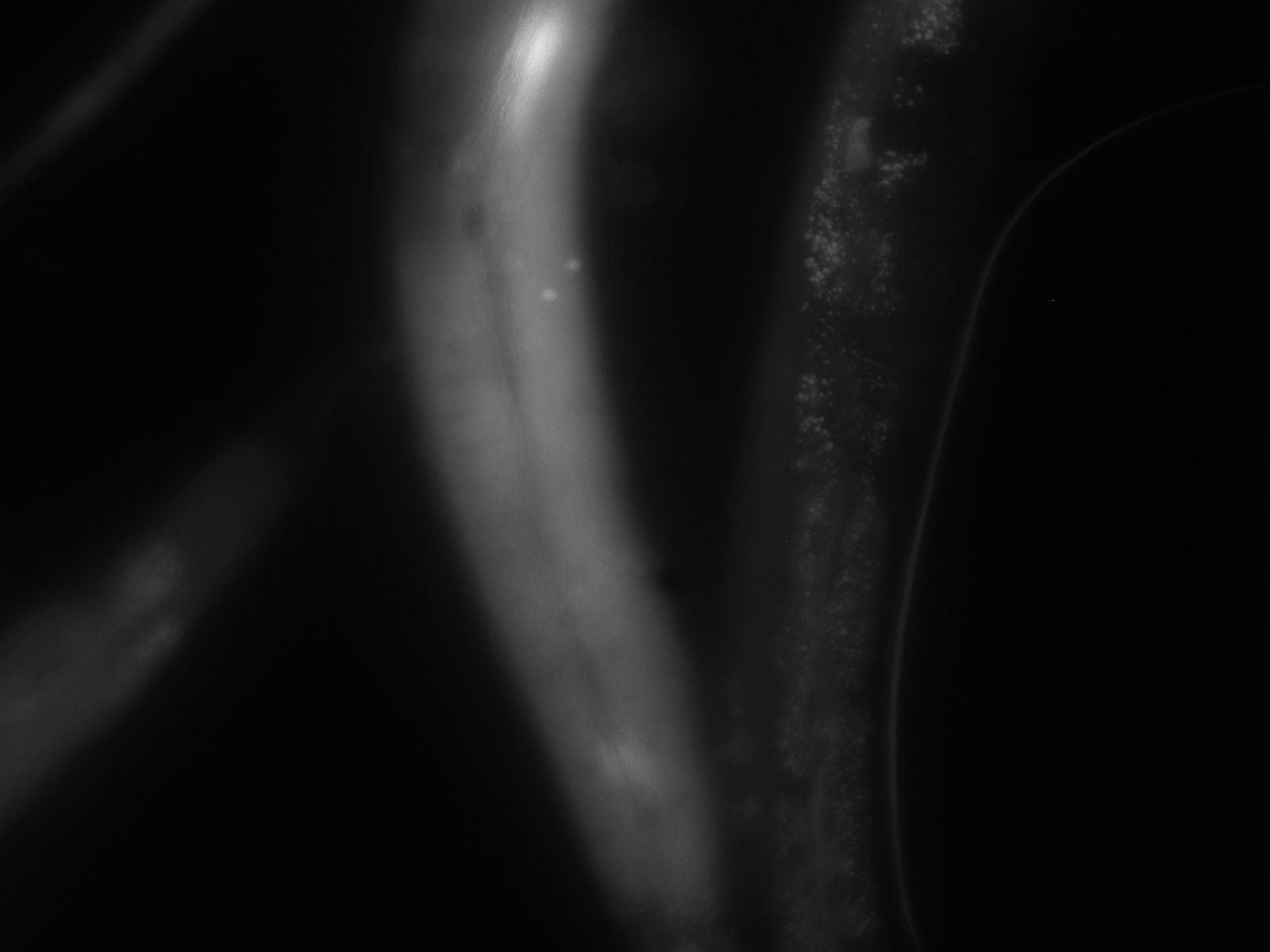

Supplement: S1 File — This file contains all the scoring data of the revised SYTO12 staining experiments. Each of the three biological replicates for Figs 2A, 4D, S2 and S4B–S4C were done in parallel in all strains. Hence, the wild type animals in Fig 2A and in S2 Fig are the same. In most cases animals were scored by live imaging without accompanied image acquisition. Representative images are provided. Consecutive images may image the same gonad. The scoring of apoptotic corpses was performed per gonad, not per image. (ZIP) [file pgen.1011061.s001.zip › SYTO staining experiment united/syto12 staining - 1_rep - 14.5.23 - JPEG/n2+pad1230.jpg]

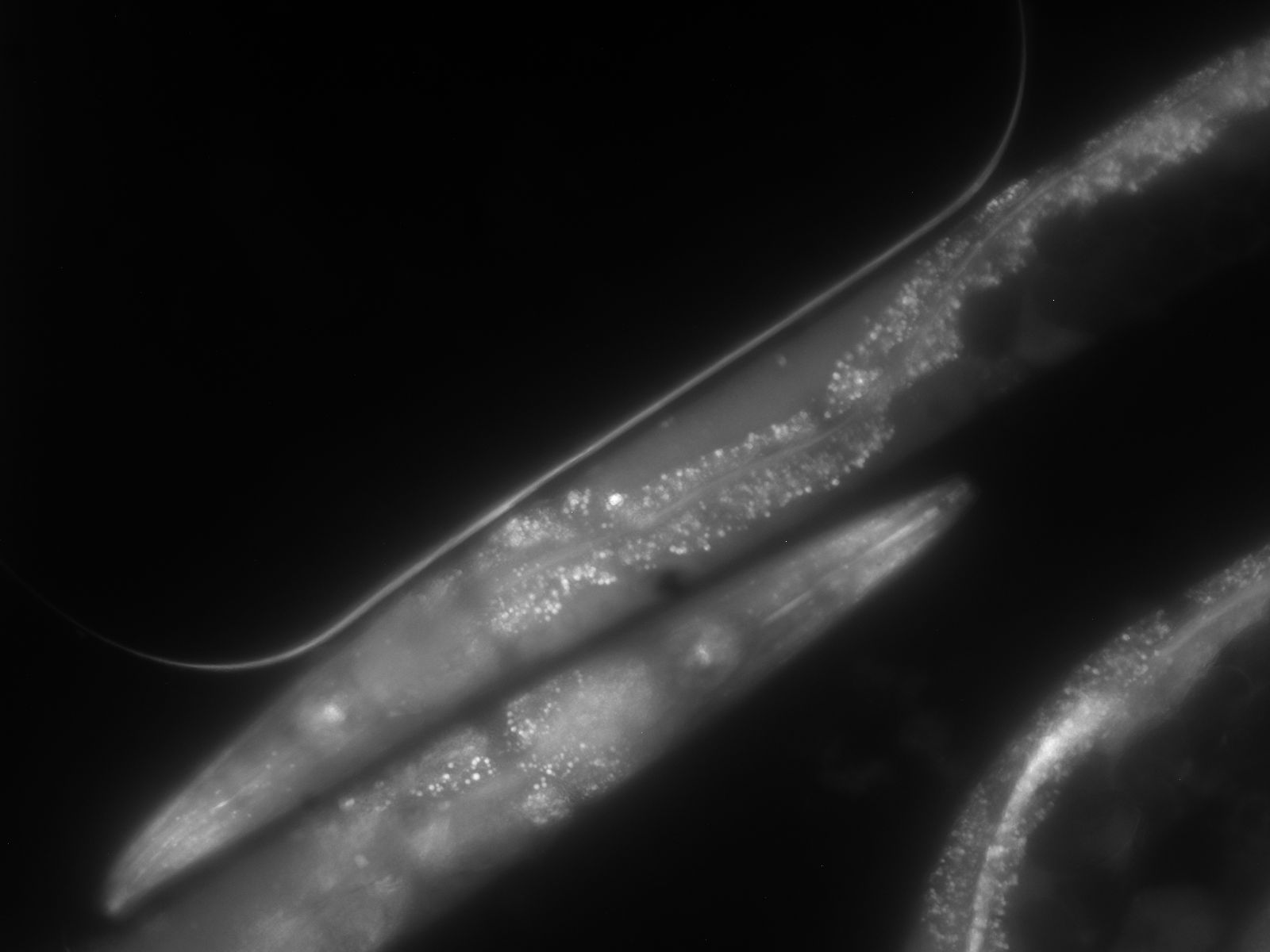

Supplement: S1 File — This file contains all the scoring data of the revised SYTO12 staining experiments. Each of the three biological replicates for Figs 2A, 4D, S2 and S4B–S4C were done in parallel in all strains. Hence, the wild type animals in Fig 2A and in S2 Fig are the same. In most cases animals were scored by live imaging without accompanied image acquisition. Representative images are provided. Consecutive images may image the same gonad. The scoring of apoptotic corpses was performed per gonad, not per image. (ZIP) [file pgen.1011061.s001.zip › SYTO staining experiment united/syto12 staining - 1_rep - 14.5.23 - JPEG/n2+pad1231.jpg]

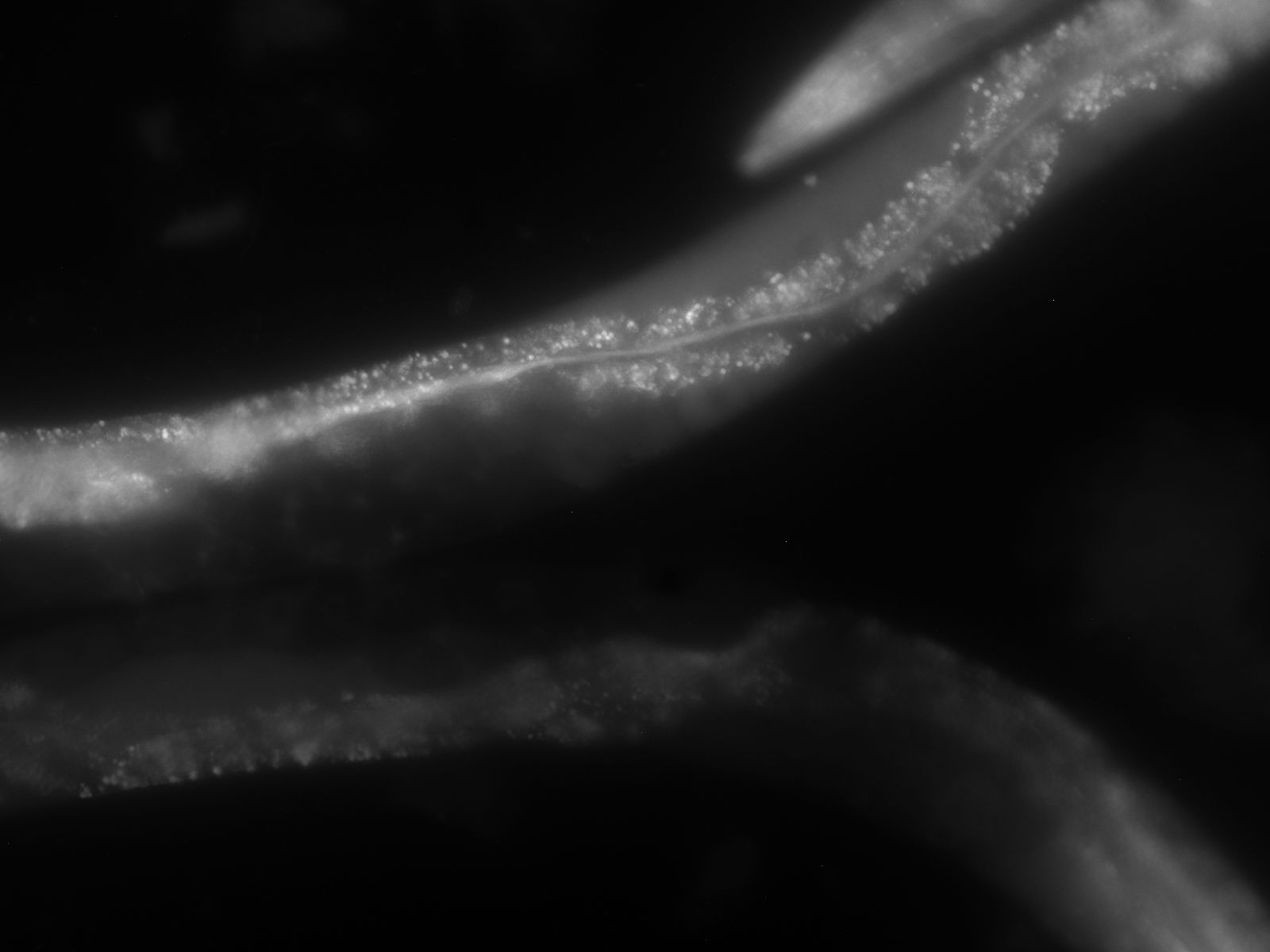

Supplement: S1 File — This file contains all the scoring data of the revised SYTO12 staining experiments. Each of the three biological replicates for Figs 2A, 4D, S2 and S4B–S4C were done in parallel in all strains. Hence, the wild type animals in Fig 2A and in S2 Fig are the same. In most cases animals were scored by live imaging without accompanied image acquisition. Representative images are provided. Consecutive images may image the same gonad. The scoring of apoptotic corpses was performed per gonad, not per image. (ZIP) [file pgen.1011061.s001.zip › SYTO staining experiment united/syto12 staining - 1_rep - 14.5.23 - JPEG/n2+pad1232.jpg]

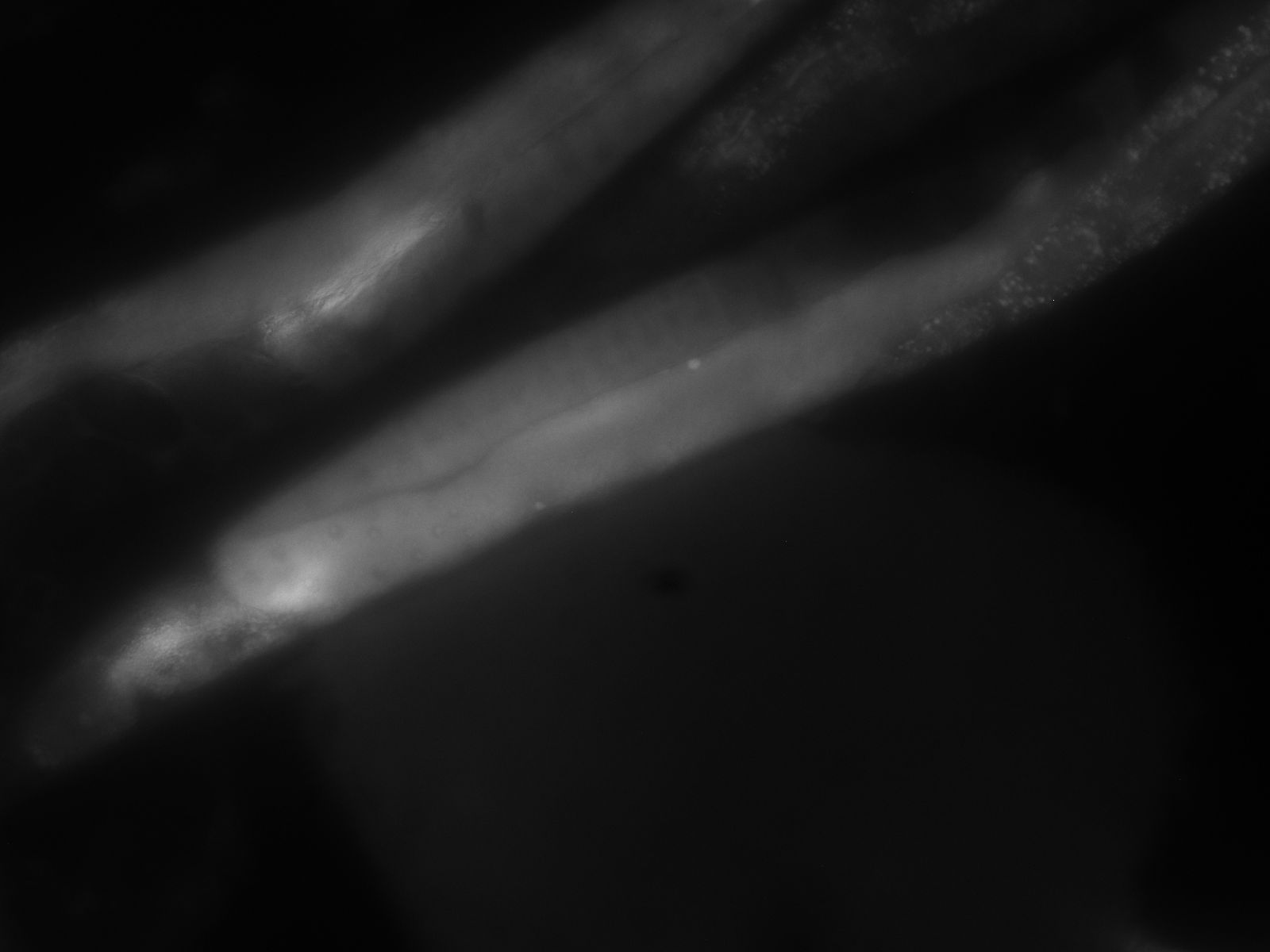

Supplement: S1 File — This file contains all the scoring data of the revised SYTO12 staining experiments. Each of the three biological replicates for Figs 2A, 4D, S2 and S4B–S4C were done in parallel in all strains. Hence, the wild type animals in Fig 2A and in S2 Fig are the same. In most cases animals were scored by live imaging without accompanied image acquisition. Representative images are provided. Consecutive images may image the same gonad. The scoring of apoptotic corpses was performed per gonad, not per image. (ZIP) [file pgen.1011061.s001.zip › SYTO staining experiment united/syto12 staining - 1_rep - 14.5.23 - JPEG/n2+pad1233.jpg]

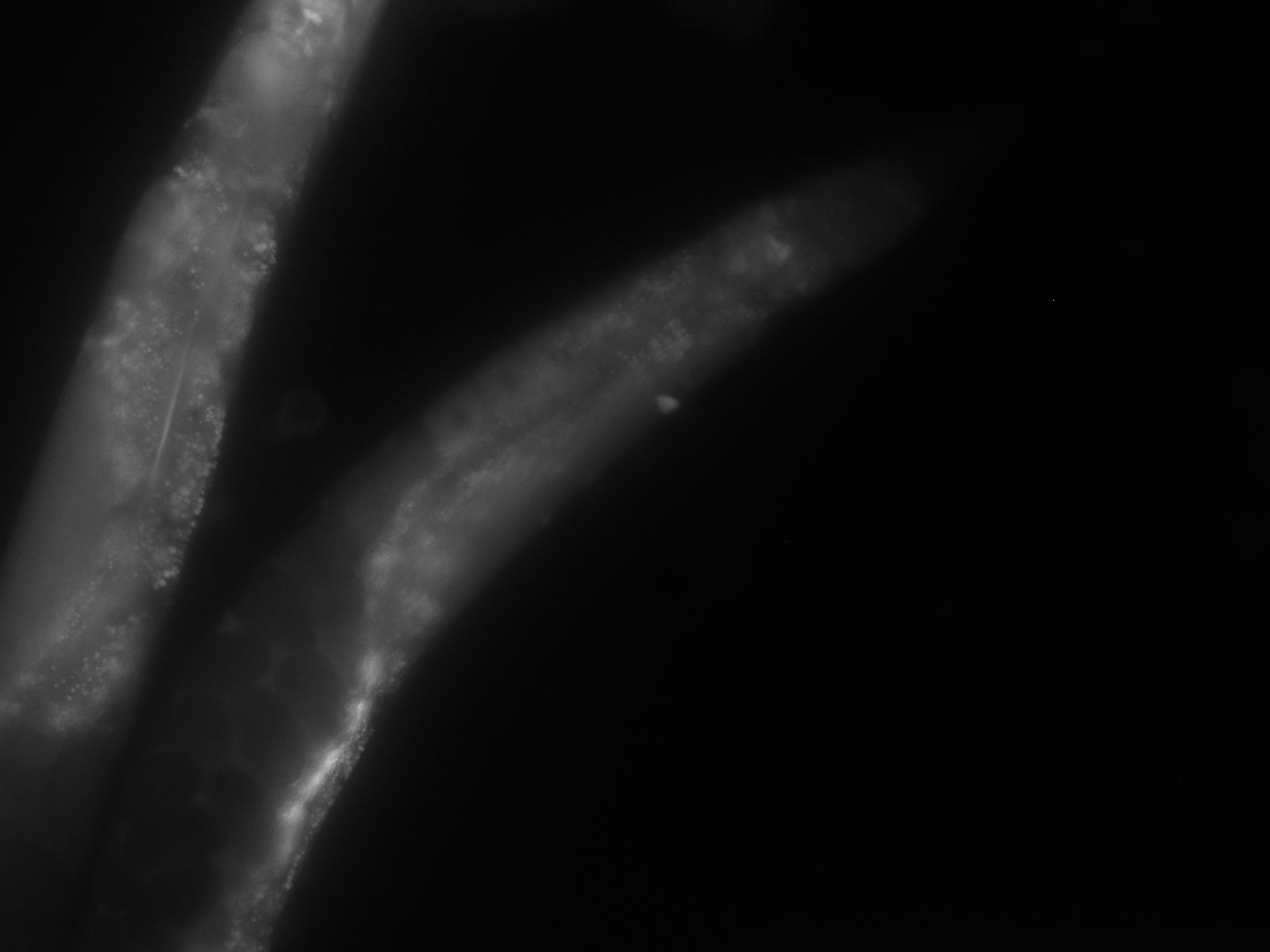

Supplement: S1 File — This file contains all the scoring data of the revised SYTO12 staining experiments. Each of the three biological replicates for Figs 2A, 4D, S2 and S4B–S4C were done in parallel in all strains. Hence, the wild type animals in Fig 2A and in S2 Fig are the same. In most cases animals were scored by live imaging without accompanied image acquisition. Representative images are provided. Consecutive images may image the same gonad. The scoring of apoptotic corpses was performed per gonad, not per image. (ZIP) [file pgen.1011061.s001.zip › SYTO staining experiment united/syto12 staining - 1_rep - 14.5.23 - JPEG/n2+pad1234.jpg]

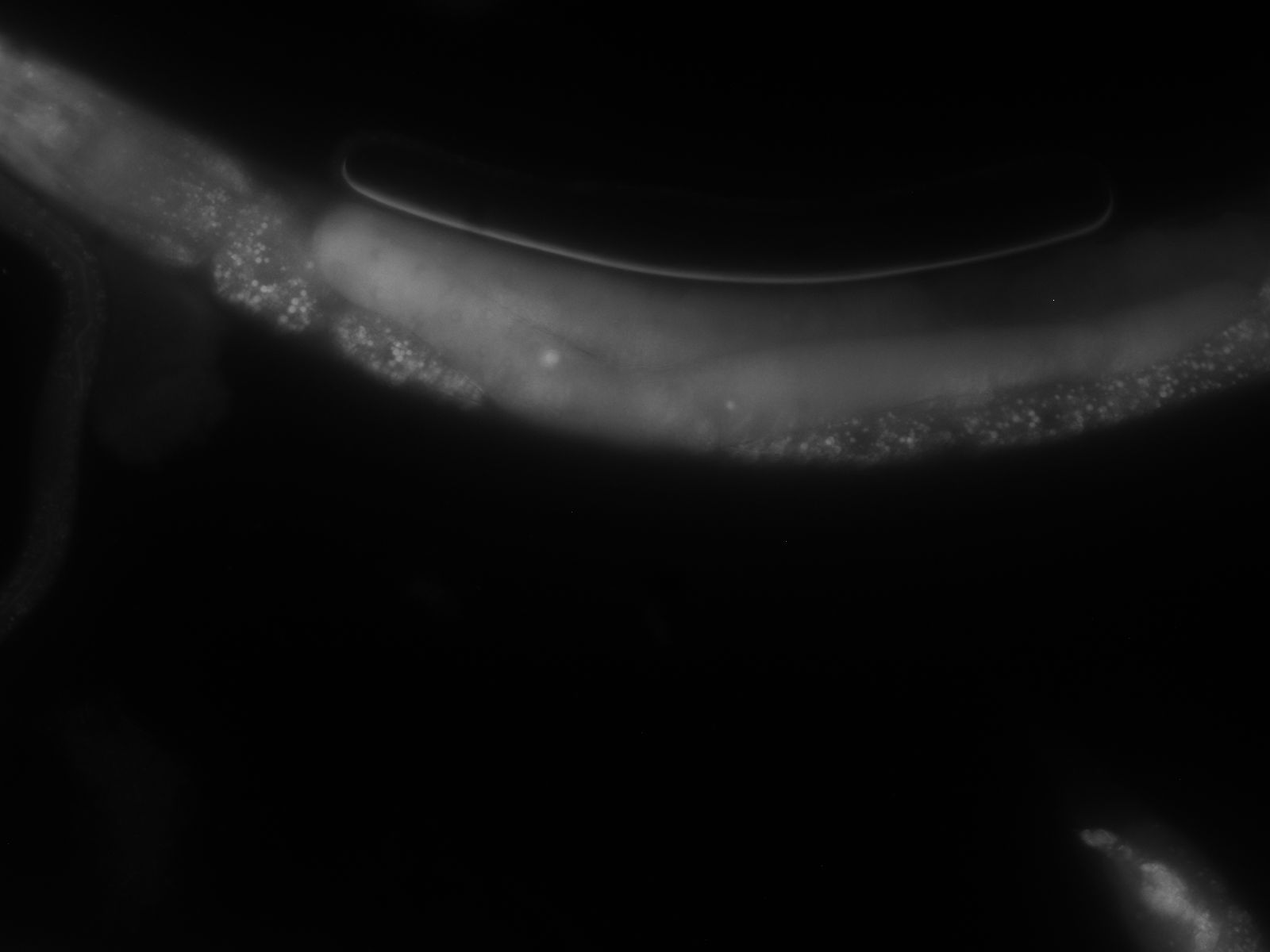

Supplement: S1 File — This file contains all the scoring data of the revised SYTO12 staining experiments. Each of the three biological replicates for Figs 2A, 4D, S2 and S4B–S4C were done in parallel in all strains. Hence, the wild type animals in Fig 2A and in S2 Fig are the same. In most cases animals were scored by live imaging without accompanied image acquisition. Representative images are provided. Consecutive images may image the same gonad. The scoring of apoptotic corpses was performed per gonad, not per image. (ZIP) [file pgen.1011061.s001.zip › SYTO staining experiment united/syto12 staining - 1_rep - 14.5.23 - JPEG/n2+pad1235.jpg]

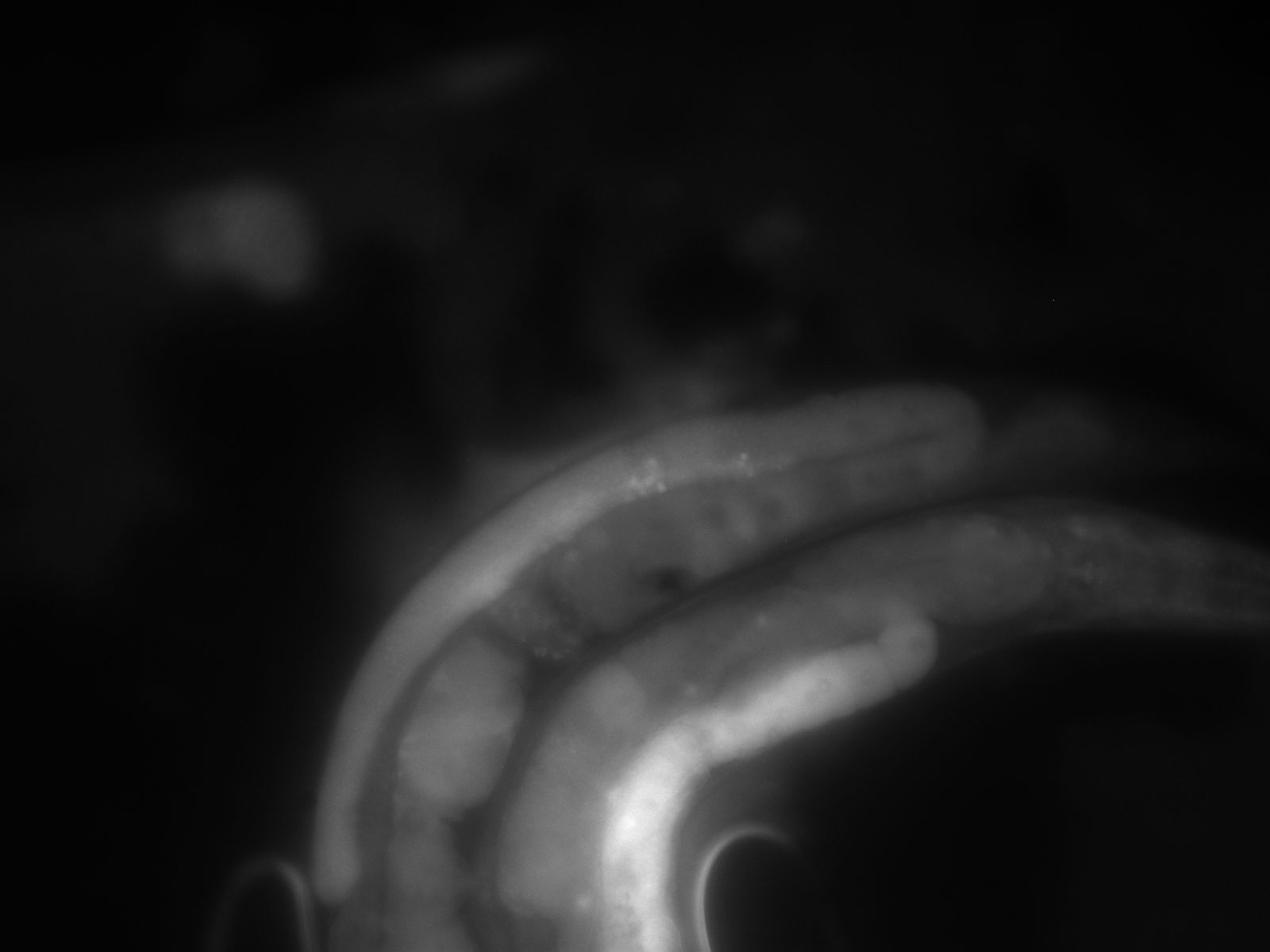

Supplement: S1 File — This file contains all the scoring data of the revised SYTO12 staining experiments. Each of the three biological replicates for Figs 2A, 4D, S2 and S4B–S4C were done in parallel in all strains. Hence, the wild type animals in Fig 2A and in S2 Fig are the same. In most cases animals were scored by live imaging without accompanied image acquisition. Representative images are provided. Consecutive images may image the same gonad. The scoring of apoptotic corpses was performed per gonad, not per image. (ZIP) [file pgen.1011061.s001.zip › SYTO staining experiment united/syto12 staining - 1_rep - 14.5.23 - JPEG/n2+tfg-1_189.jpg]

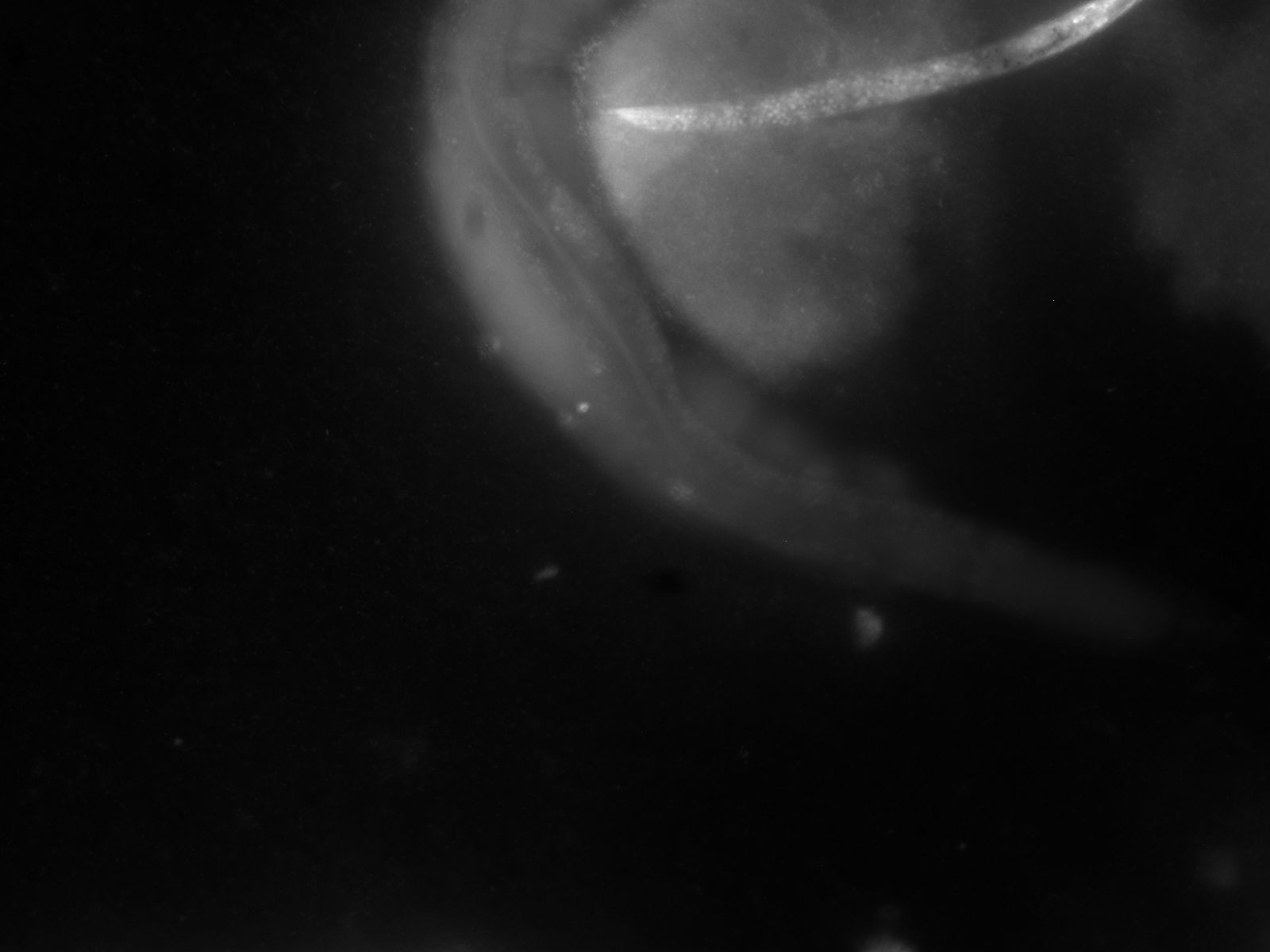

Supplement: S1 File — This file contains all the scoring data of the revised SYTO12 staining experiments. Each of the three biological replicates for Figs 2A, 4D, S2 and S4B–S4C were done in parallel in all strains. Hence, the wild type animals in Fig 2A and in S2 Fig are the same. In most cases animals were scored by live imaging without accompanied image acquisition. Representative images are provided. Consecutive images may image the same gonad. The scoring of apoptotic corpses was performed per gonad, not per image. (ZIP) [file pgen.1011061.s001.zip › SYTO staining experiment united/syto12 staining - 1_rep - 14.5.23 - JPEG/n2+tfg-1_190.jpg]

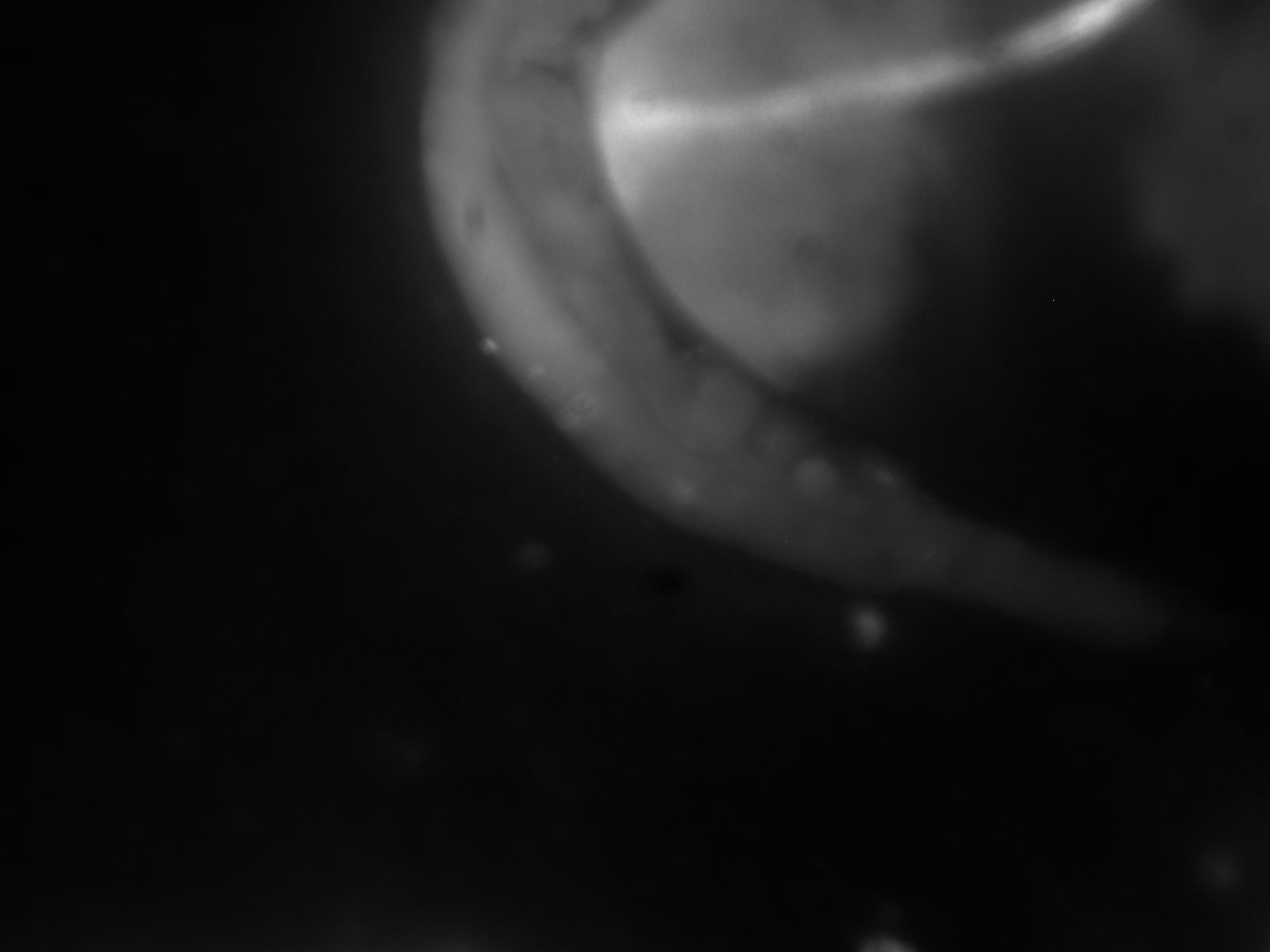

Supplement: S1 File — This file contains all the scoring data of the revised SYTO12 staining experiments. Each of the three biological replicates for Figs 2A, 4D, S2 and S4B–S4C were done in parallel in all strains. Hence, the wild type animals in Fig 2A and in S2 Fig are the same. In most cases animals were scored by live imaging without accompanied image acquisition. Representative images are provided. Consecutive images may image the same gonad. The scoring of apoptotic corpses was performed per gonad, not per image. (ZIP) [file pgen.1011061.s001.zip › SYTO staining experiment united/syto12 staining - 1_rep - 14.5.23 - JPEG/n2+tfg-1_191.jpg]

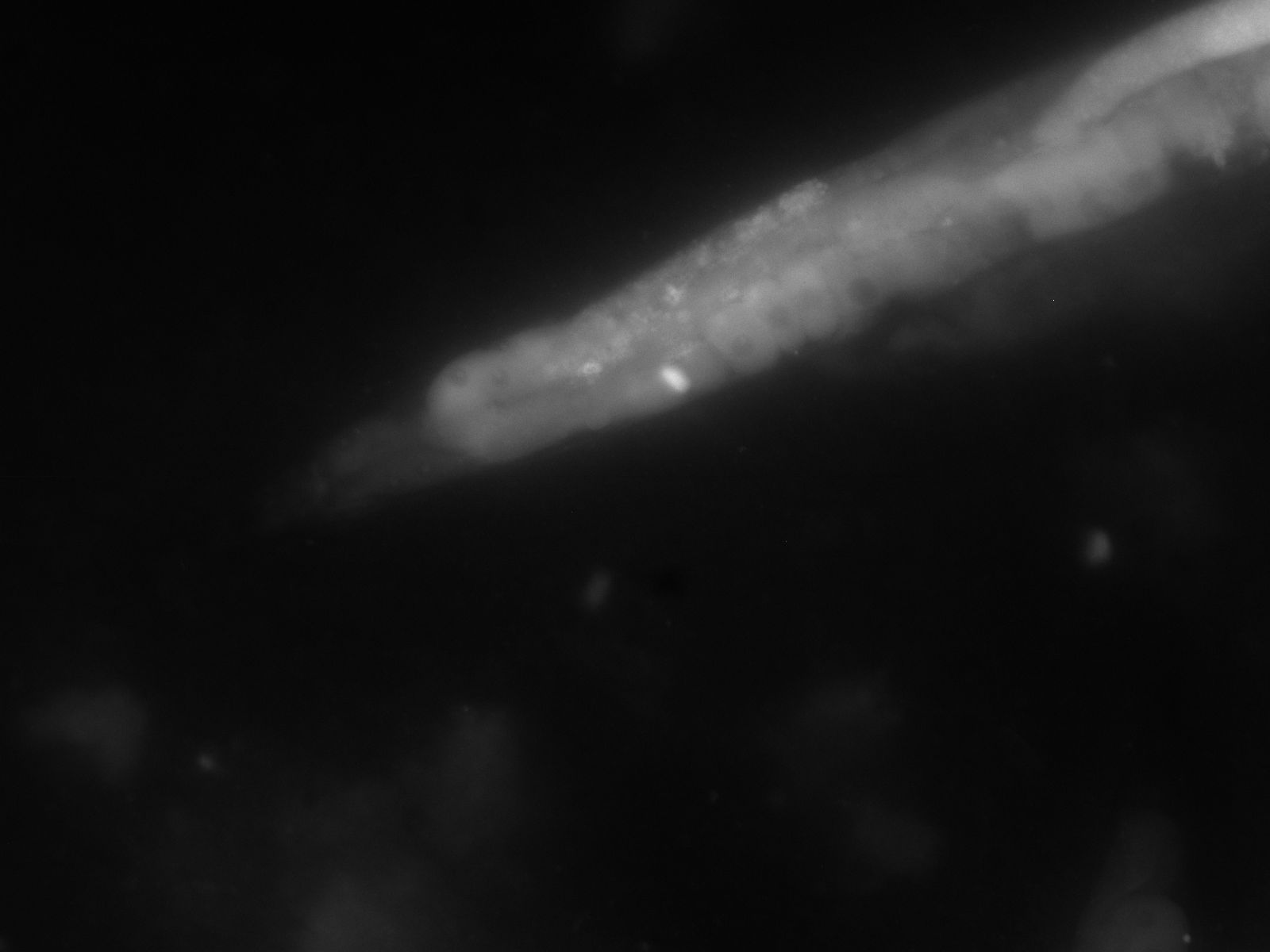

Supplement: S1 File — This file contains all the scoring data of the revised SYTO12 staining experiments. Each of the three biological replicates for Figs 2A, 4D, S2 and S4B–S4C were done in parallel in all strains. Hence, the wild type animals in Fig 2A and in S2 Fig are the same. In most cases animals were scored by live imaging without accompanied image acquisition. Representative images are provided. Consecutive images may image the same gonad. The scoring of apoptotic corpses was performed per gonad, not per image. (ZIP) [file pgen.1011061.s001.zip › SYTO staining experiment united/syto12 staining - 1_rep - 14.5.23 - JPEG/n2+tfg-1_192.jpg]

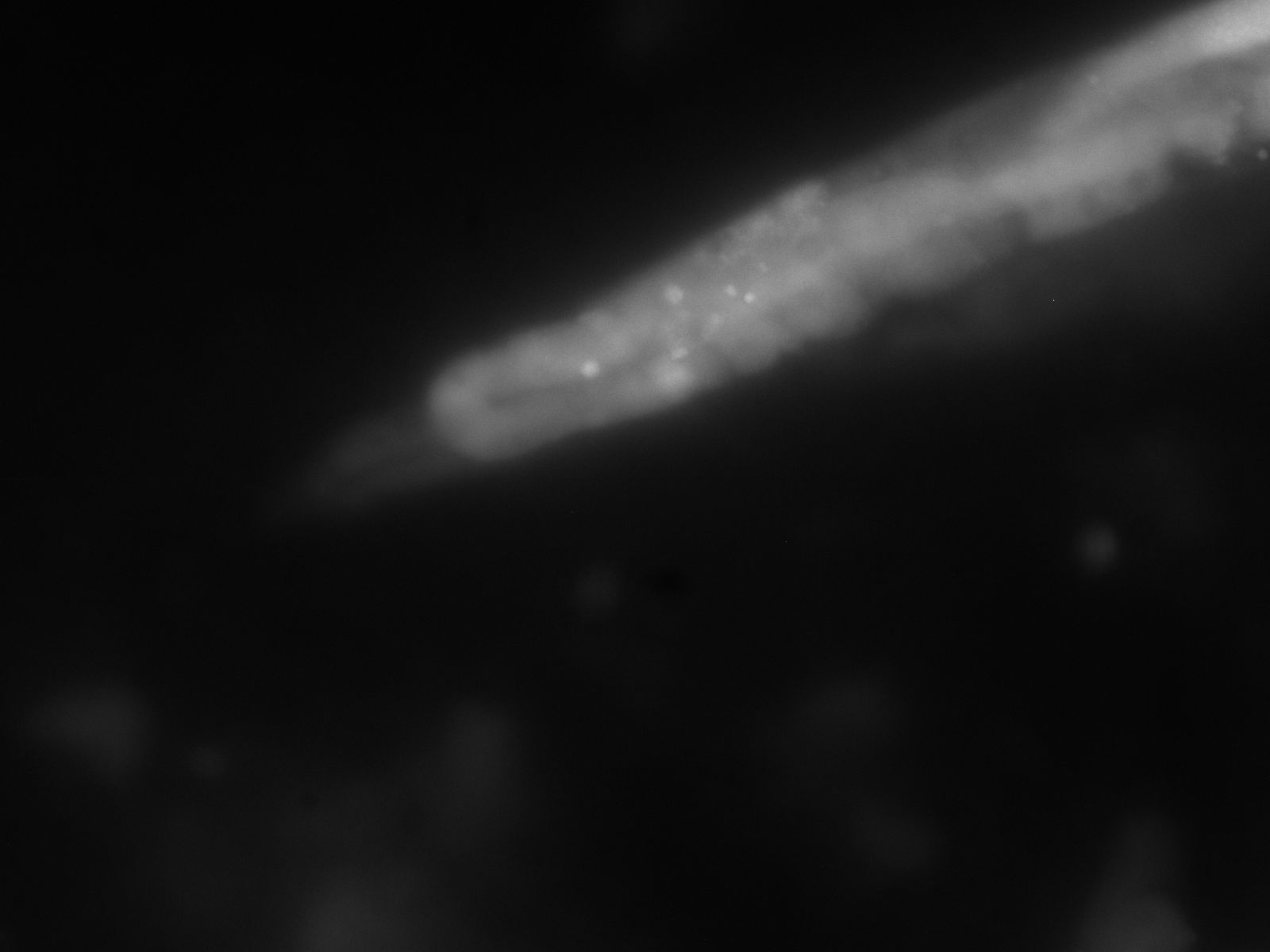

Supplement: S1 File — This file contains all the scoring data of the revised SYTO12 staining experiments. Each of the three biological replicates for Figs 2A, 4D, S2 and S4B–S4C were done in parallel in all strains. Hence, the wild type animals in Fig 2A and in S2 Fig are the same. In most cases animals were scored by live imaging without accompanied image acquisition. Representative images are provided. Consecutive images may image the same gonad. The scoring of apoptotic corpses was performed per gonad, not per image. (ZIP) [file pgen.1011061.s001.zip › SYTO staining experiment united/syto12 staining - 1_rep - 14.5.23 - JPEG/n2+tfg-1_193.jpg]

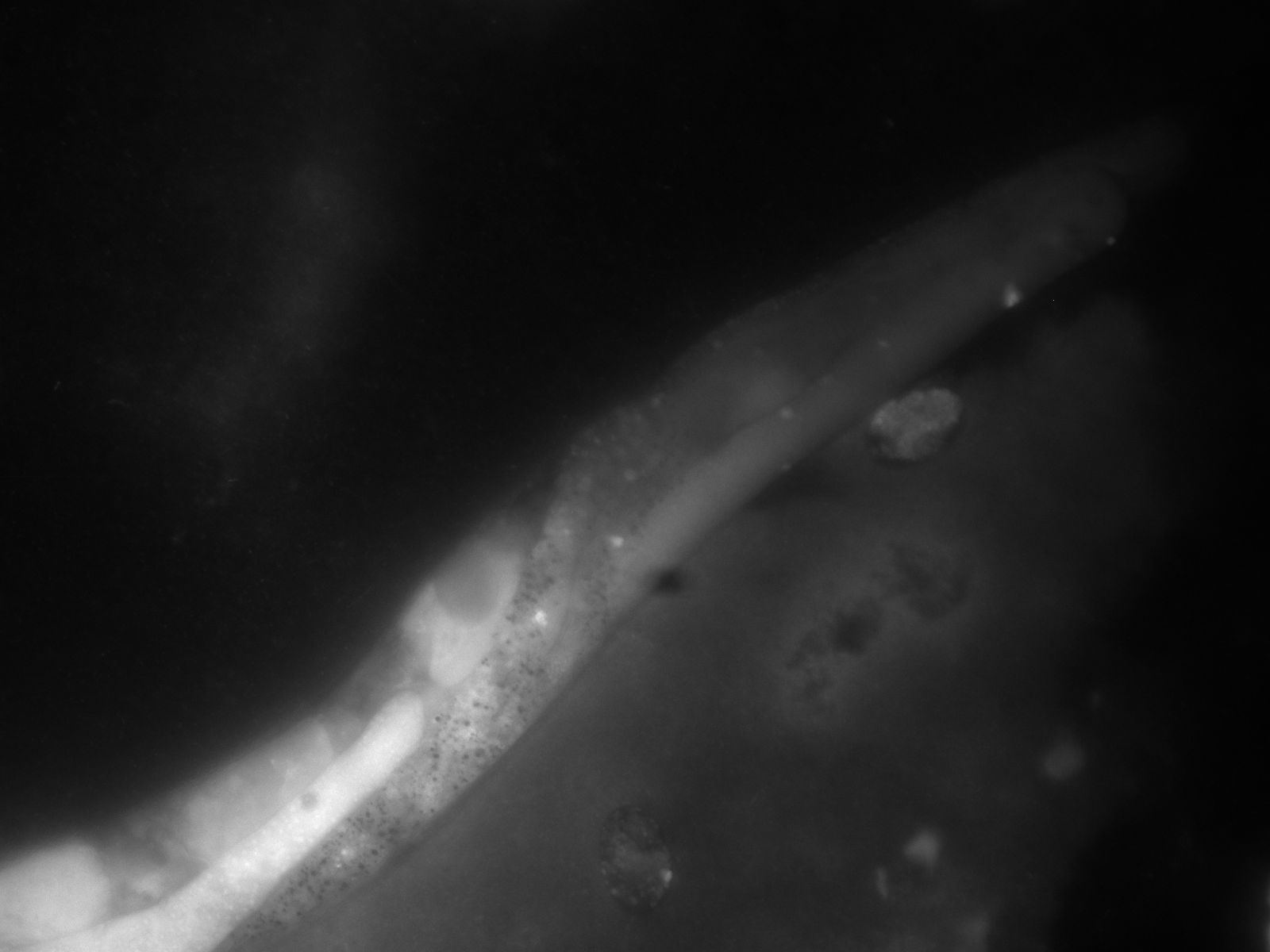

Supplement: S1 File — This file contains all the scoring data of the revised SYTO12 staining experiments. Each of the three biological replicates for Figs 2A, 4D, S2 and S4B–S4C were done in parallel in all strains. Hence, the wild type animals in Fig 2A and in S2 Fig are the same. In most cases animals were scored by live imaging without accompanied image acquisition. Representative images are provided. Consecutive images may image the same gonad. The scoring of apoptotic corpses was performed per gonad, not per image. (ZIP) [file pgen.1011061.s001.zip › SYTO staining experiment united/syto12 staining - 1_rep - 14.5.23 - JPEG/n2+tfg-1_194.jpg]

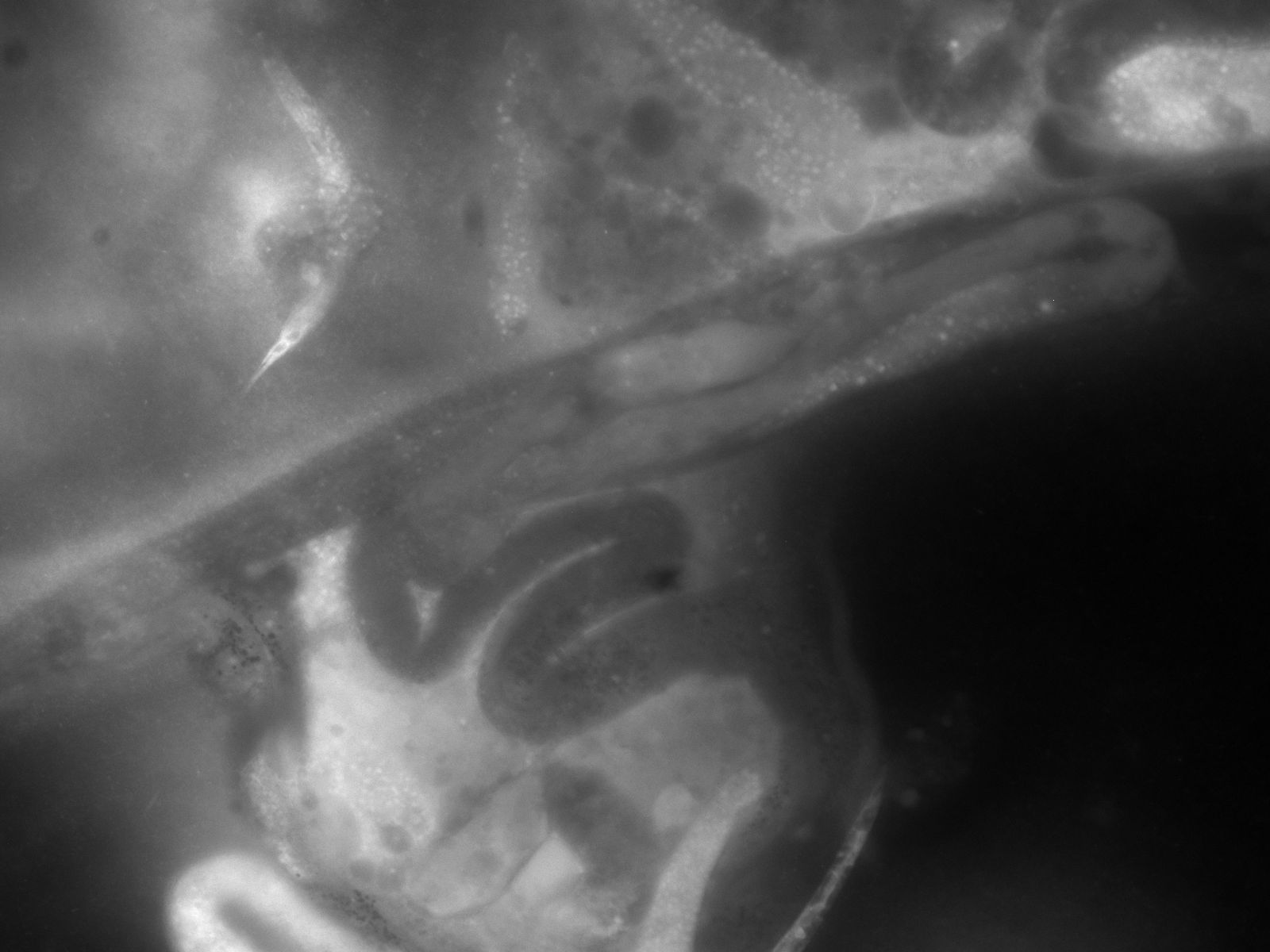

Supplement: S1 File — This file contains all the scoring data of the revised SYTO12 staining experiments. Each of the three biological replicates for Figs 2A, 4D, S2 and S4B–S4C were done in parallel in all strains. Hence, the wild type animals in Fig 2A and in S2 Fig are the same. In most cases animals were scored by live imaging without accompanied image acquisition. Representative images are provided. Consecutive images may image the same gonad. The scoring of apoptotic corpses was performed per gonad, not per image. (ZIP) [file pgen.1011061.s001.zip › SYTO staining experiment united/syto12 staining - 1_rep - 14.5.23 - JPEG/n2+tfg-1_195.jpg]

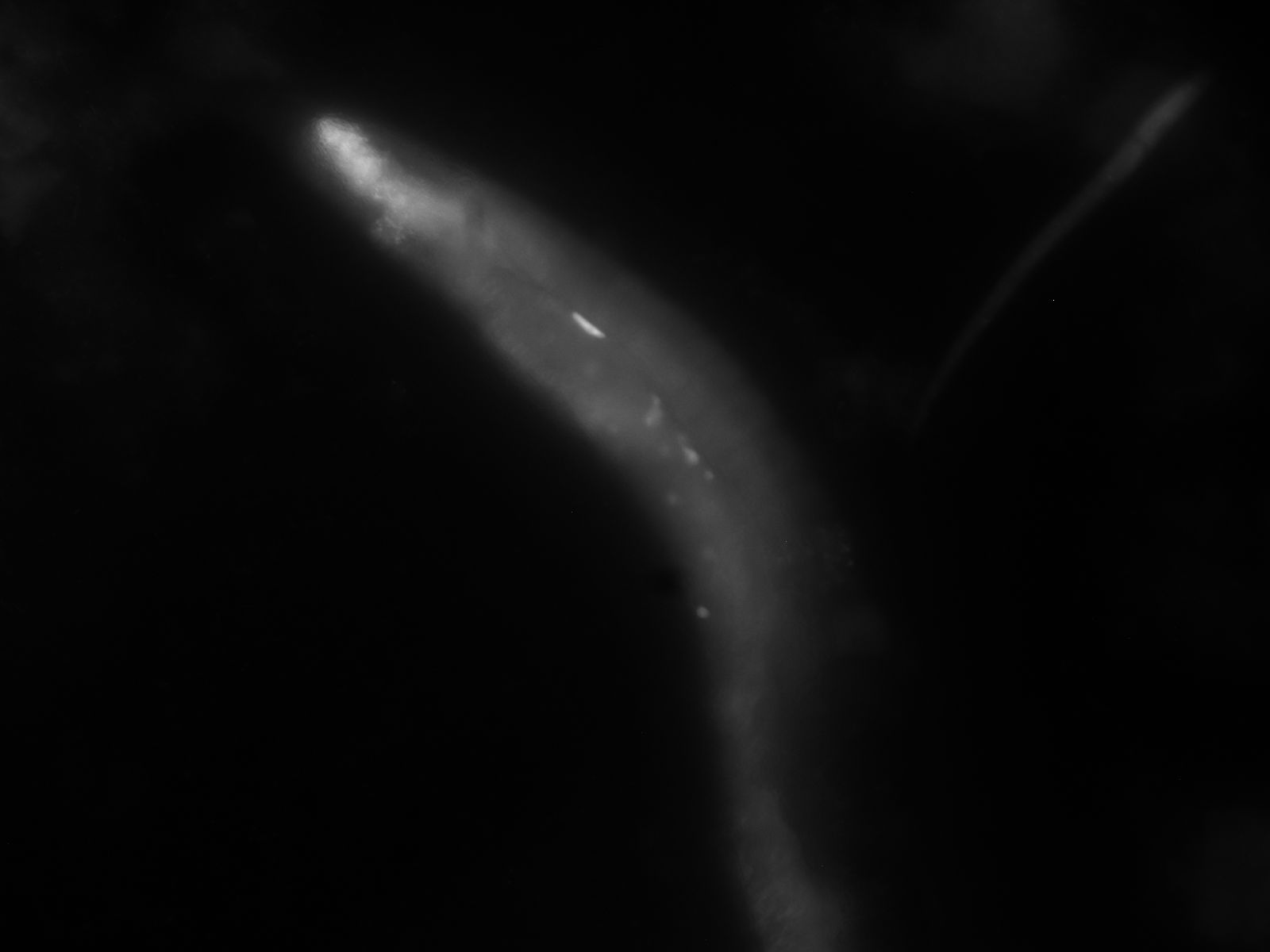

Supplement: S1 File — This file contains all the scoring data of the revised SYTO12 staining experiments. Each of the three biological replicates for Figs 2A, 4D, S2 and S4B–S4C were done in parallel in all strains. Hence, the wild type animals in Fig 2A and in S2 Fig are the same. In most cases animals were scored by live imaging without accompanied image acquisition. Representative images are provided. Consecutive images may image the same gonad. The scoring of apoptotic corpses was performed per gonad, not per image. (ZIP) [file pgen.1011061.s001.zip › SYTO staining experiment united/syto12 staining - 1_rep - 14.5.23 - JPEG/n2+tfg100.jpg]

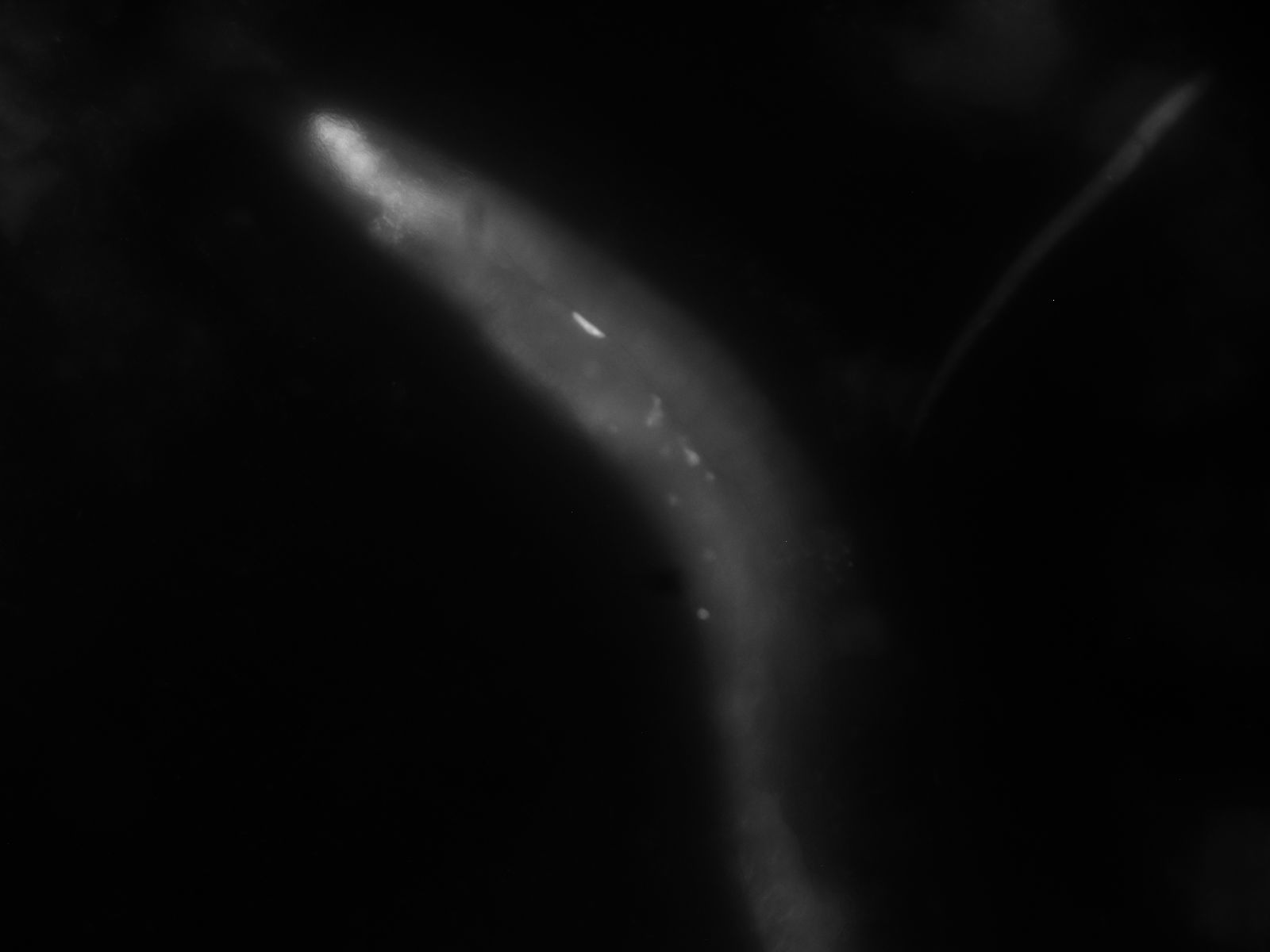

Supplement: S1 File — This file contains all the scoring data of the revised SYTO12 staining experiments. Each of the three biological replicates for Figs 2A, 4D, S2 and S4B–S4C were done in parallel in all strains. Hence, the wild type animals in Fig 2A and in S2 Fig are the same. In most cases animals were scored by live imaging without accompanied image acquisition. Representative images are provided. Consecutive images may image the same gonad. The scoring of apoptotic corpses was performed per gonad, not per image. (ZIP) [file pgen.1011061.s001.zip › SYTO staining experiment united/syto12 staining - 1_rep - 14.5.23 - JPEG/n2+tfg101.jpg]

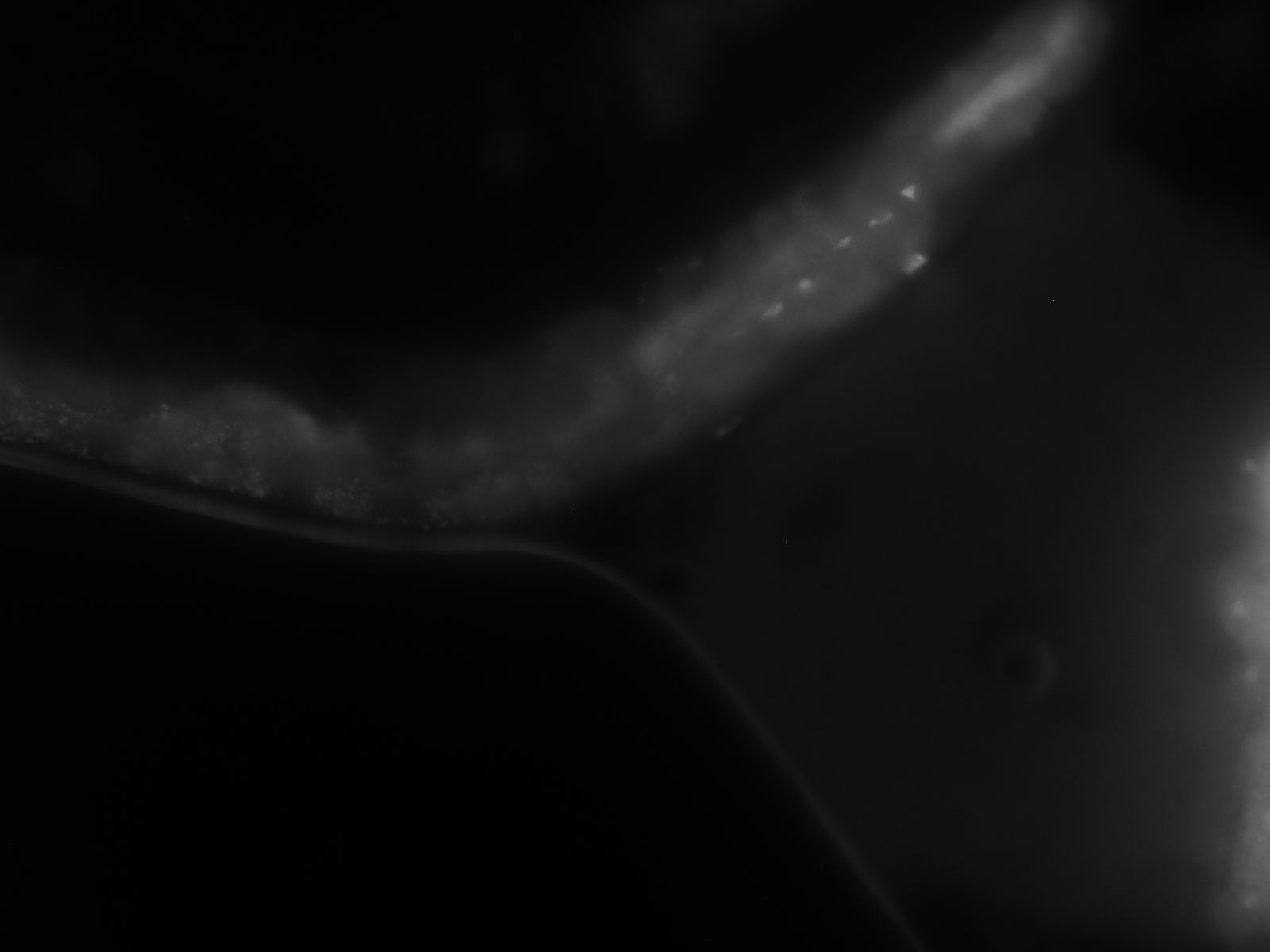

Supplement: S1 File — This file contains all the scoring data of the revised SYTO12 staining experiments. Each of the three biological replicates for Figs 2A, 4D, S2 and S4B–S4C were done in parallel in all strains. Hence, the wild type animals in Fig 2A and in S2 Fig are the same. In most cases animals were scored by live imaging without accompanied image acquisition. Representative images are provided. Consecutive images may image the same gonad. The scoring of apoptotic corpses was performed per gonad, not per image. (ZIP) [file pgen.1011061.s001.zip › SYTO staining experiment united/syto12 staining - 1_rep - 14.5.23 - JPEG/n2+tfg102.jpg]

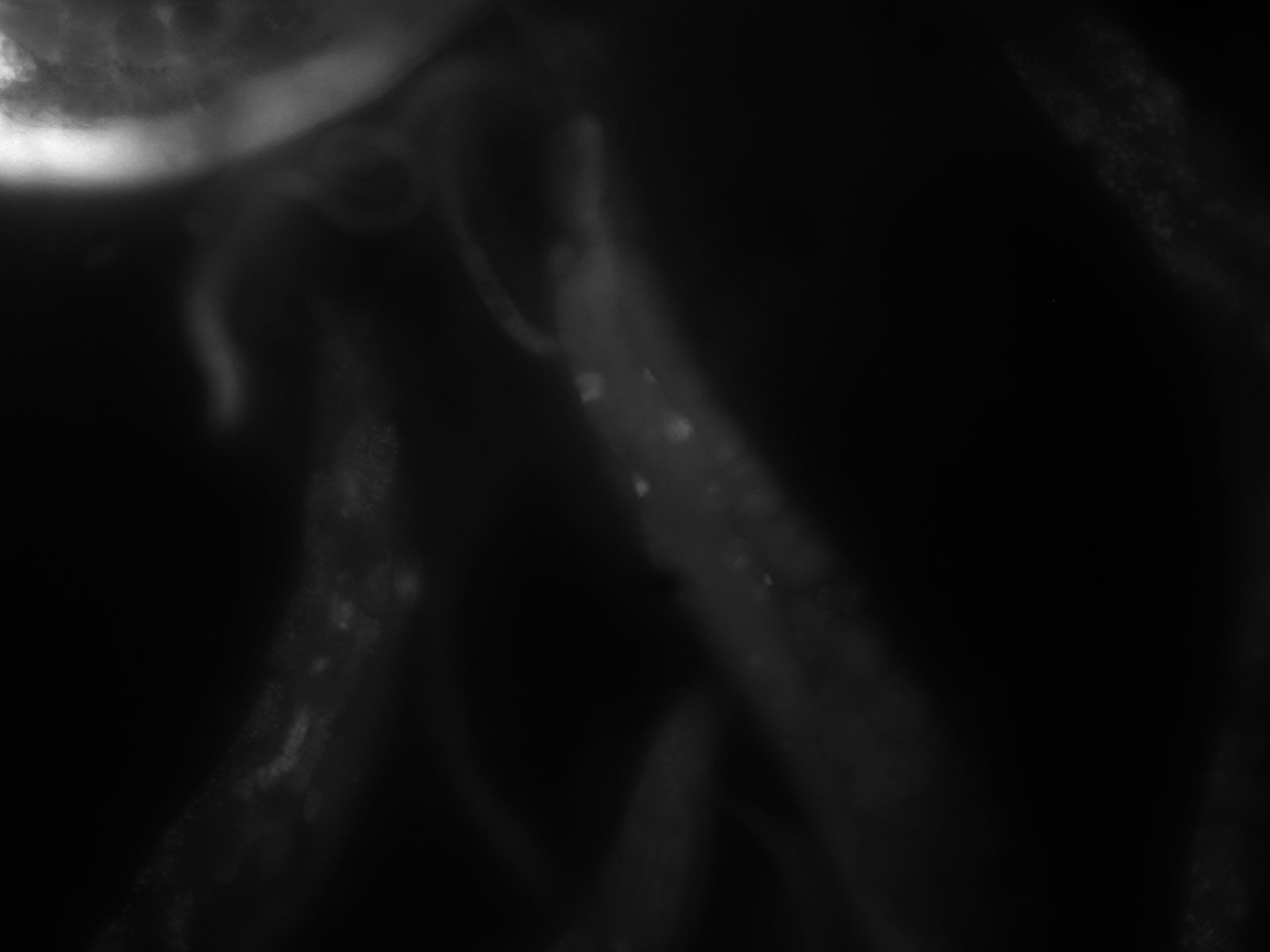

Supplement: S1 File — This file contains all the scoring data of the revised SYTO12 staining experiments. Each of the three biological replicates for Figs 2A, 4D, S2 and S4B–S4C were done in parallel in all strains. Hence, the wild type animals in Fig 2A and in S2 Fig are the same. In most cases animals were scored by live imaging without accompanied image acquisition. Representative images are provided. Consecutive images may image the same gonad. The scoring of apoptotic corpses was performed per gonad, not per image. (ZIP) [file pgen.1011061.s001.zip › SYTO staining experiment united/syto12 staining - 1_rep - 14.5.23 - JPEG/n2+tfg103.jpg]

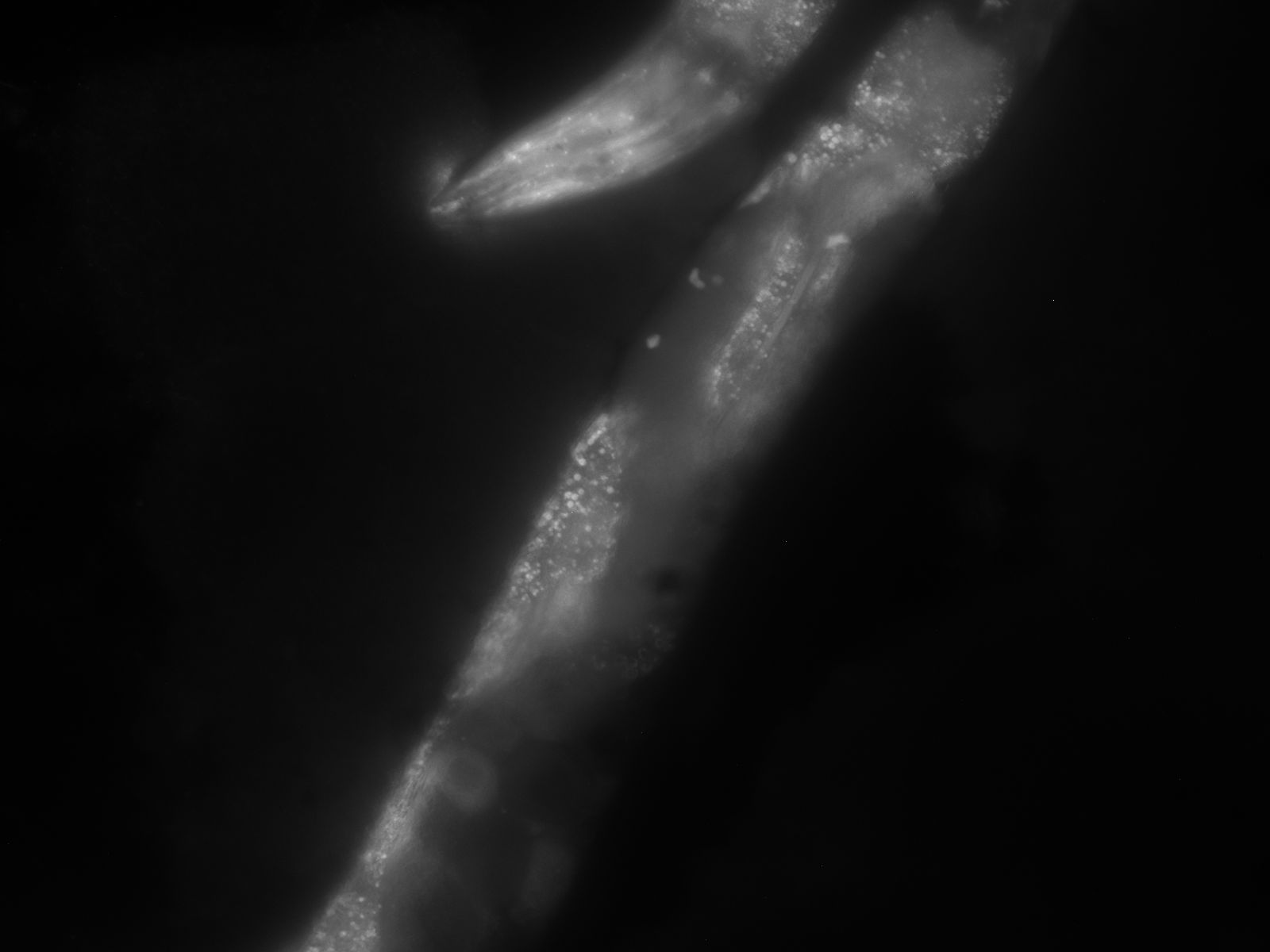

Supplement: S1 File — This file contains all the scoring data of the revised SYTO12 staining experiments. Each of the three biological replicates for Figs 2A, 4D, S2 and S4B–S4C were done in parallel in all strains. Hence, the wild type animals in Fig 2A and in S2 Fig are the same. In most cases animals were scored by live imaging without accompanied image acquisition. Representative images are provided. Consecutive images may image the same gonad. The scoring of apoptotic corpses was performed per gonad, not per image. (ZIP) [file pgen.1011061.s001.zip › SYTO staining experiment united/syto12 staining - 1_rep - 14.5.23 - JPEG/n2+tfg104.jpg]

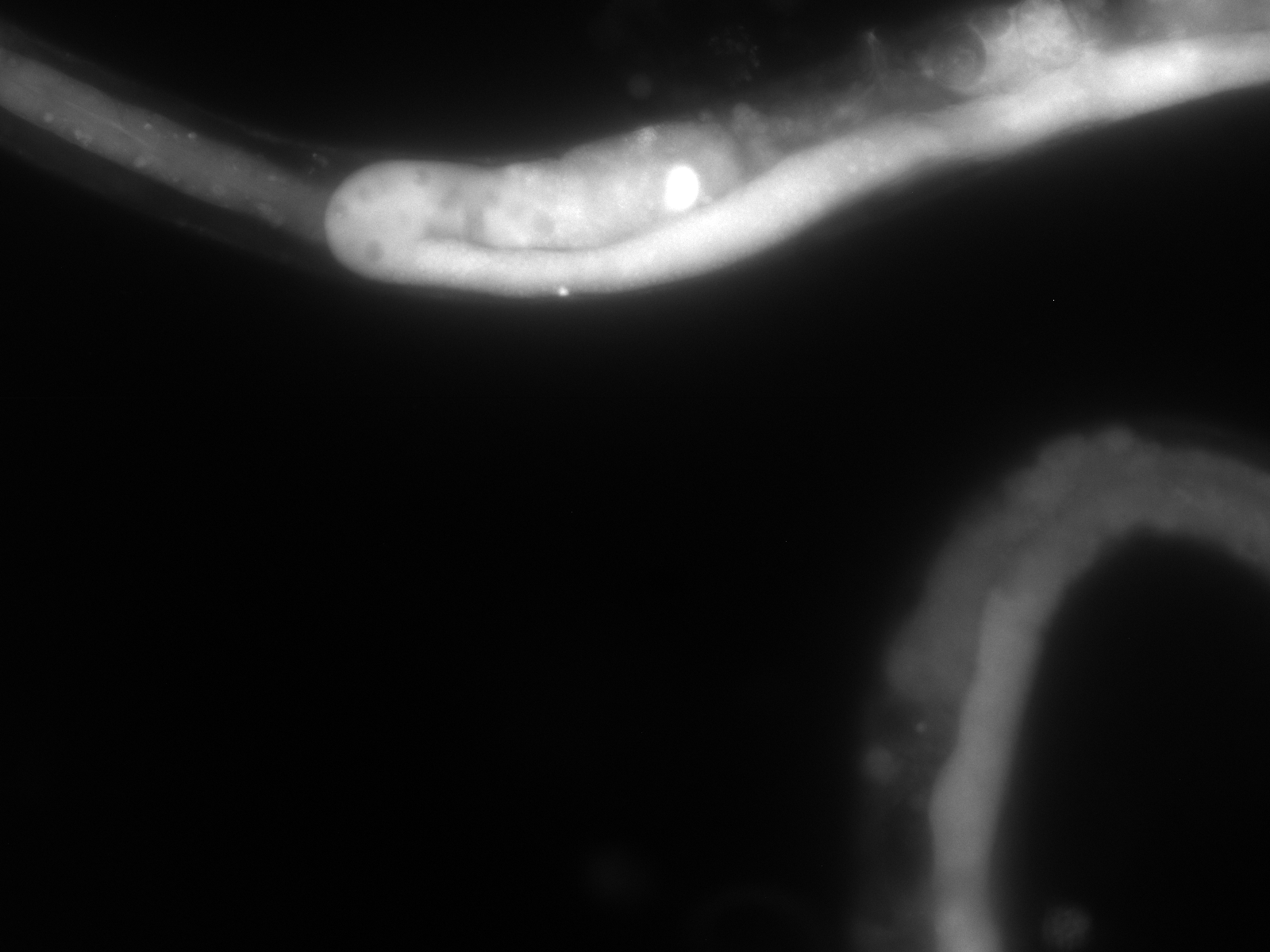

Supplement: S2 File — (ZIP) [file pgen.1011061.s002.zip › Fig.2A - Original files/Fig.2A pictures selected for figure 2A/ire-1+pad12119.tif]
