# Supplementary figures and images for: Correction: It’s All in Your Mind: Determining Germ Cell Fate by Neuronal IRE-1 in C. elegans (part 4 of 7)
Source: PLoS Genet. 2023 Nov 30;19(11):e1011061. doi: 10.1371/journal.pgen.1011061 (PMC10688620; doi:10.1371/journal.pgen.1011061)

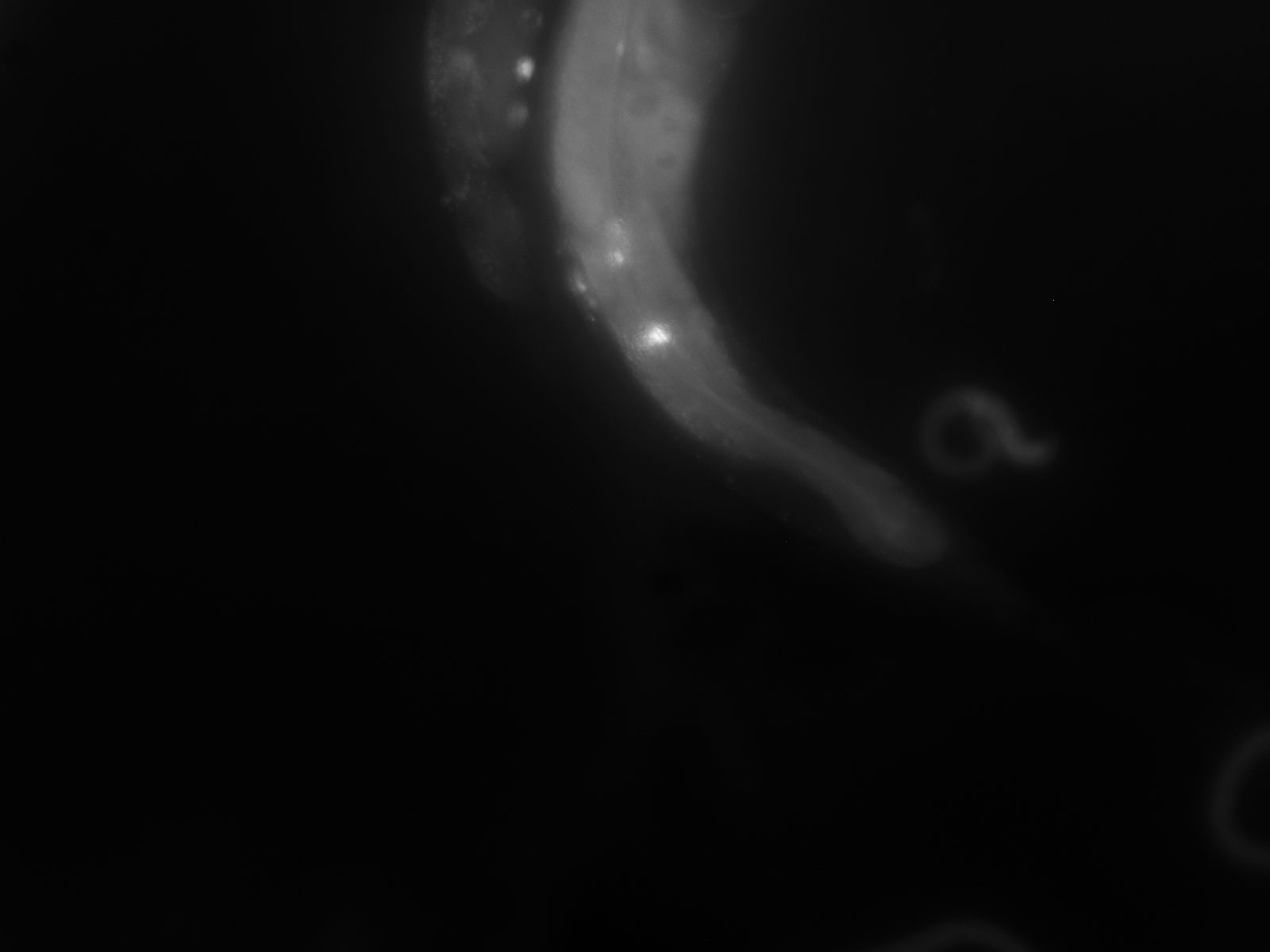

Supplement: S2 File — (ZIP) [file pgen.1011061.s002.zip › Fig.2A - Original files/Fig.2A RAW data and photos JPEG/syto12 staining - fig 2A - 1_rep - 14.5.23 jpeg/xbp-1+pad12101.jpg]

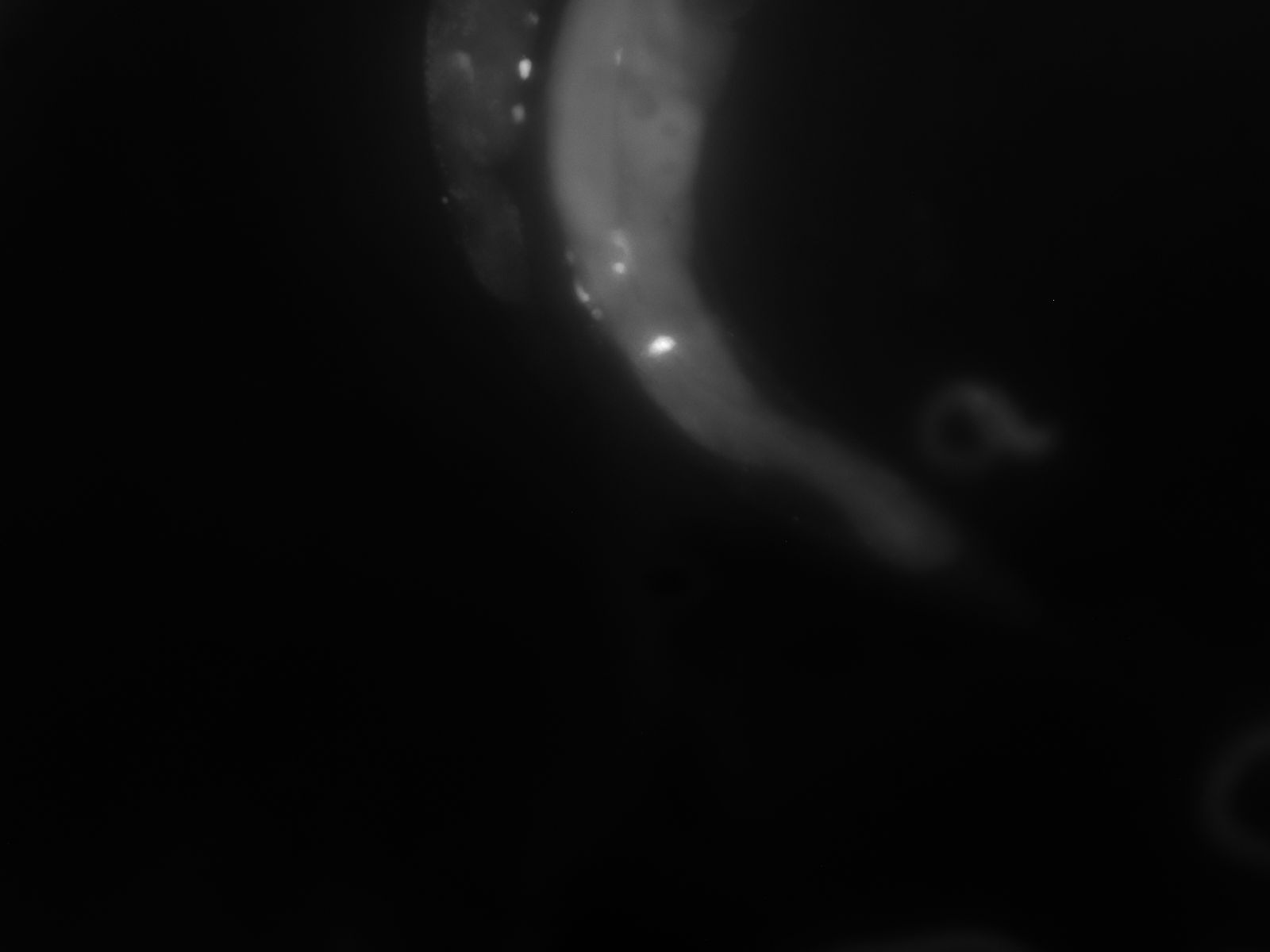

Supplement: S2 File — (ZIP) [file pgen.1011061.s002.zip › Fig.2A - Original files/Fig.2A RAW data and photos JPEG/syto12 staining - fig 2A - 1_rep - 14.5.23 jpeg/xbp-1+pad12102.jpg]

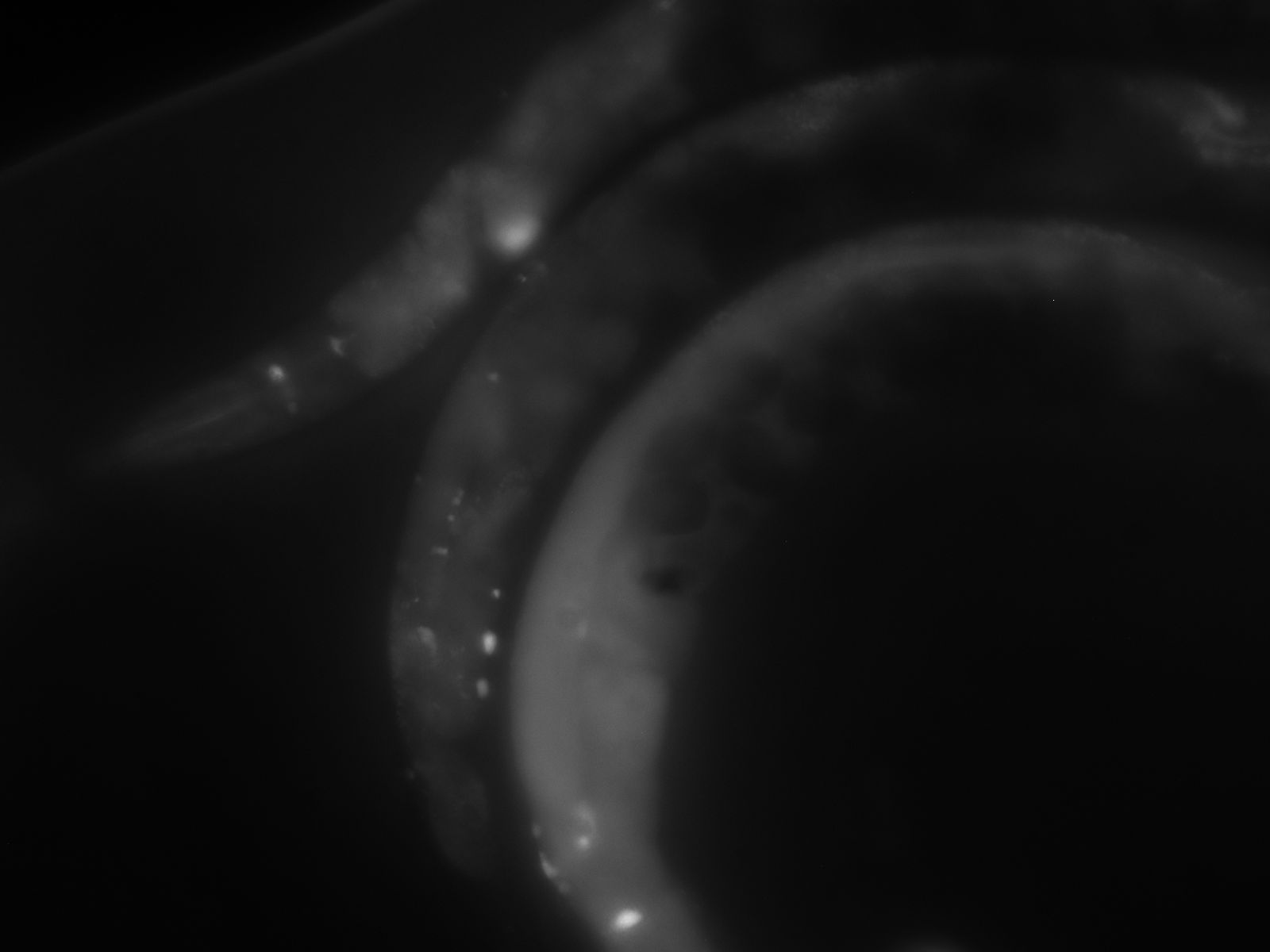

Supplement: S2 File — (ZIP) [file pgen.1011061.s002.zip › Fig.2A - Original files/Fig.2A RAW data and photos JPEG/syto12 staining - fig 2A - 1_rep - 14.5.23 jpeg/xbp-1+pad12103.jpg]

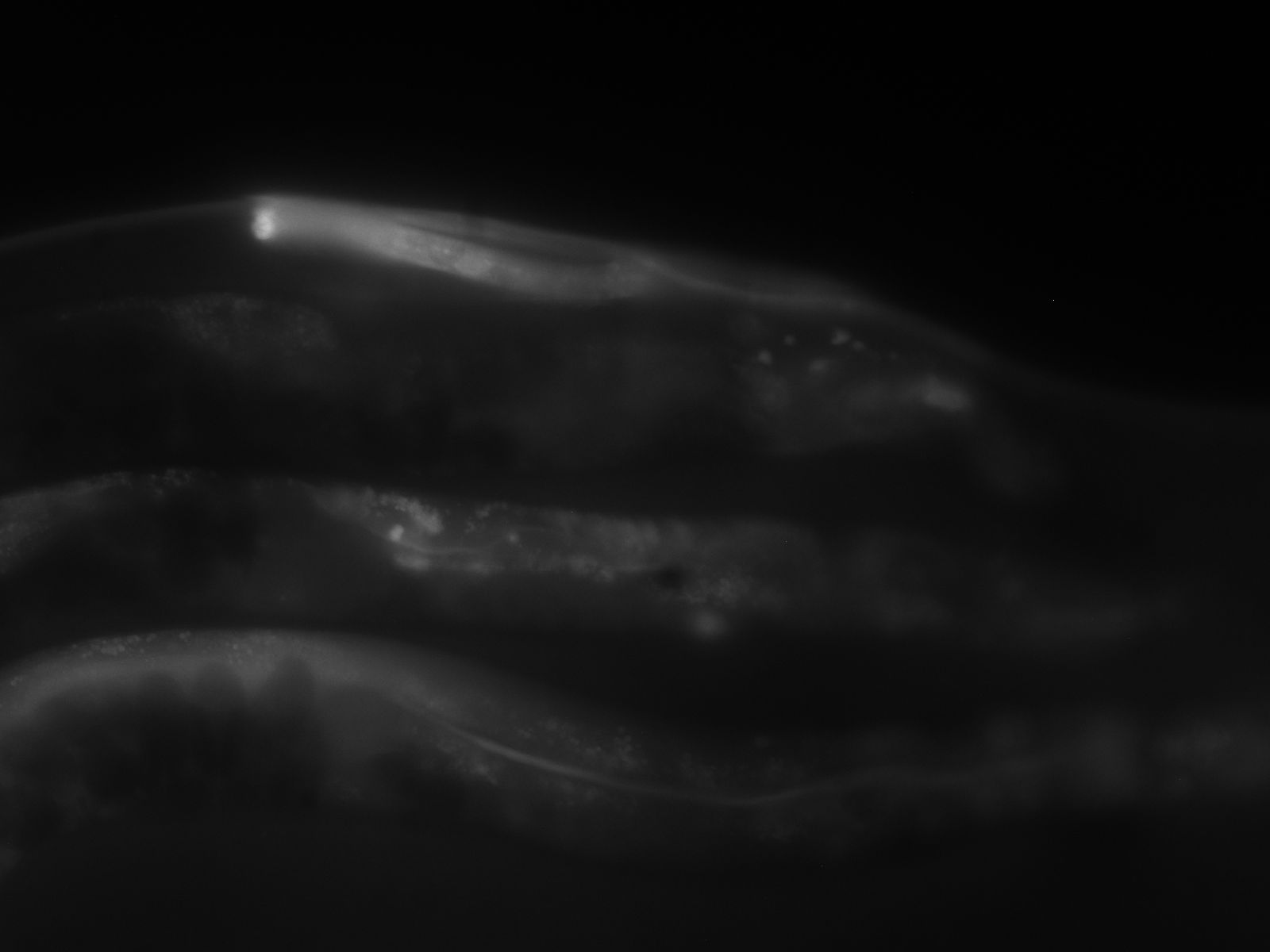

Supplement: S2 File — (ZIP) [file pgen.1011061.s002.zip › Fig.2A - Original files/Fig.2A RAW data and photos JPEG/syto12 staining - fig 2A - 1_rep - 14.5.23 jpeg/xbp-1+pad12104.jpg]

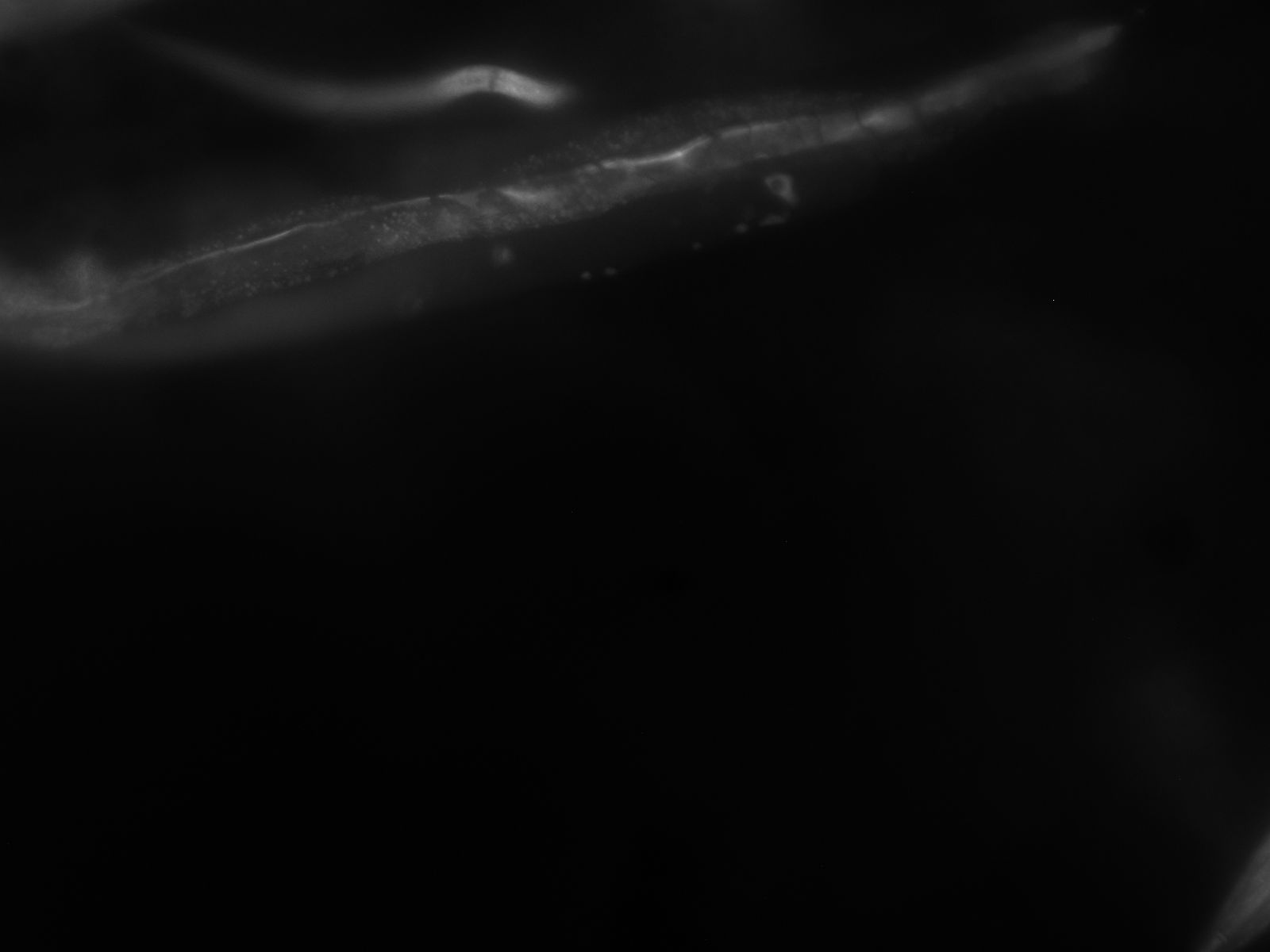

Supplement: S2 File — (ZIP) [file pgen.1011061.s002.zip › Fig.2A - Original files/Fig.2A RAW data and photos JPEG/syto12 staining - fig 2A - 1_rep - 14.5.23 jpeg/xbp-1+pad12105.jpg]

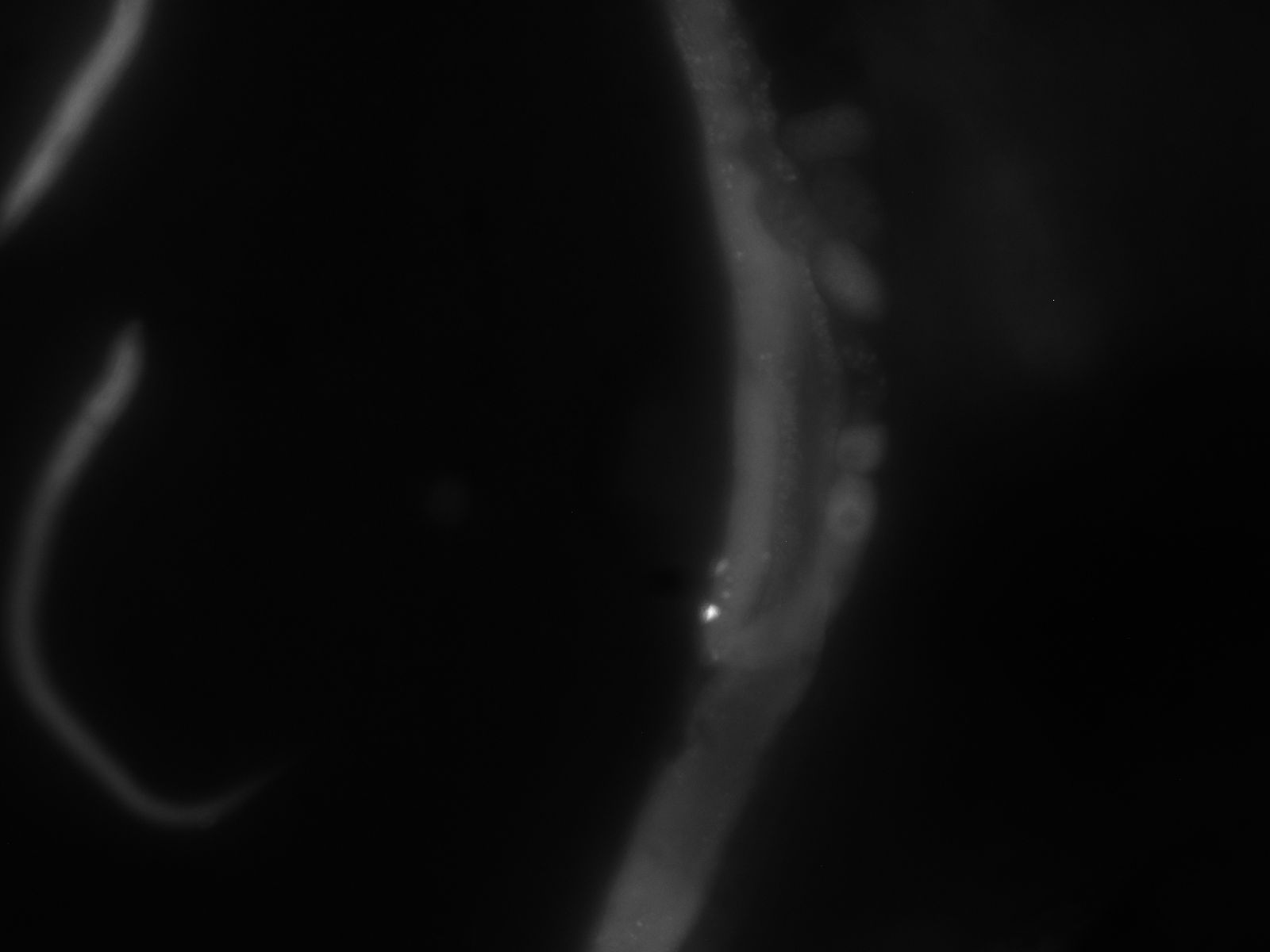

Supplement: S2 File — (ZIP) [file pgen.1011061.s002.zip › Fig.2A - Original files/Fig.2A RAW data and photos JPEG/syto12 staining - fig 2A - 1_rep - 14.5.23 jpeg/xbp-1+pad12106.jpg]

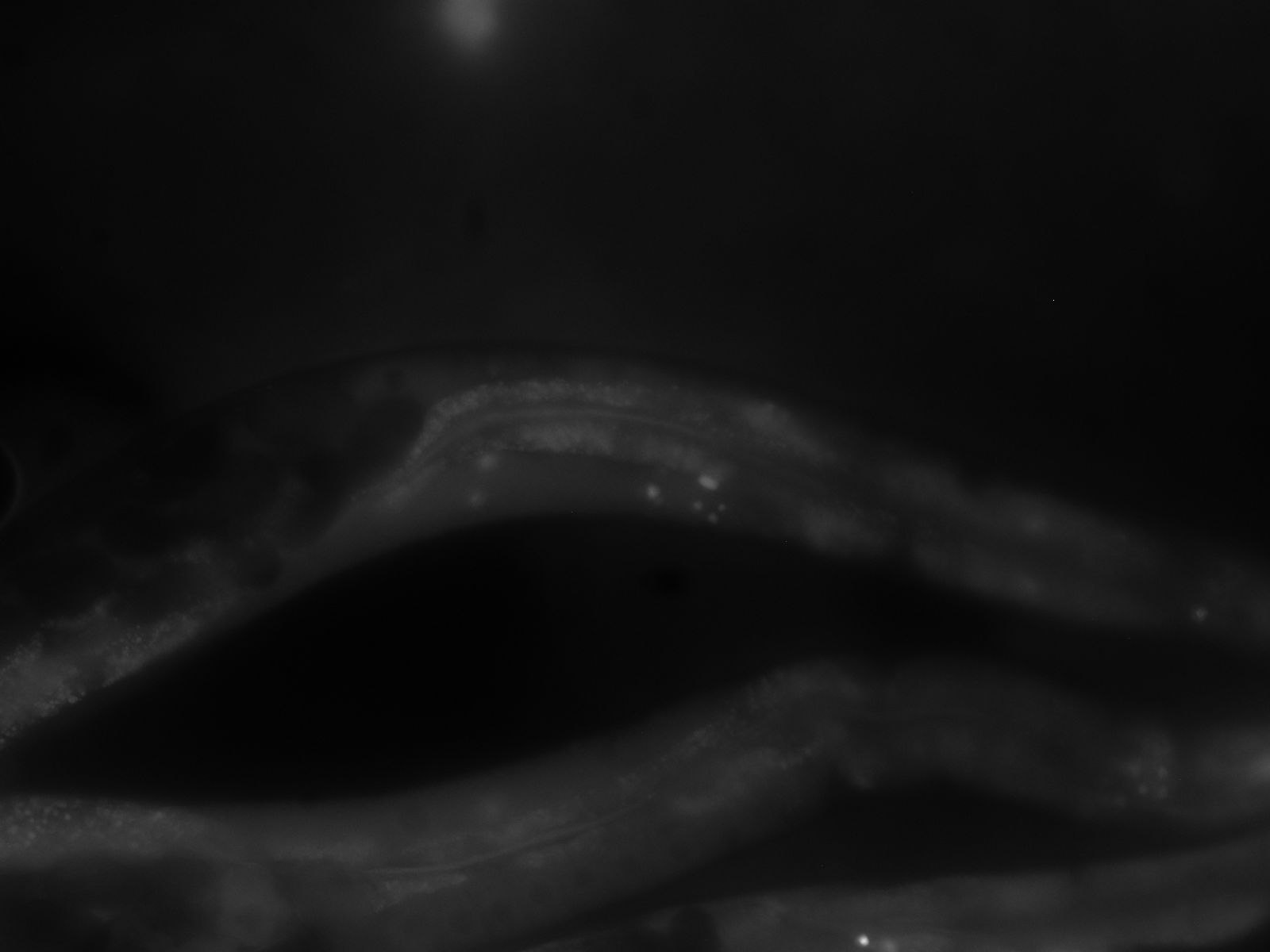

Supplement: S2 File — (ZIP) [file pgen.1011061.s002.zip › Fig.2A - Original files/Fig.2A RAW data and photos JPEG/syto12 staining - fig 2A - 1_rep - 14.5.23 jpeg/xbp-1+pad12107.jpg]

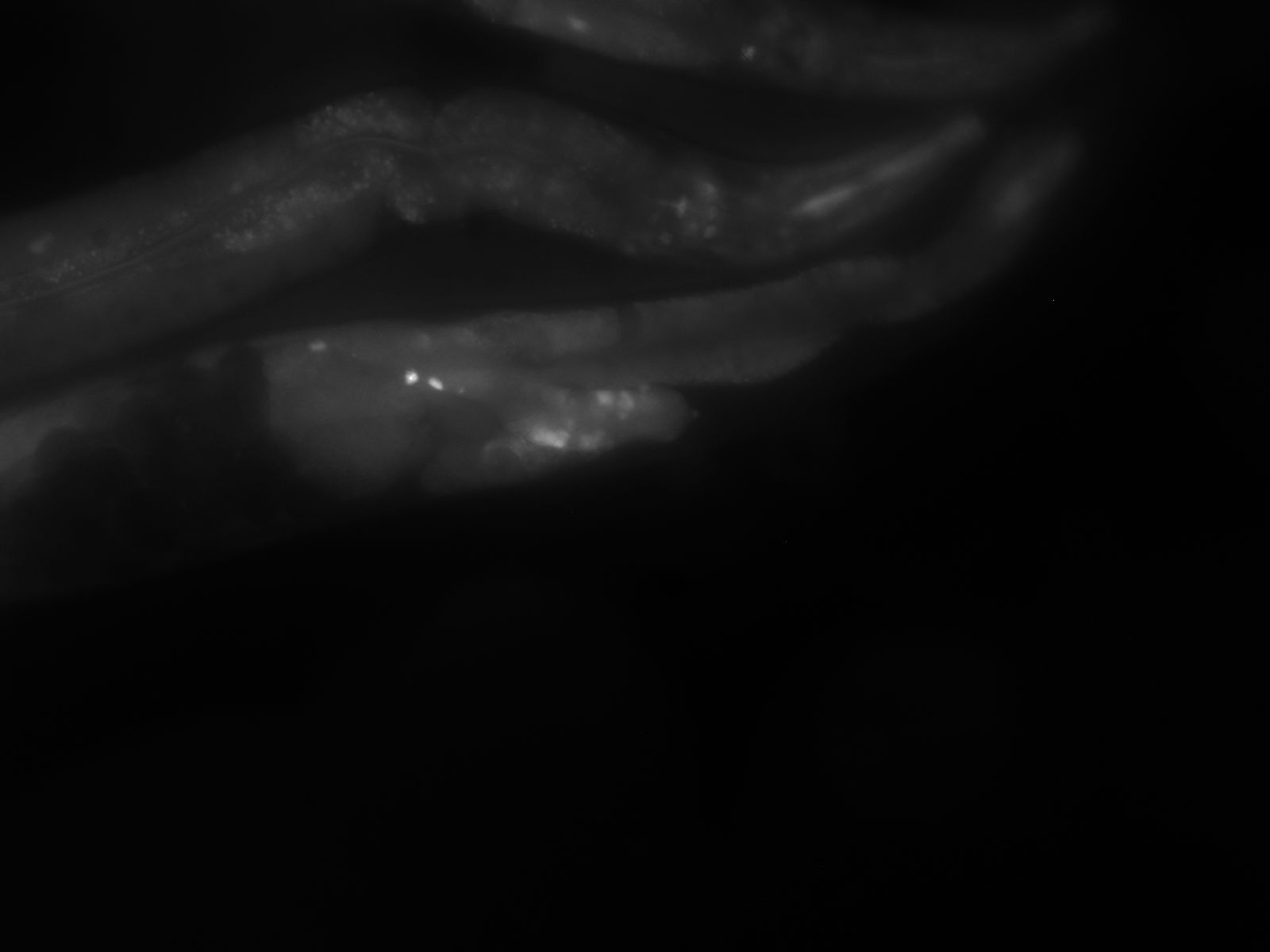

Supplement: S2 File — (ZIP) [file pgen.1011061.s002.zip › Fig.2A - Original files/Fig.2A RAW data and photos JPEG/syto12 staining - fig 2A - 1_rep - 14.5.23 jpeg/xbp-1+pad12108.jpg]

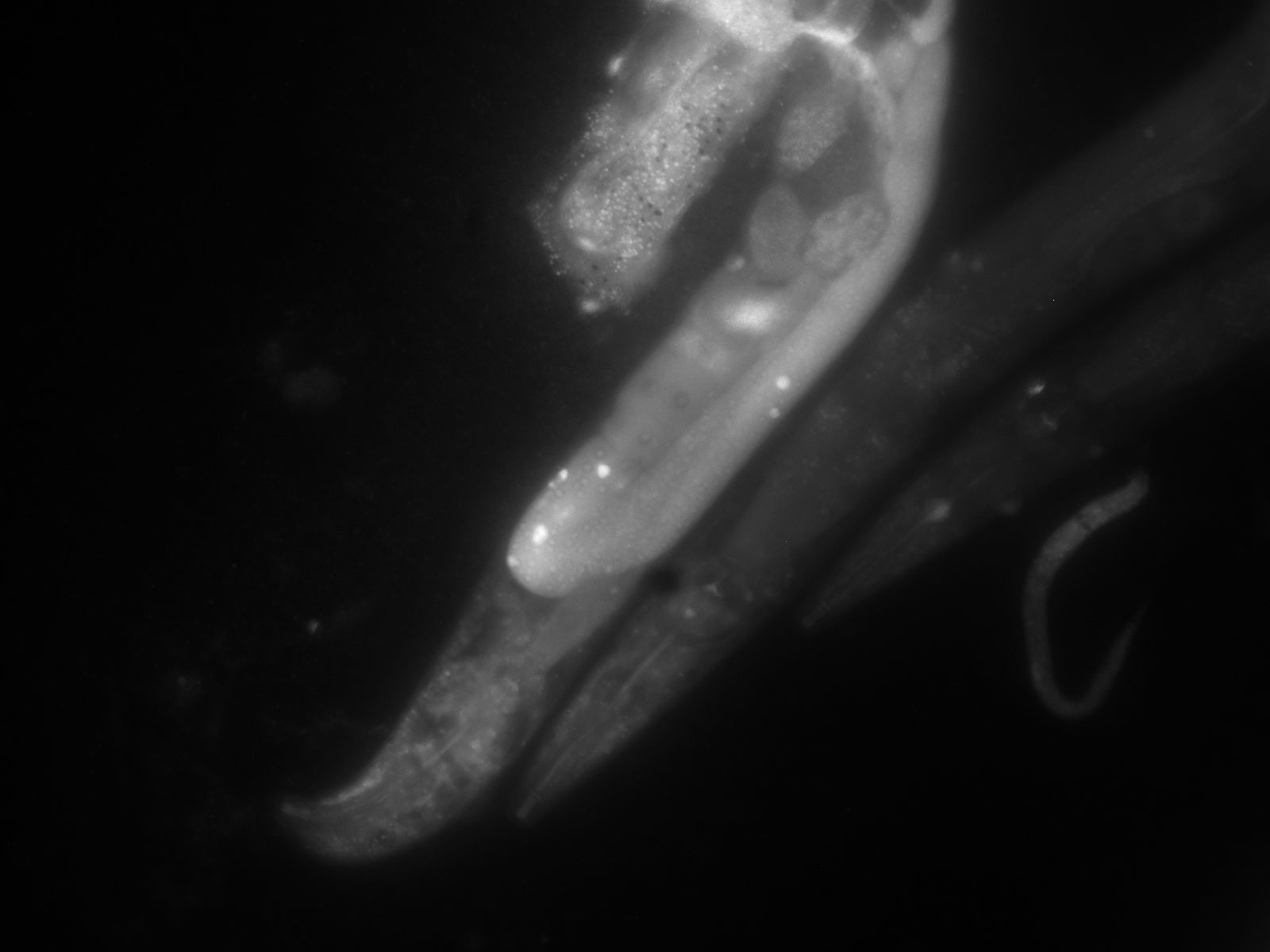

Supplement: S2 File — (ZIP) [file pgen.1011061.s002.zip › Fig.2A - Original files/Fig.2A RAW data and photos JPEG/syto12 staining - fig 2A - 1_rep - 14.5.23 jpeg/xbp-1+pad12109.jpg]

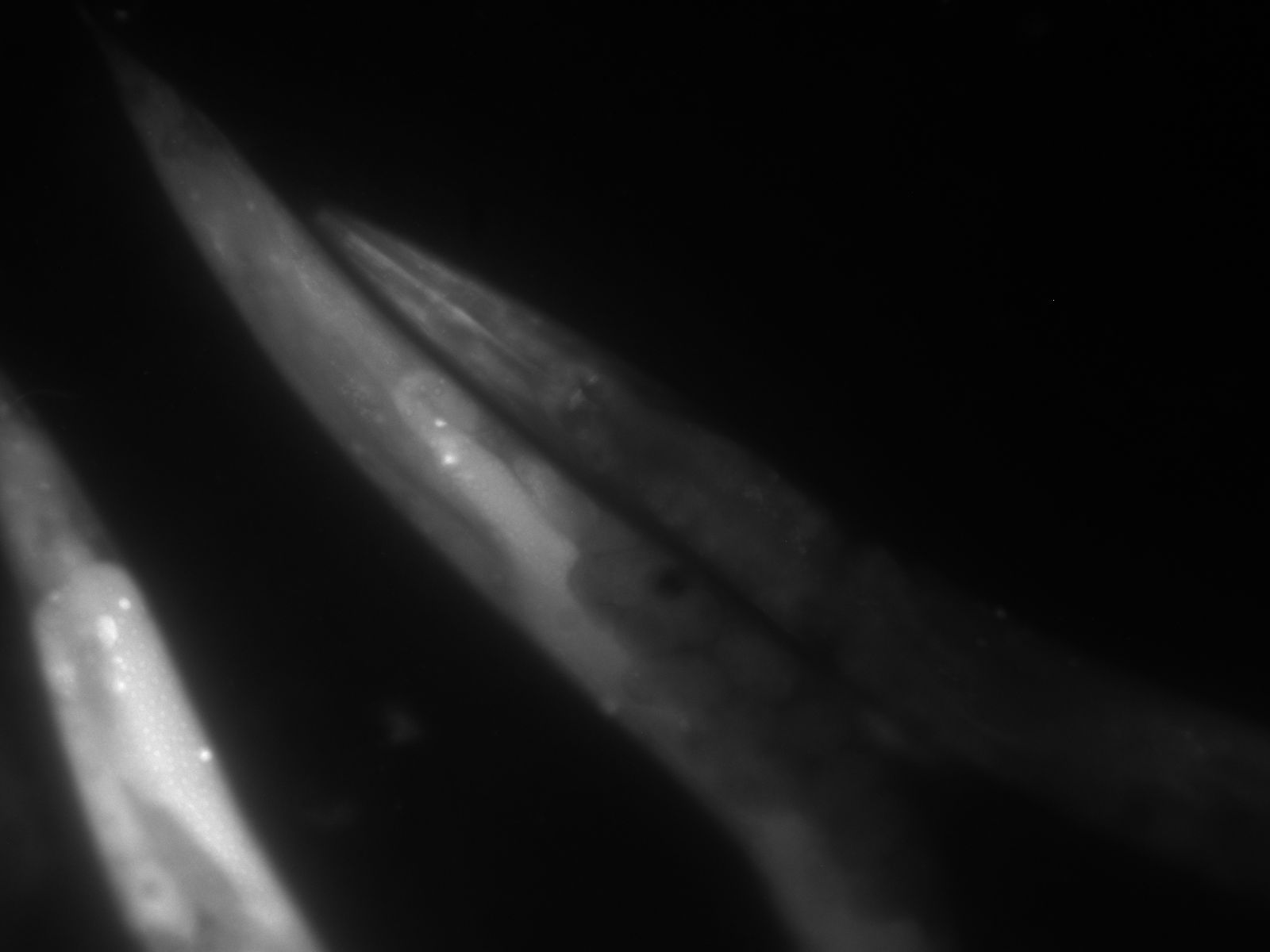

Supplement: S2 File — (ZIP) [file pgen.1011061.s002.zip › Fig.2A - Original files/Fig.2A RAW data and photos JPEG/syto12 staining - fig 2A - 1_rep - 14.5.23 jpeg/xbp-1+pad12110.jpg]

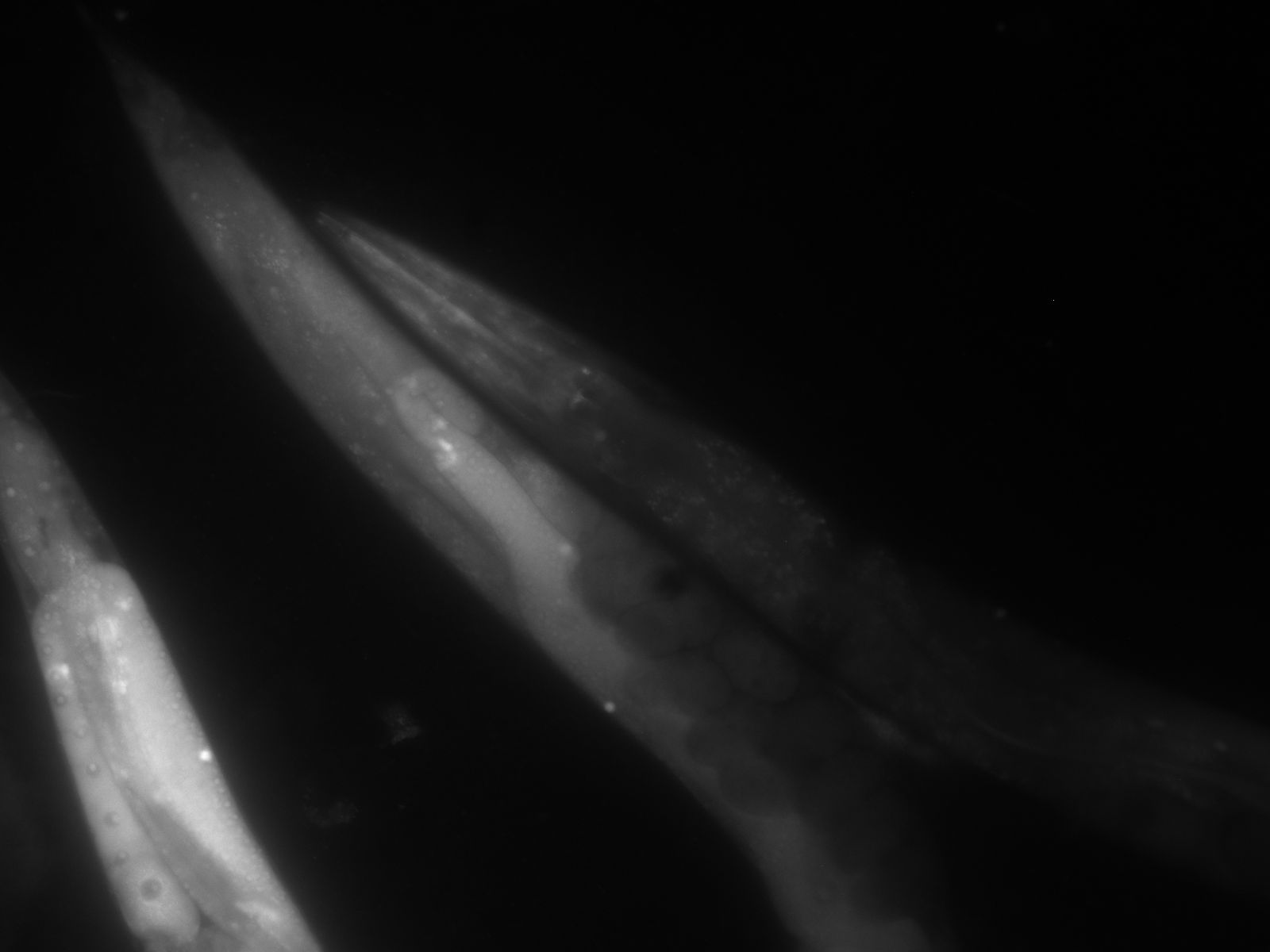

Supplement: S2 File — (ZIP) [file pgen.1011061.s002.zip › Fig.2A - Original files/Fig.2A RAW data and photos JPEG/syto12 staining - fig 2A - 1_rep - 14.5.23 jpeg/xbp-1+pad12111.jpg]

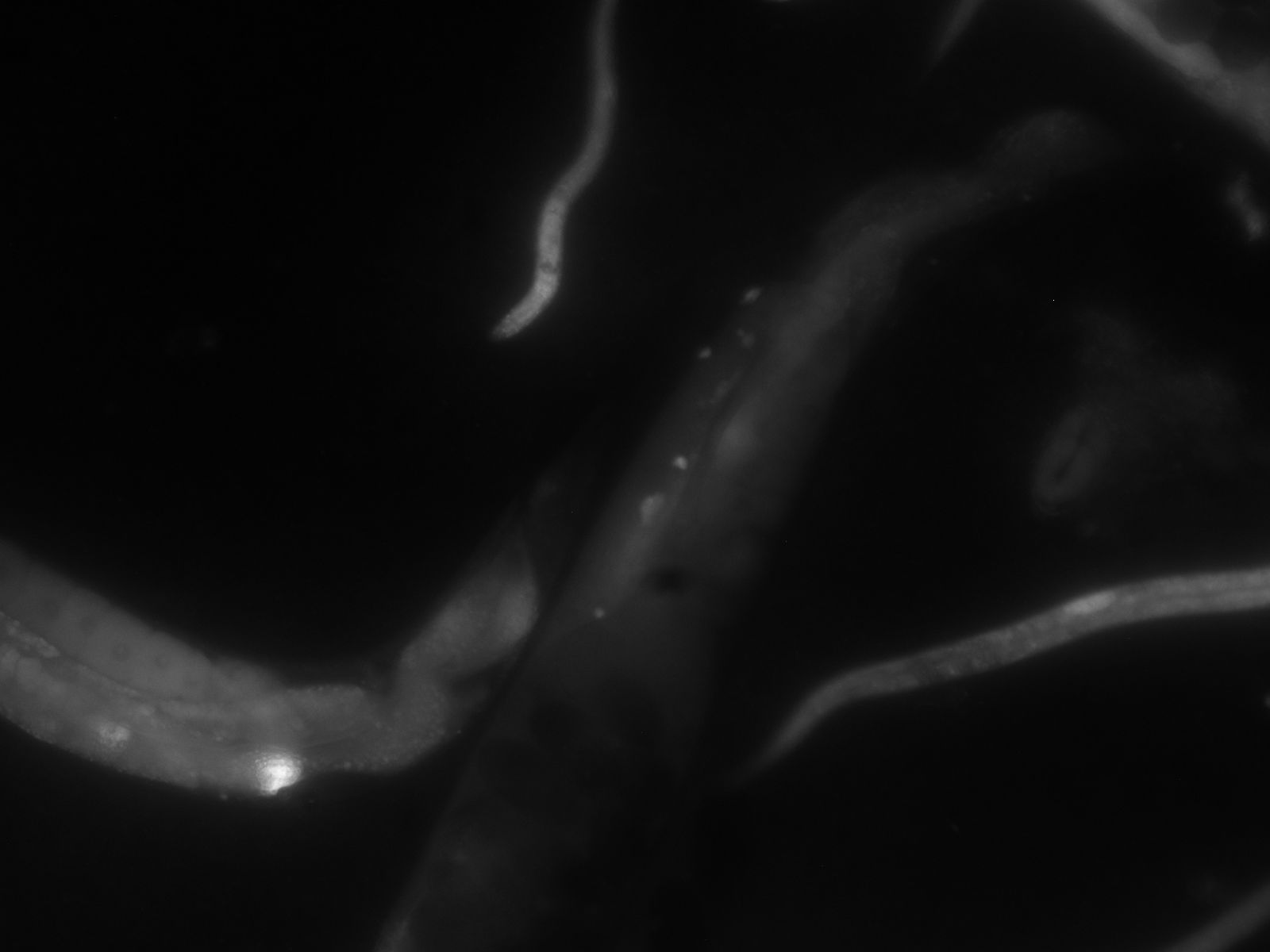

Supplement: S2 File — (ZIP) [file pgen.1011061.s002.zip › Fig.2A - Original files/Fig.2A RAW data and photos JPEG/syto12 staining - fig 2A - 1_rep - 14.5.23 jpeg/xbp-1+pad12112.jpg]

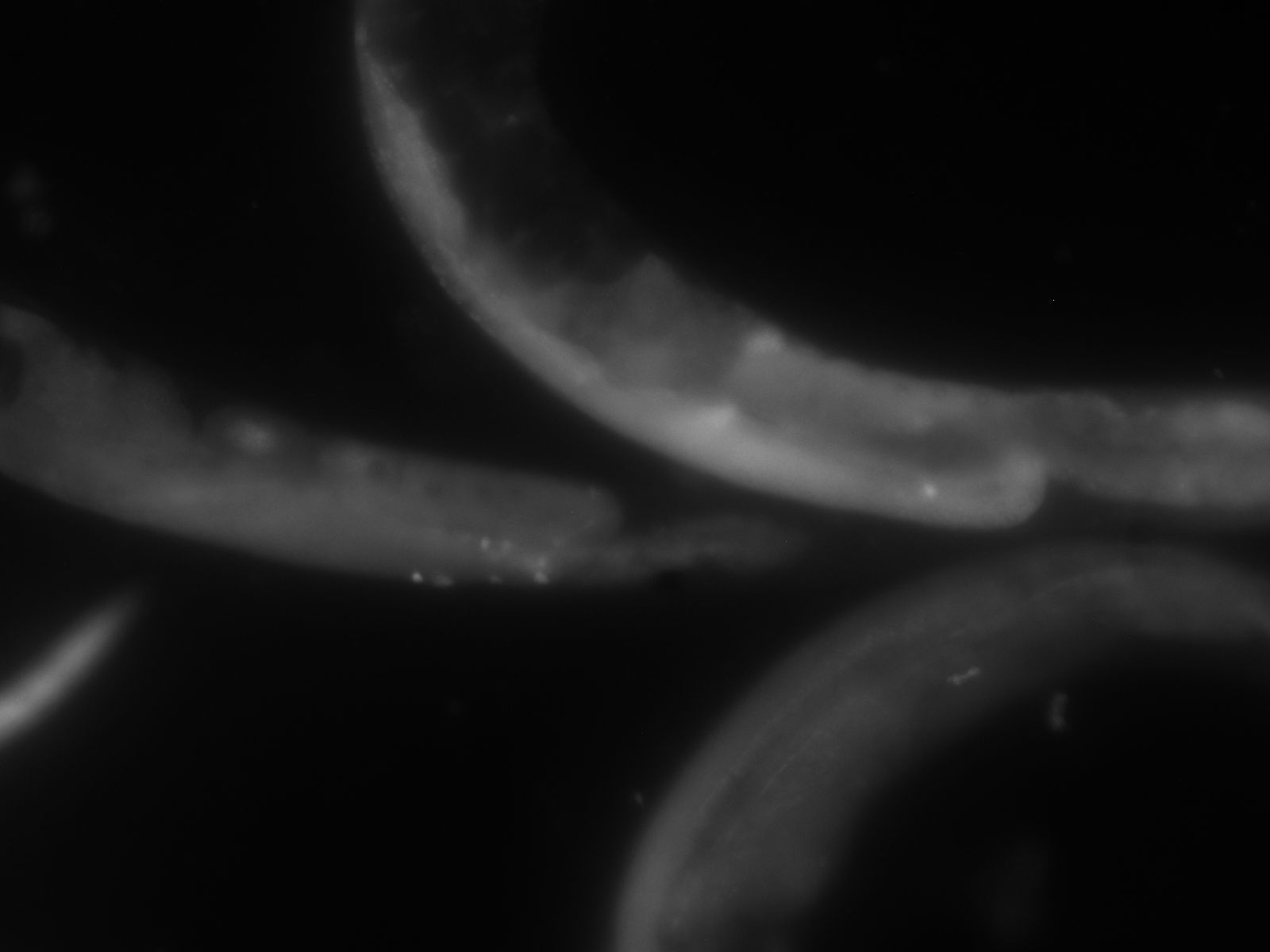

Supplement: S2 File — (ZIP) [file pgen.1011061.s002.zip › Fig.2A - Original files/Fig.2A RAW data and photos JPEG/syto12 staining - fig 2A - 1_rep - 14.5.23 jpeg/xbp-1+pad12113.jpg]

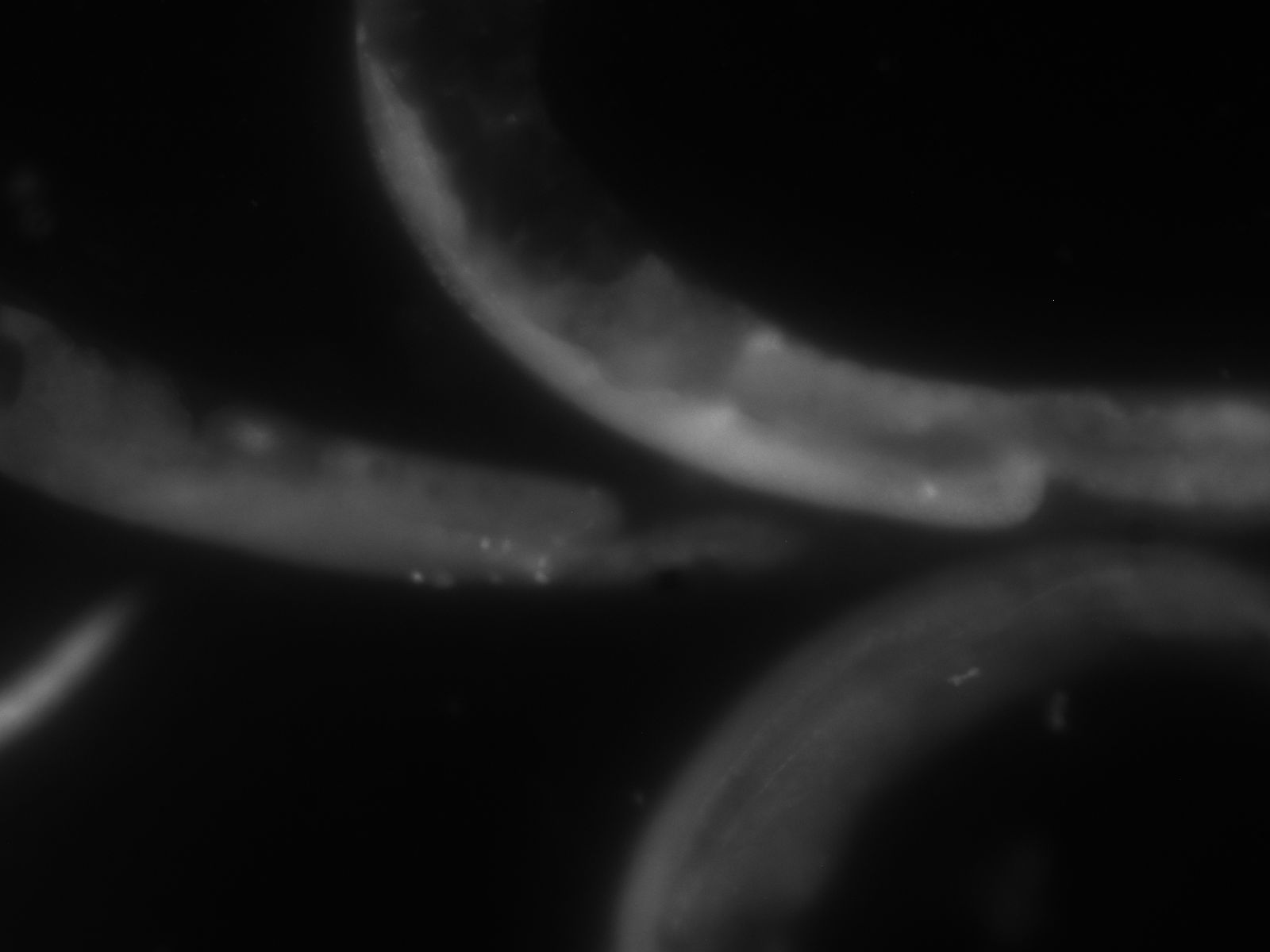

Supplement: S2 File — (ZIP) [file pgen.1011061.s002.zip › Fig.2A - Original files/Fig.2A RAW data and photos JPEG/syto12 staining - fig 2A - 1_rep - 14.5.23 jpeg/xbp-1+pad12114.jpg]

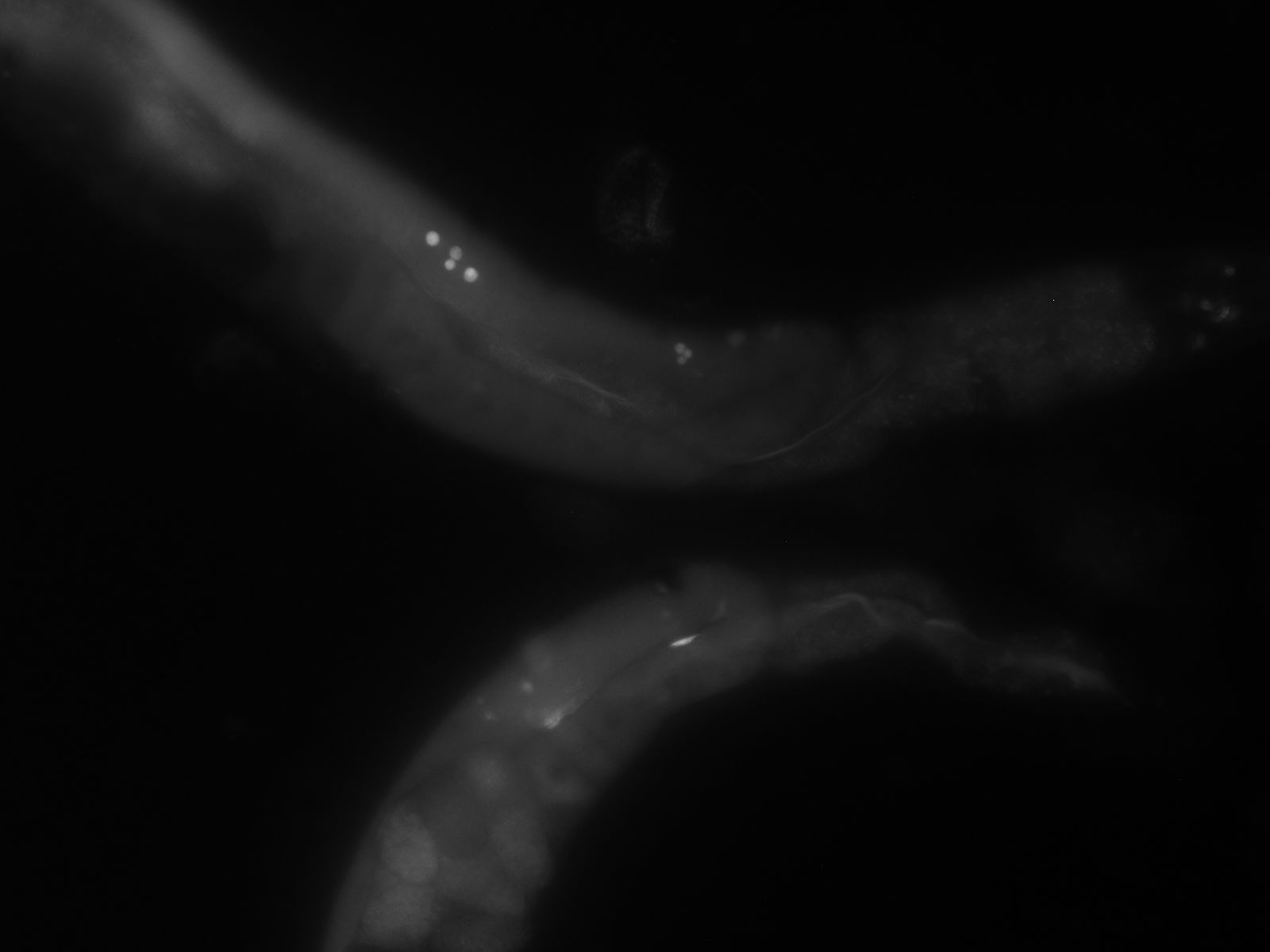

Supplement: S2 File — (ZIP) [file pgen.1011061.s002.zip › Fig.2A - Original files/Fig.2A RAW data and photos JPEG/syto12 staining - fig 2A - 1_rep - 14.5.23 jpeg/xbp-1+pad12115.jpg]

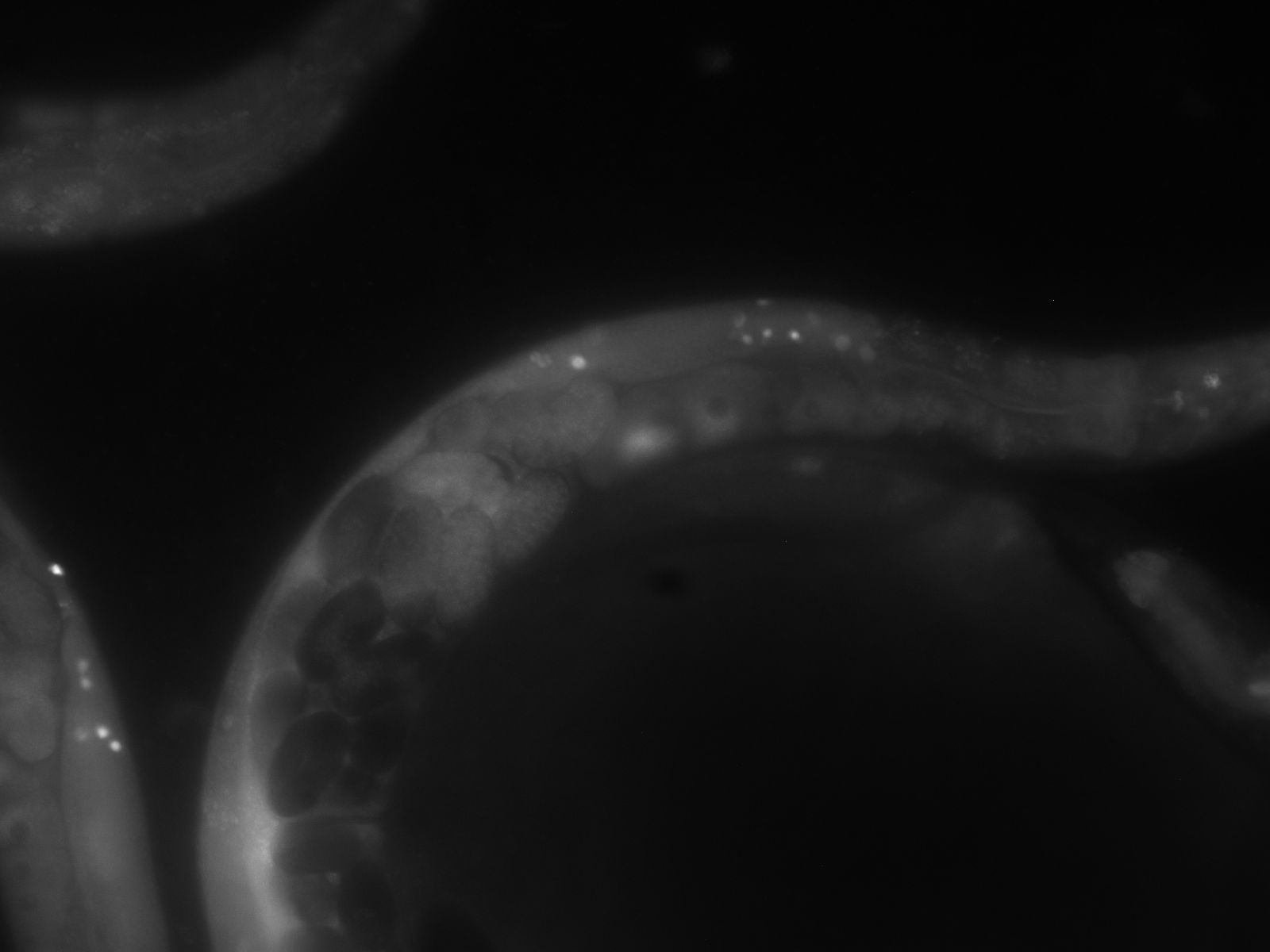

Supplement: S2 File — (ZIP) [file pgen.1011061.s002.zip › Fig.2A - Original files/Fig.2A RAW data and photos JPEG/syto12 staining - fig 2A - 1_rep - 14.5.23 jpeg/xbp-1+pad12116.jpg]

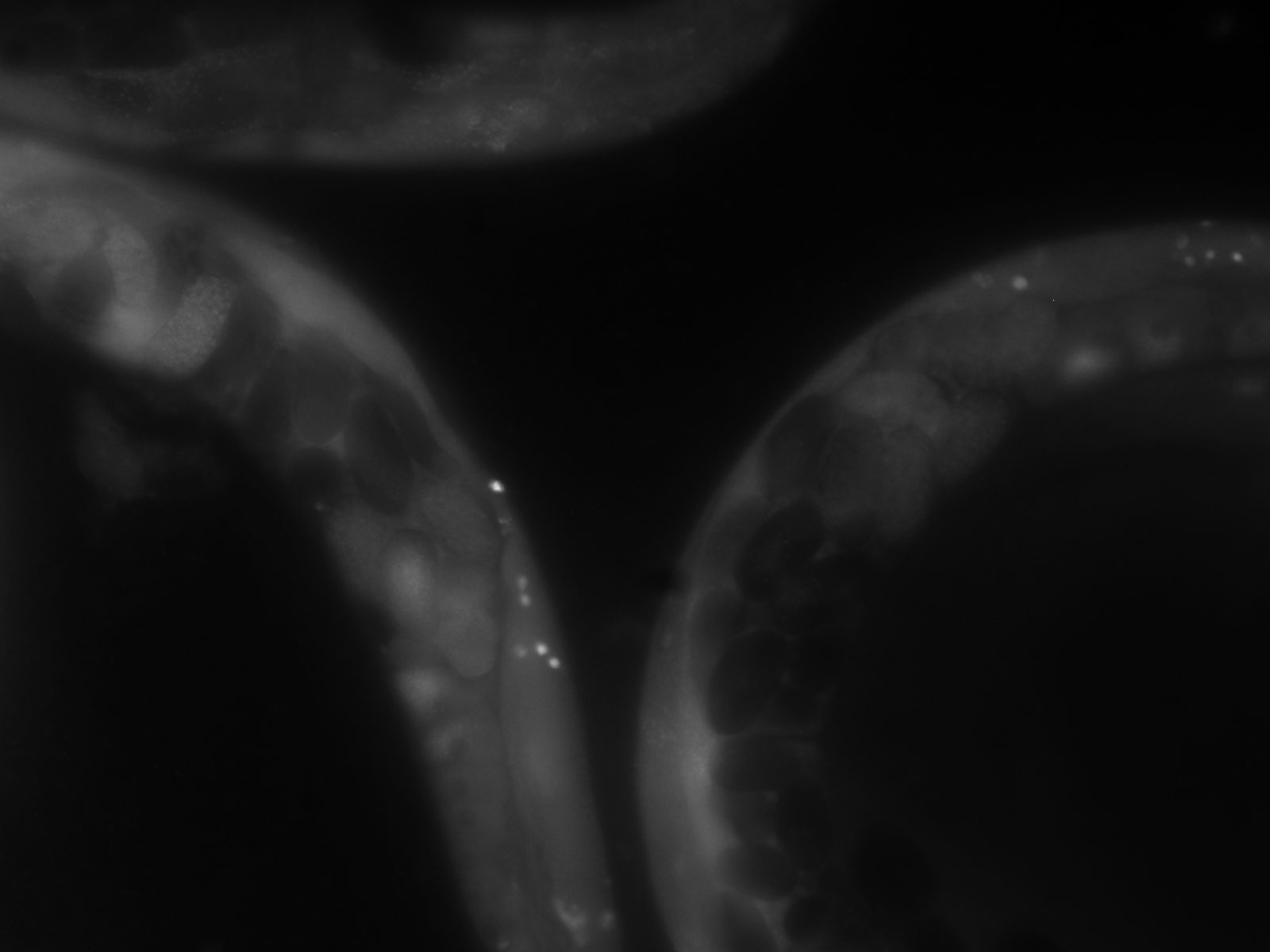

Supplement: S2 File — (ZIP) [file pgen.1011061.s002.zip › Fig.2A - Original files/Fig.2A RAW data and photos JPEG/syto12 staining - fig 2A - 1_rep - 14.5.23 jpeg/xbp-1+pad12117.jpg]

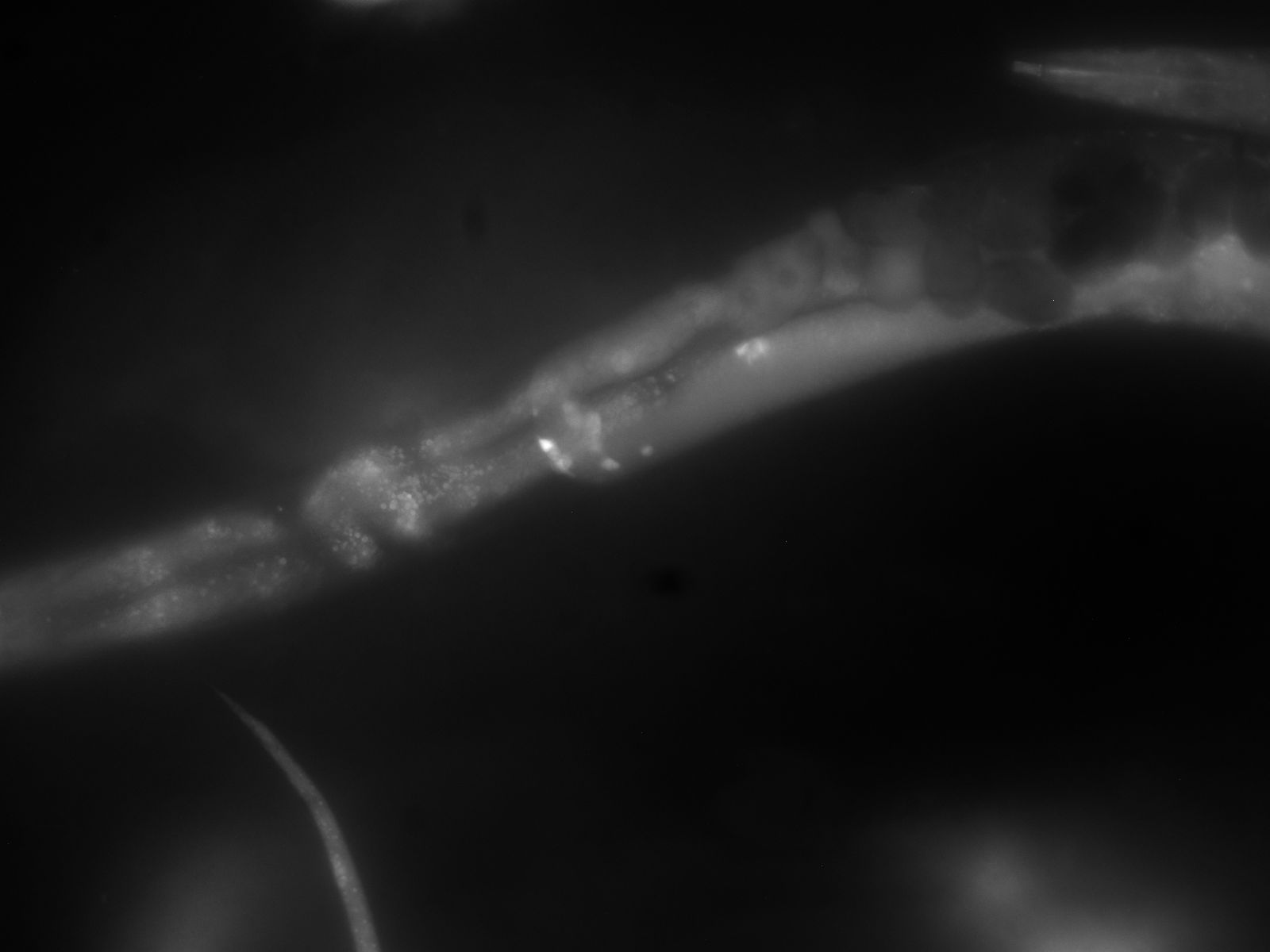

Supplement: S2 File — (ZIP) [file pgen.1011061.s002.zip › Fig.2A - Original files/Fig.2A RAW data and photos JPEG/syto12 staining - fig 2A - 1_rep - 14.5.23 jpeg/xbp-1+pad1296.jpg]

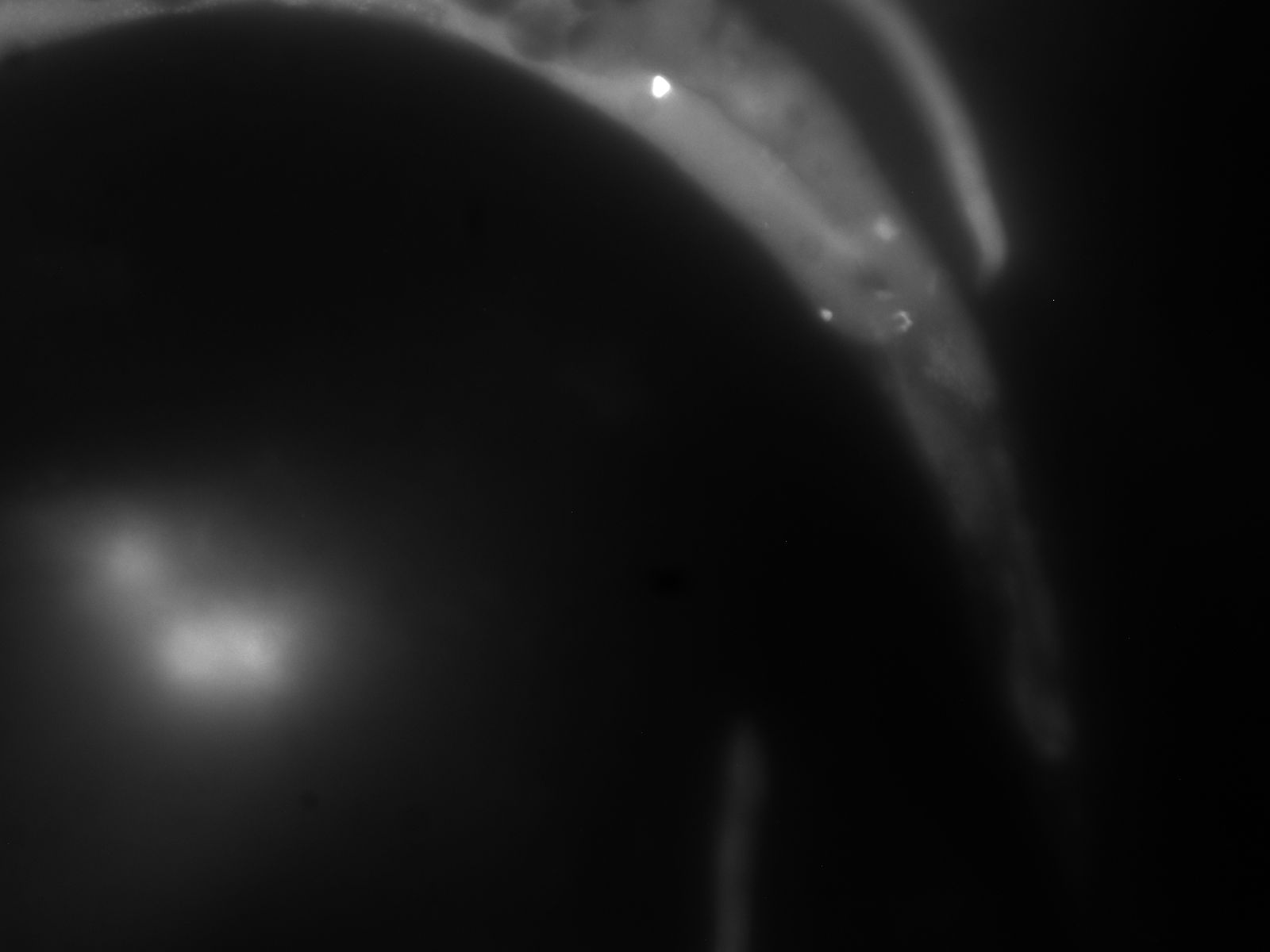

Supplement: S2 File — (ZIP) [file pgen.1011061.s002.zip › Fig.2A - Original files/Fig.2A RAW data and photos JPEG/syto12 staining - fig 2A - 1_rep - 14.5.23 jpeg/xbp-1+pad1297.jpg]

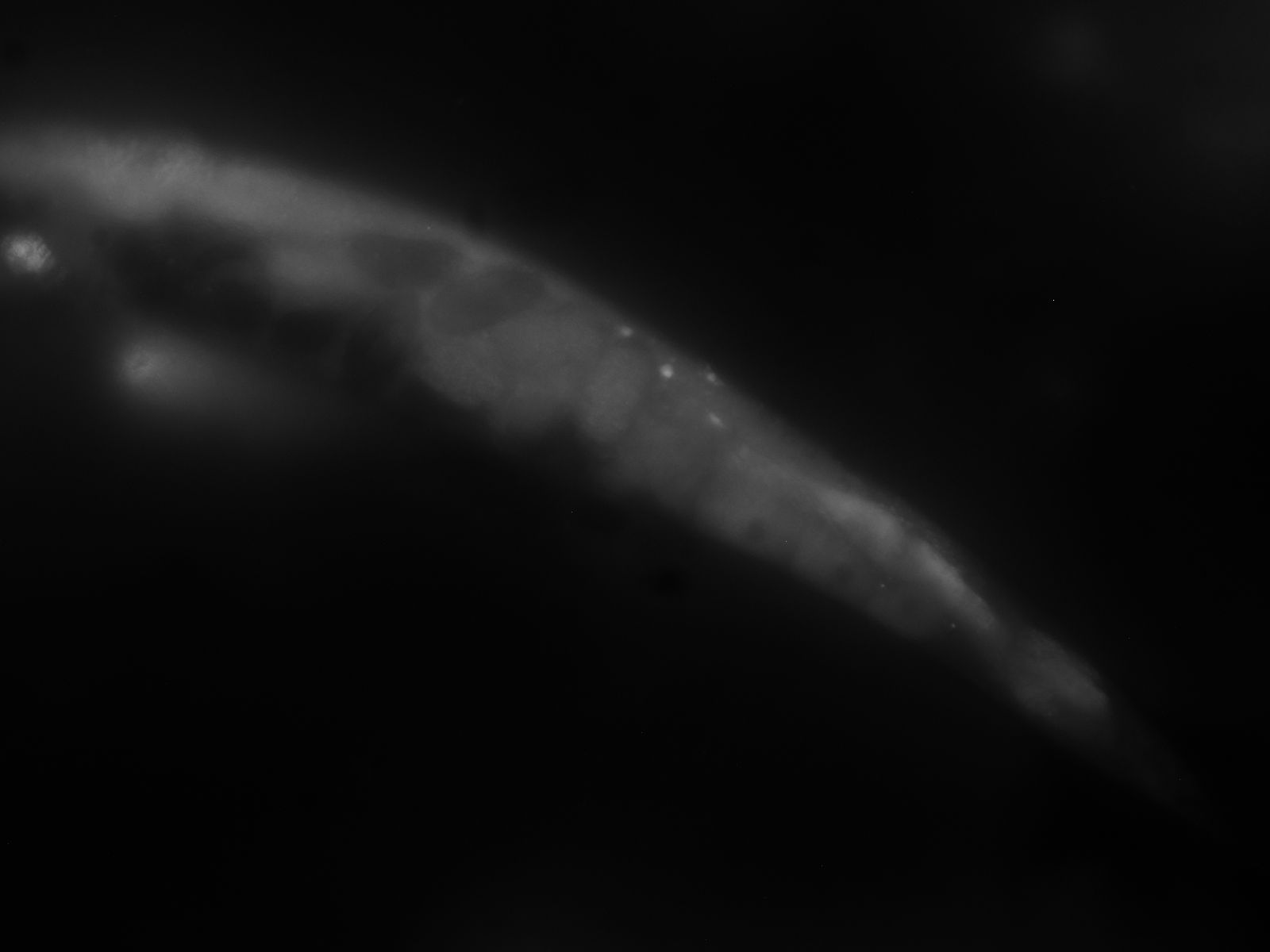

Supplement: S2 File — (ZIP) [file pgen.1011061.s002.zip › Fig.2A - Original files/Fig.2A RAW data and photos JPEG/syto12 staining - fig 2A - 1_rep - 14.5.23 jpeg/xbp-1+pad1298.jpg]

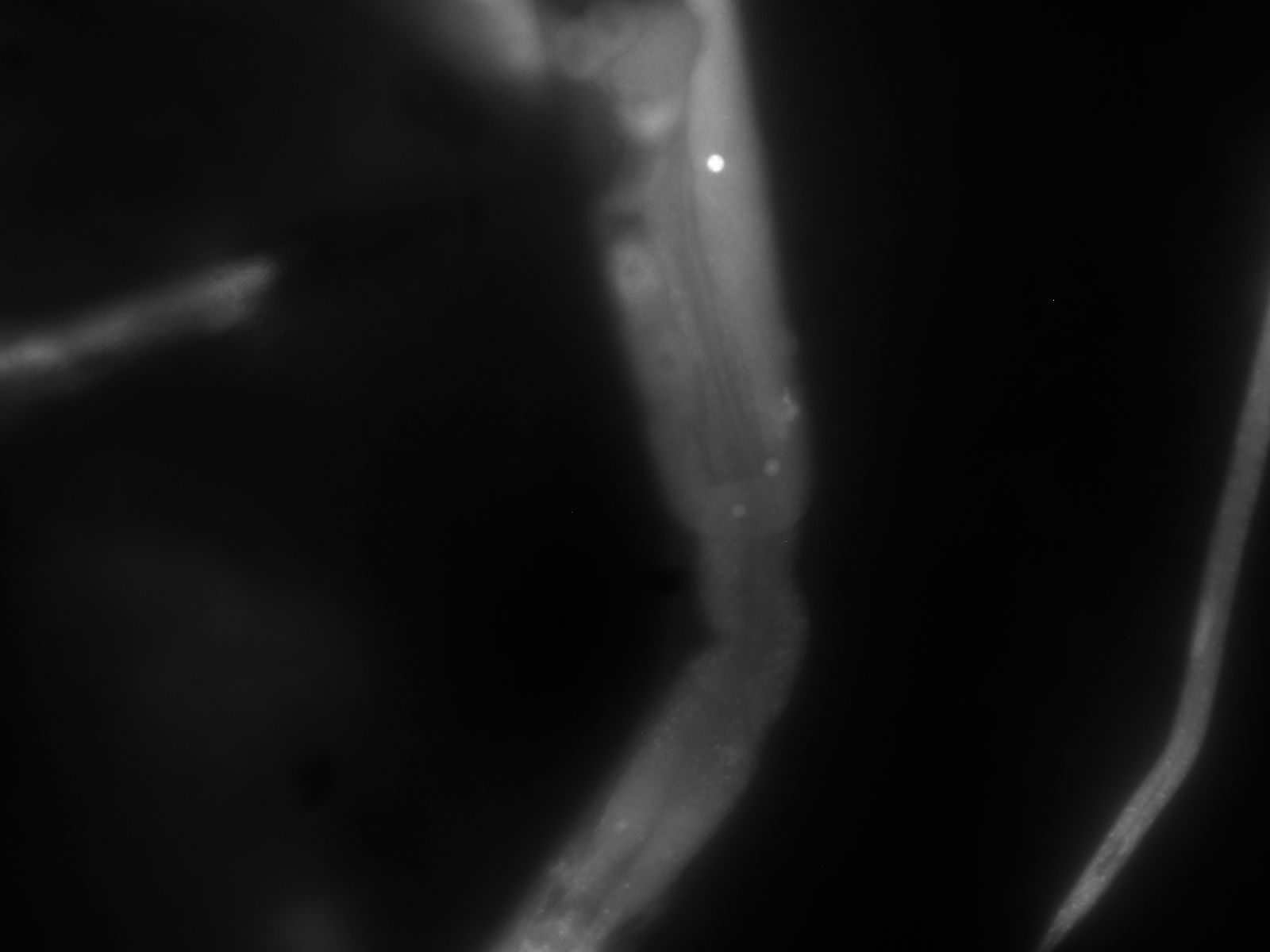

Supplement: S2 File — (ZIP) [file pgen.1011061.s002.zip › Fig.2A - Original files/Fig.2A RAW data and photos JPEG/syto12 staining - fig 2A - 1_rep - 14.5.23 jpeg/xbp-1+pad1299.jpg]

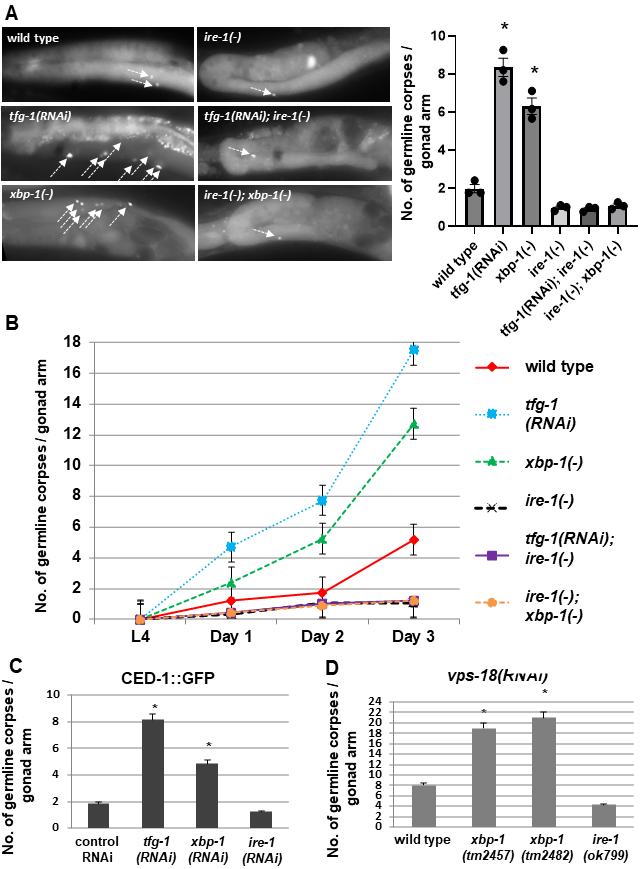

Supplement: S2 File — (ZIP) [file pgen.1011061.s002.zip › Fig.2A - Original files/Updated Figure 2 - 13.7.23.tif]

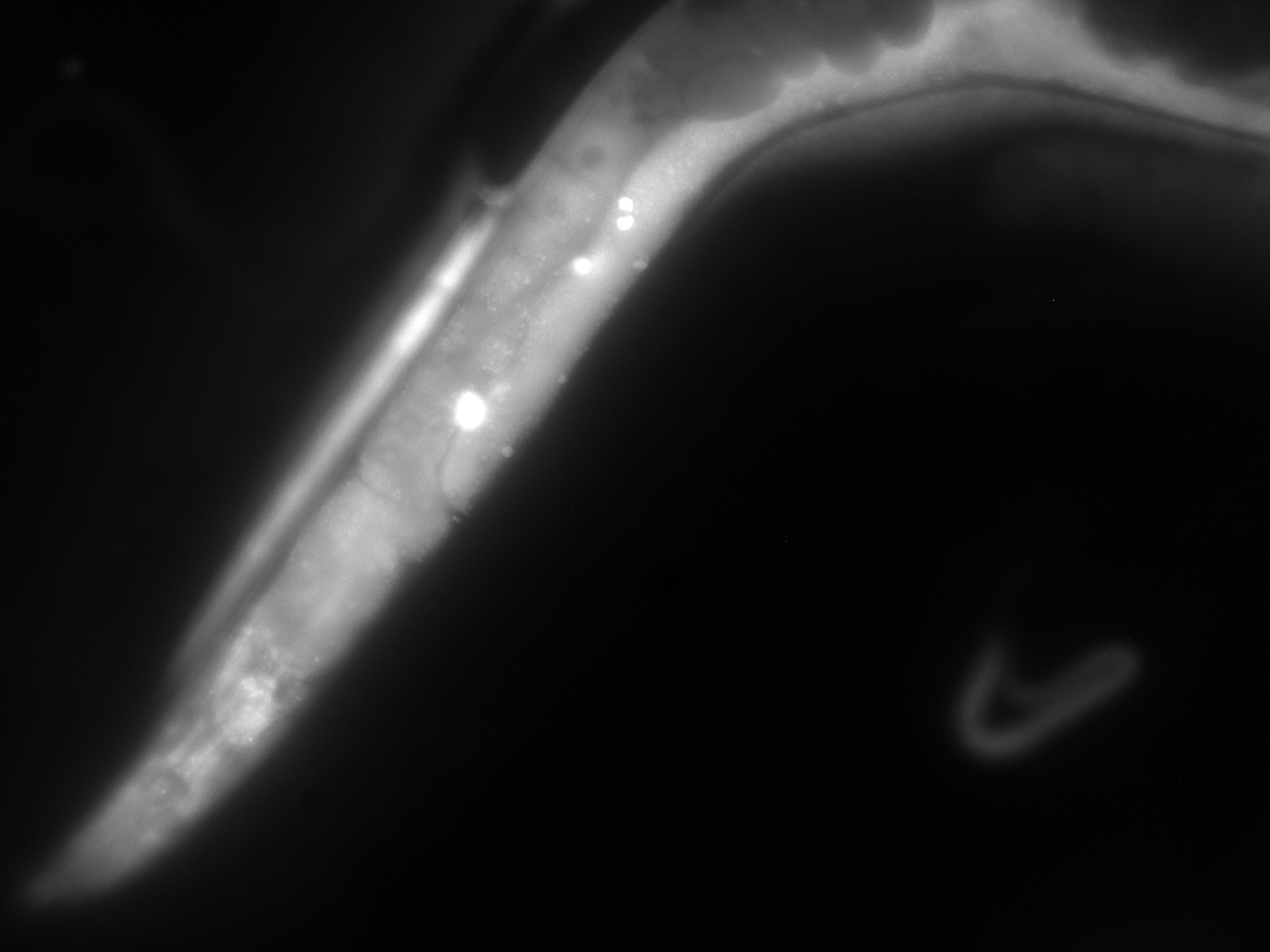

Supplement: S3 File — (ZIP) [file pgen.1011061.s003.zip › Fig.4D - Original files/Fig. 4D pictures selected for Figure 4D/daf-28_sa+ pad1232.tif]

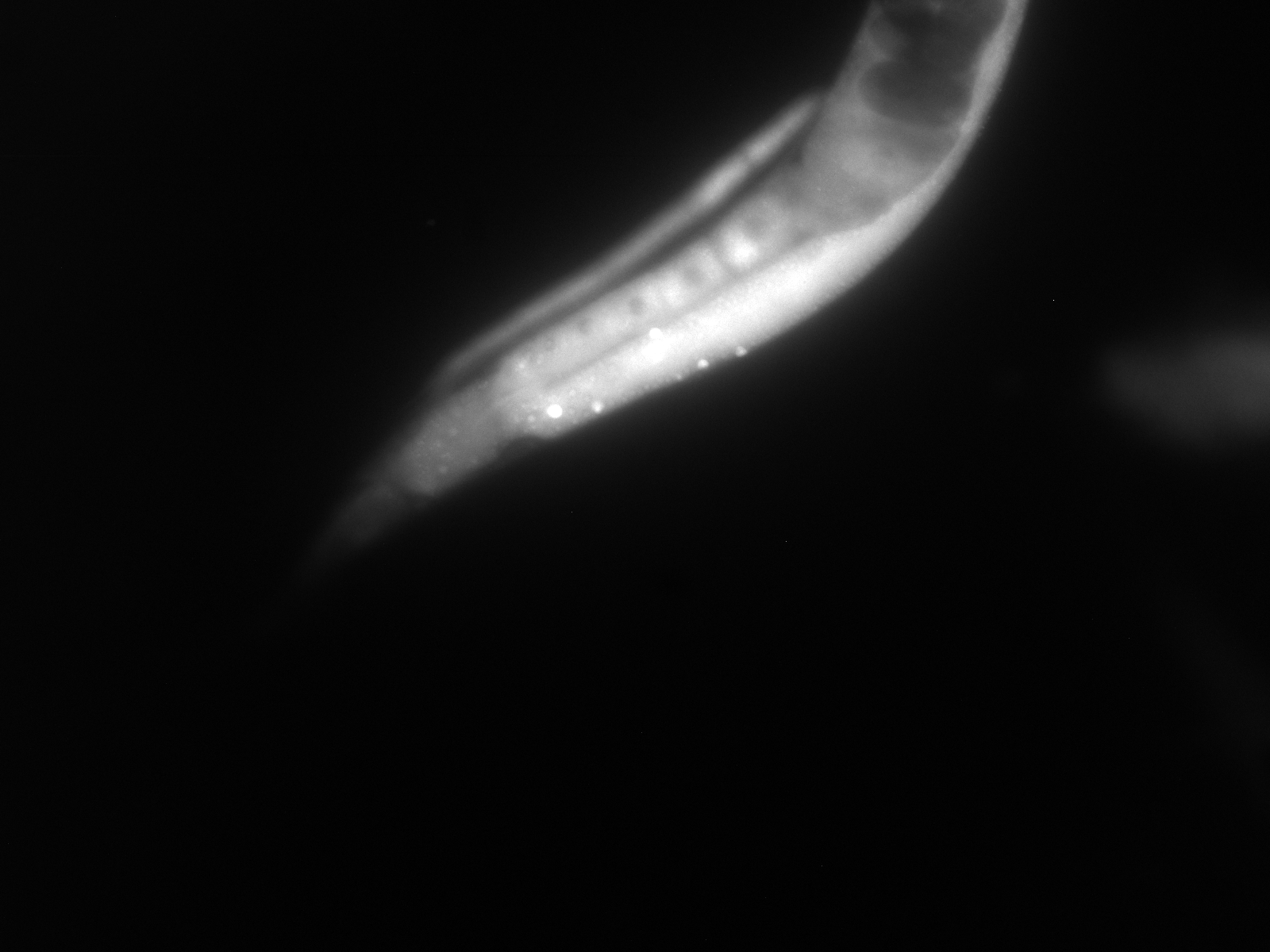

Supplement: S3 File — (ZIP) [file pgen.1011061.s003.zip › Fig.4D - Original files/Fig. 4D pictures selected for Figure 4D/daf-28_sa+tfg-134.tif]

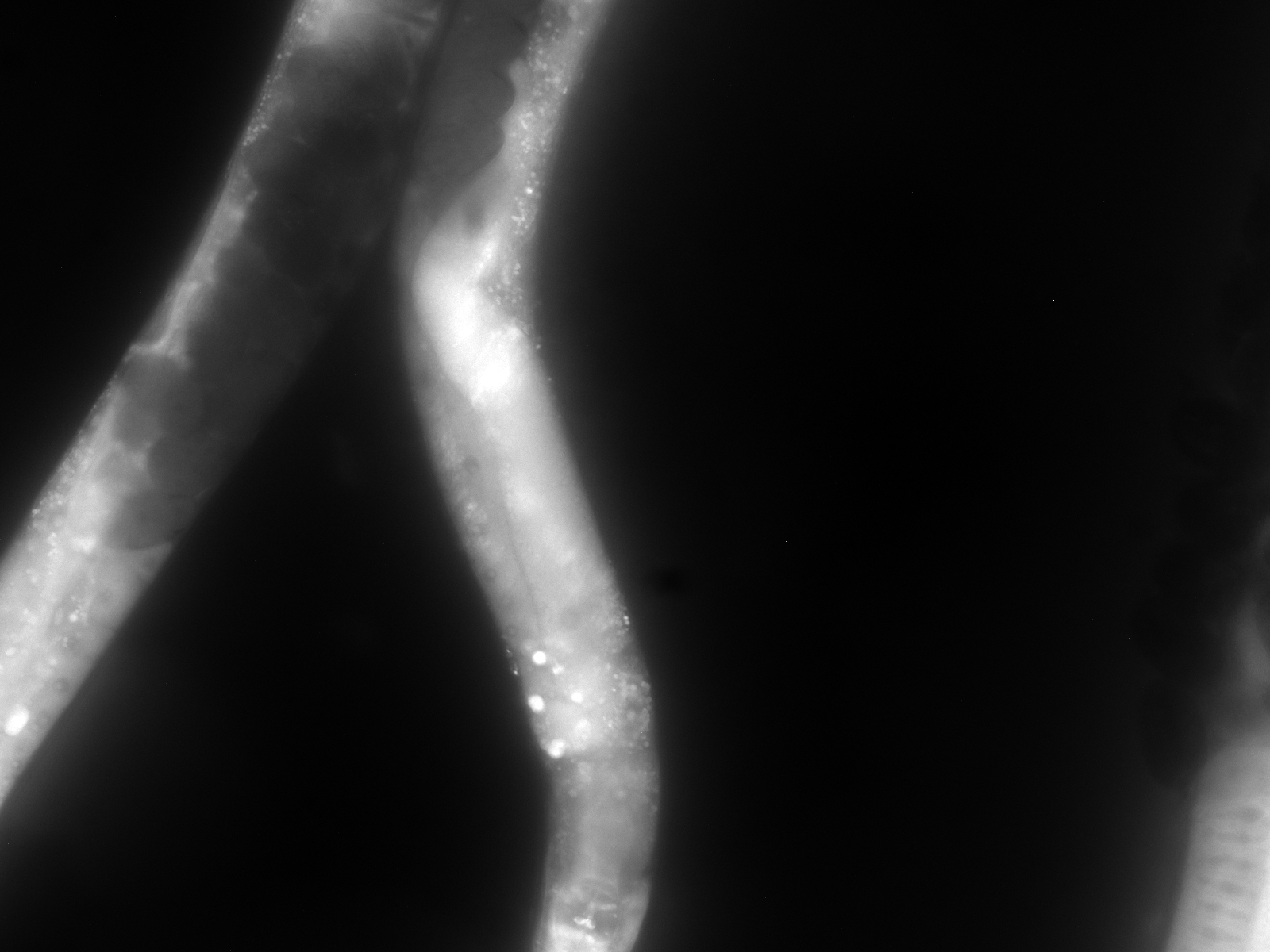

Supplement: S3 File — (ZIP) [file pgen.1011061.s003.zip › Fig.4D - Original files/Fig. 4D pictures selected for Figure 4D/daf-28_tm2308+ tfg-124.tif]

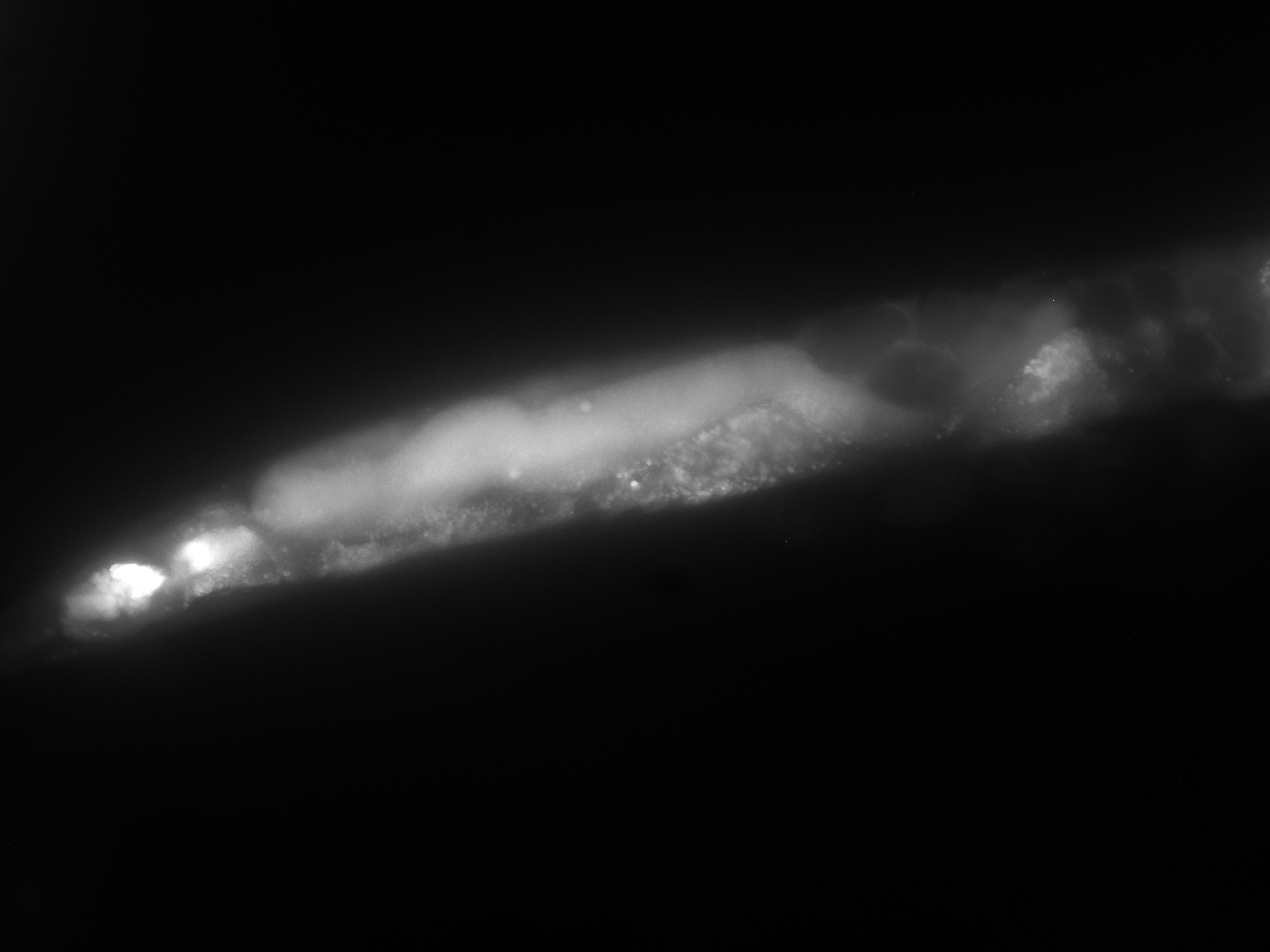

Supplement: S3 File — (ZIP) [file pgen.1011061.s003.zip › Fig.4D - Original files/Fig. 4D pictures selected for Figure 4D/daf-28_tm2308+pad1219.tif]

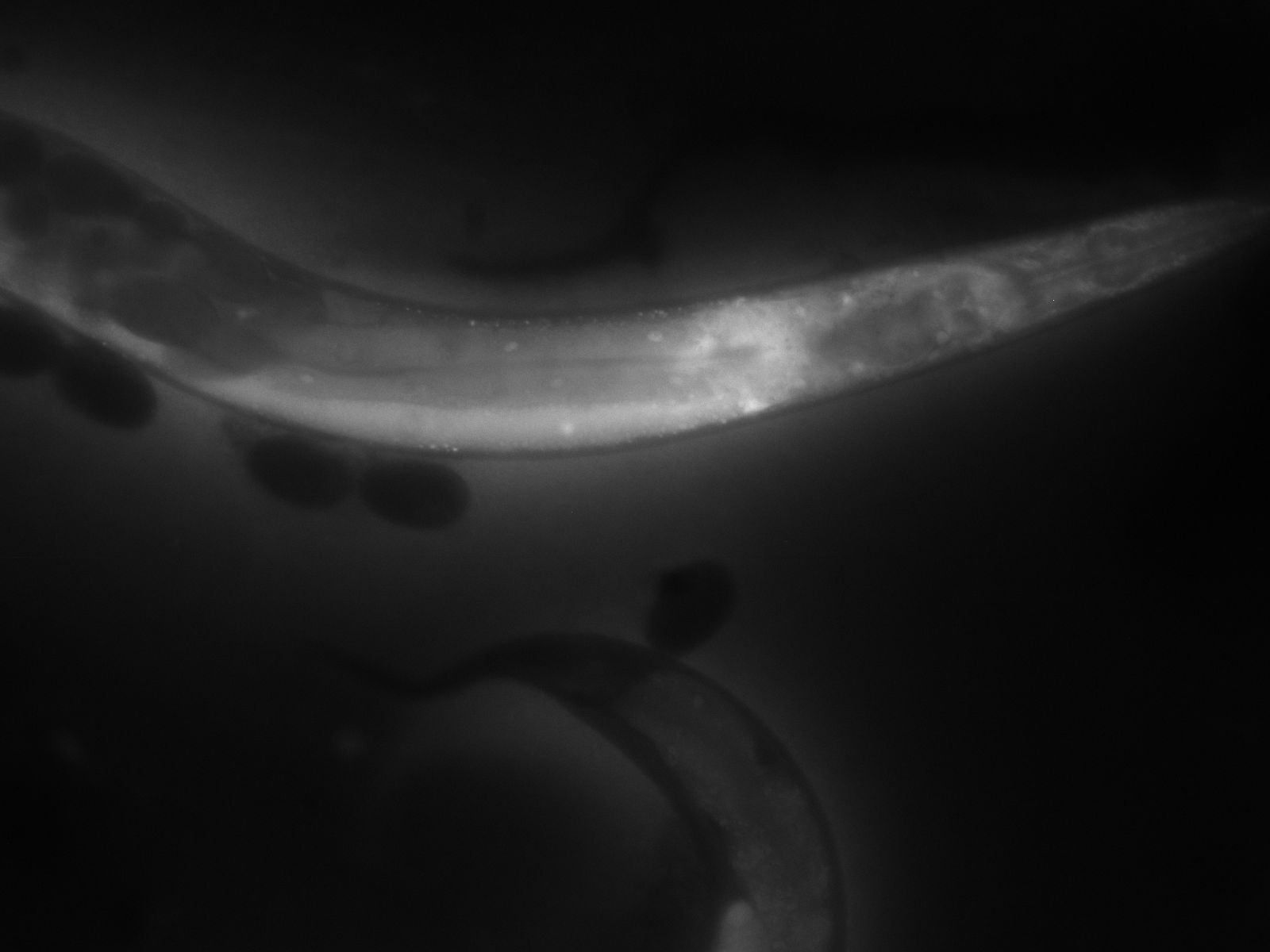

Supplement: S3 File — (ZIP) [file pgen.1011061.s003.zip › Fig.4D - Original files/Fig.4D RAW data and photos JPEG/syto12 staining - Fig 4D - 2 rep_15.5.23 jpeg/daf-28_sa+ pad1230.jpg]

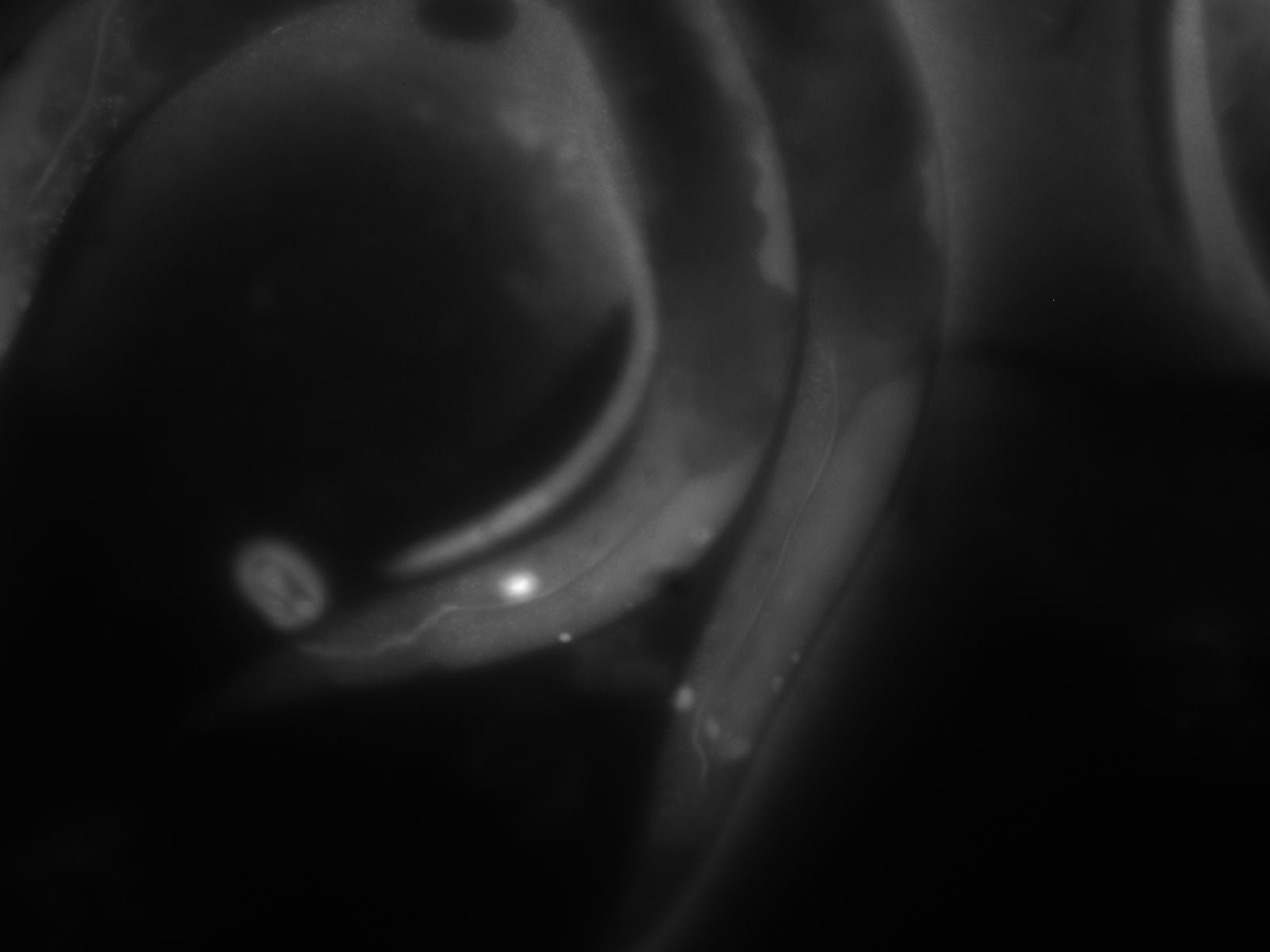

Supplement: S3 File — (ZIP) [file pgen.1011061.s003.zip › Fig.4D - Original files/Fig.4D RAW data and photos JPEG/syto12 staining - Fig 4D - 2 rep_15.5.23 jpeg/daf-28_sa+ pad1231.jpg]

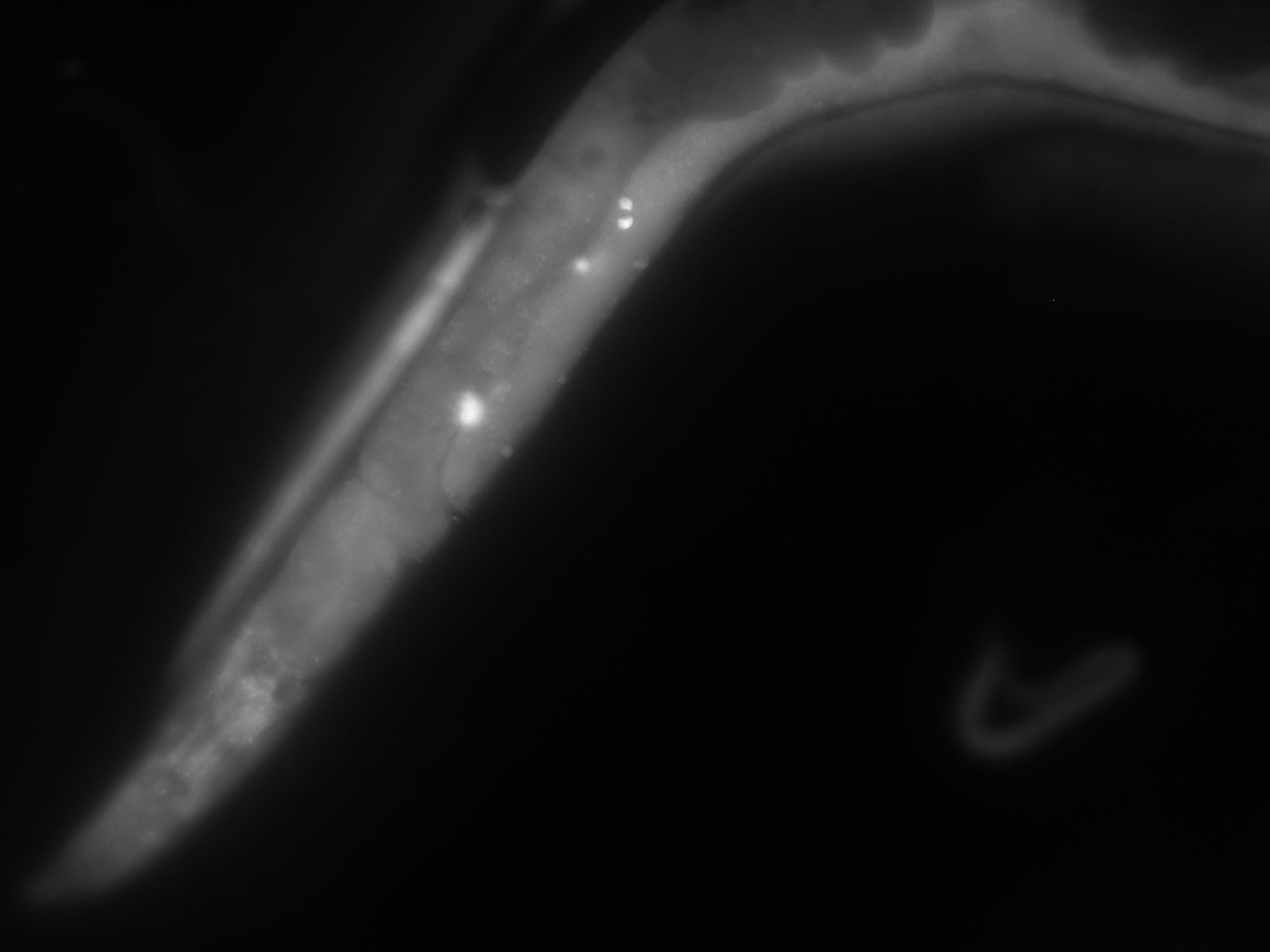

Supplement: S3 File — (ZIP) [file pgen.1011061.s003.zip › Fig.4D - Original files/Fig.4D RAW data and photos JPEG/syto12 staining - Fig 4D - 2 rep_15.5.23 jpeg/daf-28_sa+ pad1232.jpg]

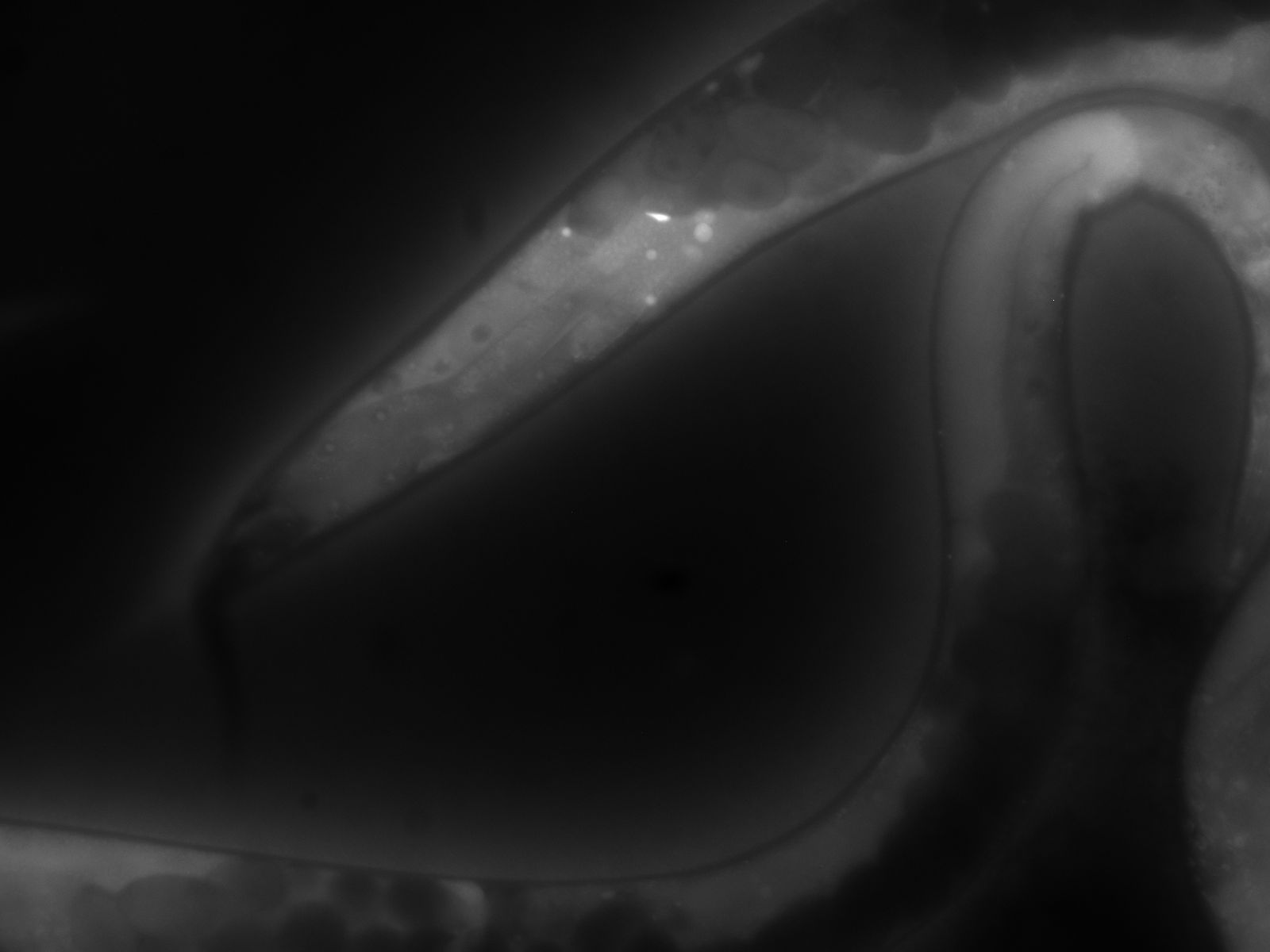

Supplement: S3 File — (ZIP) [file pgen.1011061.s003.zip › Fig.4D - Original files/Fig.4D RAW data and photos JPEG/syto12 staining - Fig 4D - 2 rep_15.5.23 jpeg/daf-28_sa+ pad1233.jpg]

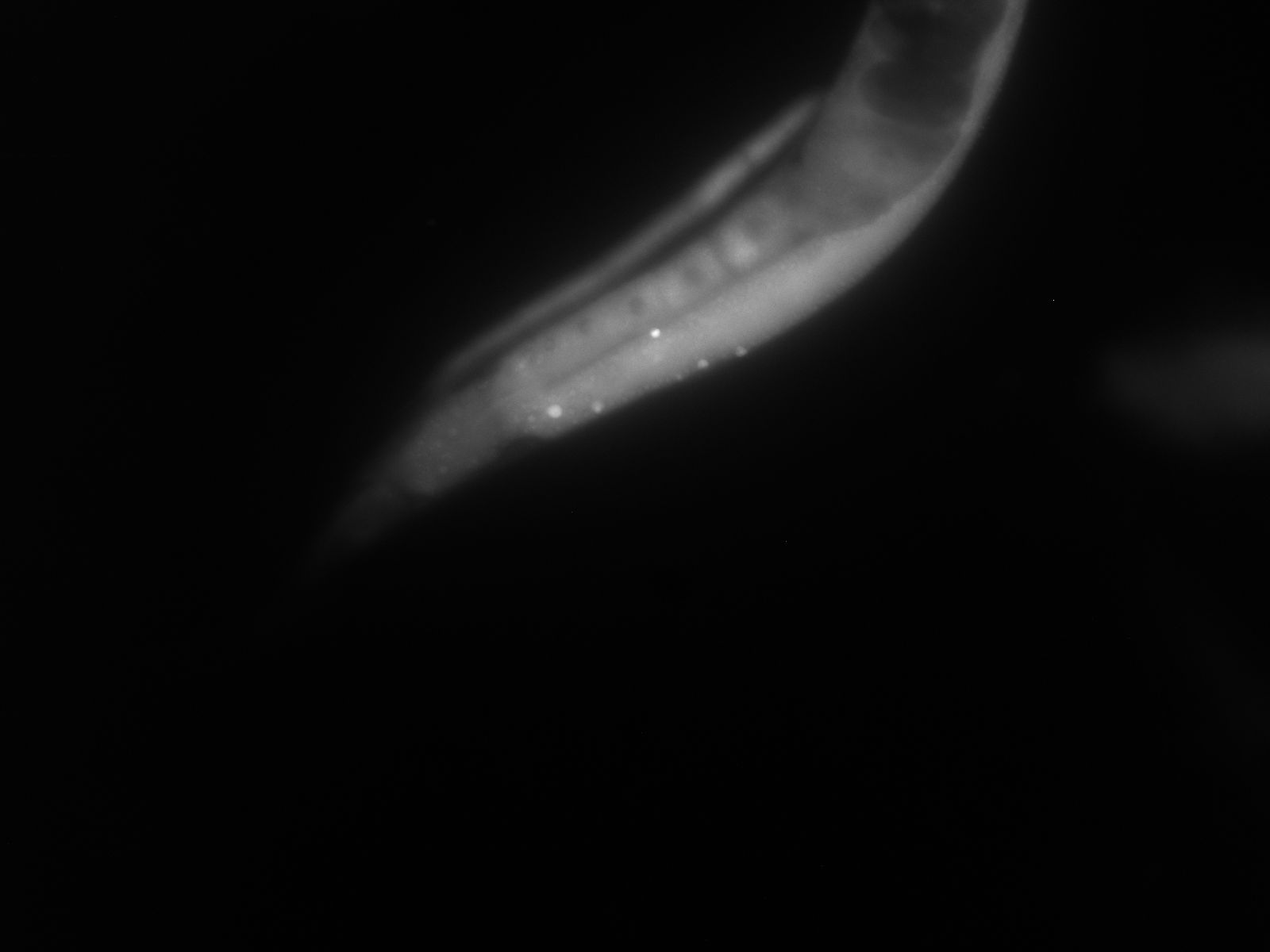

Supplement: S3 File — (ZIP) [file pgen.1011061.s003.zip › Fig.4D - Original files/Fig.4D RAW data and photos JPEG/syto12 staining - Fig 4D - 2 rep_15.5.23 jpeg/daf-28_sa+tfg-134.jpg]

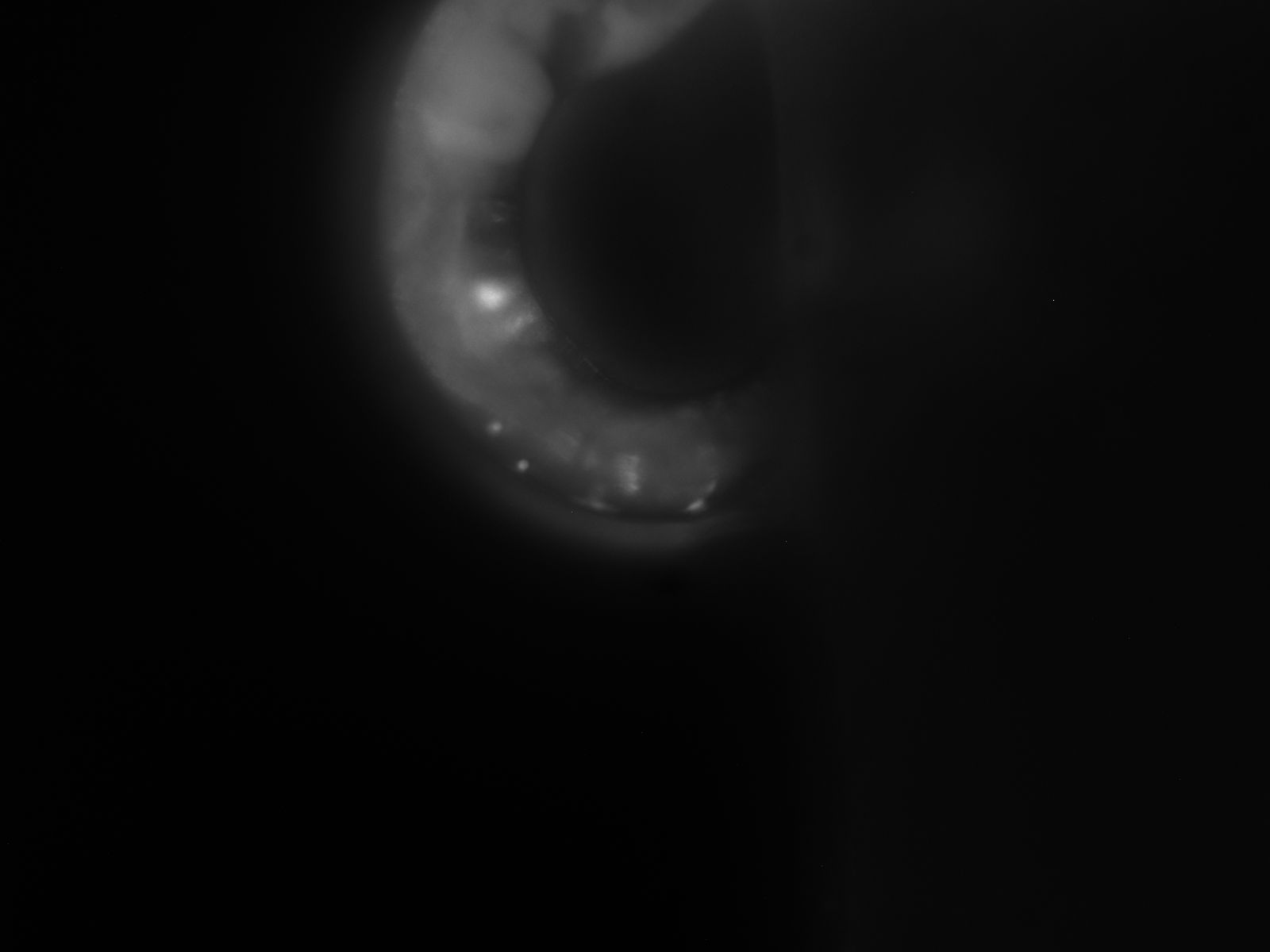

Supplement: S3 File — (ZIP) [file pgen.1011061.s003.zip › Fig.4D - Original files/Fig.4D RAW data and photos JPEG/syto12 staining - Fig 4D - 2 rep_15.5.23 jpeg/daf-28_sa+tfg-135.jpg]

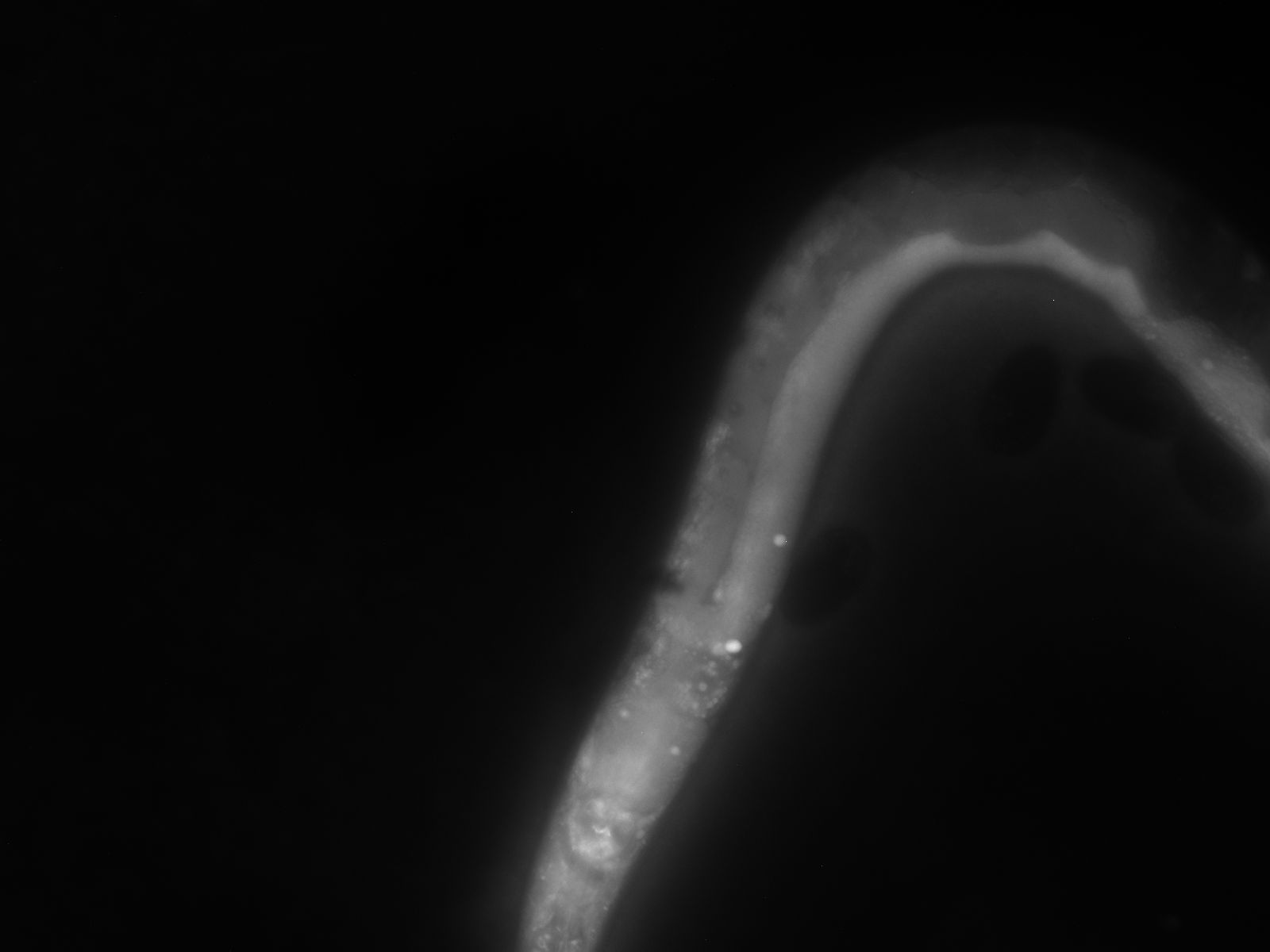

Supplement: S3 File — (ZIP) [file pgen.1011061.s003.zip › Fig.4D - Original files/Fig.4D RAW data and photos JPEG/syto12 staining - Fig 4D - 2 rep_15.5.23 jpeg/daf-28_sa+tfg-136.jpg]

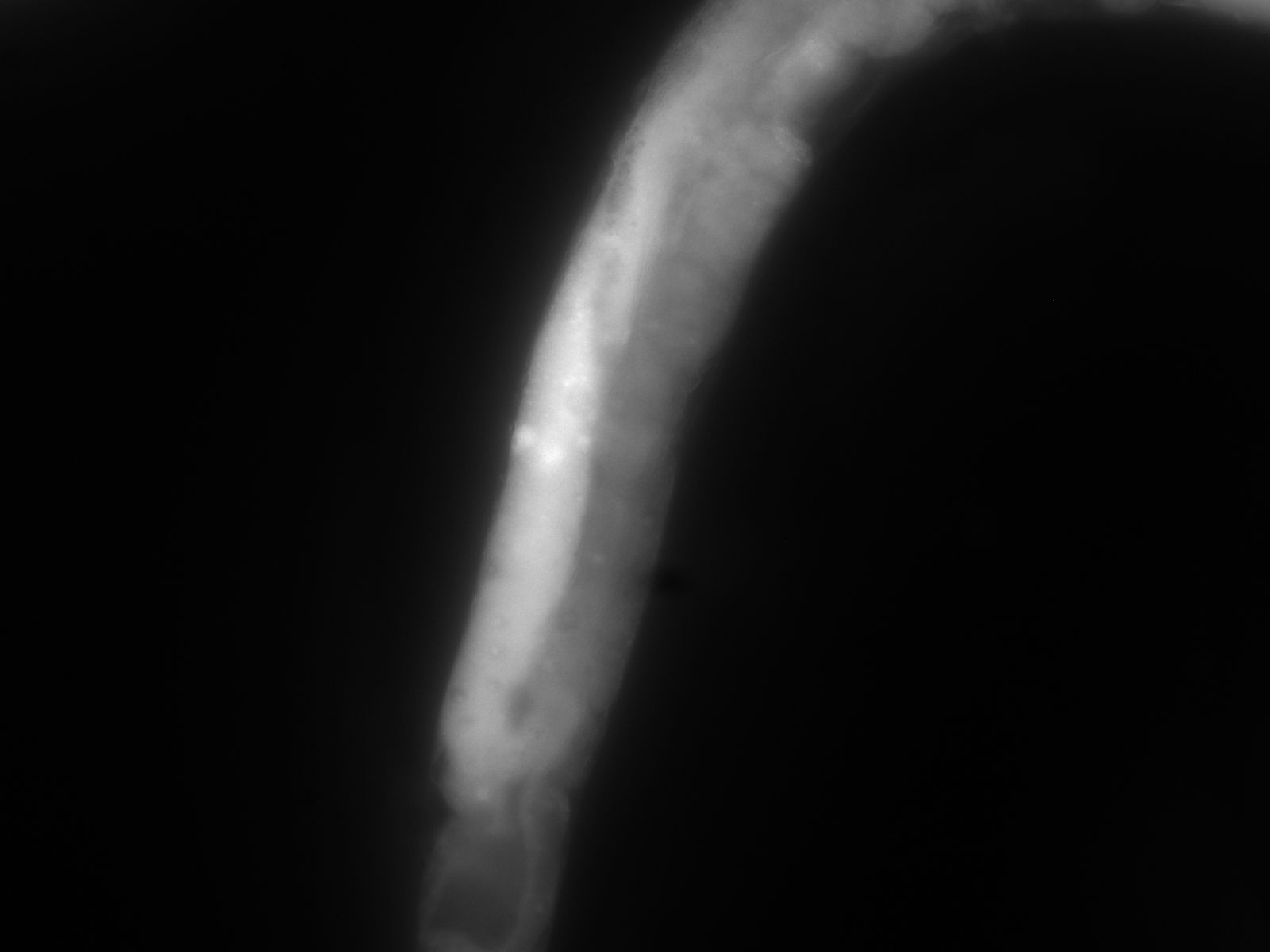

Supplement: S3 File — (ZIP) [file pgen.1011061.s003.zip › Fig.4D - Original files/Fig.4D RAW data and photos JPEG/syto12 staining - Fig 4D - 2 rep_15.5.23 jpeg/daf-28_tm2308+ tfg-121.jpg]

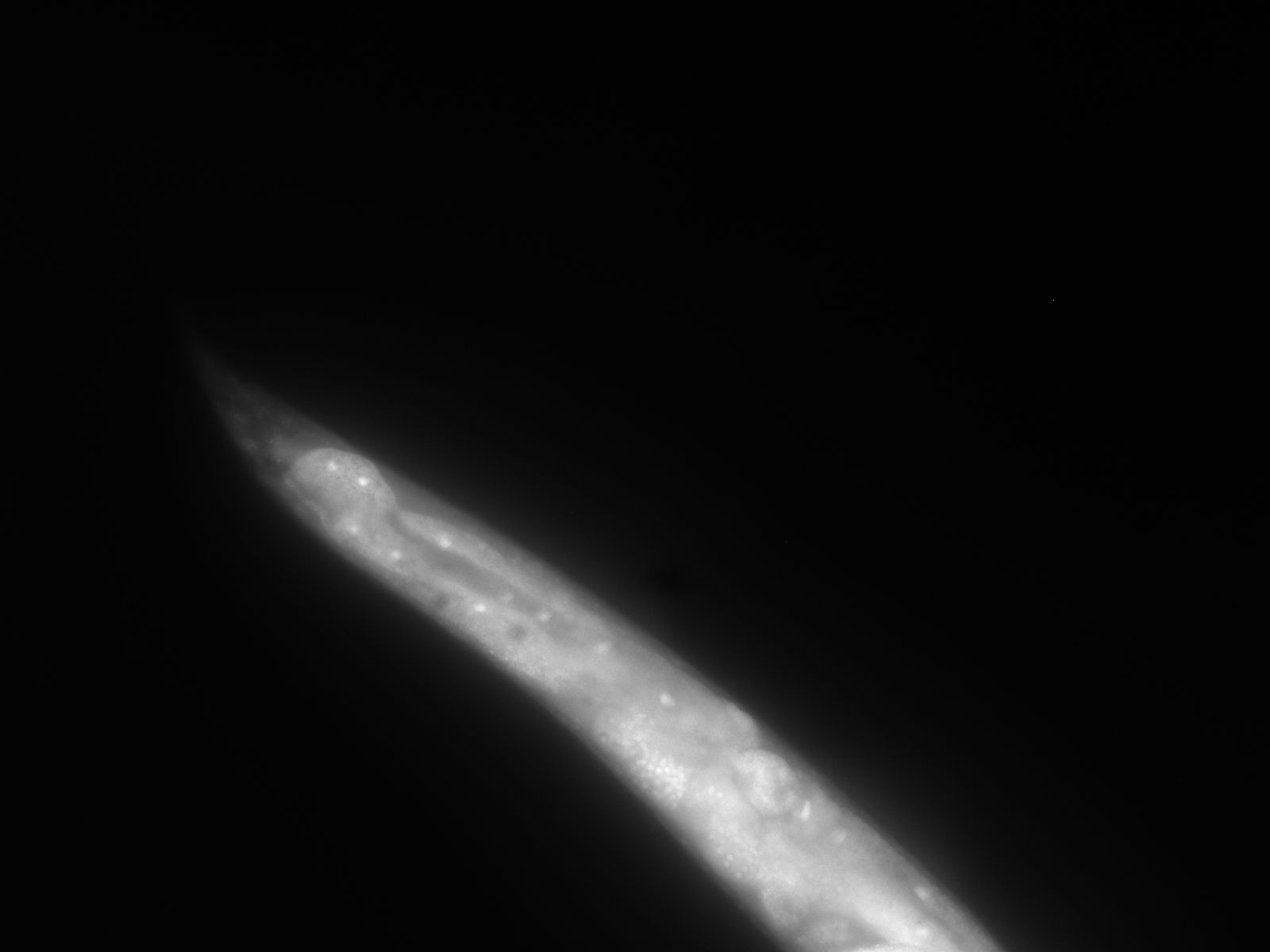

Supplement: S3 File — (ZIP) [file pgen.1011061.s003.zip › Fig.4D - Original files/Fig.4D RAW data and photos JPEG/syto12 staining - Fig 4D - 2 rep_15.5.23 jpeg/daf-28_tm2308+ tfg-122.jpg]

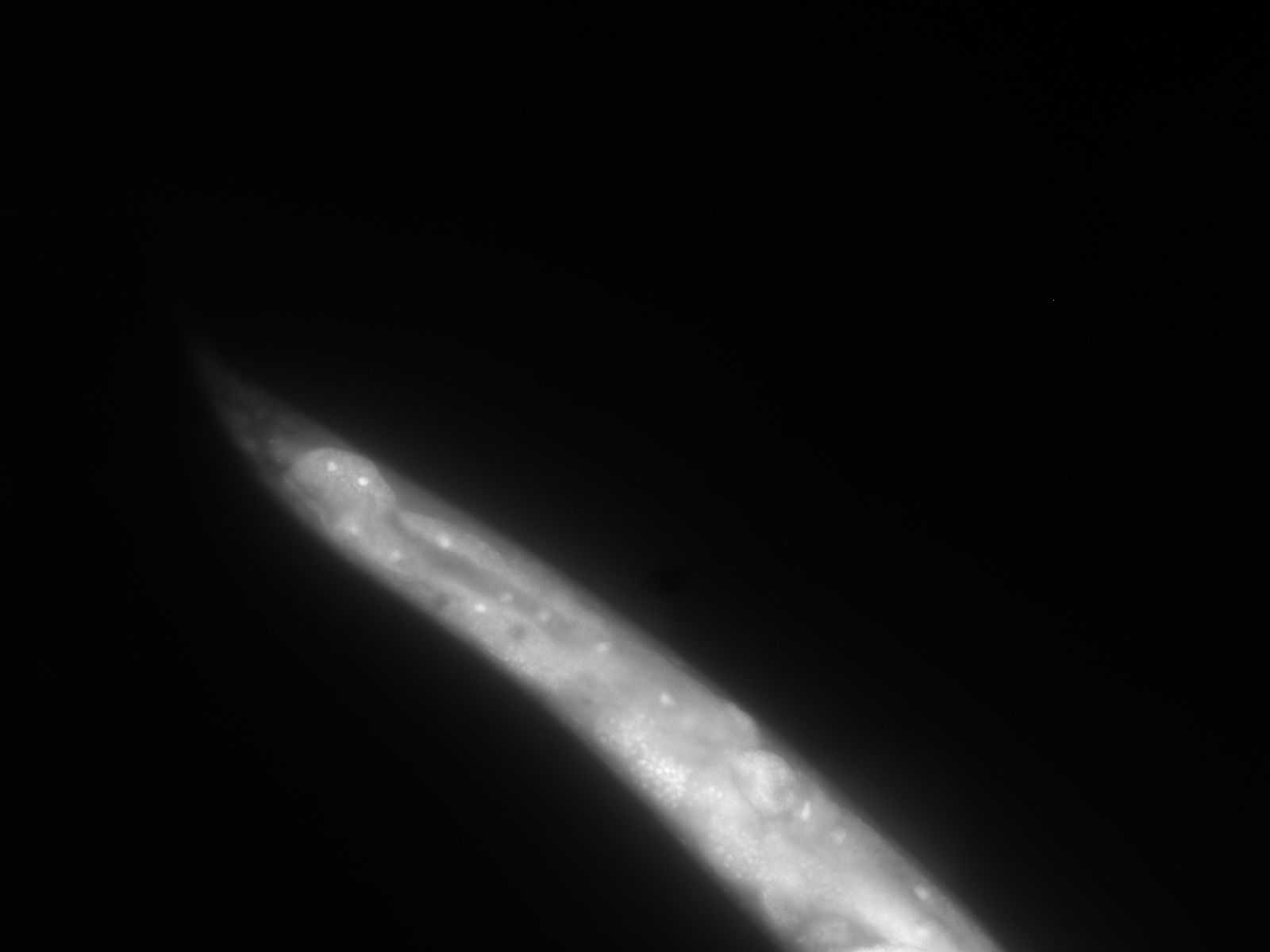

Supplement: S3 File — (ZIP) [file pgen.1011061.s003.zip › Fig.4D - Original files/Fig.4D RAW data and photos JPEG/syto12 staining - Fig 4D - 2 rep_15.5.23 jpeg/daf-28_tm2308+ tfg-123.jpg]

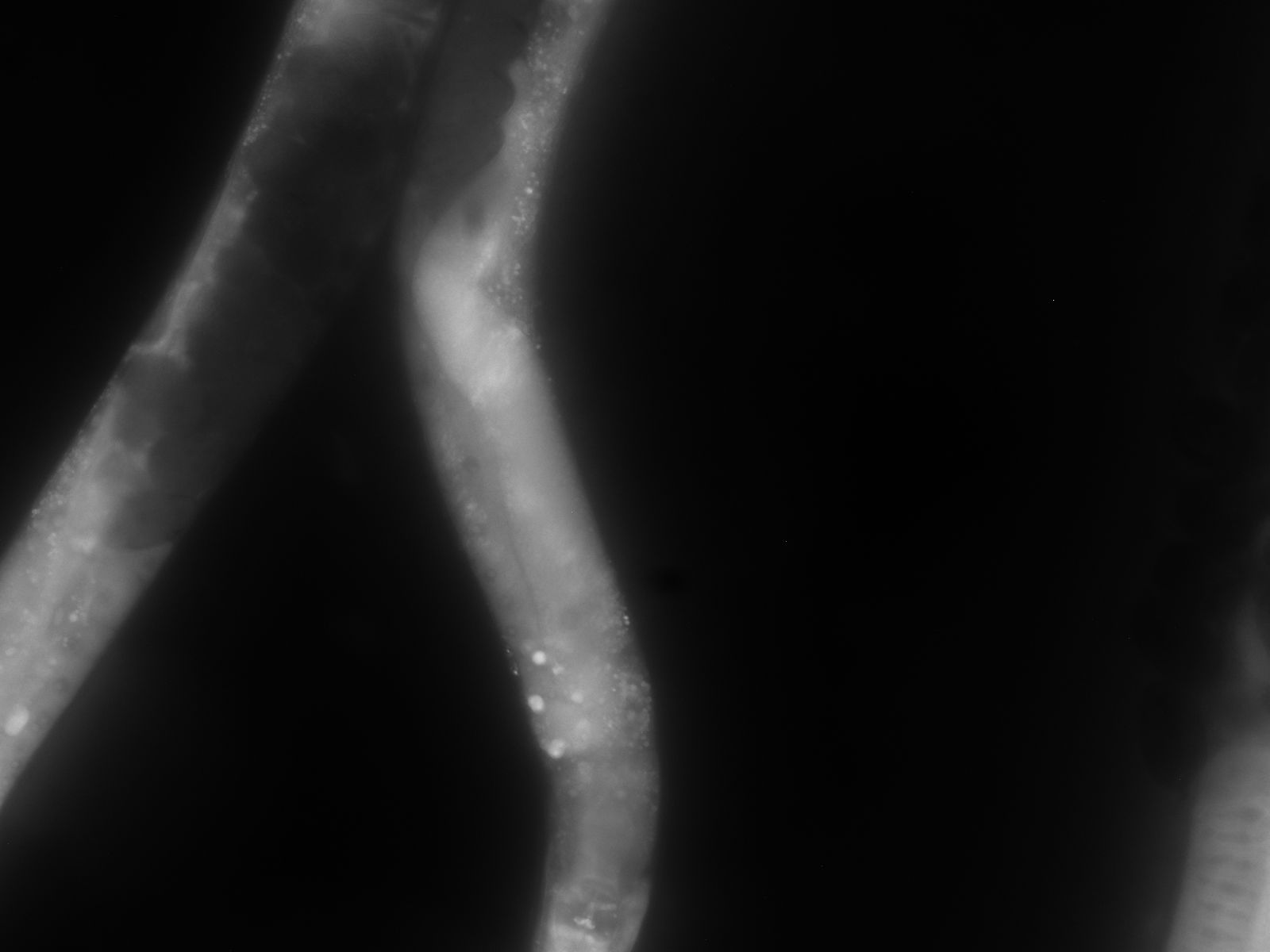

Supplement: S3 File — (ZIP) [file pgen.1011061.s003.zip › Fig.4D - Original files/Fig.4D RAW data and photos JPEG/syto12 staining - Fig 4D - 2 rep_15.5.23 jpeg/daf-28_tm2308+ tfg-124.jpg]

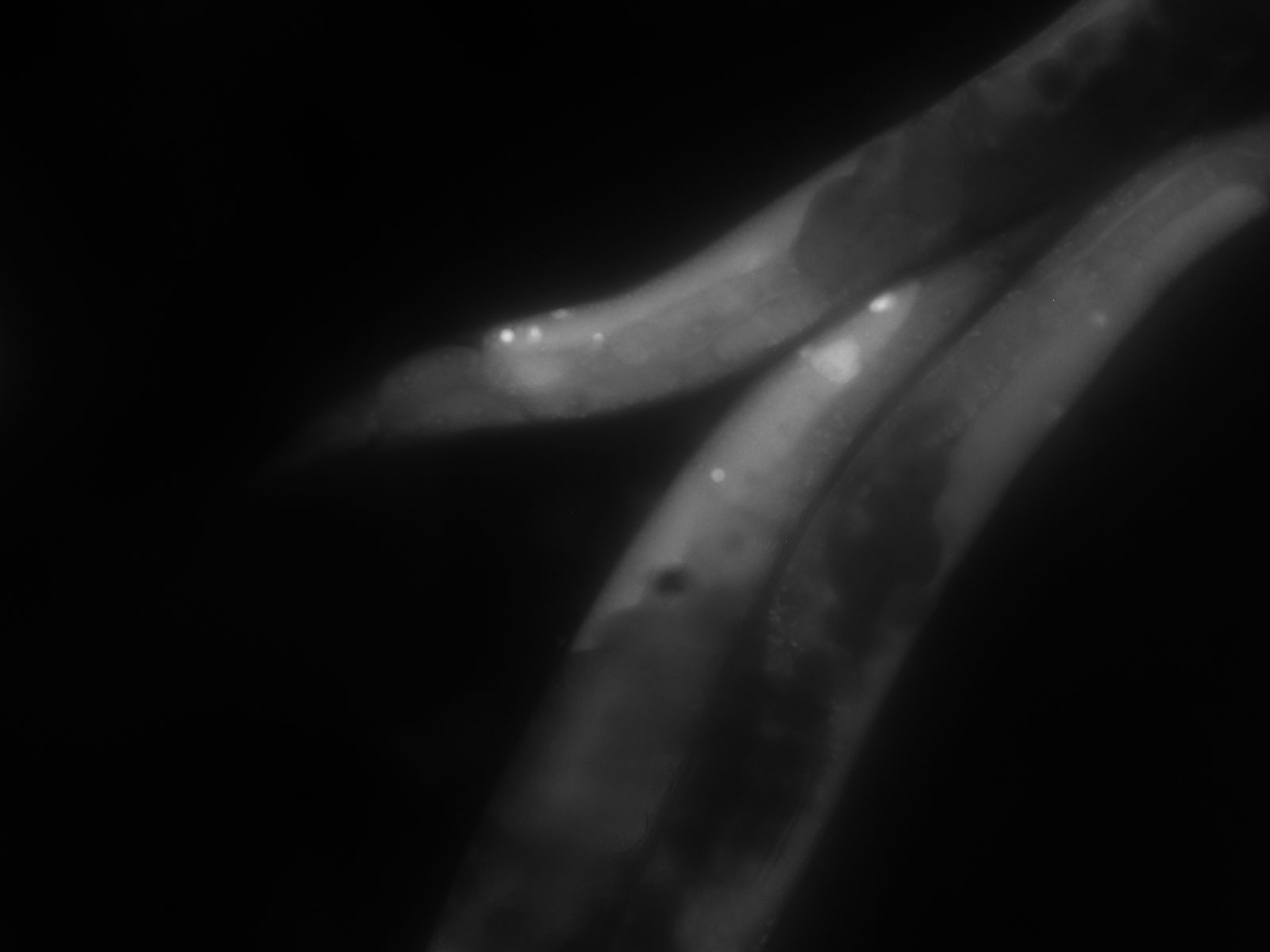

Supplement: S3 File — (ZIP) [file pgen.1011061.s003.zip › Fig.4D - Original files/Fig.4D RAW data and photos JPEG/syto12 staining - Fig 4D - 2 rep_15.5.23 jpeg/daf-28_tm2308+ tfg-125.jpg]

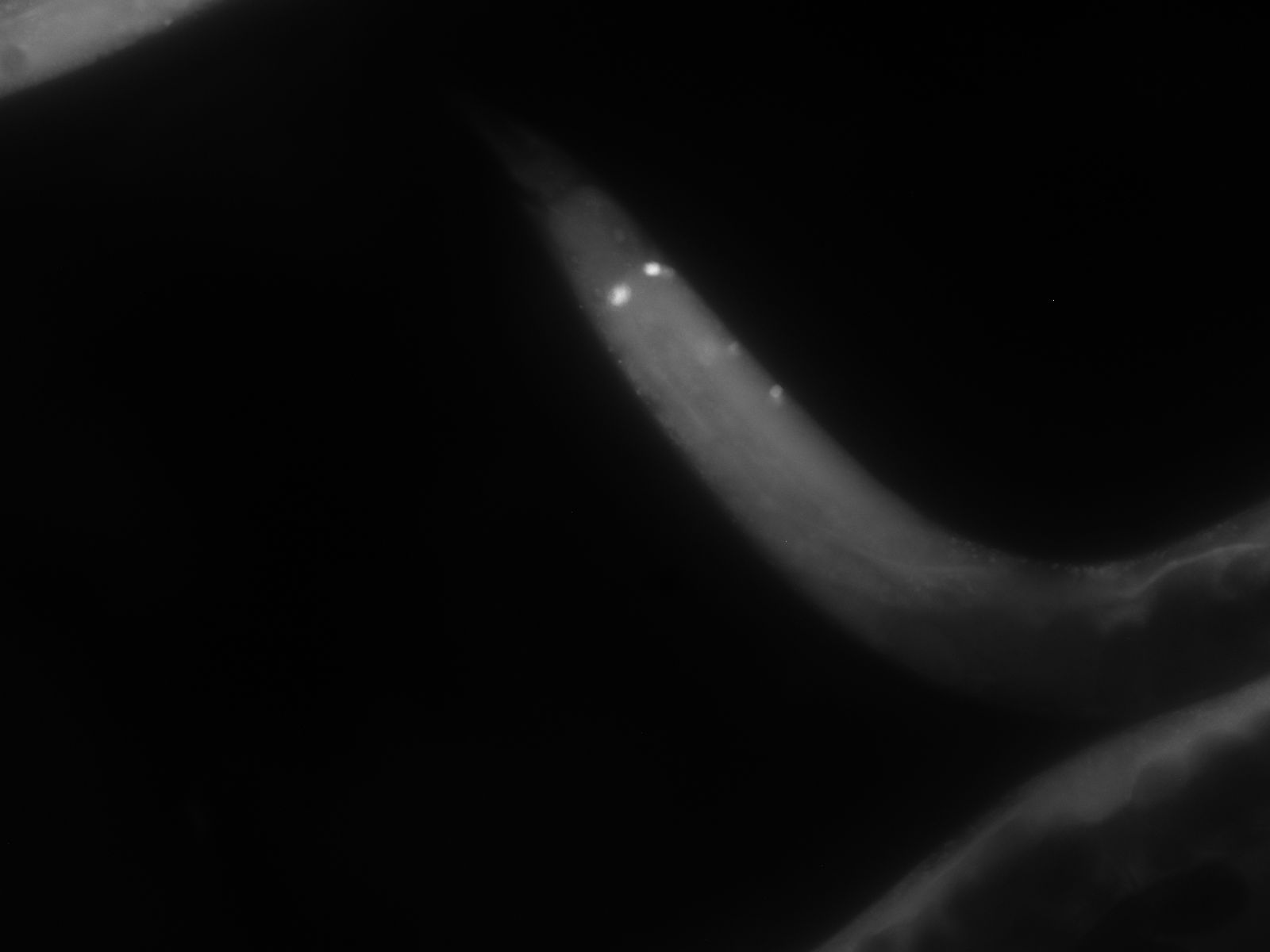

Supplement: S3 File — (ZIP) [file pgen.1011061.s003.zip › Fig.4D - Original files/Fig.4D RAW data and photos JPEG/syto12 staining - Fig 4D - 2 rep_15.5.23 jpeg/daf-28_tm2308+ tfg-126.jpg]

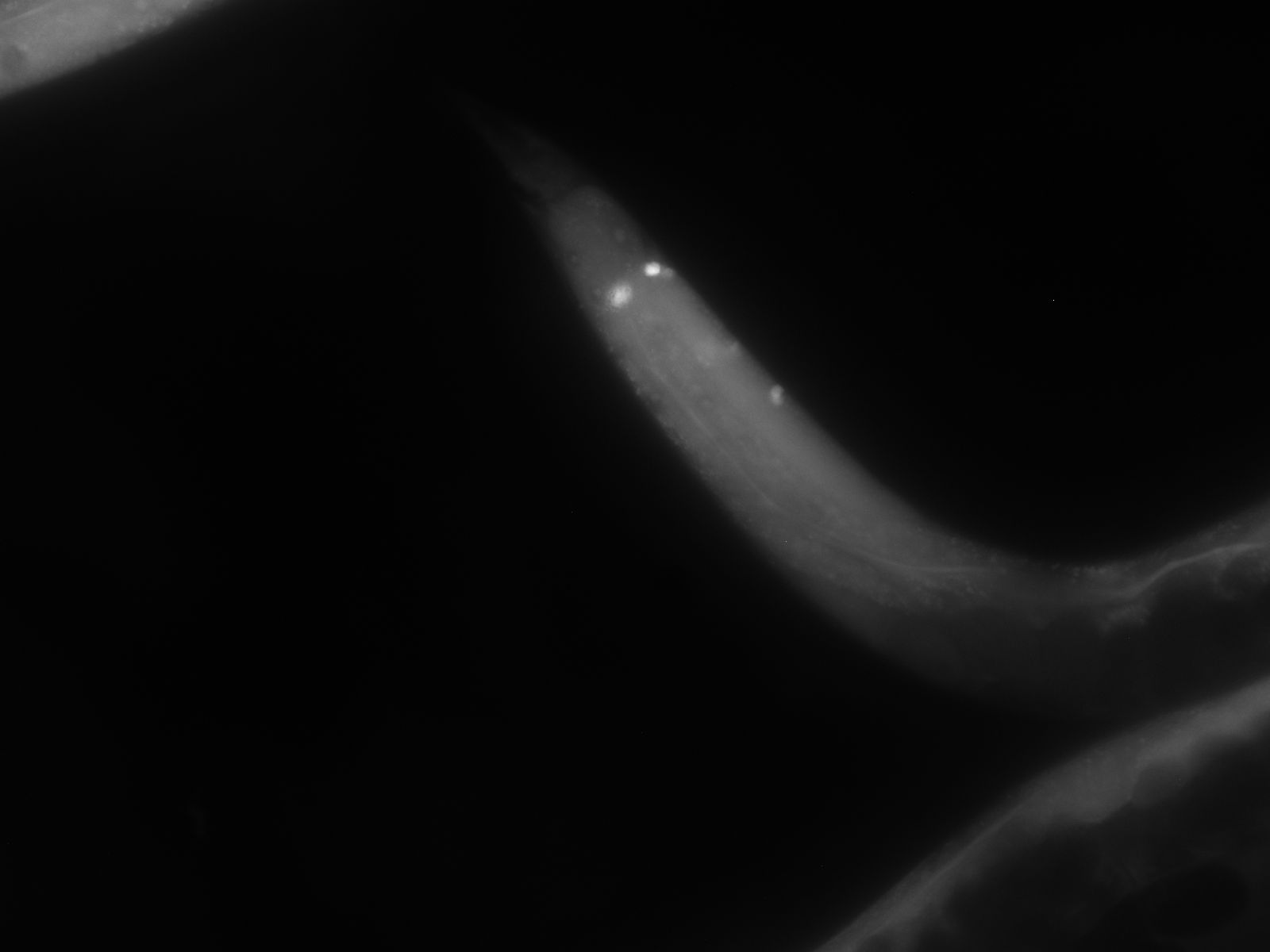

Supplement: S3 File — (ZIP) [file pgen.1011061.s003.zip › Fig.4D - Original files/Fig.4D RAW data and photos JPEG/syto12 staining - Fig 4D - 2 rep_15.5.23 jpeg/daf-28_tm2308+ tfg-127.jpg]

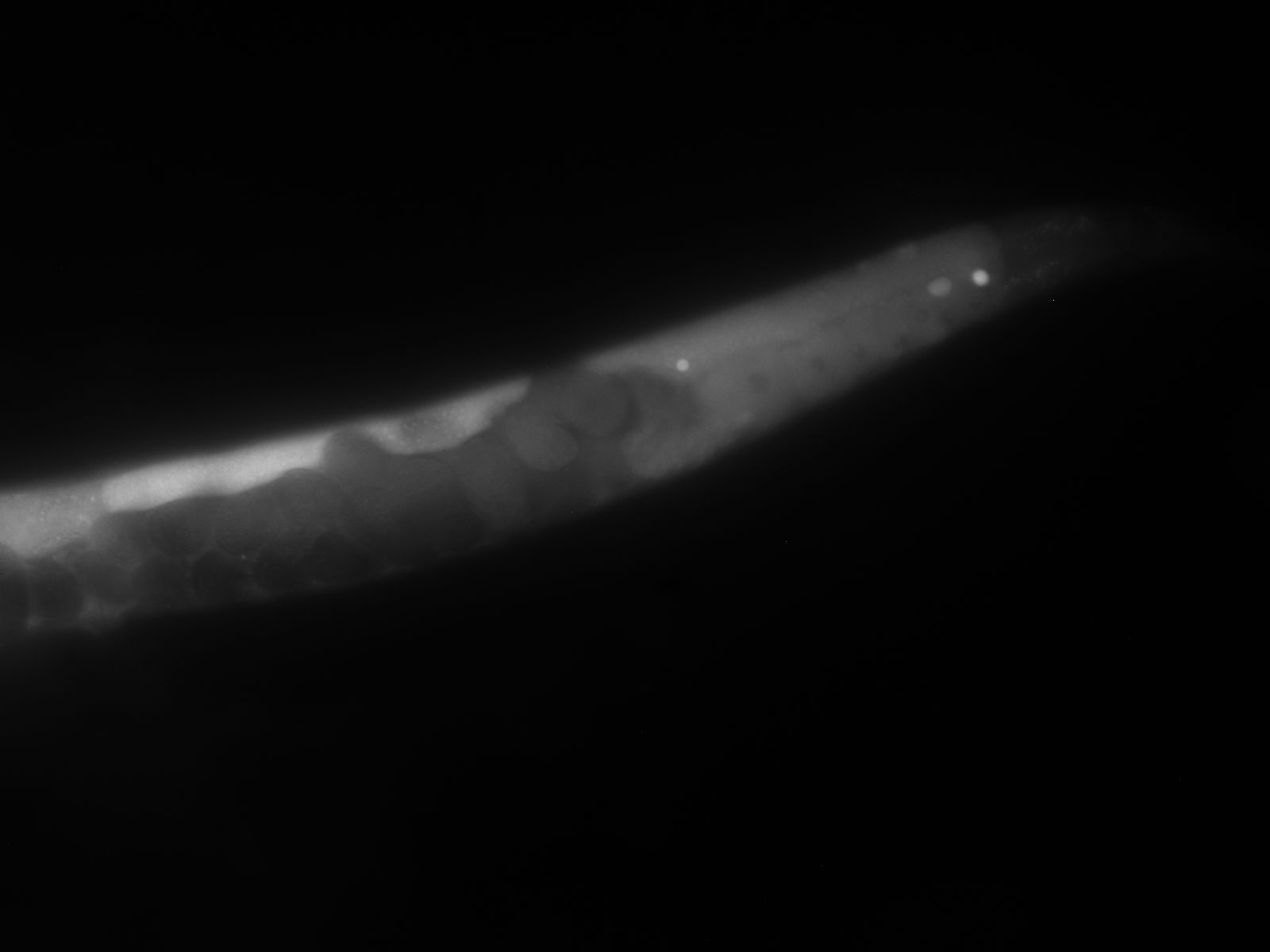

Supplement: S3 File — (ZIP) [file pgen.1011061.s003.zip › Fig.4D - Original files/Fig.4D RAW data and photos JPEG/syto12 staining - Fig 4D - 2 rep_15.5.23 jpeg/daf-28_tm2308+ tfg-128.jpg]

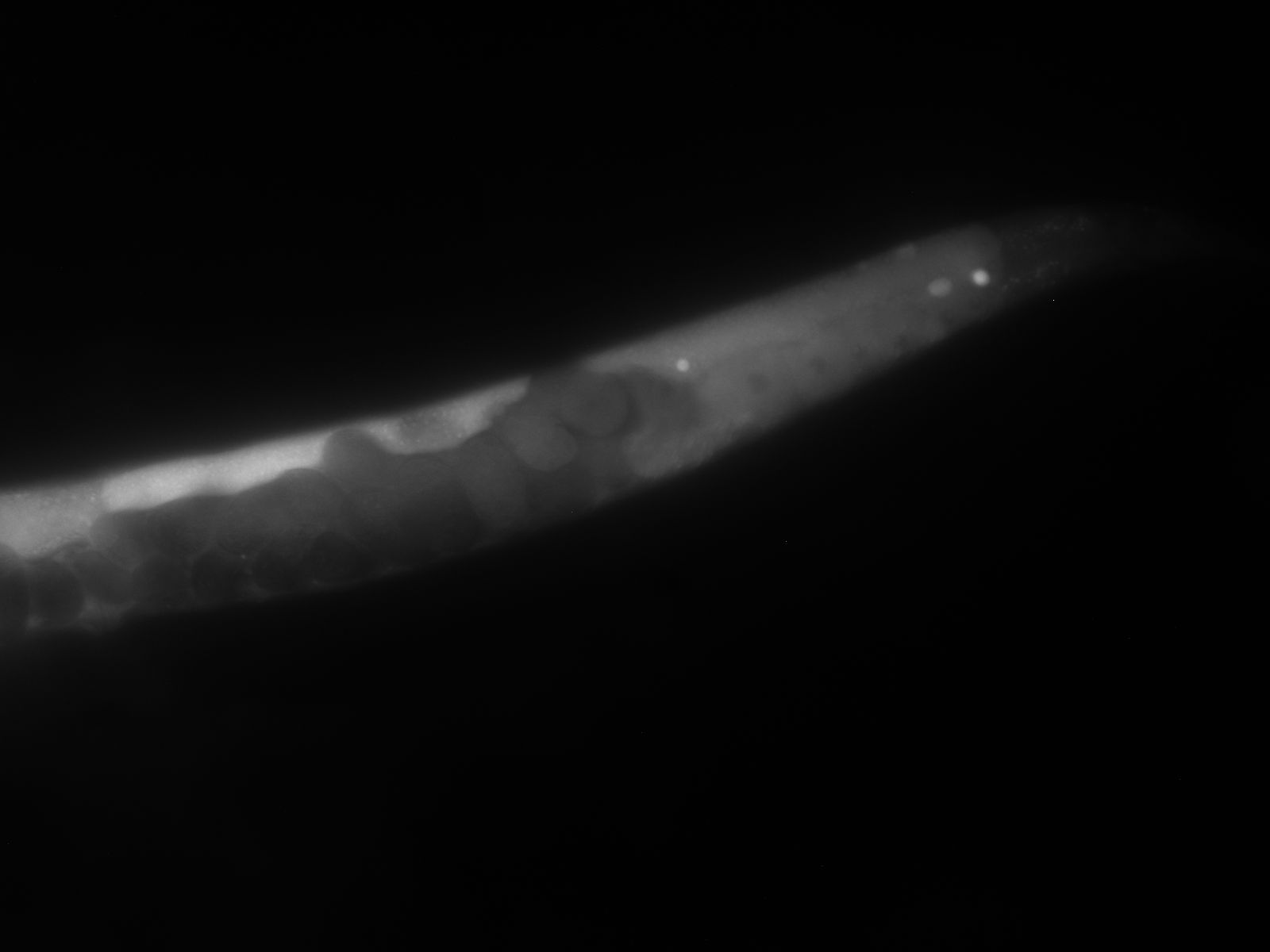

Supplement: S3 File — (ZIP) [file pgen.1011061.s003.zip › Fig.4D - Original files/Fig.4D RAW data and photos JPEG/syto12 staining - Fig 4D - 2 rep_15.5.23 jpeg/daf-28_tm2308+ tfg-129.jpg]

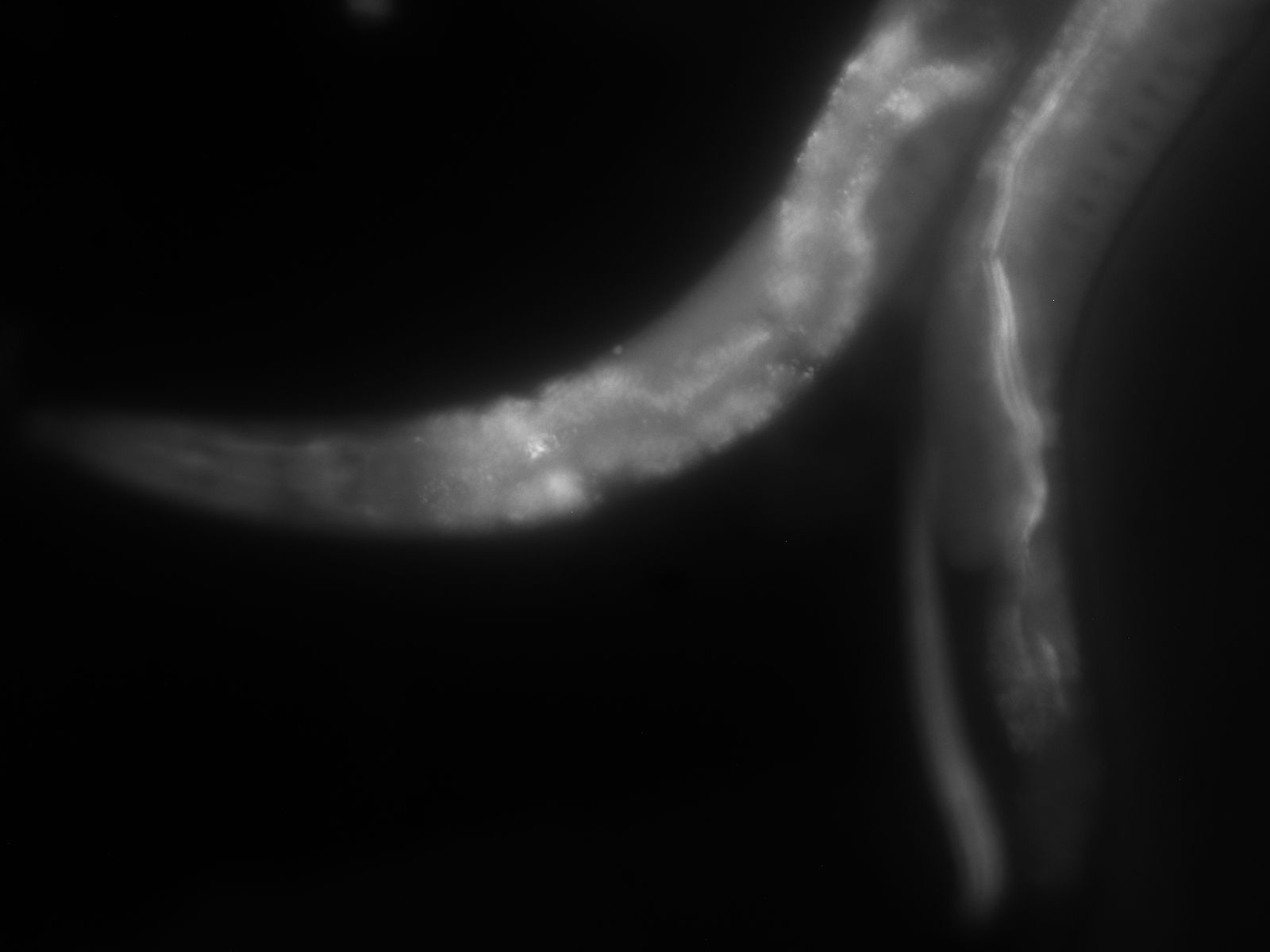

Supplement: S3 File — (ZIP) [file pgen.1011061.s003.zip › Fig.4D - Original files/Fig.4D RAW data and photos JPEG/syto12 staining - Fig 4D - 2 rep_15.5.23 jpeg/daf-28_tm2308+pad1215.jpg]

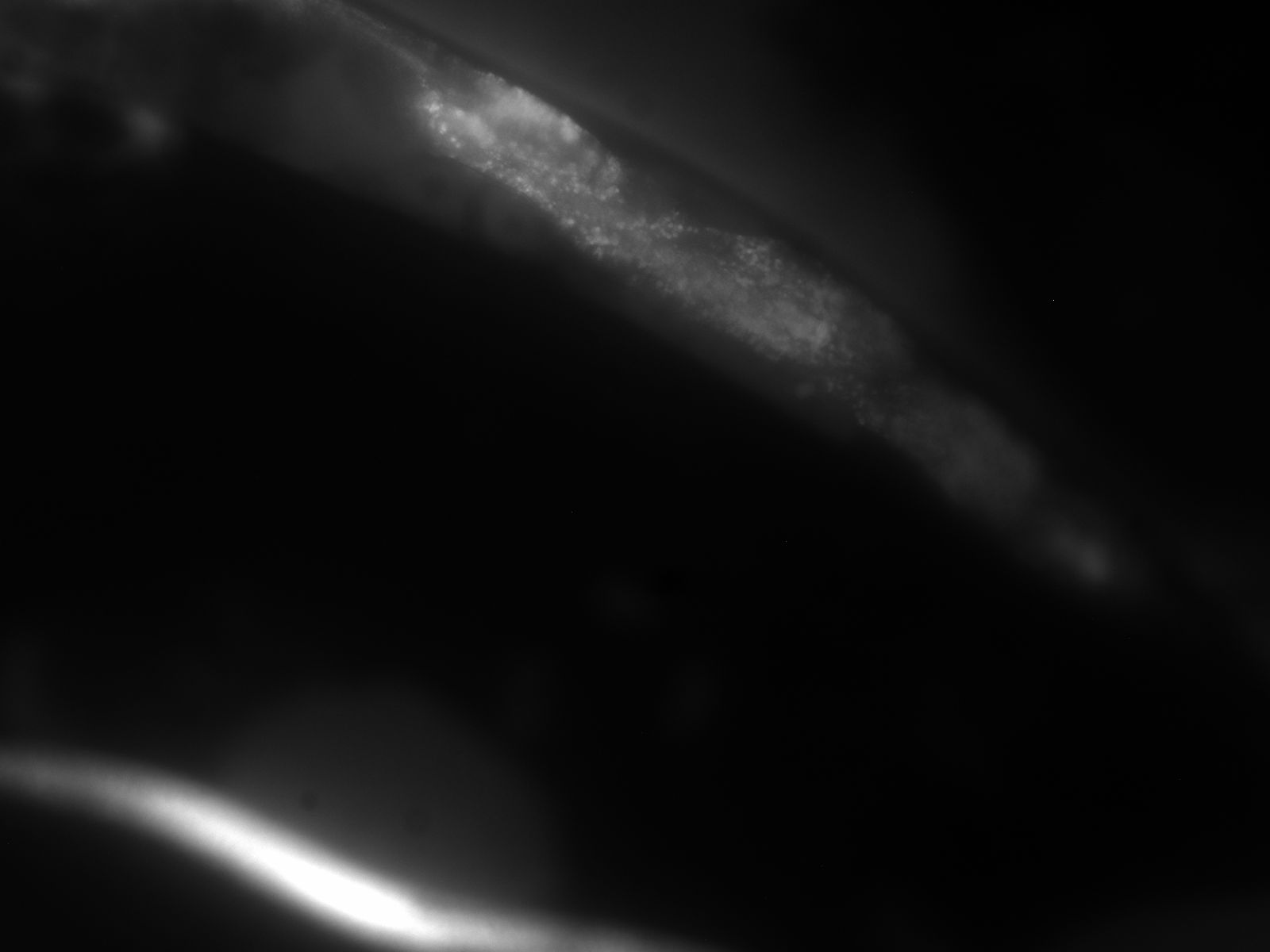

Supplement: S3 File — (ZIP) [file pgen.1011061.s003.zip › Fig.4D - Original files/Fig.4D RAW data and photos JPEG/syto12 staining - Fig 4D - 2 rep_15.5.23 jpeg/daf-28_tm2308+pad1216.jpg]

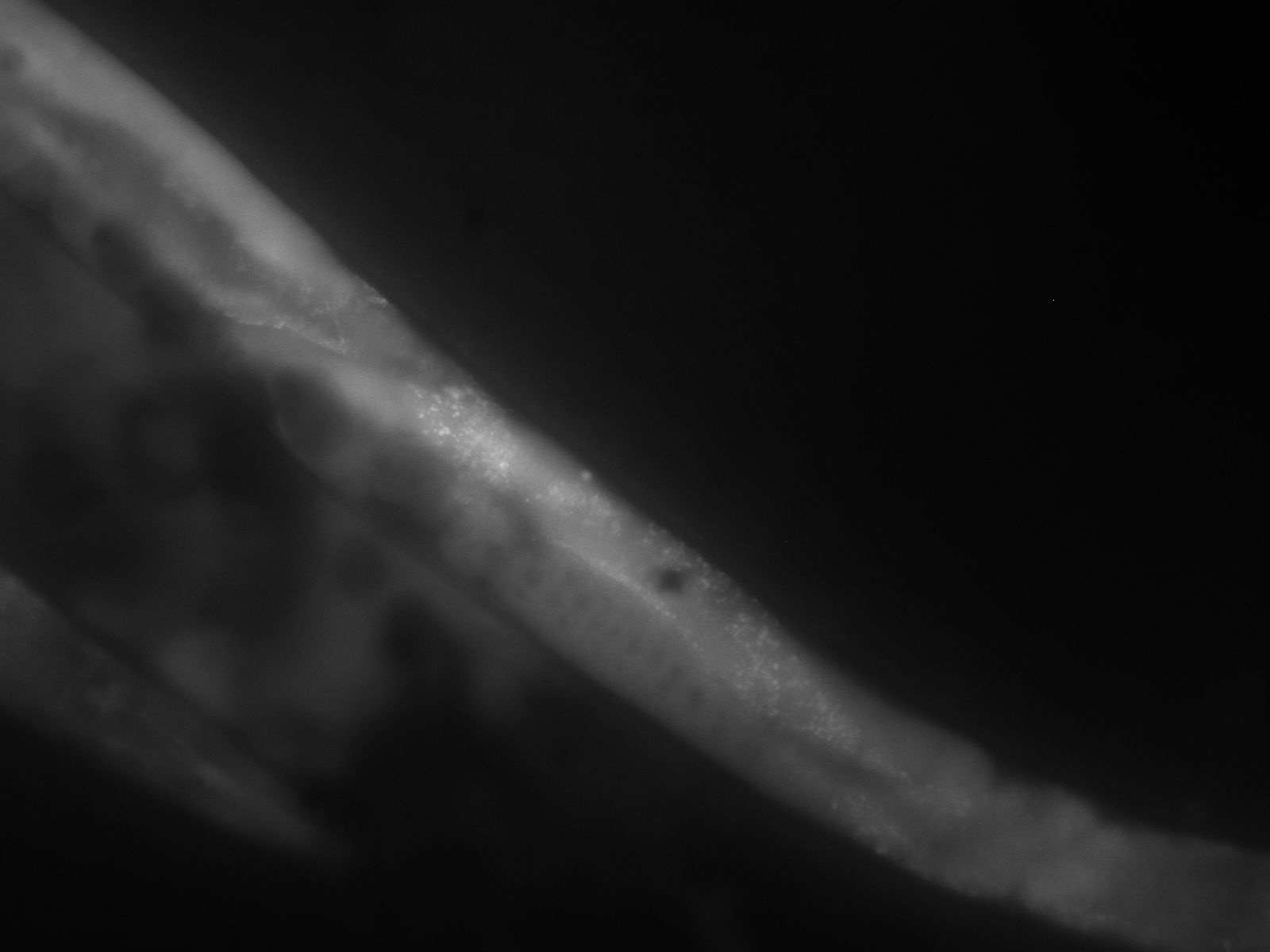

Supplement: S3 File — (ZIP) [file pgen.1011061.s003.zip › Fig.4D - Original files/Fig.4D RAW data and photos JPEG/syto12 staining - Fig 4D - 2 rep_15.5.23 jpeg/daf-28_tm2308+pad1217.jpg]

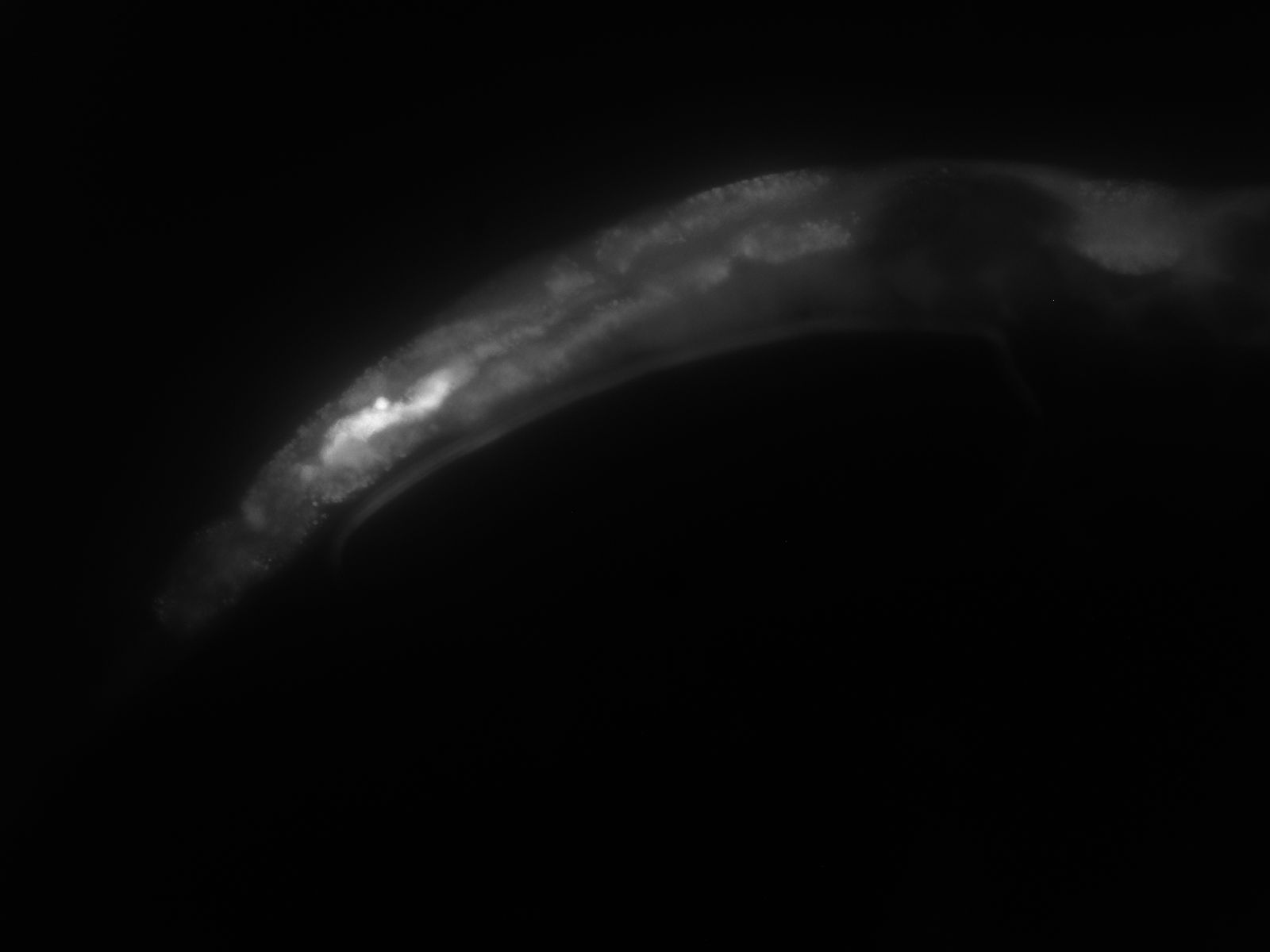

Supplement: S3 File — (ZIP) [file pgen.1011061.s003.zip › Fig.4D - Original files/Fig.4D RAW data and photos JPEG/syto12 staining - Fig 4D - 2 rep_15.5.23 jpeg/daf-28_tm2308+pad1218.jpg]

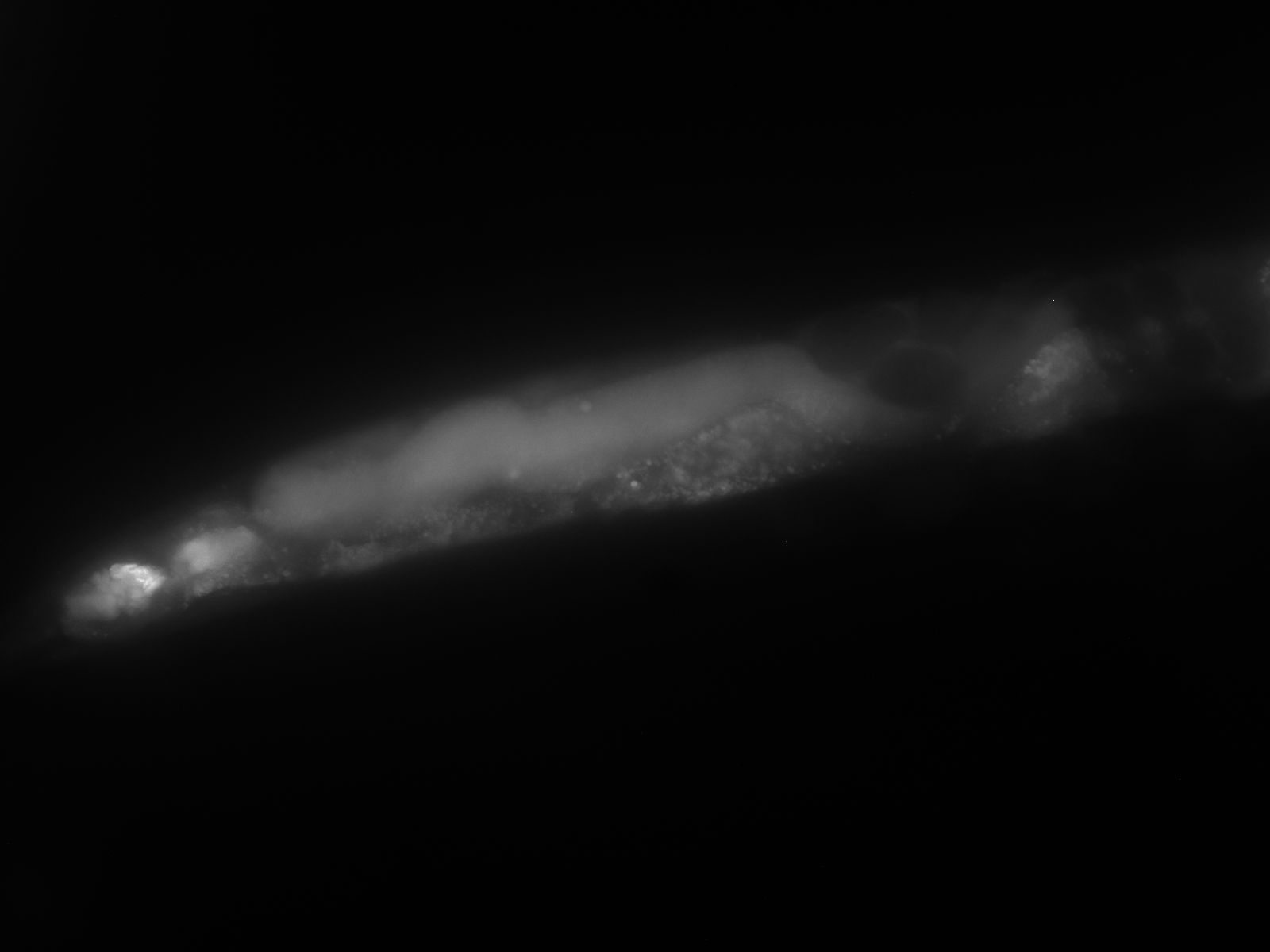

Supplement: S3 File — (ZIP) [file pgen.1011061.s003.zip › Fig.4D - Original files/Fig.4D RAW data and photos JPEG/syto12 staining - Fig 4D - 2 rep_15.5.23 jpeg/daf-28_tm2308+pad1219.jpg]

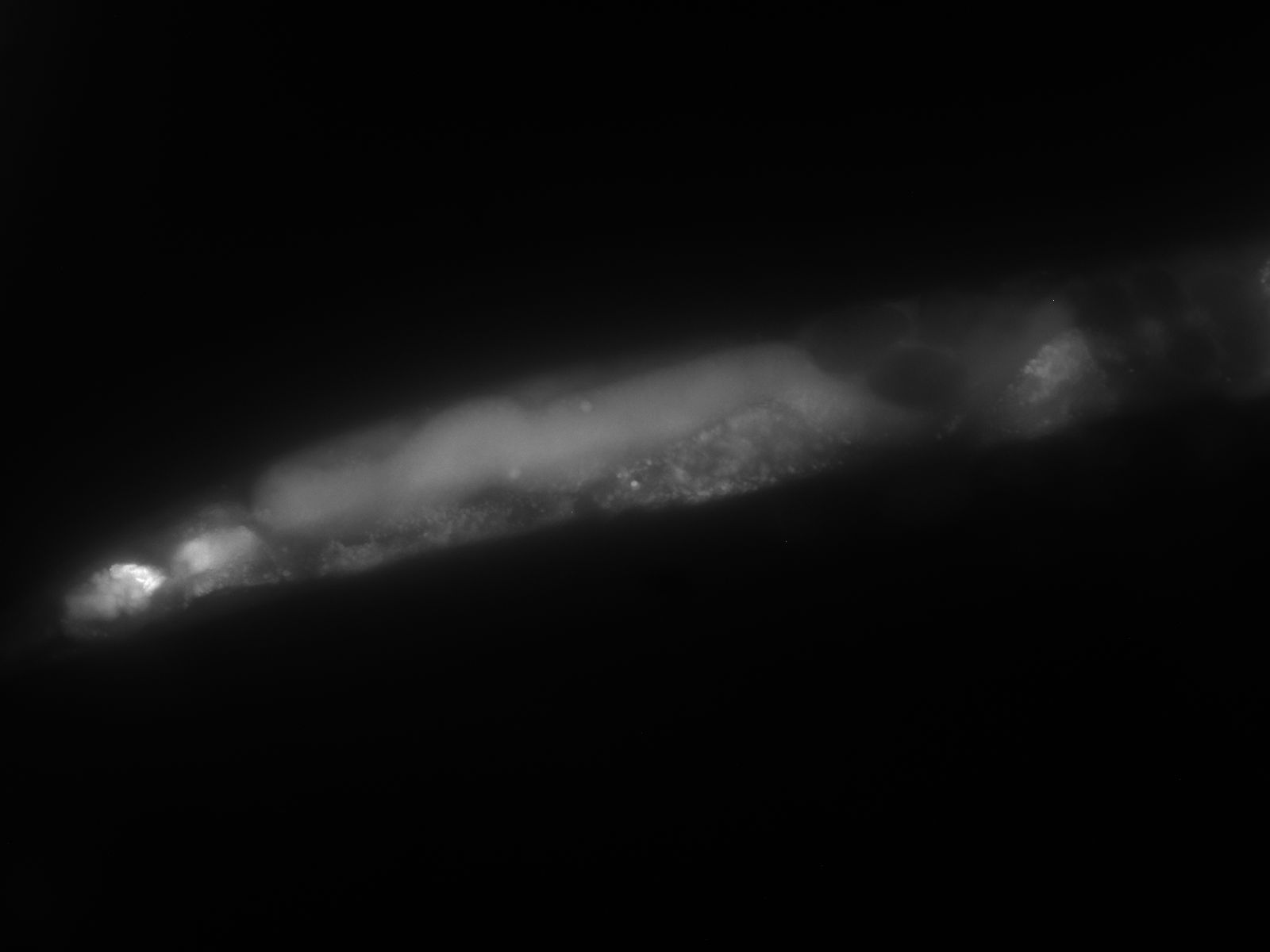

Supplement: S3 File — (ZIP) [file pgen.1011061.s003.zip › Fig.4D - Original files/Fig.4D RAW data and photos JPEG/syto12 staining - Fig 4D - 2 rep_15.5.23 jpeg/daf-28_tm2308+pad1220.jpg]

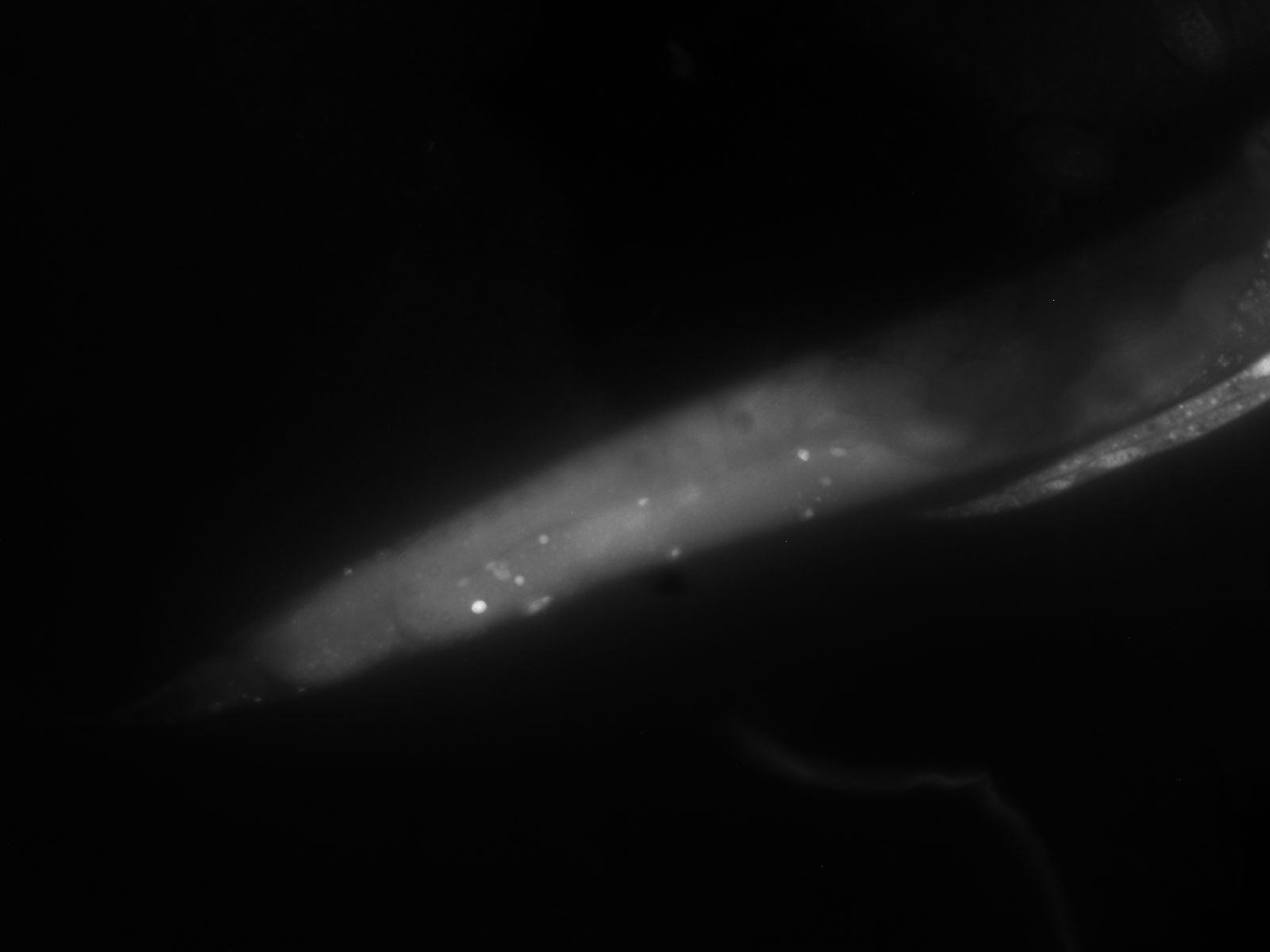

Supplement: S3 File — (ZIP) [file pgen.1011061.s003.zip › Fig.4D - Original files/Fig.4D RAW data and photos JPEG/syto12 staining - Fig 4D - 3_rep - 22.5.23 jpeg/daf-28_sa+pad1237.jpg]

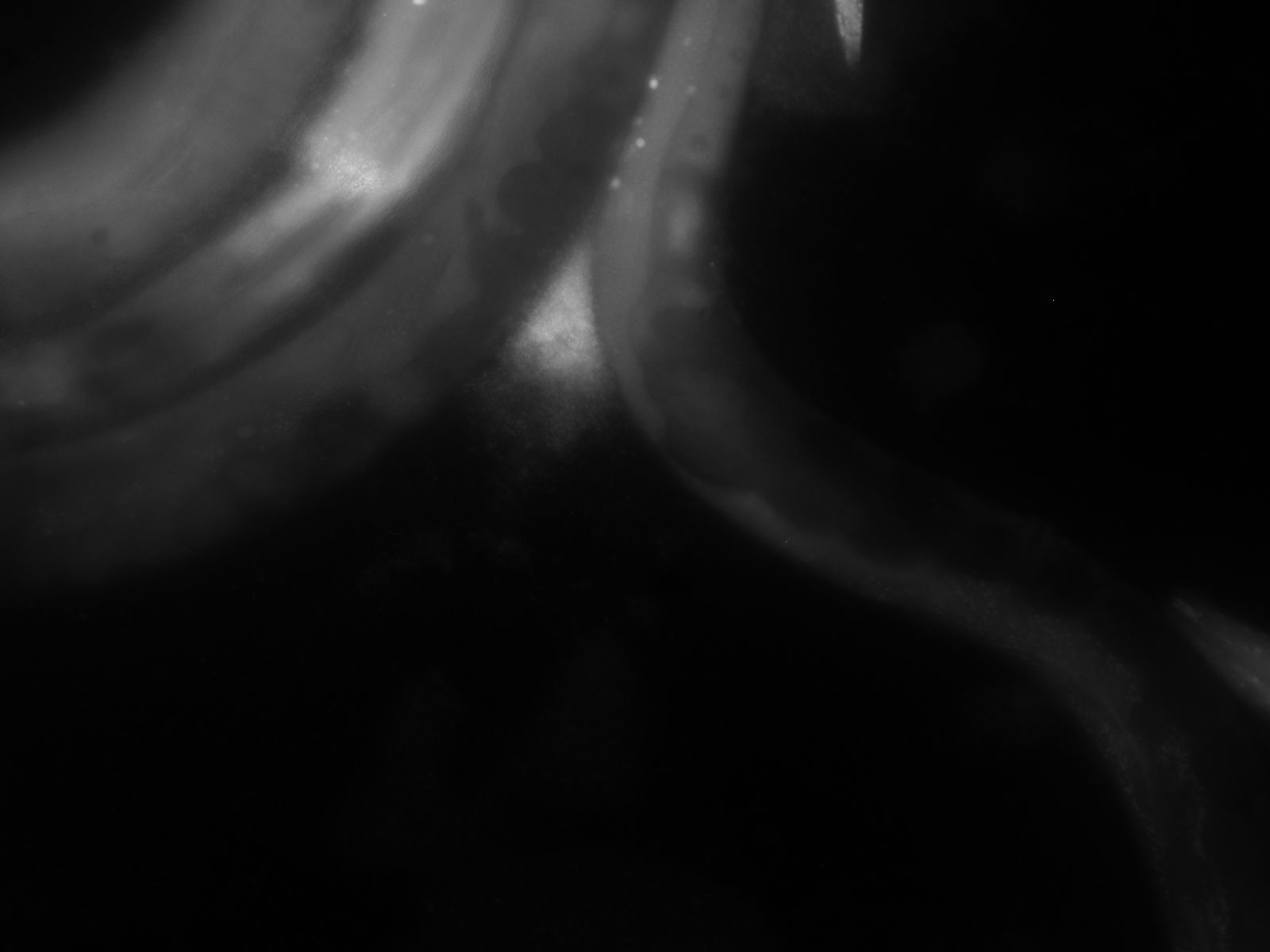

Supplement: S3 File — (ZIP) [file pgen.1011061.s003.zip › Fig.4D - Original files/Fig.4D RAW data and photos JPEG/syto12 staining - Fig 4D - 3_rep - 22.5.23 jpeg/daf-28_sa+pad1238.jpg]

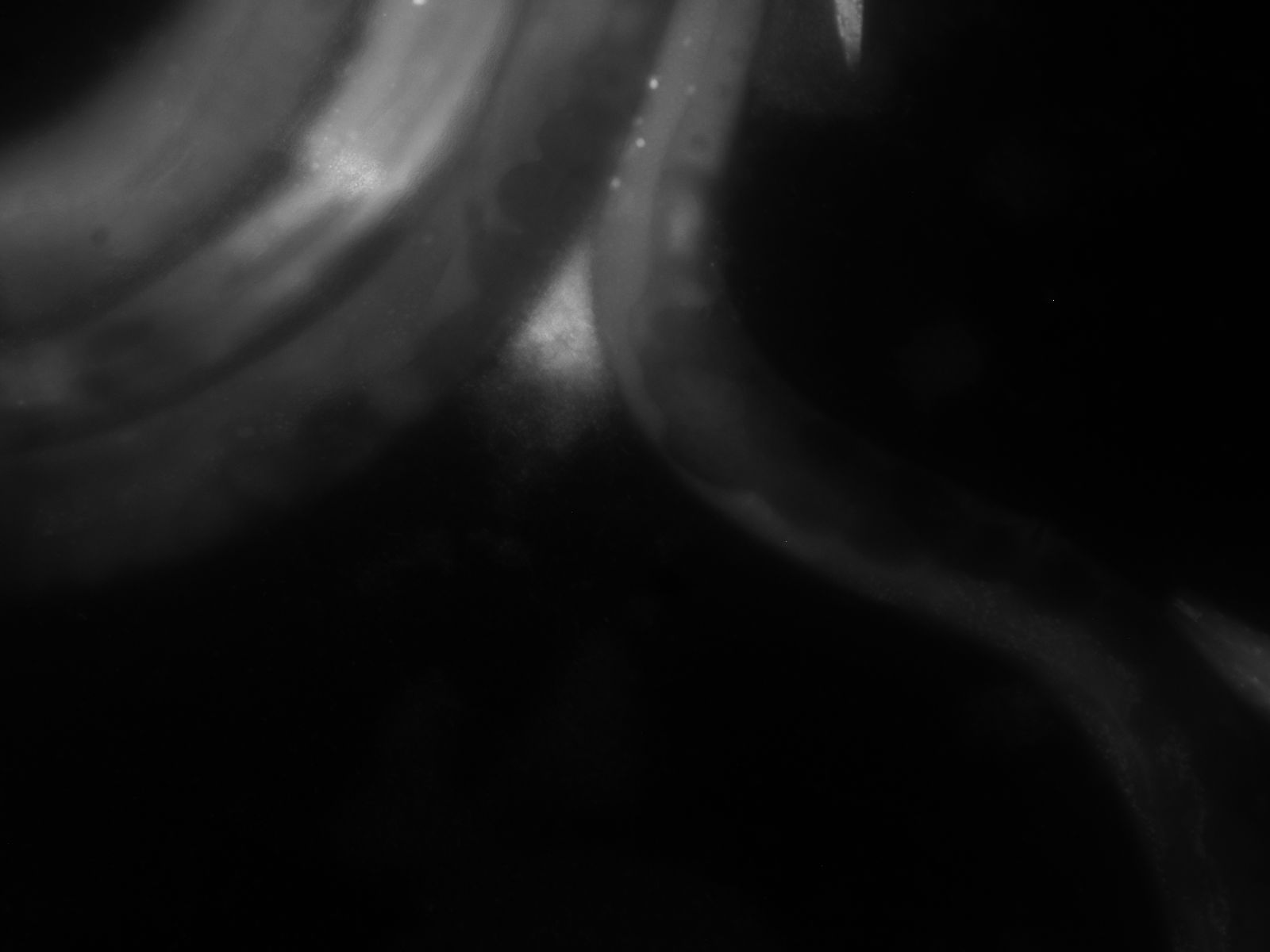

Supplement: S3 File — (ZIP) [file pgen.1011061.s003.zip › Fig.4D - Original files/Fig.4D RAW data and photos JPEG/syto12 staining - Fig 4D - 3_rep - 22.5.23 jpeg/daf-28_sa+pad1239.jpg]

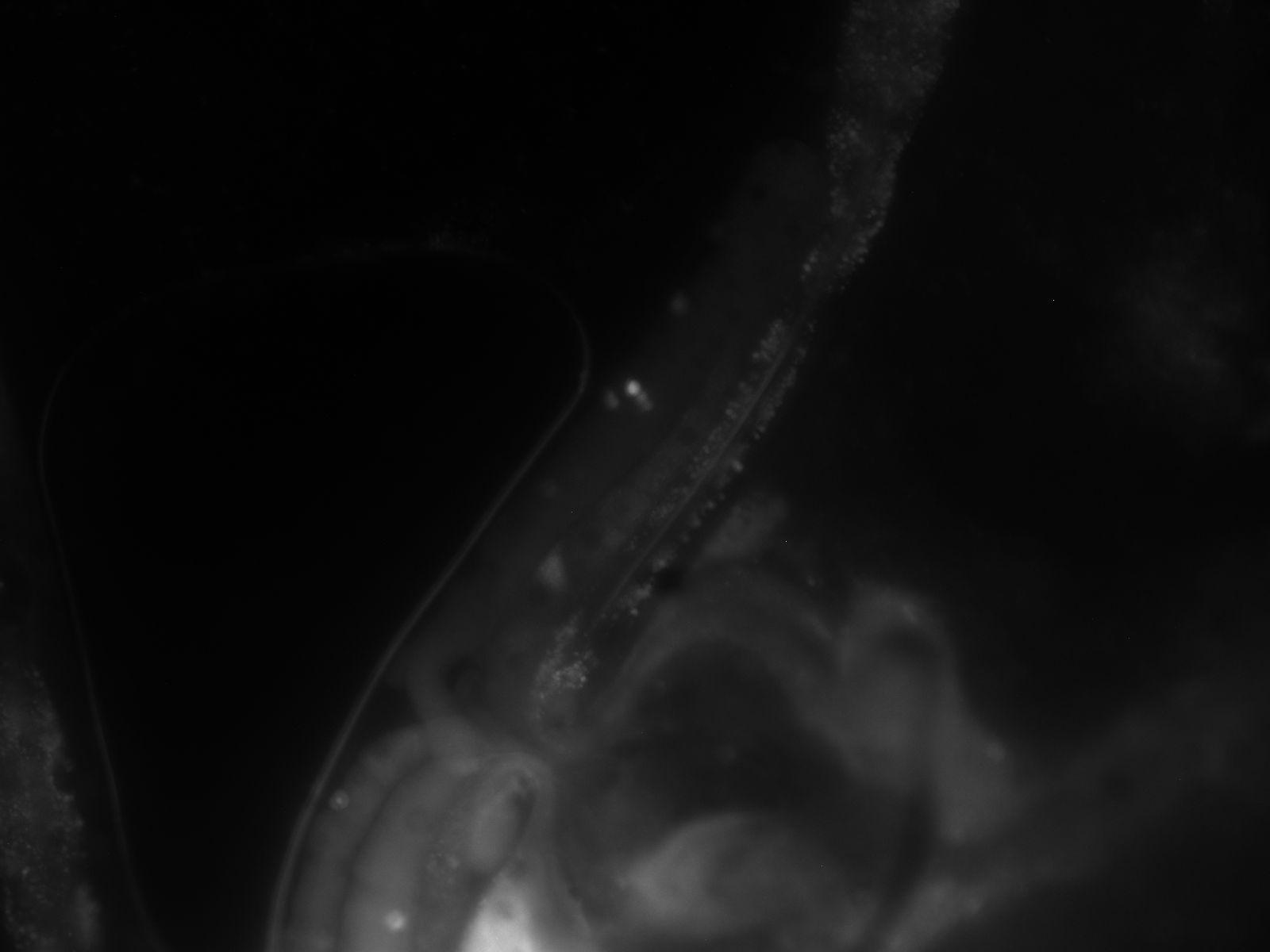

Supplement: S3 File — (ZIP) [file pgen.1011061.s003.zip › Fig.4D - Original files/Fig.4D RAW data and photos JPEG/syto12 staining - Fig 4D - 3_rep - 22.5.23 jpeg/daf-28_sa+tfg-140.jpg]

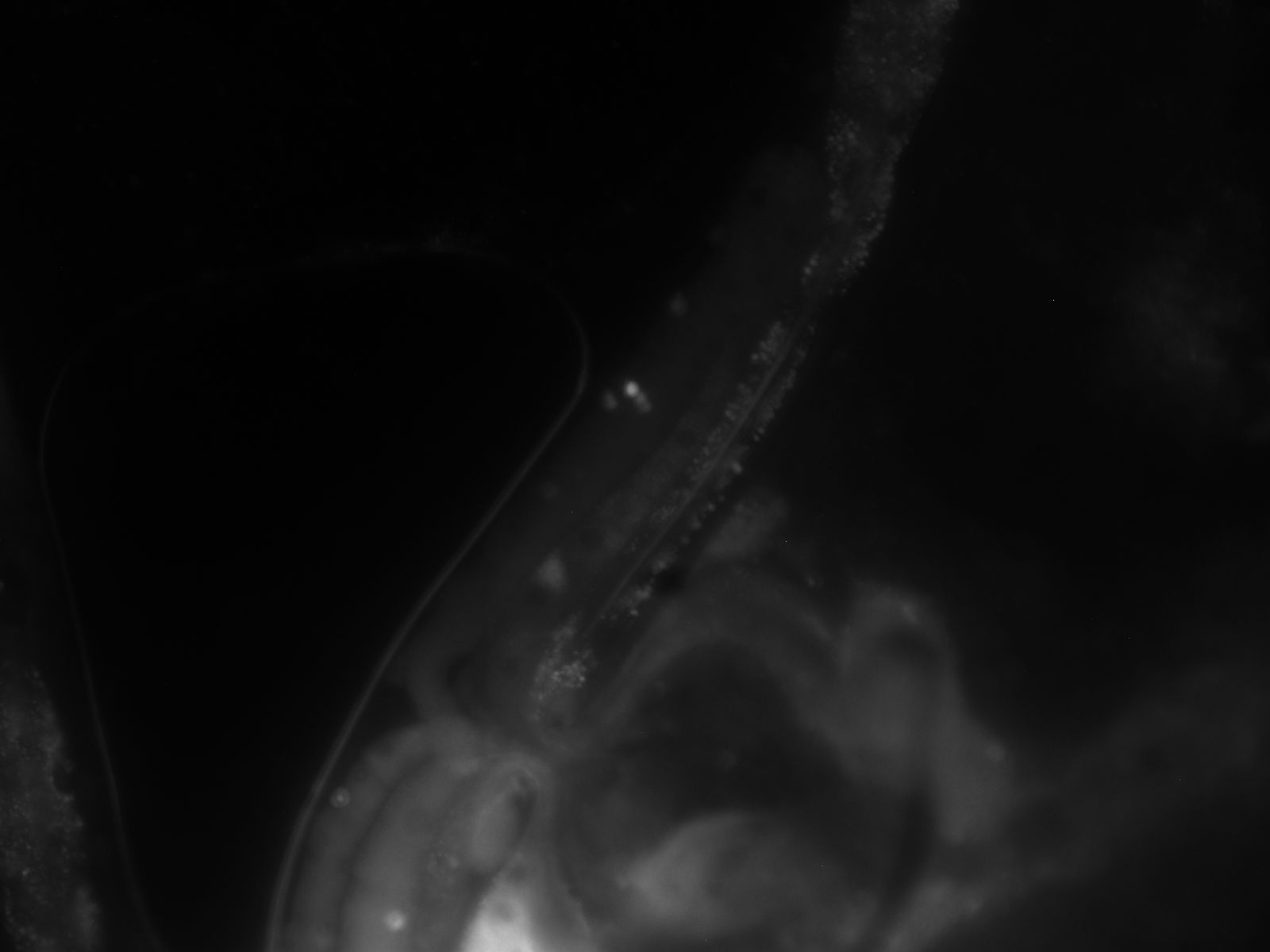

Supplement: S3 File — (ZIP) [file pgen.1011061.s003.zip › Fig.4D - Original files/Fig.4D RAW data and photos JPEG/syto12 staining - Fig 4D - 3_rep - 22.5.23 jpeg/daf-28_sa+tfg-141.jpg]

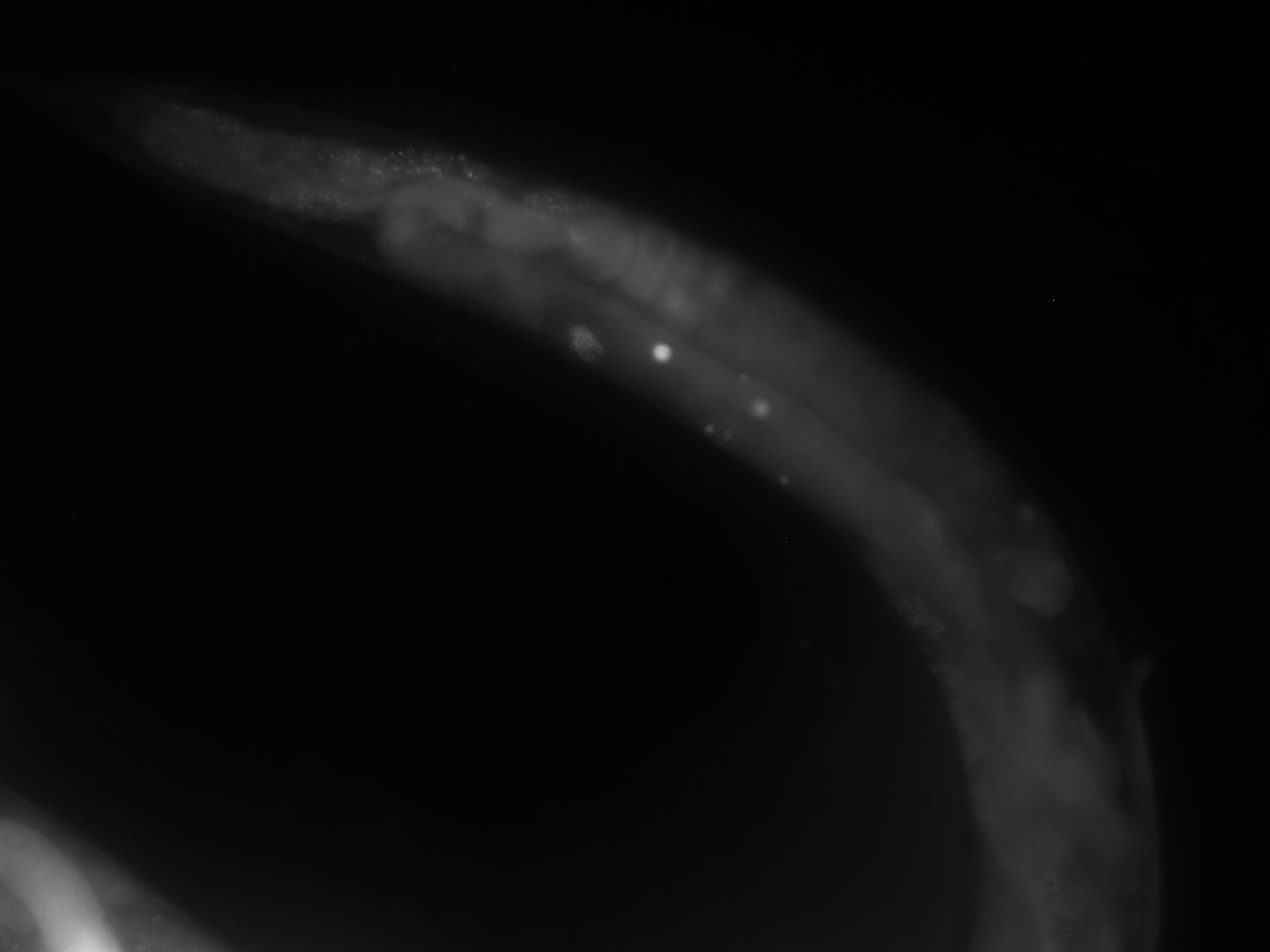

Supplement: S3 File — (ZIP) [file pgen.1011061.s003.zip › Fig.4D - Original files/Fig.4D RAW data and photos JPEG/syto12 staining - Fig 4D - 3_rep - 22.5.23 jpeg/daf-28_sa+tfg-142.jpg]

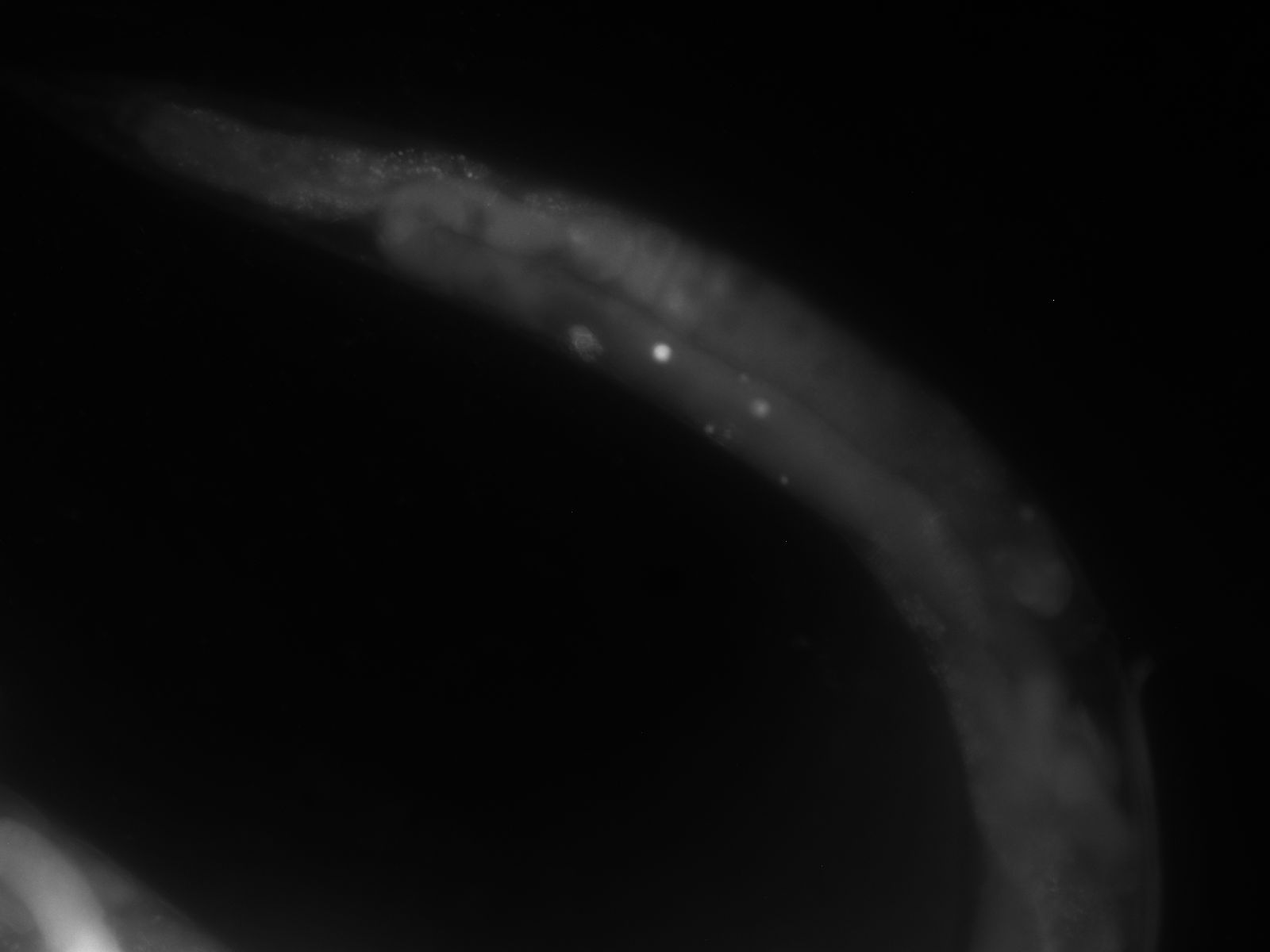

Supplement: S3 File — (ZIP) [file pgen.1011061.s003.zip › Fig.4D - Original files/Fig.4D RAW data and photos JPEG/syto12 staining - Fig 4D - 3_rep - 22.5.23 jpeg/daf-28_sa+tfg-143.jpg]

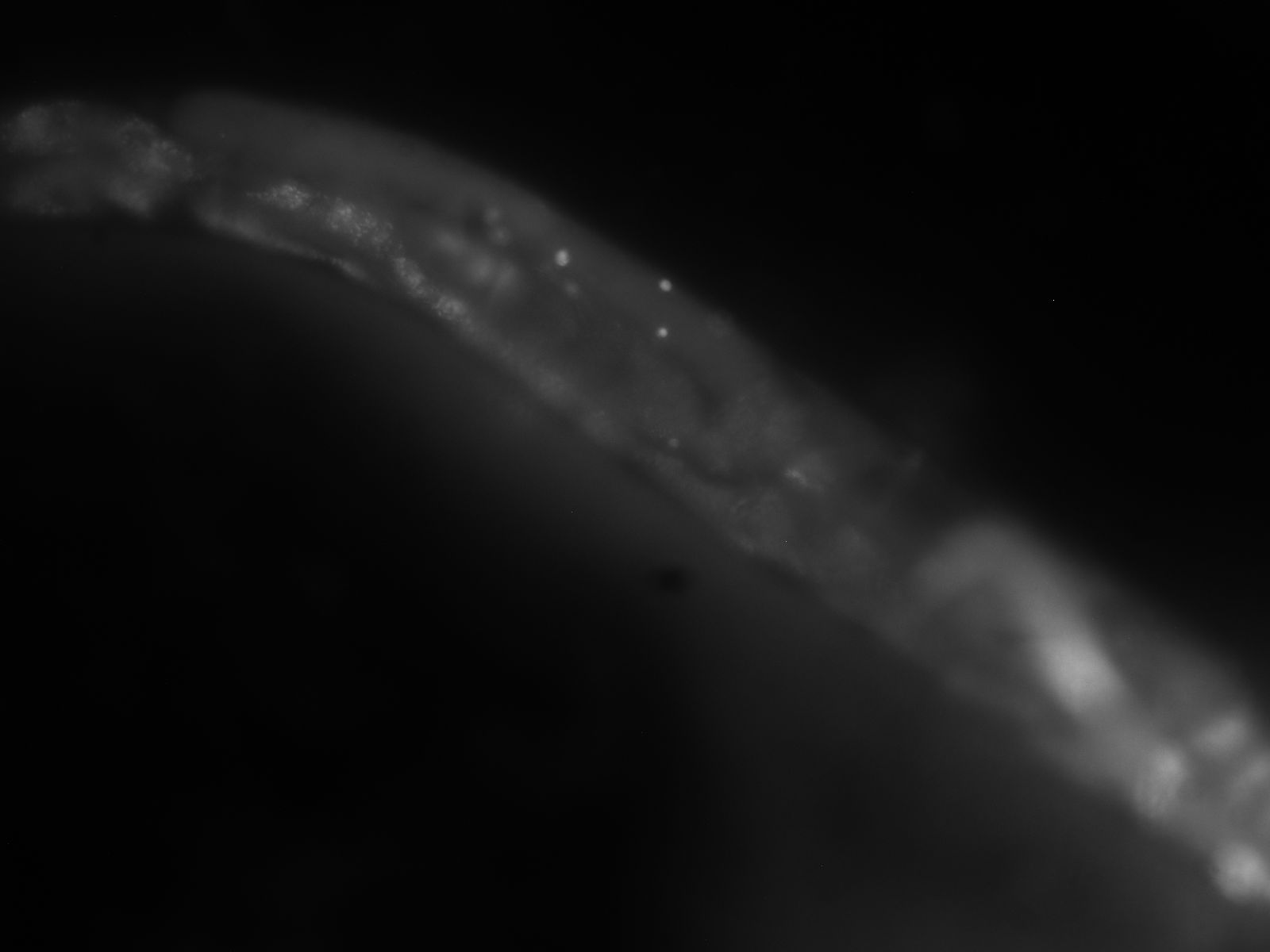

Supplement: S3 File — (ZIP) [file pgen.1011061.s003.zip › Fig.4D - Original files/Fig.4D RAW data and photos JPEG/syto12 staining - Fig 4D - 3_rep - 22.5.23 jpeg/daf-28_sa+tfg-144.jpg]

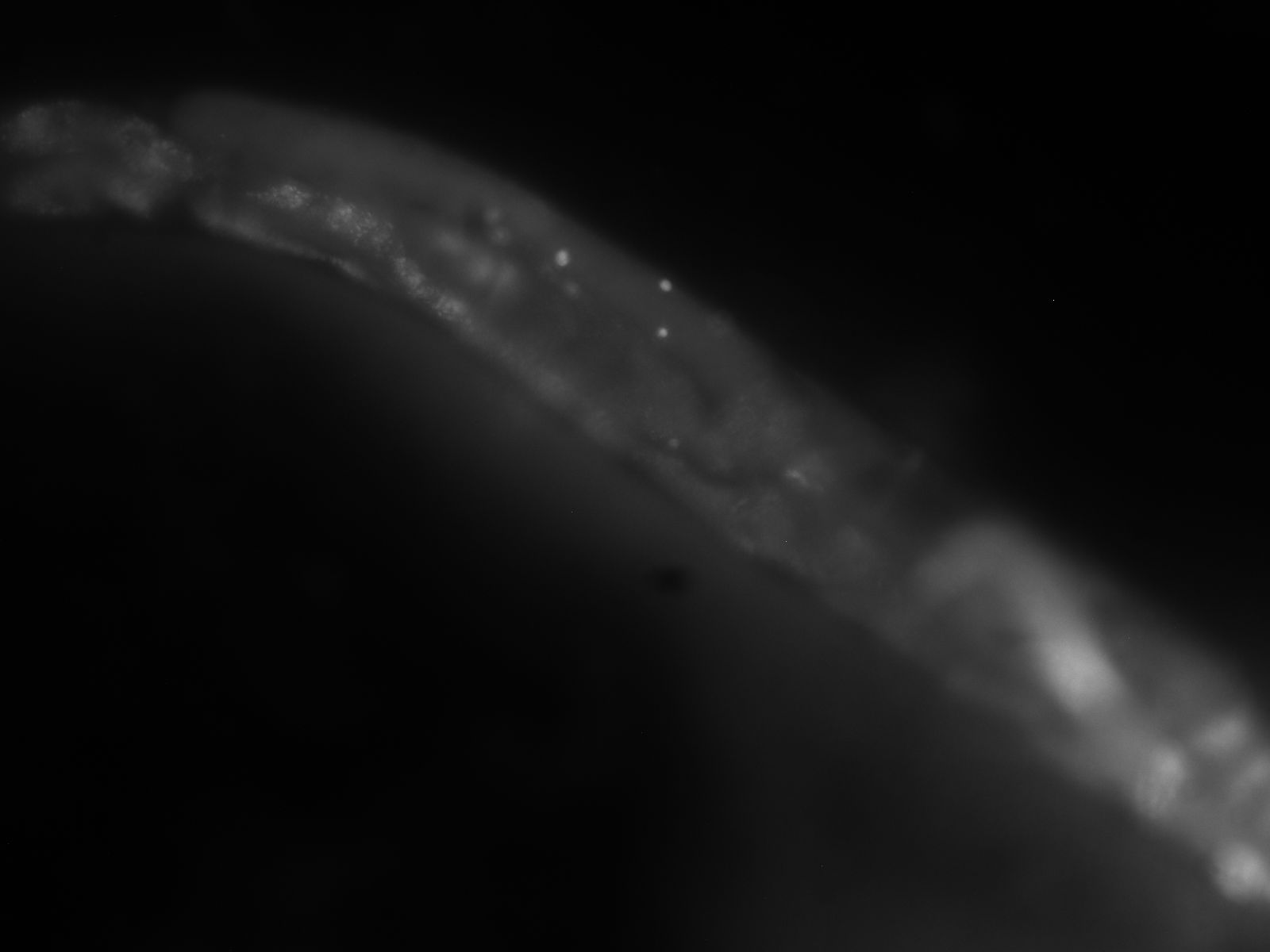

Supplement: S3 File — (ZIP) [file pgen.1011061.s003.zip › Fig.4D - Original files/Fig.4D RAW data and photos JPEG/syto12 staining - Fig 4D - 3_rep - 22.5.23 jpeg/daf-28_sa+tfg-145.jpg]

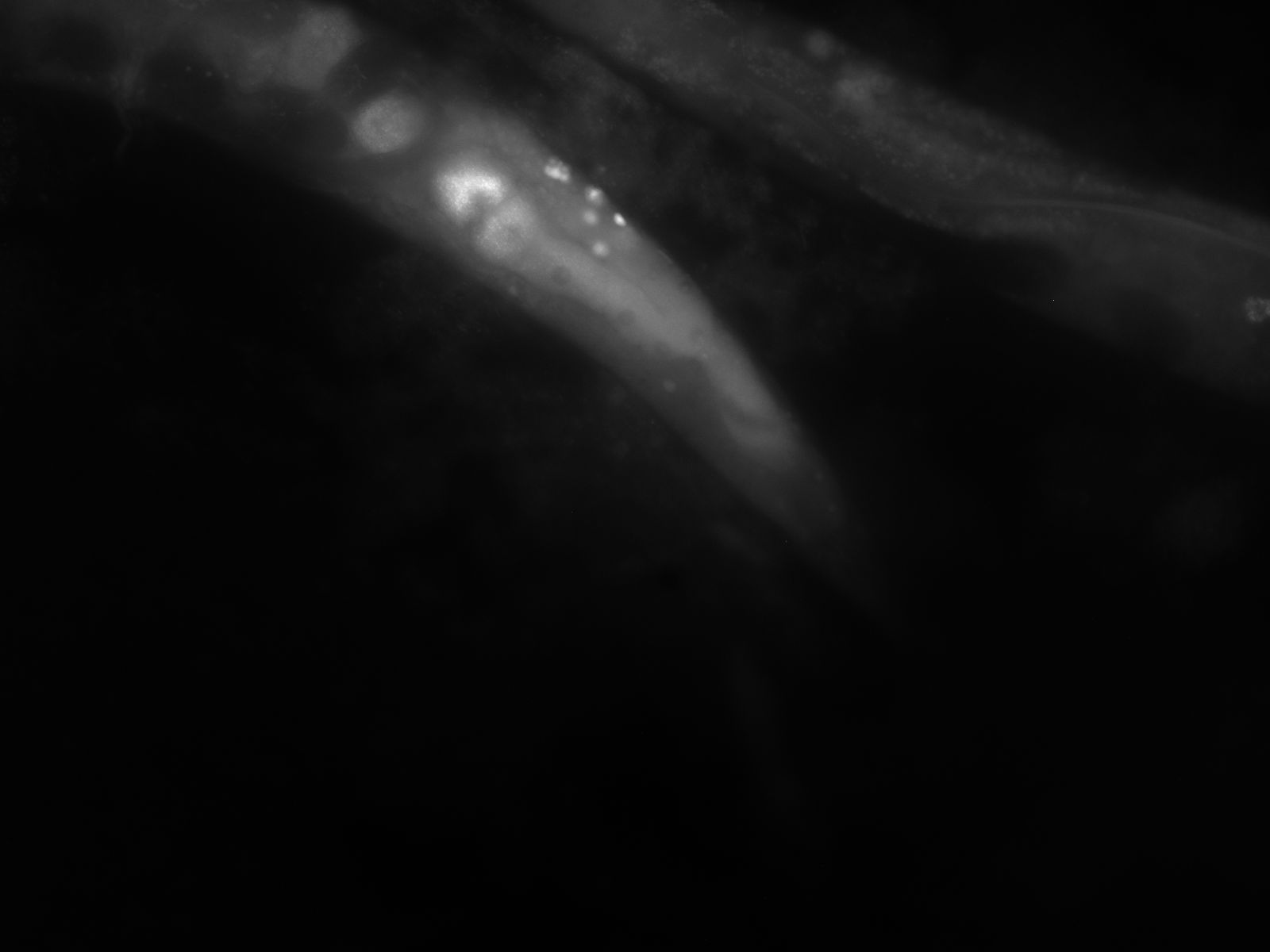

Supplement: S3 File — (ZIP) [file pgen.1011061.s003.zip › Fig.4D - Original files/Fig.4D RAW data and photos JPEG/syto12 staining - Fig 4D - 3_rep - 22.5.23 jpeg/daf-28_sa+tfg-146.jpg]

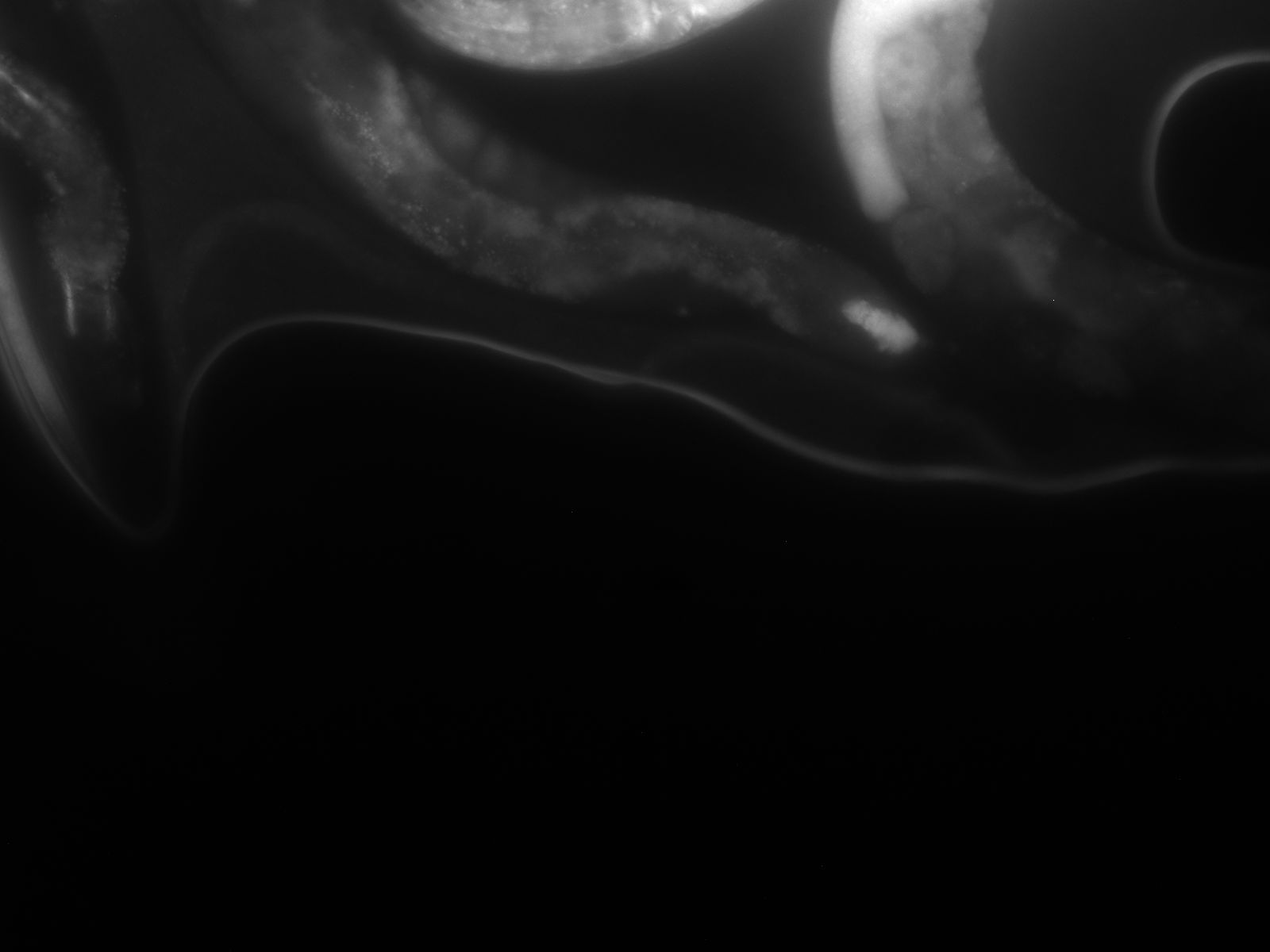

Supplement: S3 File — (ZIP) [file pgen.1011061.s003.zip › Fig.4D - Original files/Fig.4D RAW data and photos JPEG/syto12 staining - Fig 4D - 3_rep - 22.5.23 jpeg/daf-28_tm2308+pad1227.jpg]

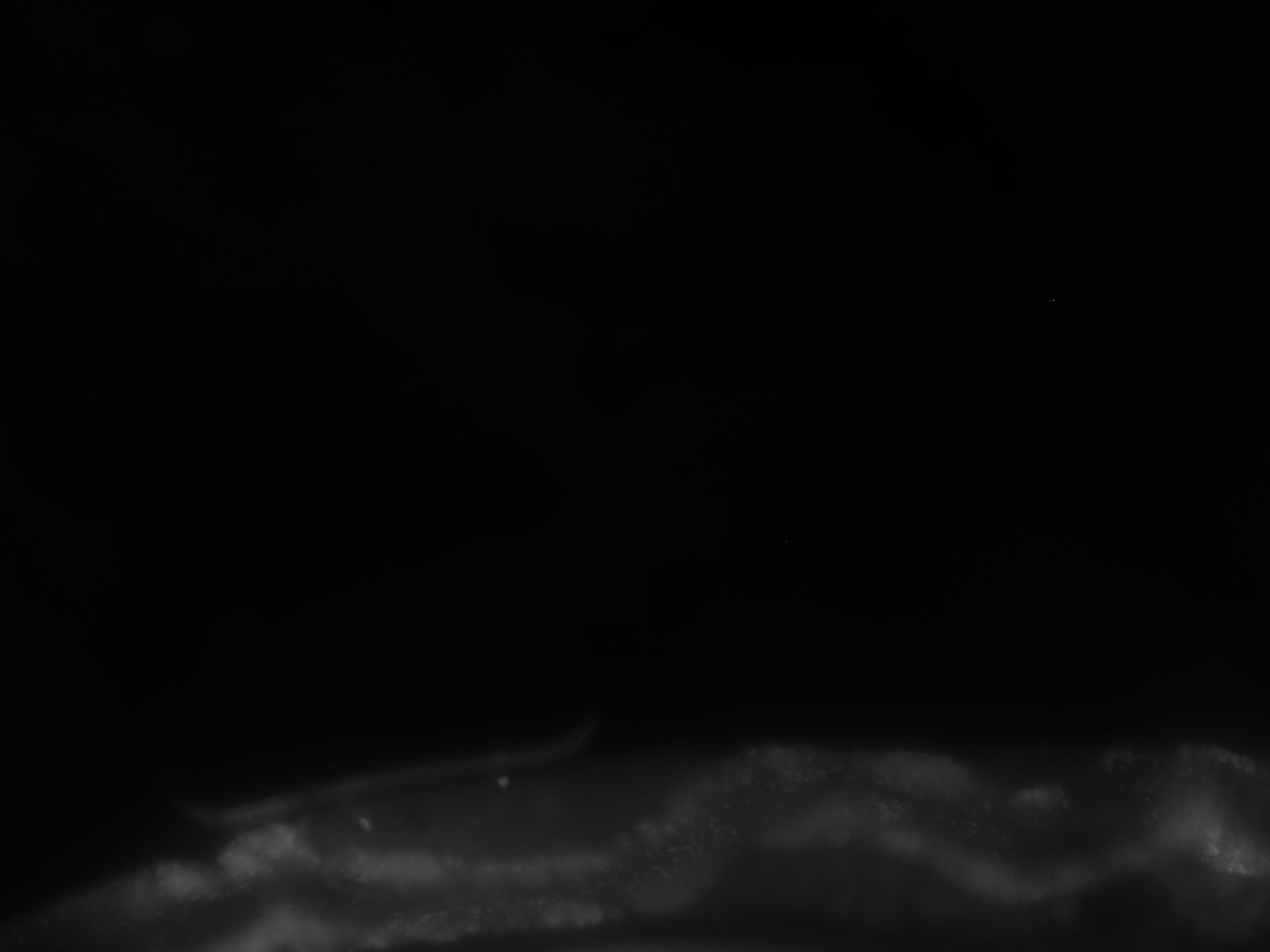

Supplement: S3 File — (ZIP) [file pgen.1011061.s003.zip › Fig.4D - Original files/Fig.4D RAW data and photos JPEG/syto12 staining - Fig 4D - 3_rep - 22.5.23 jpeg/daf-28_tm2308+pad1228.jpg]

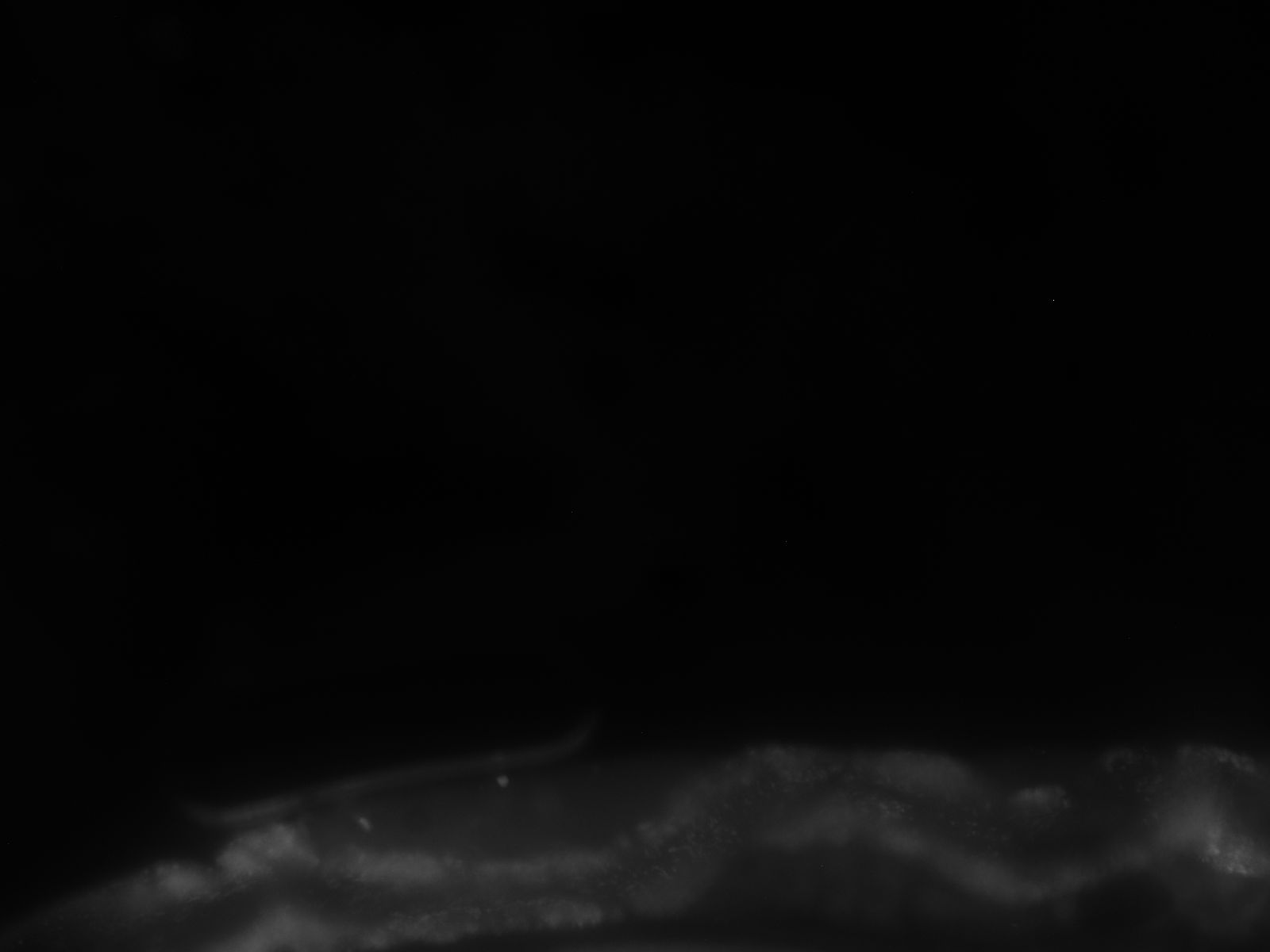

Supplement: S3 File — (ZIP) [file pgen.1011061.s003.zip › Fig.4D - Original files/Fig.4D RAW data and photos JPEG/syto12 staining - Fig 4D - 3_rep - 22.5.23 jpeg/daf-28_tm2308+pad1229.jpg]

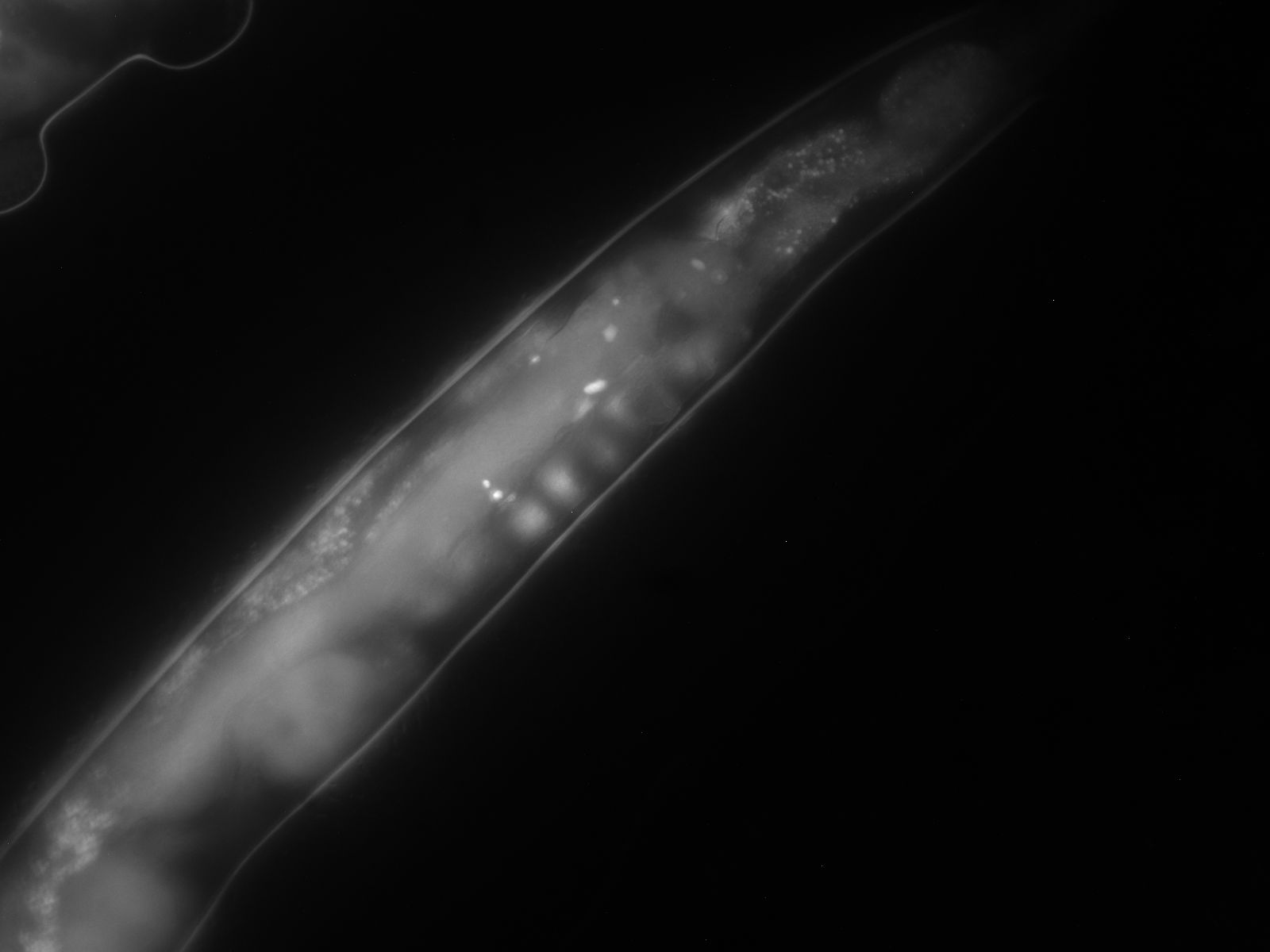

Supplement: S3 File — (ZIP) [file pgen.1011061.s003.zip › Fig.4D - Original files/Fig.4D RAW data and photos JPEG/syto12 staining - Fig 4D - 3_rep - 22.5.23 jpeg/daf-28_tm2308+tfg-130.jpg]

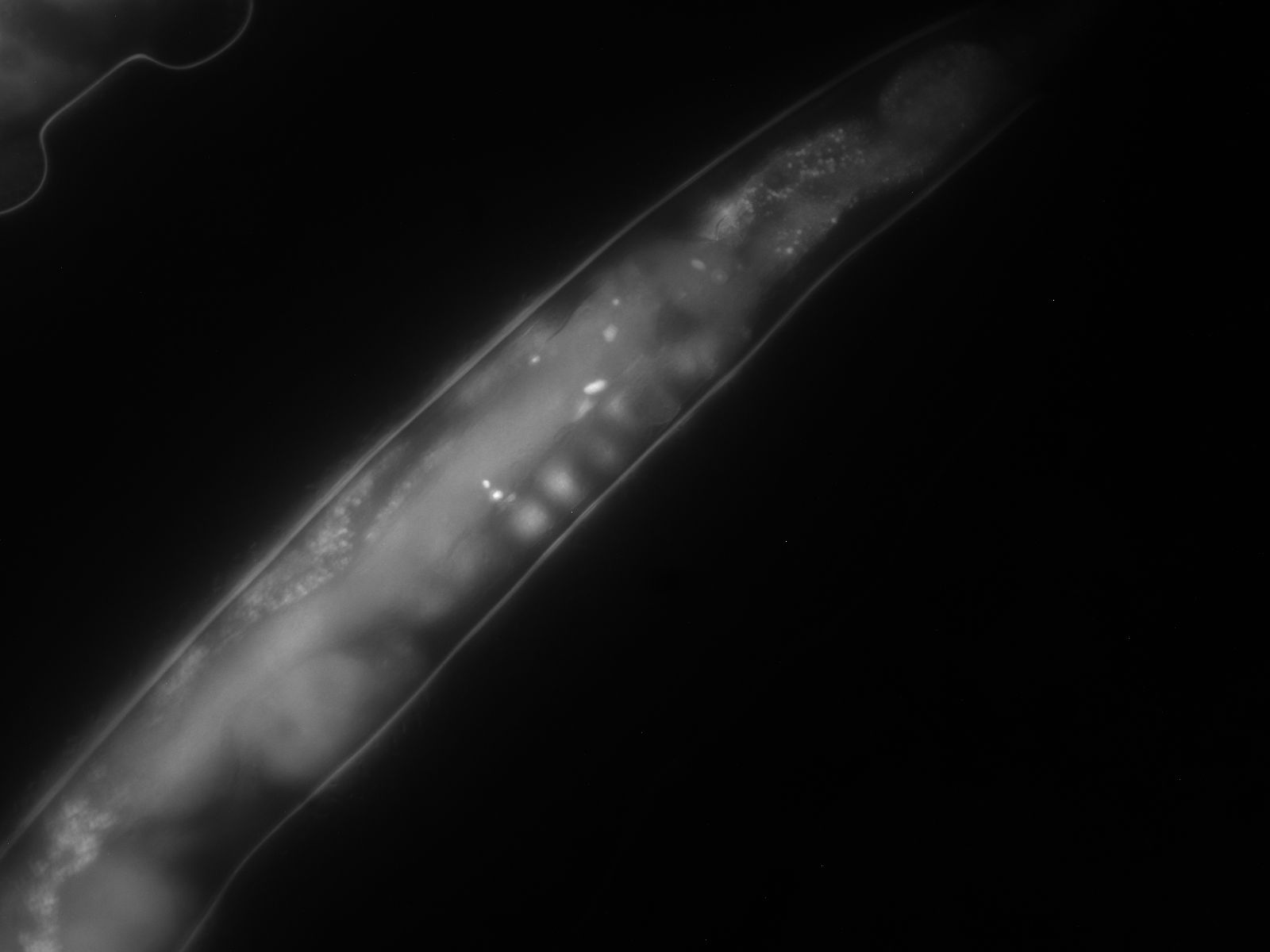

Supplement: S3 File — (ZIP) [file pgen.1011061.s003.zip › Fig.4D - Original files/Fig.4D RAW data and photos JPEG/syto12 staining - Fig 4D - 3_rep - 22.5.23 jpeg/daf-28_tm2308+tfg-131.jpg]

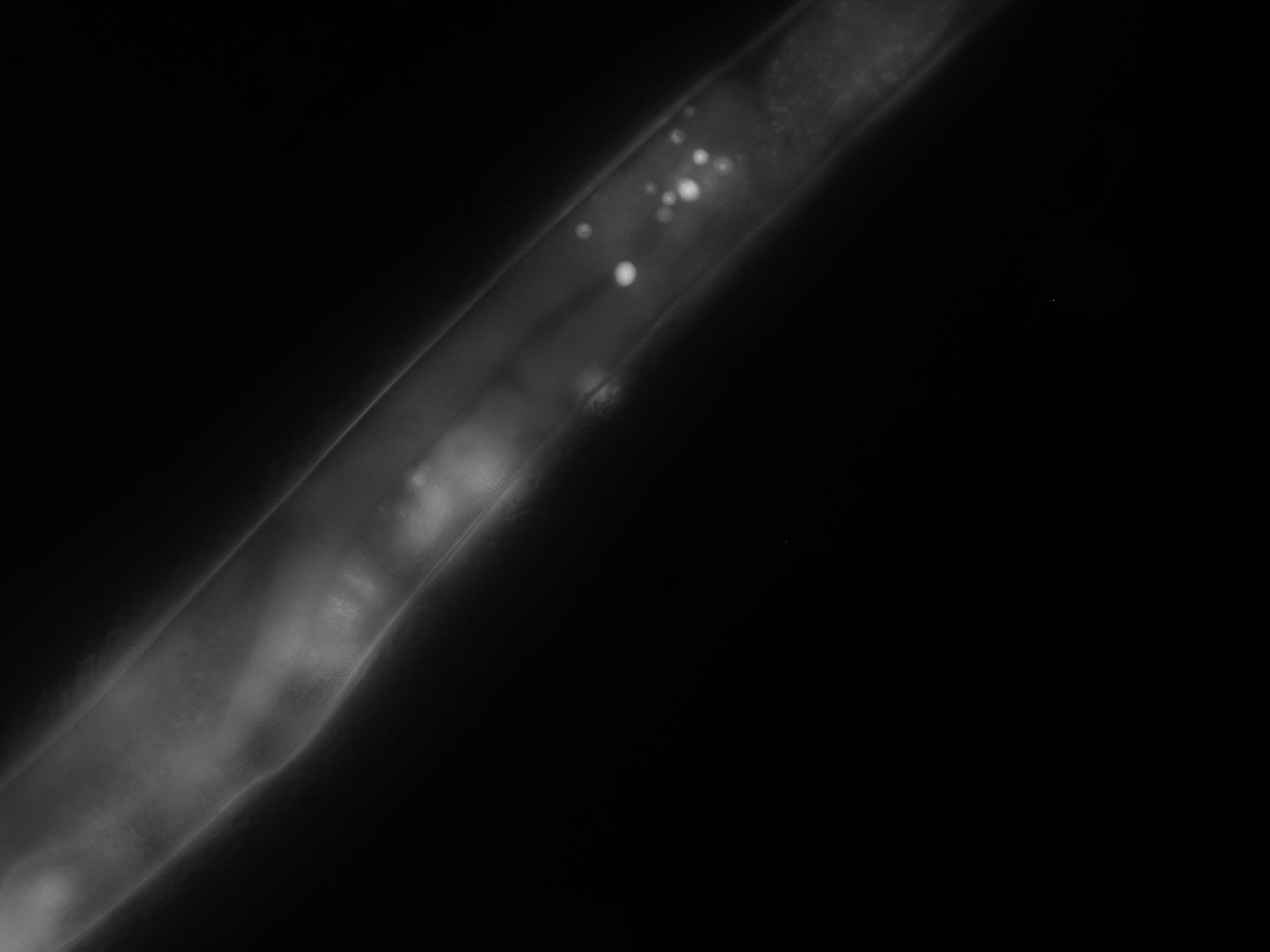

Supplement: S3 File — (ZIP) [file pgen.1011061.s003.zip › Fig.4D - Original files/Fig.4D RAW data and photos JPEG/syto12 staining - Fig 4D - 3_rep - 22.5.23 jpeg/daf-28_tm2308+tfg-132.jpg]

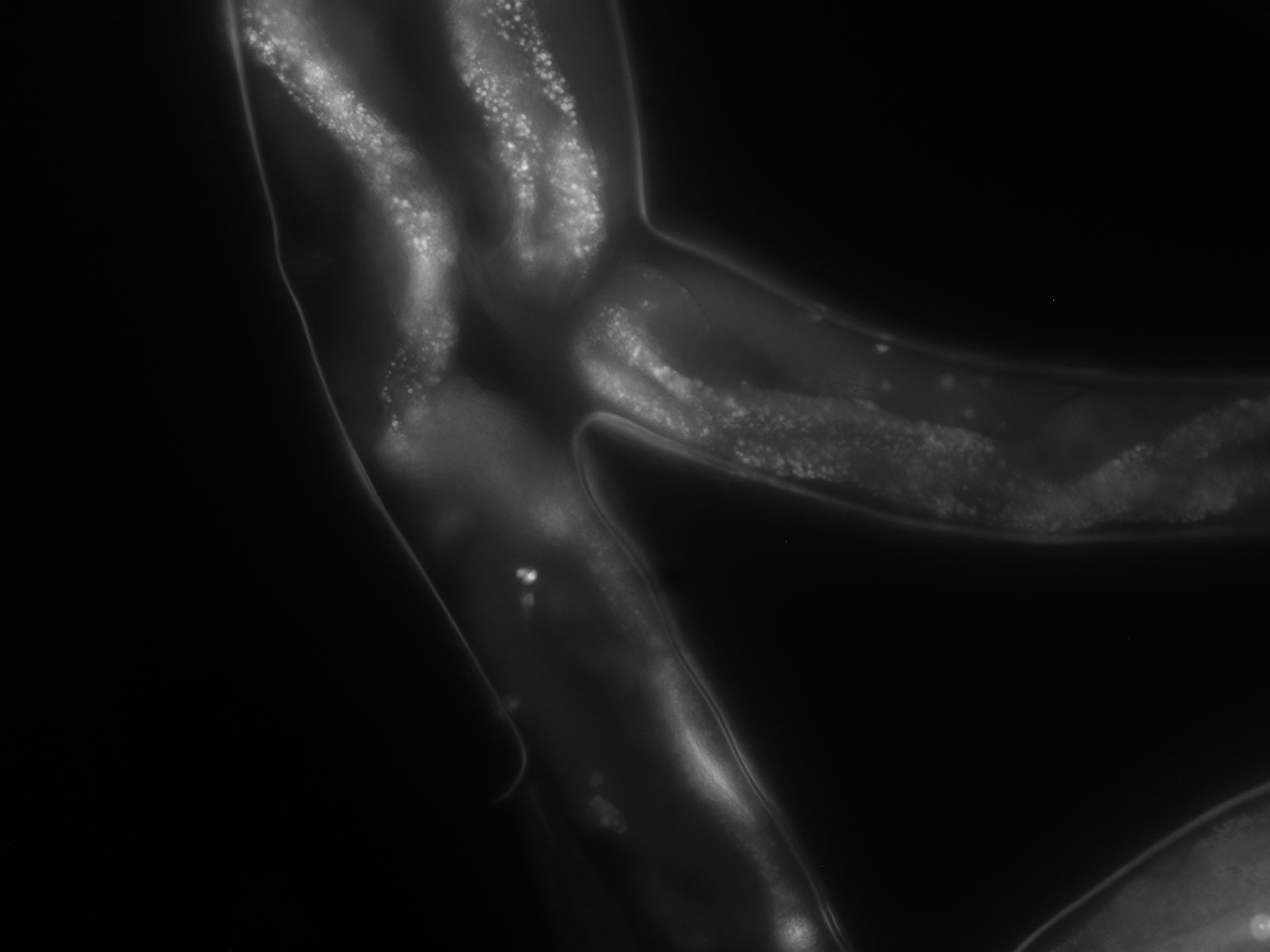

Supplement: S3 File — (ZIP) [file pgen.1011061.s003.zip › Fig.4D - Original files/Fig.4D RAW data and photos JPEG/syto12 staining - Fig 4D - 3_rep - 22.5.23 jpeg/daf-28_tm2308+tfg-133.jpg]

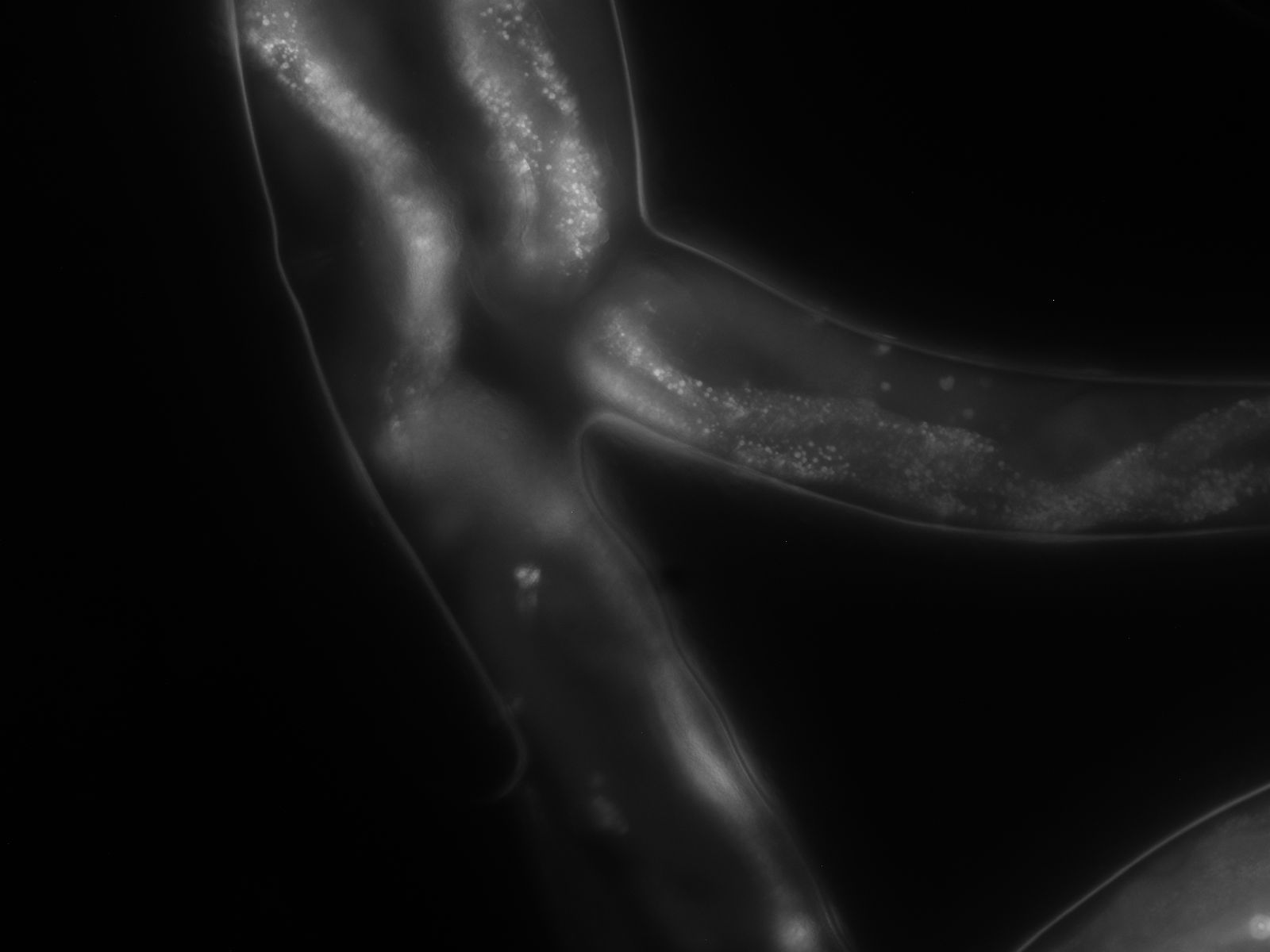

Supplement: S3 File — (ZIP) [file pgen.1011061.s003.zip › Fig.4D - Original files/Fig.4D RAW data and photos JPEG/syto12 staining - Fig 4D - 3_rep - 22.5.23 jpeg/daf-28_tm2308+tfg-134.jpg]

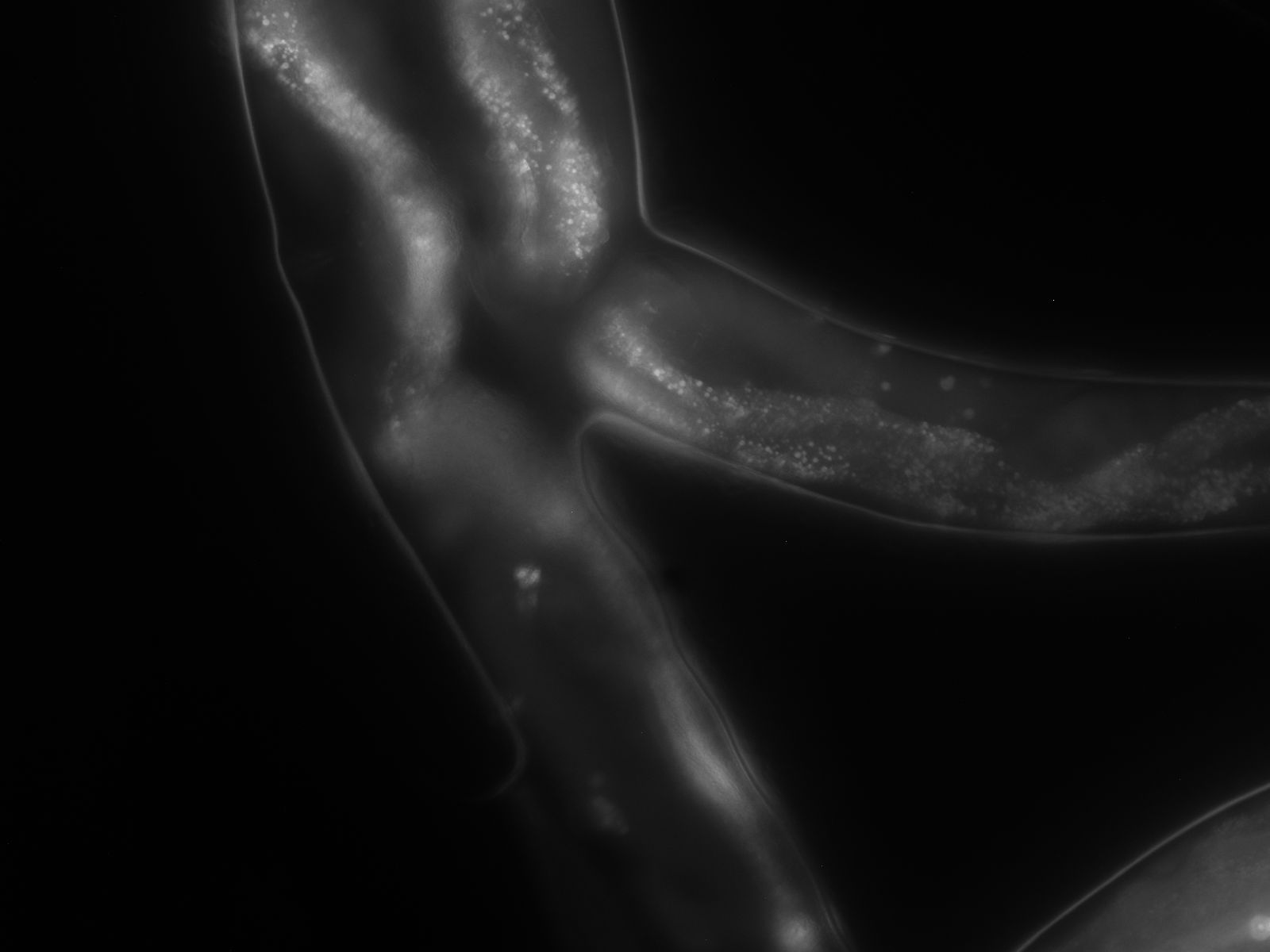

Supplement: S3 File — (ZIP) [file pgen.1011061.s003.zip › Fig.4D - Original files/Fig.4D RAW data and photos JPEG/syto12 staining - Fig 4D - 3_rep - 22.5.23 jpeg/daf-28_tm2308+tfg-135.jpg]

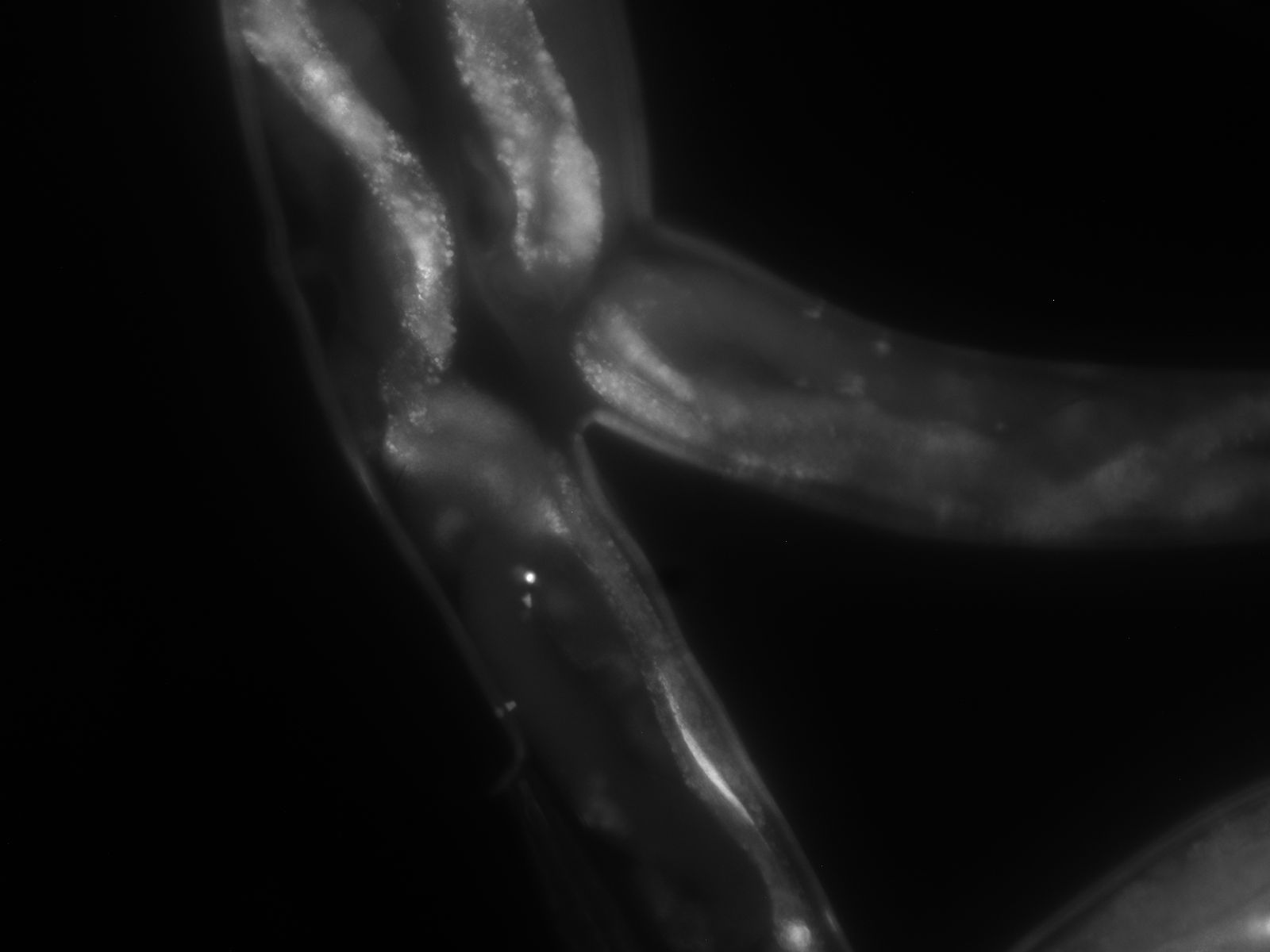

Supplement: S3 File — (ZIP) [file pgen.1011061.s003.zip › Fig.4D - Original files/Fig.4D RAW data and photos JPEG/syto12 staining - Fig 4D - 3_rep - 22.5.23 jpeg/daf-28_tm2308+tfg-136.jpg]

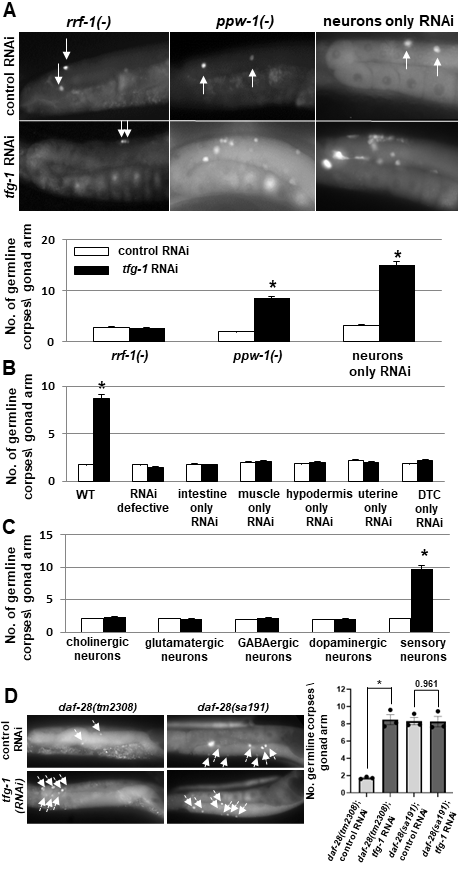

Supplement: S3 File — (ZIP) [file pgen.1011061.s003.zip › Fig.4D - Original files/Updated Figure 4 - 25.6.23.tif]

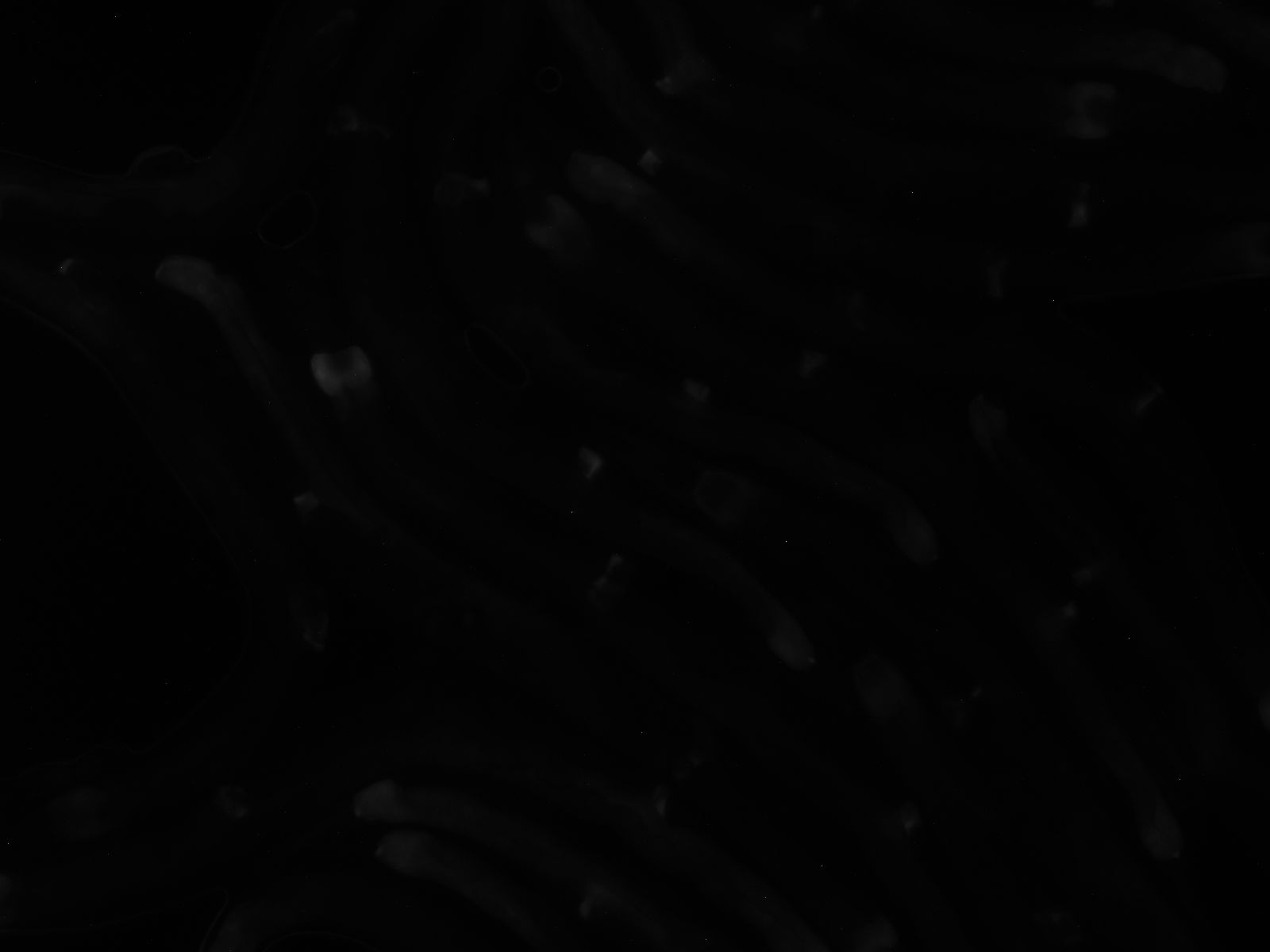

Supplement: S4 File — Since images include multiple worms, images were independently measured several times to achieve measurements for distinct worms in one shared image. Only animals whose body was fully imaged (at least from front to back intestine) were measured. Exposure levels were maintained constant per worm strain. (ZIP) [file pgen.1011061.s004.zip › Fig.S1 - Original files/Fig S1 RAW data and photos - JPEG/reporters on PAD12 or TFG-1 RNAi - 11.5.23 _ 2 rep JPEG/cf2260_hsp-4_gfp+ pad12-day101.jpg]

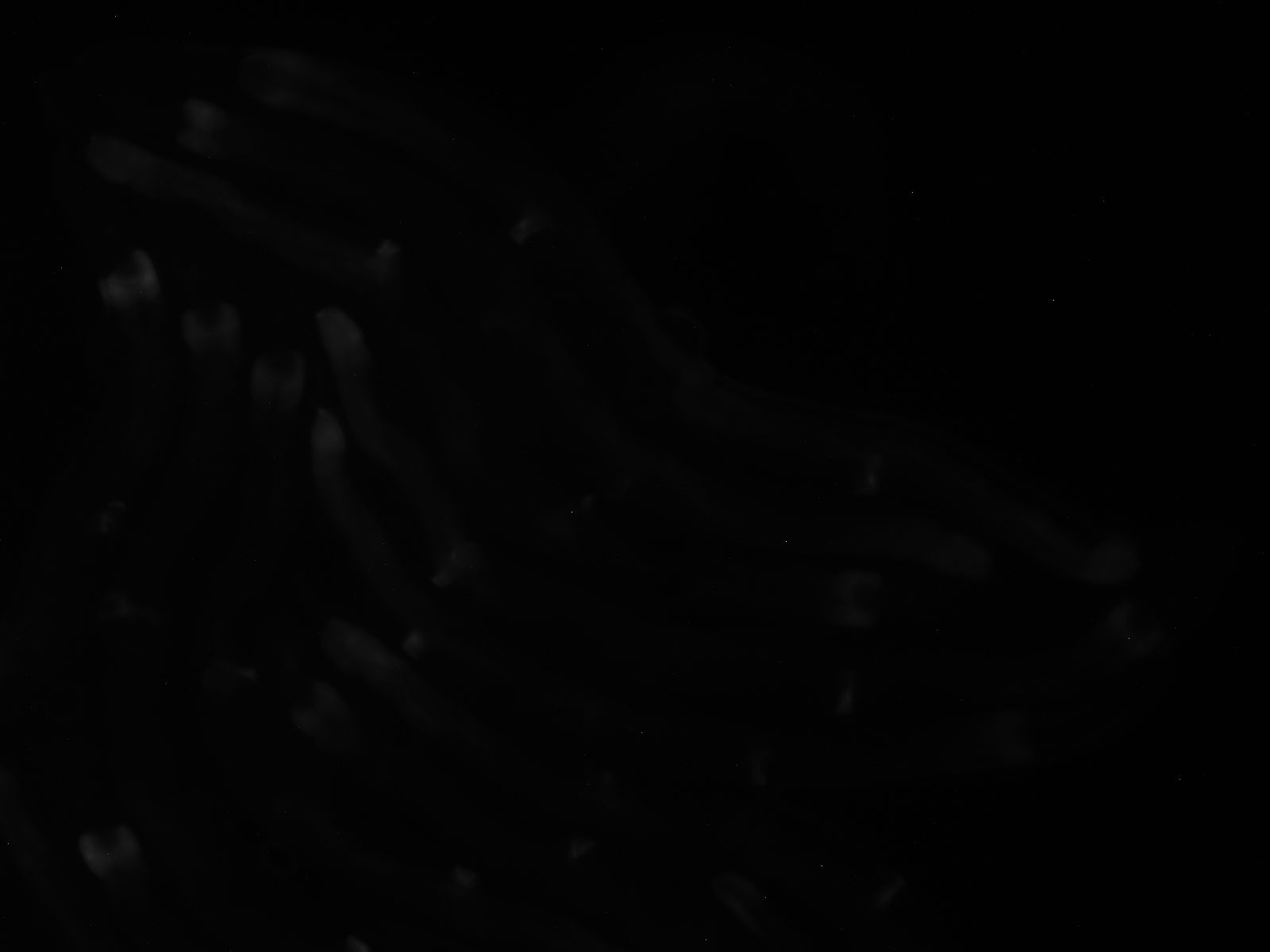

Supplement: S4 File — Since images include multiple worms, images were independently measured several times to achieve measurements for distinct worms in one shared image. Only animals whose body was fully imaged (at least from front to back intestine) were measured. Exposure levels were maintained constant per worm strain. (ZIP) [file pgen.1011061.s004.zip › Fig.S1 - Original files/Fig S1 RAW data and photos - JPEG/reporters on PAD12 or TFG-1 RNAi - 11.5.23 _ 2 rep JPEG/cf2260_hsp-4_gfp+ pad12-day102.jpg]

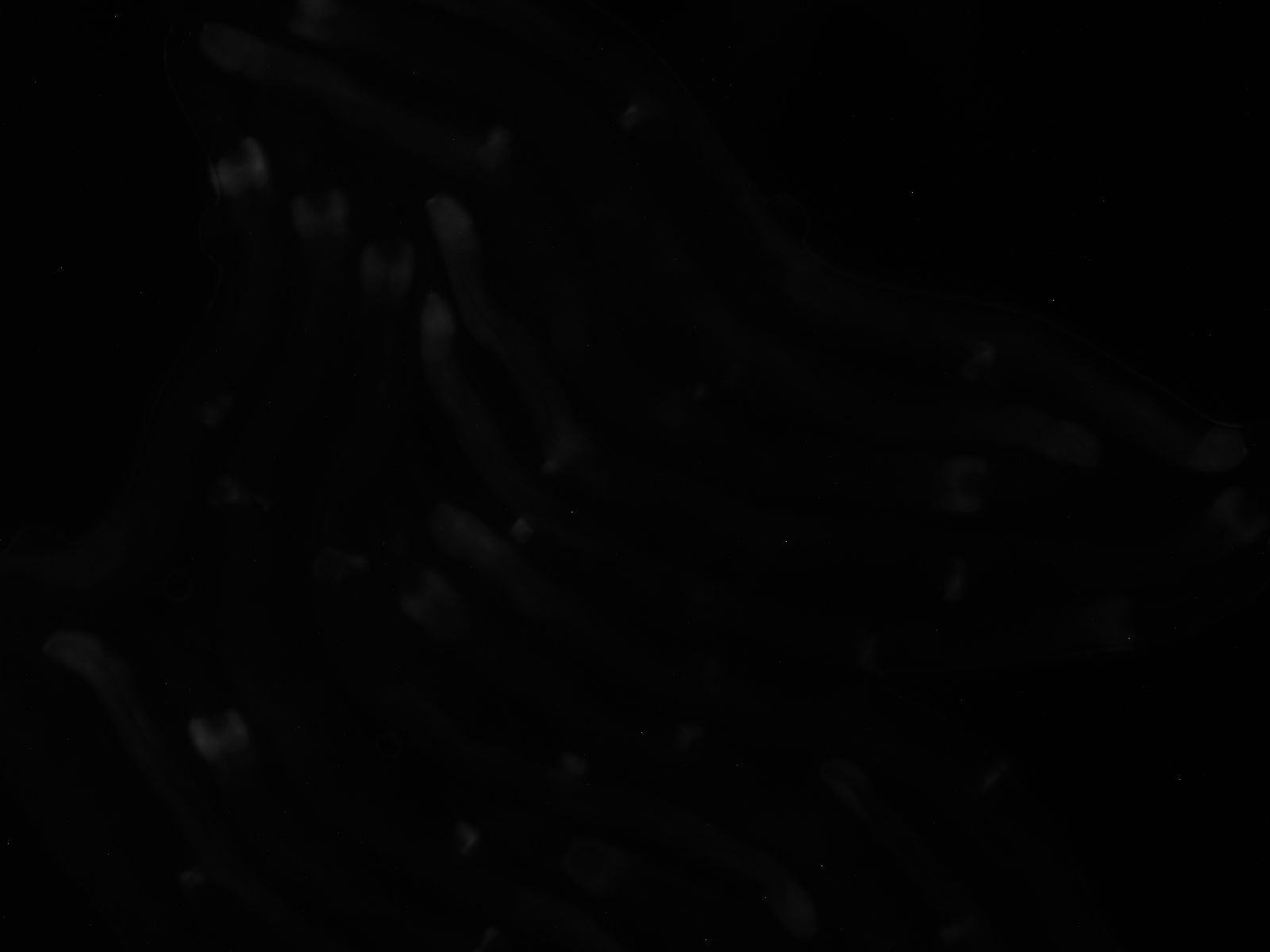

Supplement: S4 File — Since images include multiple worms, images were independently measured several times to achieve measurements for distinct worms in one shared image. Only animals whose body was fully imaged (at least from front to back intestine) were measured. Exposure levels were maintained constant per worm strain. (ZIP) [file pgen.1011061.s004.zip › Fig.S1 - Original files/Fig S1 RAW data and photos - JPEG/reporters on PAD12 or TFG-1 RNAi - 11.5.23 _ 2 rep JPEG/cf2260_hsp-4_gfp+ pad12-day103.jpg]

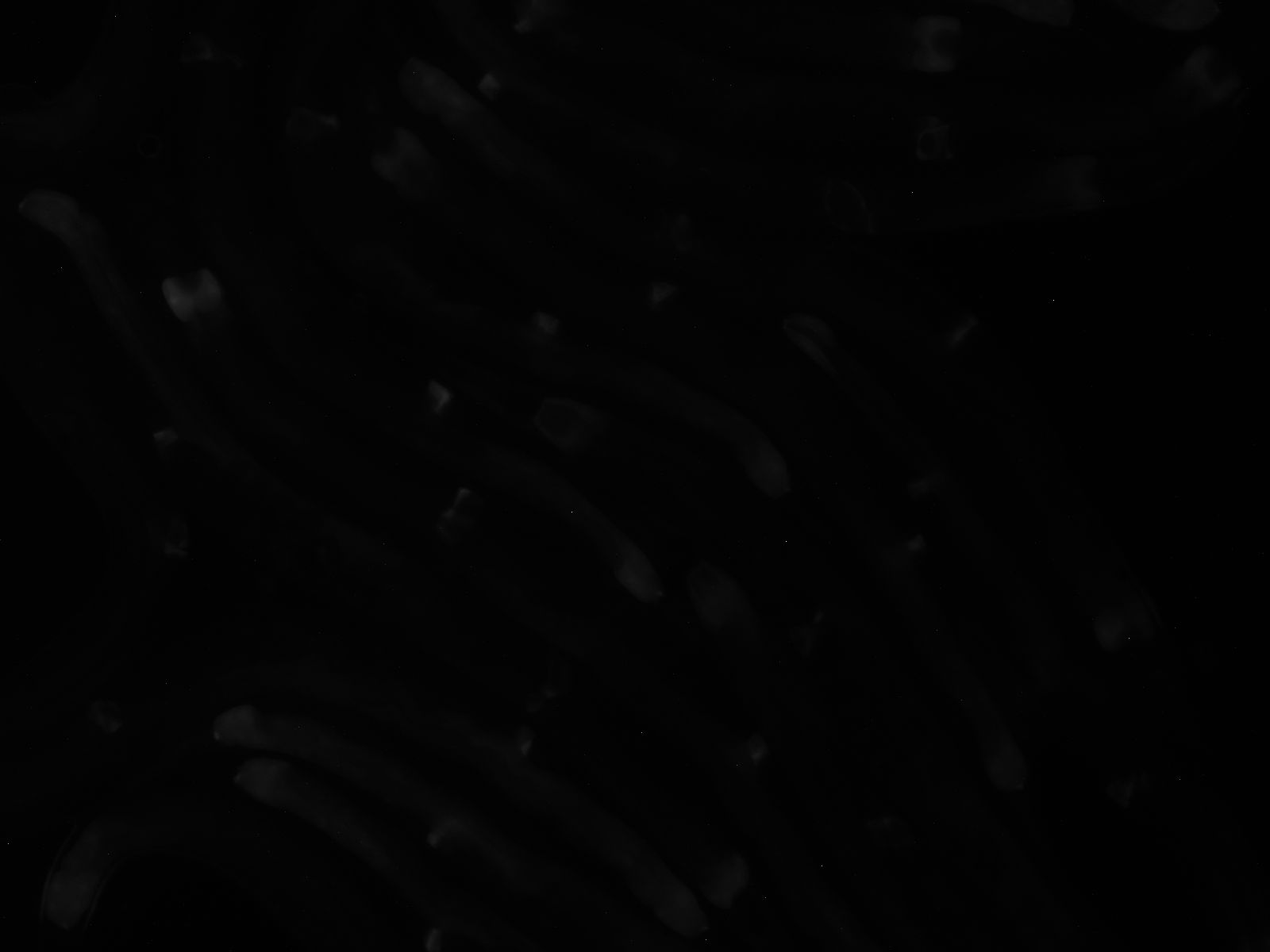

Supplement: S4 File — Since images include multiple worms, images were independently measured several times to achieve measurements for distinct worms in one shared image. Only animals whose body was fully imaged (at least from front to back intestine) were measured. Exposure levels were maintained constant per worm strain. (ZIP) [file pgen.1011061.s004.zip › Fig.S1 - Original files/Fig S1 RAW data and photos - JPEG/reporters on PAD12 or TFG-1 RNAi - 11.5.23 _ 2 rep JPEG/cf2260_hsp-4_gfp+ pad12-day104.jpg]

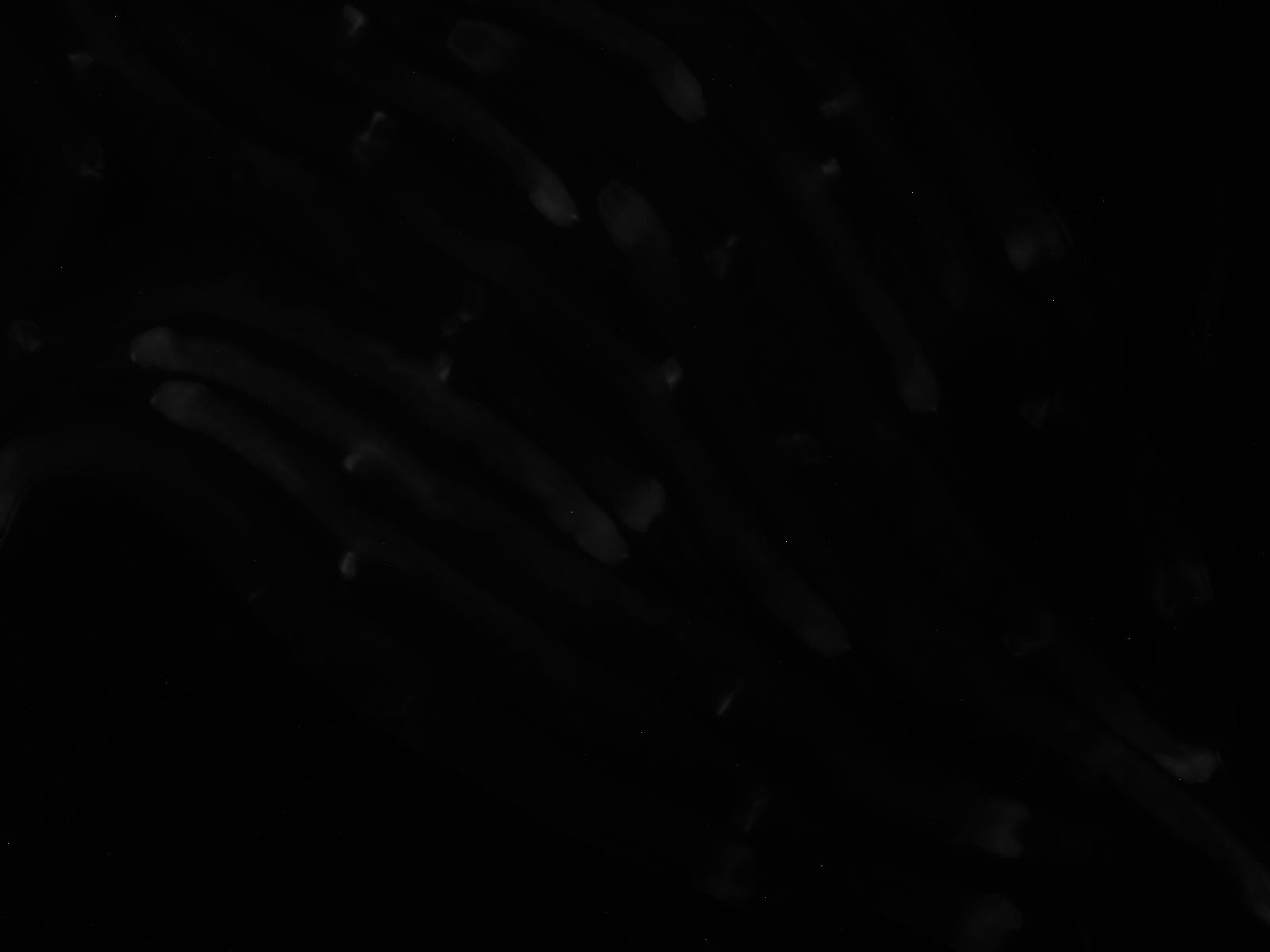

Supplement: S4 File — Since images include multiple worms, images were independently measured several times to achieve measurements for distinct worms in one shared image. Only animals whose body was fully imaged (at least from front to back intestine) were measured. Exposure levels were maintained constant per worm strain. (ZIP) [file pgen.1011061.s004.zip › Fig.S1 - Original files/Fig S1 RAW data and photos - JPEG/reporters on PAD12 or TFG-1 RNAi - 11.5.23 _ 2 rep JPEG/cf2260_hsp-4_gfp+ pad12-day105.jpg]

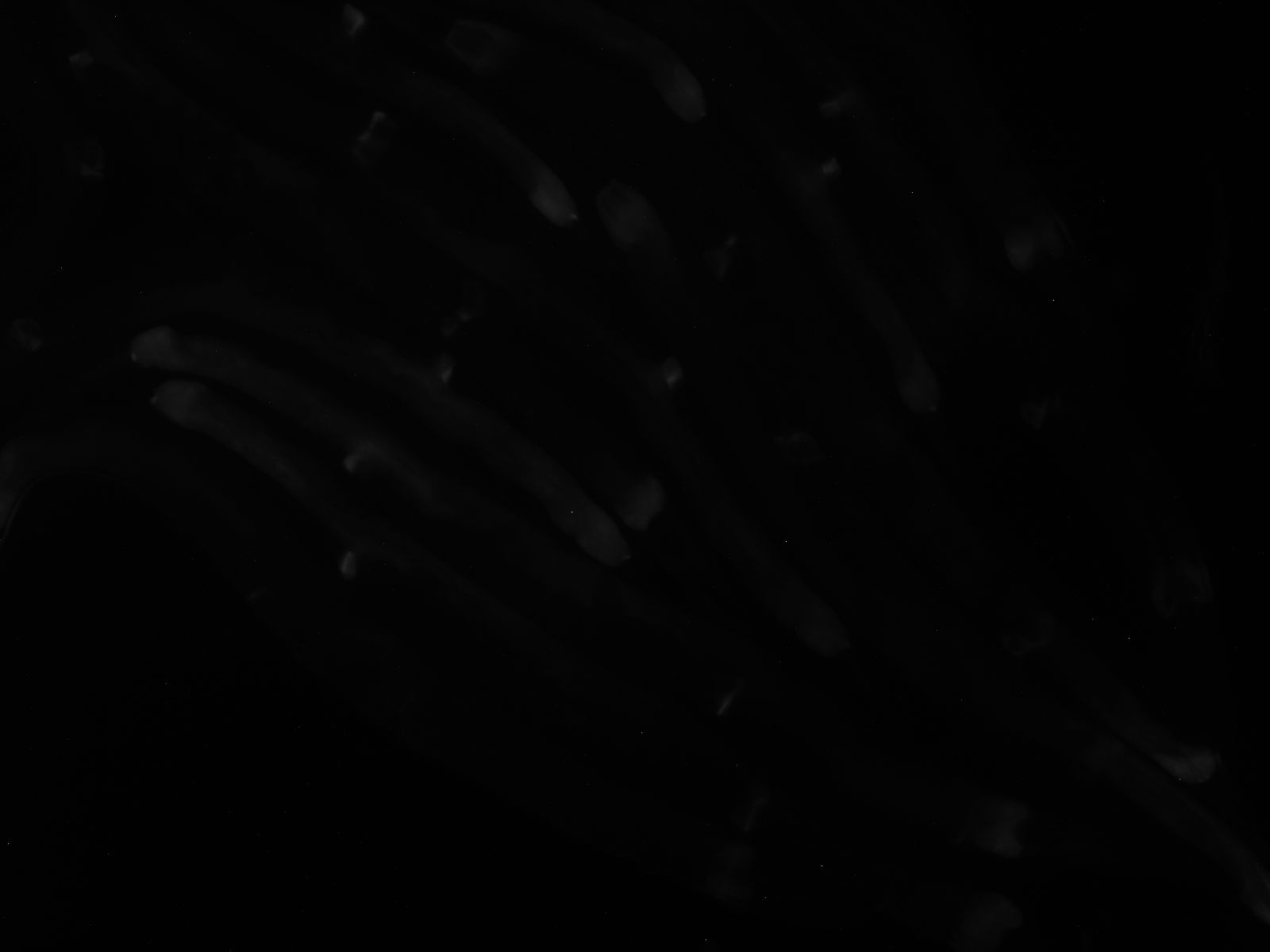

Supplement: S4 File — Since images include multiple worms, images were independently measured several times to achieve measurements for distinct worms in one shared image. Only animals whose body was fully imaged (at least from front to back intestine) were measured. Exposure levels were maintained constant per worm strain. (ZIP) [file pgen.1011061.s004.zip › Fig.S1 - Original files/Fig S1 RAW data and photos - JPEG/reporters on PAD12 or TFG-1 RNAi - 11.5.23 _ 2 rep JPEG/cf2260_hsp-4_gfp+ pad12-day106.jpg]

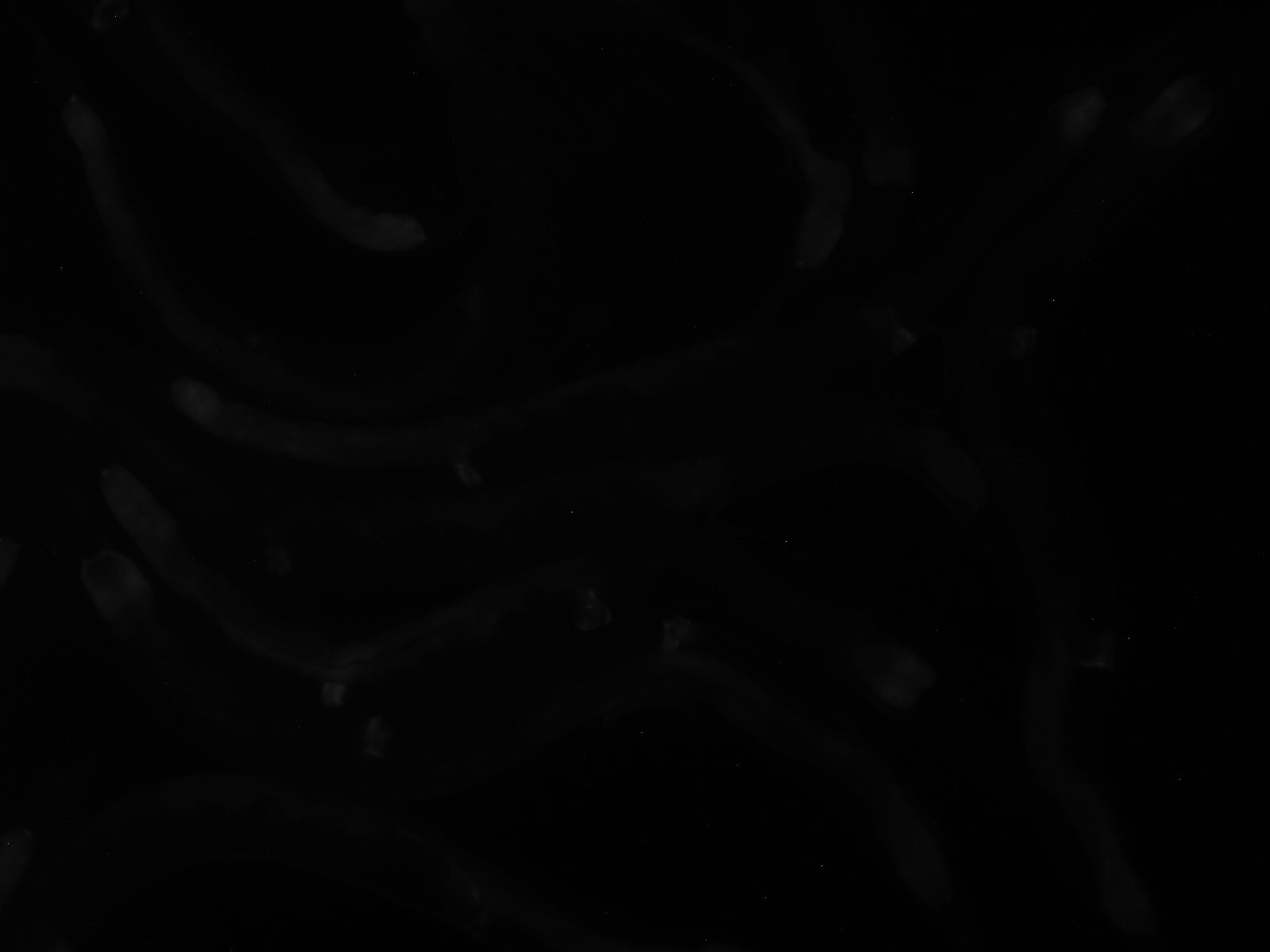

Supplement: S4 File — Since images include multiple worms, images were independently measured several times to achieve measurements for distinct worms in one shared image. Only animals whose body was fully imaged (at least from front to back intestine) were measured. Exposure levels were maintained constant per worm strain. (ZIP) [file pgen.1011061.s004.zip › Fig.S1 - Original files/Fig S1 RAW data and photos - JPEG/reporters on PAD12 or TFG-1 RNAi - 11.5.23 _ 2 rep JPEG/cf2260_hsp-4_gfp+ pad12-day107.jpg]

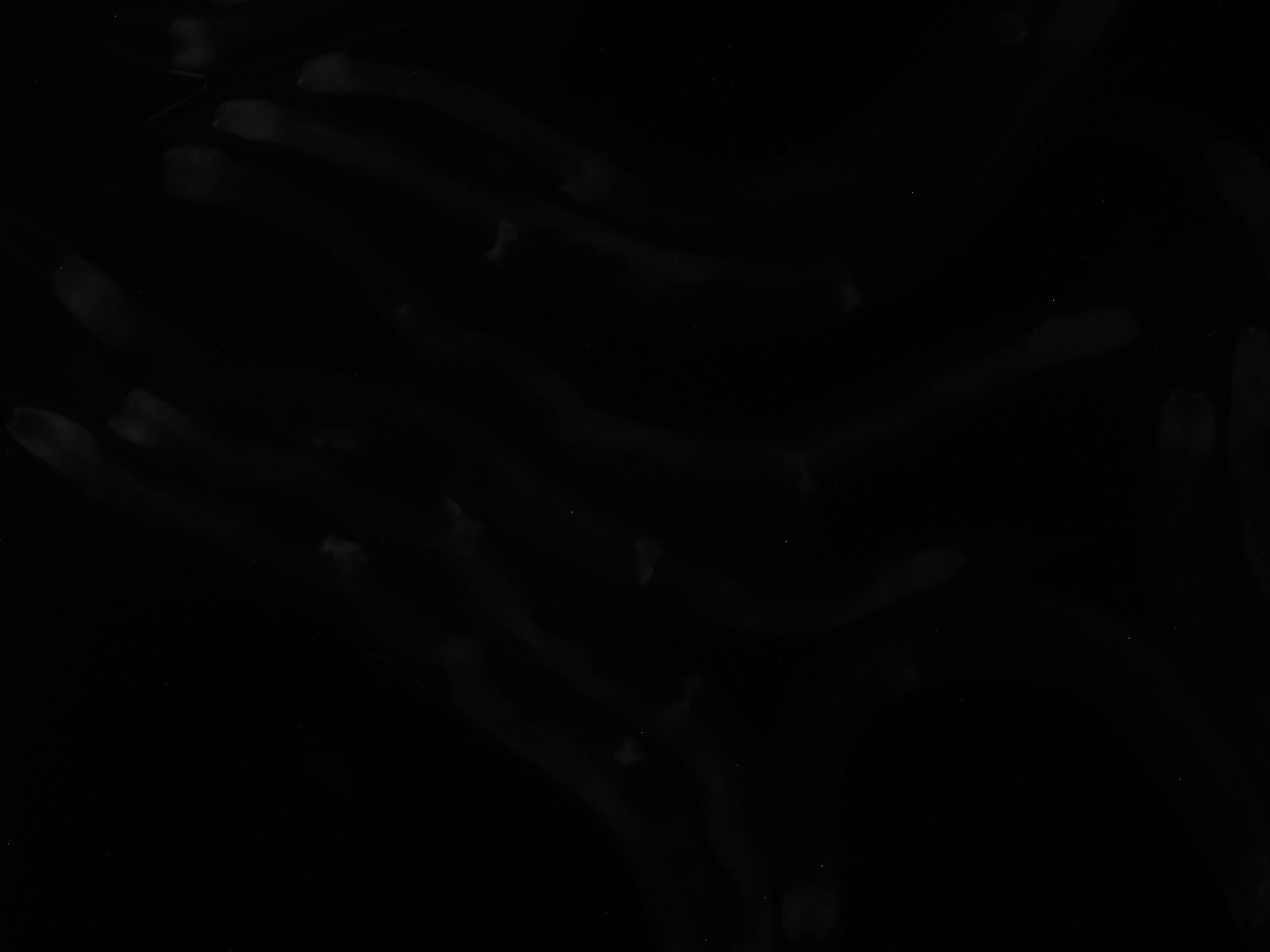

Supplement: S4 File — Since images include multiple worms, images were independently measured several times to achieve measurements for distinct worms in one shared image. Only animals whose body was fully imaged (at least from front to back intestine) were measured. Exposure levels were maintained constant per worm strain. (ZIP) [file pgen.1011061.s004.zip › Fig.S1 - Original files/Fig S1 RAW data and photos - JPEG/reporters on PAD12 or TFG-1 RNAi - 11.5.23 _ 2 rep JPEG/cf2260_hsp-4_gfp+ pad12-day108.jpg]

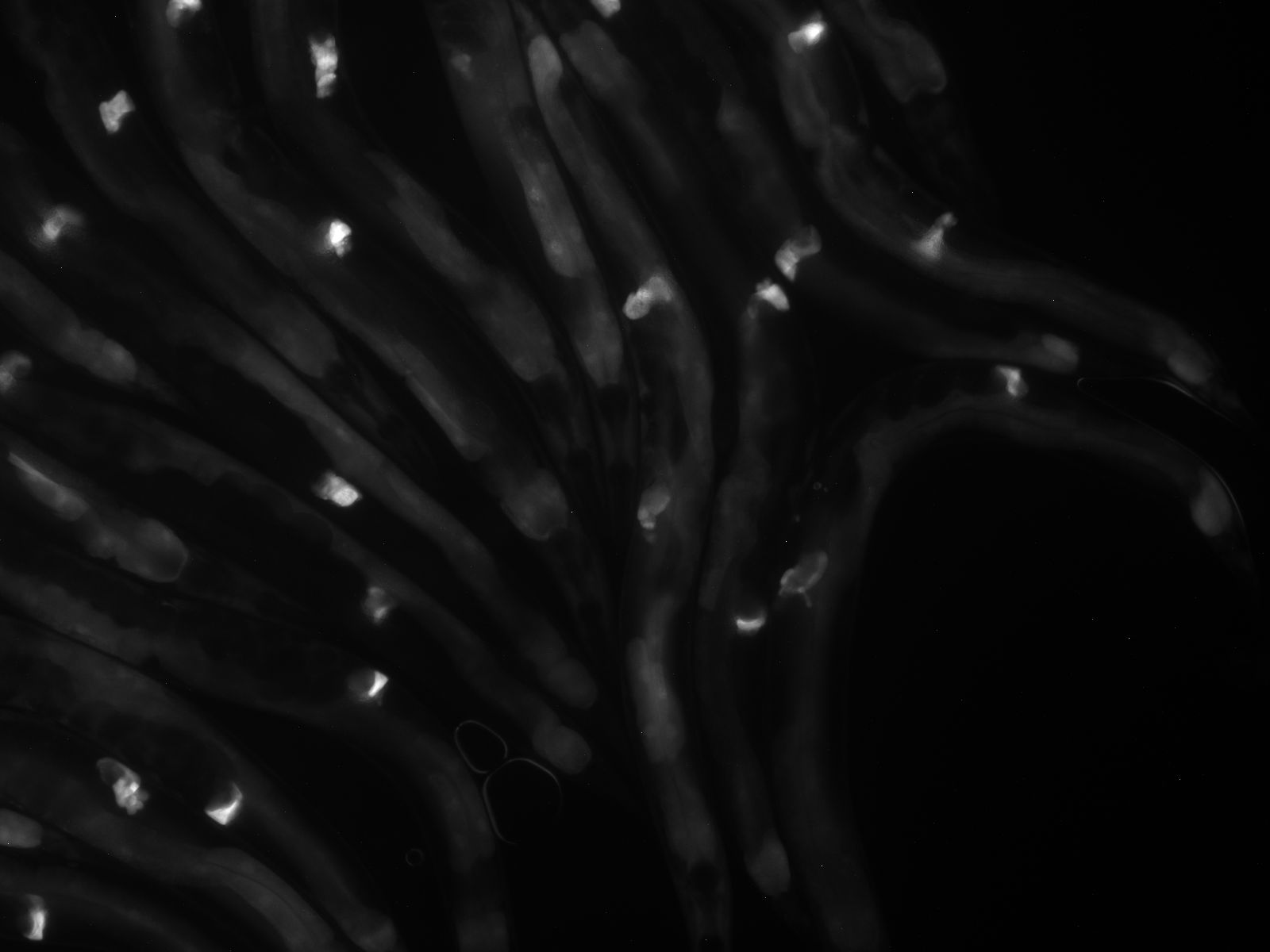

Supplement: S4 File — Since images include multiple worms, images were independently measured several times to achieve measurements for distinct worms in one shared image. Only animals whose body was fully imaged (at least from front to back intestine) were measured. Exposure levels were maintained constant per worm strain. (ZIP) [file pgen.1011061.s004.zip › Fig.S1 - Original files/Fig S1 RAW data and photos - JPEG/reporters on PAD12 or TFG-1 RNAi - 11.5.23 _ 2 rep JPEG/cf2260_hsp-4_gfp+ tfg-1-day109.jpg]

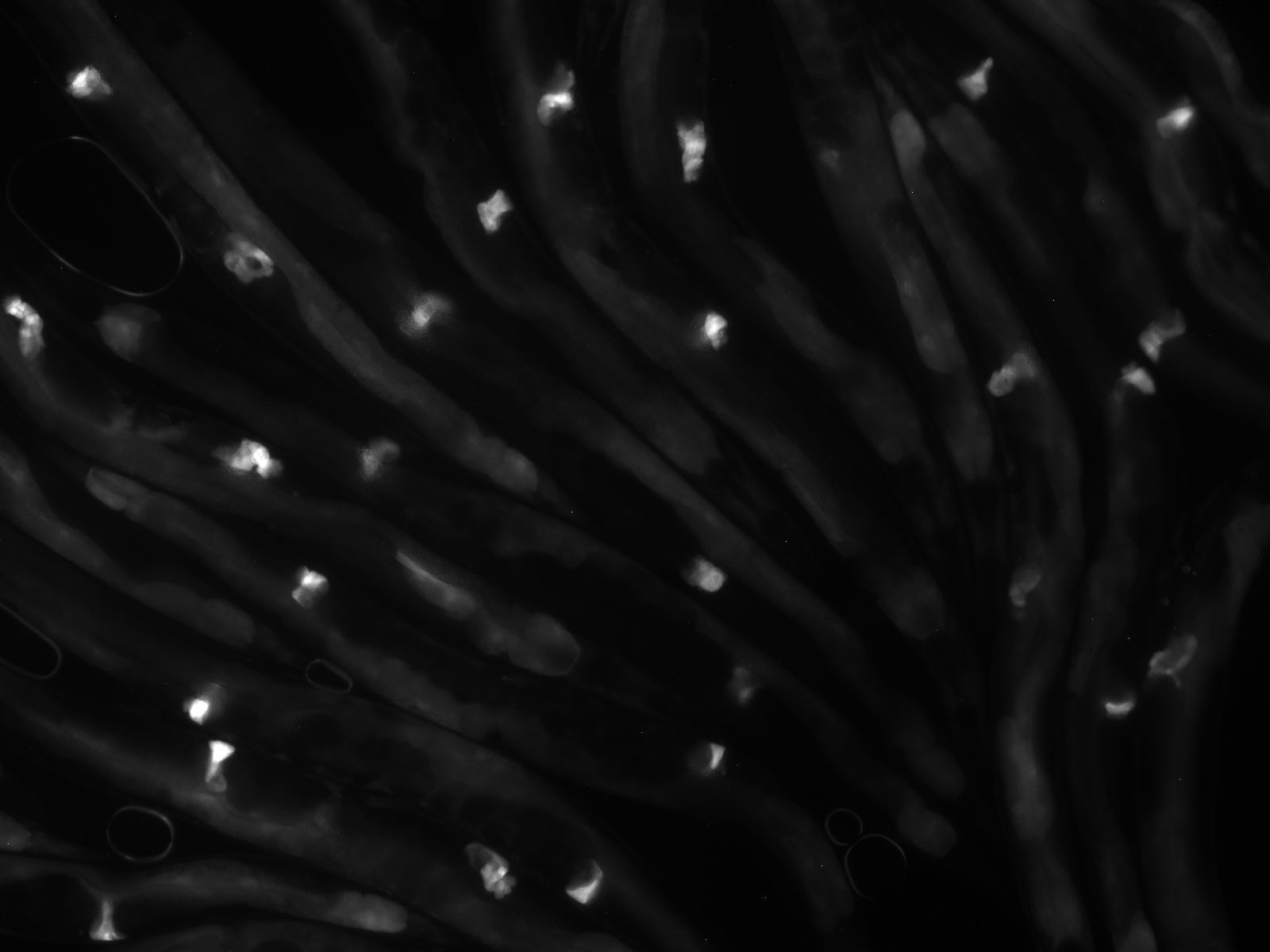

Supplement: S4 File — Since images include multiple worms, images were independently measured several times to achieve measurements for distinct worms in one shared image. Only animals whose body was fully imaged (at least from front to back intestine) were measured. Exposure levels were maintained constant per worm strain. (ZIP) [file pgen.1011061.s004.zip › Fig.S1 - Original files/Fig S1 RAW data and photos - JPEG/reporters on PAD12 or TFG-1 RNAi - 11.5.23 _ 2 rep JPEG/cf2260_hsp-4_gfp+ tfg-1-day110.jpg]

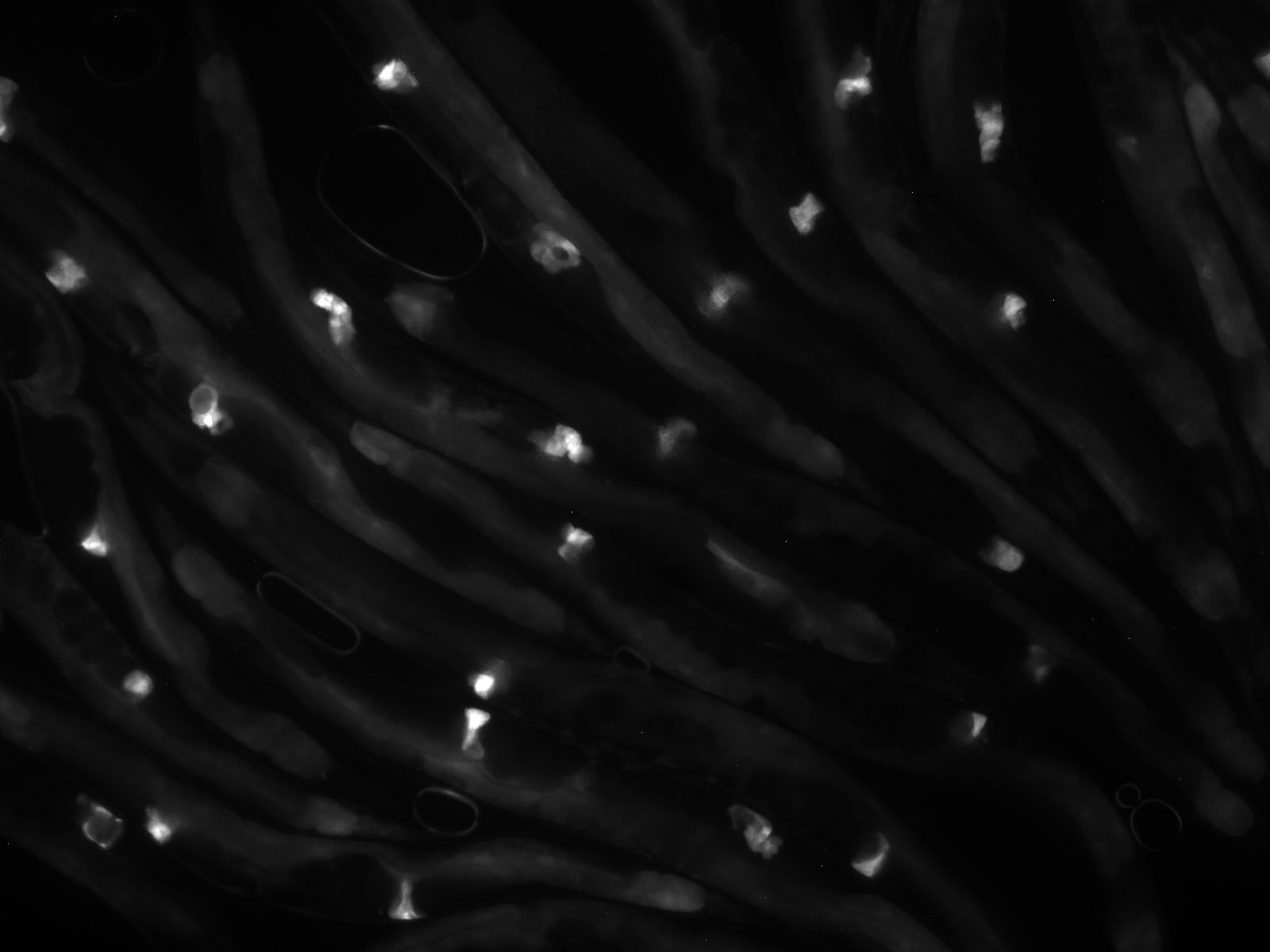

Supplement: S4 File — Since images include multiple worms, images were independently measured several times to achieve measurements for distinct worms in one shared image. Only animals whose body was fully imaged (at least from front to back intestine) were measured. Exposure levels were maintained constant per worm strain. (ZIP) [file pgen.1011061.s004.zip › Fig.S1 - Original files/Fig S1 RAW data and photos - JPEG/reporters on PAD12 or TFG-1 RNAi - 11.5.23 _ 2 rep JPEG/cf2260_hsp-4_gfp+ tfg-1-day111.jpg]

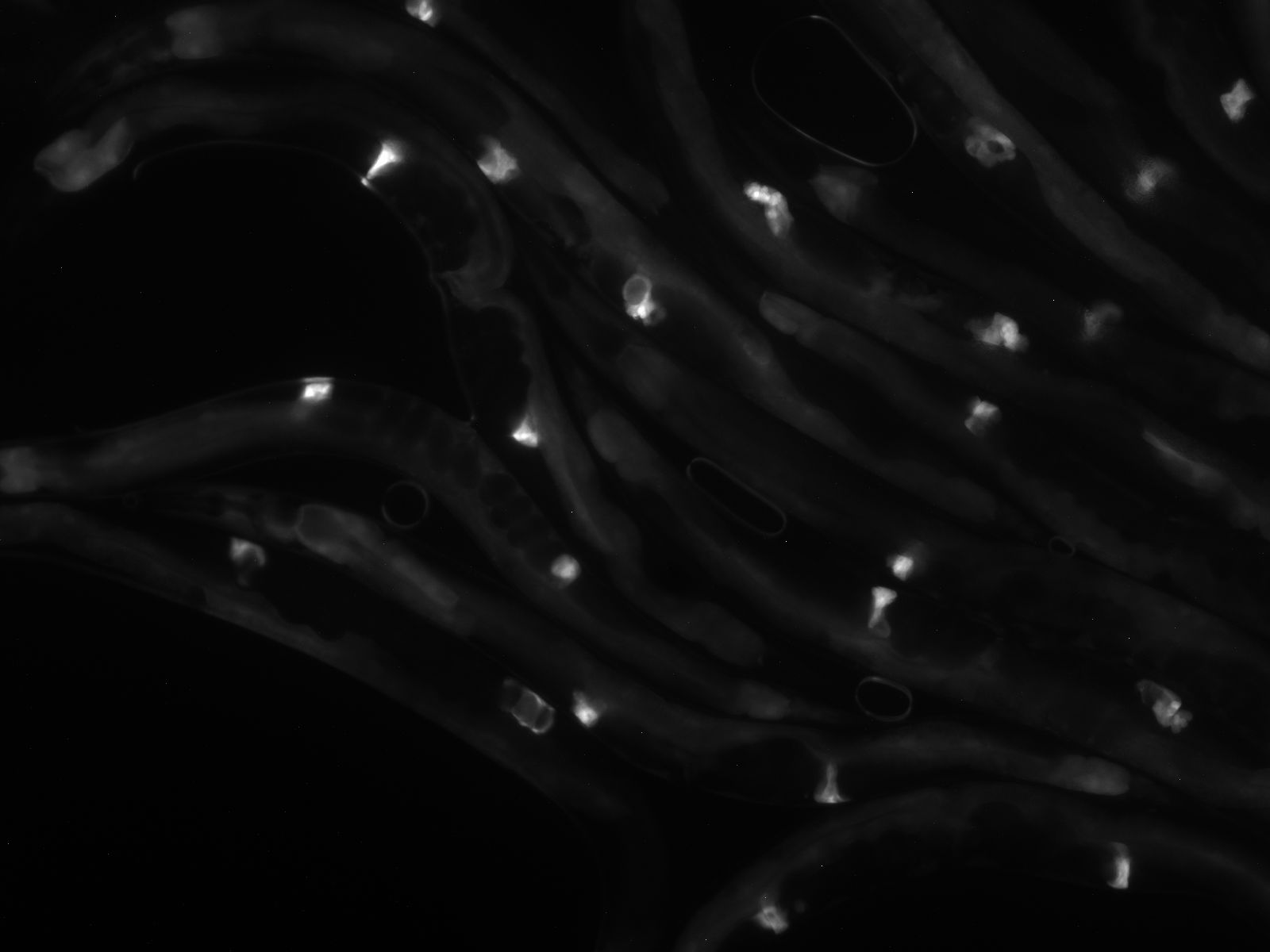

Supplement: S4 File — Since images include multiple worms, images were independently measured several times to achieve measurements for distinct worms in one shared image. Only animals whose body was fully imaged (at least from front to back intestine) were measured. Exposure levels were maintained constant per worm strain. (ZIP) [file pgen.1011061.s004.zip › Fig.S1 - Original files/Fig S1 RAW data and photos - JPEG/reporters on PAD12 or TFG-1 RNAi - 11.5.23 _ 2 rep JPEG/cf2260_hsp-4_gfp+ tfg-1-day112.jpg]

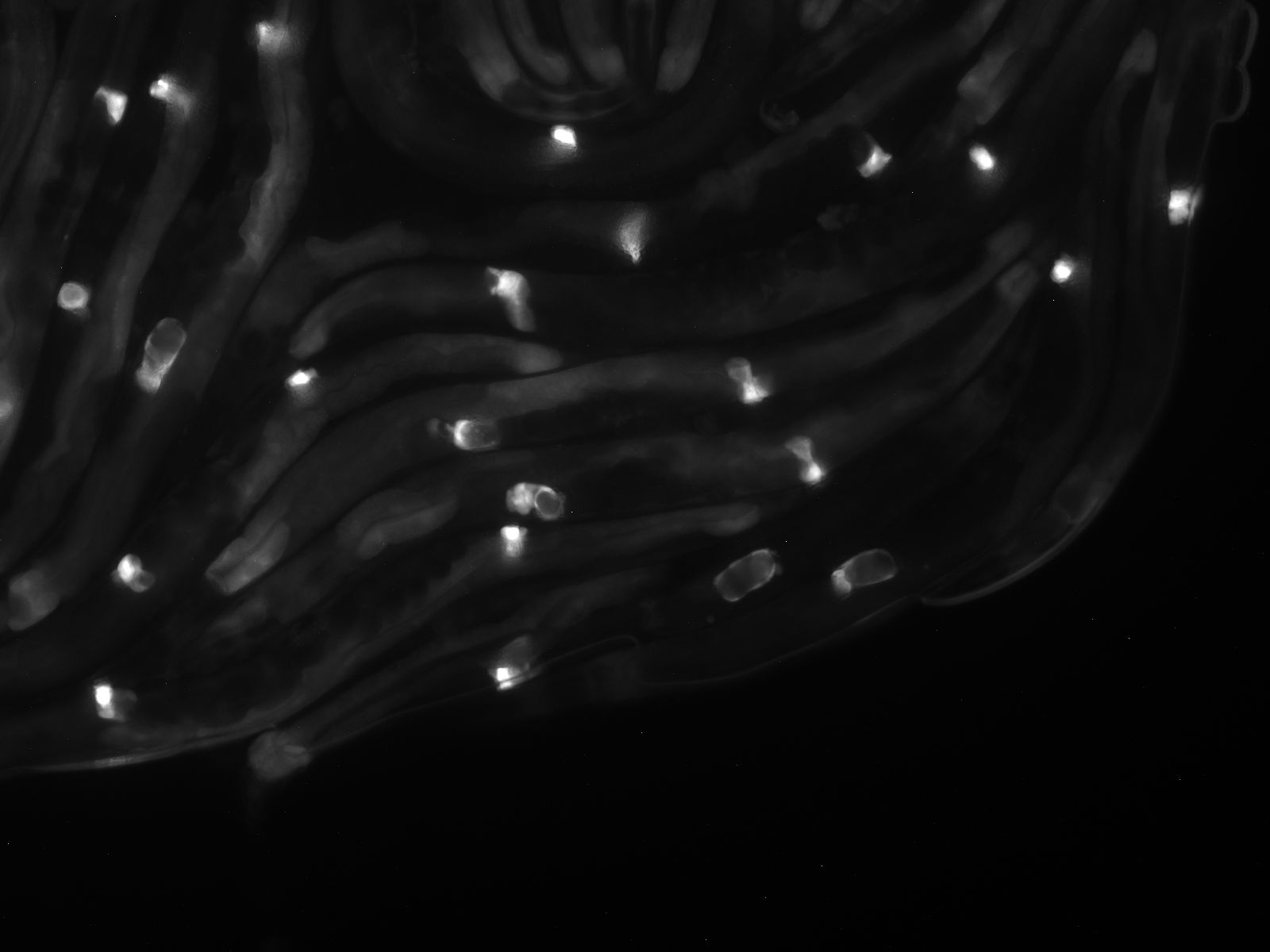

Supplement: S4 File — Since images include multiple worms, images were independently measured several times to achieve measurements for distinct worms in one shared image. Only animals whose body was fully imaged (at least from front to back intestine) were measured. Exposure levels were maintained constant per worm strain. (ZIP) [file pgen.1011061.s004.zip › Fig.S1 - Original files/Fig S1 RAW data and photos - JPEG/reporters on PAD12 or TFG-1 RNAi - 11.5.23 _ 2 rep JPEG/cf2260_hsp-4_gfp+ tfg-1-day113.jpg]

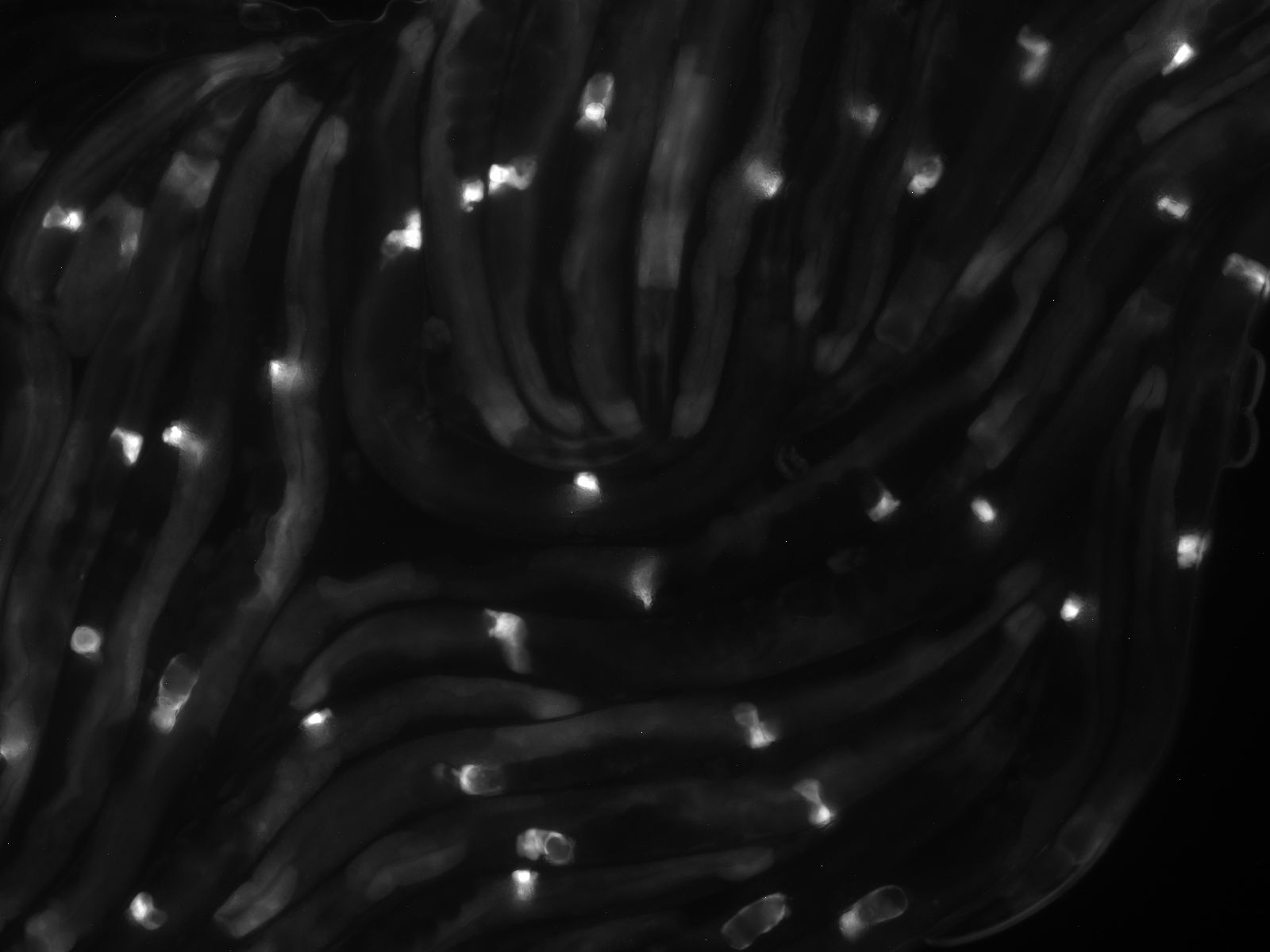

Supplement: S4 File — Since images include multiple worms, images were independently measured several times to achieve measurements for distinct worms in one shared image. Only animals whose body was fully imaged (at least from front to back intestine) were measured. Exposure levels were maintained constant per worm strain. (ZIP) [file pgen.1011061.s004.zip › Fig.S1 - Original files/Fig S1 RAW data and photos - JPEG/reporters on PAD12 or TFG-1 RNAi - 11.5.23 _ 2 rep JPEG/cf2260_hsp-4_gfp+ tfg-1-day114.jpg]

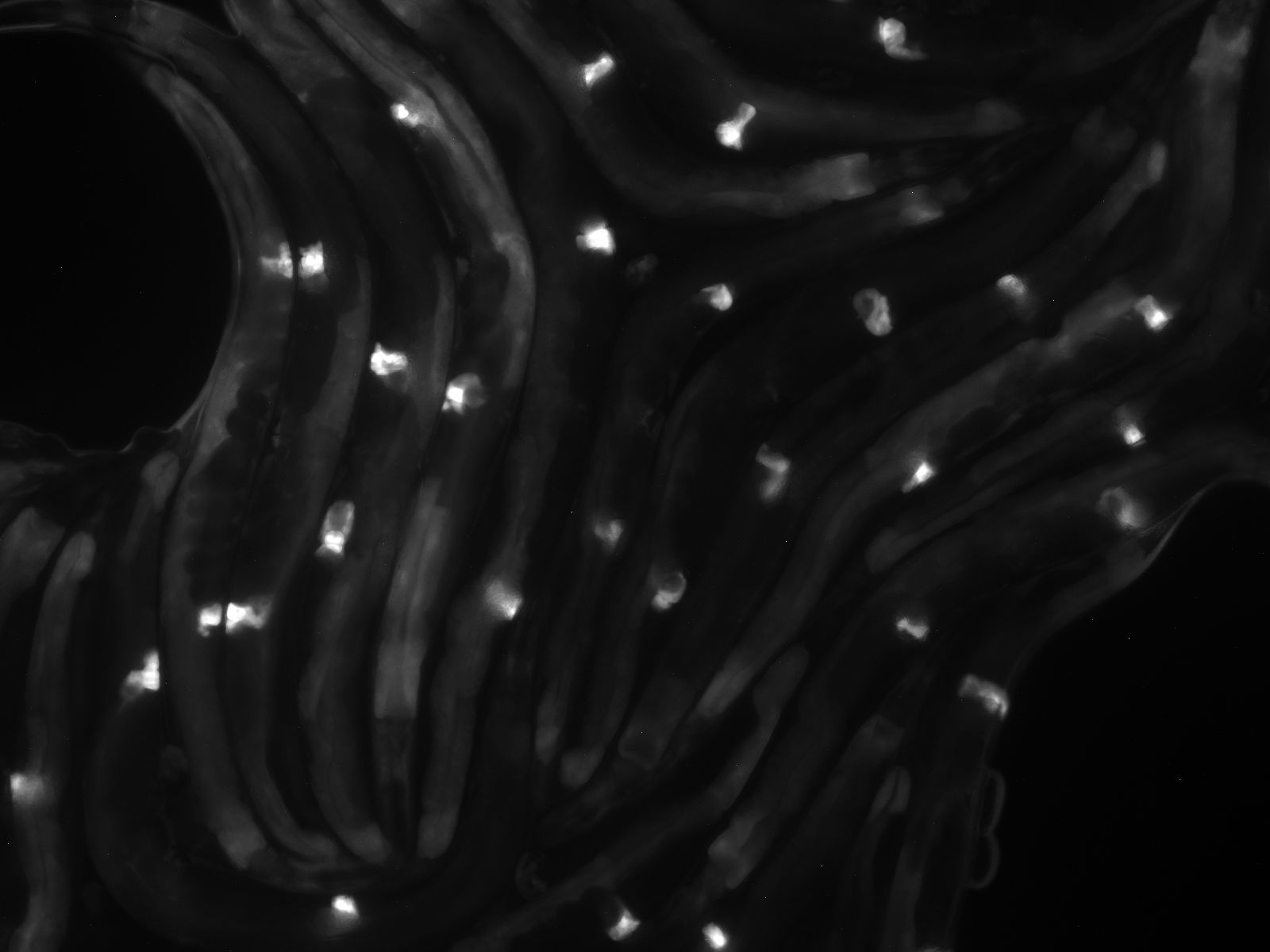

Supplement: S4 File — Since images include multiple worms, images were independently measured several times to achieve measurements for distinct worms in one shared image. Only animals whose body was fully imaged (at least from front to back intestine) were measured. Exposure levels were maintained constant per worm strain. (ZIP) [file pgen.1011061.s004.zip › Fig.S1 - Original files/Fig S1 RAW data and photos - JPEG/reporters on PAD12 or TFG-1 RNAi - 11.5.23 _ 2 rep JPEG/cf2260_hsp-4_gfp+ tfg-1-day115.jpg]

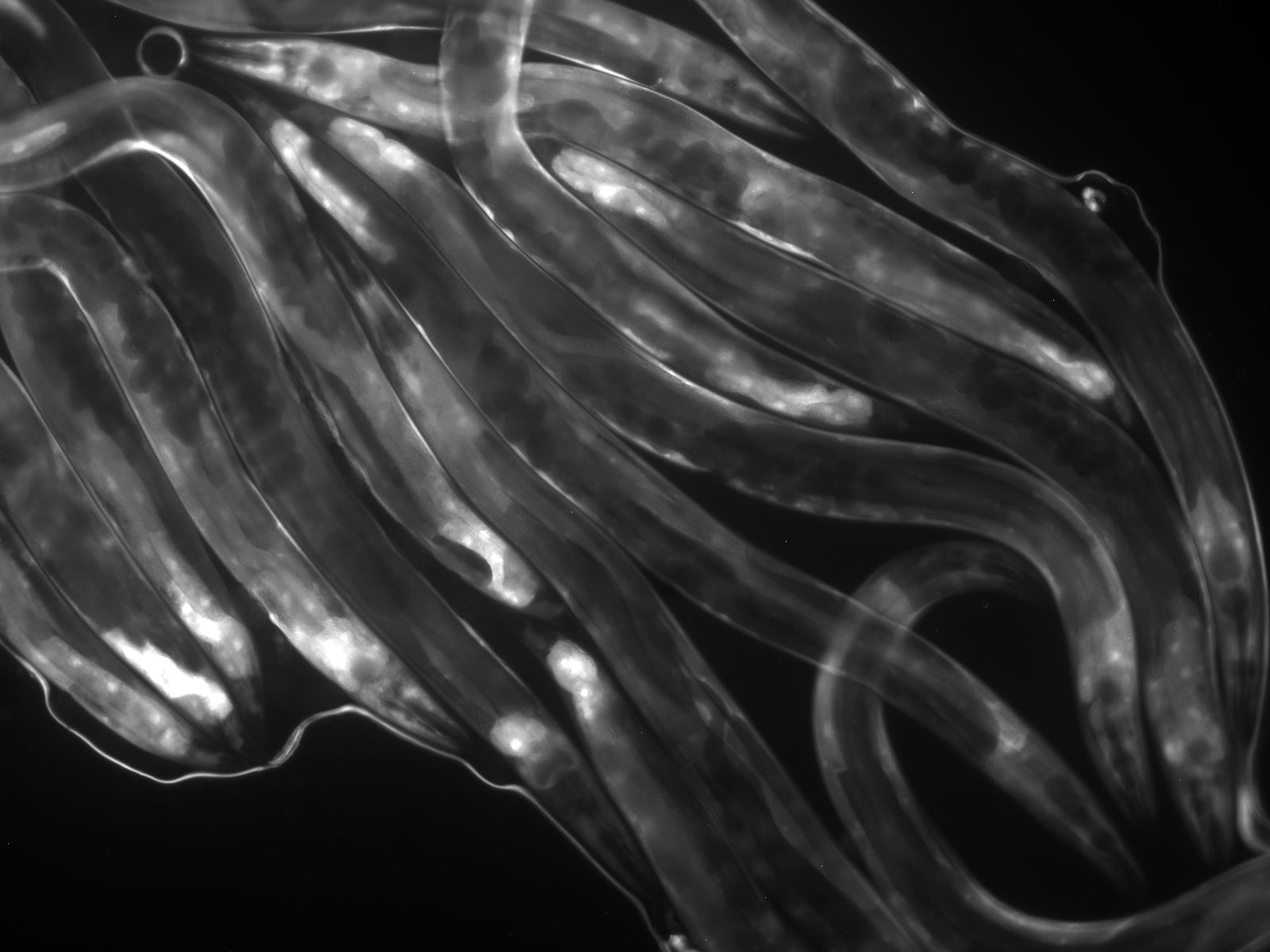

Supplement: S4 File — Since images include multiple worms, images were independently measured several times to achieve measurements for distinct worms in one shared image. Only animals whose body was fully imaged (at least from front to back intestine) were measured. Exposure levels were maintained constant per worm strain. (ZIP) [file pgen.1011061.s004.zip › Fig.S1 - Original files/Fig S1 RAW data and photos - JPEG/reporters on PAD12 or TFG-1 RNAi - 11.5.23 _ 2 rep JPEG/cl2166_gst-4_gfp+ pad12 -day116.jpg]

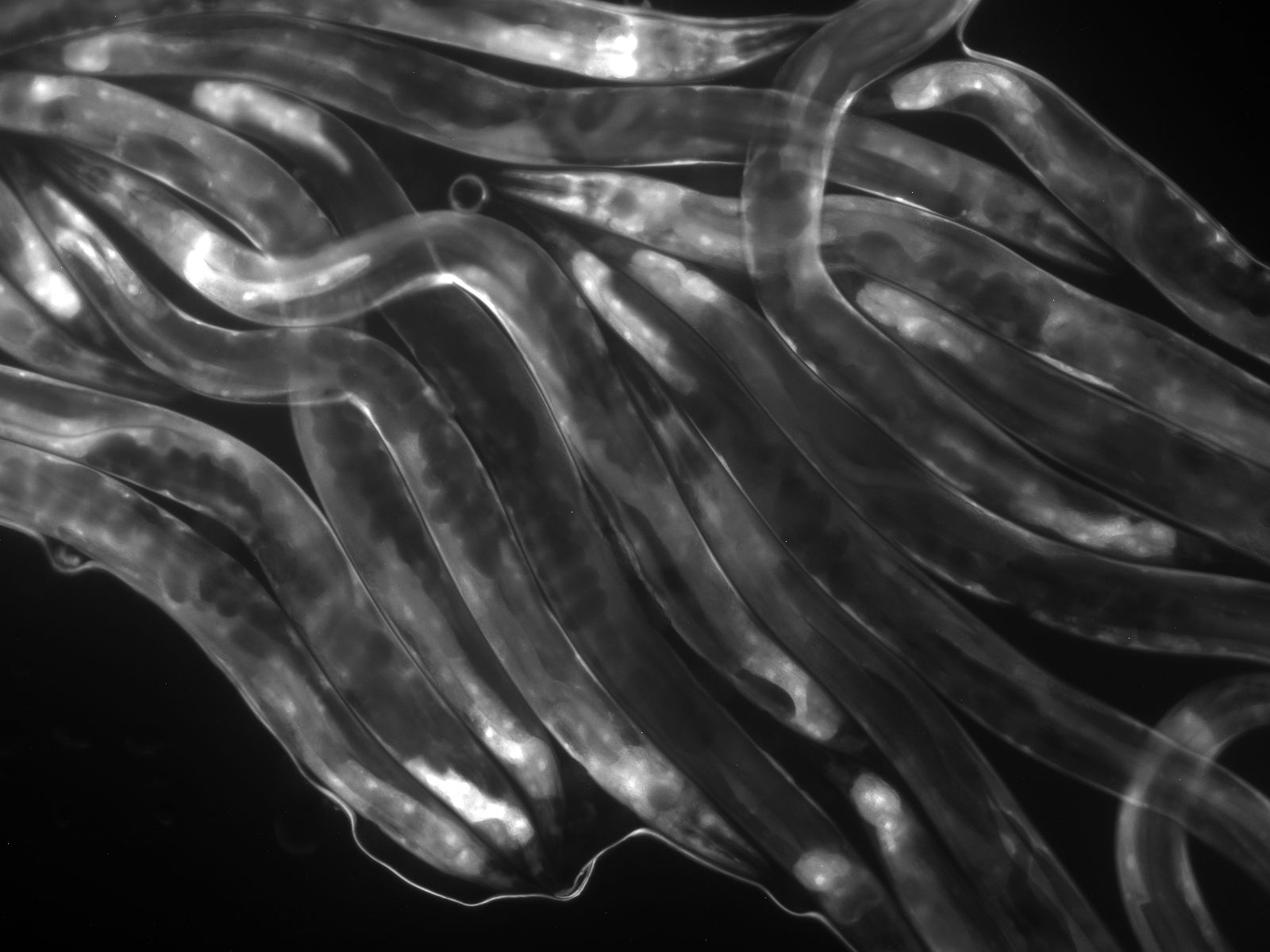

Supplement: S4 File — Since images include multiple worms, images were independently measured several times to achieve measurements for distinct worms in one shared image. Only animals whose body was fully imaged (at least from front to back intestine) were measured. Exposure levels were maintained constant per worm strain. (ZIP) [file pgen.1011061.s004.zip › Fig.S1 - Original files/Fig S1 RAW data and photos - JPEG/reporters on PAD12 or TFG-1 RNAi - 11.5.23 _ 2 rep JPEG/cl2166_gst-4_gfp+ pad12 -day117.jpg]

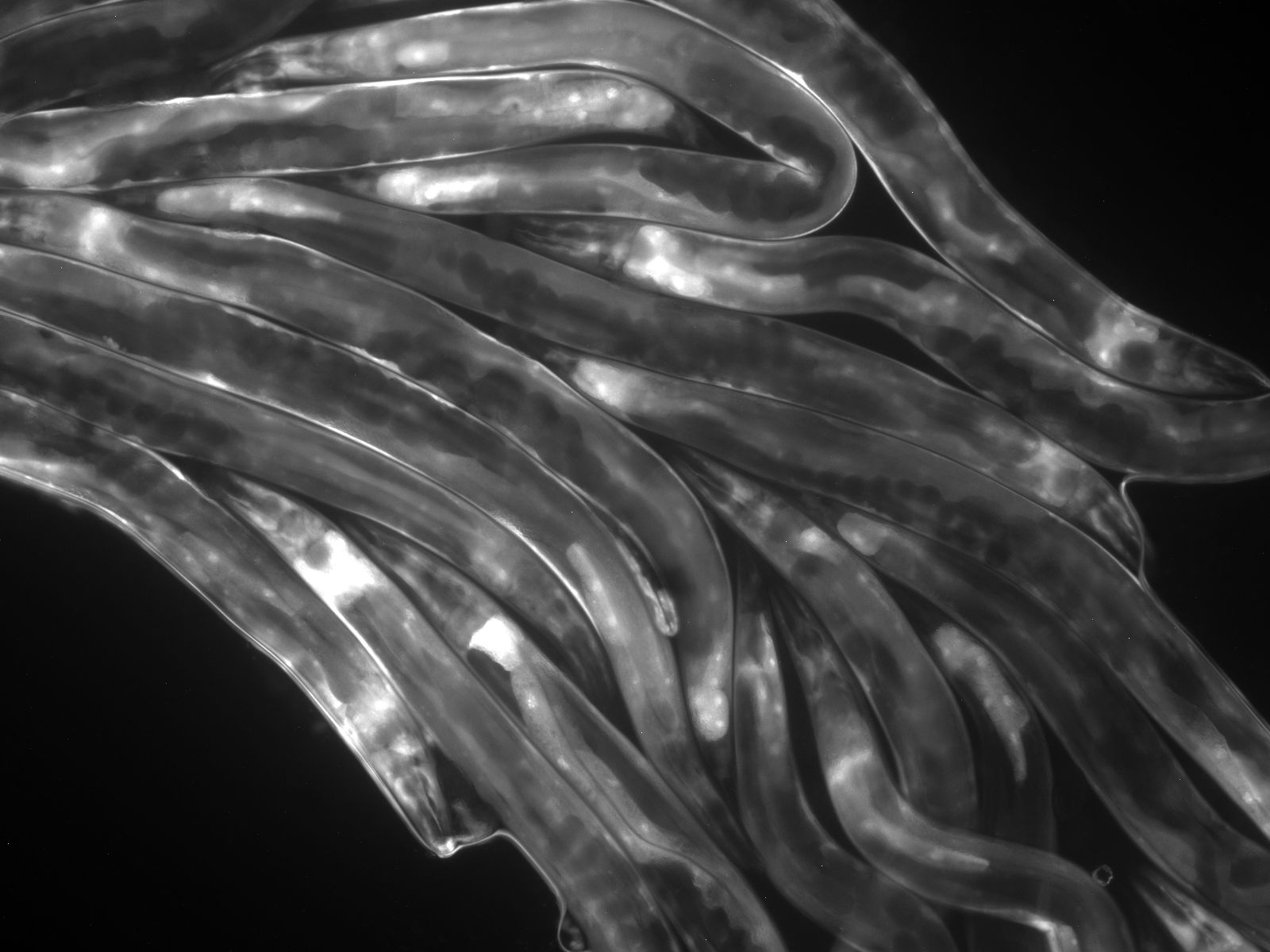

Supplement: S4 File — Since images include multiple worms, images were independently measured several times to achieve measurements for distinct worms in one shared image. Only animals whose body was fully imaged (at least from front to back intestine) were measured. Exposure levels were maintained constant per worm strain. (ZIP) [file pgen.1011061.s004.zip › Fig.S1 - Original files/Fig S1 RAW data and photos - JPEG/reporters on PAD12 or TFG-1 RNAi - 11.5.23 _ 2 rep JPEG/cl2166_gst-4_gfp+ pad12 -day118.jpg]

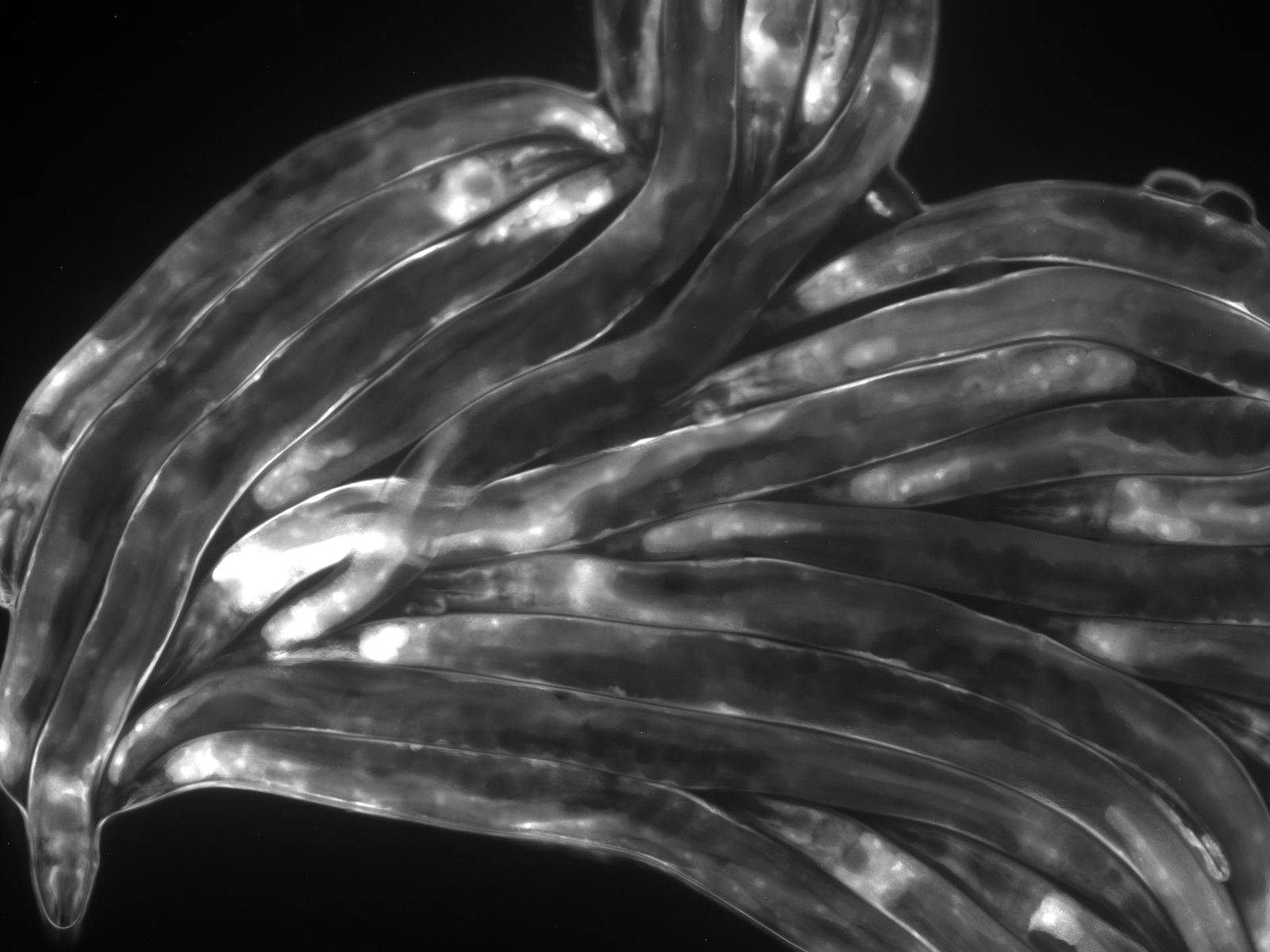

Supplement: S4 File — Since images include multiple worms, images were independently measured several times to achieve measurements for distinct worms in one shared image. Only animals whose body was fully imaged (at least from front to back intestine) were measured. Exposure levels were maintained constant per worm strain. (ZIP) [file pgen.1011061.s004.zip › Fig.S1 - Original files/Fig S1 RAW data and photos - JPEG/reporters on PAD12 or TFG-1 RNAi - 11.5.23 _ 2 rep JPEG/cl2166_gst-4_gfp+ pad12 -day119.jpg]

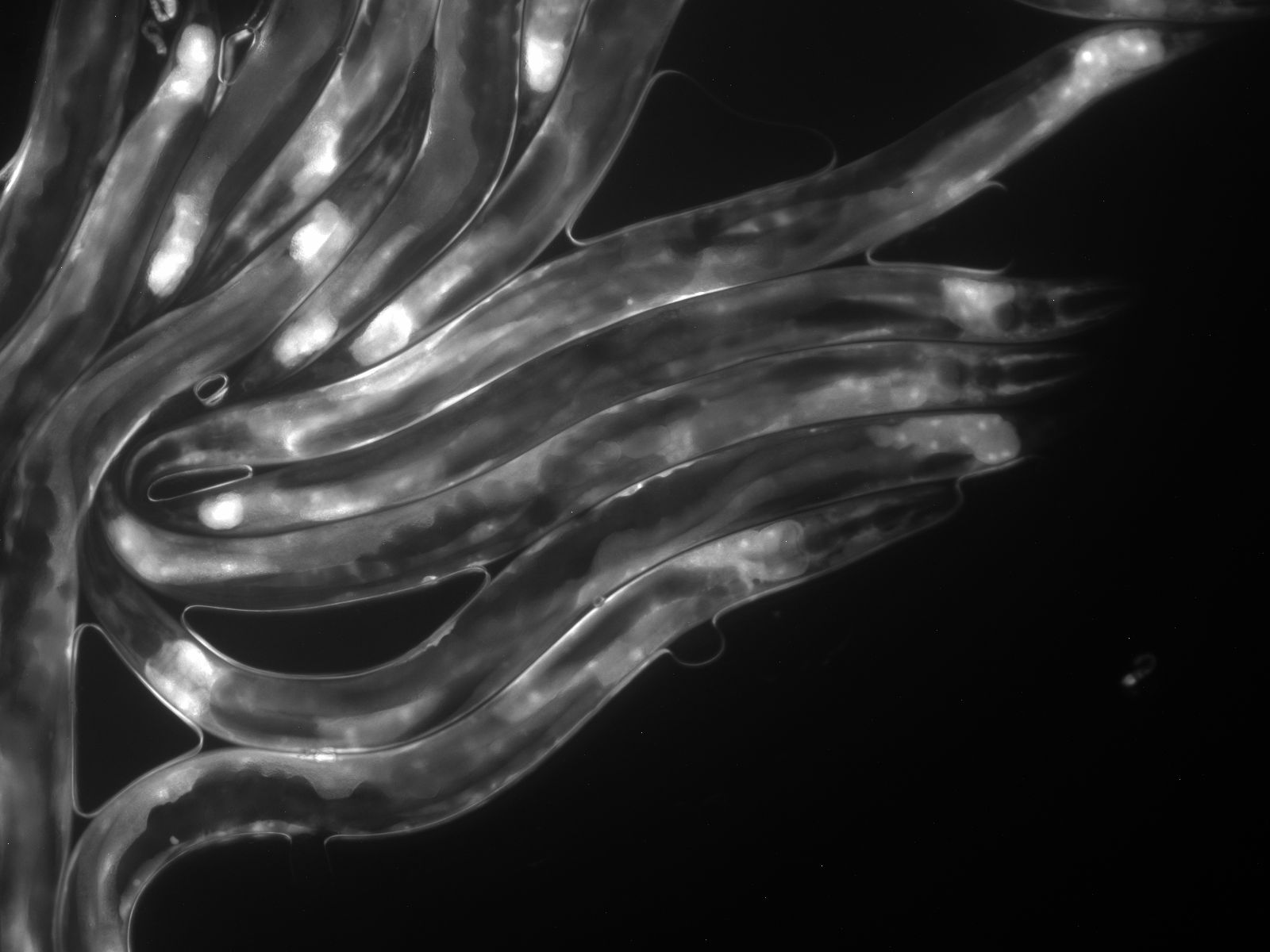

Supplement: S4 File — Since images include multiple worms, images were independently measured several times to achieve measurements for distinct worms in one shared image. Only animals whose body was fully imaged (at least from front to back intestine) were measured. Exposure levels were maintained constant per worm strain. (ZIP) [file pgen.1011061.s004.zip › Fig.S1 - Original files/Fig S1 RAW data and photos - JPEG/reporters on PAD12 or TFG-1 RNAi - 11.5.23 _ 2 rep JPEG/cl2166_gst-4_gfp+ pad12 -day120.jpg]

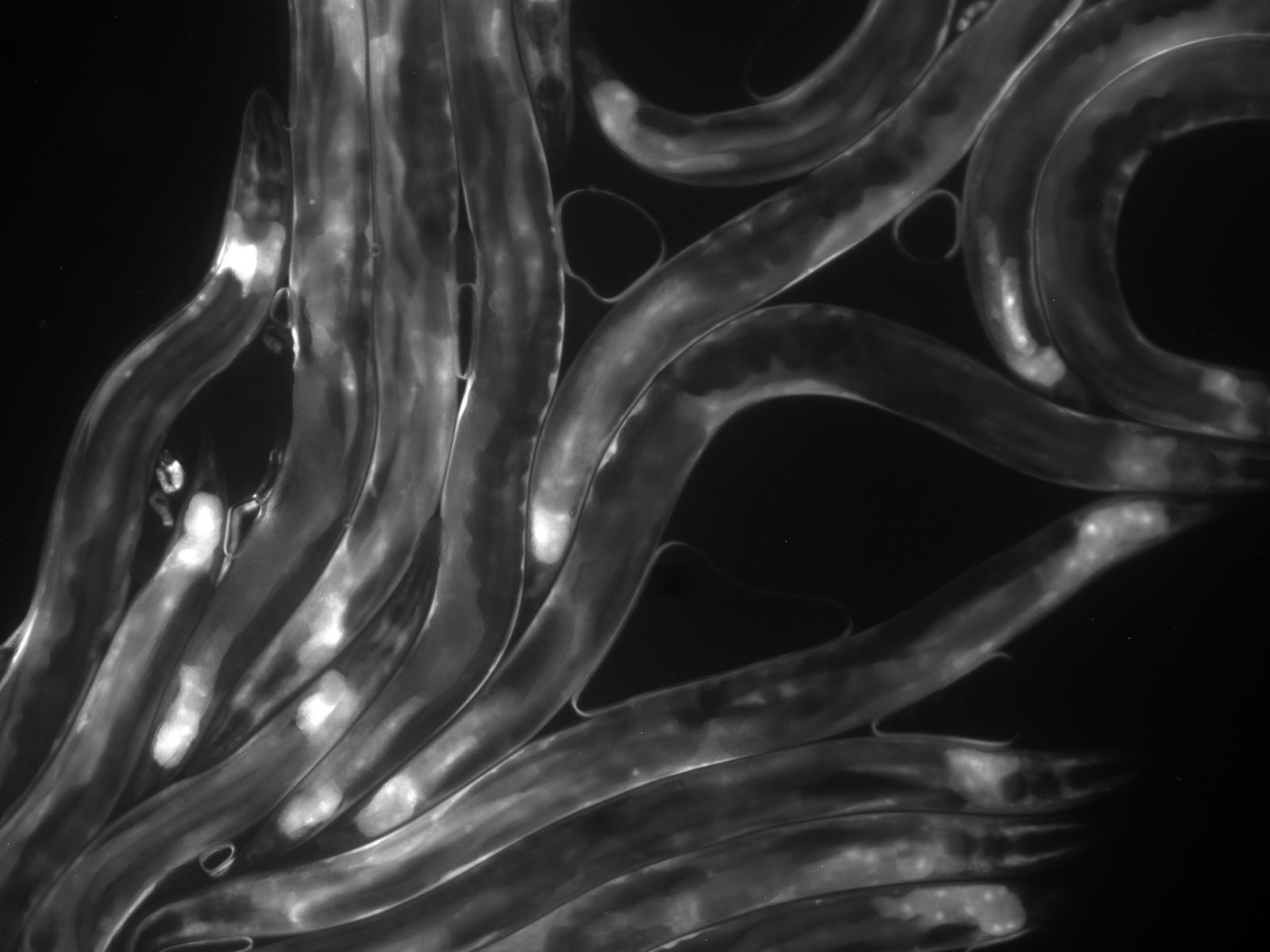

Supplement: S4 File — Since images include multiple worms, images were independently measured several times to achieve measurements for distinct worms in one shared image. Only animals whose body was fully imaged (at least from front to back intestine) were measured. Exposure levels were maintained constant per worm strain. (ZIP) [file pgen.1011061.s004.zip › Fig.S1 - Original files/Fig S1 RAW data and photos - JPEG/reporters on PAD12 or TFG-1 RNAi - 11.5.23 _ 2 rep JPEG/cl2166_gst-4_gfp+ pad12 -day121.jpg]

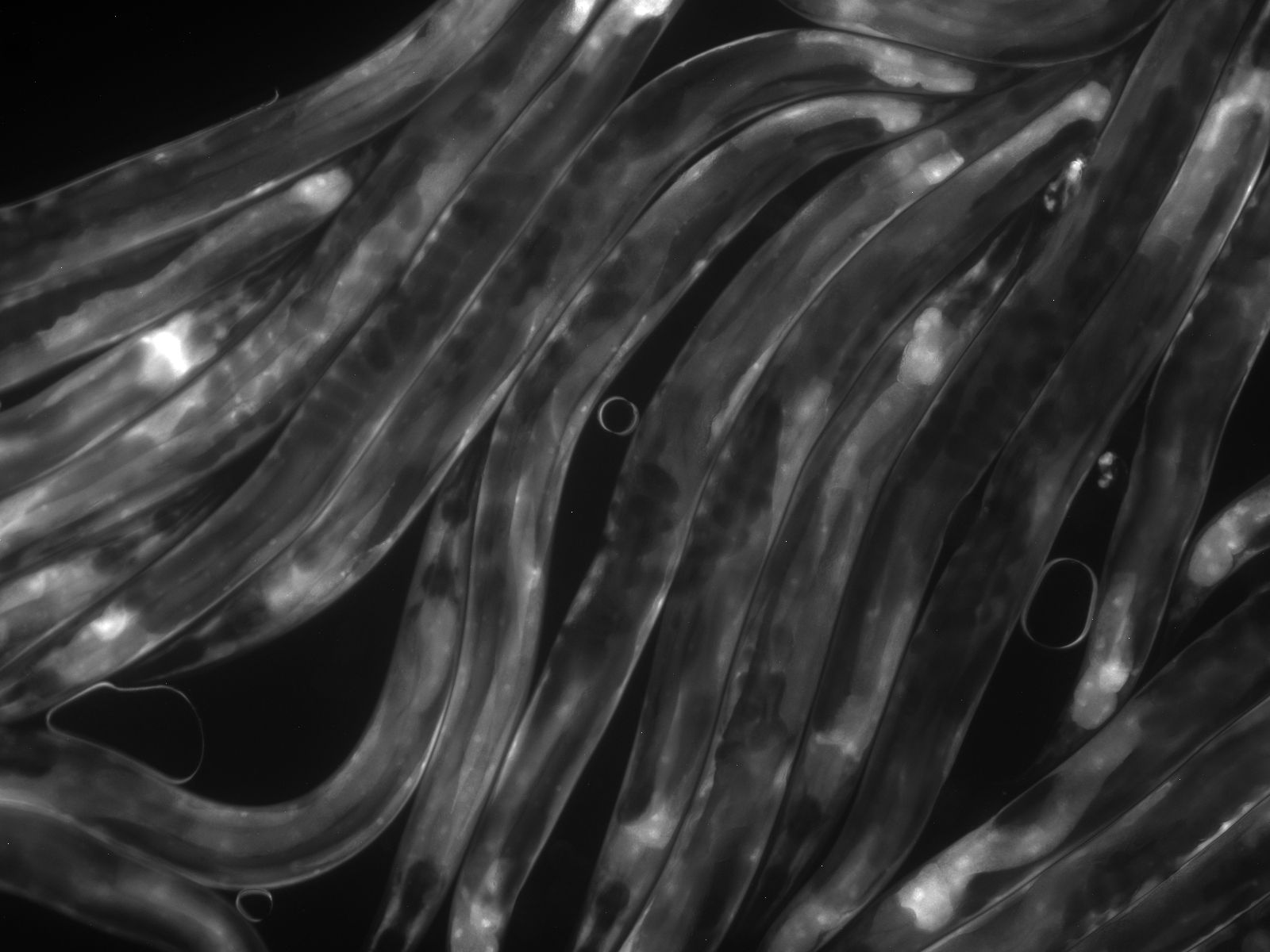

Supplement: S4 File — Since images include multiple worms, images were independently measured several times to achieve measurements for distinct worms in one shared image. Only animals whose body was fully imaged (at least from front to back intestine) were measured. Exposure levels were maintained constant per worm strain. (ZIP) [file pgen.1011061.s004.zip › Fig.S1 - Original files/Fig S1 RAW data and photos - JPEG/reporters on PAD12 or TFG-1 RNAi - 11.5.23 _ 2 rep JPEG/cl2166_gst-4_gfp+ pad12 -day122.jpg]

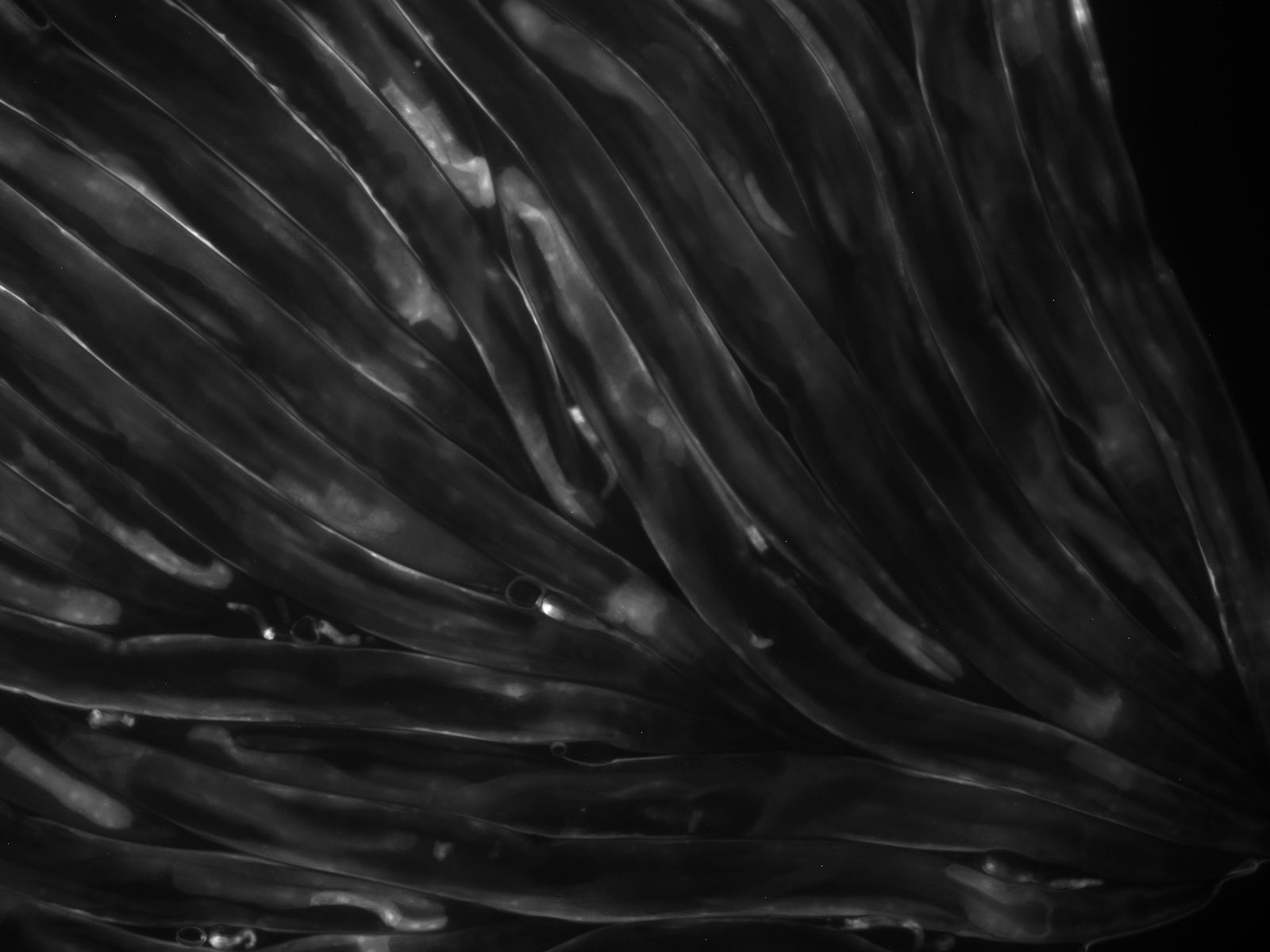

Supplement: S4 File — Since images include multiple worms, images were independently measured several times to achieve measurements for distinct worms in one shared image. Only animals whose body was fully imaged (at least from front to back intestine) were measured. Exposure levels were maintained constant per worm strain. (ZIP) [file pgen.1011061.s004.zip › Fig.S1 - Original files/Fig S1 RAW data and photos - JPEG/reporters on PAD12 or TFG-1 RNAi - 11.5.23 _ 2 rep JPEG/cl2166_gst-4_gfp+tfg-1 -day123.jpg]

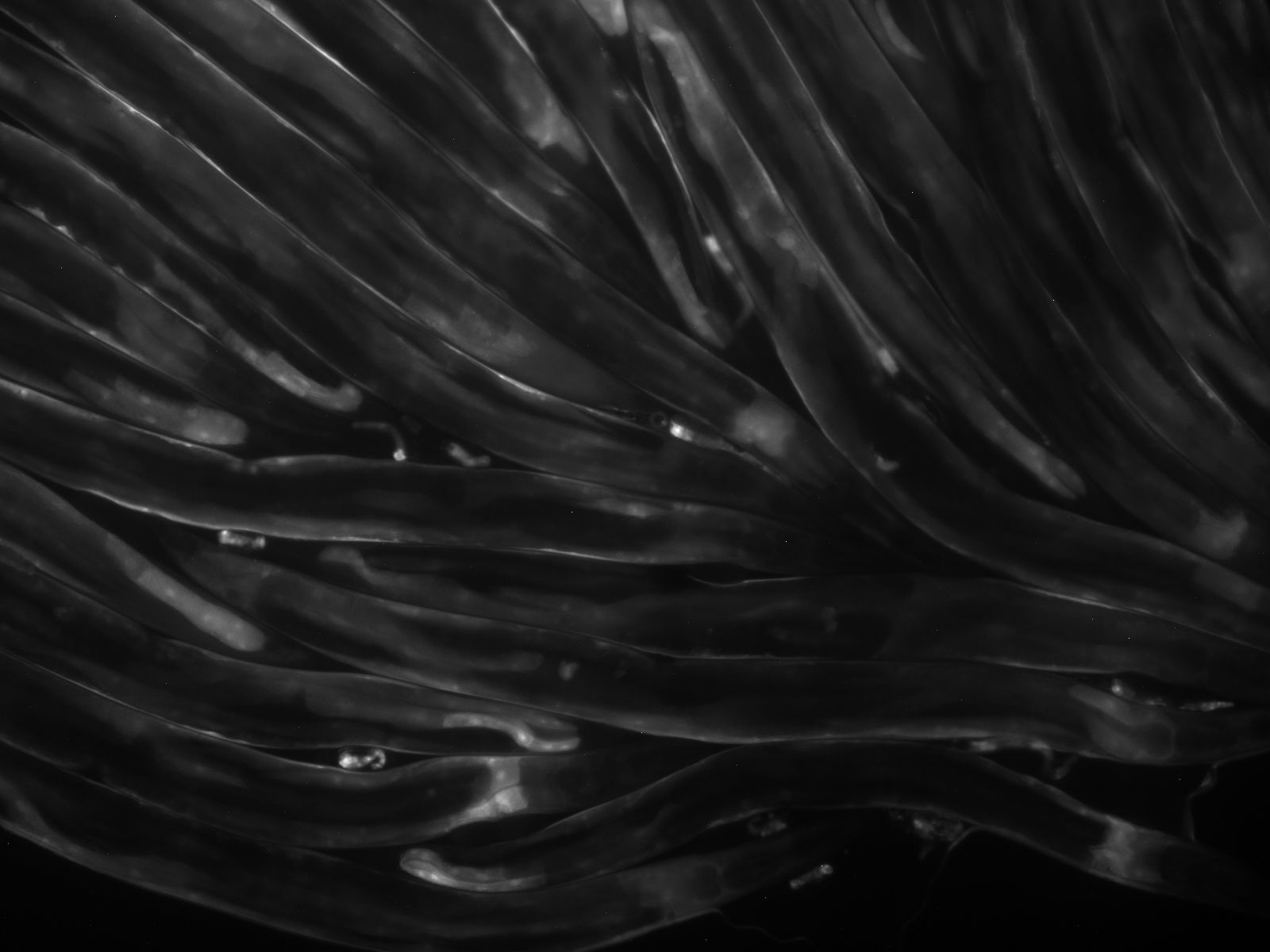

Supplement: S4 File — Since images include multiple worms, images were independently measured several times to achieve measurements for distinct worms in one shared image. Only animals whose body was fully imaged (at least from front to back intestine) were measured. Exposure levels were maintained constant per worm strain. (ZIP) [file pgen.1011061.s004.zip › Fig.S1 - Original files/Fig S1 RAW data and photos - JPEG/reporters on PAD12 or TFG-1 RNAi - 11.5.23 _ 2 rep JPEG/cl2166_gst-4_gfp+tfg-1 -day124.jpg]

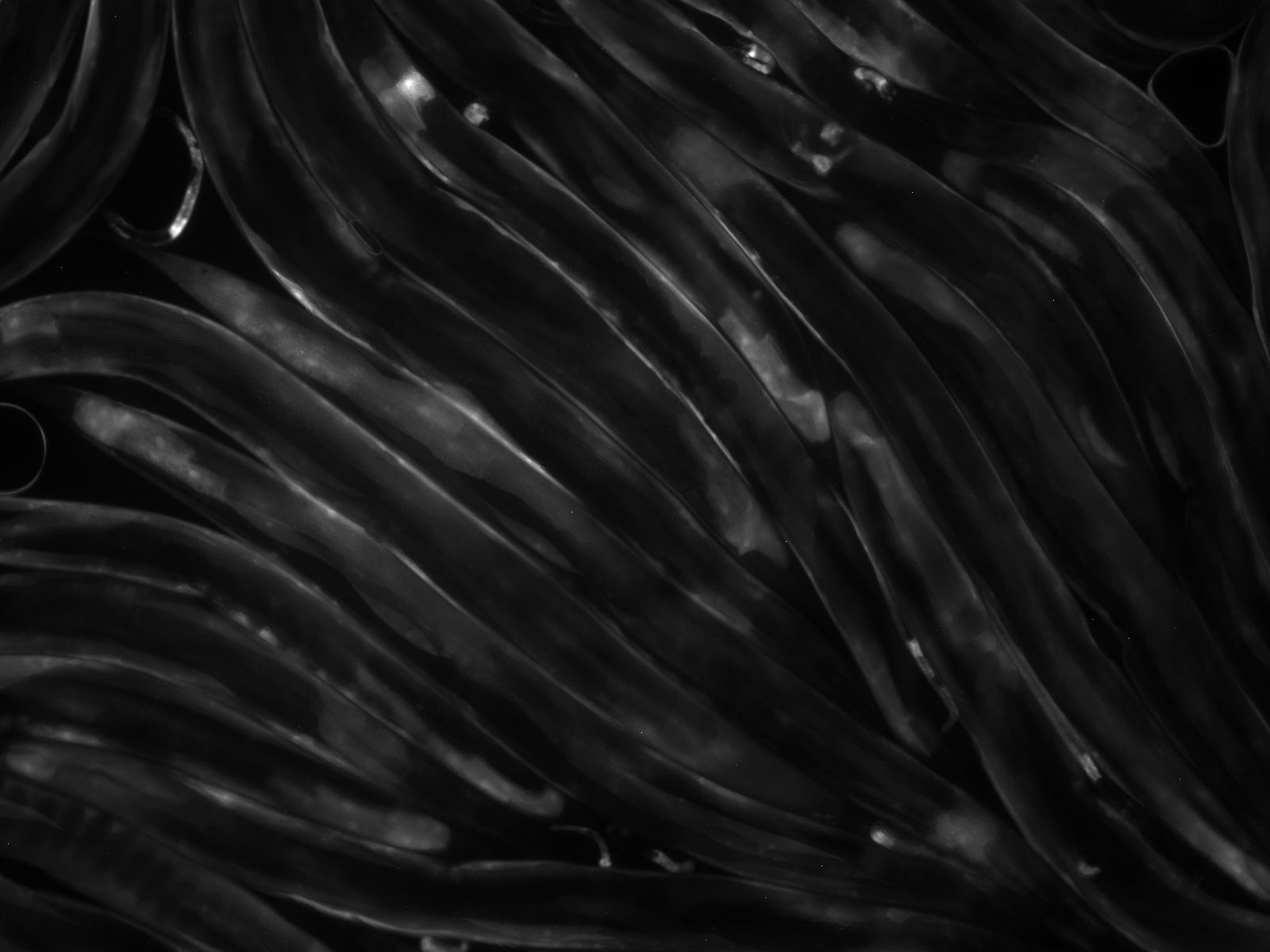

Supplement: S4 File — Since images include multiple worms, images were independently measured several times to achieve measurements for distinct worms in one shared image. Only animals whose body was fully imaged (at least from front to back intestine) were measured. Exposure levels were maintained constant per worm strain. (ZIP) [file pgen.1011061.s004.zip › Fig.S1 - Original files/Fig S1 RAW data and photos - JPEG/reporters on PAD12 or TFG-1 RNAi - 11.5.23 _ 2 rep JPEG/cl2166_gst-4_gfp+tfg-1 -day125.jpg]

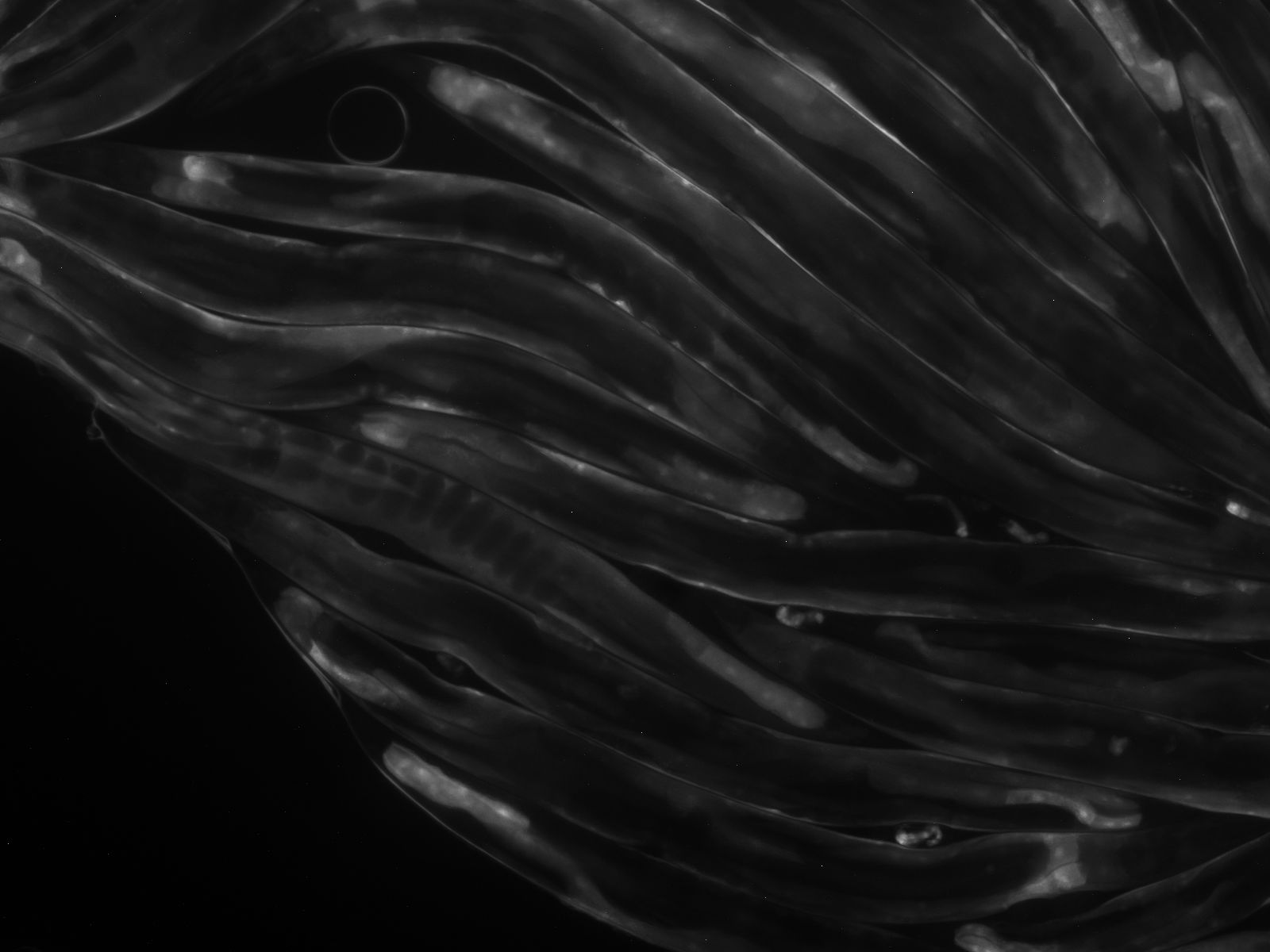

Supplement: S4 File — Since images include multiple worms, images were independently measured several times to achieve measurements for distinct worms in one shared image. Only animals whose body was fully imaged (at least from front to back intestine) were measured. Exposure levels were maintained constant per worm strain. (ZIP) [file pgen.1011061.s004.zip › Fig.S1 - Original files/Fig S1 RAW data and photos - JPEG/reporters on PAD12 or TFG-1 RNAi - 11.5.23 _ 2 rep JPEG/cl2166_gst-4_gfp+tfg-1 -day126.jpg]

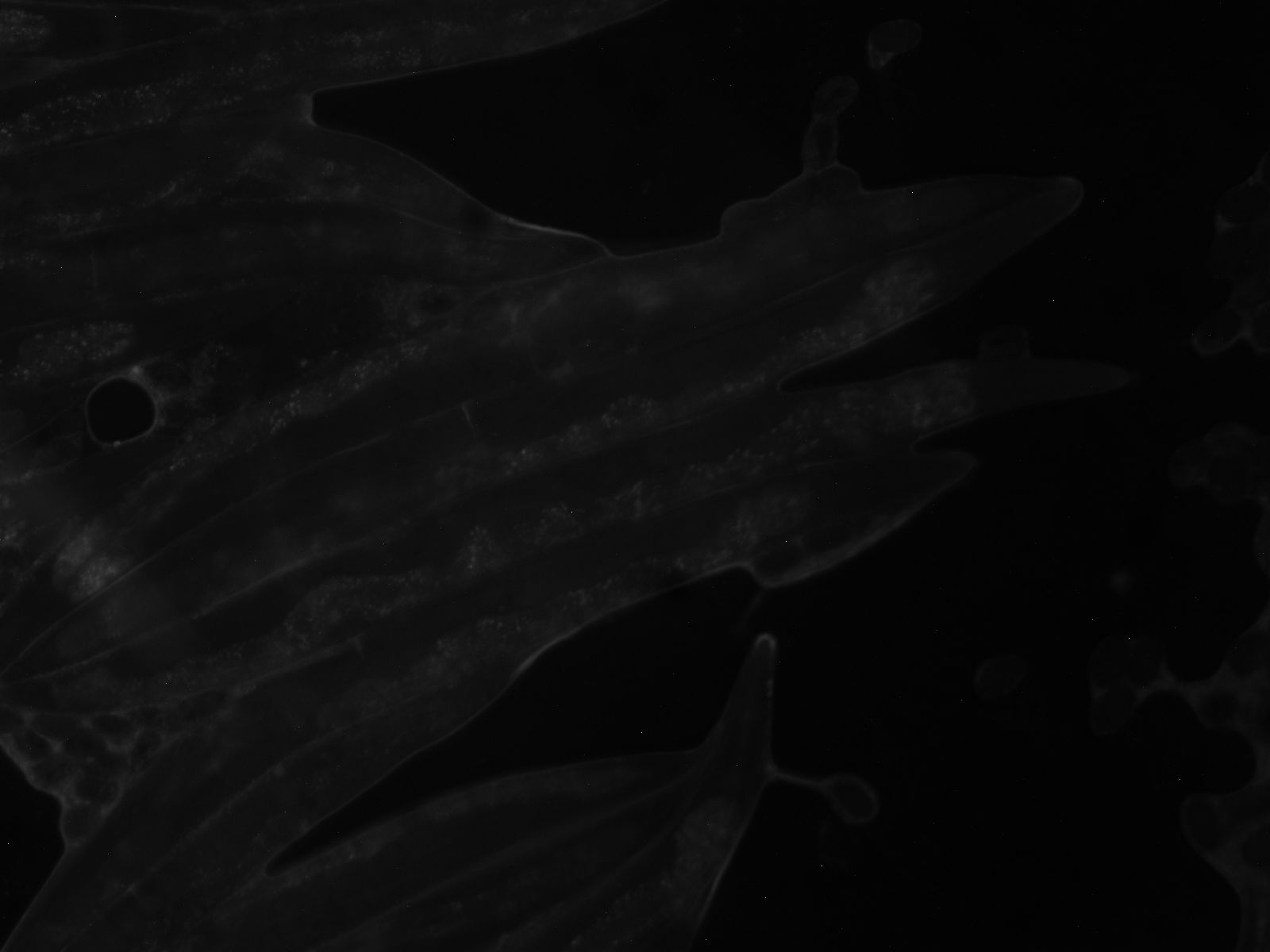

Supplement: S4 File — Since images include multiple worms, images were independently measured several times to achieve measurements for distinct worms in one shared image. Only animals whose body was fully imaged (at least from front to back intestine) were measured. Exposure levels were maintained constant per worm strain. (ZIP) [file pgen.1011061.s004.zip › Fig.S1 - Original files/Fig S1 RAW data and photos - JPEG/reporters on PAD12 or TFG-1 RNAi - 11.5.23 _ 2 rep JPEG/hsp-16.2_gfp+pad12-day160.jpg]

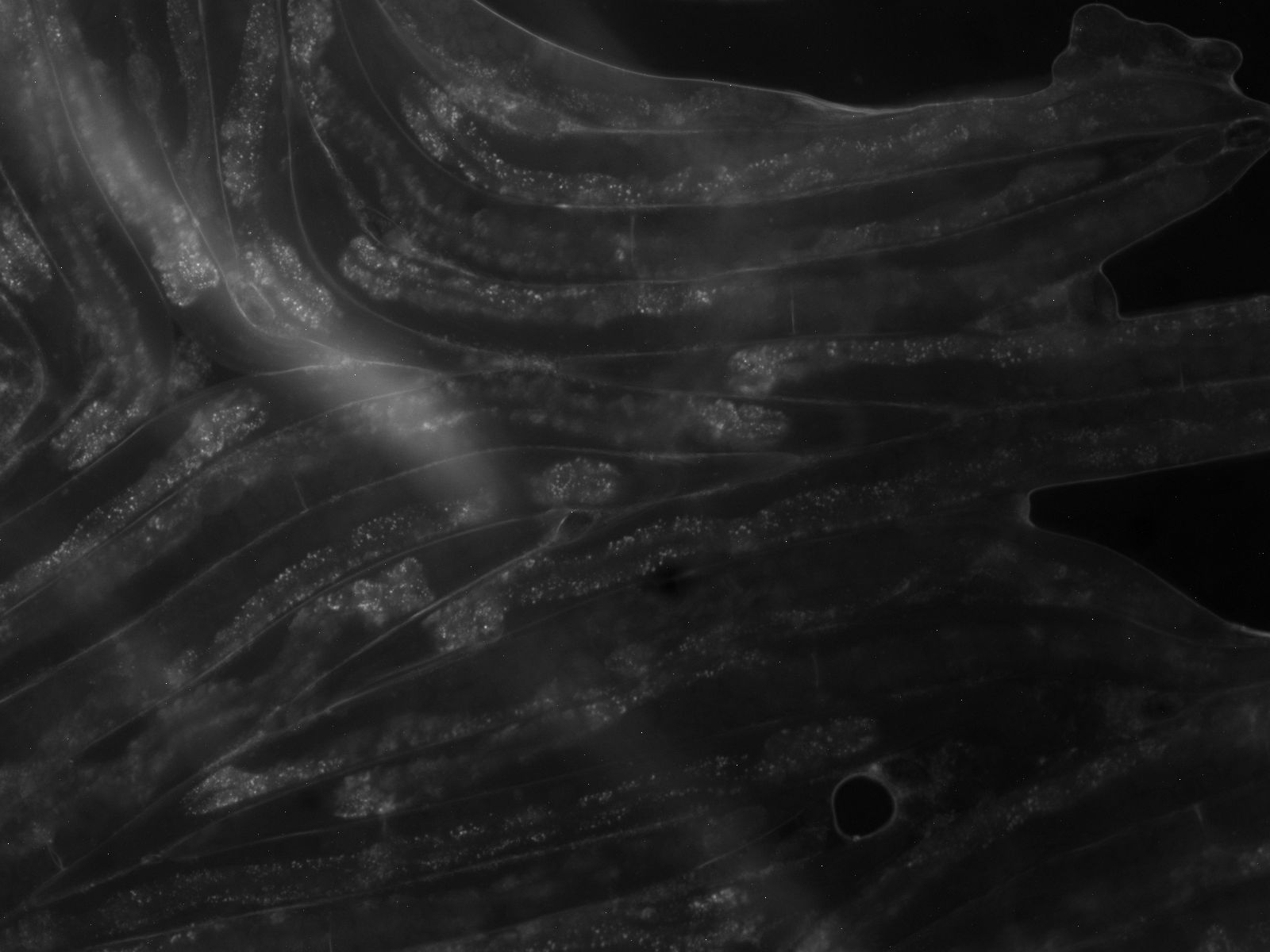

Supplement: S4 File — Since images include multiple worms, images were independently measured several times to achieve measurements for distinct worms in one shared image. Only animals whose body was fully imaged (at least from front to back intestine) were measured. Exposure levels were maintained constant per worm strain. (ZIP) [file pgen.1011061.s004.zip › Fig.S1 - Original files/Fig S1 RAW data and photos - JPEG/reporters on PAD12 or TFG-1 RNAi - 11.5.23 _ 2 rep JPEG/hsp-16.2_gfp+pad12-day161.jpg]

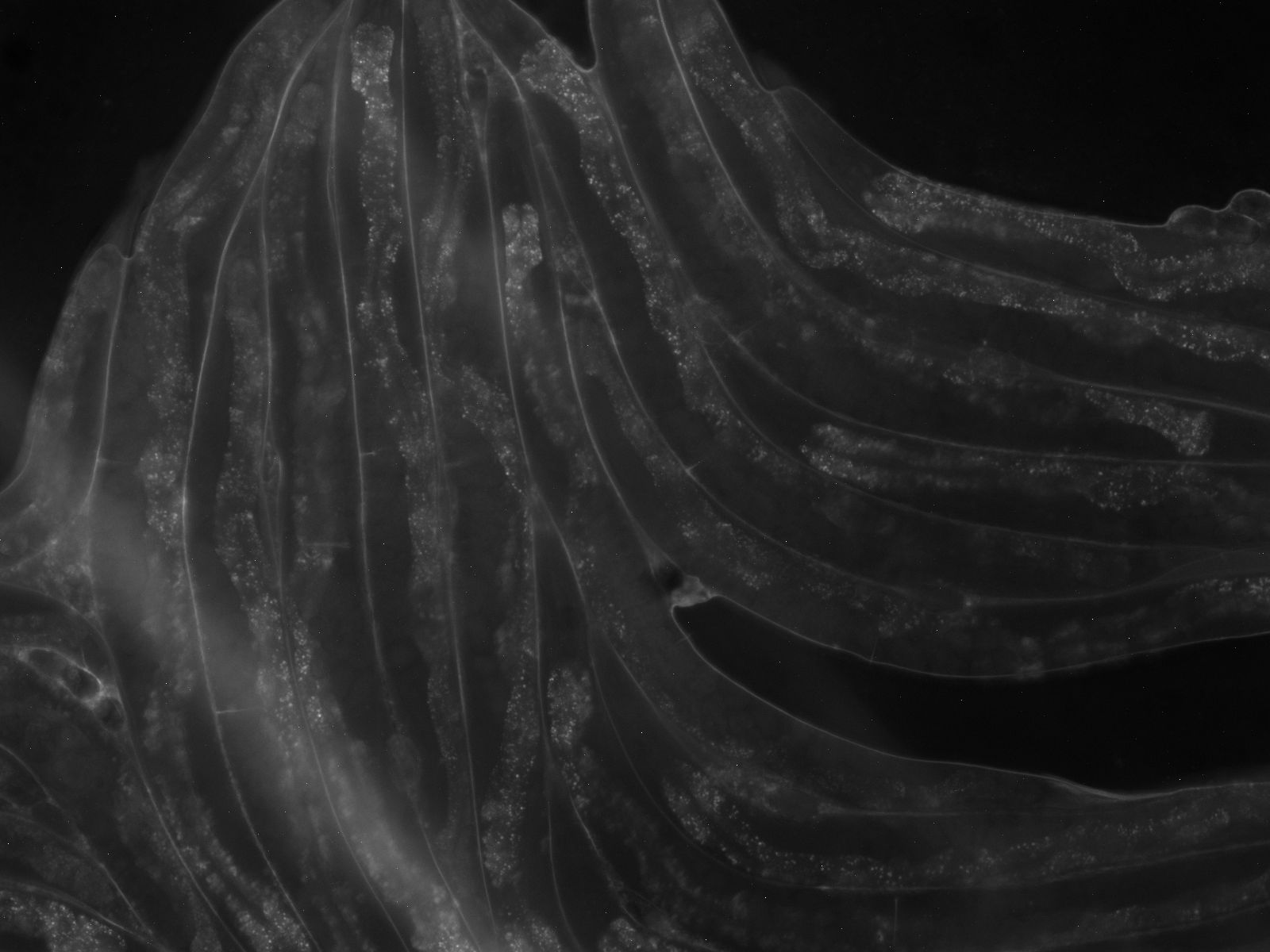

Supplement: S4 File — Since images include multiple worms, images were independently measured several times to achieve measurements for distinct worms in one shared image. Only animals whose body was fully imaged (at least from front to back intestine) were measured. Exposure levels were maintained constant per worm strain. (ZIP) [file pgen.1011061.s004.zip › Fig.S1 - Original files/Fig S1 RAW data and photos - JPEG/reporters on PAD12 or TFG-1 RNAi - 11.5.23 _ 2 rep JPEG/hsp-16.2_gfp+pad12-day162.jpg]

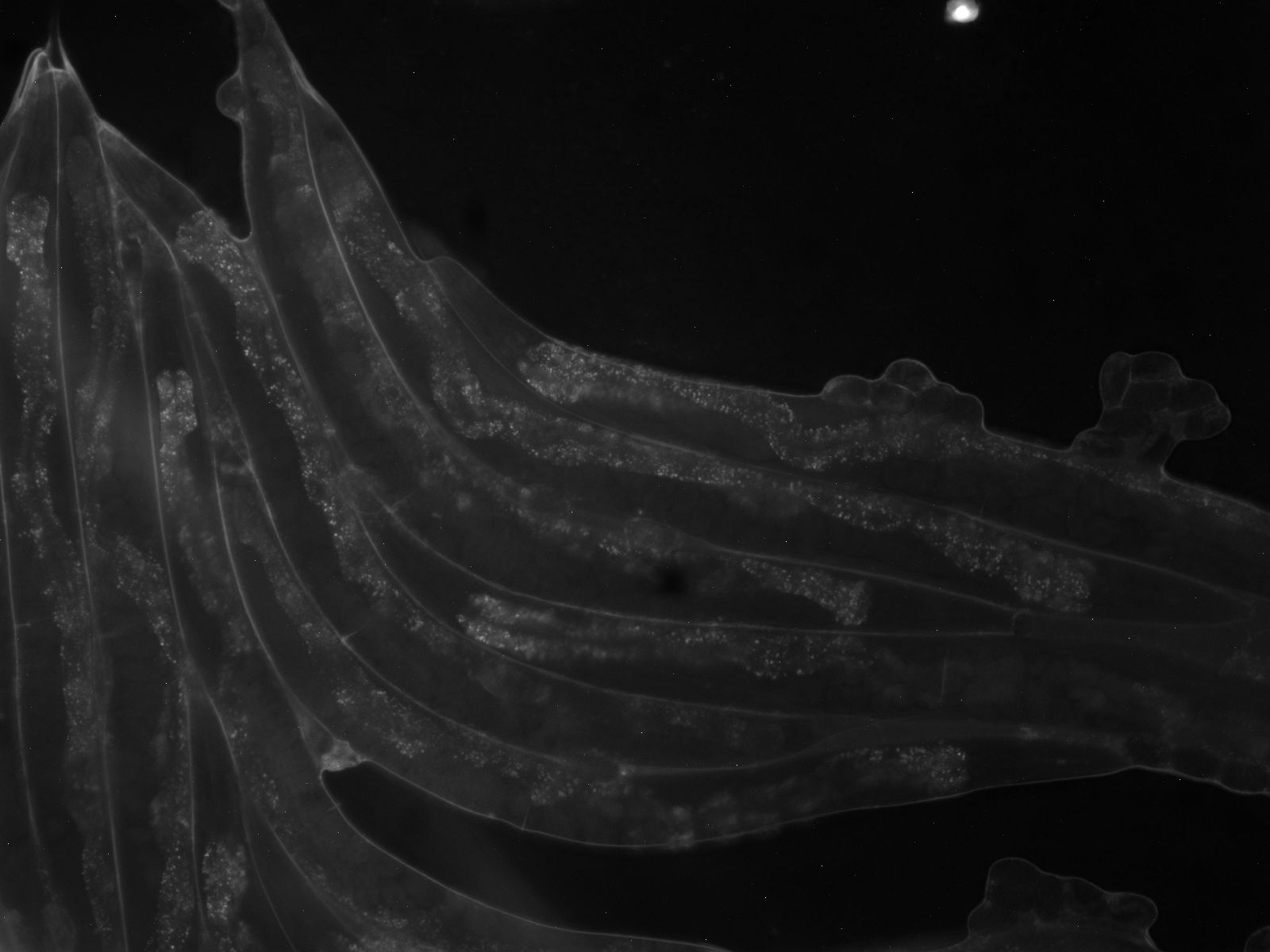

Supplement: S4 File — Since images include multiple worms, images were independently measured several times to achieve measurements for distinct worms in one shared image. Only animals whose body was fully imaged (at least from front to back intestine) were measured. Exposure levels were maintained constant per worm strain. (ZIP) [file pgen.1011061.s004.zip › Fig.S1 - Original files/Fig S1 RAW data and photos - JPEG/reporters on PAD12 or TFG-1 RNAi - 11.5.23 _ 2 rep JPEG/hsp-16.2_gfp+pad12-day163.jpg]

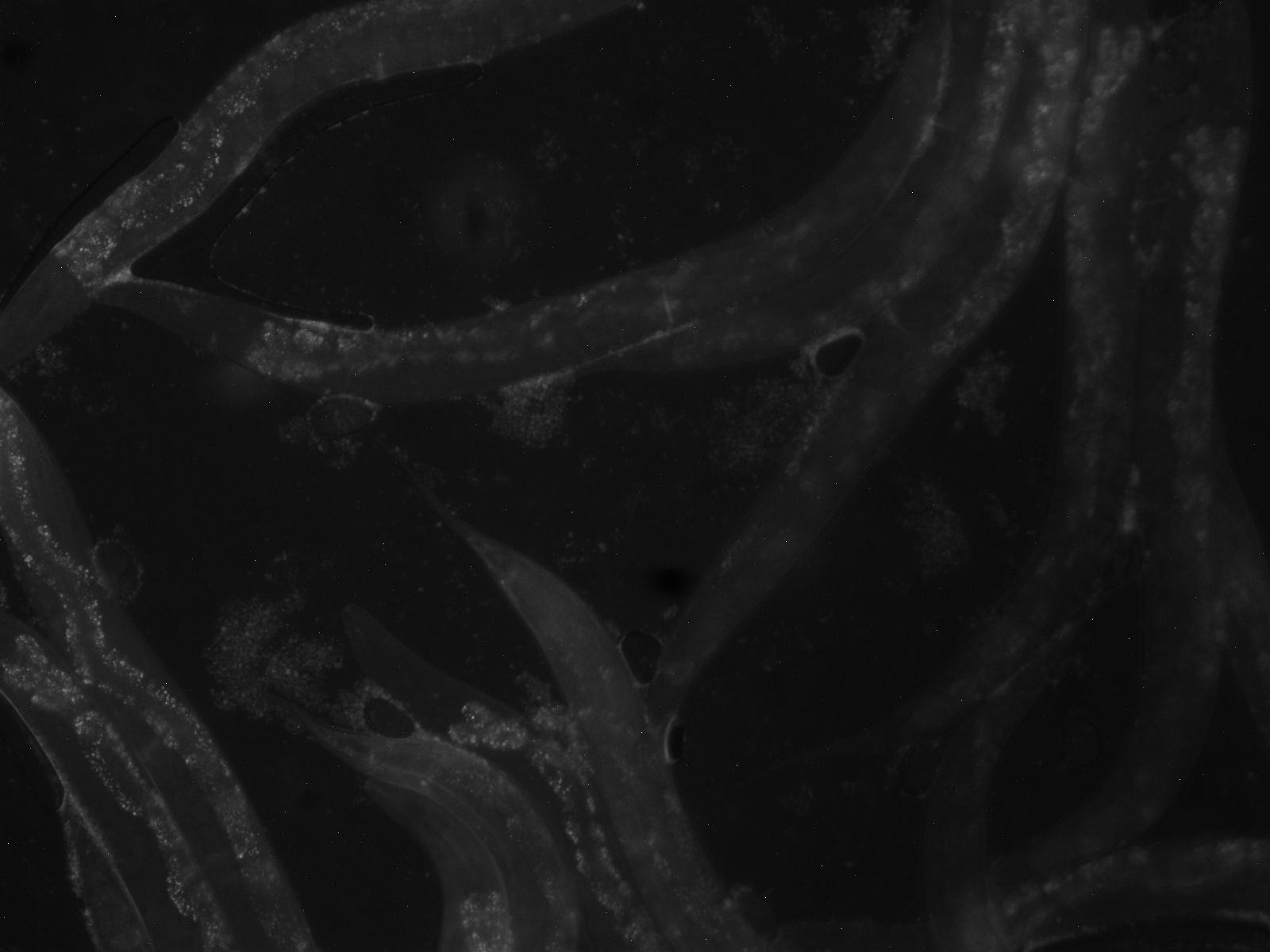

Supplement: S4 File — Since images include multiple worms, images were independently measured several times to achieve measurements for distinct worms in one shared image. Only animals whose body was fully imaged (at least from front to back intestine) were measured. Exposure levels were maintained constant per worm strain. (ZIP) [file pgen.1011061.s004.zip › Fig.S1 - Original files/Fig S1 RAW data and photos - JPEG/reporters on PAD12 or TFG-1 RNAi - 11.5.23 _ 2 rep JPEG/hsp-16.2_gfp+tfg-1-day164.jpg]
