# Supplementary figures and images for: Correction: It’s All in Your Mind: Determining Germ Cell Fate by Neuronal IRE-1 in C. elegans (part 3 of 7)
Source: PLoS Genet. 2023 Nov 30;19(11):e1011061. doi: 10.1371/journal.pgen.1011061 (PMC10688620; doi:10.1371/journal.pgen.1011061)

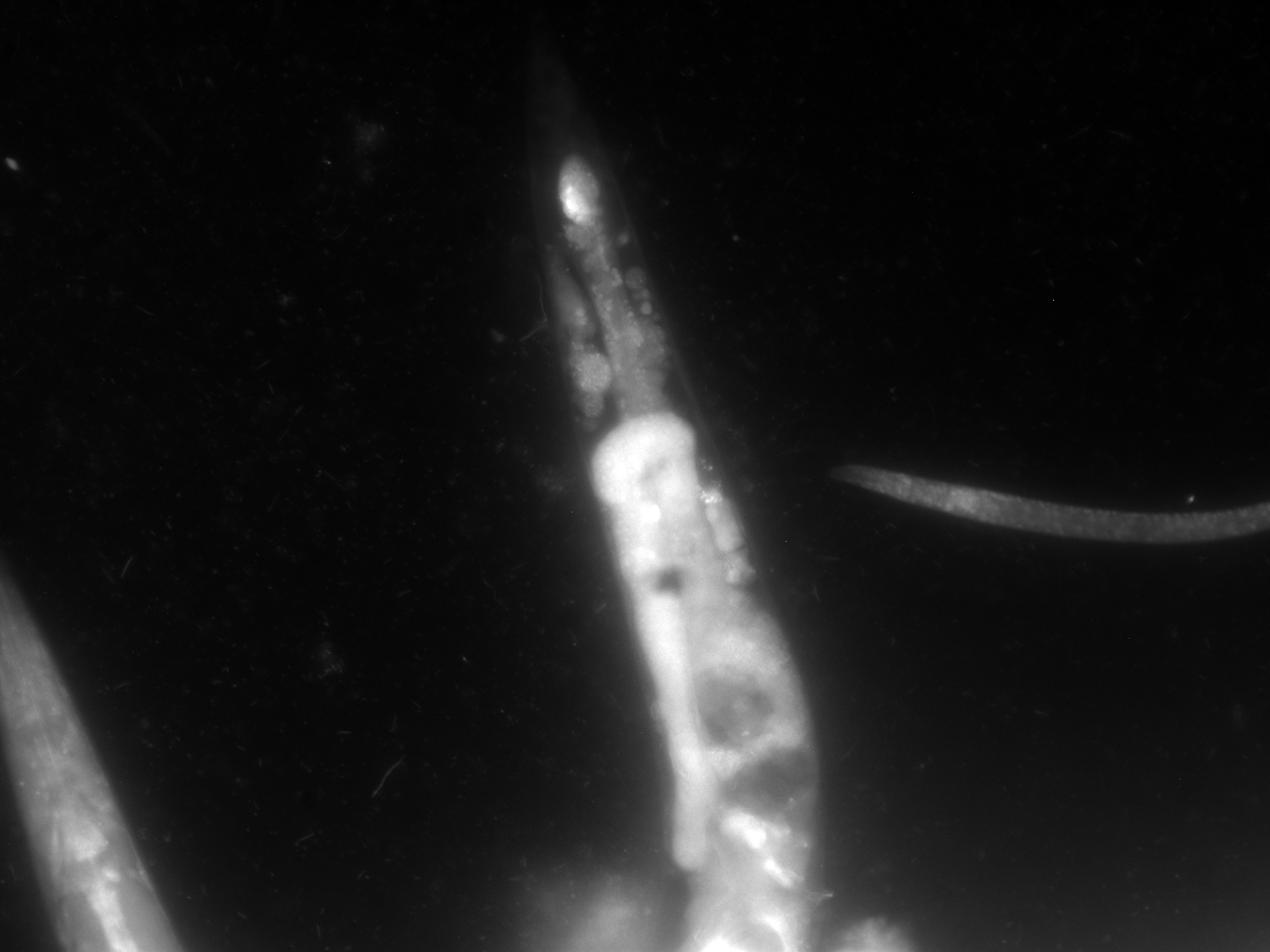

Supplement: S2 File — (ZIP) [file pgen.1011061.s002.zip › Fig.2A - Original files/Fig.2A pictures selected for figure 2A/ire-1+tfg1153.tif]

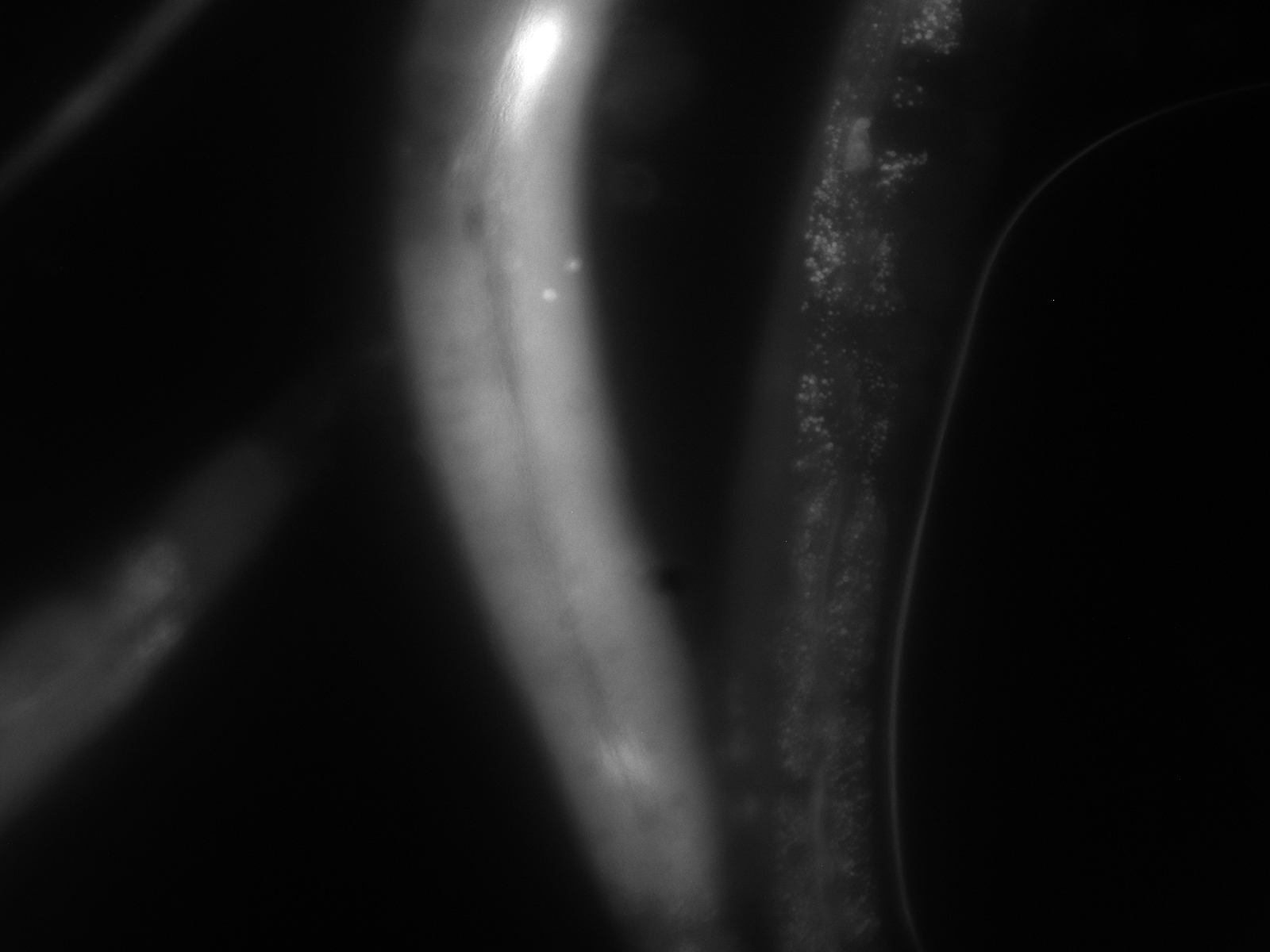

Supplement: S2 File — (ZIP) [file pgen.1011061.s002.zip › Fig.2A - Original files/Fig.2A pictures selected for figure 2A/n2+pad1230.tif]

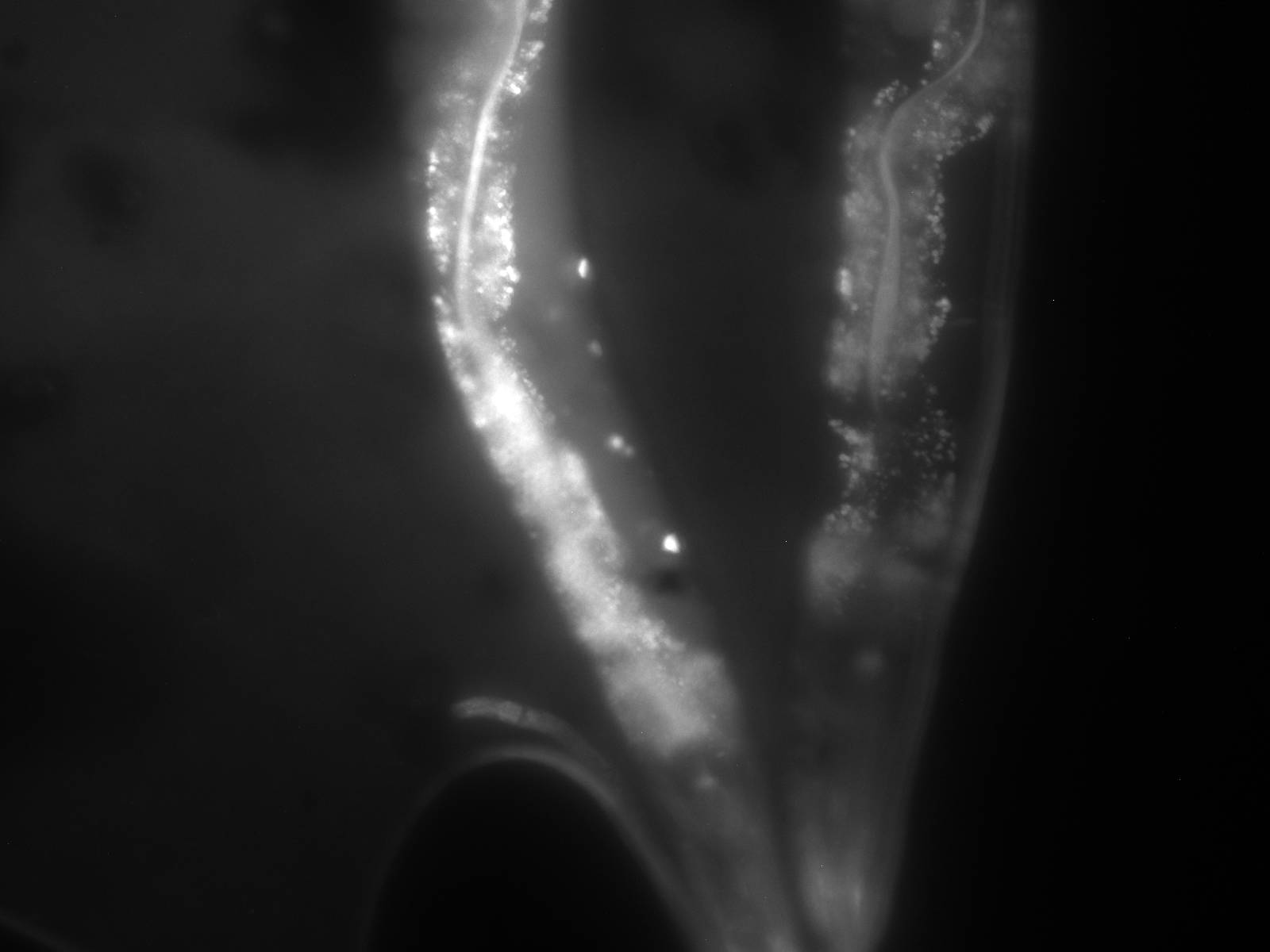

Supplement: S2 File — (ZIP) [file pgen.1011061.s002.zip › Fig.2A - Original files/Fig.2A pictures selected for figure 2A/n2+tfg117.tif]

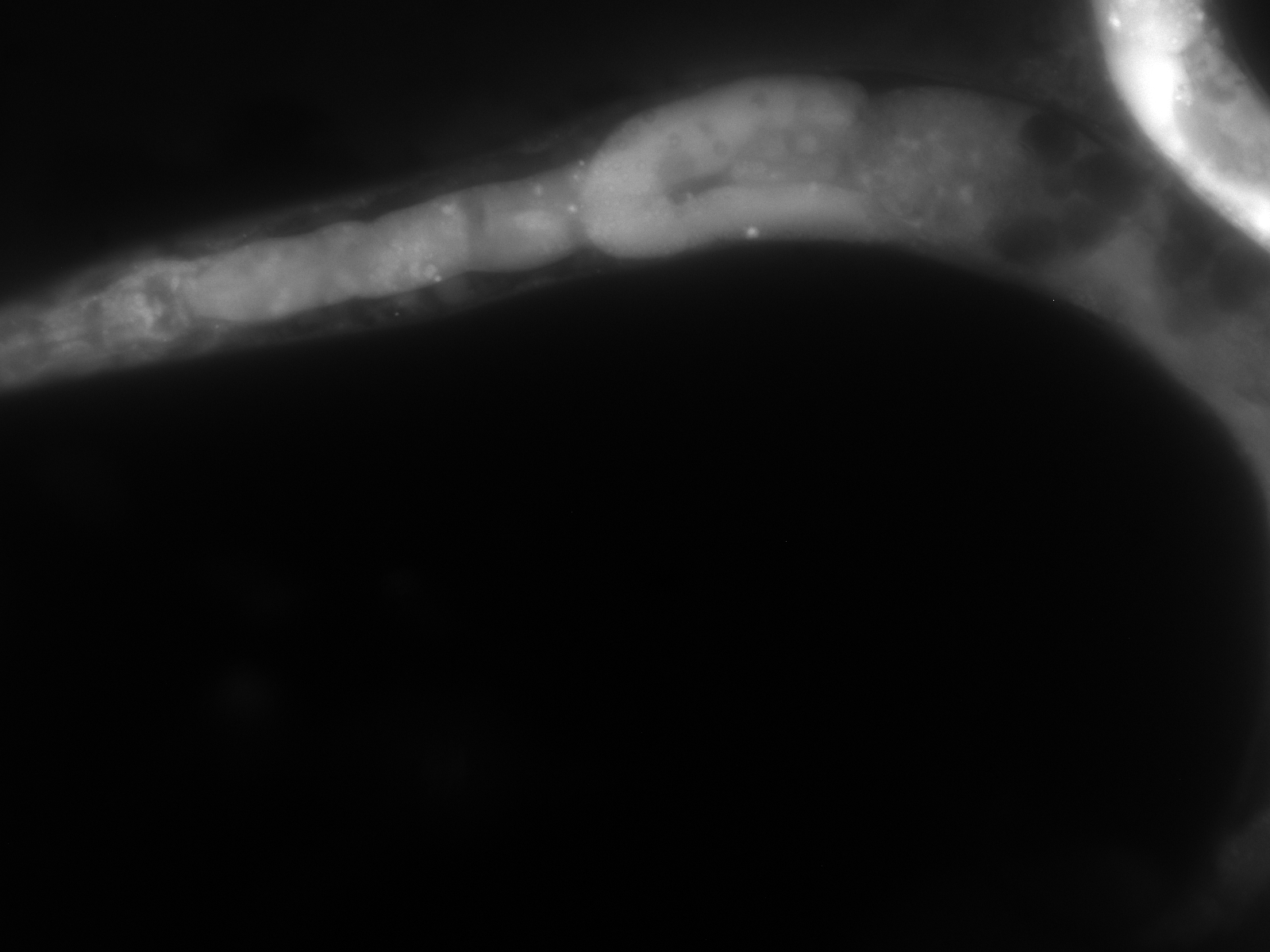

Supplement: S2 File — (ZIP) [file pgen.1011061.s002.zip › Fig.2A - Original files/Fig.2A pictures selected for figure 2A/xbp-1_ire-1+pad12178.tif]

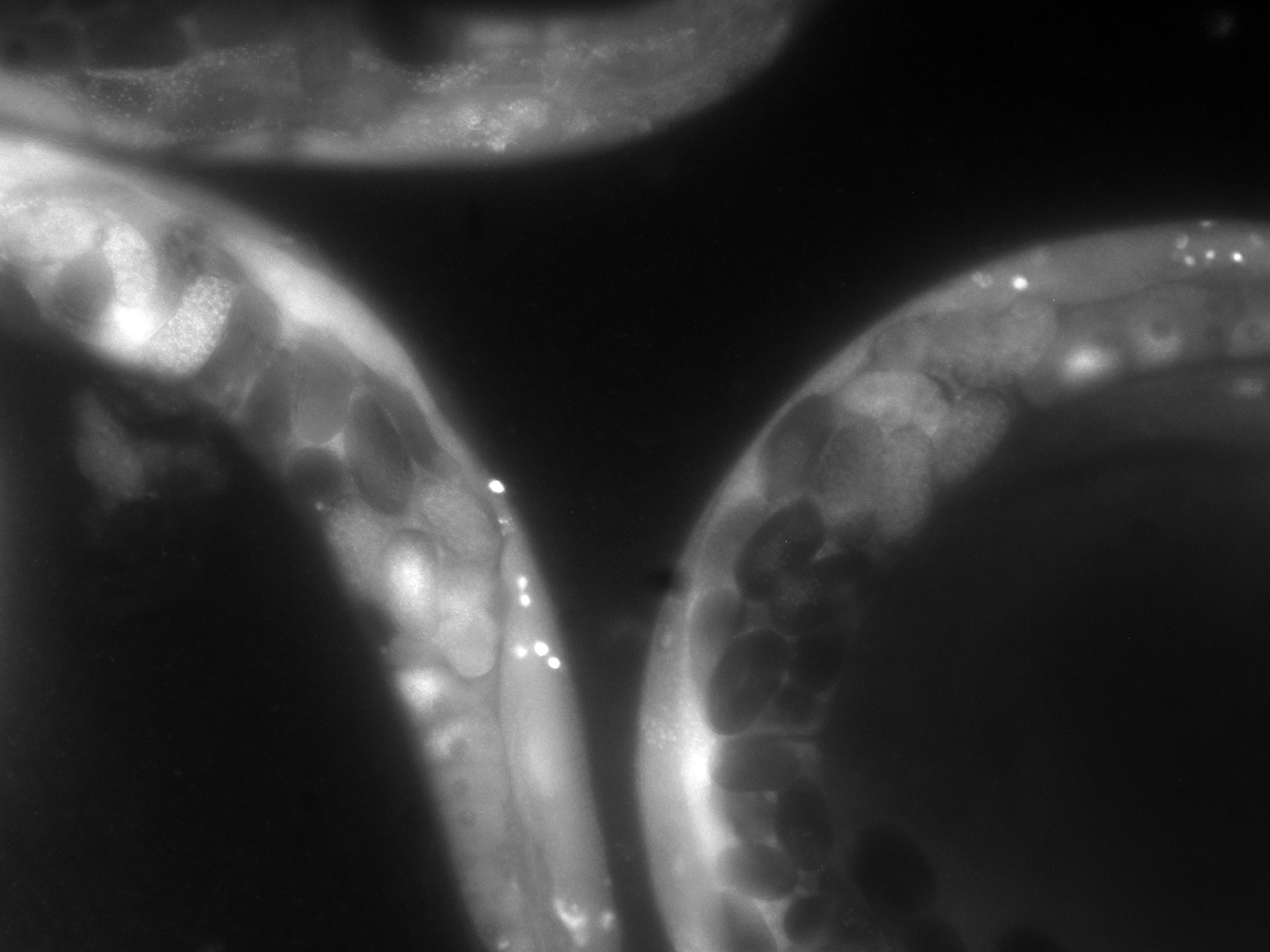

Supplement: S2 File — (ZIP) [file pgen.1011061.s002.zip › Fig.2A - Original files/Fig.2A pictures selected for figure 2A/xbp-1+pad12117.tif]

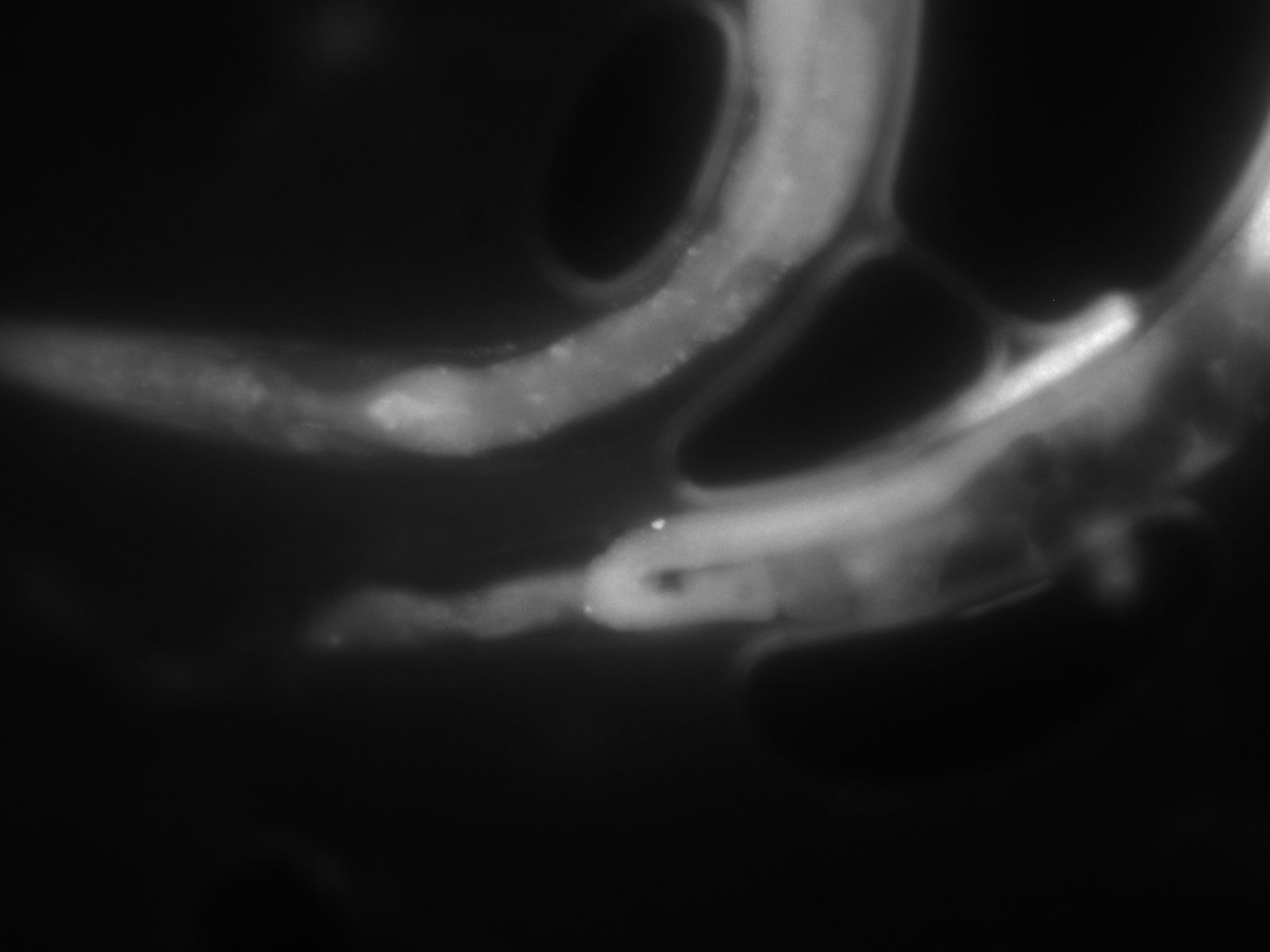

Supplement: S2 File — (ZIP) [file pgen.1011061.s002.zip › Fig.2A - Original files/Fig.2A RAW data and photos JPEG/syto12 staining - fig 2A - 2 rep_15.5.23 jpeg/ire-1+pad1200.jpg]

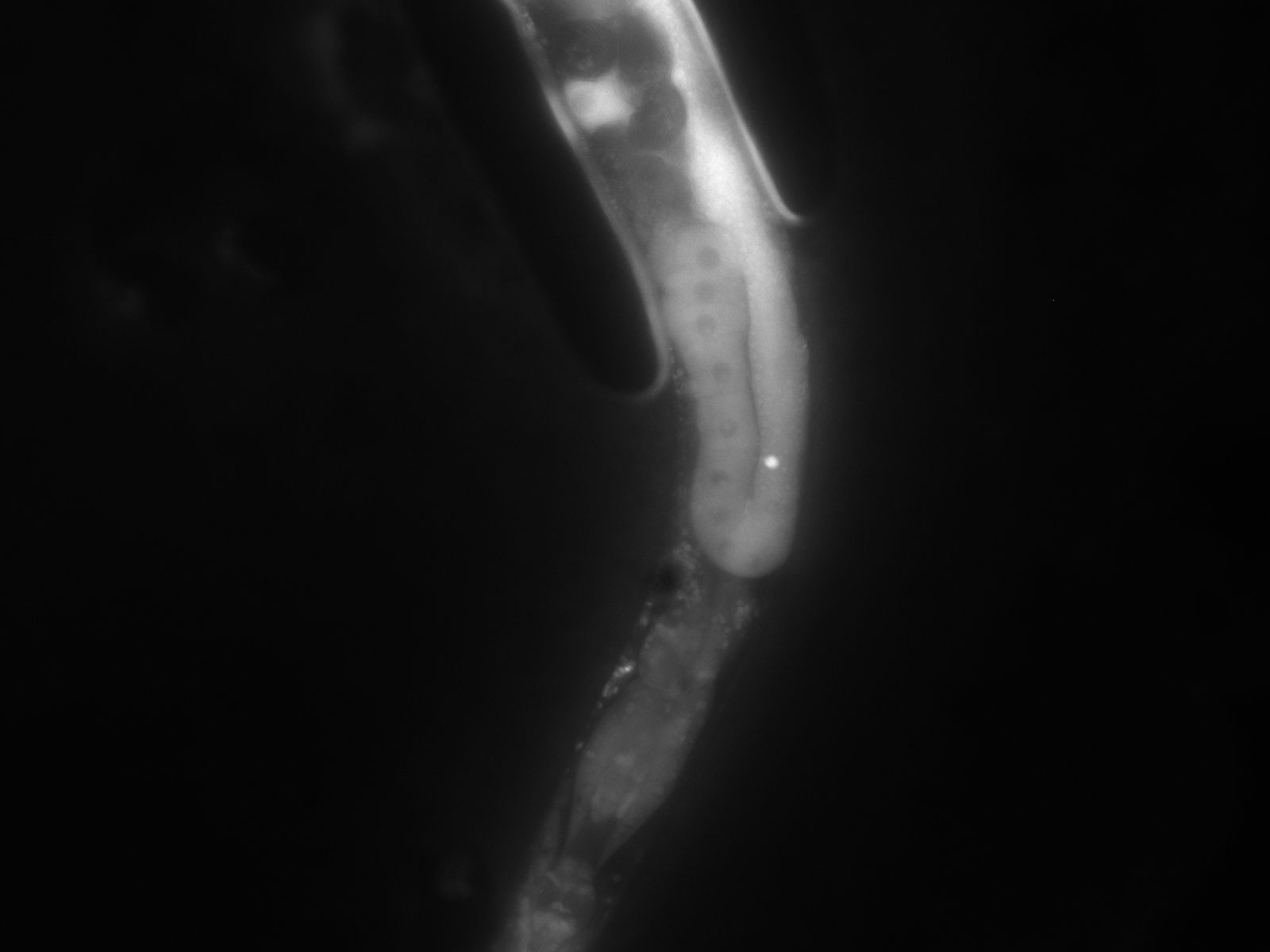

Supplement: S2 File — (ZIP) [file pgen.1011061.s002.zip › Fig.2A - Original files/Fig.2A RAW data and photos JPEG/syto12 staining - fig 2A - 2 rep_15.5.23 jpeg/ire-1+pad1201.jpg]

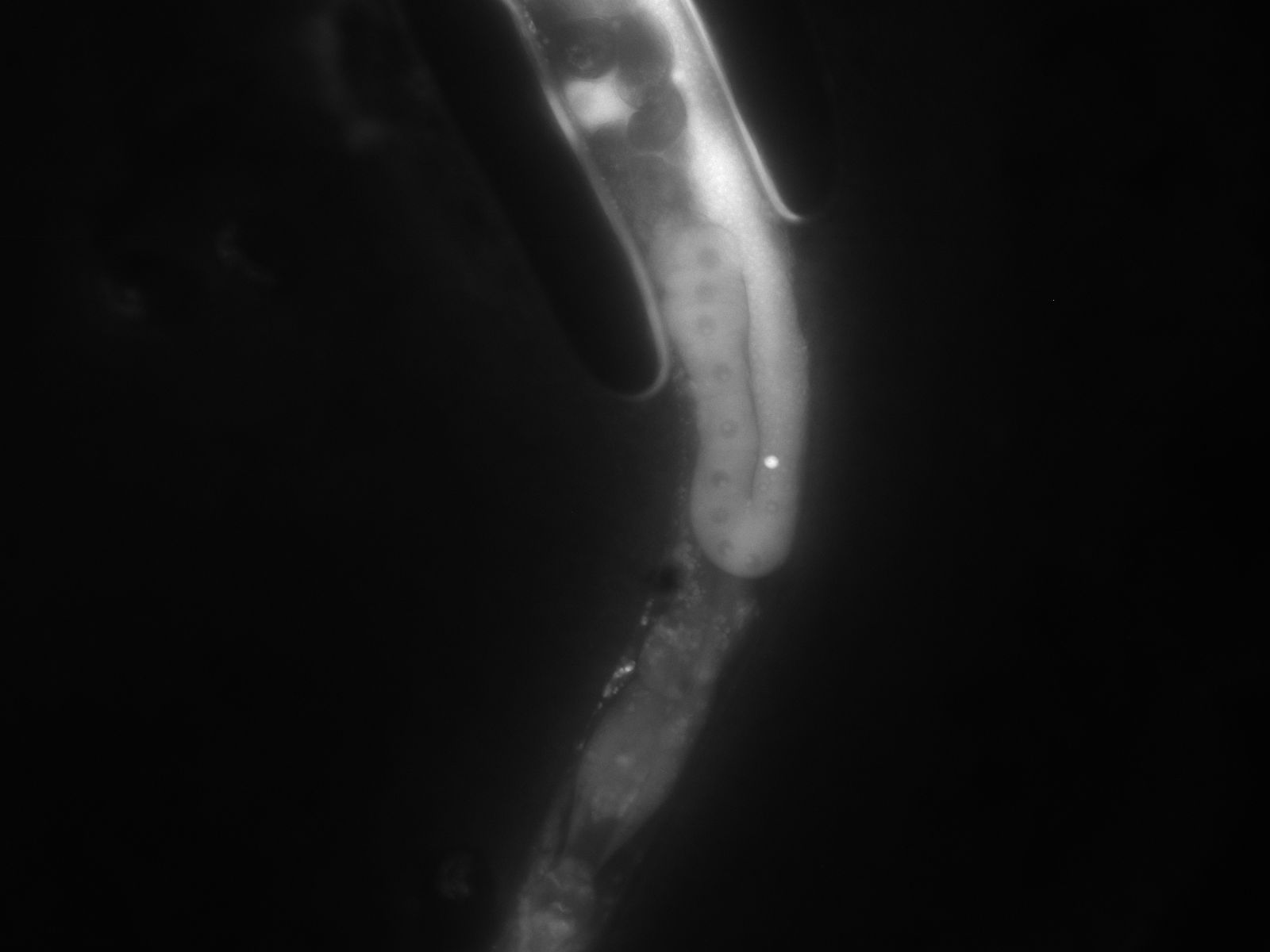

Supplement: S2 File — (ZIP) [file pgen.1011061.s002.zip › Fig.2A - Original files/Fig.2A RAW data and photos JPEG/syto12 staining - fig 2A - 2 rep_15.5.23 jpeg/ire-1+pad1202.jpg]

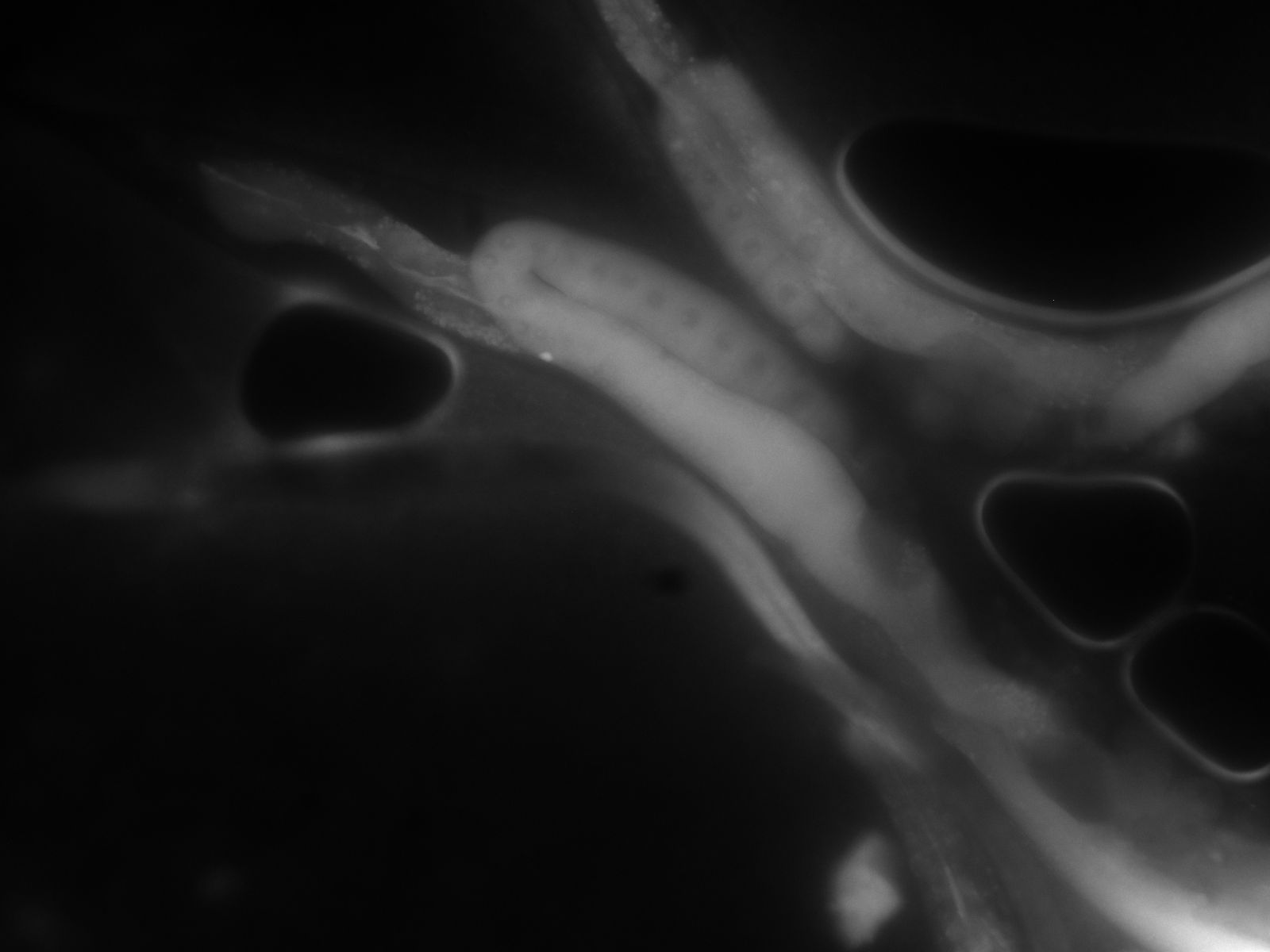

Supplement: S2 File — (ZIP) [file pgen.1011061.s002.zip › Fig.2A - Original files/Fig.2A RAW data and photos JPEG/syto12 staining - fig 2A - 2 rep_15.5.23 jpeg/ire-1+pad1203.jpg]

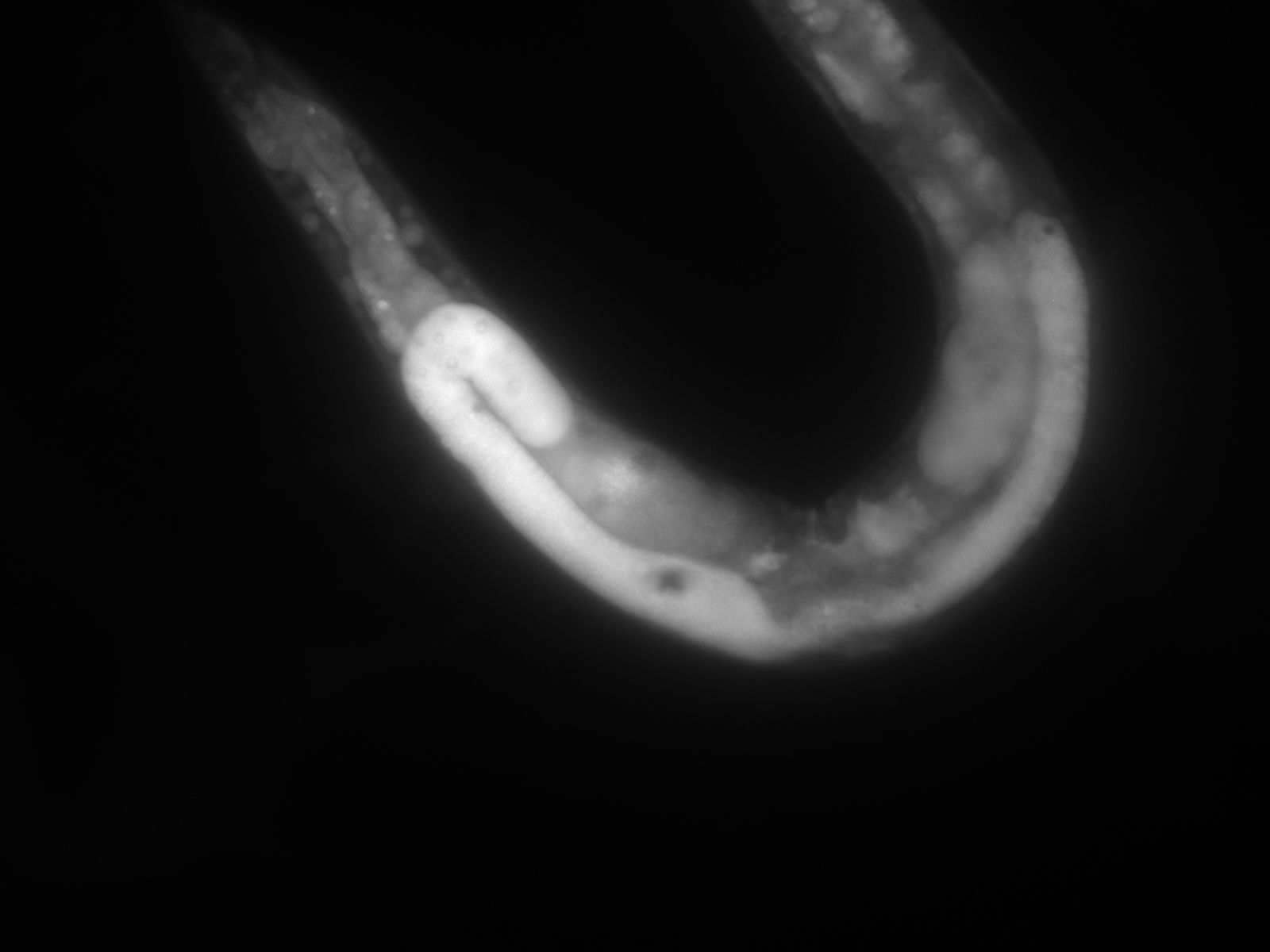

Supplement: S2 File — (ZIP) [file pgen.1011061.s002.zip › Fig.2A - Original files/Fig.2A RAW data and photos JPEG/syto12 staining - fig 2A - 2 rep_15.5.23 jpeg/ire-1+tfg-104.jpg]

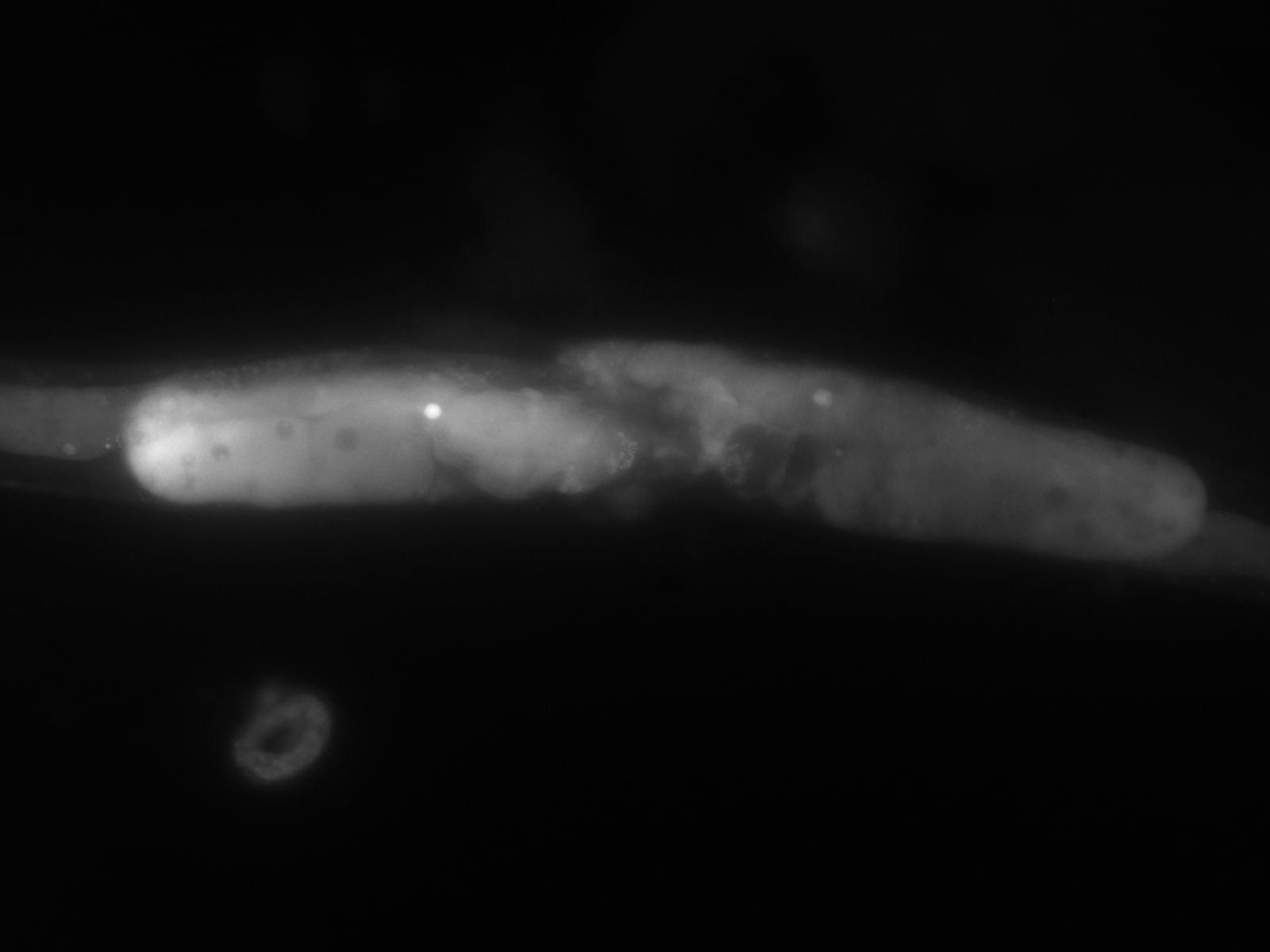

Supplement: S2 File — (ZIP) [file pgen.1011061.s002.zip › Fig.2A - Original files/Fig.2A RAW data and photos JPEG/syto12 staining - fig 2A - 2 rep_15.5.23 jpeg/ire-1+tfg-105.jpg]

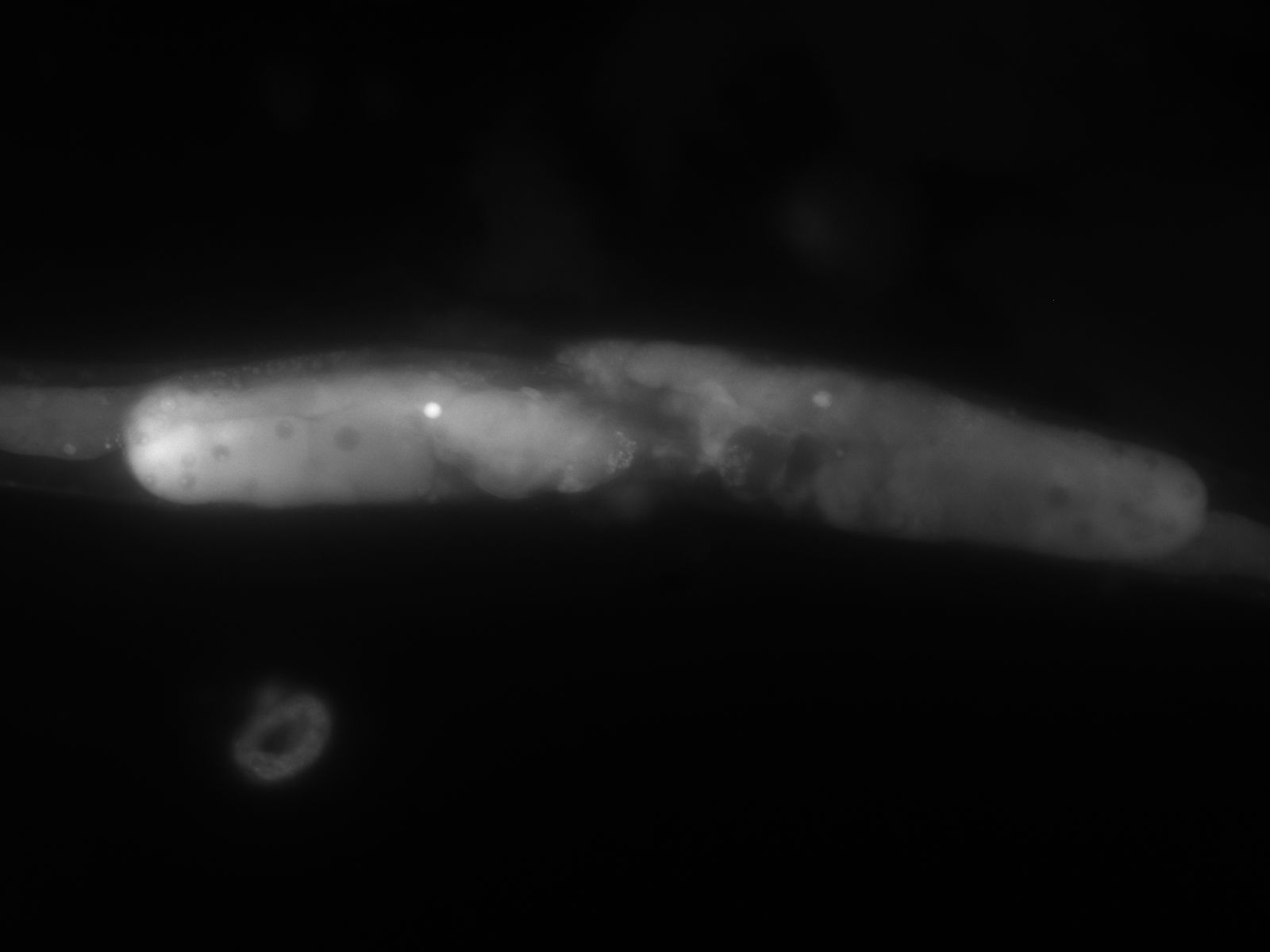

Supplement: S2 File — (ZIP) [file pgen.1011061.s002.zip › Fig.2A - Original files/Fig.2A RAW data and photos JPEG/syto12 staining - fig 2A - 2 rep_15.5.23 jpeg/ire-1+tfg-106.jpg]

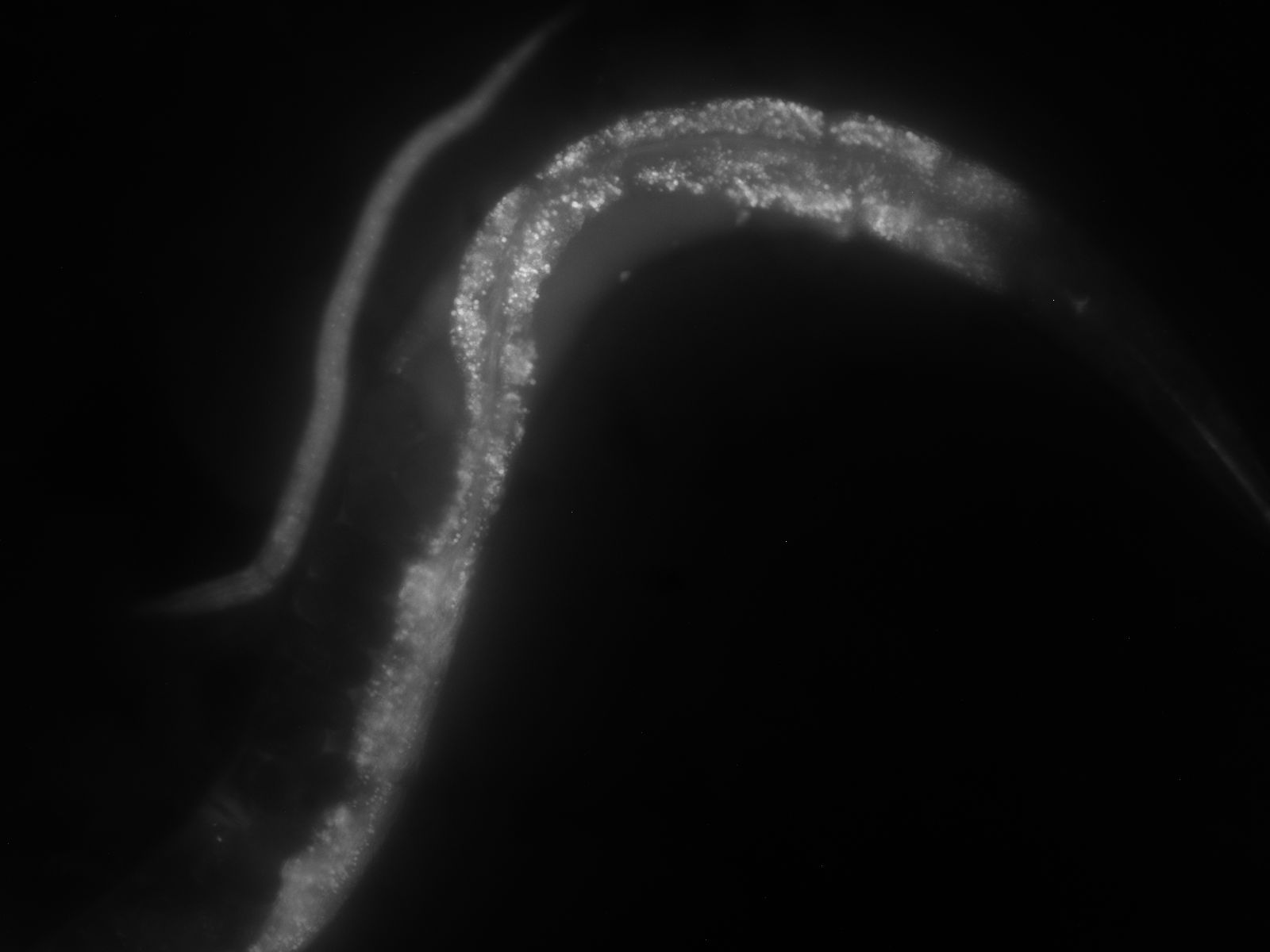

Supplement: S2 File — (ZIP) [file pgen.1011061.s002.zip › Fig.2A - Original files/Fig.2A RAW data and photos JPEG/syto12 staining - fig 2A - 2 rep_15.5.23 jpeg/n2+pad1264.jpg]

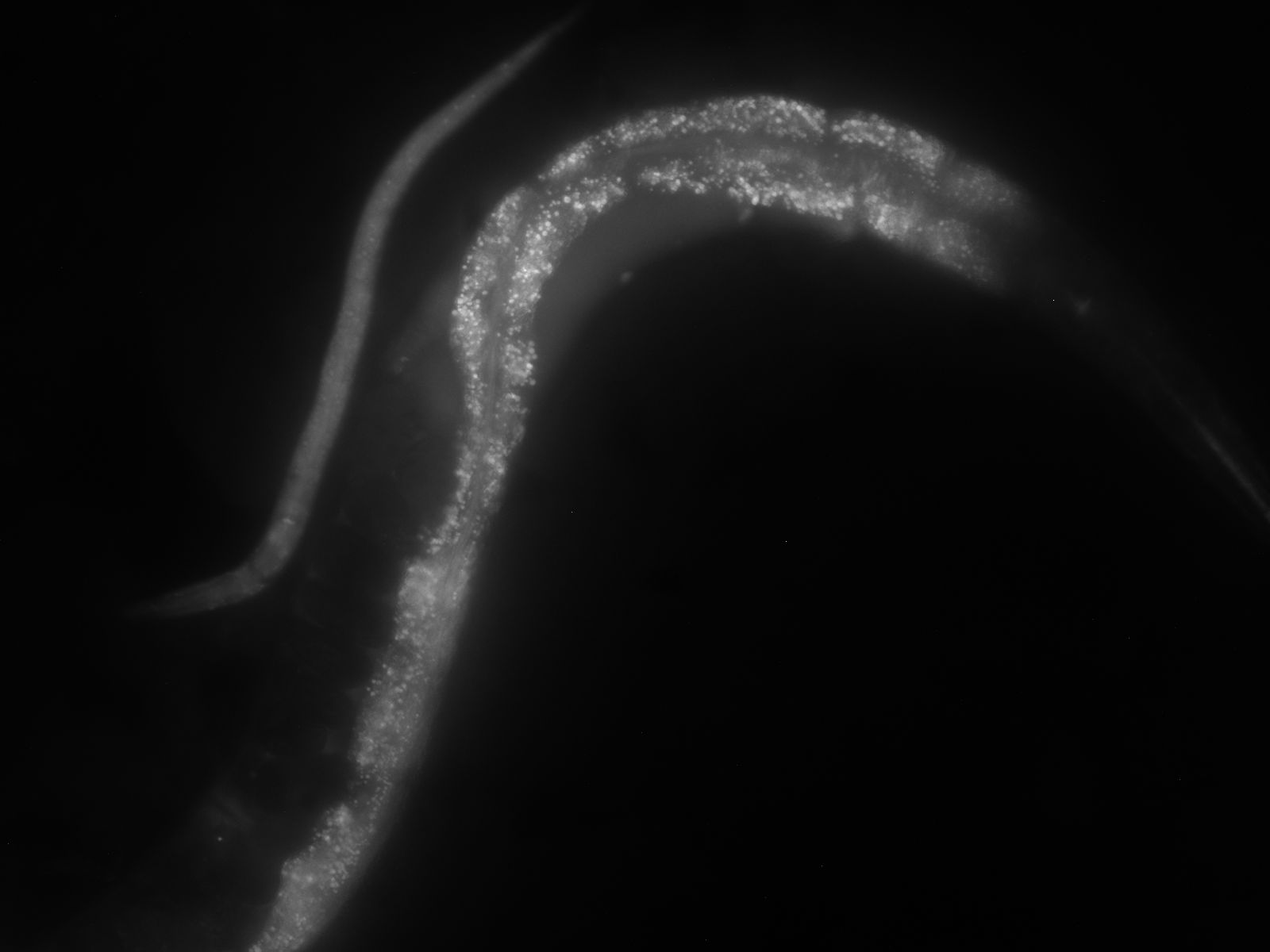

Supplement: S2 File — (ZIP) [file pgen.1011061.s002.zip › Fig.2A - Original files/Fig.2A RAW data and photos JPEG/syto12 staining - fig 2A - 2 rep_15.5.23 jpeg/n2+pad1265.jpg]

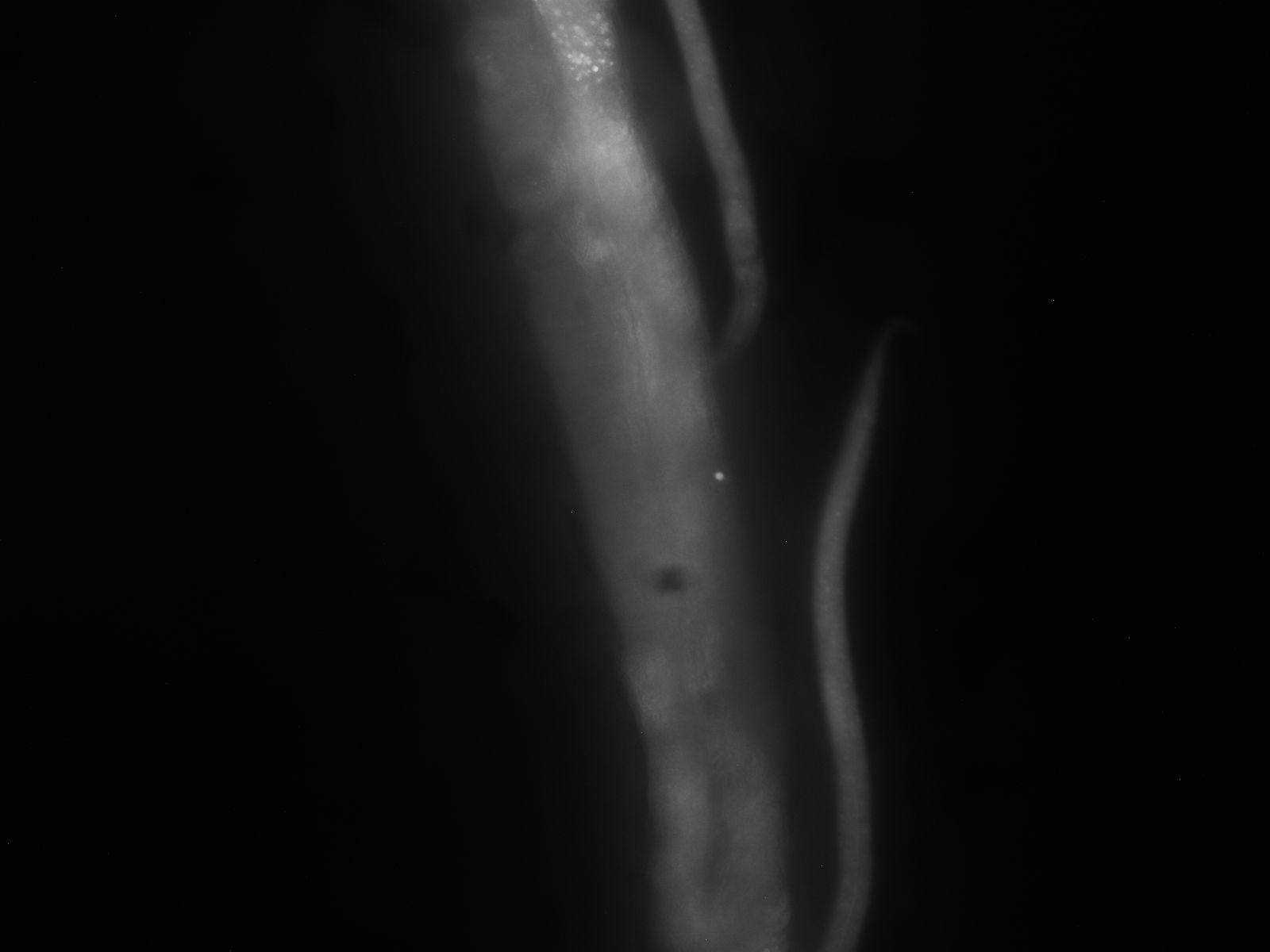

Supplement: S2 File — (ZIP) [file pgen.1011061.s002.zip › Fig.2A - Original files/Fig.2A RAW data and photos JPEG/syto12 staining - fig 2A - 2 rep_15.5.23 jpeg/n2+pad1266.jpg]

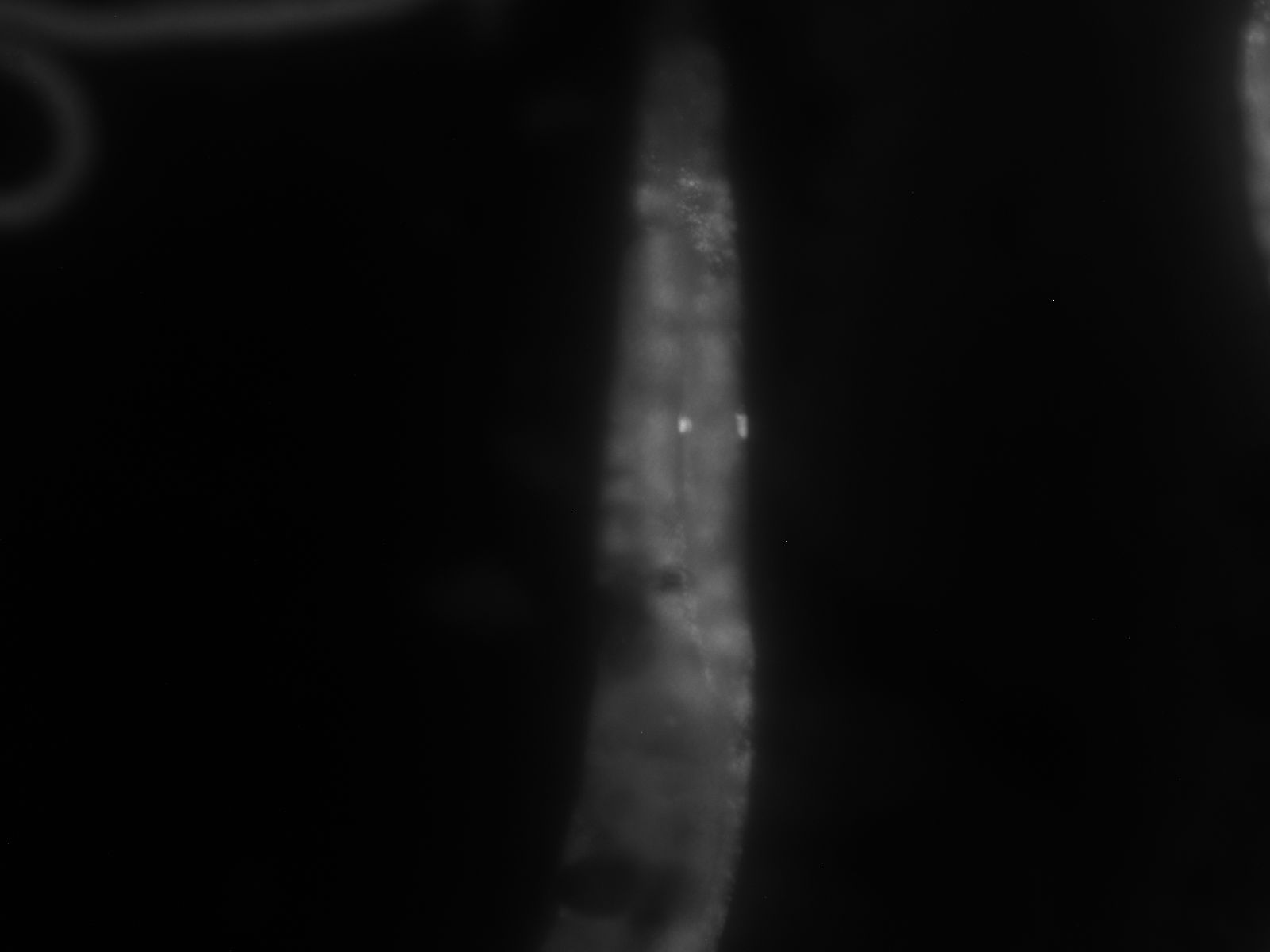

Supplement: S2 File — (ZIP) [file pgen.1011061.s002.zip › Fig.2A - Original files/Fig.2A RAW data and photos JPEG/syto12 staining - fig 2A - 2 rep_15.5.23 jpeg/n2+pad1267.jpg]

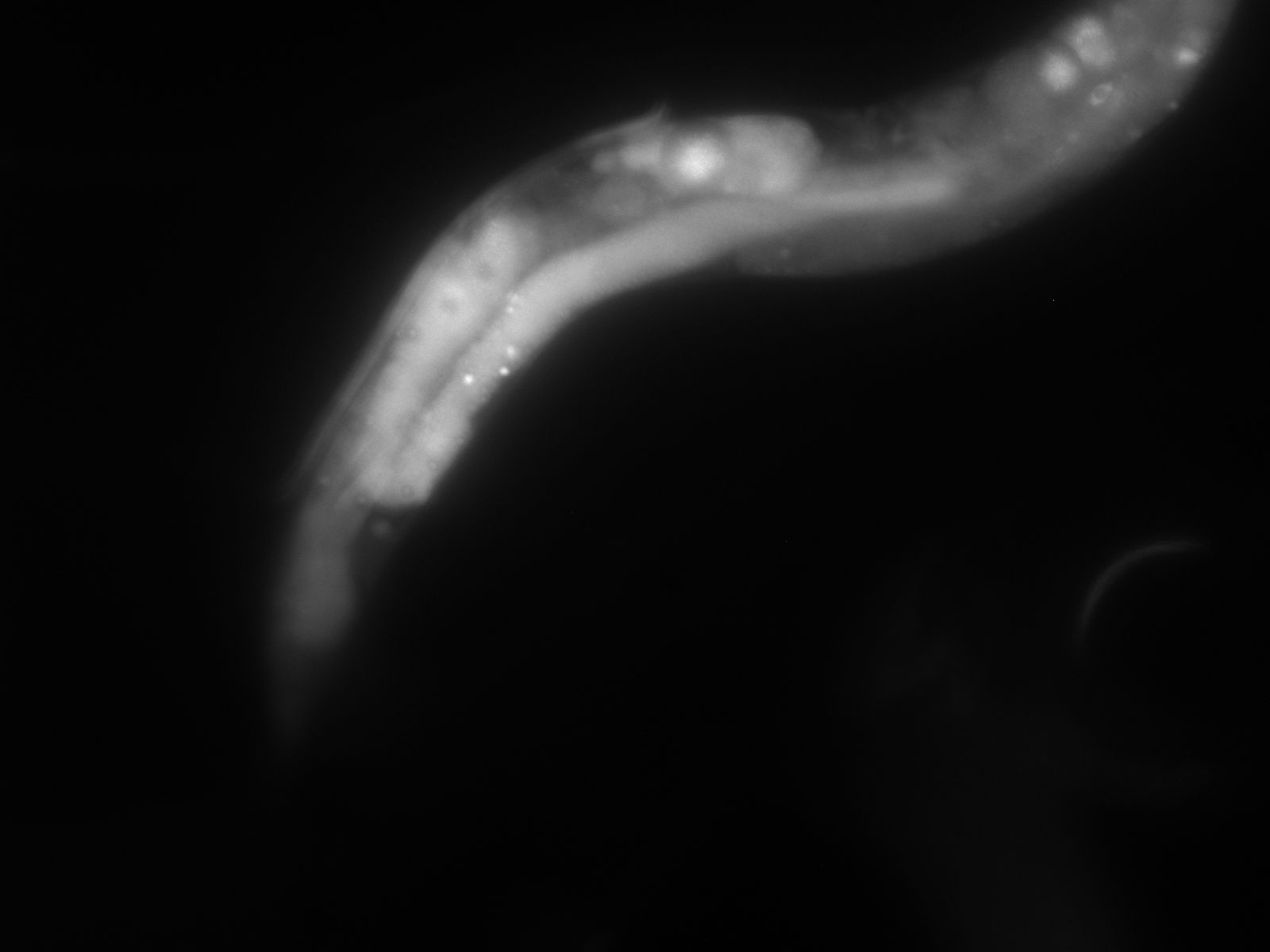

Supplement: S2 File — (ZIP) [file pgen.1011061.s002.zip › Fig.2A - Original files/Fig.2A RAW data and photos JPEG/syto12 staining - fig 2A - 2 rep_15.5.23 jpeg/n2+tfg158.jpg]

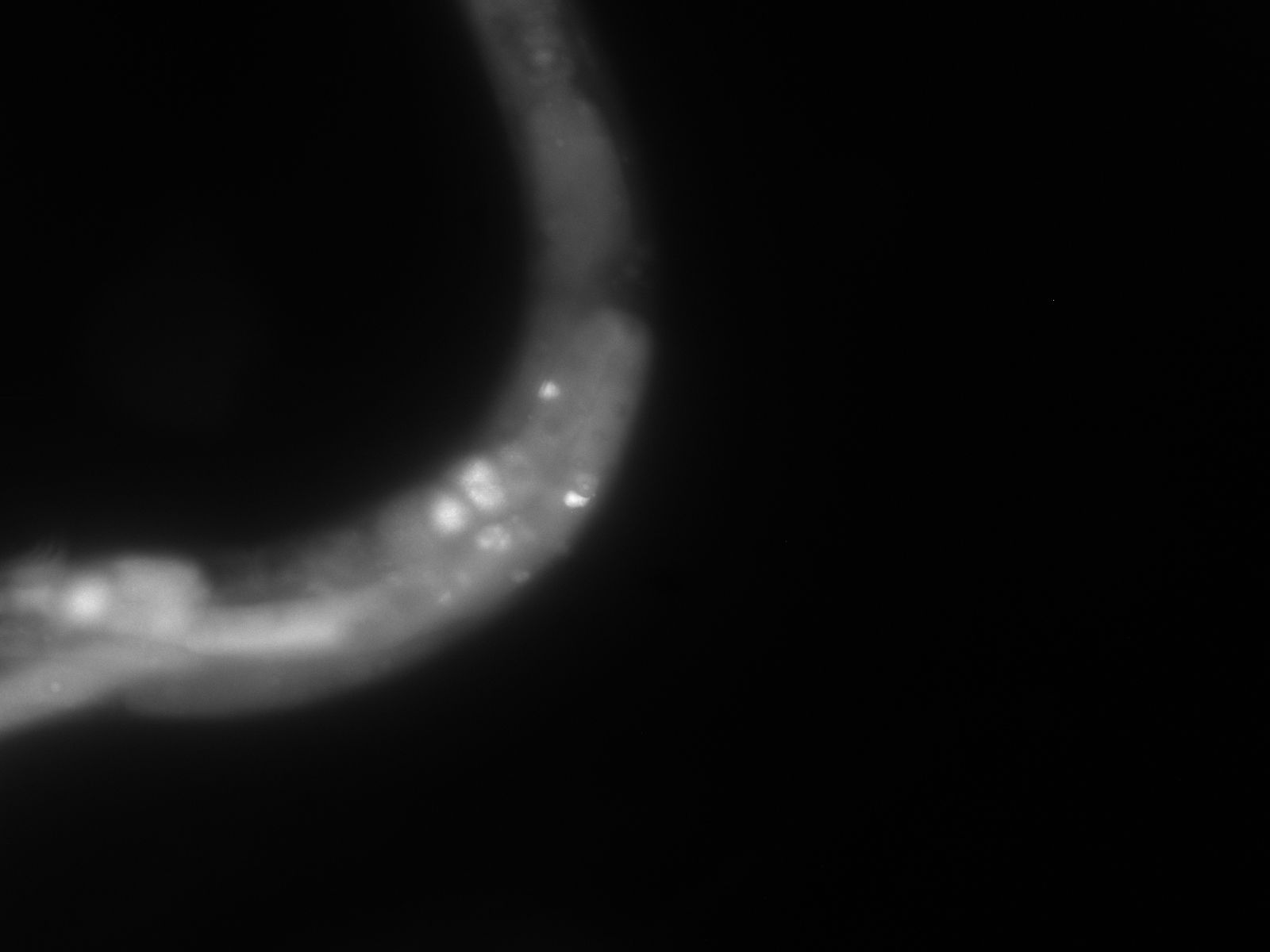

Supplement: S2 File — (ZIP) [file pgen.1011061.s002.zip › Fig.2A - Original files/Fig.2A RAW data and photos JPEG/syto12 staining - fig 2A - 2 rep_15.5.23 jpeg/n2+tfg159.jpg]

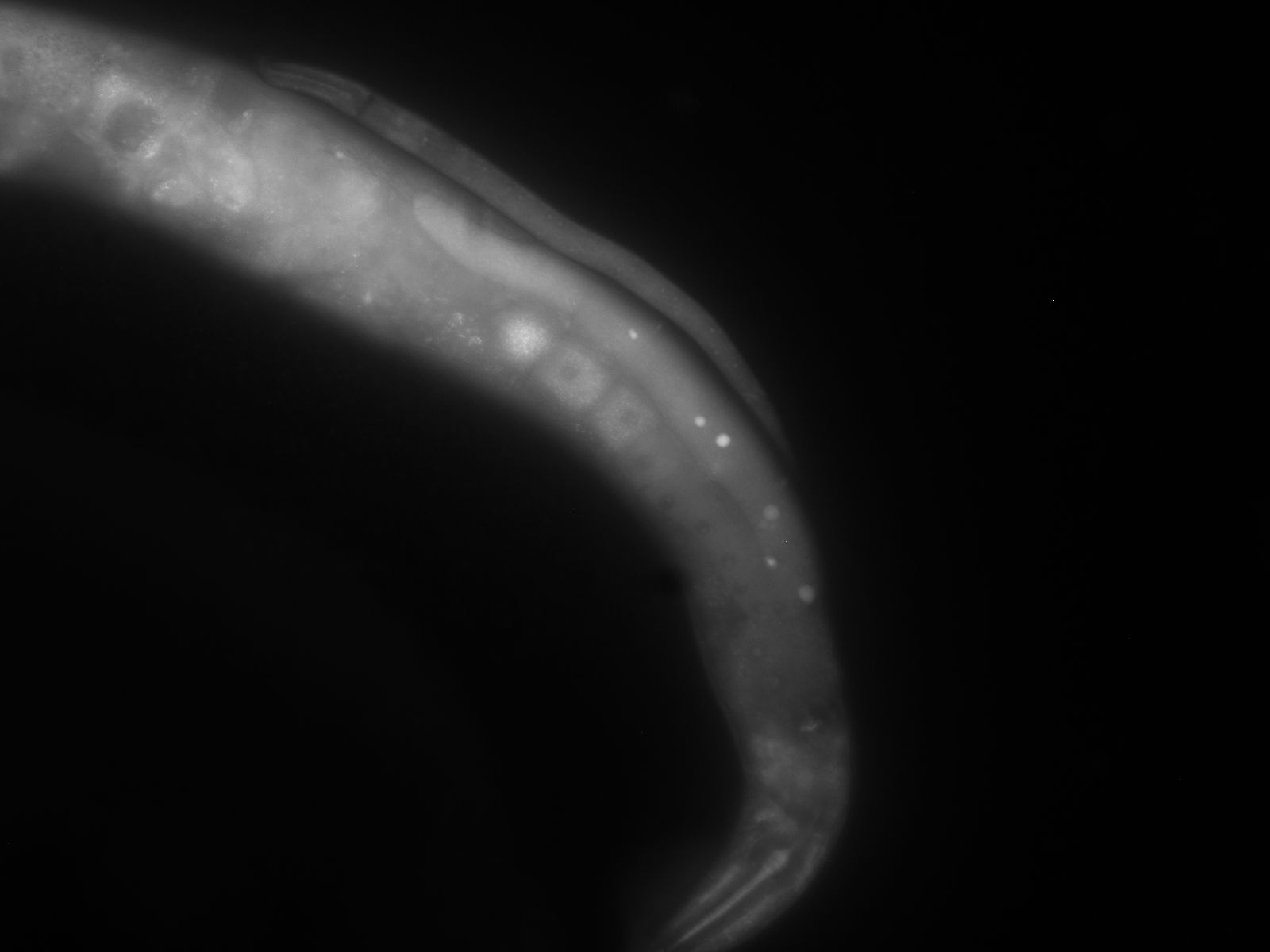

Supplement: S2 File — (ZIP) [file pgen.1011061.s002.zip › Fig.2A - Original files/Fig.2A RAW data and photos JPEG/syto12 staining - fig 2A - 2 rep_15.5.23 jpeg/n2+tfg160.jpg]

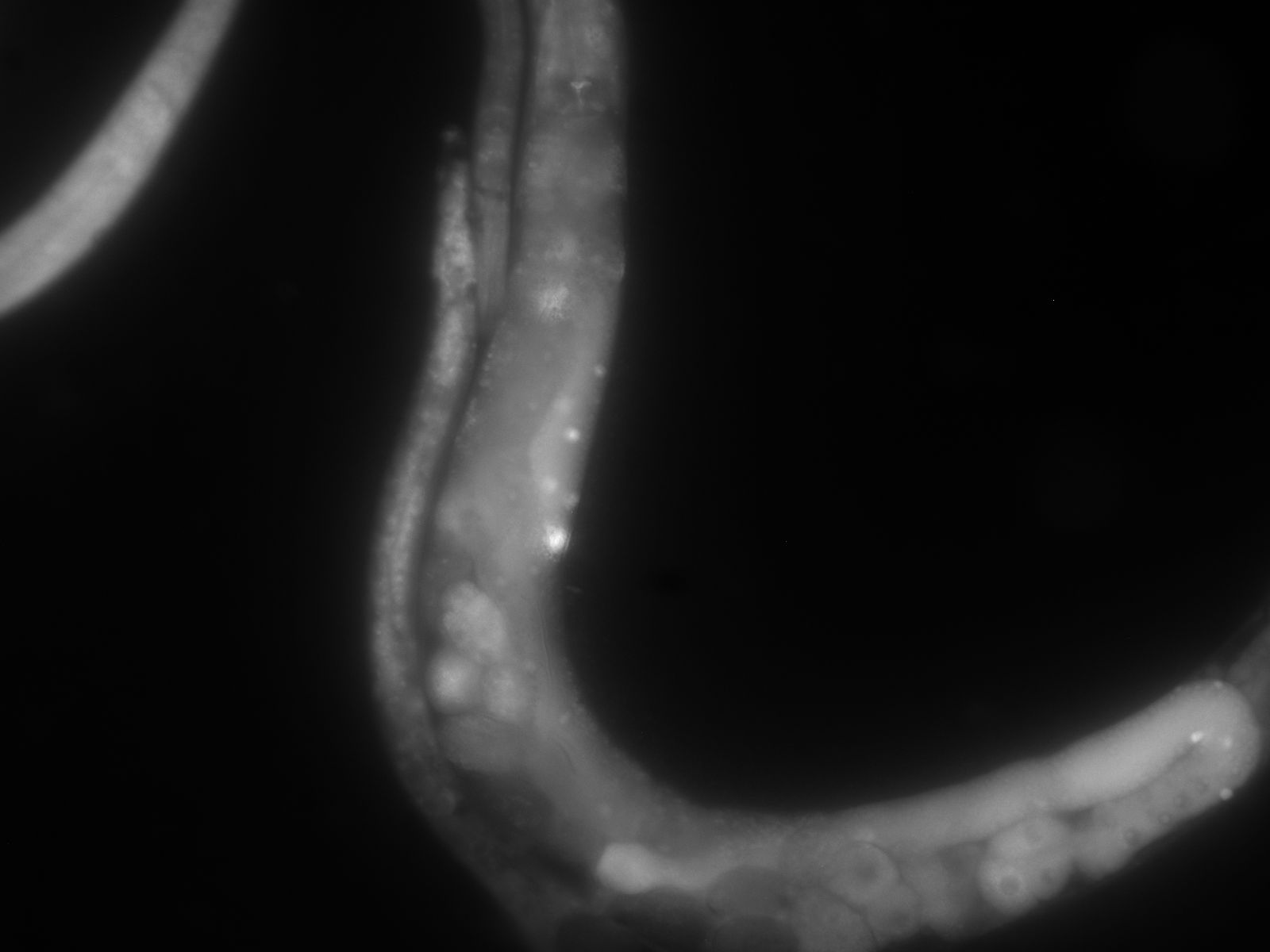

Supplement: S2 File — (ZIP) [file pgen.1011061.s002.zip › Fig.2A - Original files/Fig.2A RAW data and photos JPEG/syto12 staining - fig 2A - 2 rep_15.5.23 jpeg/n2+tfg161.jpg]

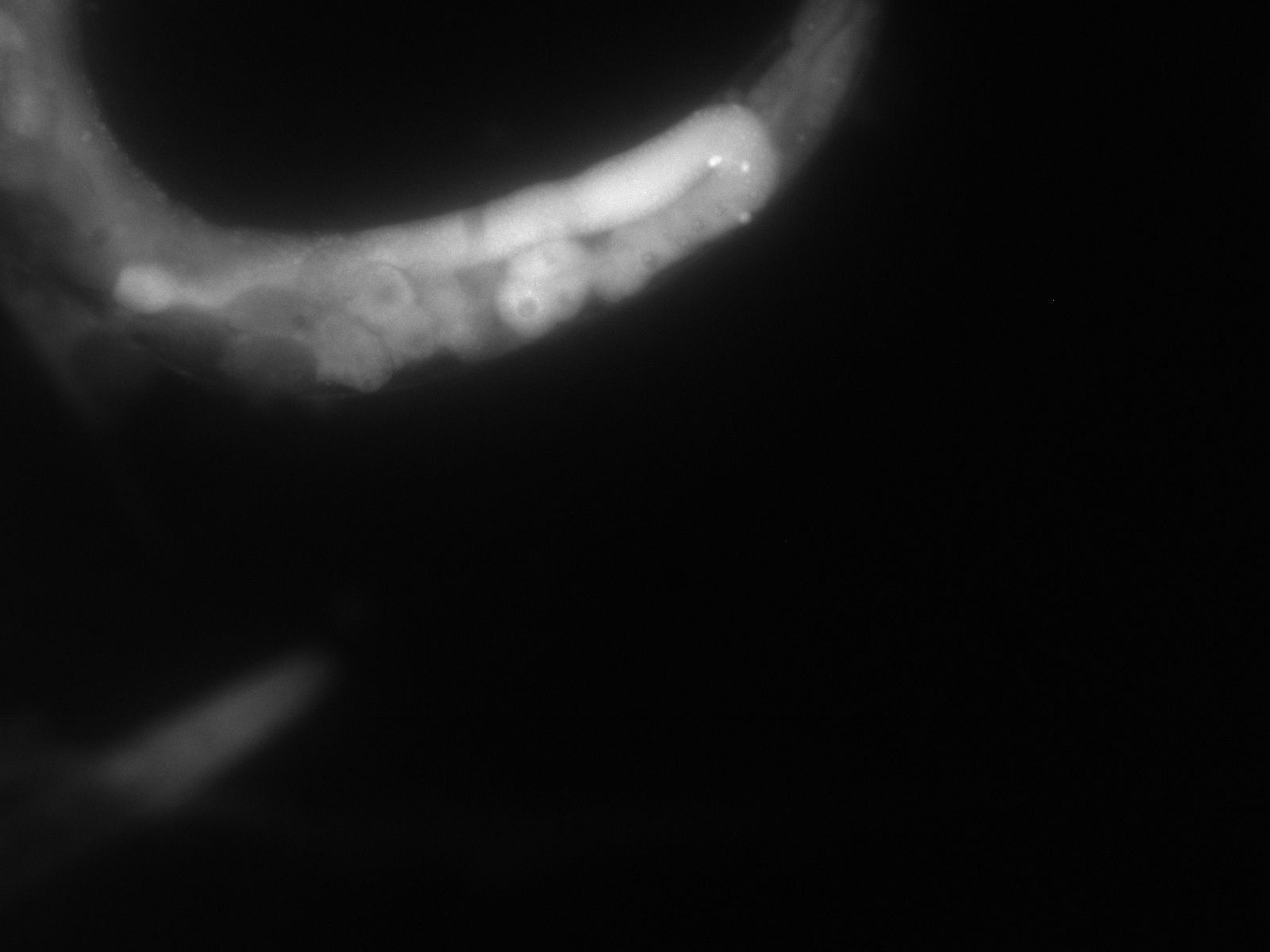

Supplement: S2 File — (ZIP) [file pgen.1011061.s002.zip › Fig.2A - Original files/Fig.2A RAW data and photos JPEG/syto12 staining - fig 2A - 2 rep_15.5.23 jpeg/n2+tfg162.jpg]

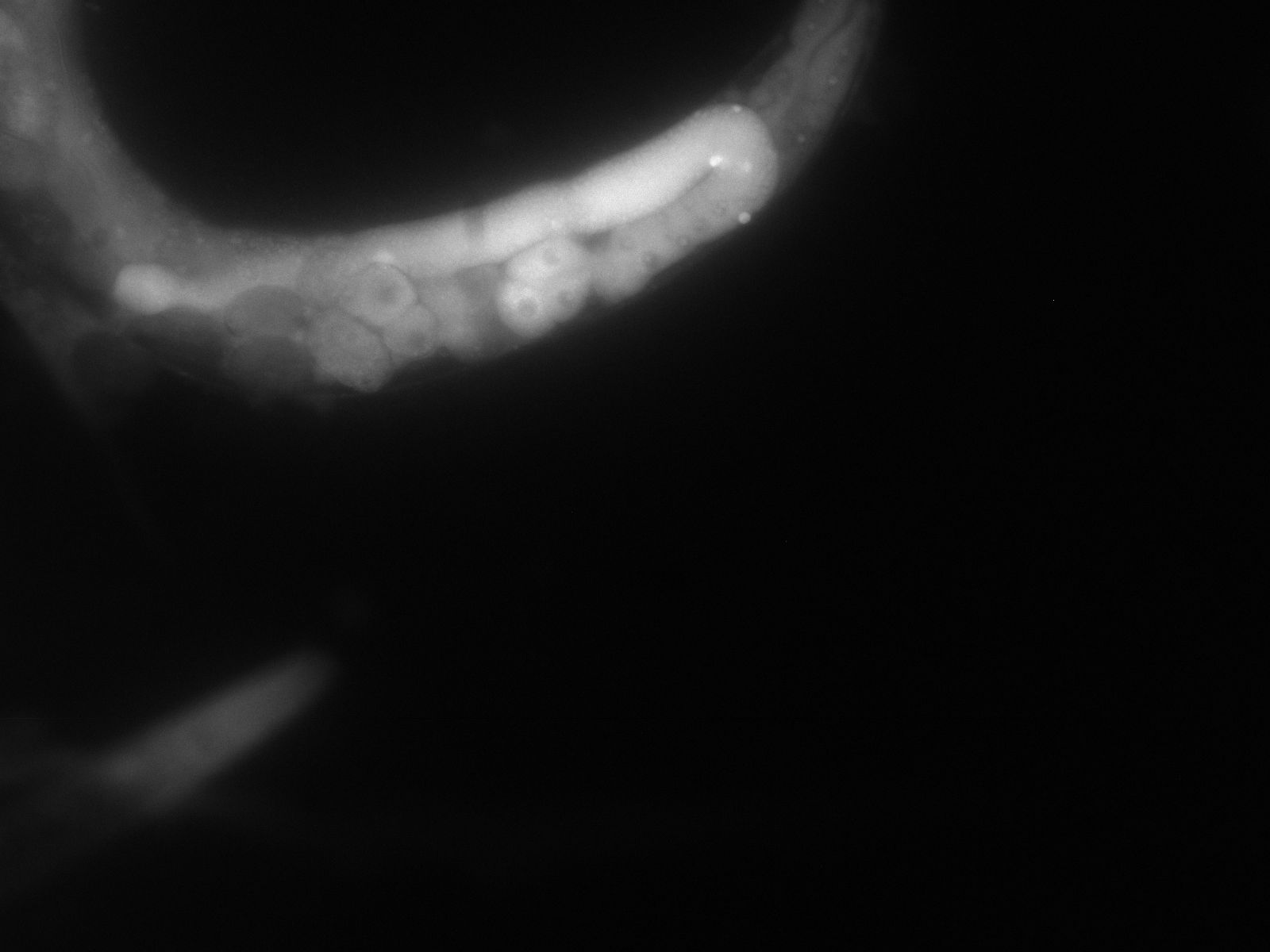

Supplement: S2 File — (ZIP) [file pgen.1011061.s002.zip › Fig.2A - Original files/Fig.2A RAW data and photos JPEG/syto12 staining - fig 2A - 2 rep_15.5.23 jpeg/n2+tfg163.jpg]

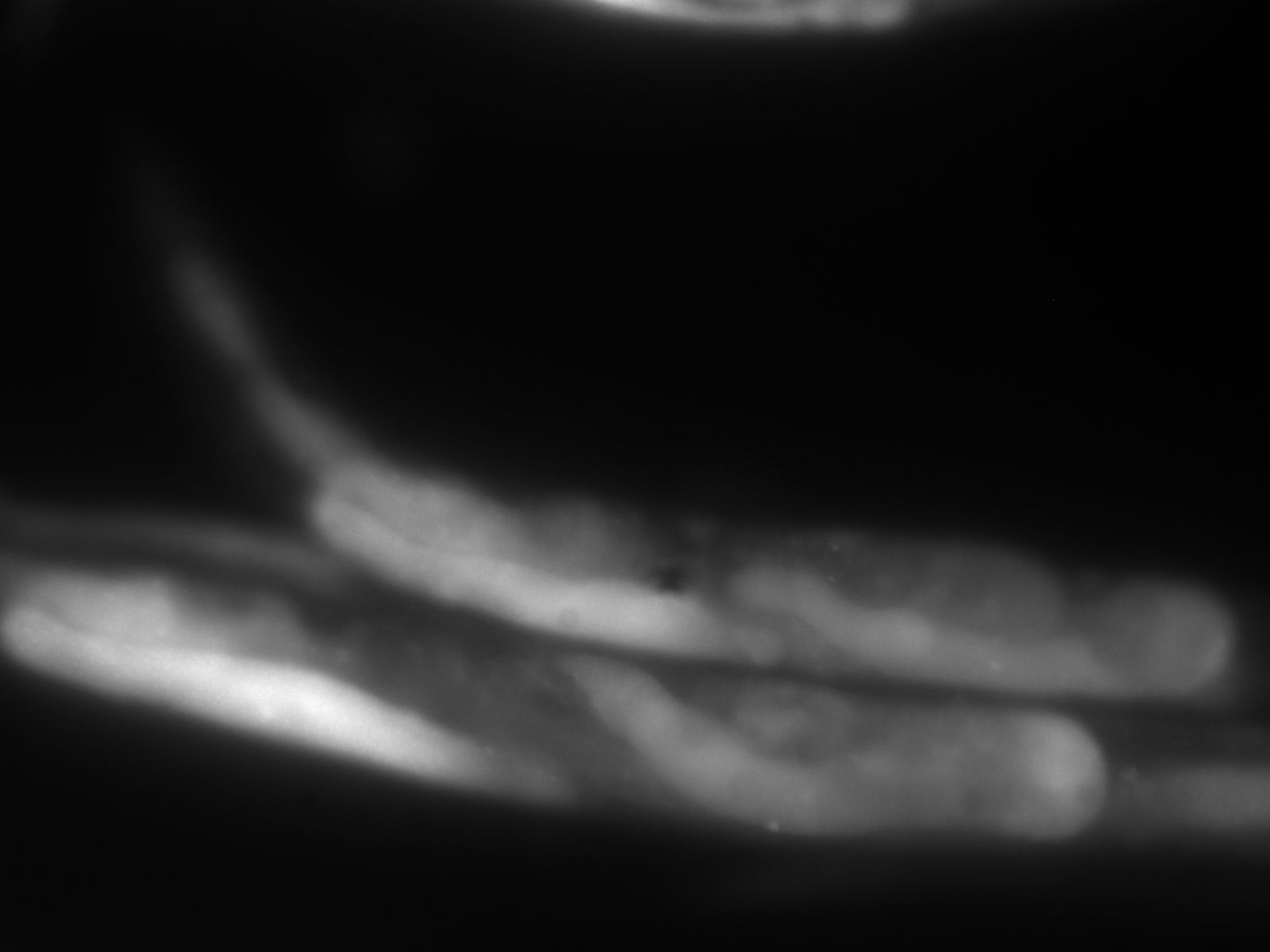

Supplement: S2 File — (ZIP) [file pgen.1011061.s002.zip › Fig.2A - Original files/Fig.2A RAW data and photos JPEG/syto12 staining - fig 2A - 2 rep_15.5.23 jpeg/xbp-1_ire-1+pad1207.jpg]

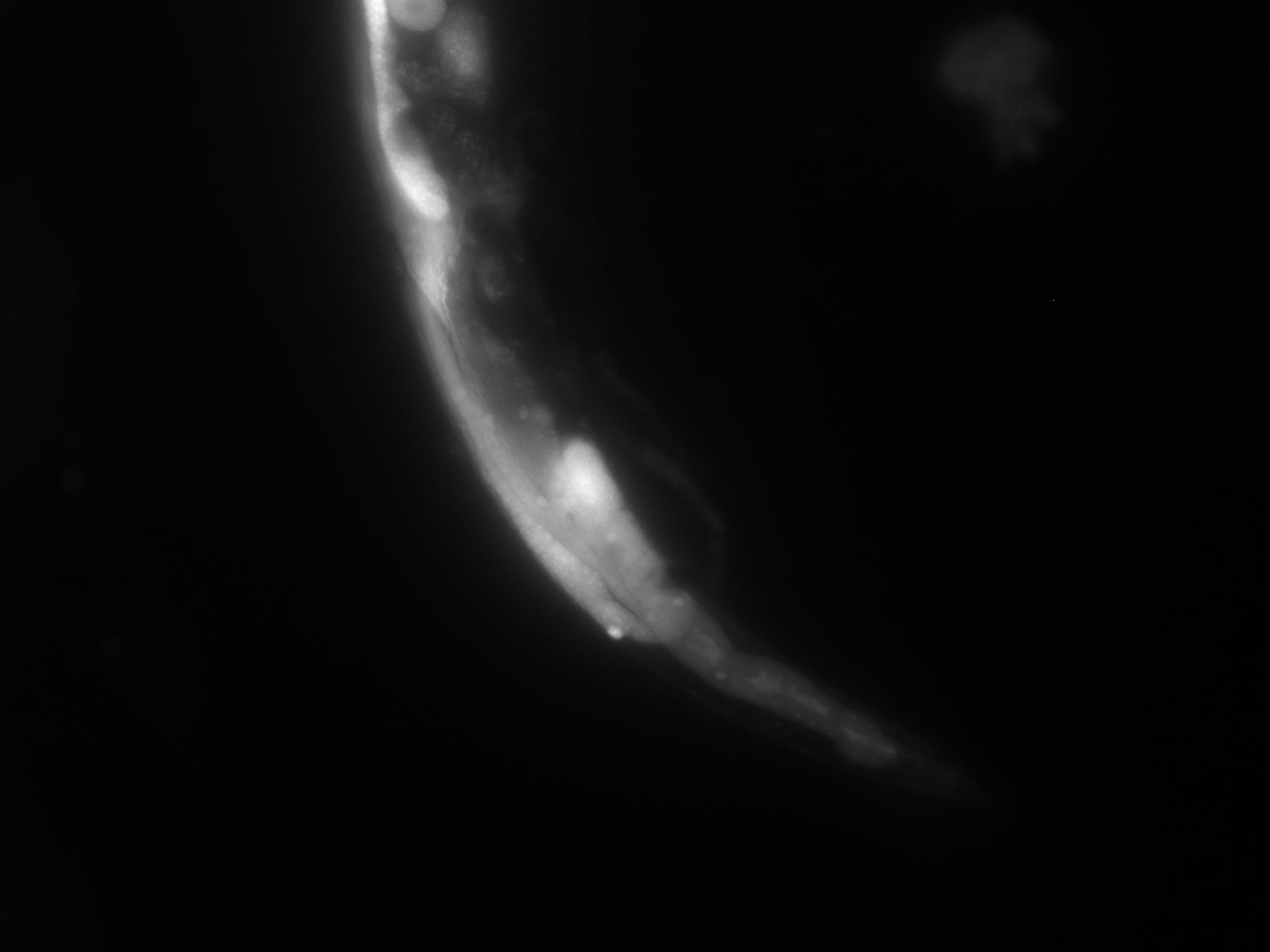

Supplement: S2 File — (ZIP) [file pgen.1011061.s002.zip › Fig.2A - Original files/Fig.2A RAW data and photos JPEG/syto12 staining - fig 2A - 2 rep_15.5.23 jpeg/xbp-1_ire-1+pad1208.jpg]

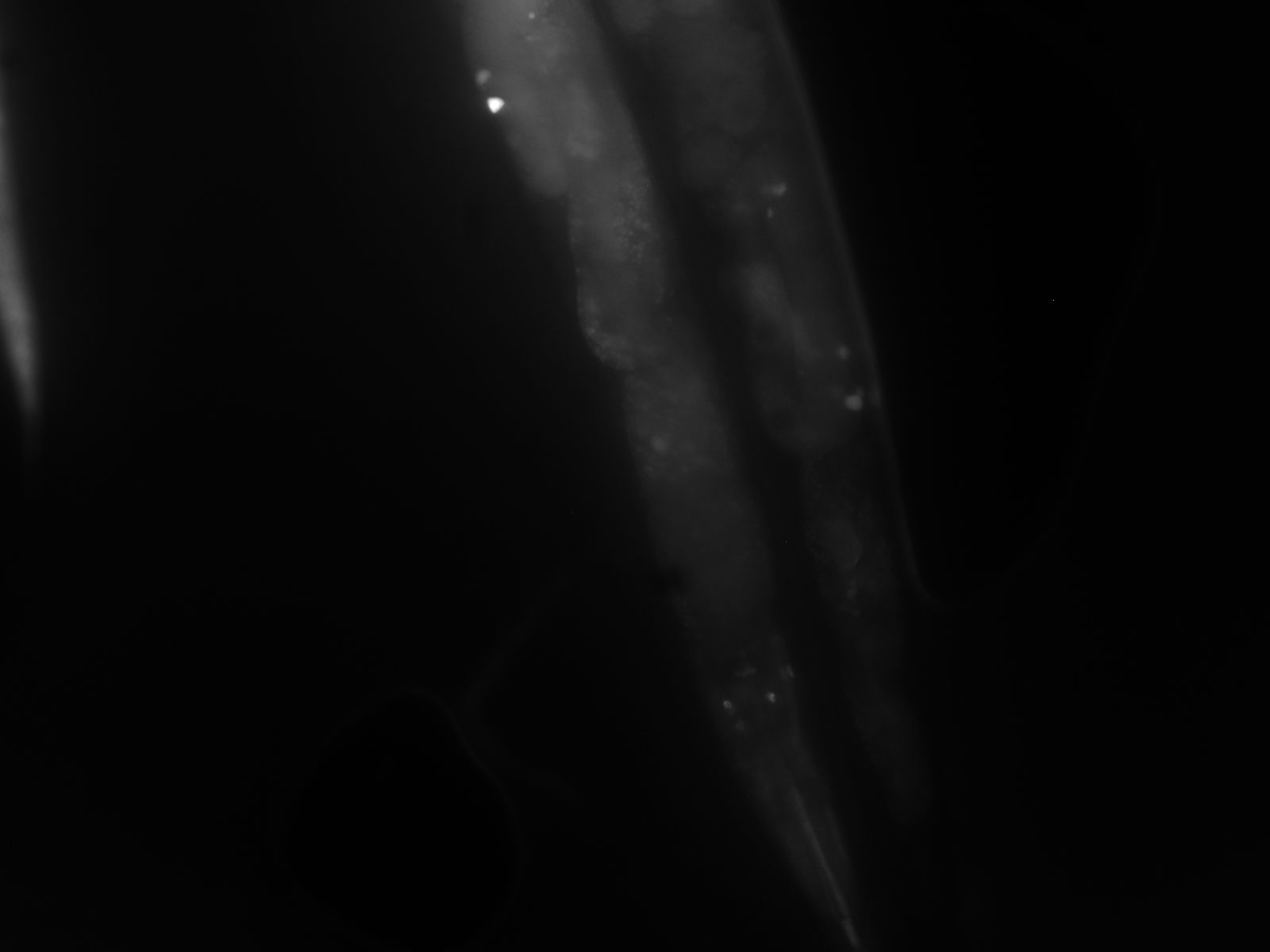

Supplement: S2 File — (ZIP) [file pgen.1011061.s002.zip › Fig.2A - Original files/Fig.2A RAW data and photos JPEG/syto12 staining - fig 2A - 2 rep_15.5.23 jpeg/xbp-1+pad1268.jpg]

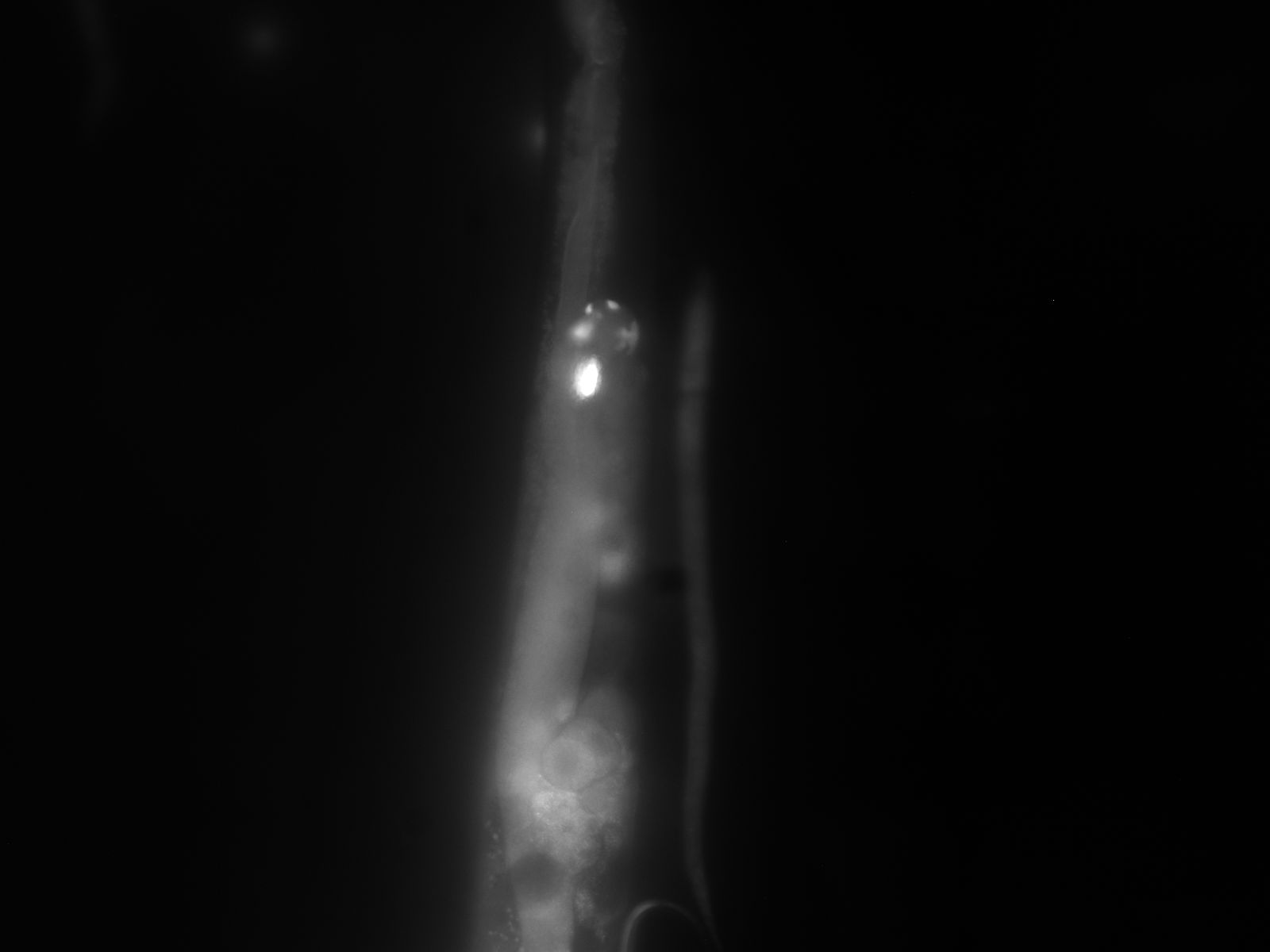

Supplement: S2 File — (ZIP) [file pgen.1011061.s002.zip › Fig.2A - Original files/Fig.2A RAW data and photos JPEG/syto12 staining - fig 2A - 2 rep_15.5.23 jpeg/xbp-1+pad1269.jpg]

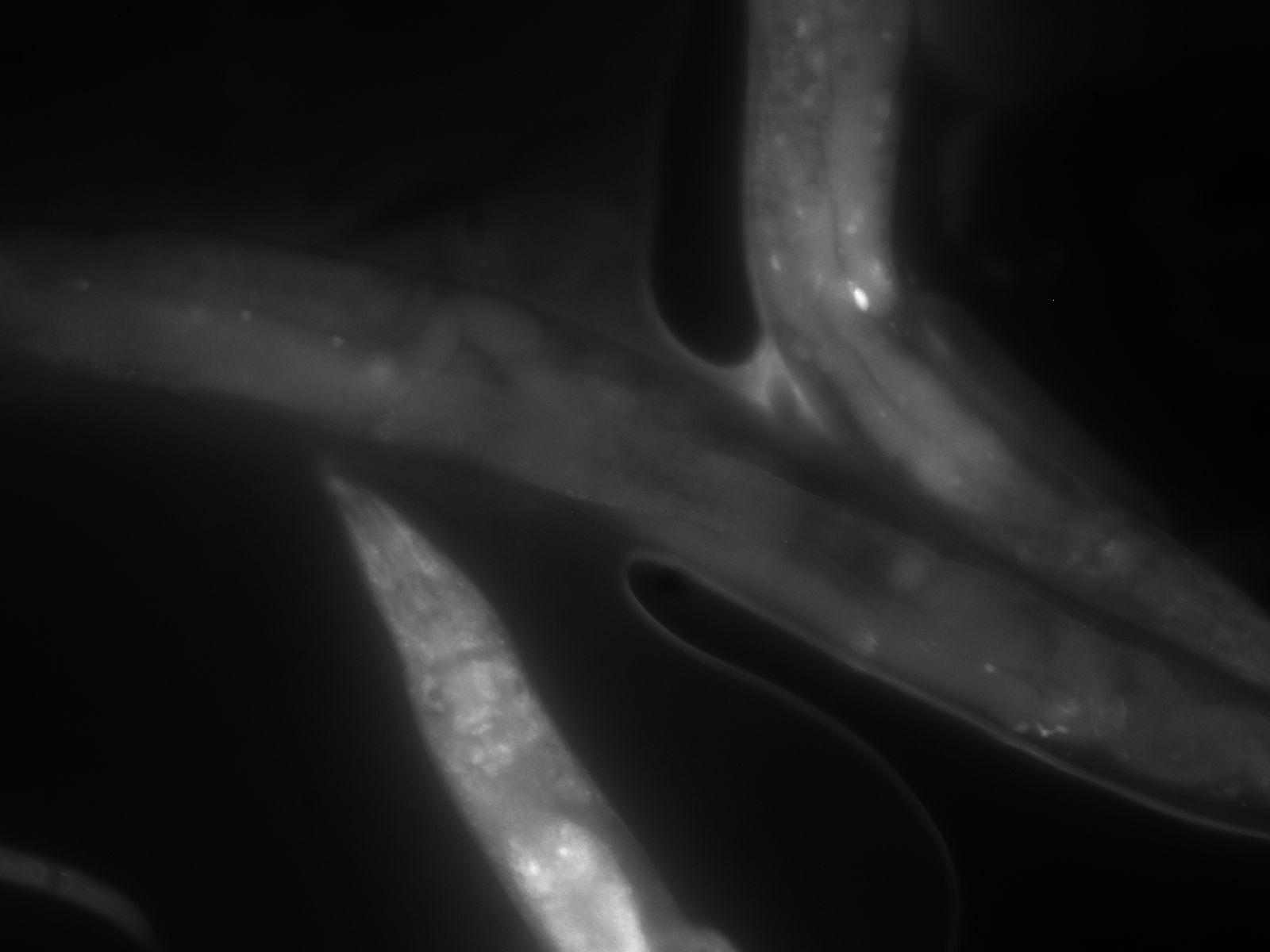

Supplement: S2 File — (ZIP) [file pgen.1011061.s002.zip › Fig.2A - Original files/Fig.2A RAW data and photos JPEG/syto12 staining - fig 2A - 2 rep_15.5.23 jpeg/xbp-1+pad1270.jpg]

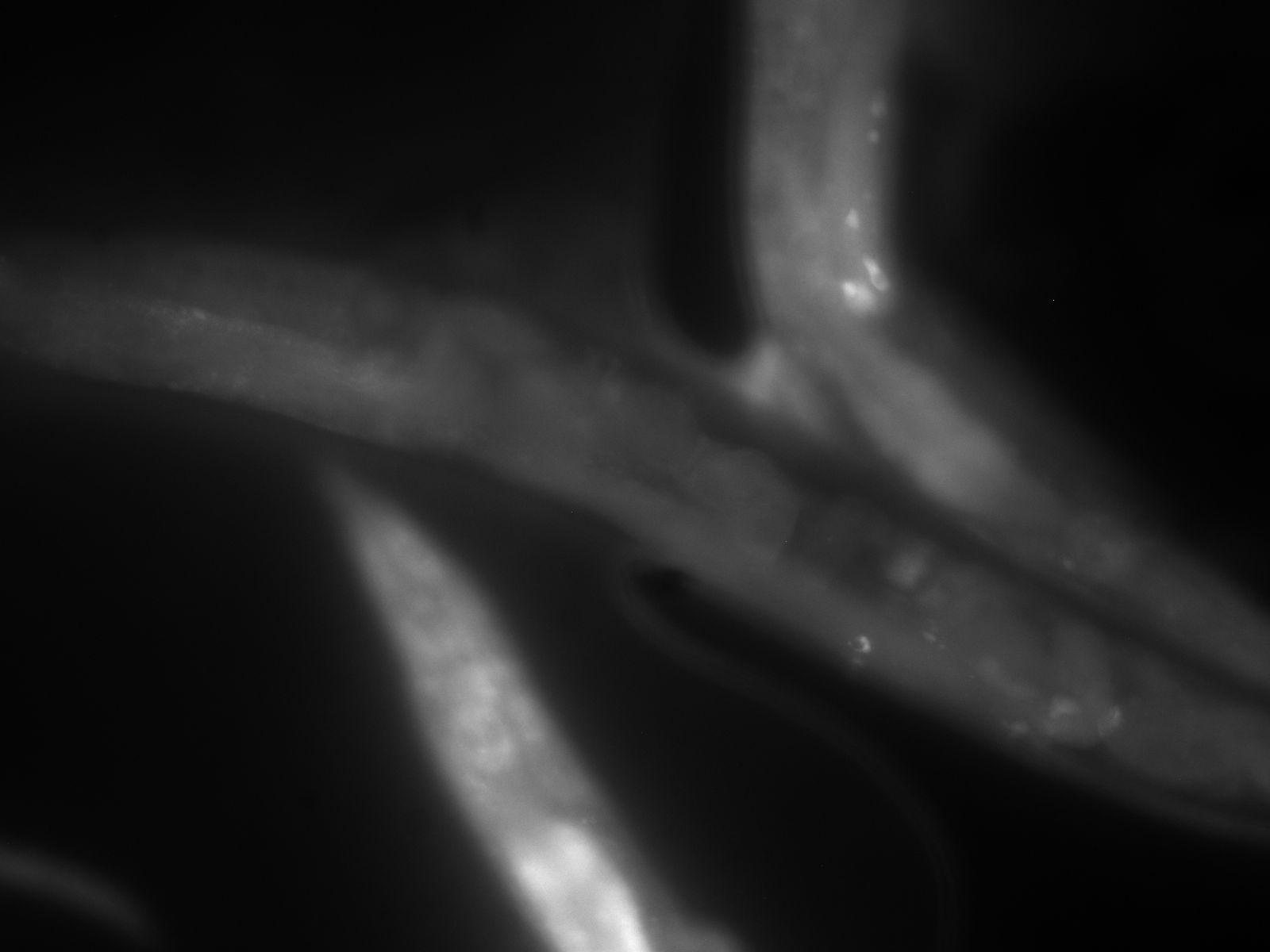

Supplement: S2 File — (ZIP) [file pgen.1011061.s002.zip › Fig.2A - Original files/Fig.2A RAW data and photos JPEG/syto12 staining - fig 2A - 2 rep_15.5.23 jpeg/xbp-1+pad1271.jpg]

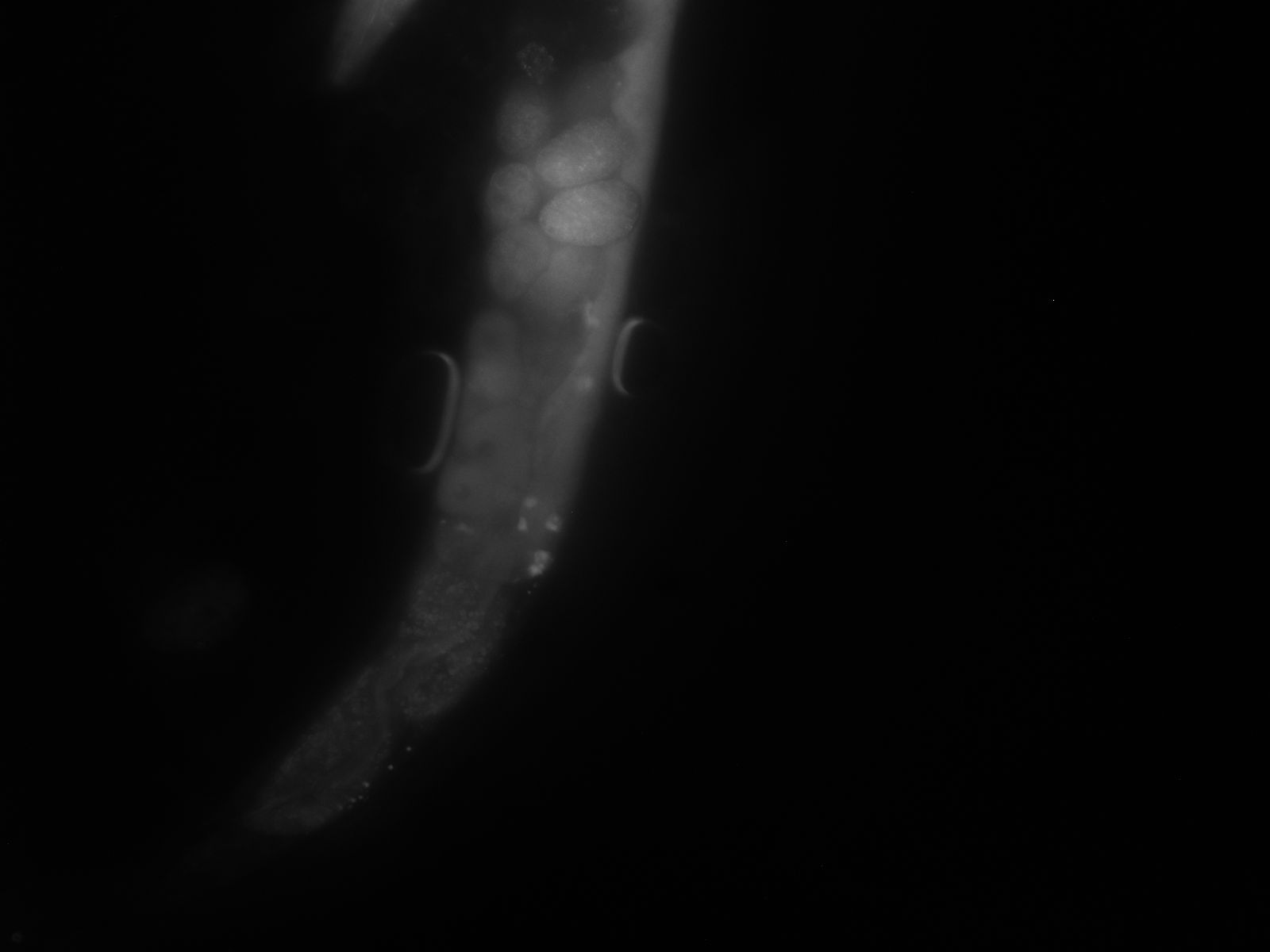

Supplement: S2 File — (ZIP) [file pgen.1011061.s002.zip › Fig.2A - Original files/Fig.2A RAW data and photos JPEG/syto12 staining - fig 2A - 2 rep_15.5.23 jpeg/xbp-1+pad1272.jpg]

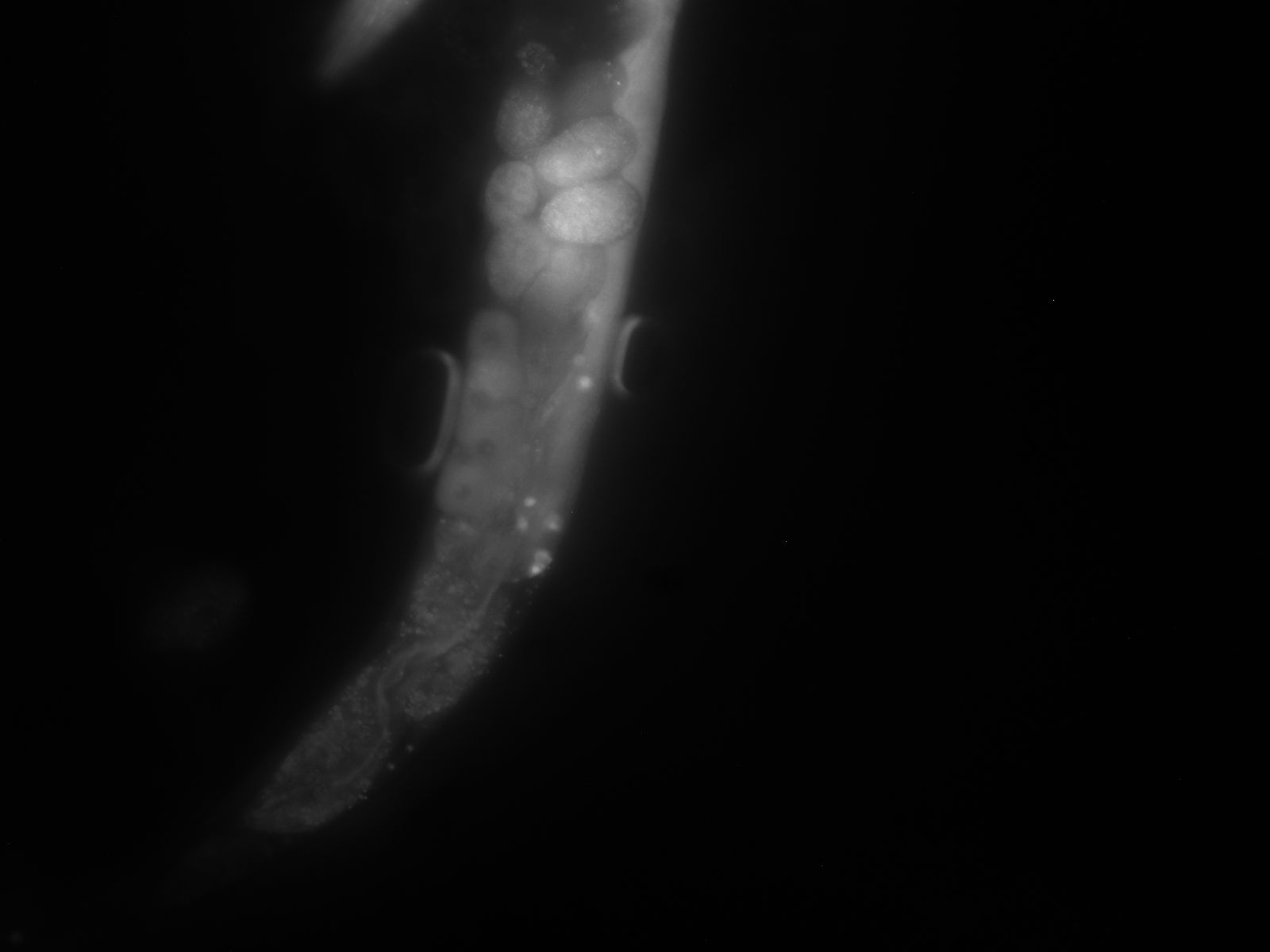

Supplement: S2 File — (ZIP) [file pgen.1011061.s002.zip › Fig.2A - Original files/Fig.2A RAW data and photos JPEG/syto12 staining - fig 2A - 2 rep_15.5.23 jpeg/xbp-1+pad1273.jpg]

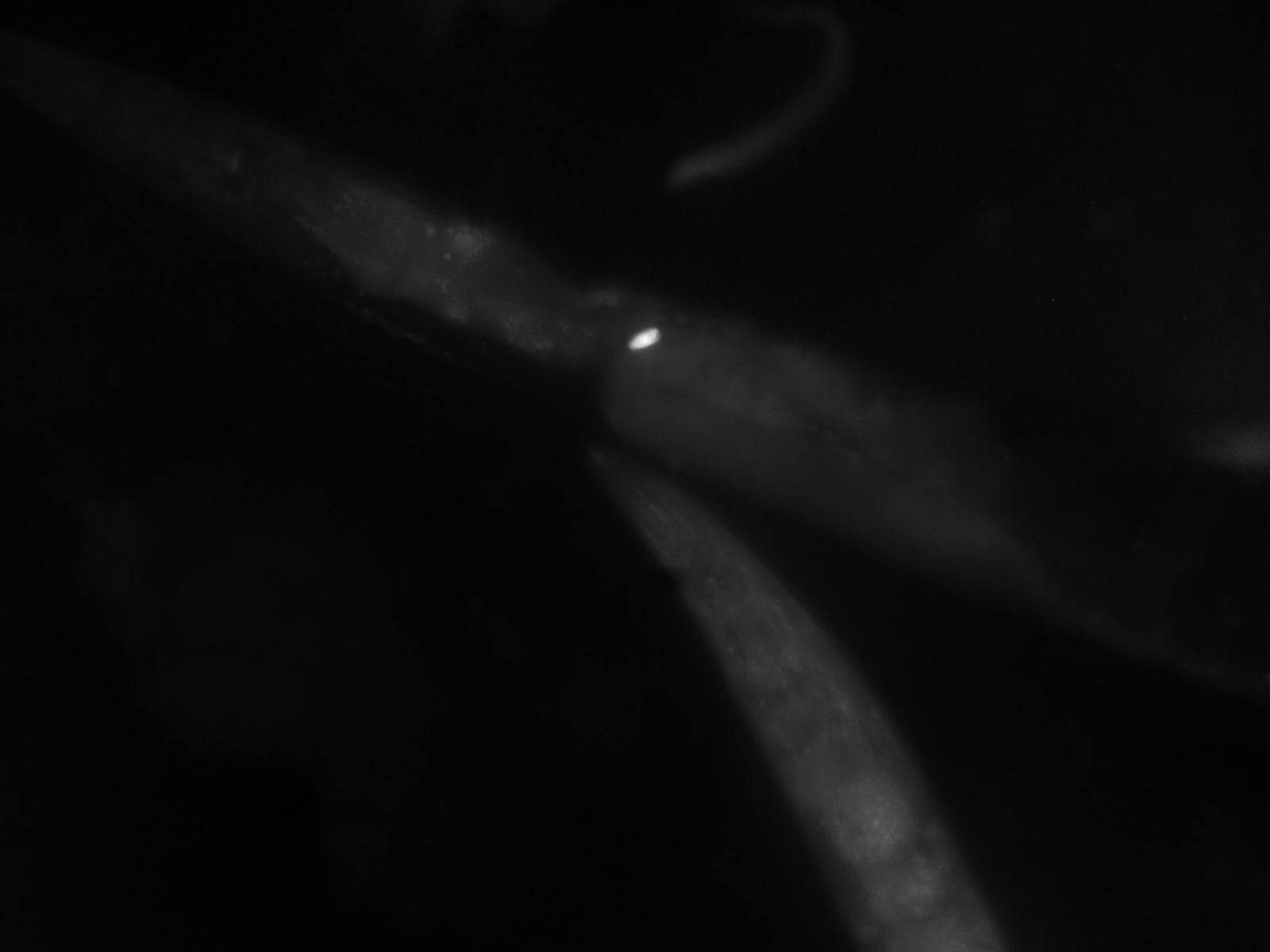

Supplement: S2 File — (ZIP) [file pgen.1011061.s002.zip › Fig.2A - Original files/Fig.2A RAW data and photos JPEG/syto12 staining - Fig 2A - 3_rep - 22.5.23 jpeg/ire-1_xbp-1+pad1206.jpg]

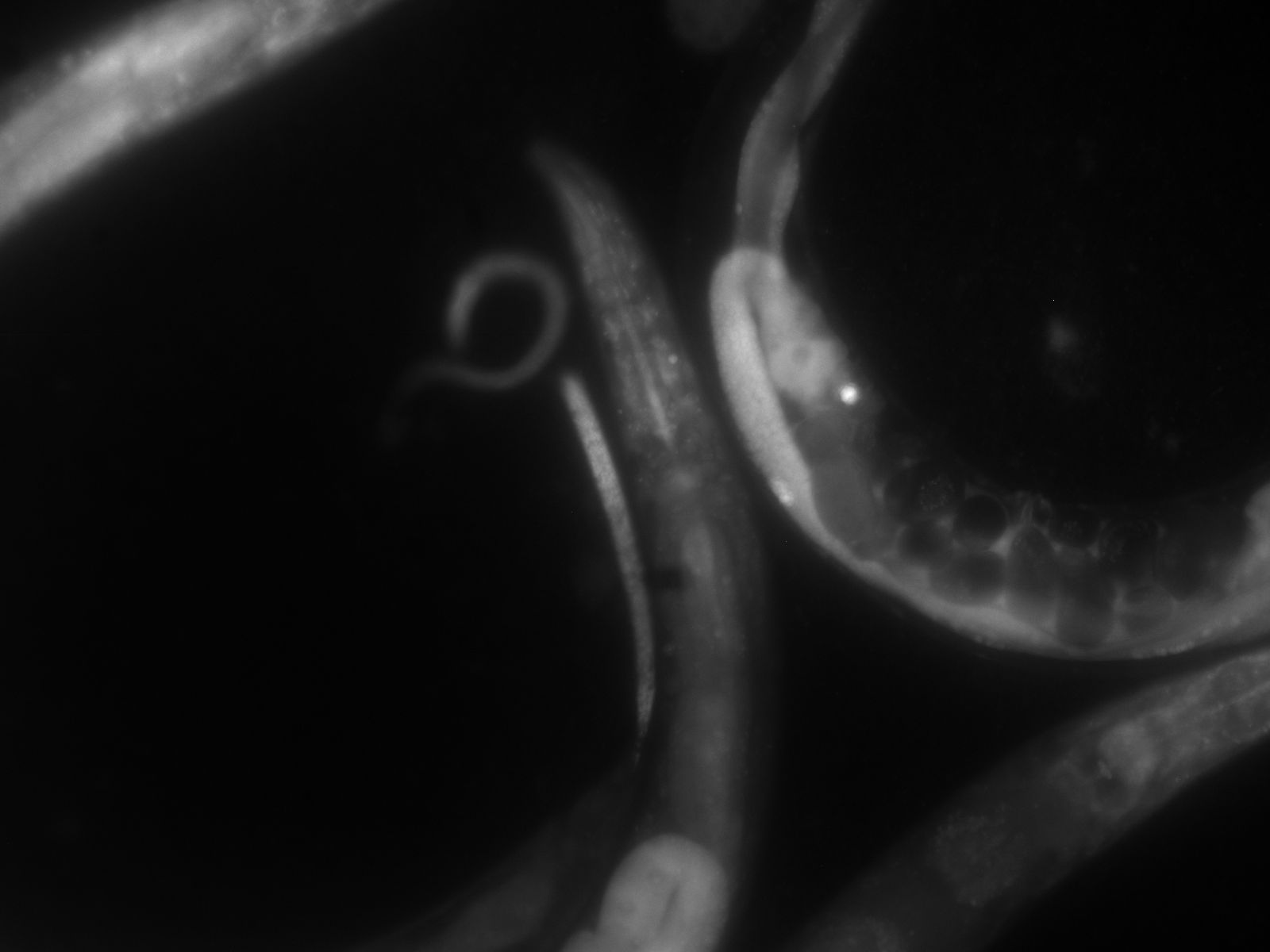

Supplement: S2 File — (ZIP) [file pgen.1011061.s002.zip › Fig.2A - Original files/Fig.2A RAW data and photos JPEG/syto12 staining - Fig 2A - 3_rep - 22.5.23 jpeg/ire-1_xbp-1+pad1207.jpg]

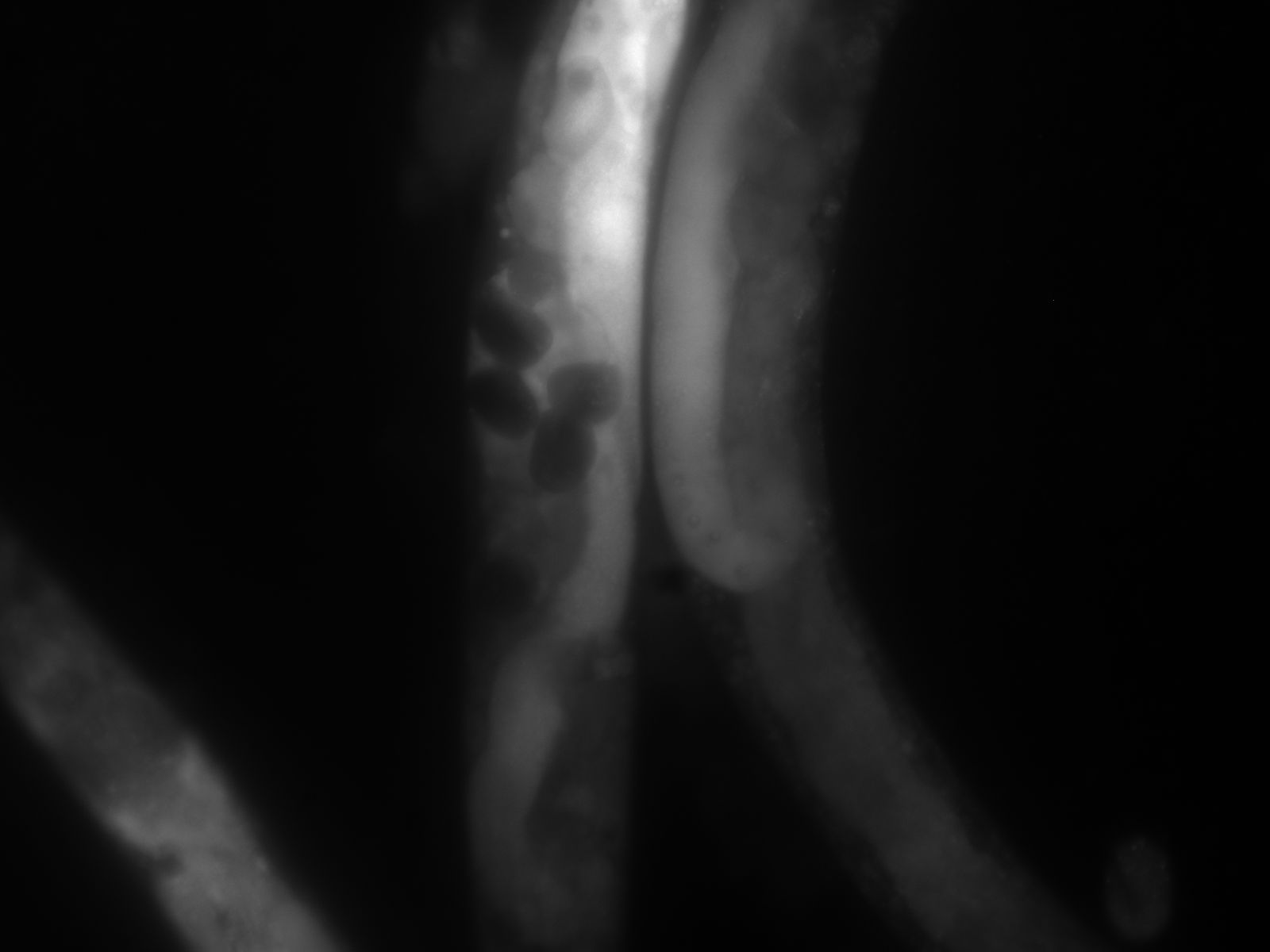

Supplement: S2 File — (ZIP) [file pgen.1011061.s002.zip › Fig.2A - Original files/Fig.2A RAW data and photos JPEG/syto12 staining - Fig 2A - 3_rep - 22.5.23 jpeg/ire-1_xbp-1+pad1208.jpg]

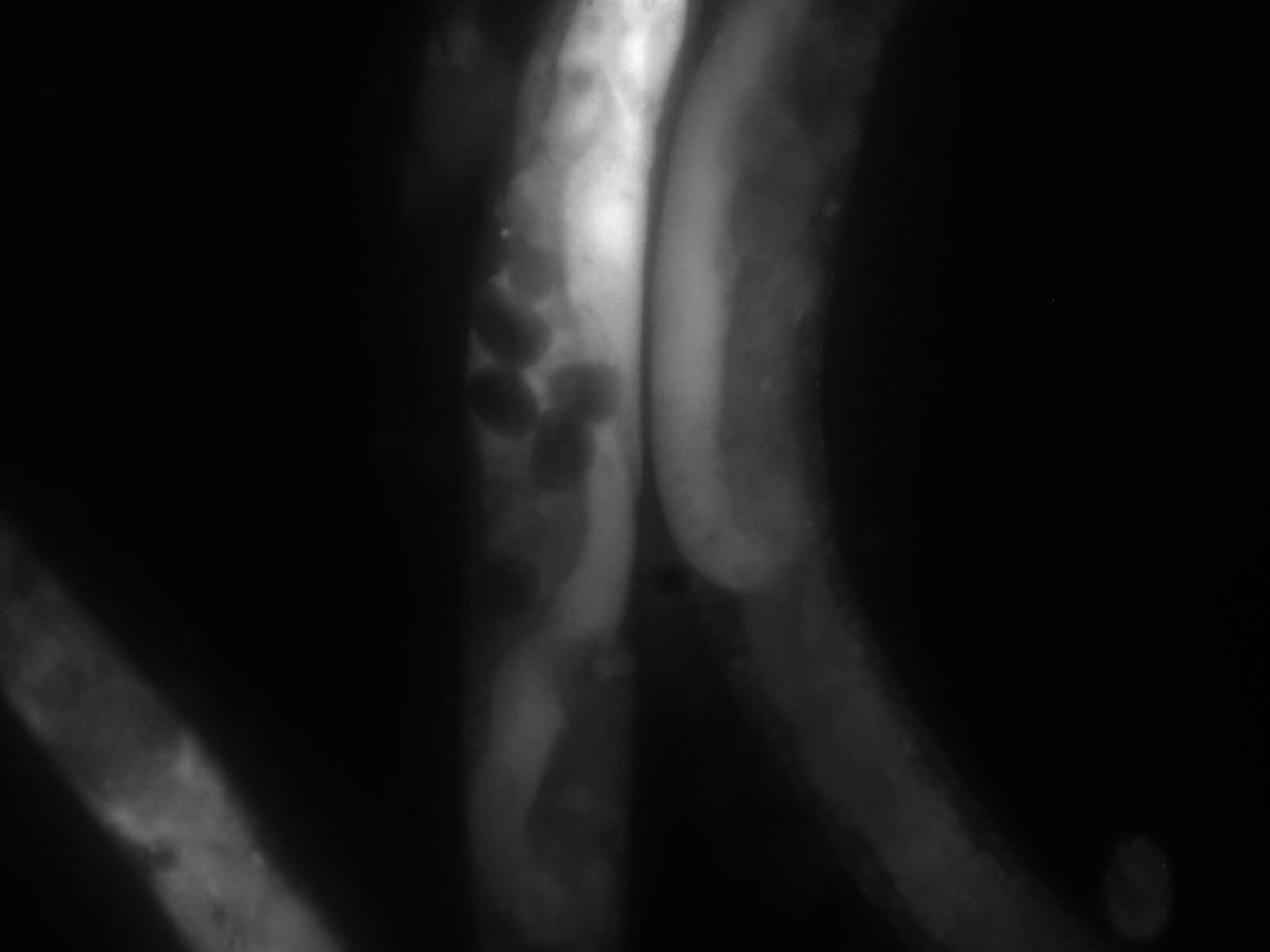

Supplement: S2 File — (ZIP) [file pgen.1011061.s002.zip › Fig.2A - Original files/Fig.2A RAW data and photos JPEG/syto12 staining - Fig 2A - 3_rep - 22.5.23 jpeg/ire-1_xbp-1+pad1209.jpg]

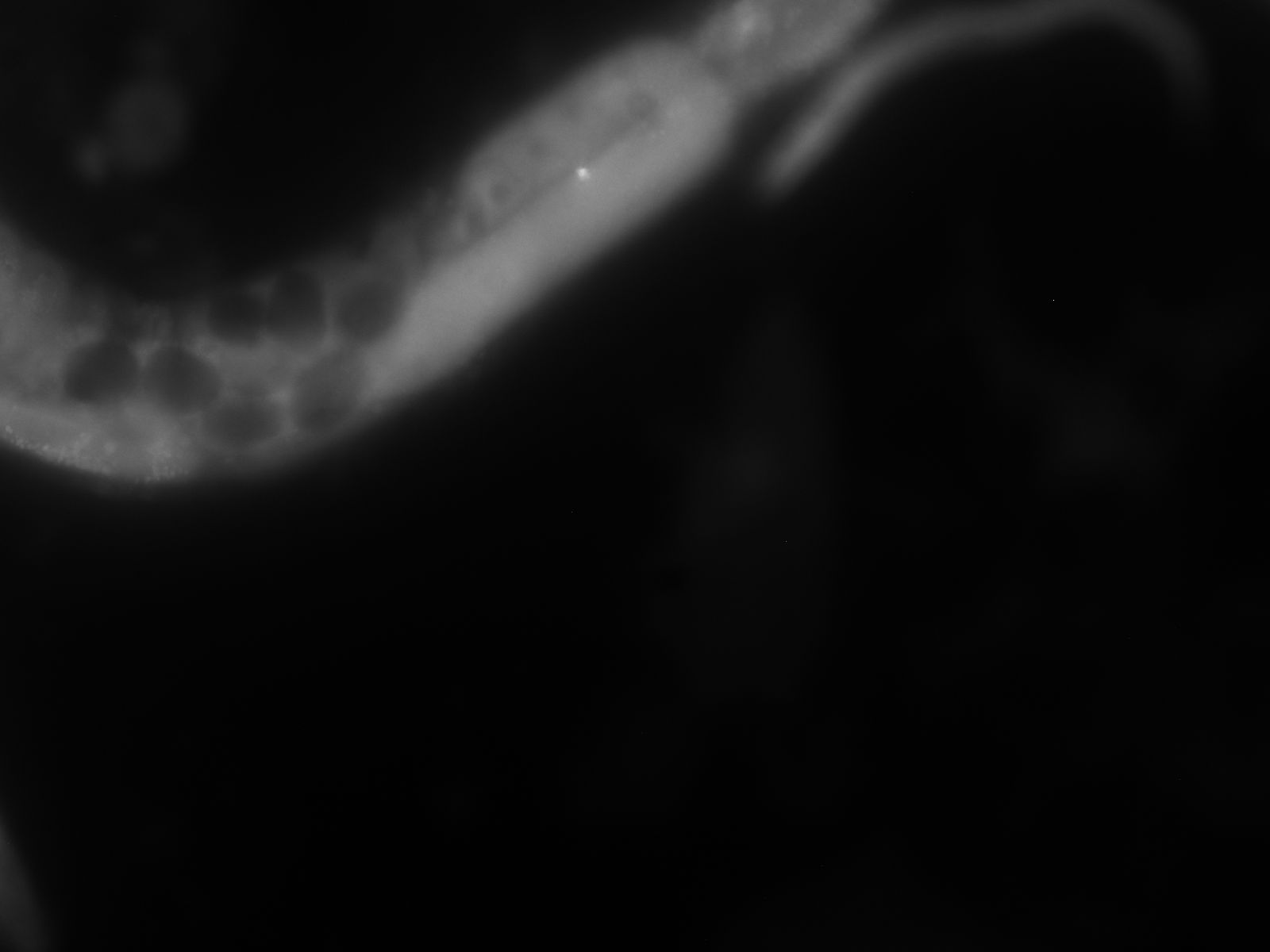

Supplement: S2 File — (ZIP) [file pgen.1011061.s002.zip › Fig.2A - Original files/Fig.2A RAW data and photos JPEG/syto12 staining - Fig 2A - 3_rep - 22.5.23 jpeg/ire-1+pad1204.jpg]

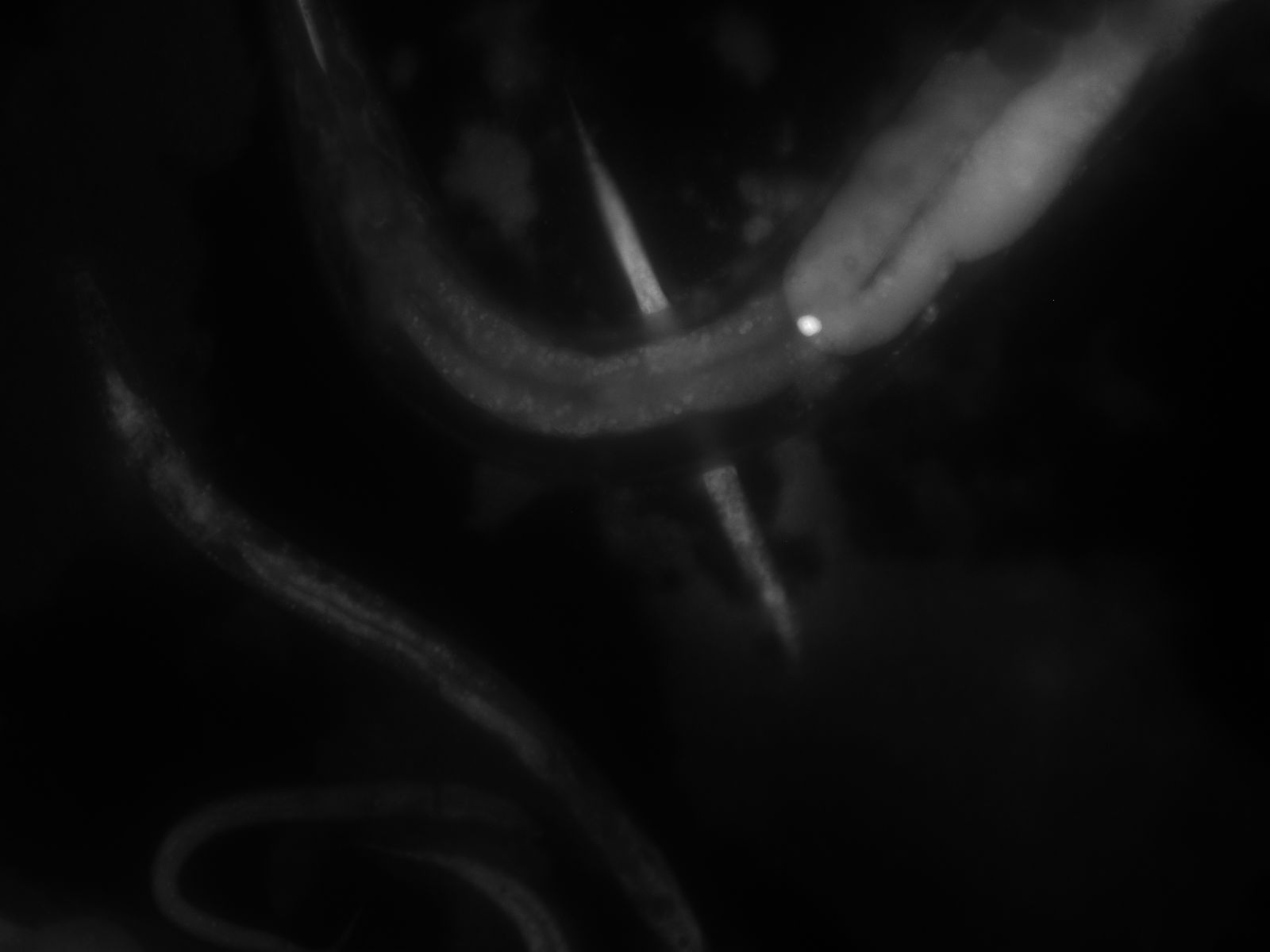

Supplement: S2 File — (ZIP) [file pgen.1011061.s002.zip › Fig.2A - Original files/Fig.2A RAW data and photos JPEG/syto12 staining - Fig 2A - 3_rep - 22.5.23 jpeg/ire-1+pad1205.jpg]

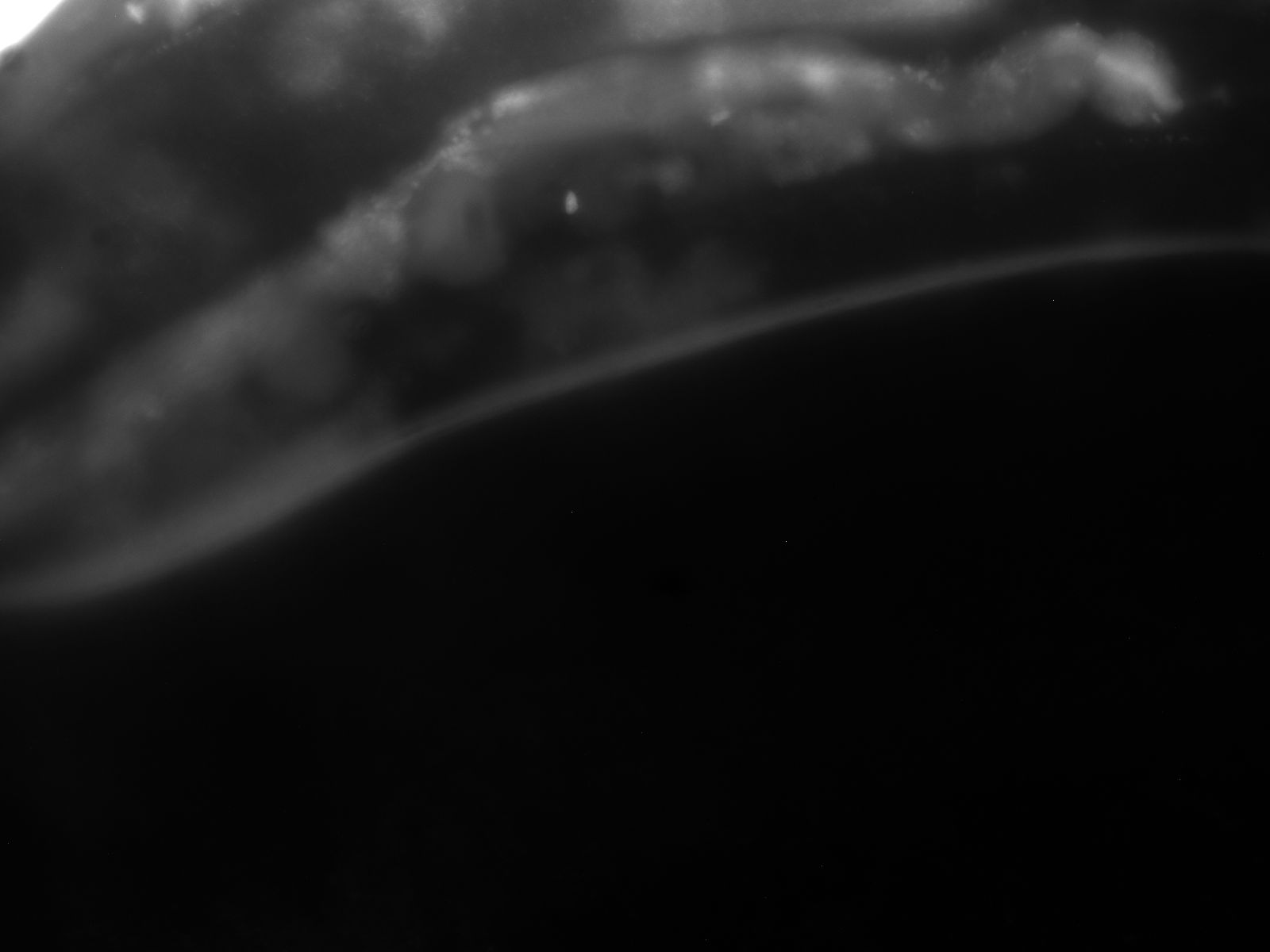

Supplement: S2 File — (ZIP) [file pgen.1011061.s002.zip › Fig.2A - Original files/Fig.2A RAW data and photos JPEG/syto12 staining - Fig 2A - 3_rep - 22.5.23 jpeg/ire-1+tfg-117.jpg]

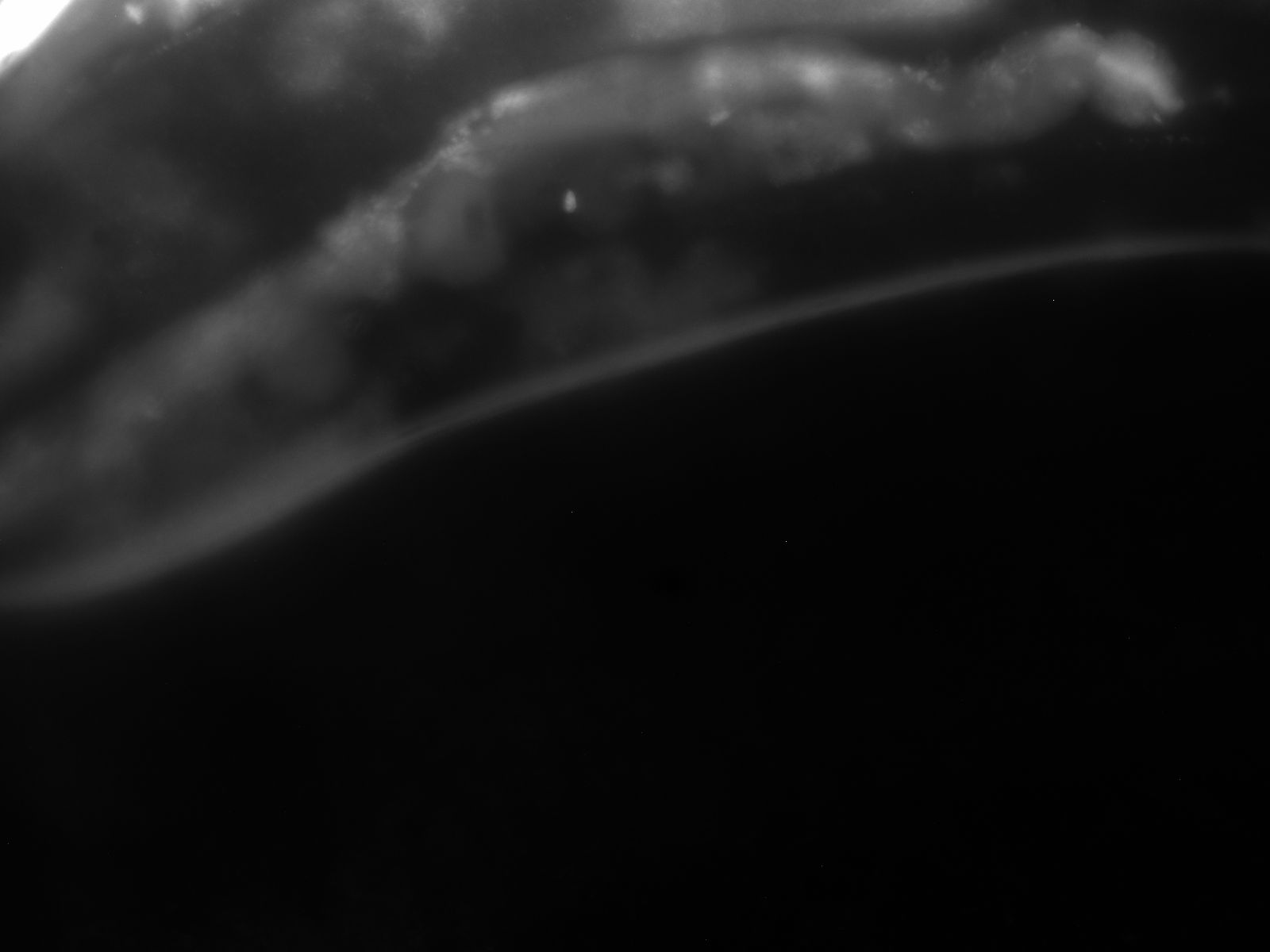

Supplement: S2 File — (ZIP) [file pgen.1011061.s002.zip › Fig.2A - Original files/Fig.2A RAW data and photos JPEG/syto12 staining - Fig 2A - 3_rep - 22.5.23 jpeg/ire-1+tfg-118.jpg]

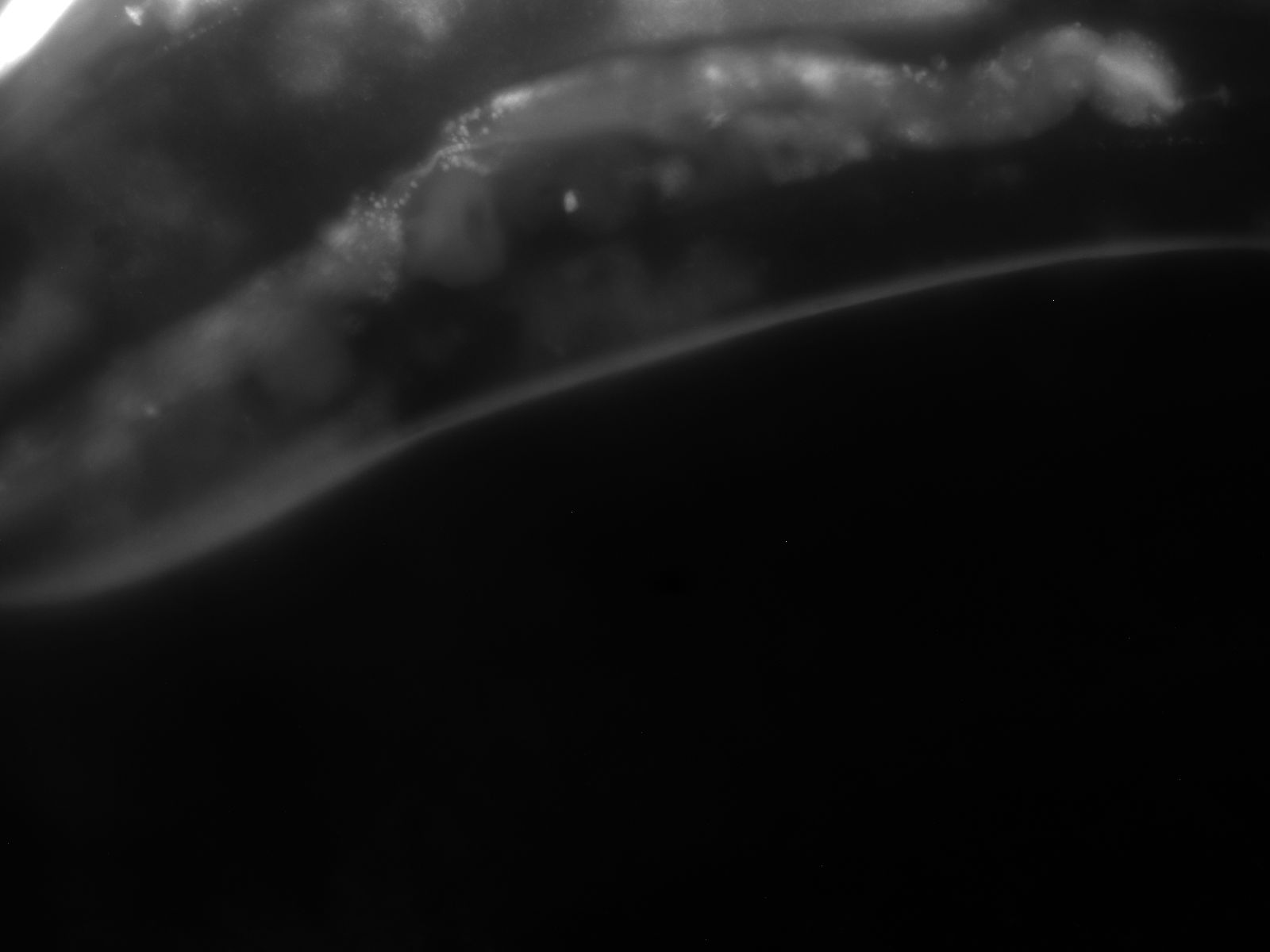

Supplement: S2 File — (ZIP) [file pgen.1011061.s002.zip › Fig.2A - Original files/Fig.2A RAW data and photos JPEG/syto12 staining - Fig 2A - 3_rep - 22.5.23 jpeg/ire-1+tfg-119.jpg]

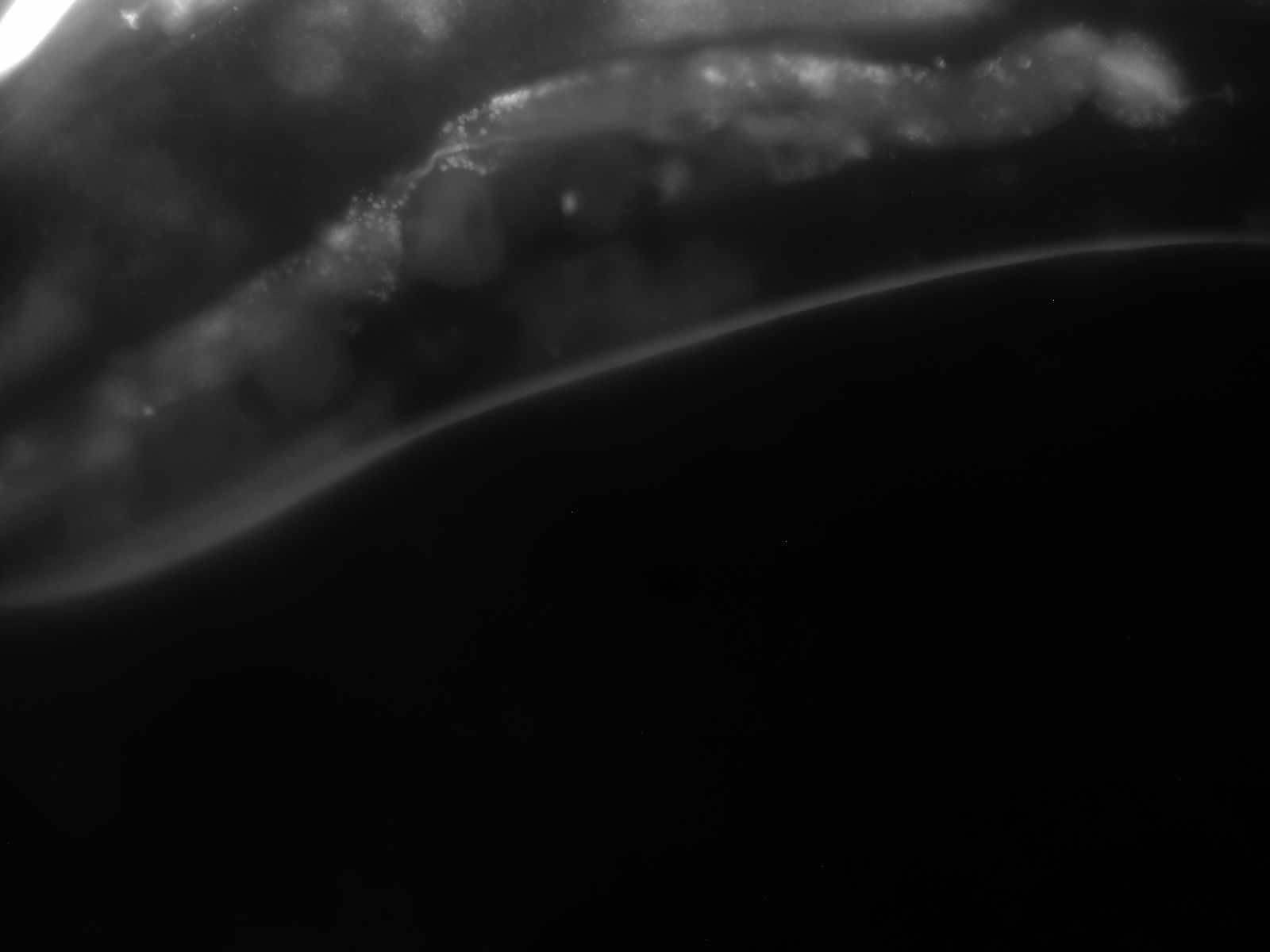

Supplement: S2 File — (ZIP) [file pgen.1011061.s002.zip › Fig.2A - Original files/Fig.2A RAW data and photos JPEG/syto12 staining - Fig 2A - 3_rep - 22.5.23 jpeg/ire-1+tfg-120.jpg]

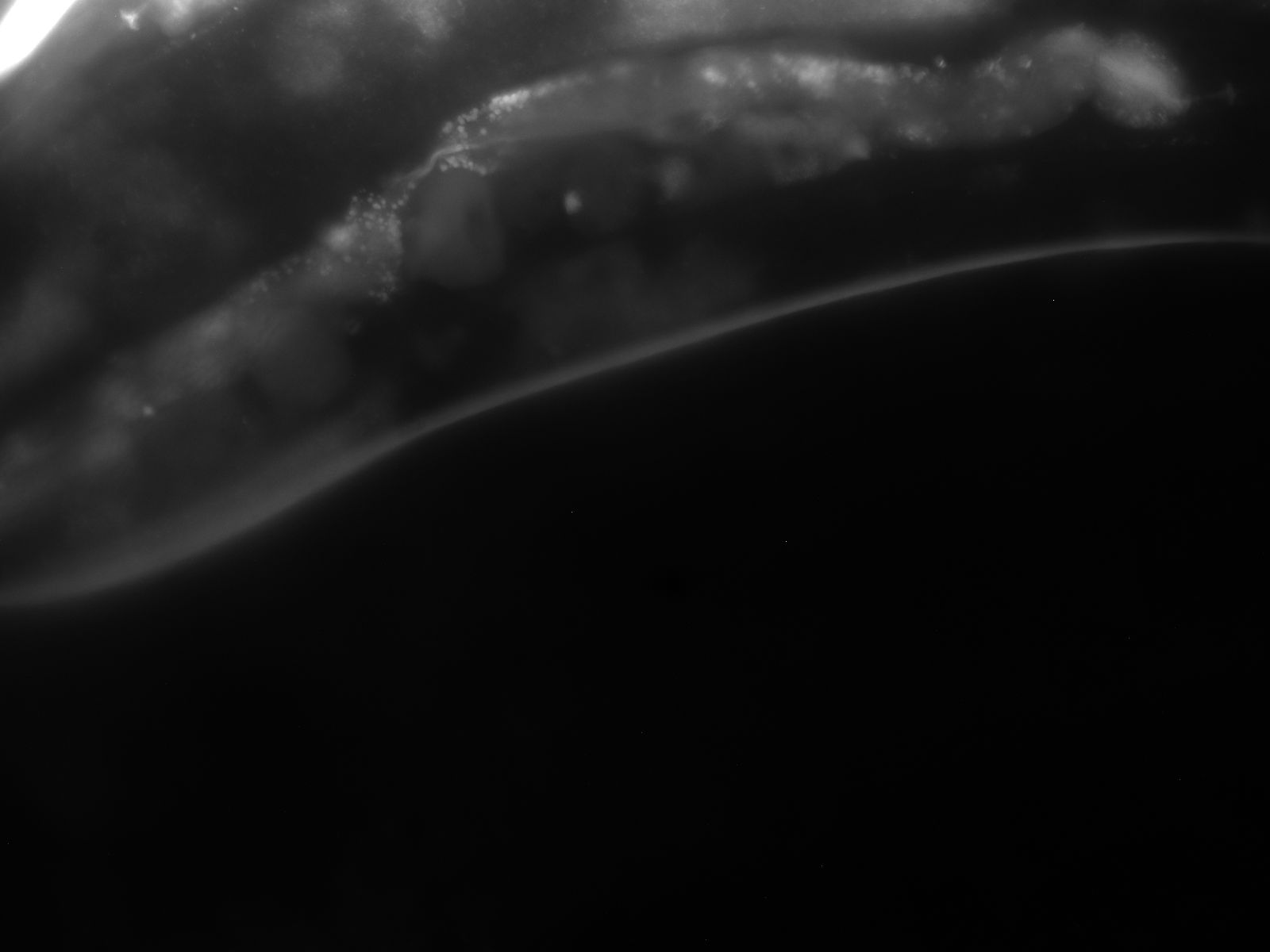

Supplement: S2 File — (ZIP) [file pgen.1011061.s002.zip › Fig.2A - Original files/Fig.2A RAW data and photos JPEG/syto12 staining - Fig 2A - 3_rep - 22.5.23 jpeg/ire-1+tfg-121.jpg]

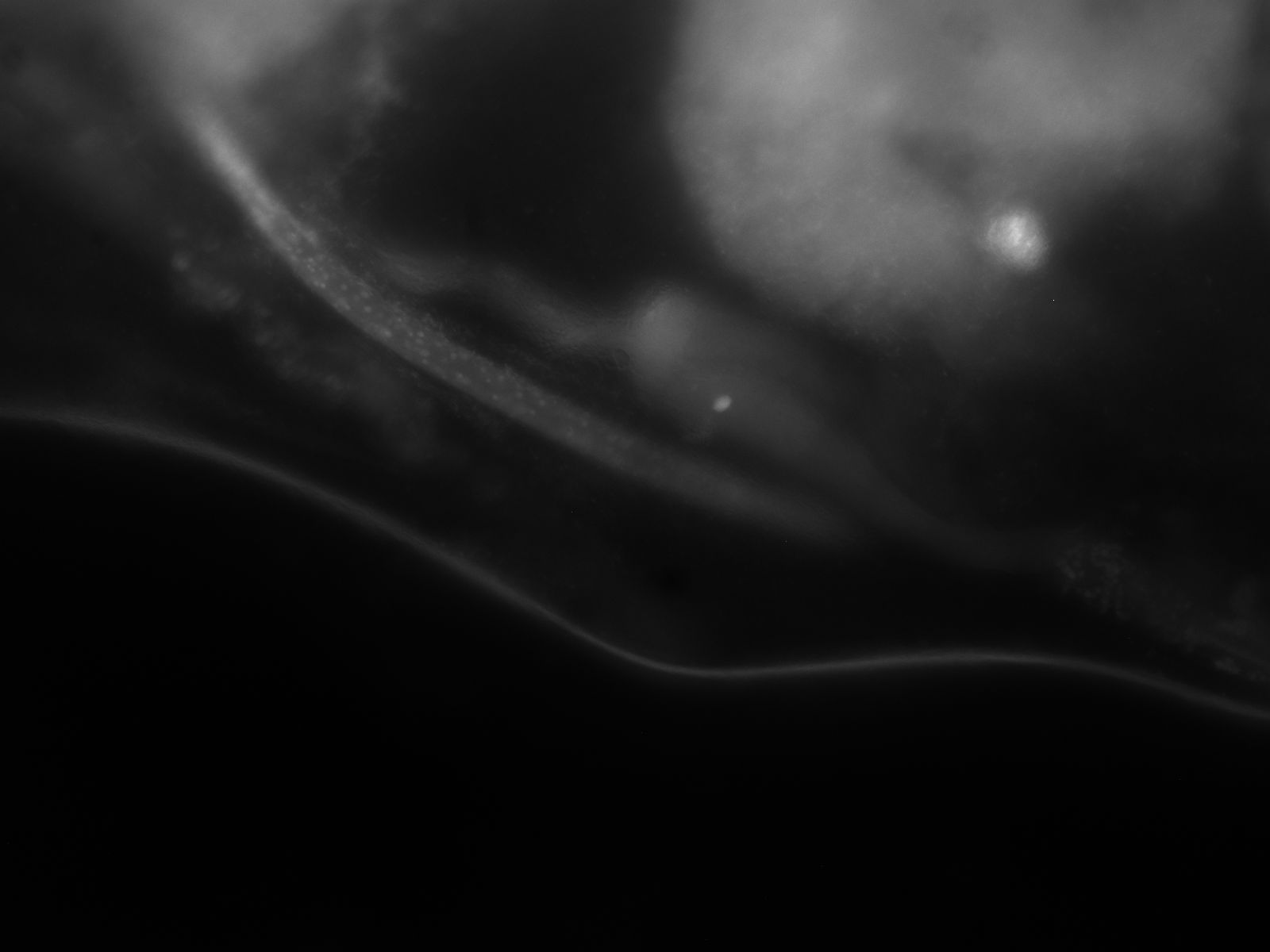

Supplement: S2 File — (ZIP) [file pgen.1011061.s002.zip › Fig.2A - Original files/Fig.2A RAW data and photos JPEG/syto12 staining - Fig 2A - 3_rep - 22.5.23 jpeg/ire-1+tfg-122.jpg]

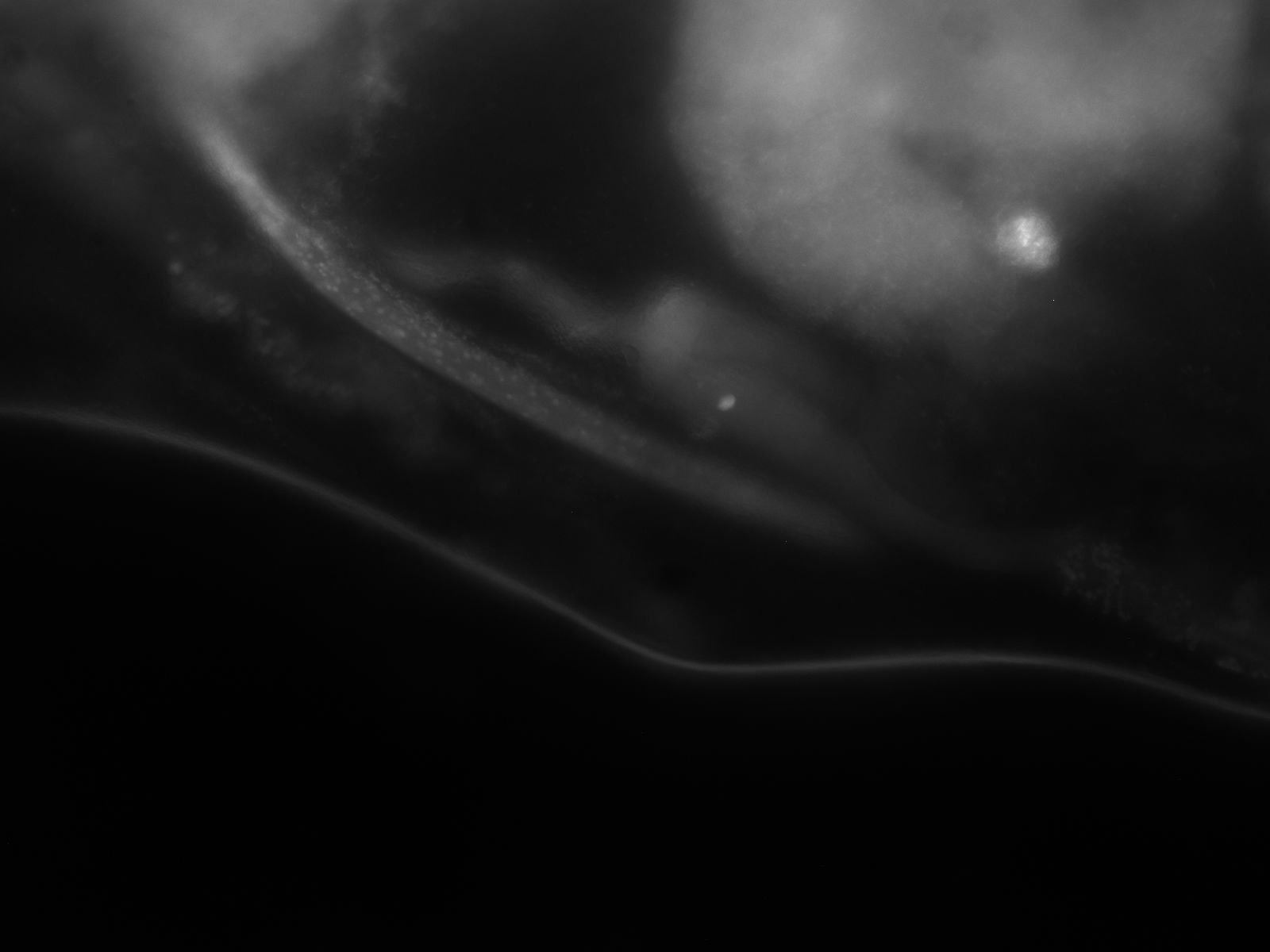

Supplement: S2 File — (ZIP) [file pgen.1011061.s002.zip › Fig.2A - Original files/Fig.2A RAW data and photos JPEG/syto12 staining - Fig 2A - 3_rep - 22.5.23 jpeg/ire-1+tfg-123.jpg]

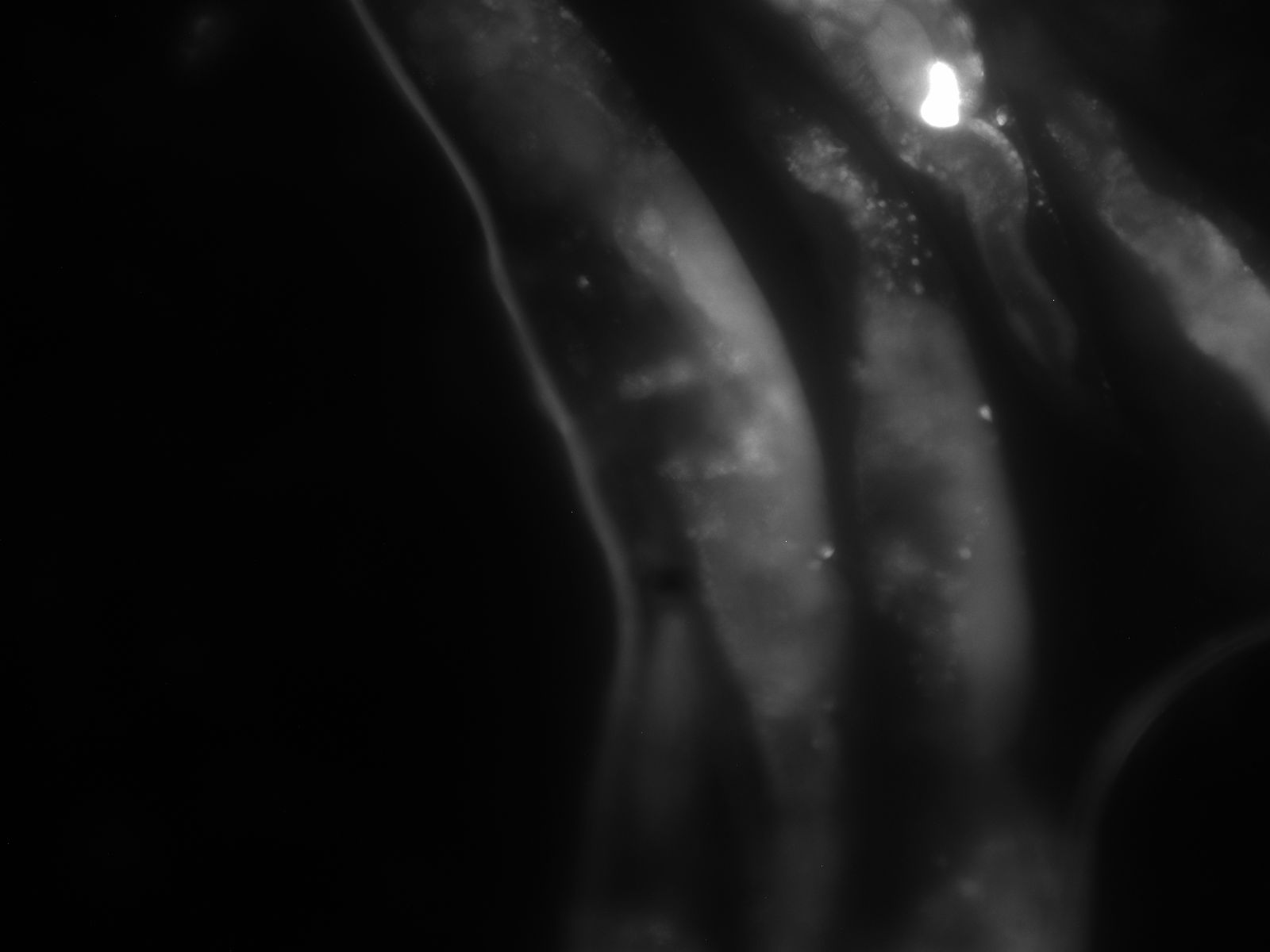

Supplement: S2 File — (ZIP) [file pgen.1011061.s002.zip › Fig.2A - Original files/Fig.2A RAW data and photos JPEG/syto12 staining - Fig 2A - 3_rep - 22.5.23 jpeg/ire-1+tfg-124.jpg]

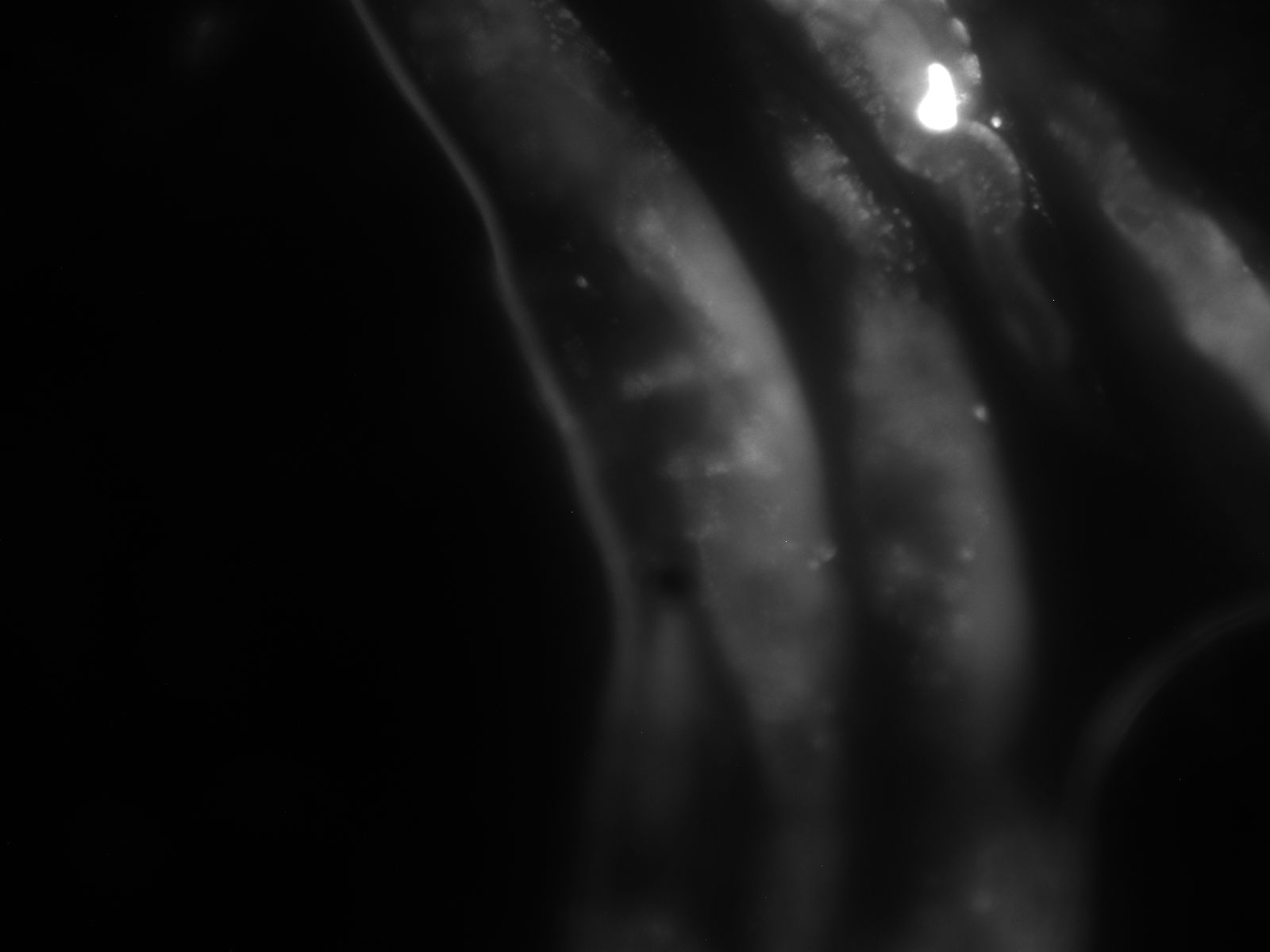

Supplement: S2 File — (ZIP) [file pgen.1011061.s002.zip › Fig.2A - Original files/Fig.2A RAW data and photos JPEG/syto12 staining - Fig 2A - 3_rep - 22.5.23 jpeg/ire-1+tfg-125.jpg]

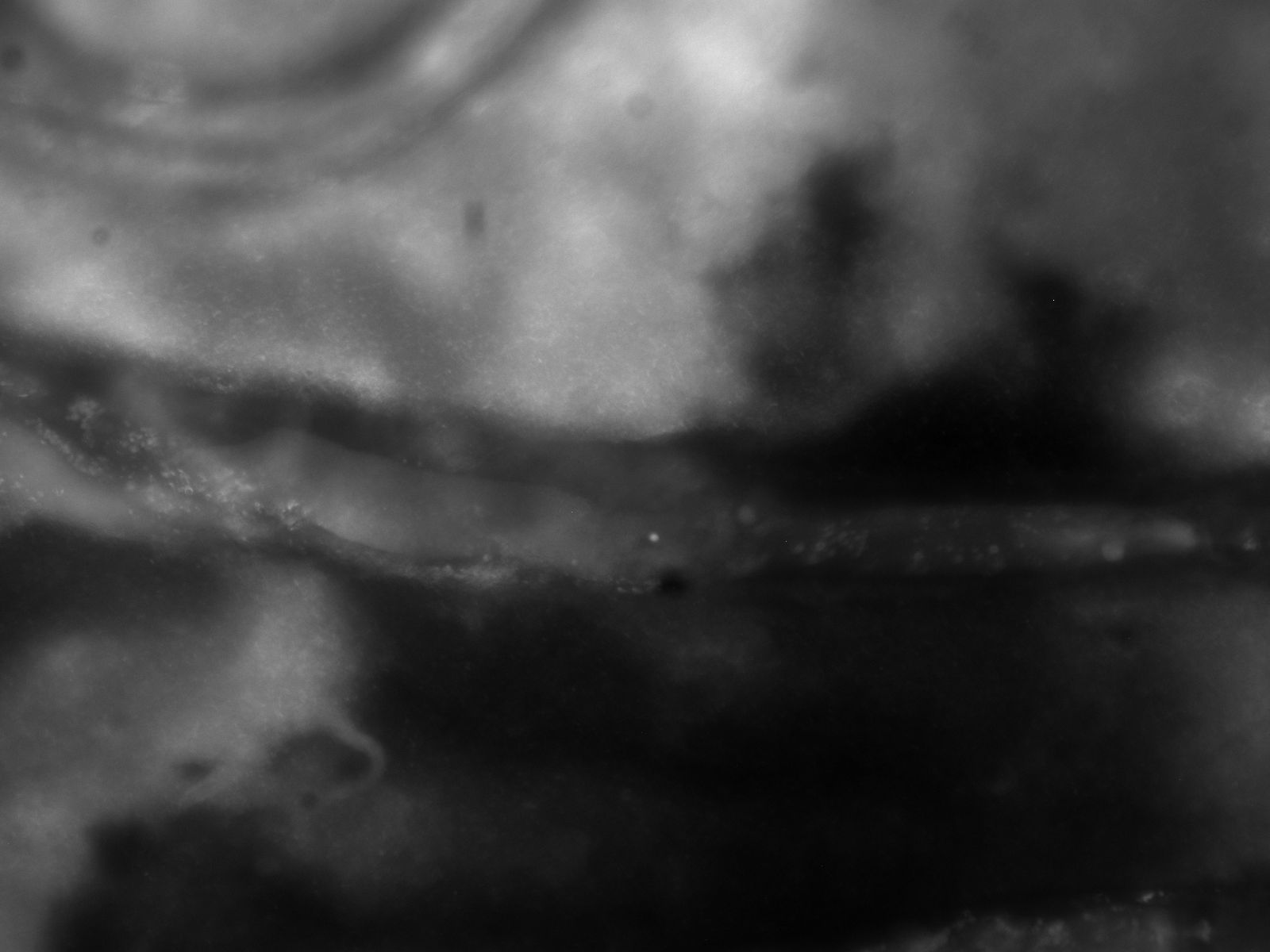

Supplement: S2 File — (ZIP) [file pgen.1011061.s002.zip › Fig.2A - Original files/Fig.2A RAW data and photos JPEG/syto12 staining - Fig 2A - 3_rep - 22.5.23 jpeg/ire-1+tfg-126.jpg]

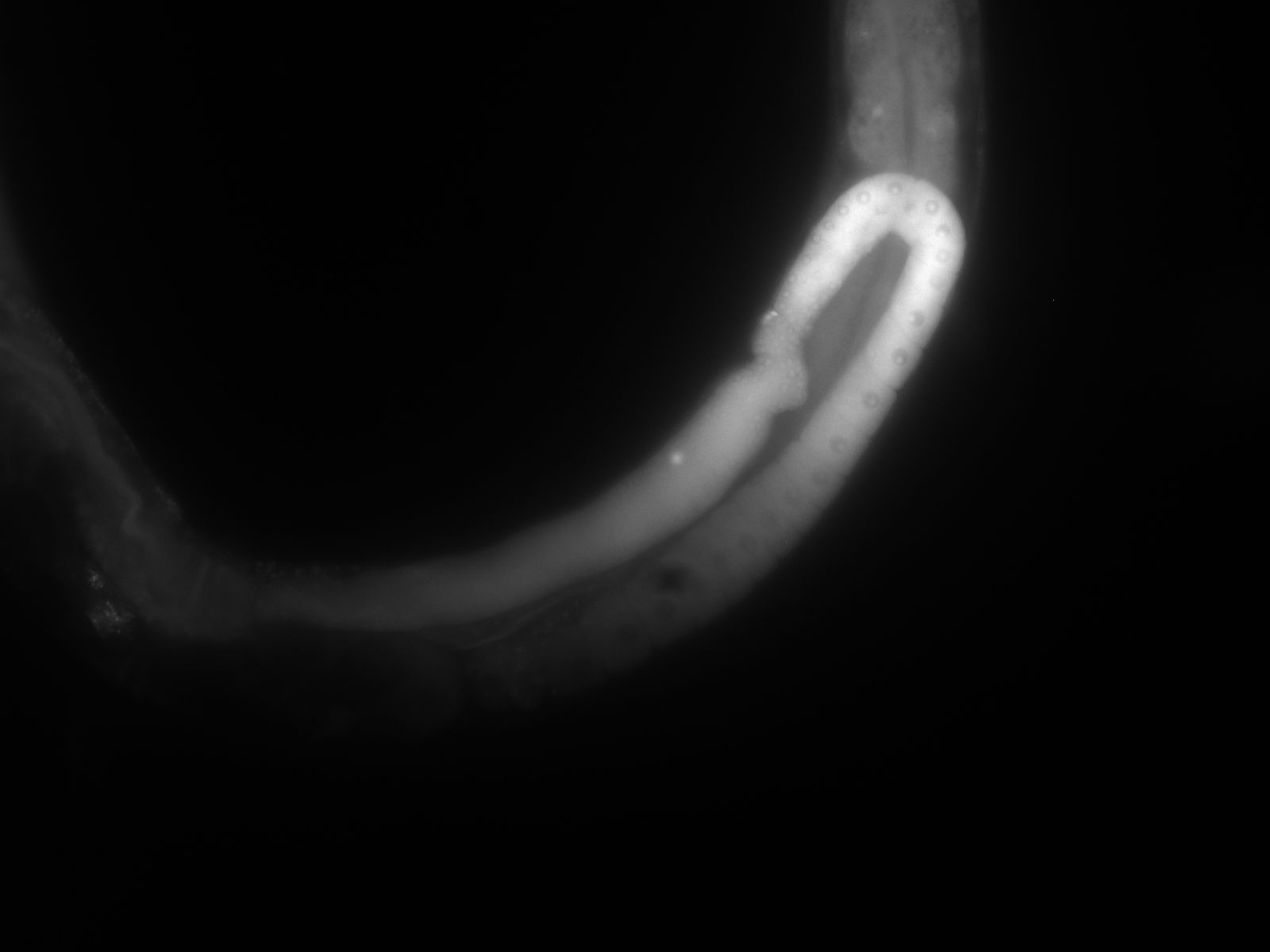

Supplement: S2 File — (ZIP) [file pgen.1011061.s002.zip › Fig.2A - Original files/Fig.2A RAW data and photos JPEG/syto12 staining - Fig 2A - 3_rep - 22.5.23 jpeg/n2+pad1200.jpg]

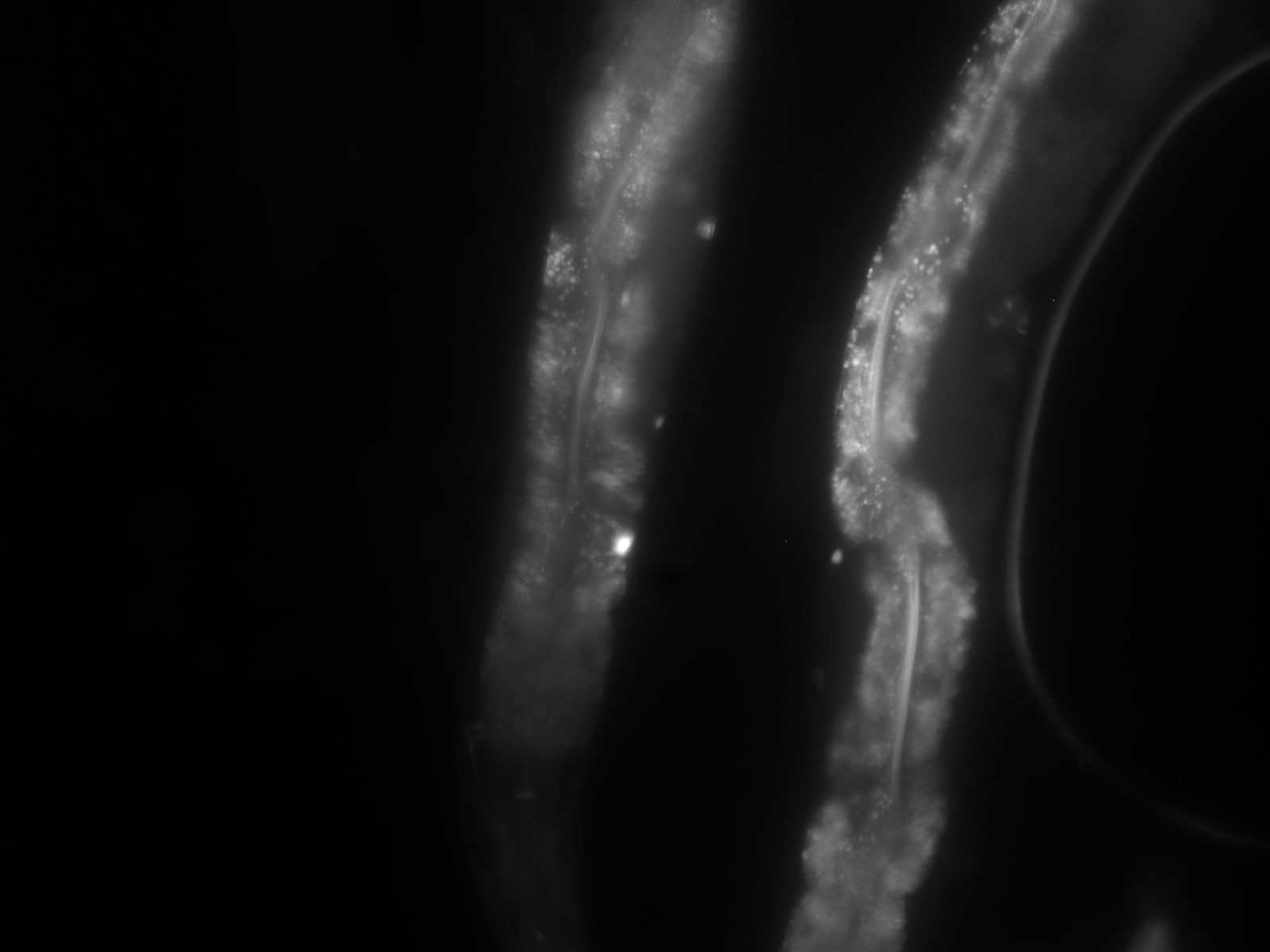

Supplement: S2 File — (ZIP) [file pgen.1011061.s002.zip › Fig.2A - Original files/Fig.2A RAW data and photos JPEG/syto12 staining - Fig 2A - 3_rep - 22.5.23 jpeg/n2+pad1201.jpg]

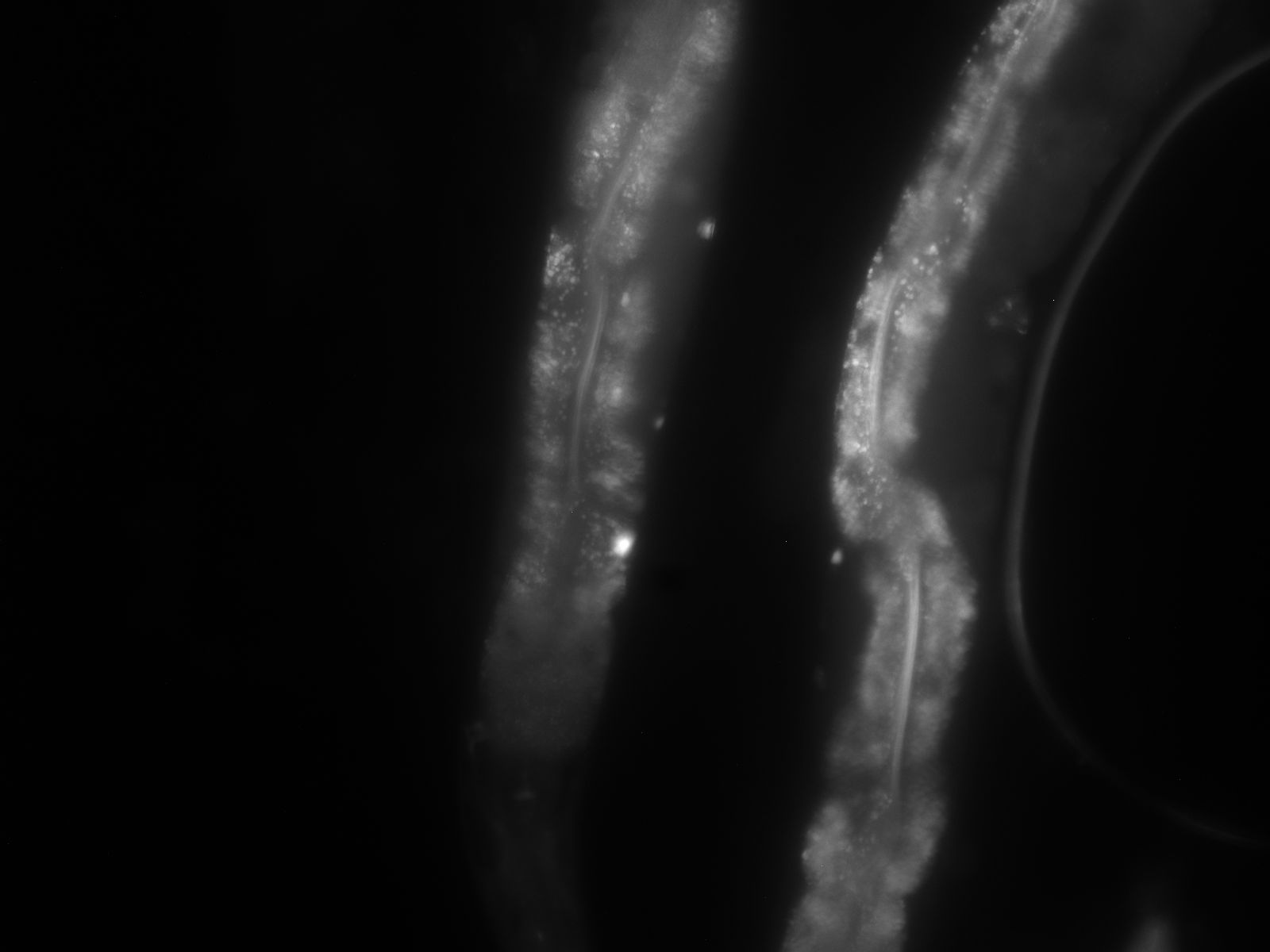

Supplement: S2 File — (ZIP) [file pgen.1011061.s002.zip › Fig.2A - Original files/Fig.2A RAW data and photos JPEG/syto12 staining - Fig 2A - 3_rep - 22.5.23 jpeg/n2+pad1202.jpg]

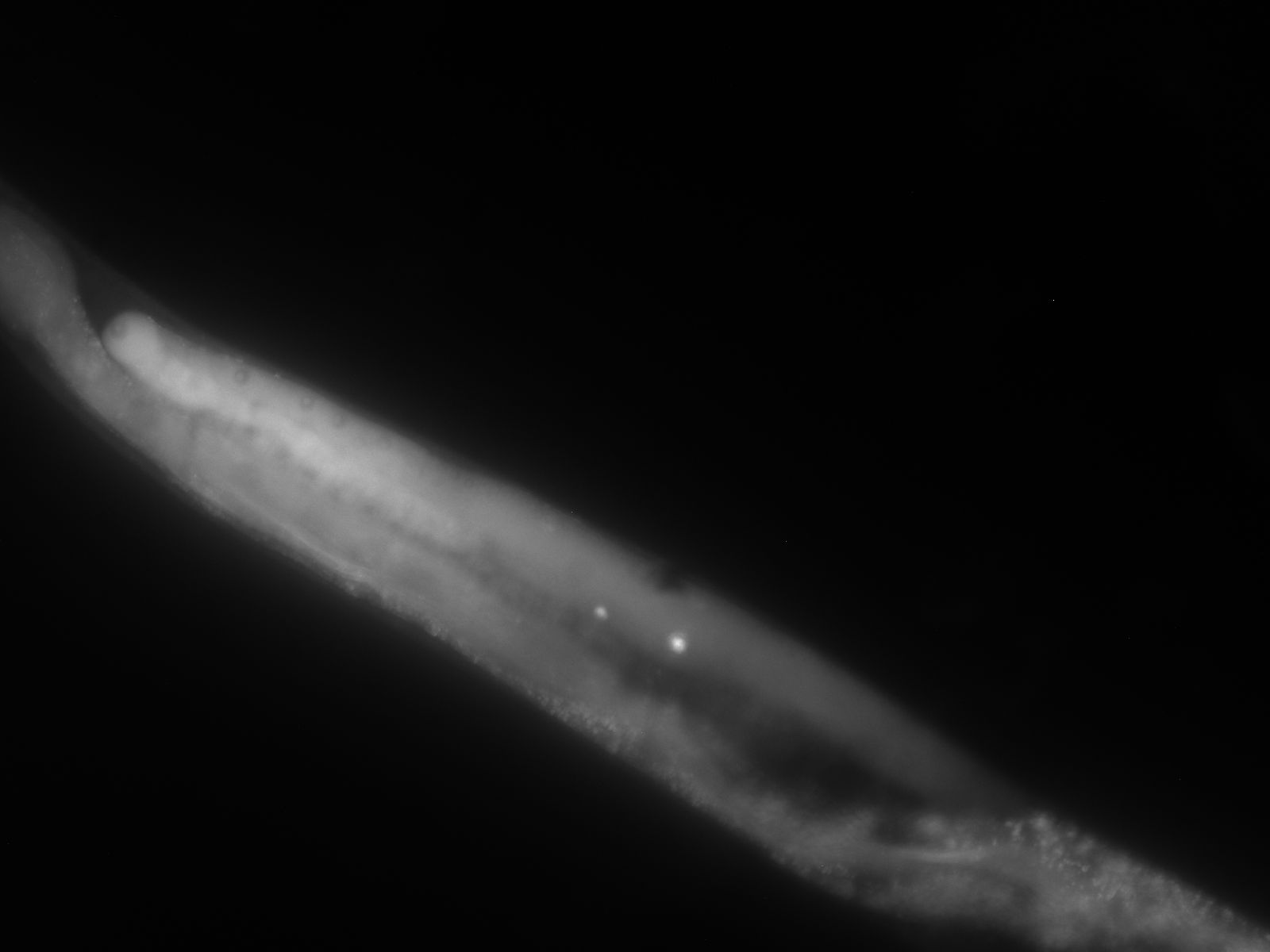

Supplement: S2 File — (ZIP) [file pgen.1011061.s002.zip › Fig.2A - Original files/Fig.2A RAW data and photos JPEG/syto12 staining - Fig 2A - 3_rep - 22.5.23 jpeg/n2+pad1203.jpg]

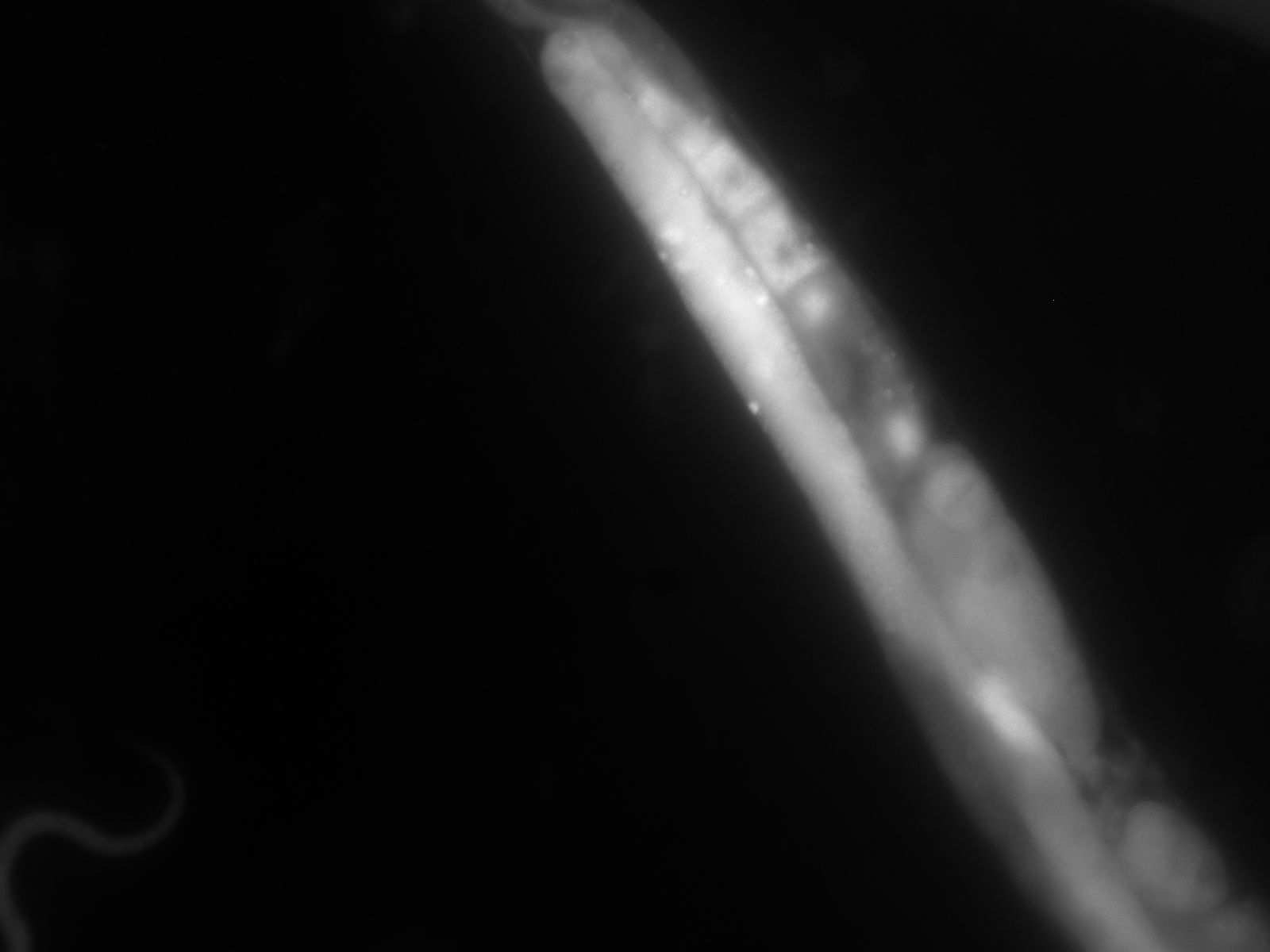

Supplement: S2 File — (ZIP) [file pgen.1011061.s002.zip › Fig.2A - Original files/Fig.2A RAW data and photos JPEG/syto12 staining - Fig 2A - 3_rep - 22.5.23 jpeg/n2+tfg-110.jpg]

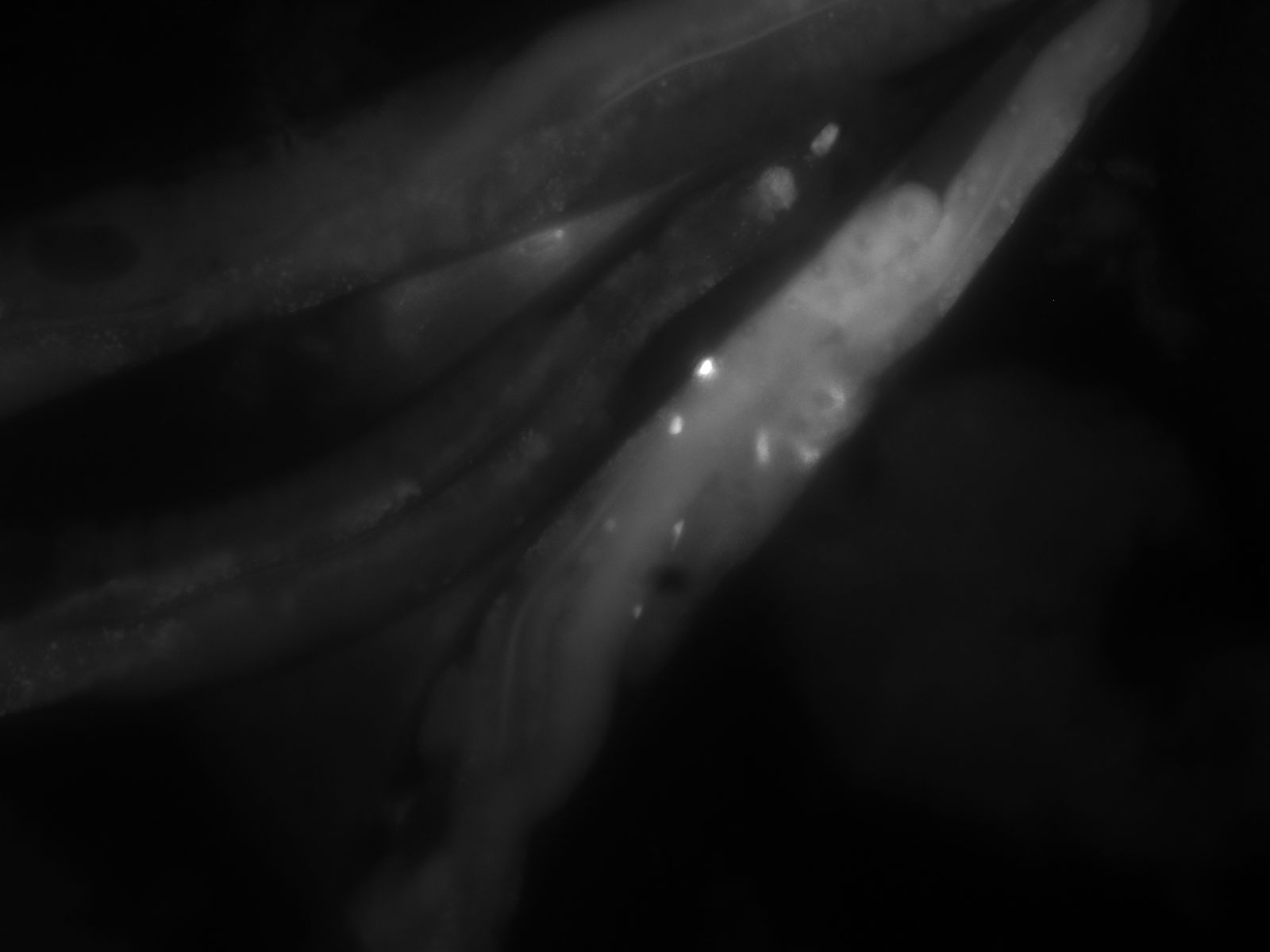

Supplement: S2 File — (ZIP) [file pgen.1011061.s002.zip › Fig.2A - Original files/Fig.2A RAW data and photos JPEG/syto12 staining - Fig 2A - 3_rep - 22.5.23 jpeg/n2+tfg-111.jpg]

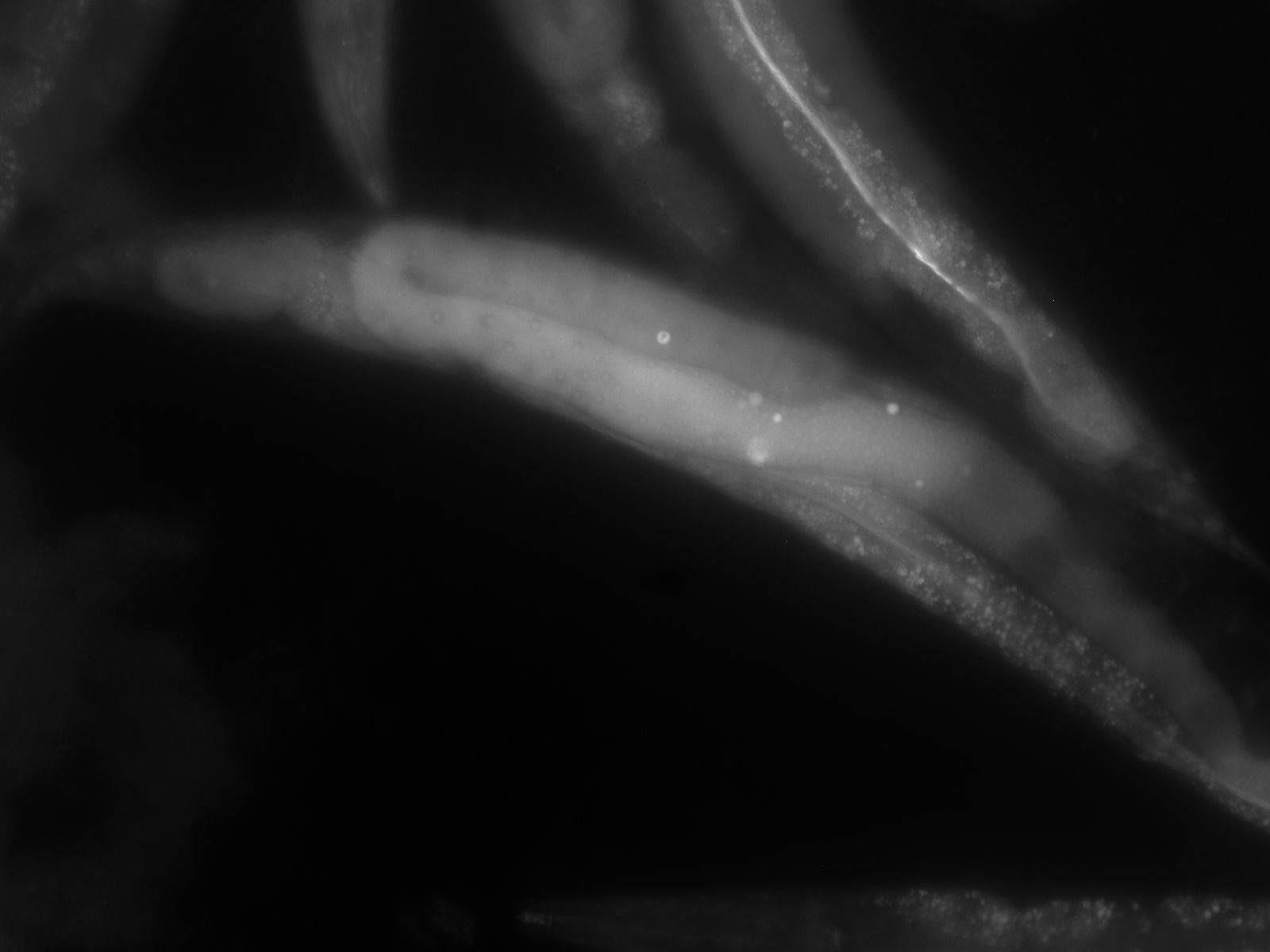

Supplement: S2 File — (ZIP) [file pgen.1011061.s002.zip › Fig.2A - Original files/Fig.2A RAW data and photos JPEG/syto12 staining - Fig 2A - 3_rep - 22.5.23 jpeg/n2+tfg-112.jpg]

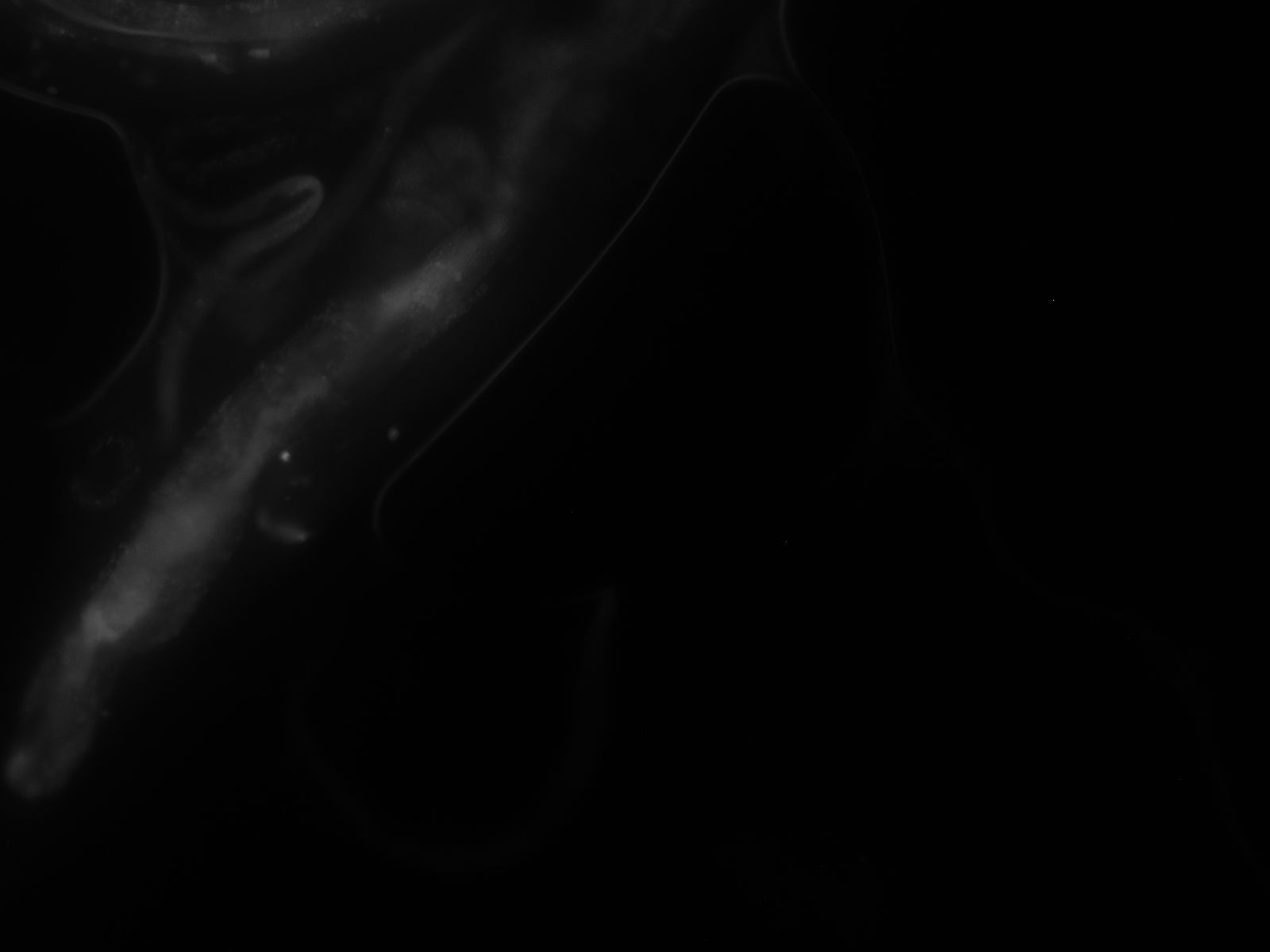

Supplement: S2 File — (ZIP) [file pgen.1011061.s002.zip › Fig.2A - Original files/Fig.2A RAW data and photos JPEG/syto12 staining - Fig 2A - 3_rep - 22.5.23 jpeg/xbp-1+pad1213.jpg]

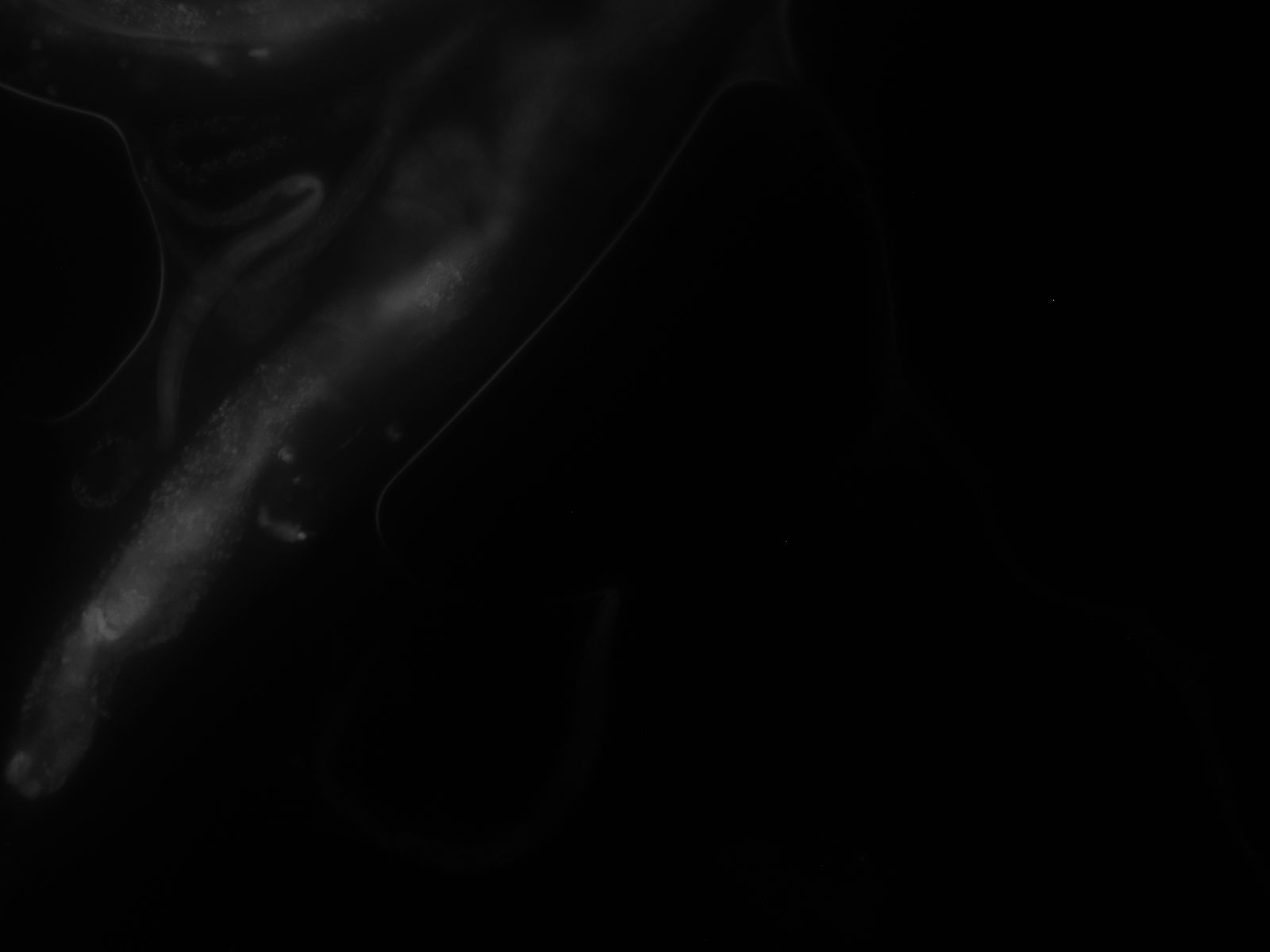

Supplement: S2 File — (ZIP) [file pgen.1011061.s002.zip › Fig.2A - Original files/Fig.2A RAW data and photos JPEG/syto12 staining - Fig 2A - 3_rep - 22.5.23 jpeg/xbp-1+pad1214.jpg]

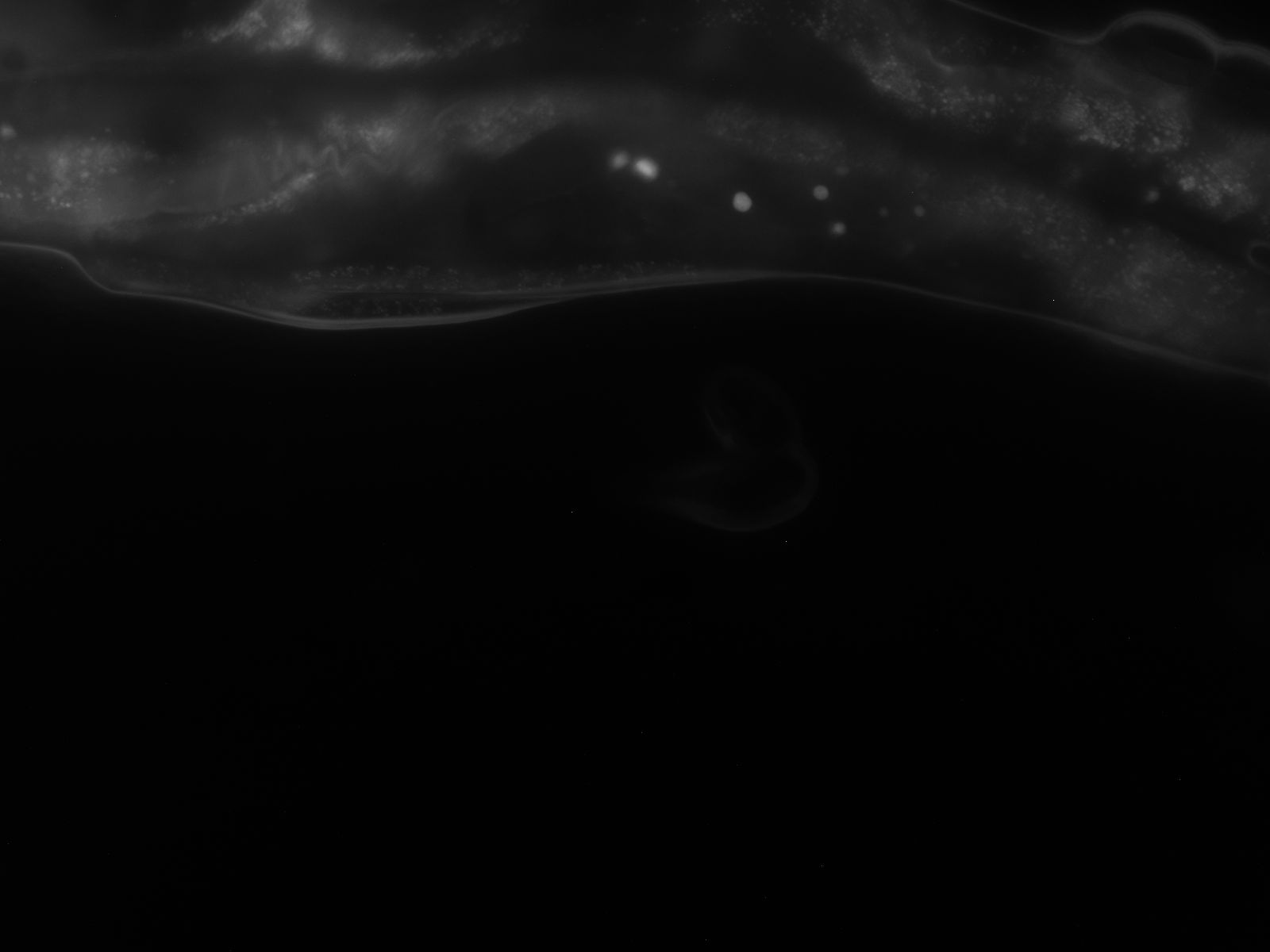

Supplement: S2 File — (ZIP) [file pgen.1011061.s002.zip › Fig.2A - Original files/Fig.2A RAW data and photos JPEG/syto12 staining - Fig 2A - 3_rep - 22.5.23 jpeg/xbp-1+pad1215.jpg]

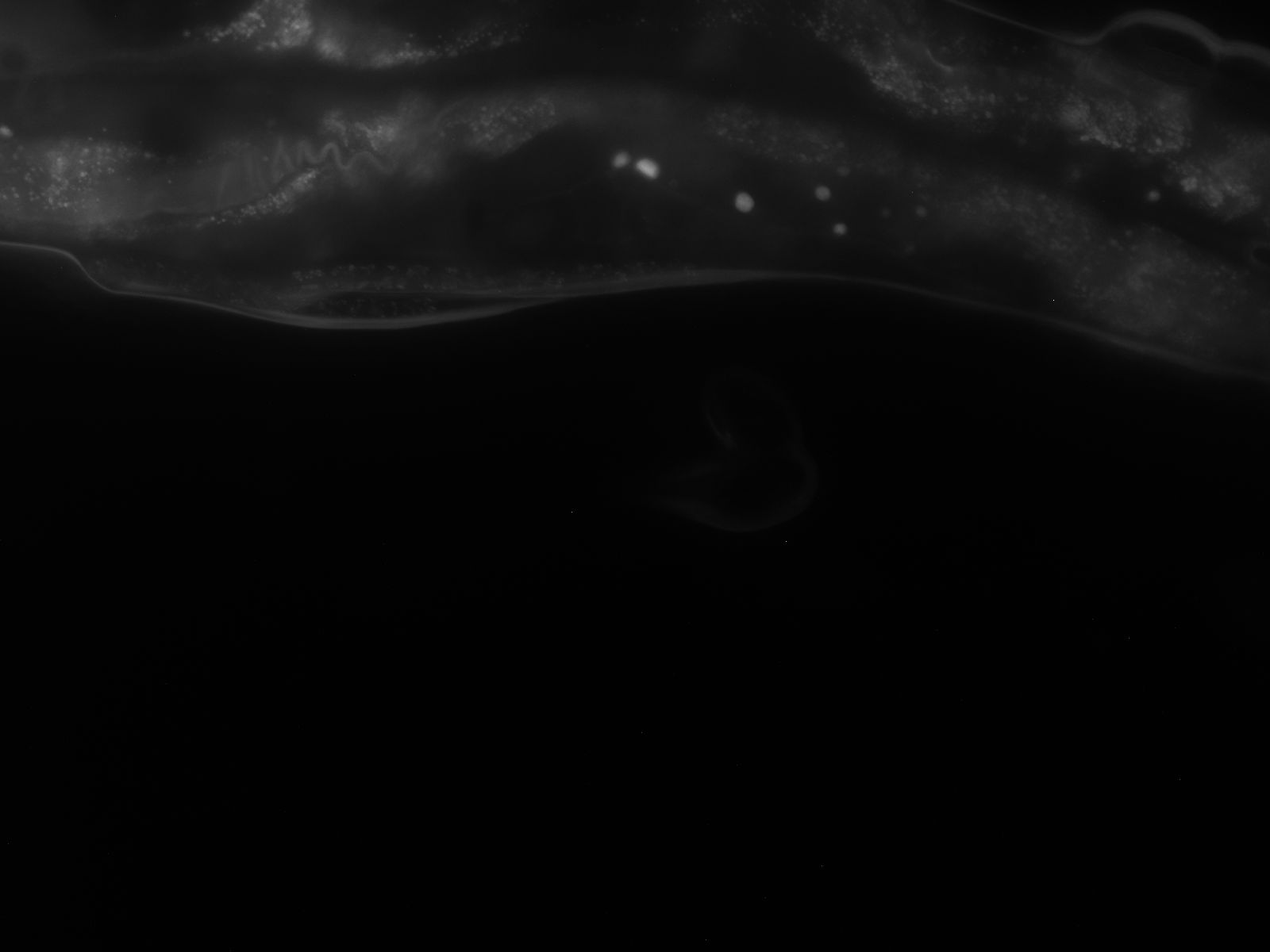

Supplement: S2 File — (ZIP) [file pgen.1011061.s002.zip › Fig.2A - Original files/Fig.2A RAW data and photos JPEG/syto12 staining - Fig 2A - 3_rep - 22.5.23 jpeg/xbp-1+pad1216.jpg]

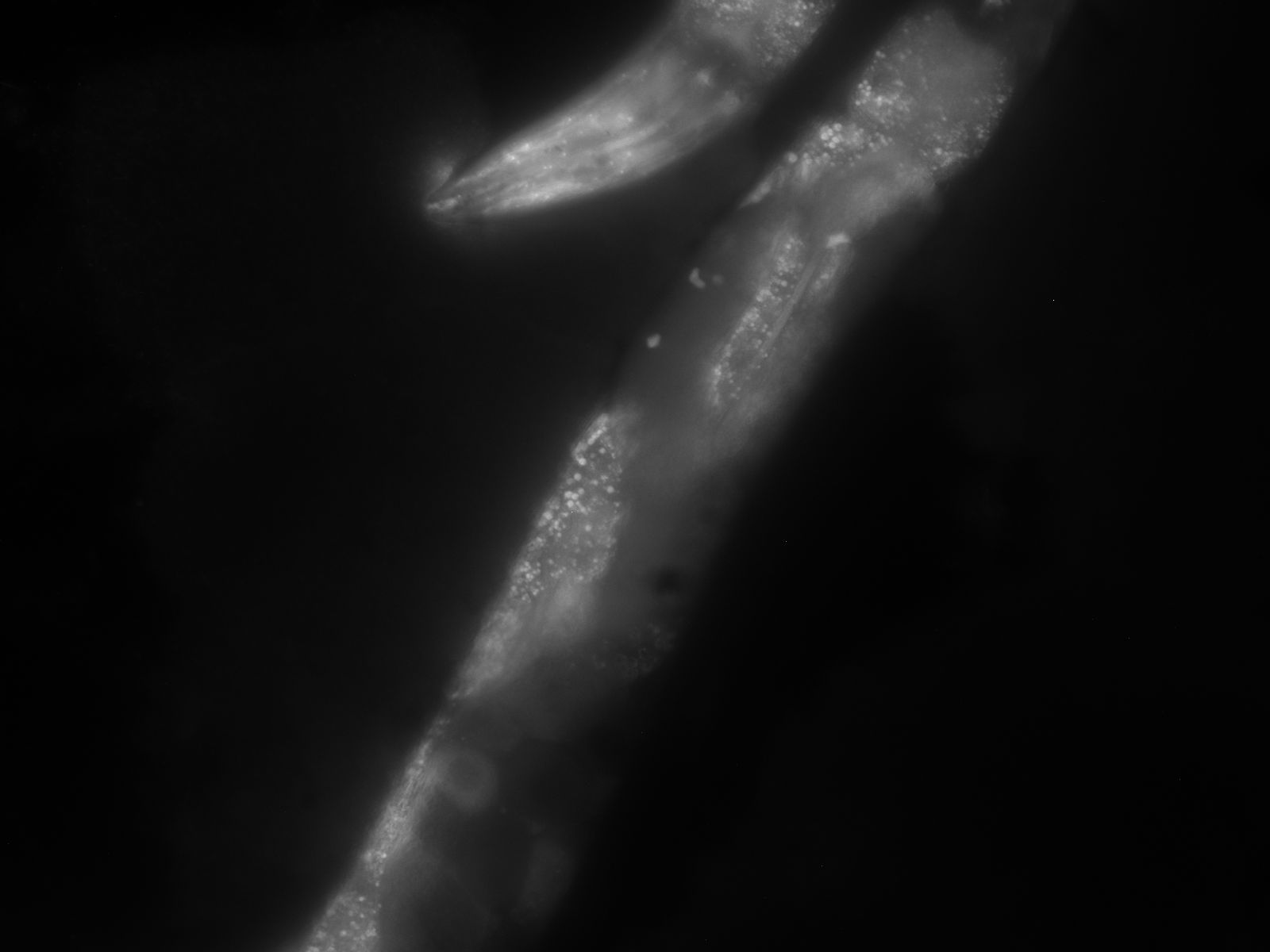

Supplement: S2 File — (ZIP) [file pgen.1011061.s002.zip › Fig.2A - Original files/Fig.2A RAW data and photos JPEG/syto12 staining - fig 2A - 1_rep - 14.5.23 jpeg/n2+tfg105.jpg]

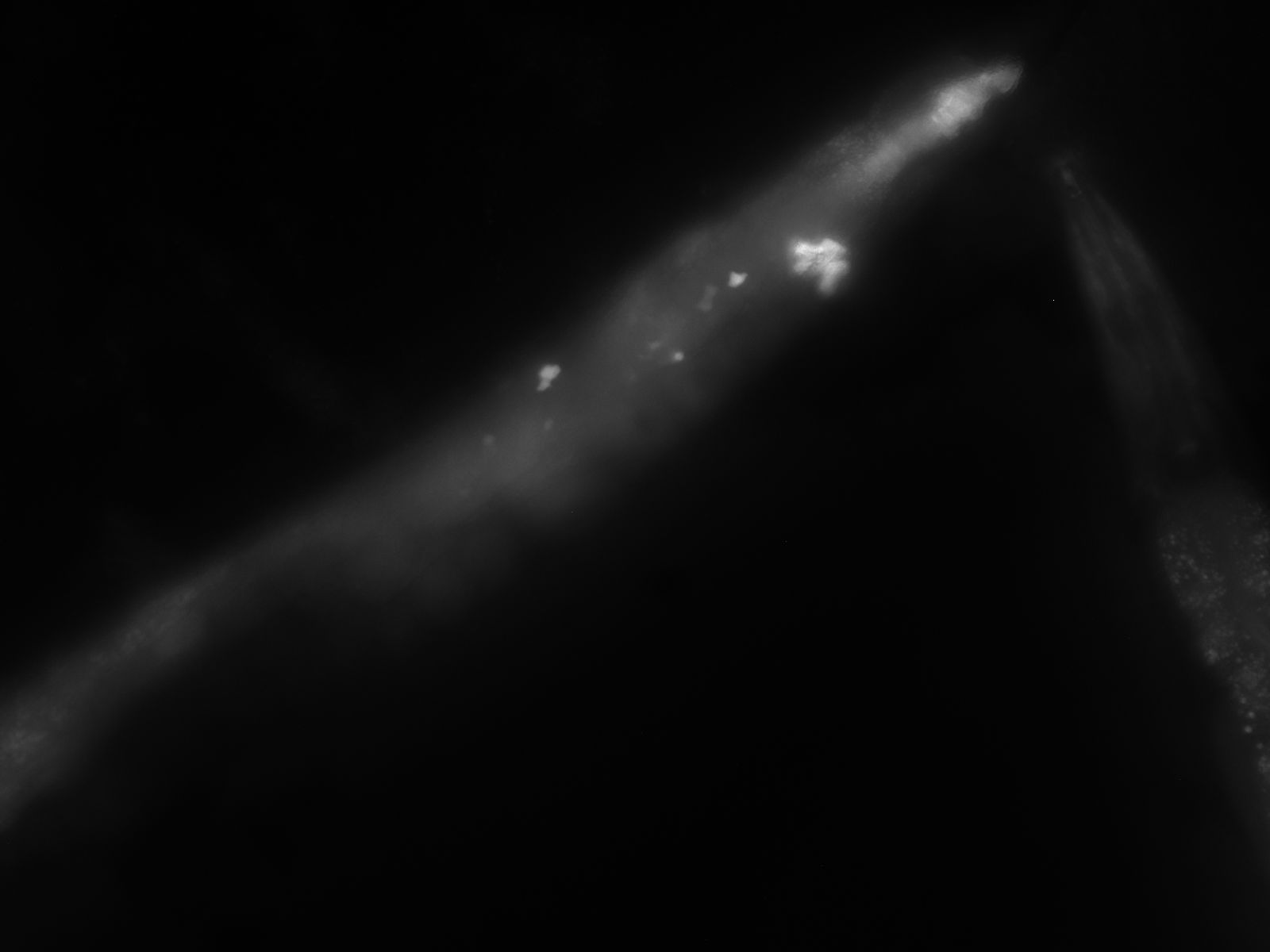

Supplement: S2 File — (ZIP) [file pgen.1011061.s002.zip › Fig.2A - Original files/Fig.2A RAW data and photos JPEG/syto12 staining - fig 2A - 1_rep - 14.5.23 jpeg/n2+tfg106.jpg]

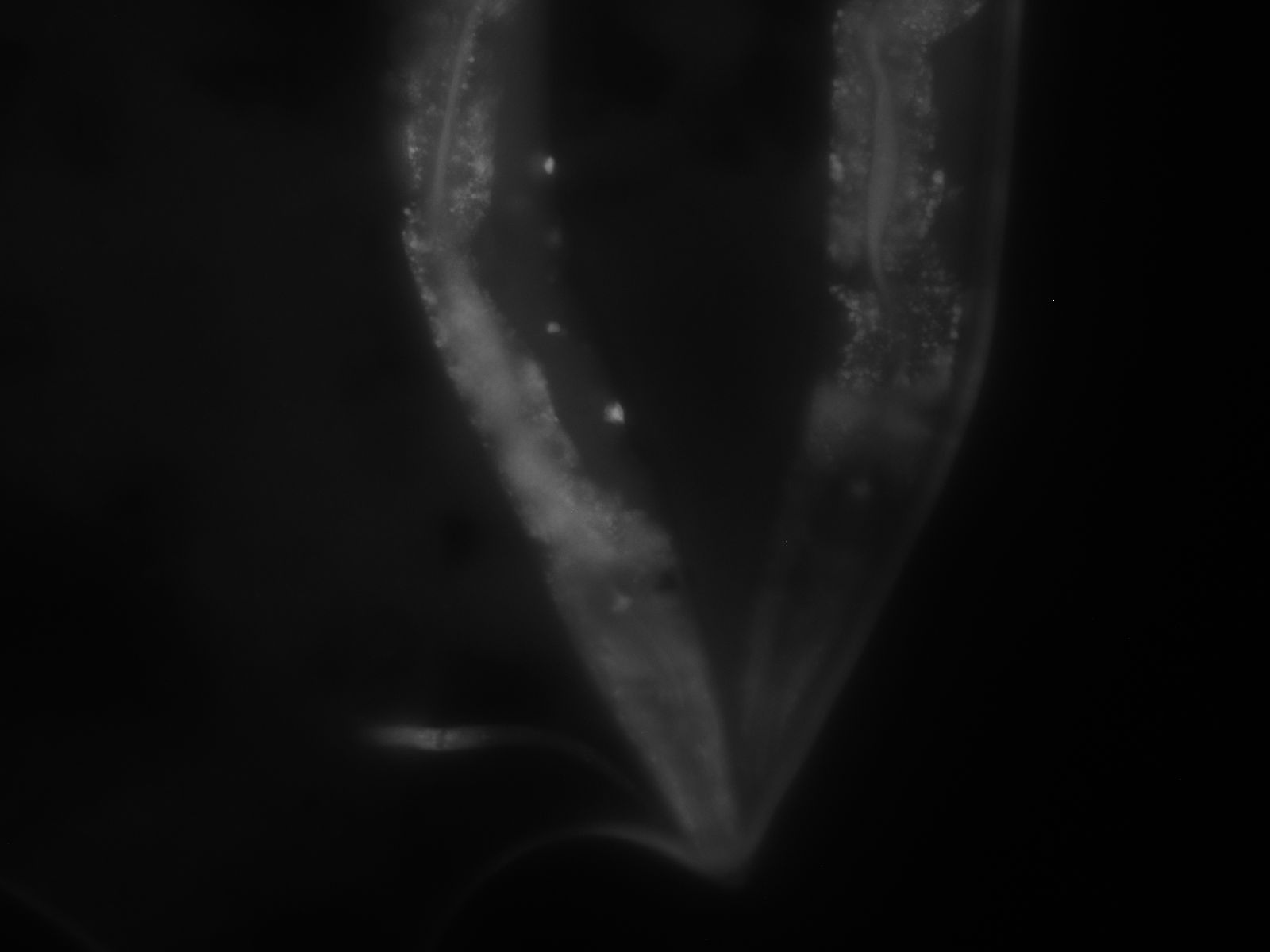

Supplement: S2 File — (ZIP) [file pgen.1011061.s002.zip › Fig.2A - Original files/Fig.2A RAW data and photos JPEG/syto12 staining - fig 2A - 1_rep - 14.5.23 jpeg/n2+tfg107.jpg]

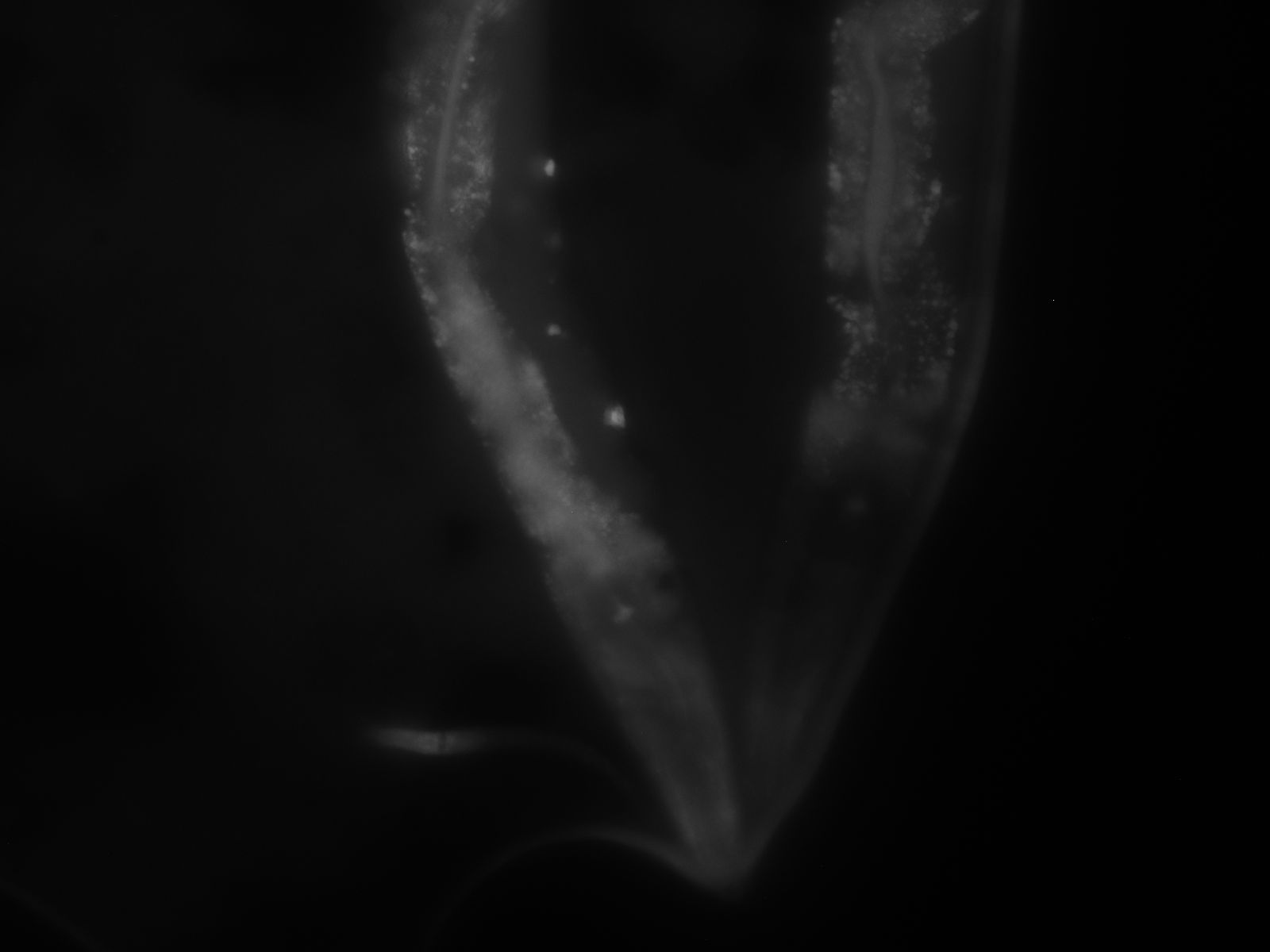

Supplement: S2 File — (ZIP) [file pgen.1011061.s002.zip › Fig.2A - Original files/Fig.2A RAW data and photos JPEG/syto12 staining - fig 2A - 1_rep - 14.5.23 jpeg/n2+tfg108.jpg]

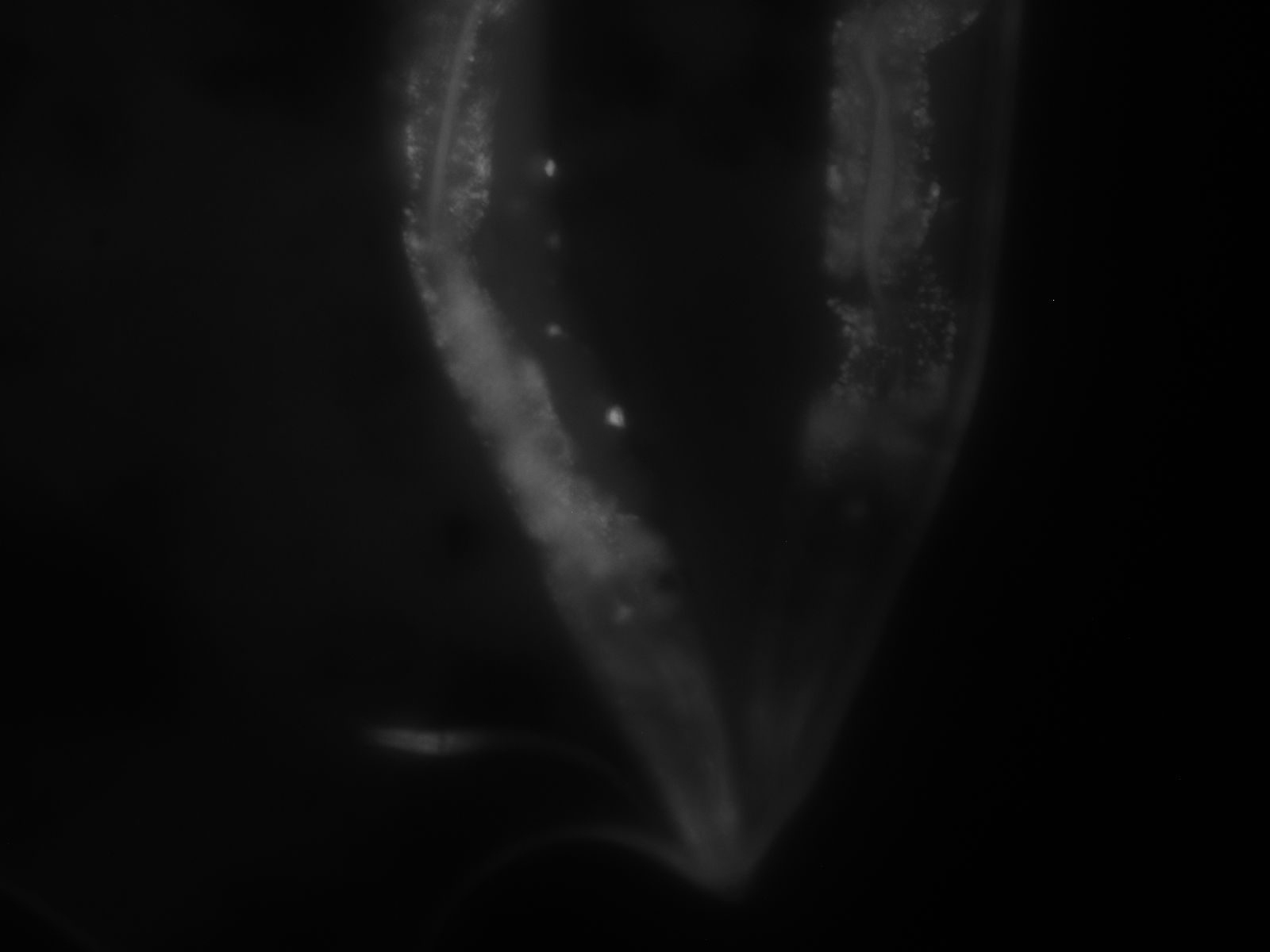

Supplement: S2 File — (ZIP) [file pgen.1011061.s002.zip › Fig.2A - Original files/Fig.2A RAW data and photos JPEG/syto12 staining - fig 2A - 1_rep - 14.5.23 jpeg/n2+tfg109.jpg]

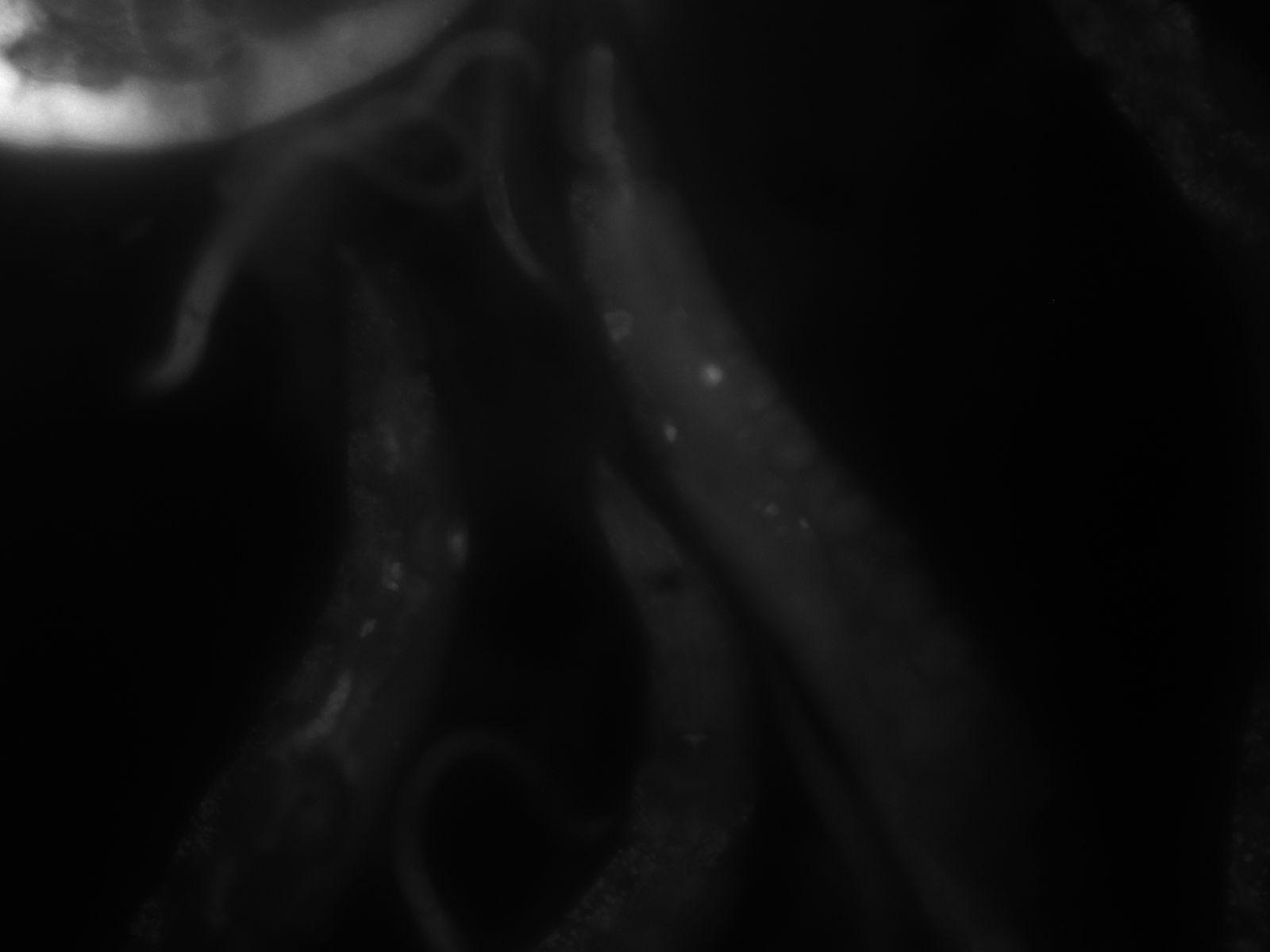

Supplement: S2 File — (ZIP) [file pgen.1011061.s002.zip › Fig.2A - Original files/Fig.2A RAW data and photos JPEG/syto12 staining - fig 2A - 1_rep - 14.5.23 jpeg/n2+tfg110.jpg]

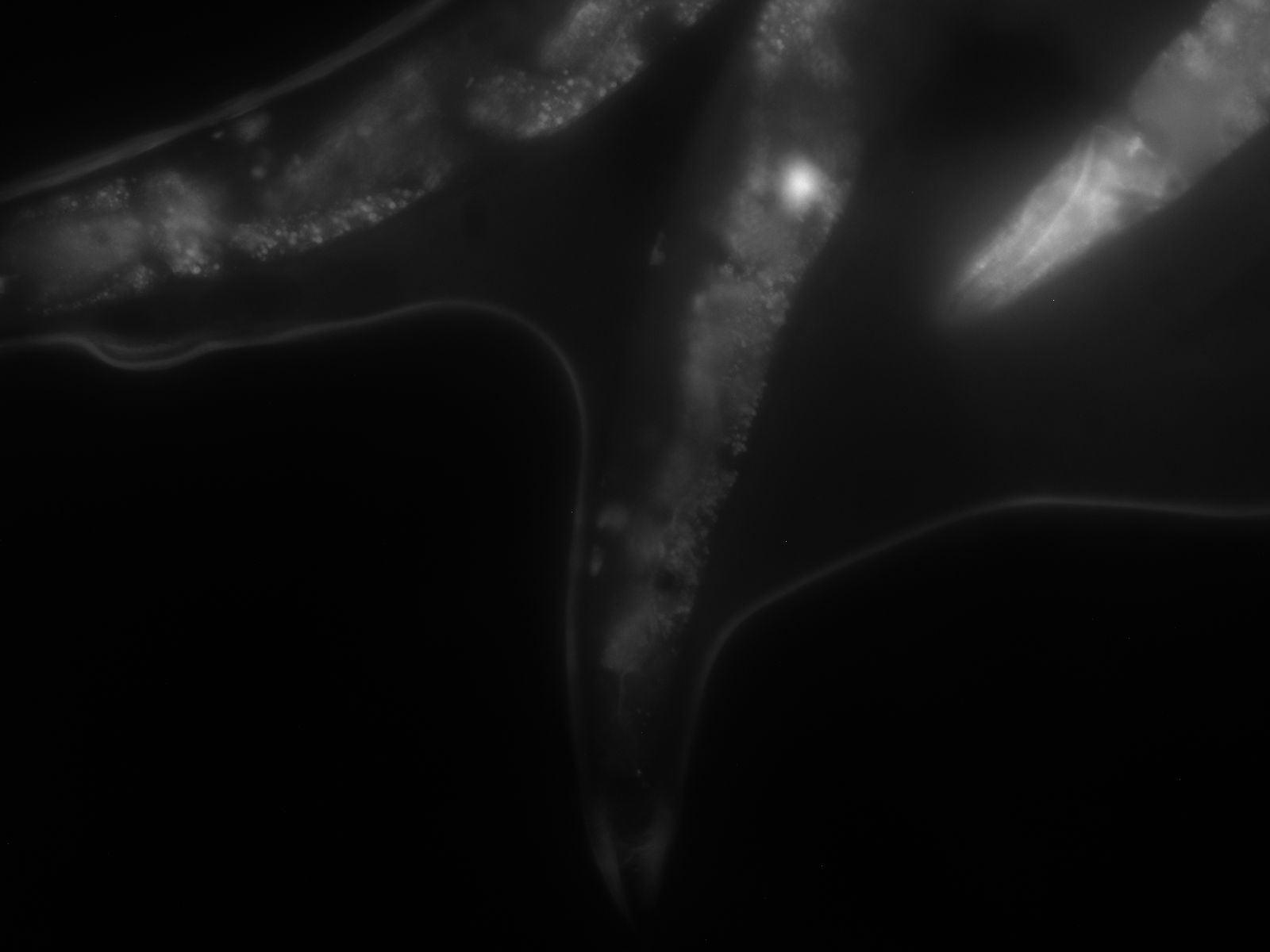

Supplement: S2 File — (ZIP) [file pgen.1011061.s002.zip › Fig.2A - Original files/Fig.2A RAW data and photos JPEG/syto12 staining - fig 2A - 1_rep - 14.5.23 jpeg/n2+tfg111.jpg]

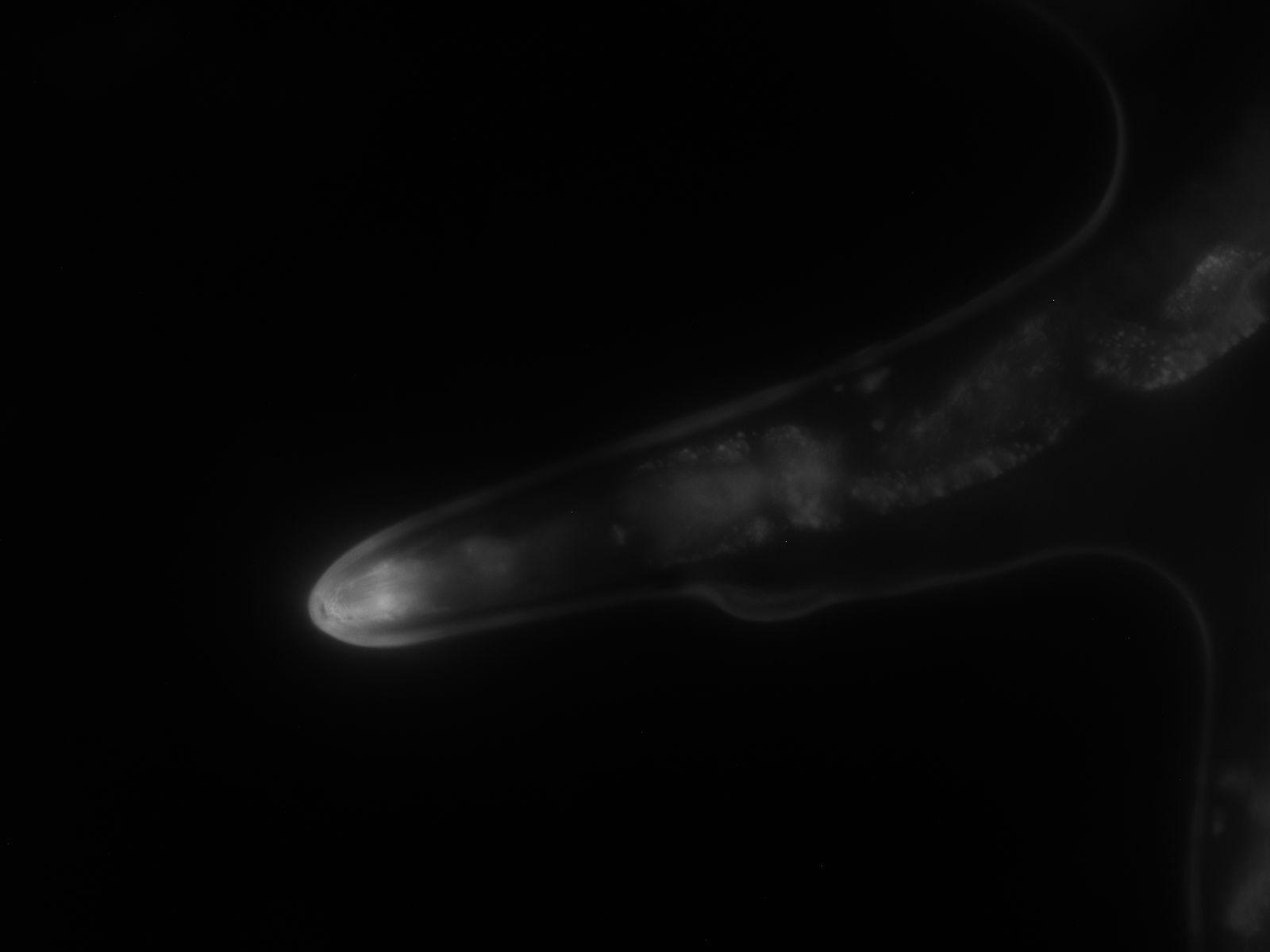

Supplement: S2 File — (ZIP) [file pgen.1011061.s002.zip › Fig.2A - Original files/Fig.2A RAW data and photos JPEG/syto12 staining - fig 2A - 1_rep - 14.5.23 jpeg/n2+tfg112.jpg]

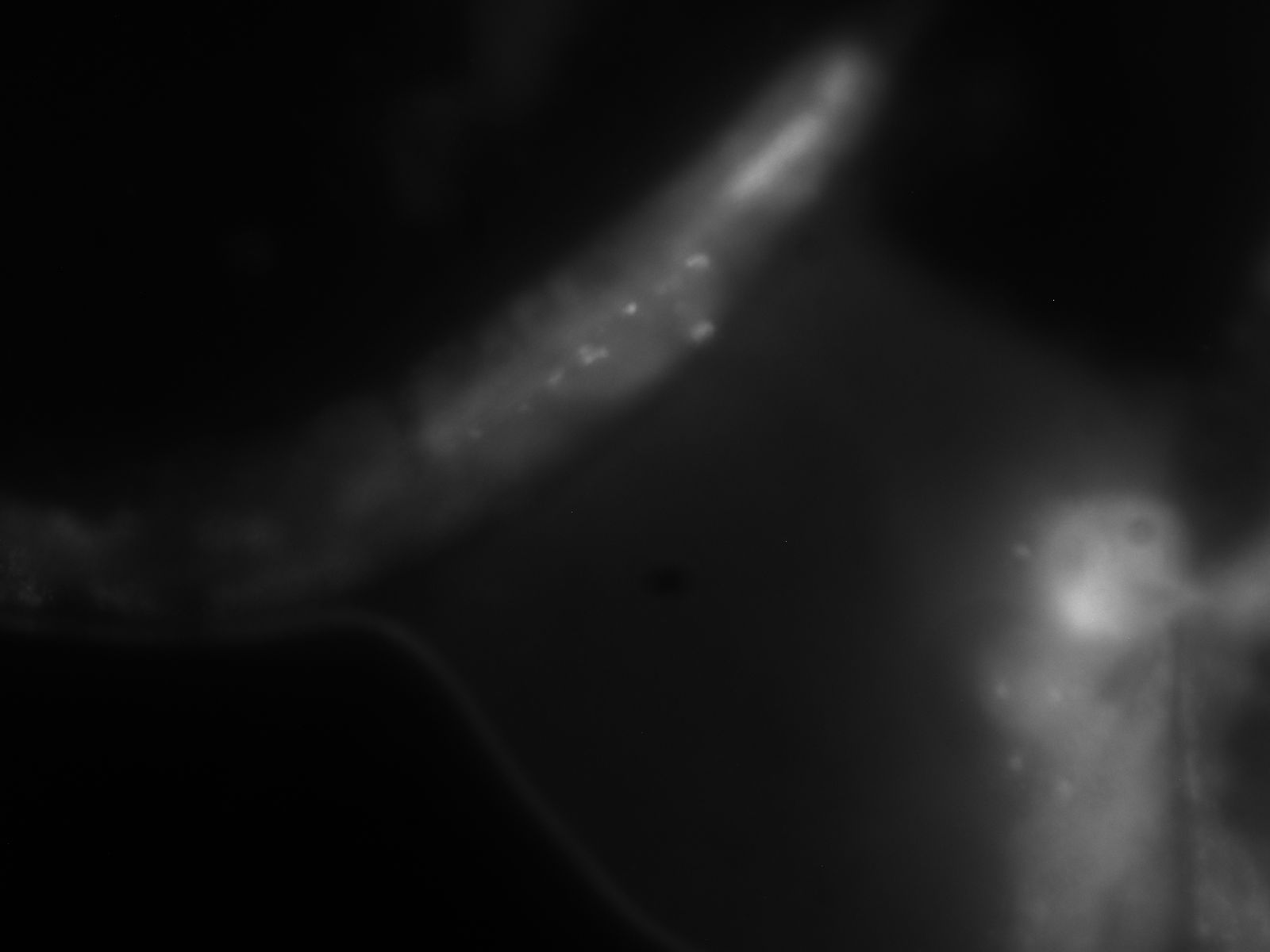

Supplement: S2 File — (ZIP) [file pgen.1011061.s002.zip › Fig.2A - Original files/Fig.2A RAW data and photos JPEG/syto12 staining - fig 2A - 1_rep - 14.5.23 jpeg/n2+tfg113.jpg]

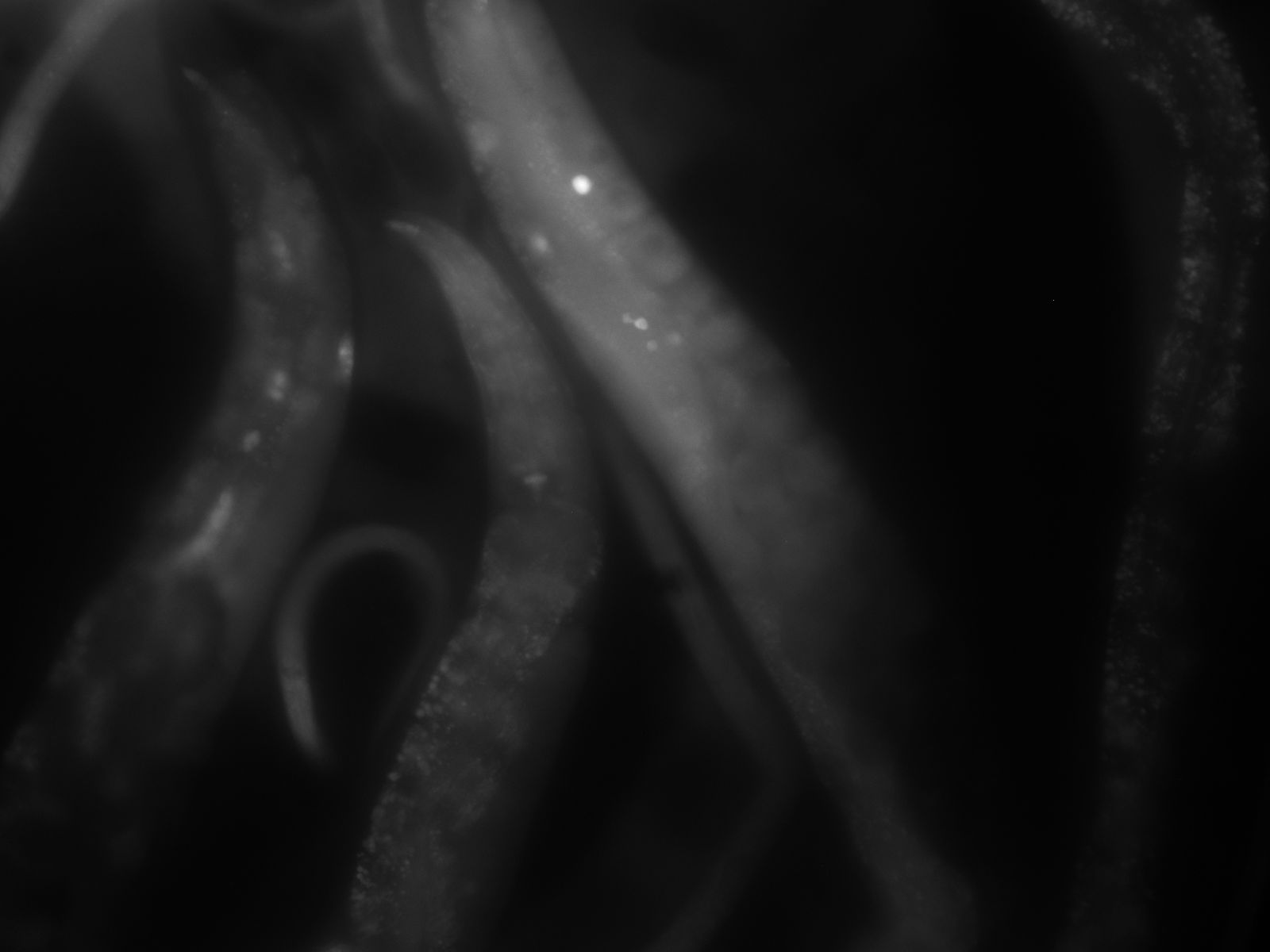

Supplement: S2 File — (ZIP) [file pgen.1011061.s002.zip › Fig.2A - Original files/Fig.2A RAW data and photos JPEG/syto12 staining - fig 2A - 1_rep - 14.5.23 jpeg/n2+tfg114.jpg]

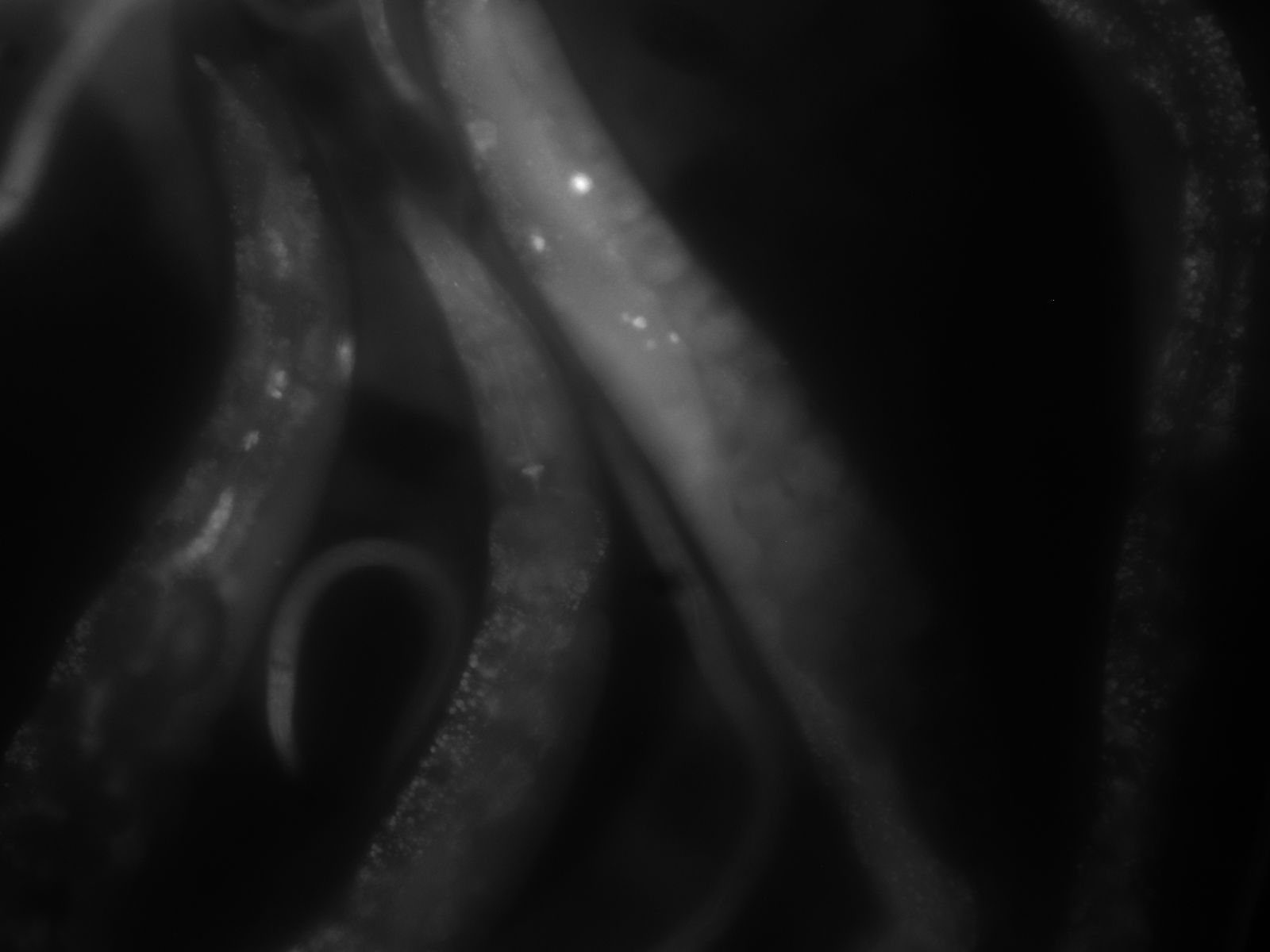

Supplement: S2 File — (ZIP) [file pgen.1011061.s002.zip › Fig.2A - Original files/Fig.2A RAW data and photos JPEG/syto12 staining - fig 2A - 1_rep - 14.5.23 jpeg/n2+tfg115.jpg]

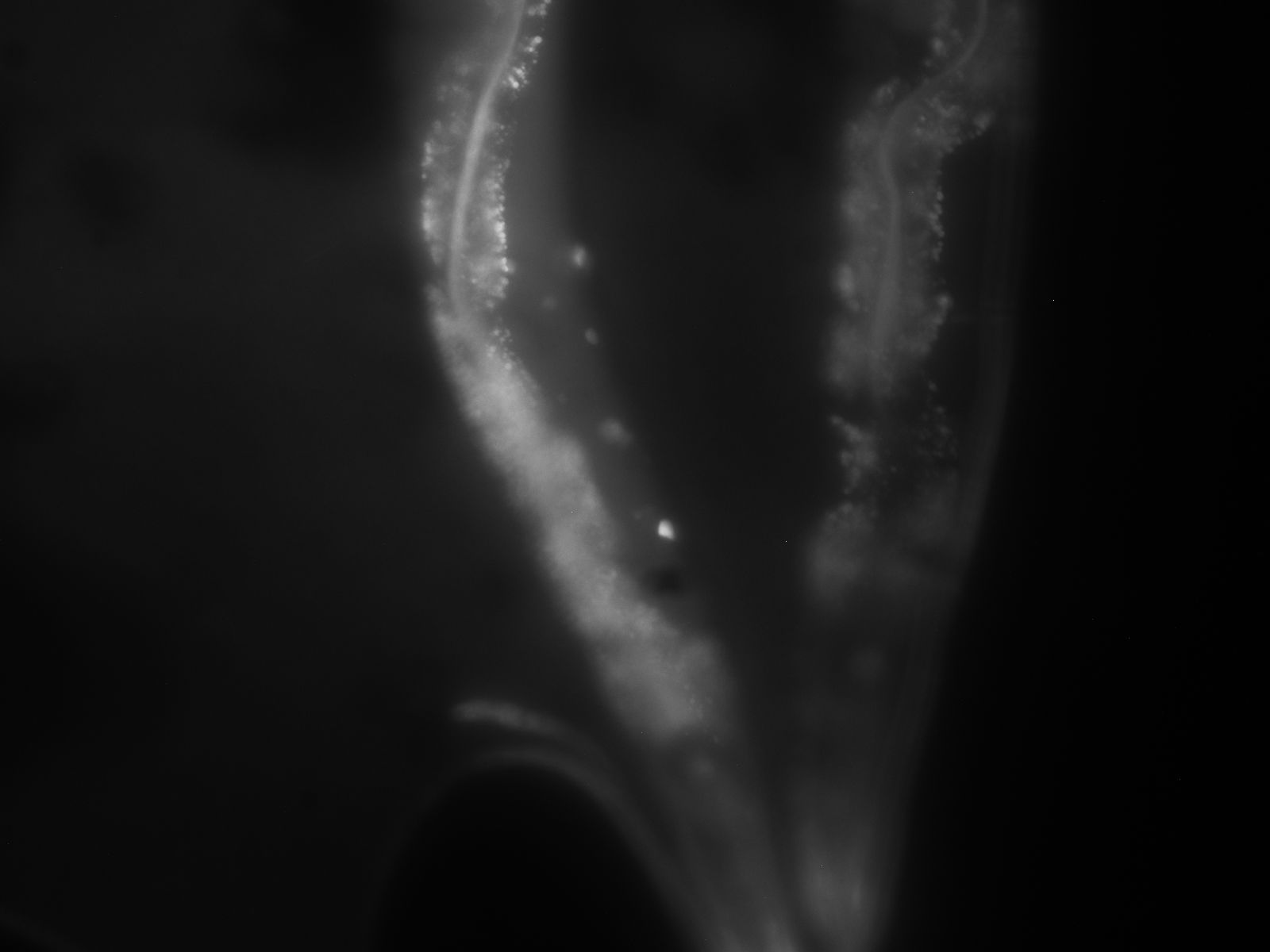

Supplement: S2 File — (ZIP) [file pgen.1011061.s002.zip › Fig.2A - Original files/Fig.2A RAW data and photos JPEG/syto12 staining - fig 2A - 1_rep - 14.5.23 jpeg/n2+tfg116.jpg]

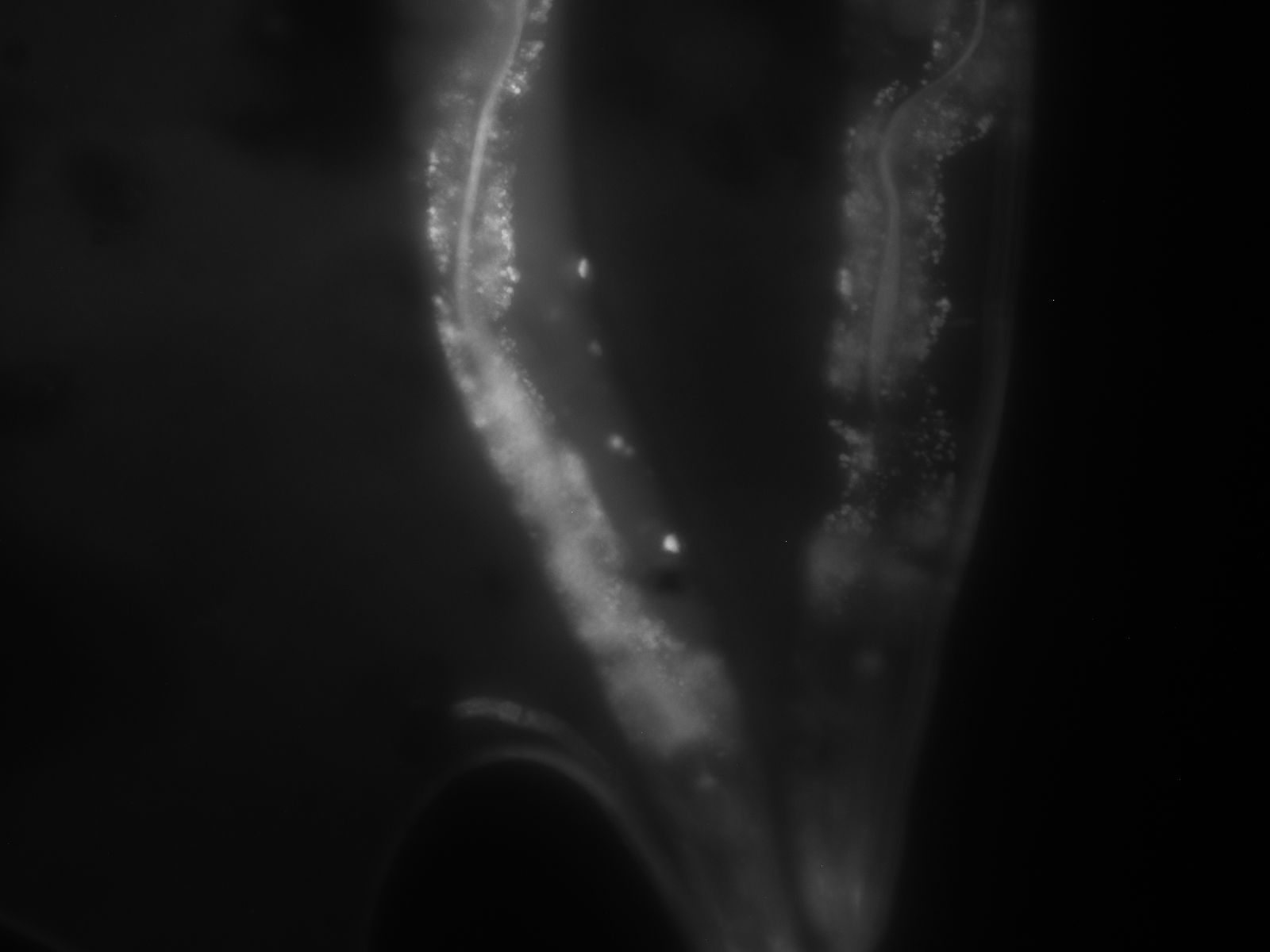

Supplement: S2 File — (ZIP) [file pgen.1011061.s002.zip › Fig.2A - Original files/Fig.2A RAW data and photos JPEG/syto12 staining - fig 2A - 1_rep - 14.5.23 jpeg/n2+tfg117.jpg]

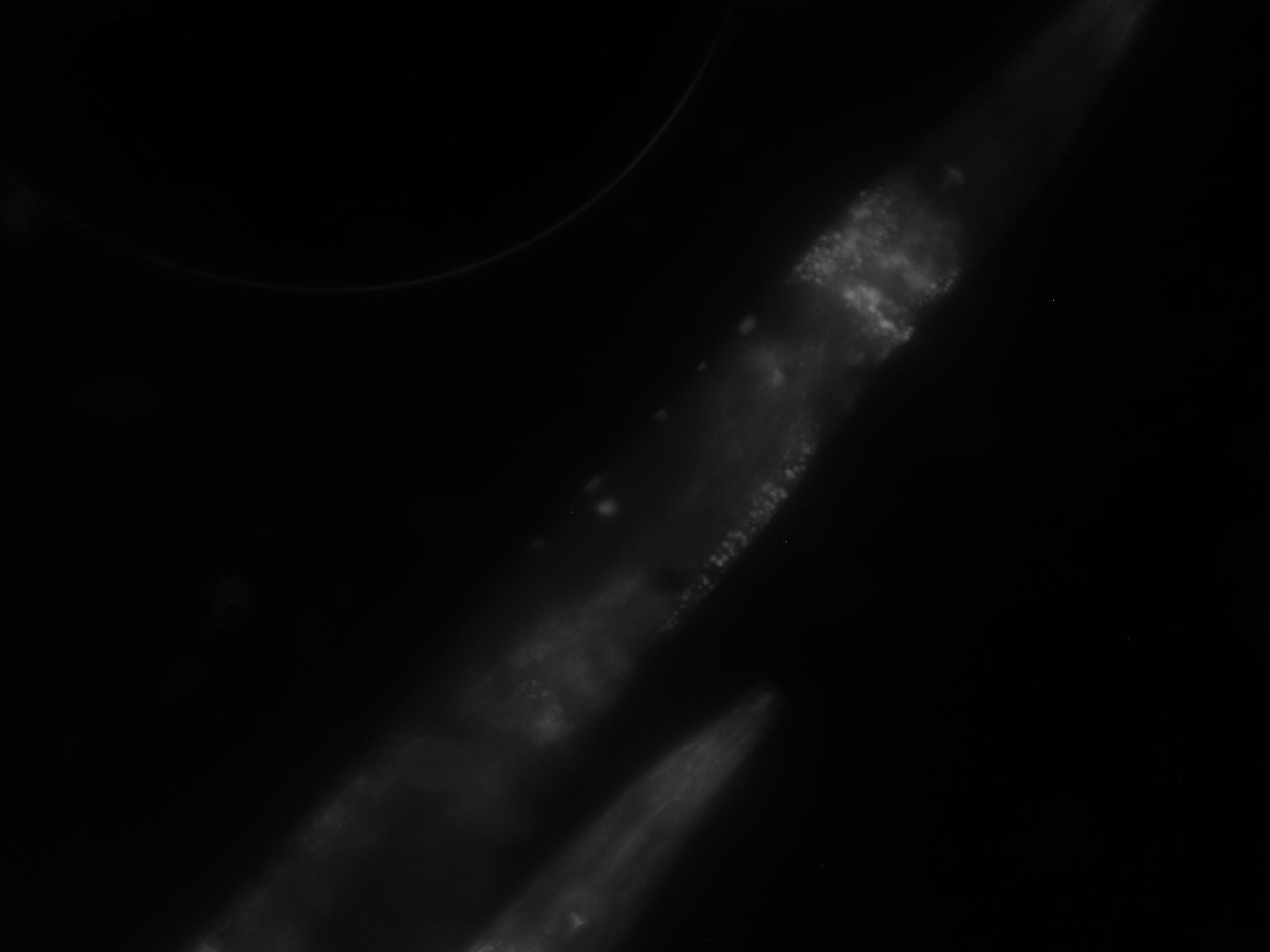

Supplement: S2 File — (ZIP) [file pgen.1011061.s002.zip › Fig.2A - Original files/Fig.2A RAW data and photos JPEG/syto12 staining - fig 2A - 1_rep - 14.5.23 jpeg/n2+tfg118.jpg]

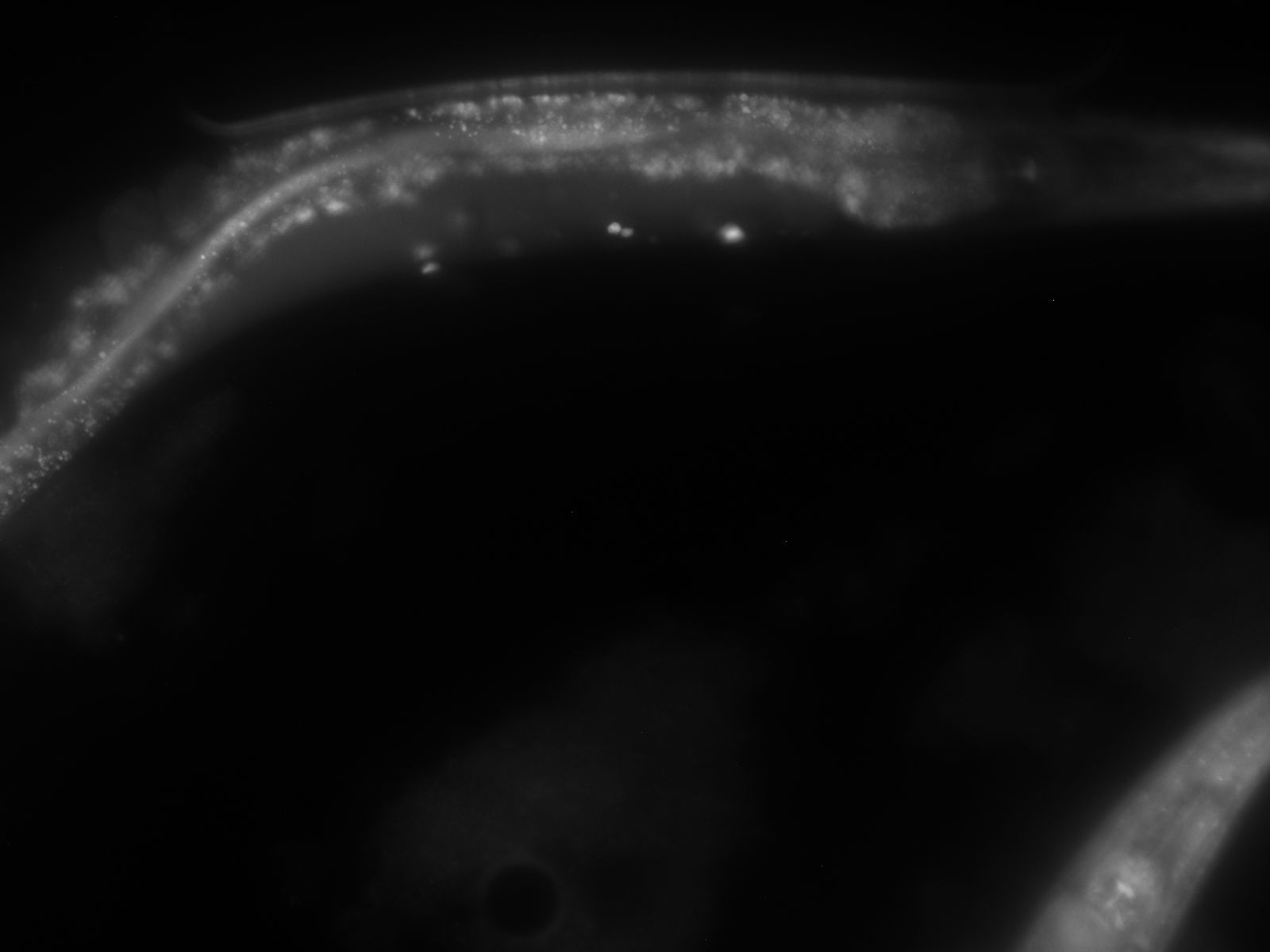

Supplement: S2 File — (ZIP) [file pgen.1011061.s002.zip › Fig.2A - Original files/Fig.2A RAW data and photos JPEG/syto12 staining - fig 2A - 1_rep - 14.5.23 jpeg/n2+tfg119.jpg]

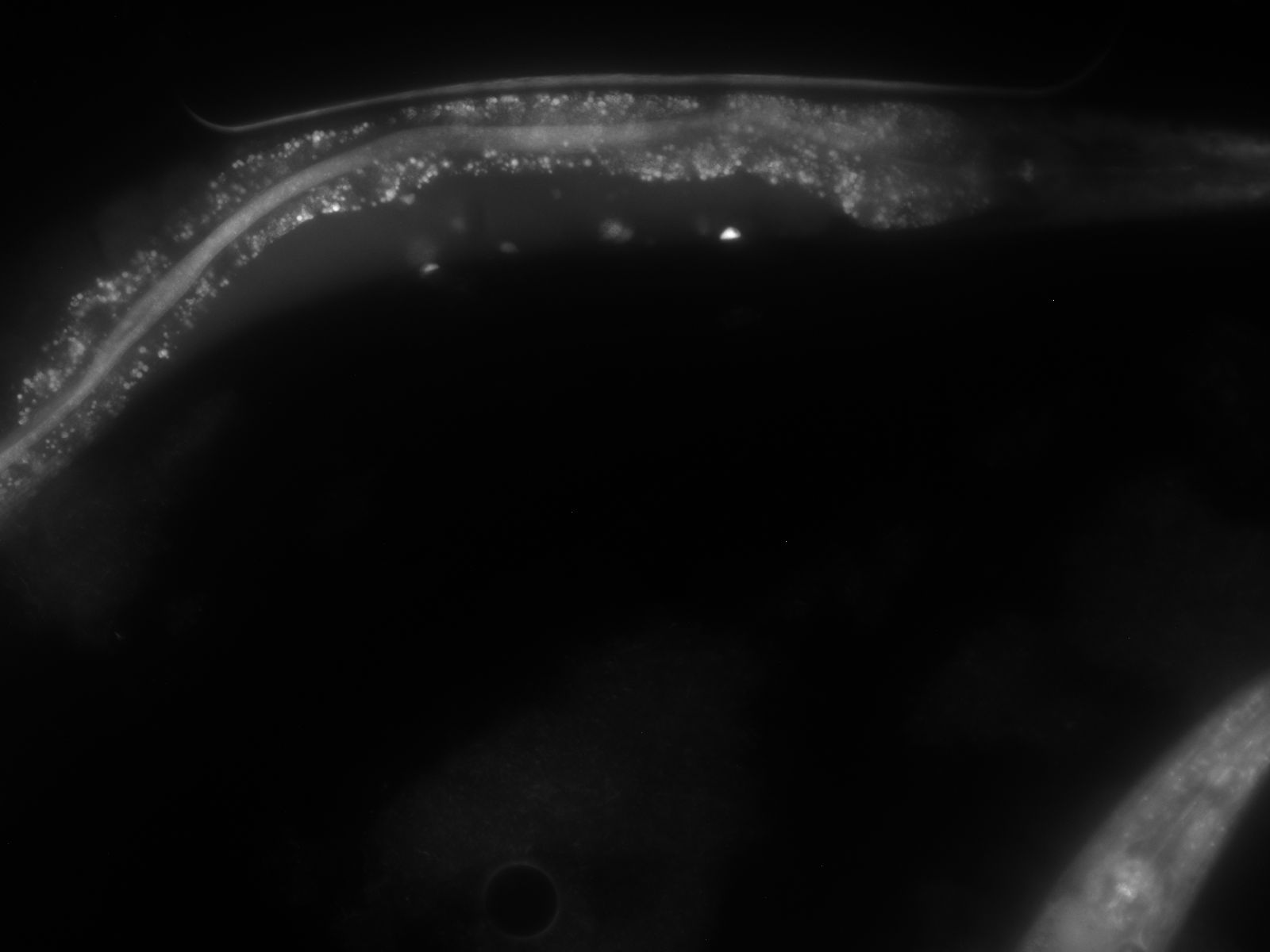

Supplement: S2 File — (ZIP) [file pgen.1011061.s002.zip › Fig.2A - Original files/Fig.2A RAW data and photos JPEG/syto12 staining - fig 2A - 1_rep - 14.5.23 jpeg/n2+tfg120.jpg]

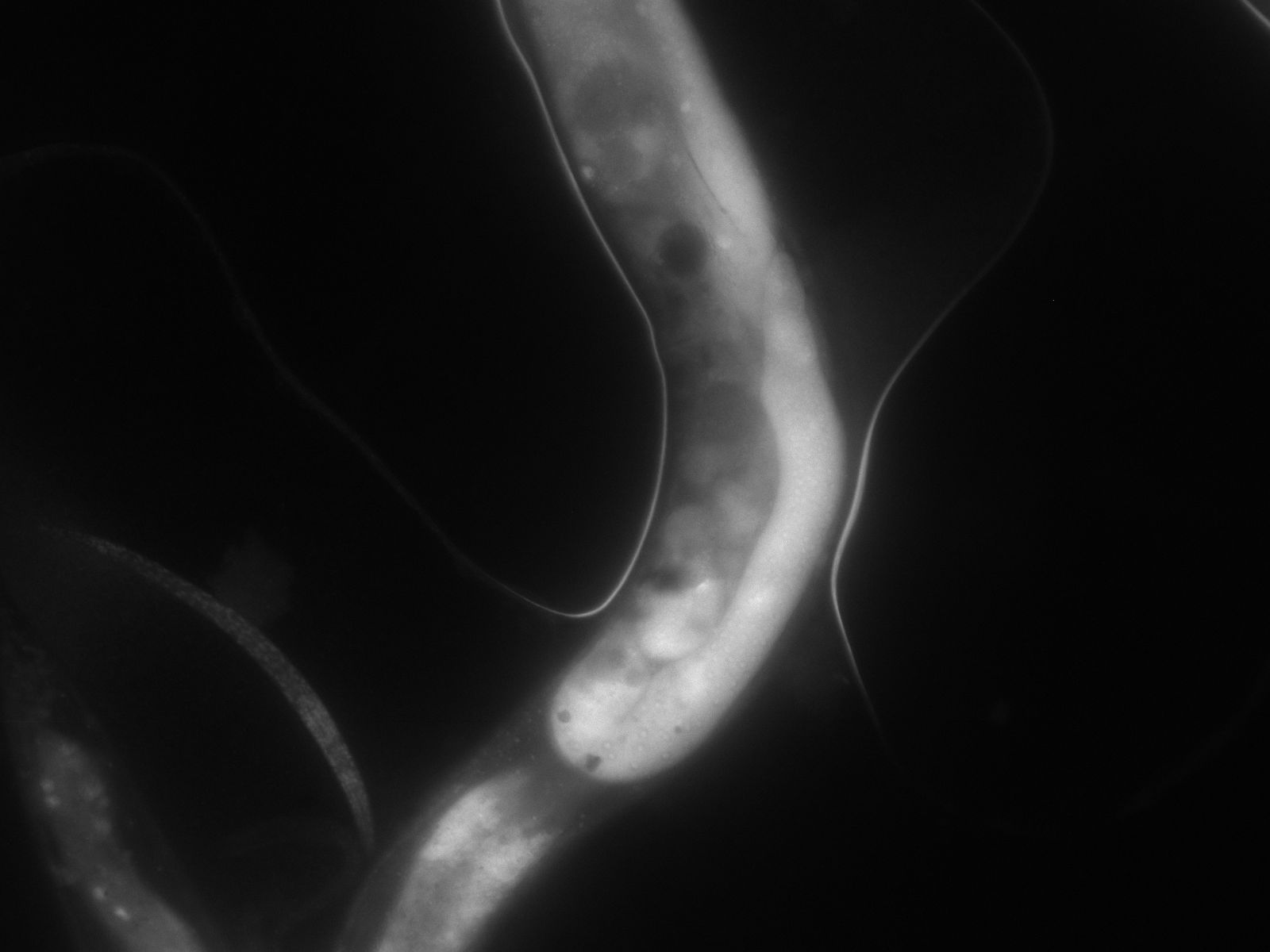

Supplement: S2 File — (ZIP) [file pgen.1011061.s002.zip › Fig.2A - Original files/Fig.2A RAW data and photos JPEG/syto12 staining - fig 2A - 1_rep - 14.5.23 jpeg/xbp-1_ire-1+pad12154.jpg]

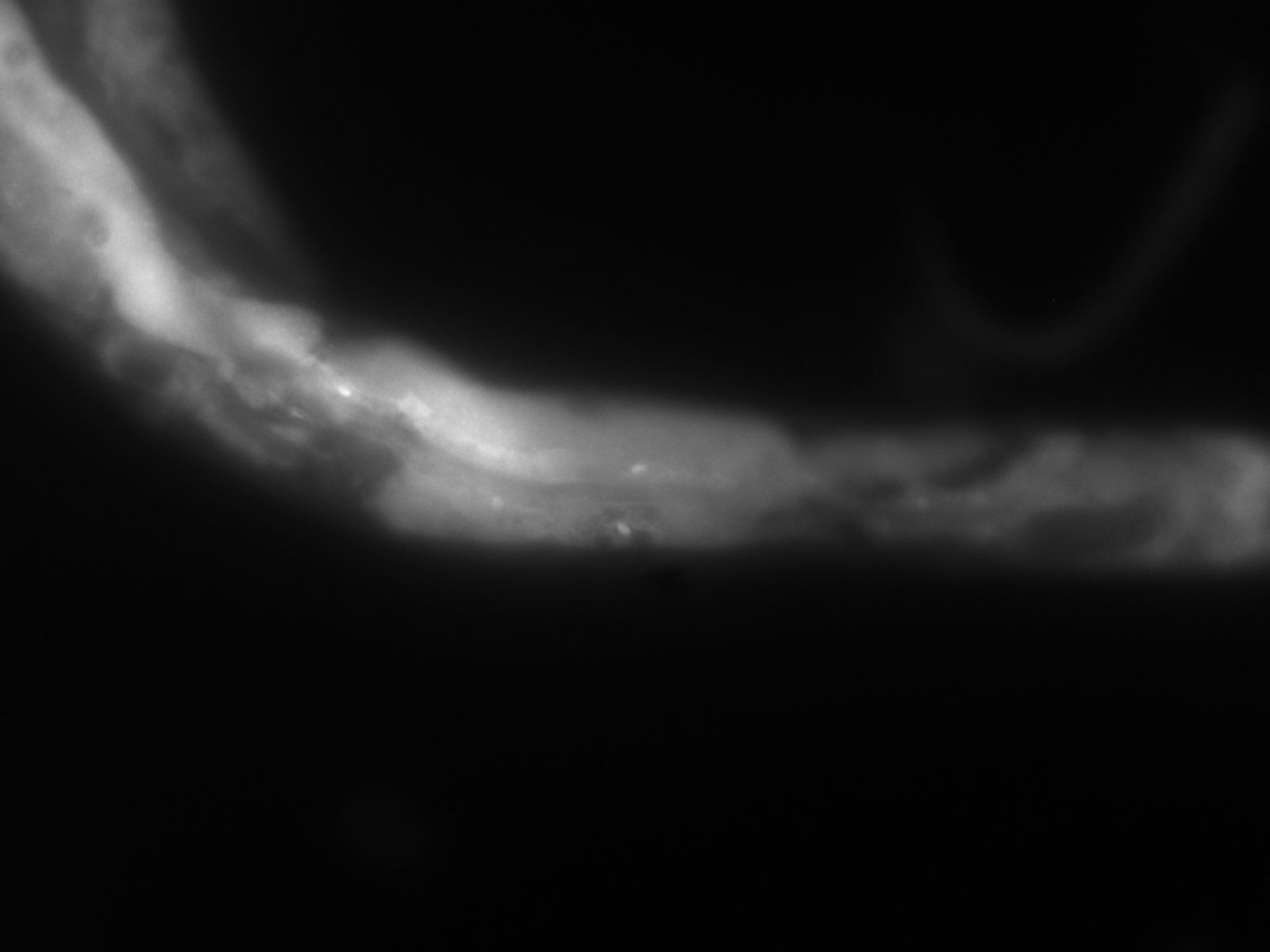

Supplement: S2 File — (ZIP) [file pgen.1011061.s002.zip › Fig.2A - Original files/Fig.2A RAW data and photos JPEG/syto12 staining - fig 2A - 1_rep - 14.5.23 jpeg/xbp-1_ire-1+pad12155.jpg]

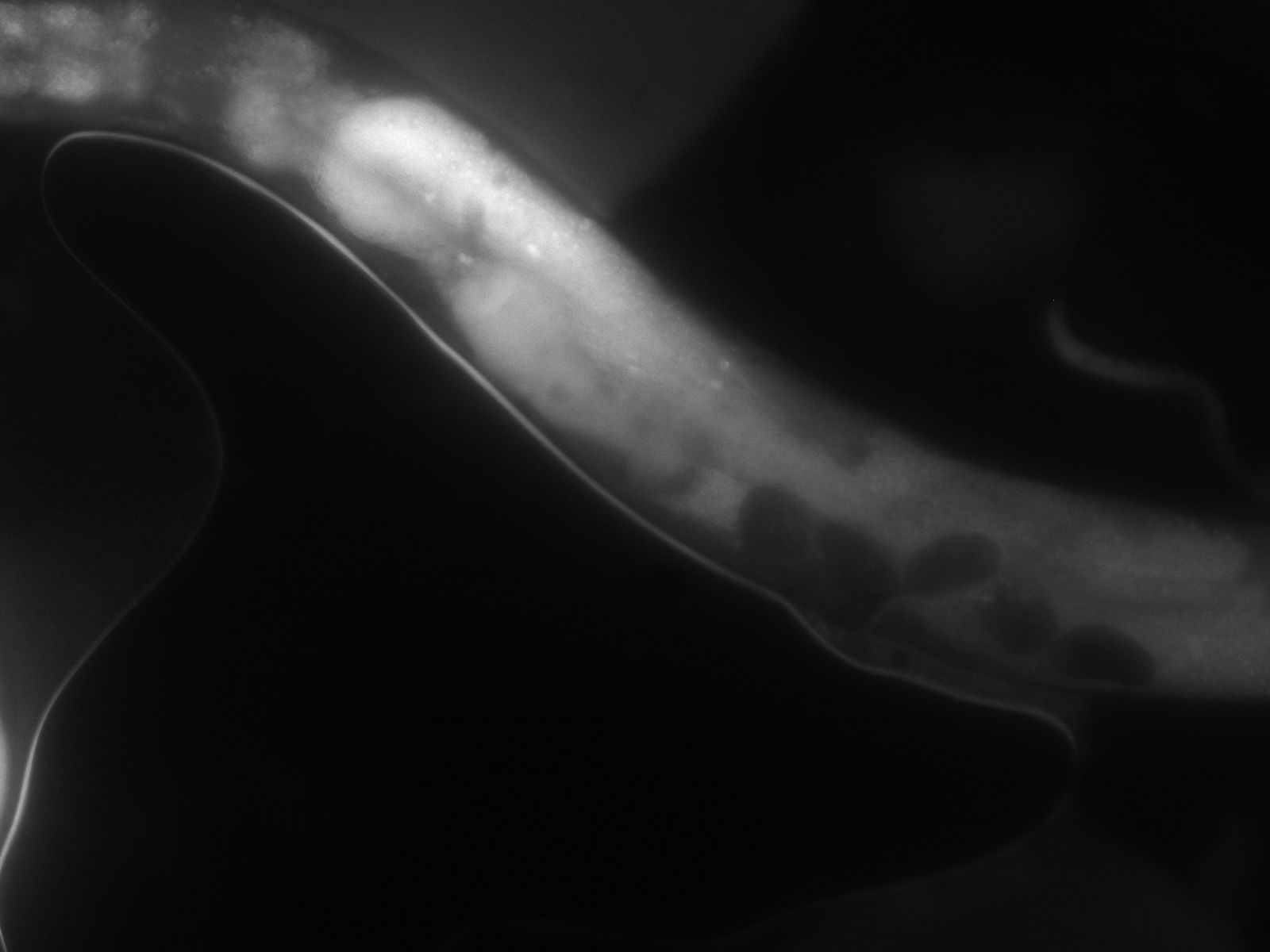

Supplement: S2 File — (ZIP) [file pgen.1011061.s002.zip › Fig.2A - Original files/Fig.2A RAW data and photos JPEG/syto12 staining - fig 2A - 1_rep - 14.5.23 jpeg/xbp-1_ire-1+pad12156.jpg]

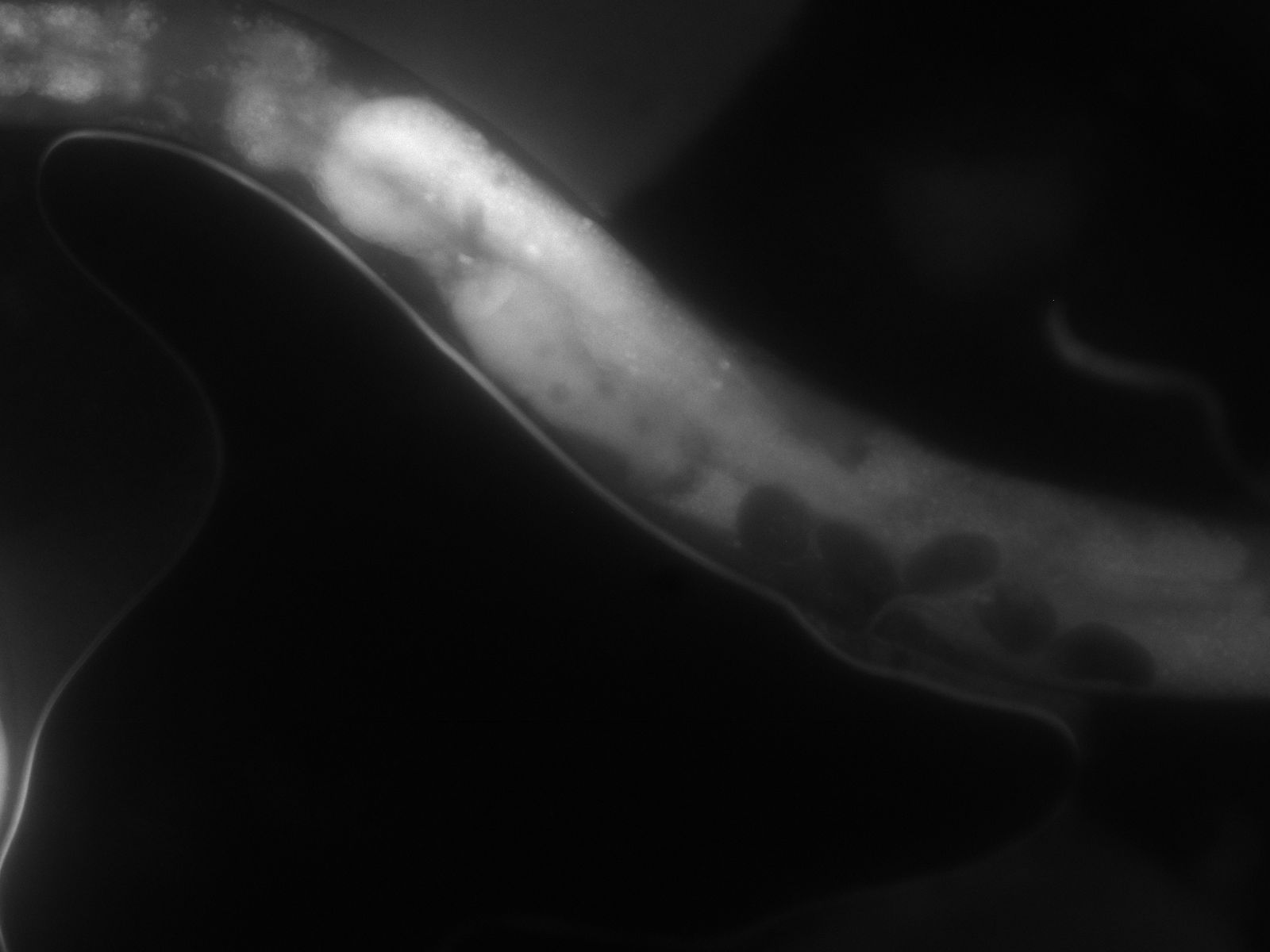

Supplement: S2 File — (ZIP) [file pgen.1011061.s002.zip › Fig.2A - Original files/Fig.2A RAW data and photos JPEG/syto12 staining - fig 2A - 1_rep - 14.5.23 jpeg/xbp-1_ire-1+pad12157.jpg]

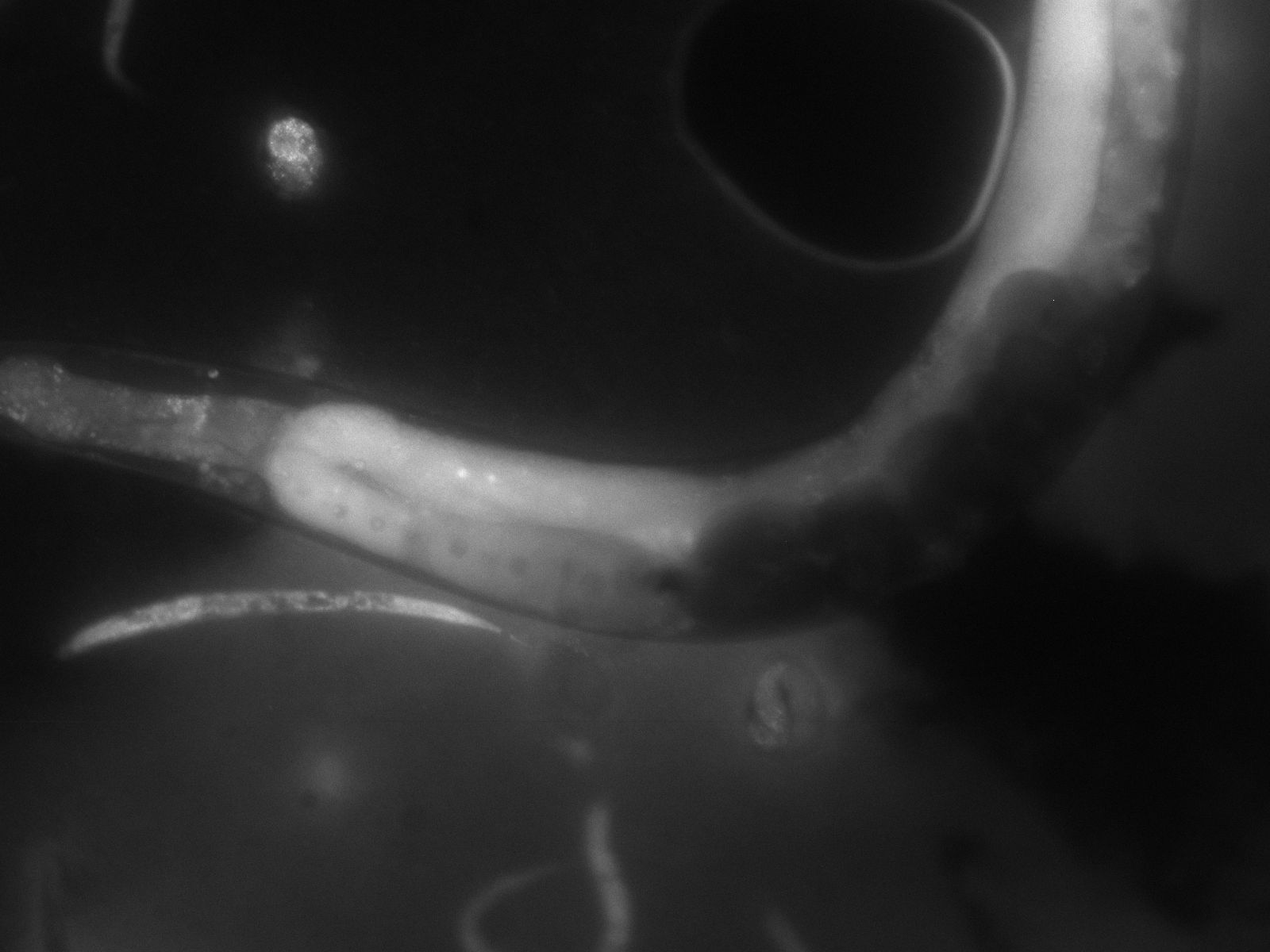

Supplement: S2 File — (ZIP) [file pgen.1011061.s002.zip › Fig.2A - Original files/Fig.2A RAW data and photos JPEG/syto12 staining - fig 2A - 1_rep - 14.5.23 jpeg/xbp-1_ire-1+pad12158.jpg]

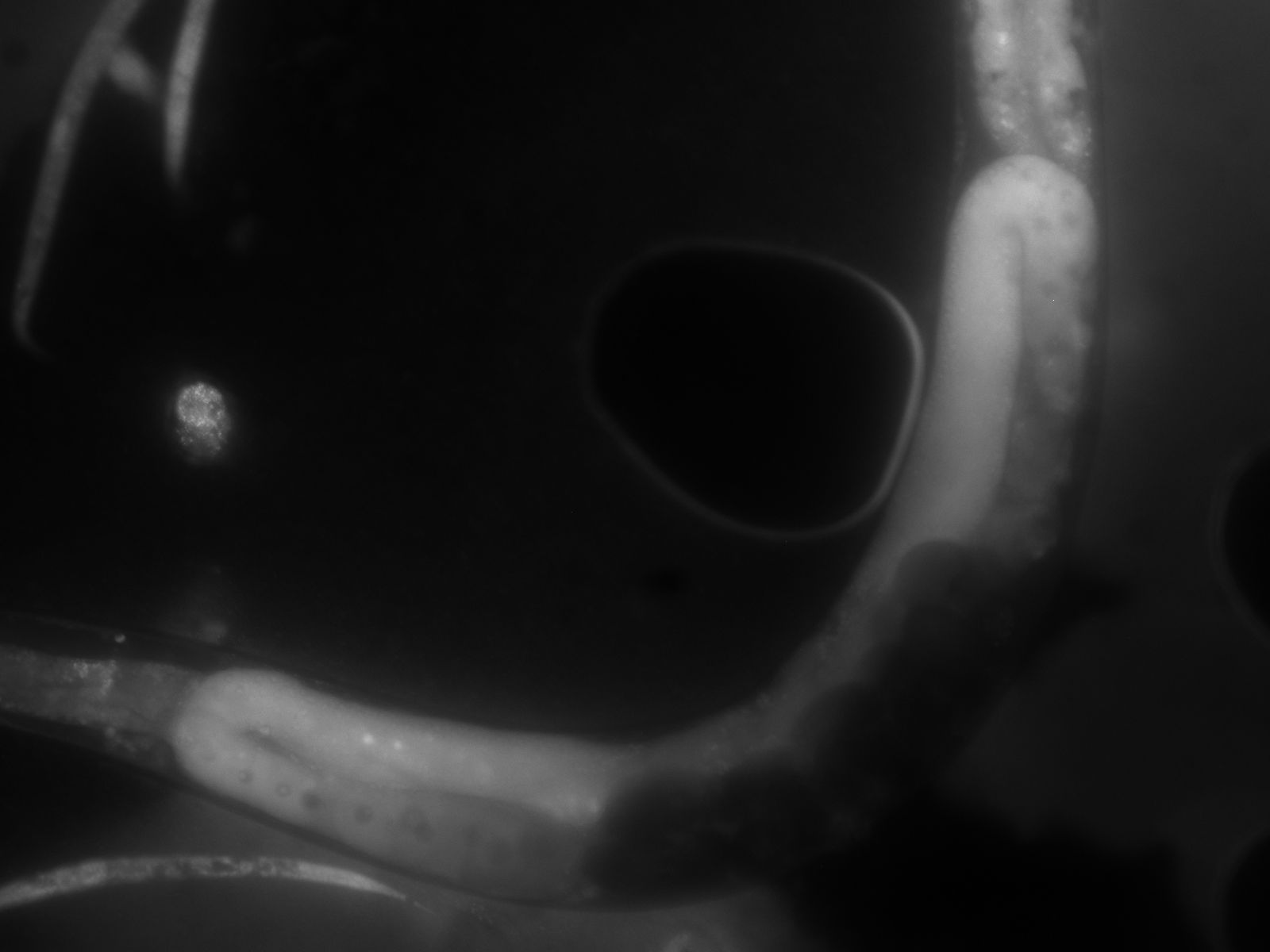

Supplement: S2 File — (ZIP) [file pgen.1011061.s002.zip › Fig.2A - Original files/Fig.2A RAW data and photos JPEG/syto12 staining - fig 2A - 1_rep - 14.5.23 jpeg/xbp-1_ire-1+pad12159.jpg]

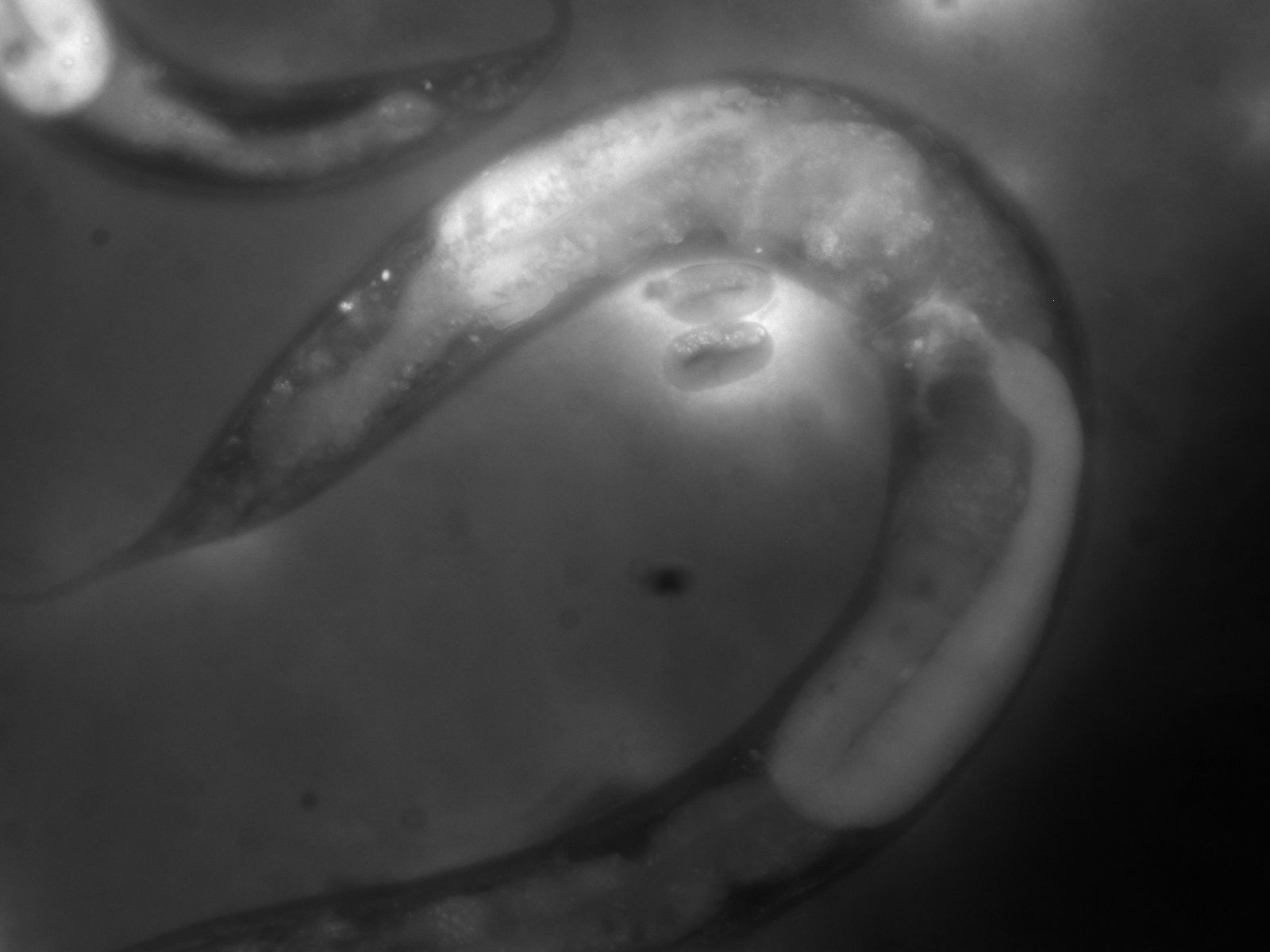

Supplement: S2 File — (ZIP) [file pgen.1011061.s002.zip › Fig.2A - Original files/Fig.2A RAW data and photos JPEG/syto12 staining - fig 2A - 1_rep - 14.5.23 jpeg/xbp-1_ire-1+pad12160.jpg]

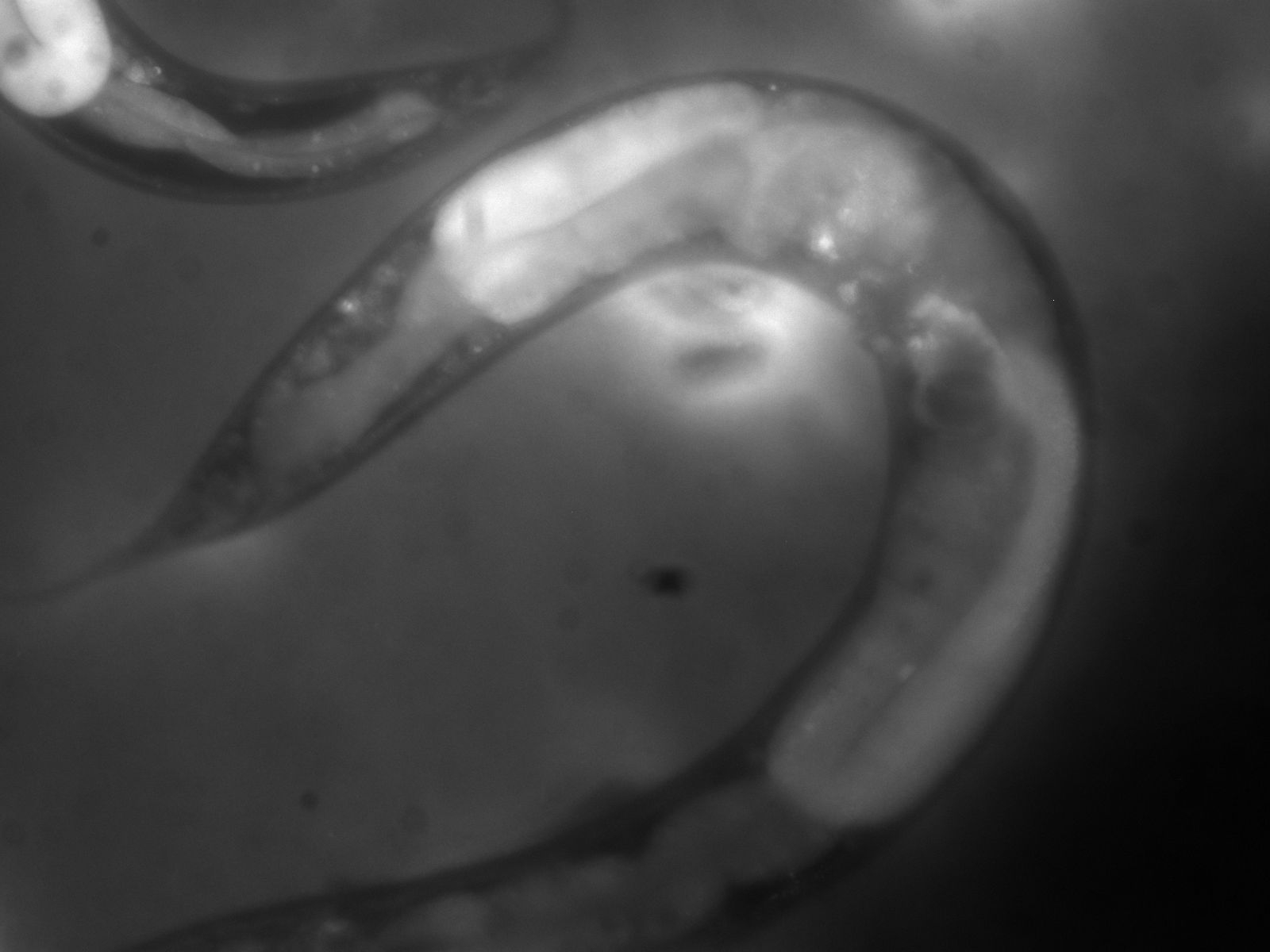

Supplement: S2 File — (ZIP) [file pgen.1011061.s002.zip › Fig.2A - Original files/Fig.2A RAW data and photos JPEG/syto12 staining - fig 2A - 1_rep - 14.5.23 jpeg/xbp-1_ire-1+pad12161.jpg]

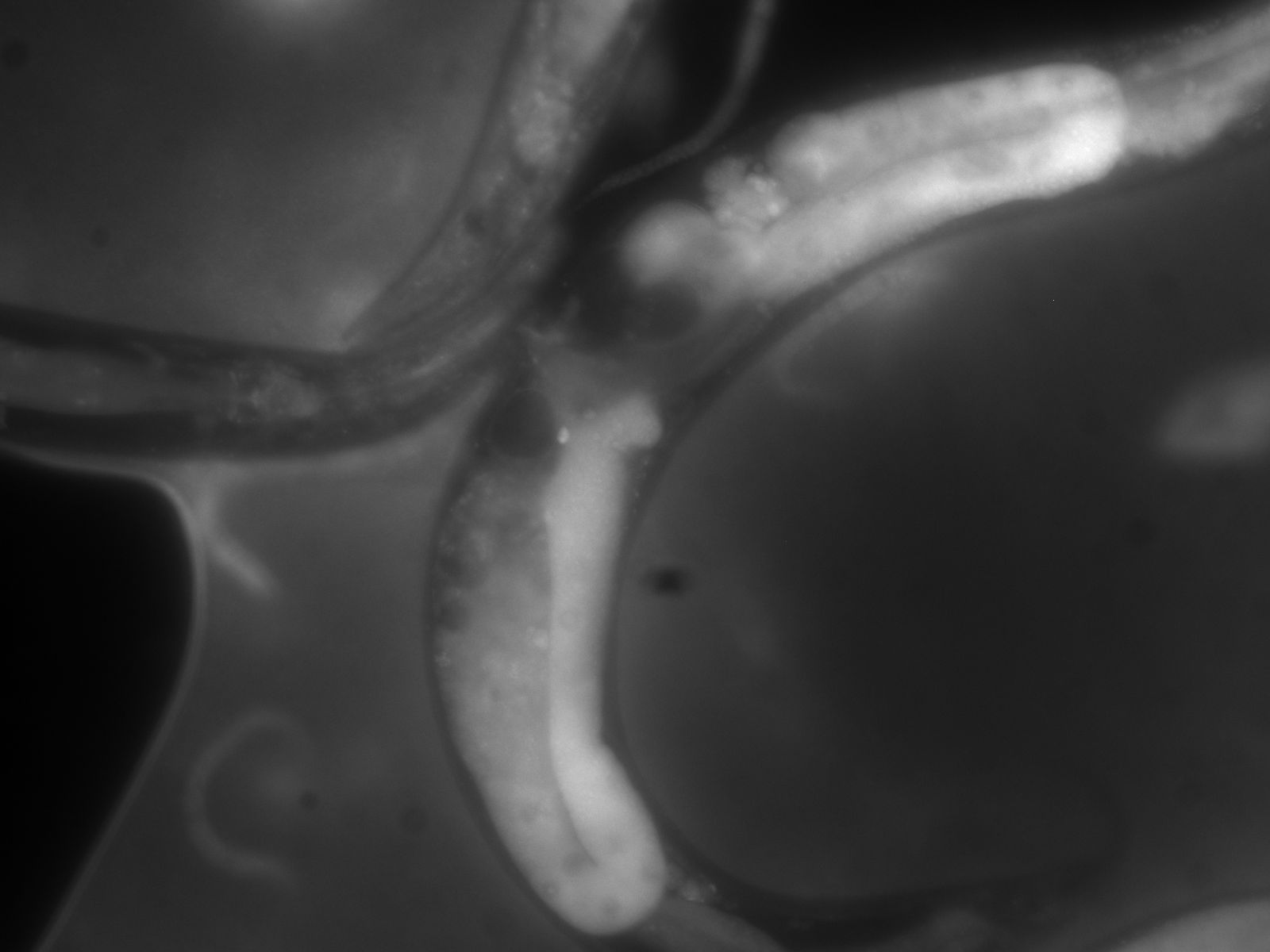

Supplement: S2 File — (ZIP) [file pgen.1011061.s002.zip › Fig.2A - Original files/Fig.2A RAW data and photos JPEG/syto12 staining - fig 2A - 1_rep - 14.5.23 jpeg/xbp-1_ire-1+pad12162.jpg]

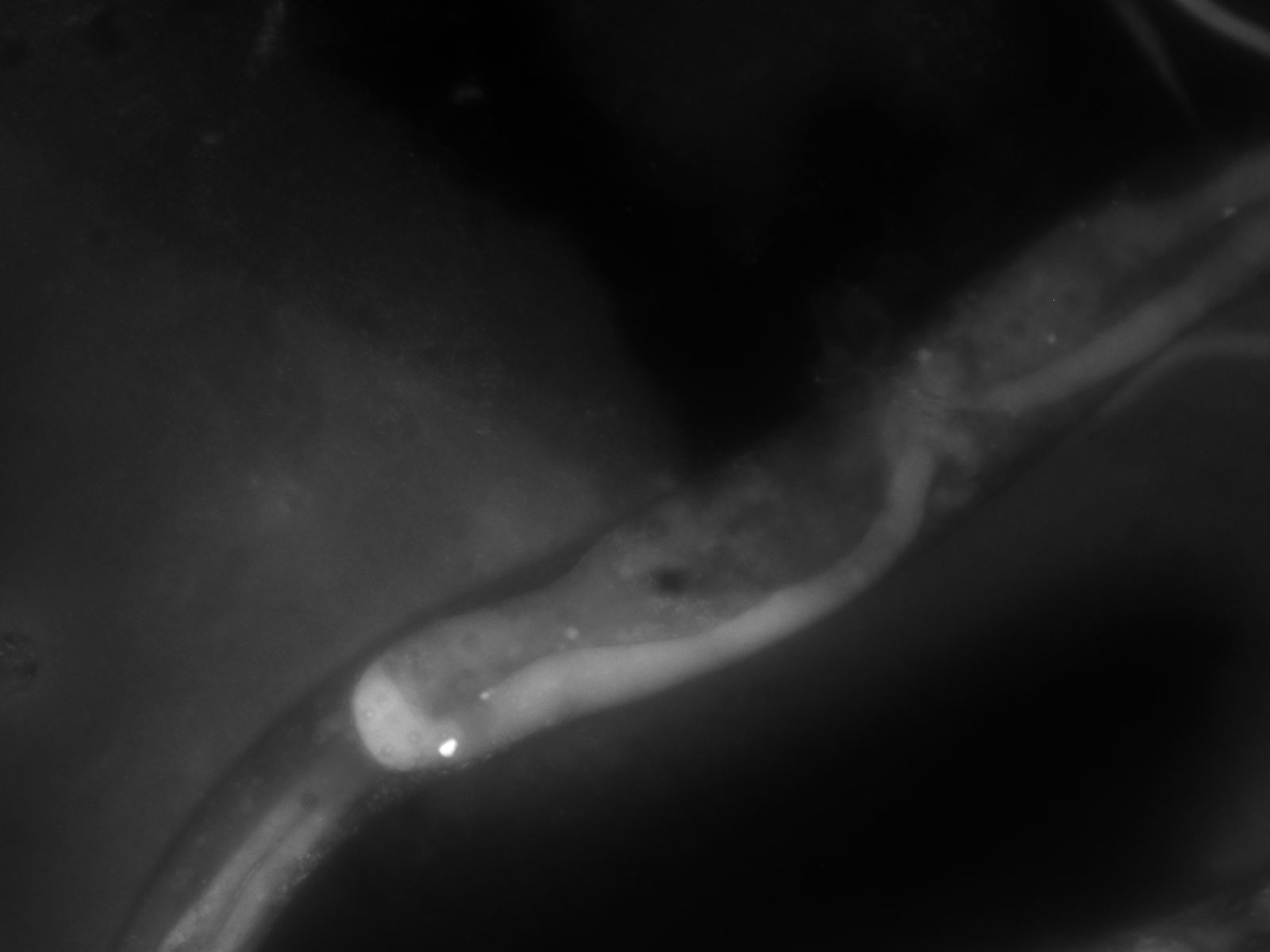

Supplement: S2 File — (ZIP) [file pgen.1011061.s002.zip › Fig.2A - Original files/Fig.2A RAW data and photos JPEG/syto12 staining - fig 2A - 1_rep - 14.5.23 jpeg/xbp-1_ire-1+pad12163.jpg]

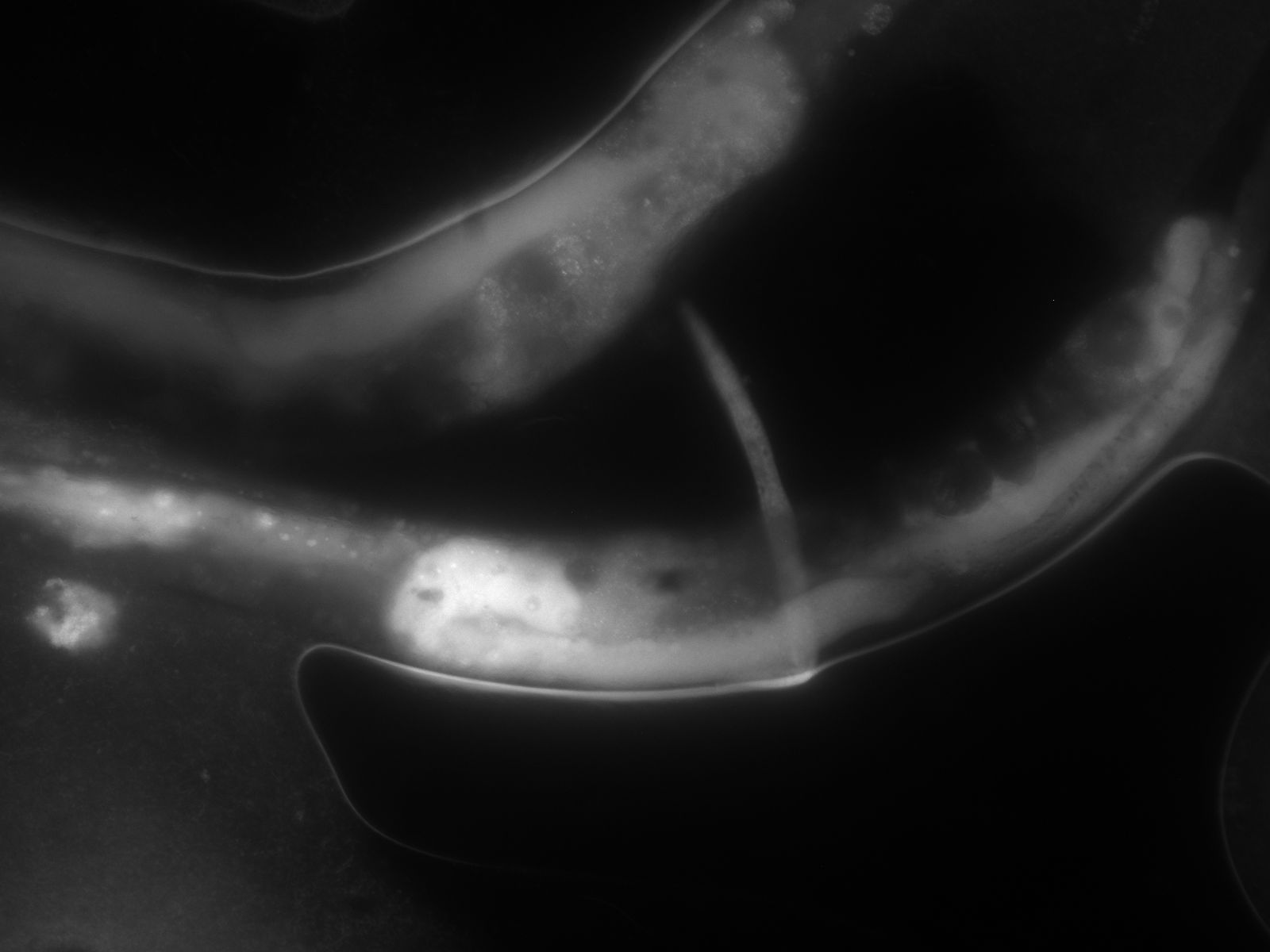

Supplement: S2 File — (ZIP) [file pgen.1011061.s002.zip › Fig.2A - Original files/Fig.2A RAW data and photos JPEG/syto12 staining - fig 2A - 1_rep - 14.5.23 jpeg/xbp-1_ire-1+pad12164.jpg]

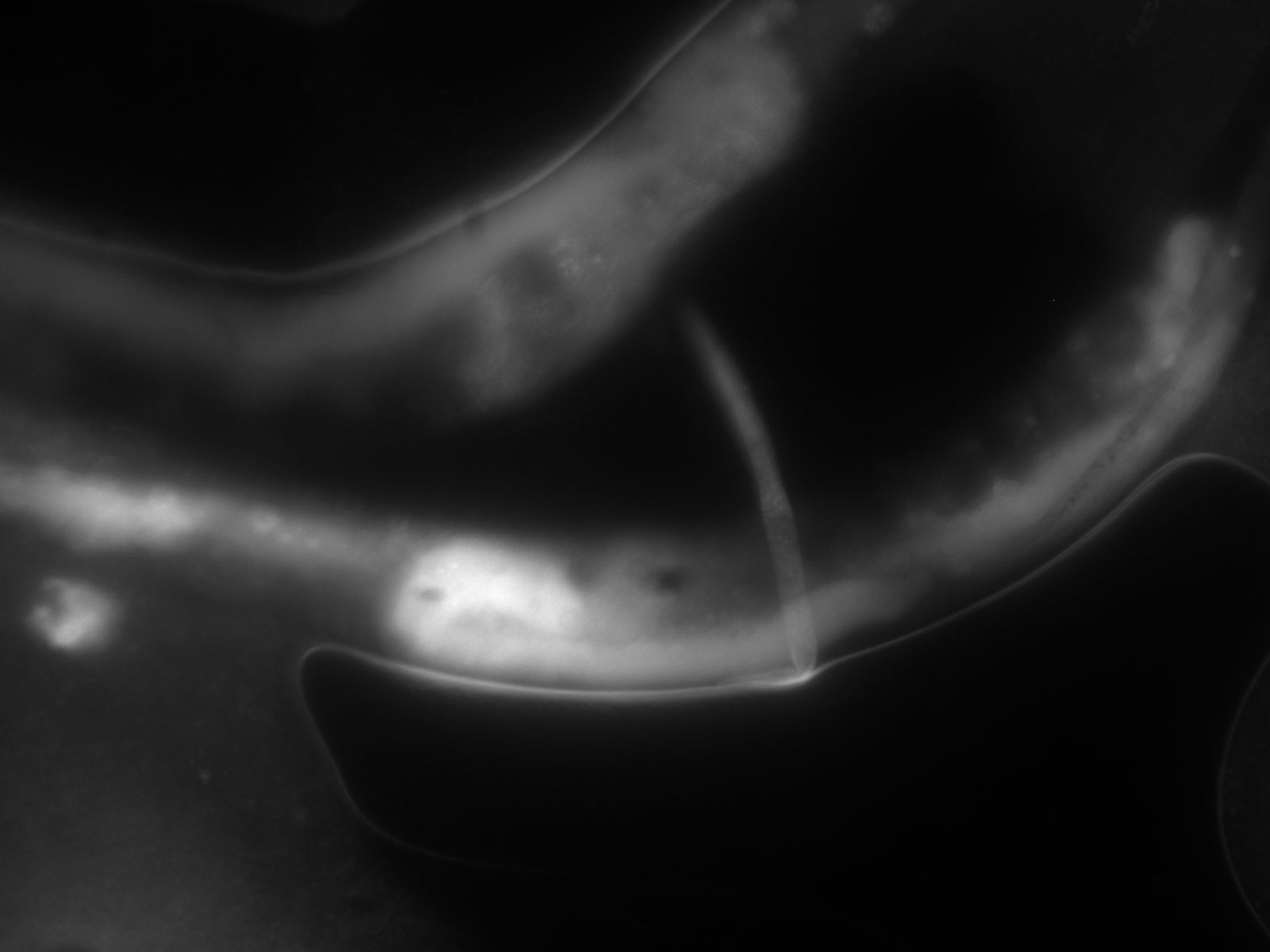

Supplement: S2 File — (ZIP) [file pgen.1011061.s002.zip › Fig.2A - Original files/Fig.2A RAW data and photos JPEG/syto12 staining - fig 2A - 1_rep - 14.5.23 jpeg/xbp-1_ire-1+pad12165.jpg]

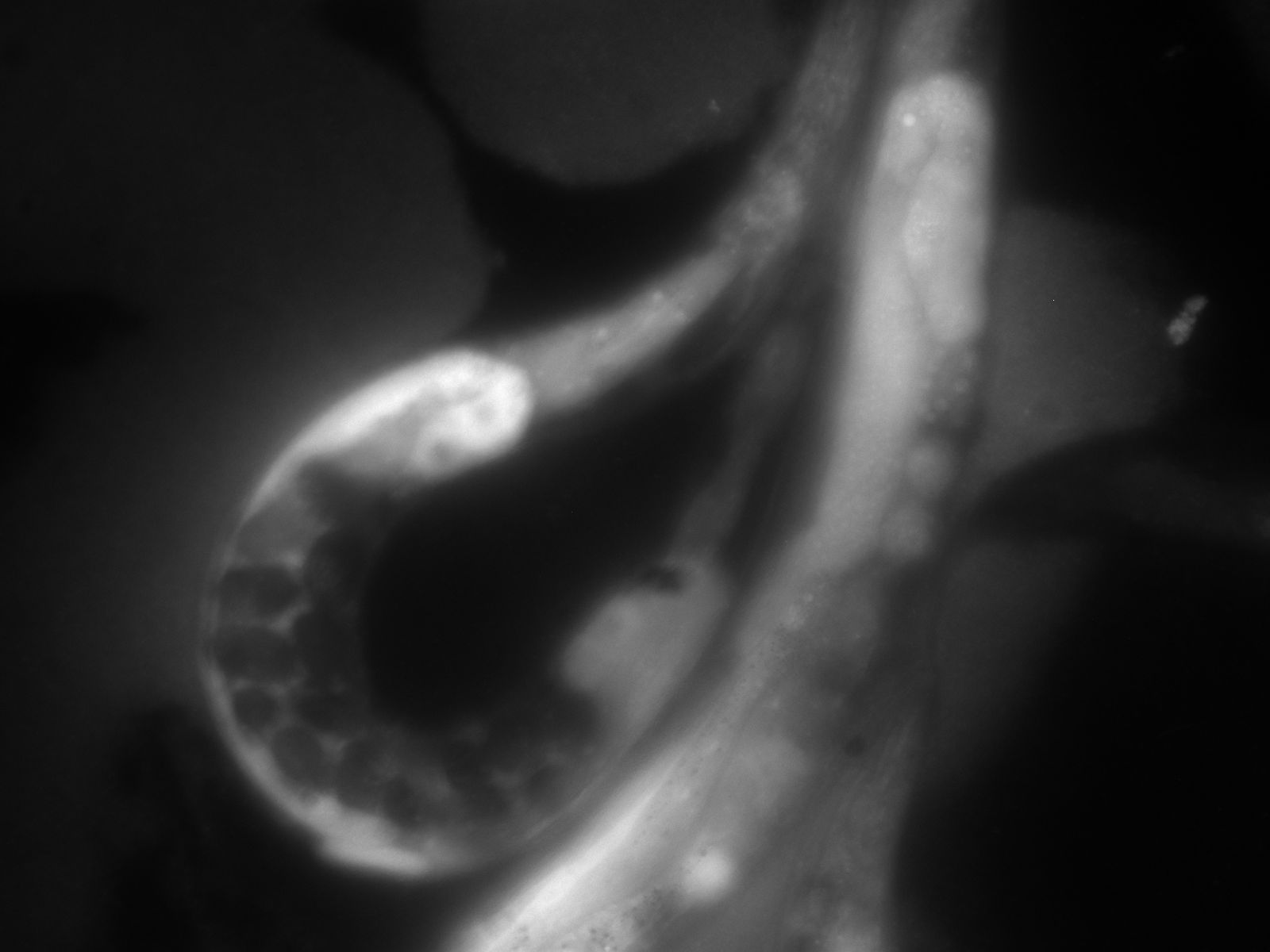

Supplement: S2 File — (ZIP) [file pgen.1011061.s002.zip › Fig.2A - Original files/Fig.2A RAW data and photos JPEG/syto12 staining - fig 2A - 1_rep - 14.5.23 jpeg/xbp-1_ire-1+pad12166.jpg]

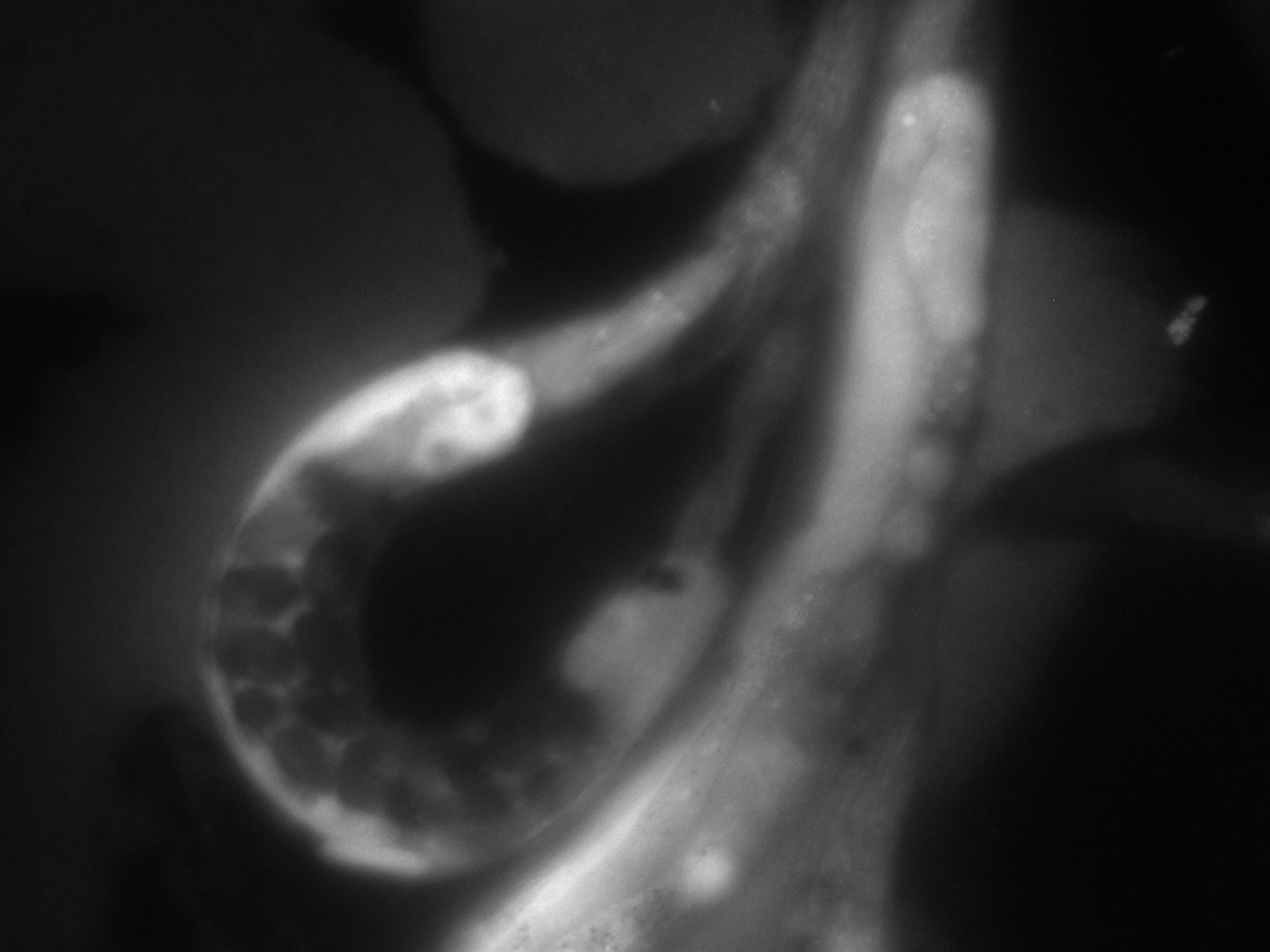

Supplement: S2 File — (ZIP) [file pgen.1011061.s002.zip › Fig.2A - Original files/Fig.2A RAW data and photos JPEG/syto12 staining - fig 2A - 1_rep - 14.5.23 jpeg/xbp-1_ire-1+pad12167.jpg]

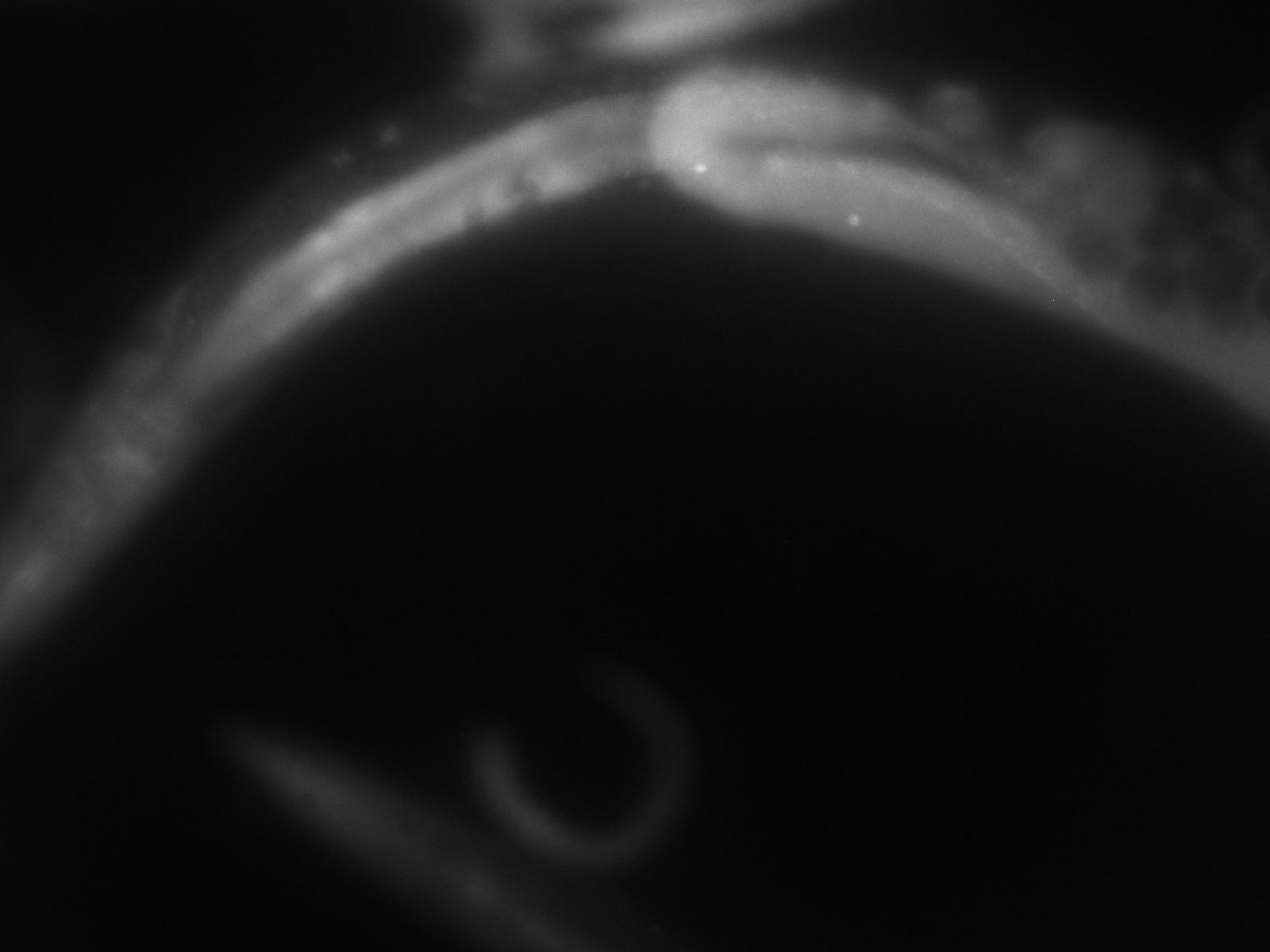

Supplement: S2 File — (ZIP) [file pgen.1011061.s002.zip › Fig.2A - Original files/Fig.2A RAW data and photos JPEG/syto12 staining - fig 2A - 1_rep - 14.5.23 jpeg/xbp-1_ire-1+pad12168.jpg]

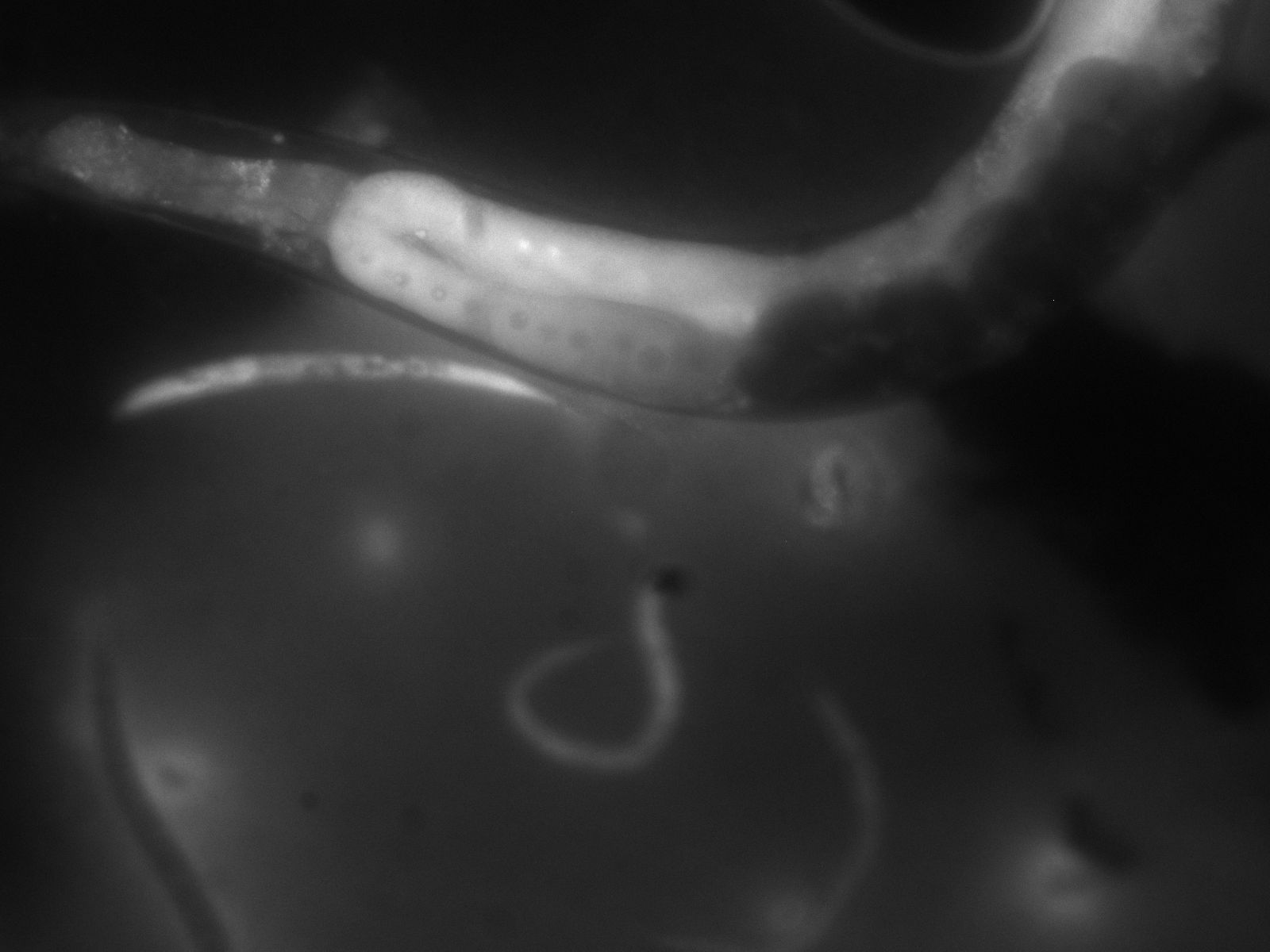

Supplement: S2 File — (ZIP) [file pgen.1011061.s002.zip › Fig.2A - Original files/Fig.2A RAW data and photos JPEG/syto12 staining - fig 2A - 1_rep - 14.5.23 jpeg/xbp-1_ire-1+pad12169.jpg]

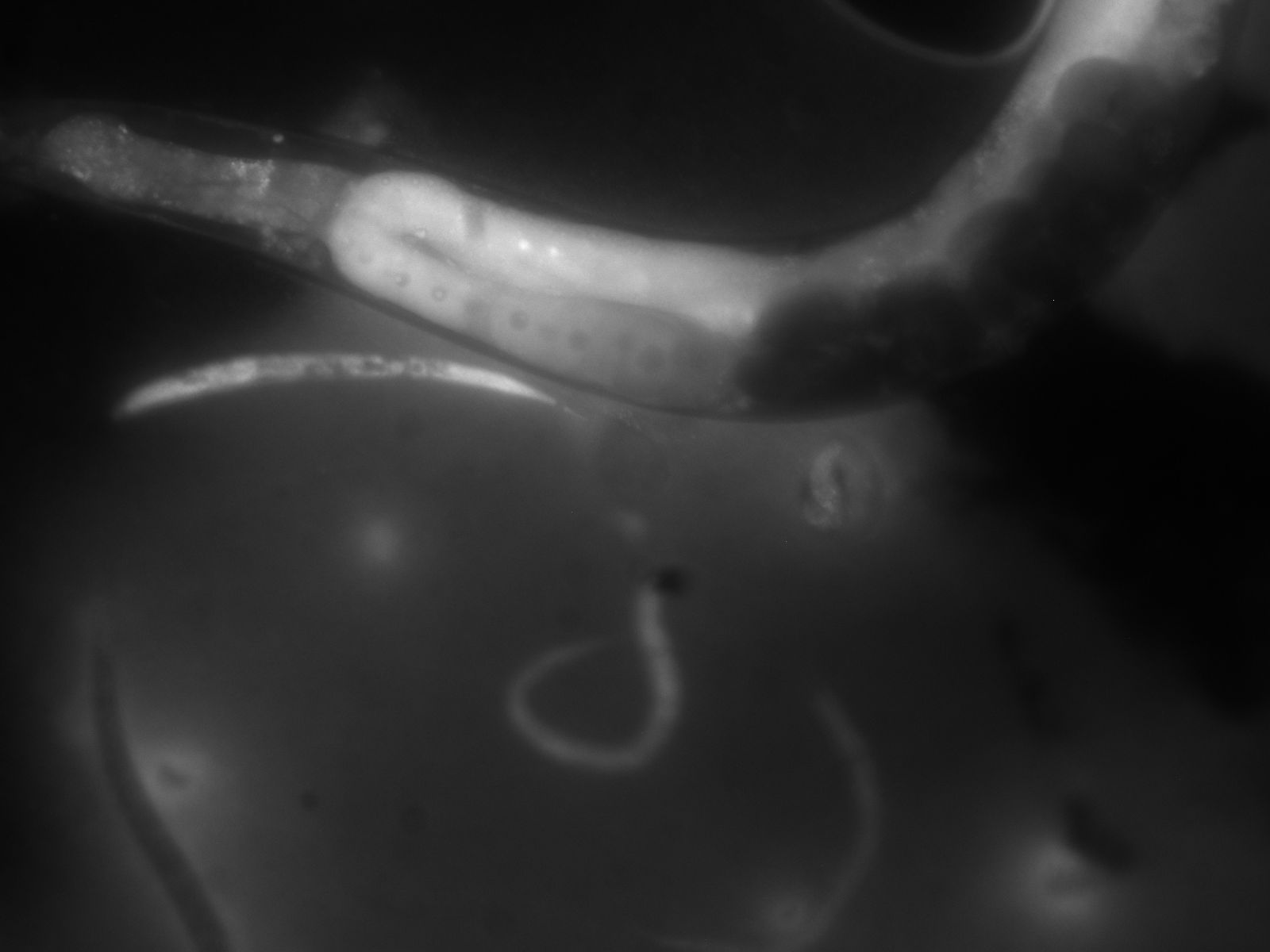

Supplement: S2 File — (ZIP) [file pgen.1011061.s002.zip › Fig.2A - Original files/Fig.2A RAW data and photos JPEG/syto12 staining - fig 2A - 1_rep - 14.5.23 jpeg/xbp-1_ire-1+pad12170.jpg]

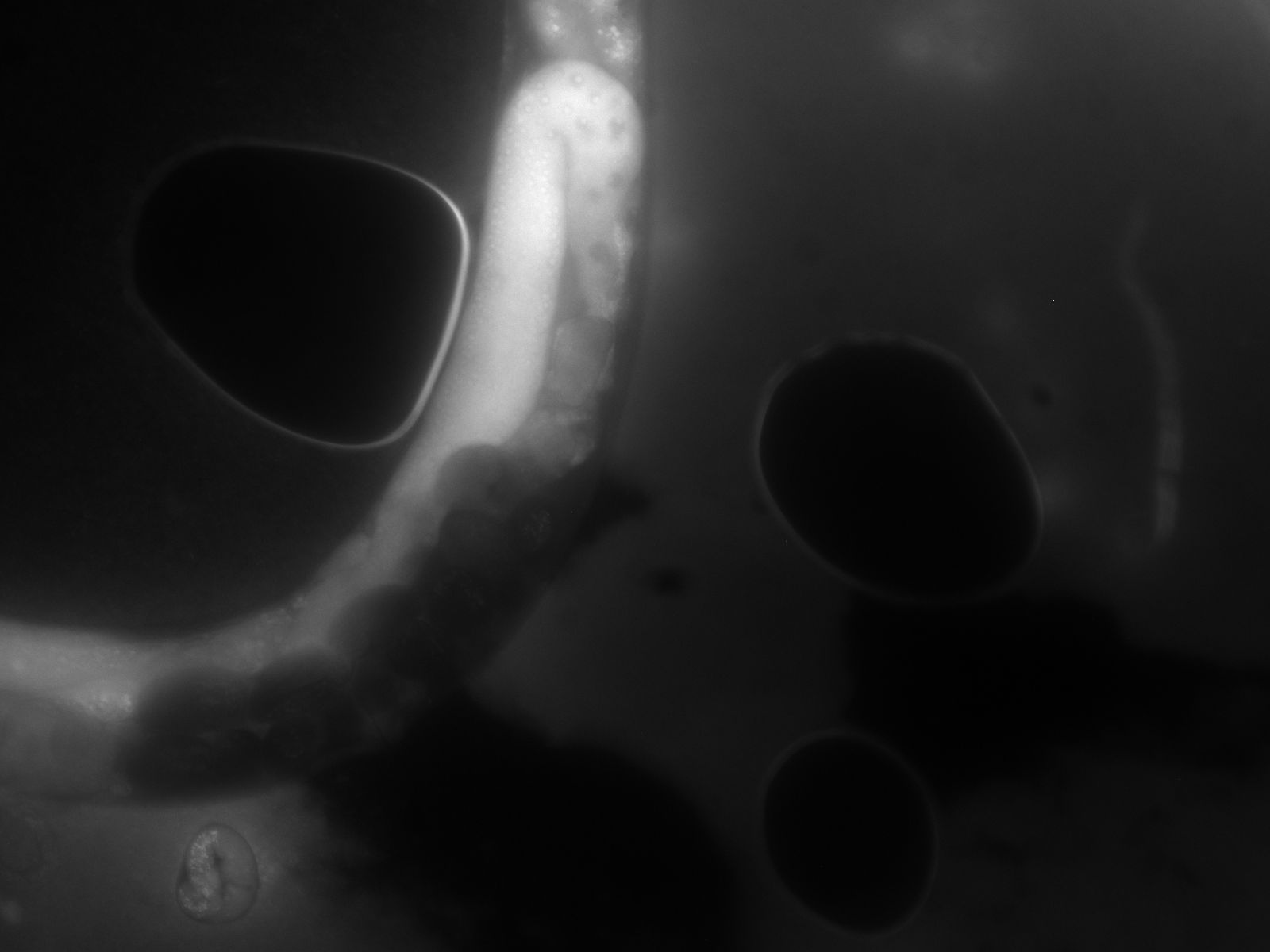

Supplement: S2 File — (ZIP) [file pgen.1011061.s002.zip › Fig.2A - Original files/Fig.2A RAW data and photos JPEG/syto12 staining - fig 2A - 1_rep - 14.5.23 jpeg/xbp-1_ire-1+pad12171.jpg]

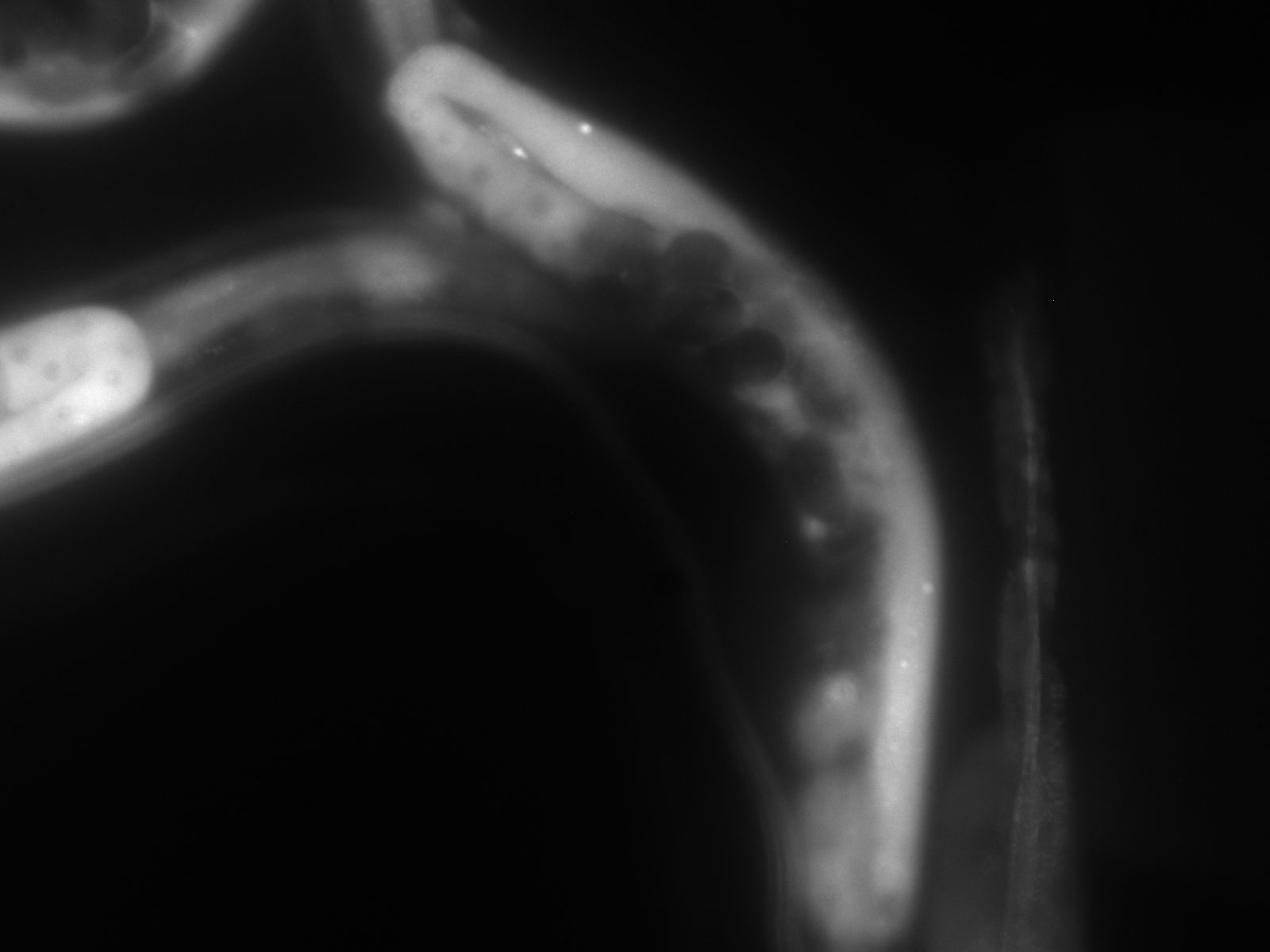

Supplement: S2 File — (ZIP) [file pgen.1011061.s002.zip › Fig.2A - Original files/Fig.2A RAW data and photos JPEG/syto12 staining - fig 2A - 1_rep - 14.5.23 jpeg/xbp-1_ire-1+pad12172.jpg]

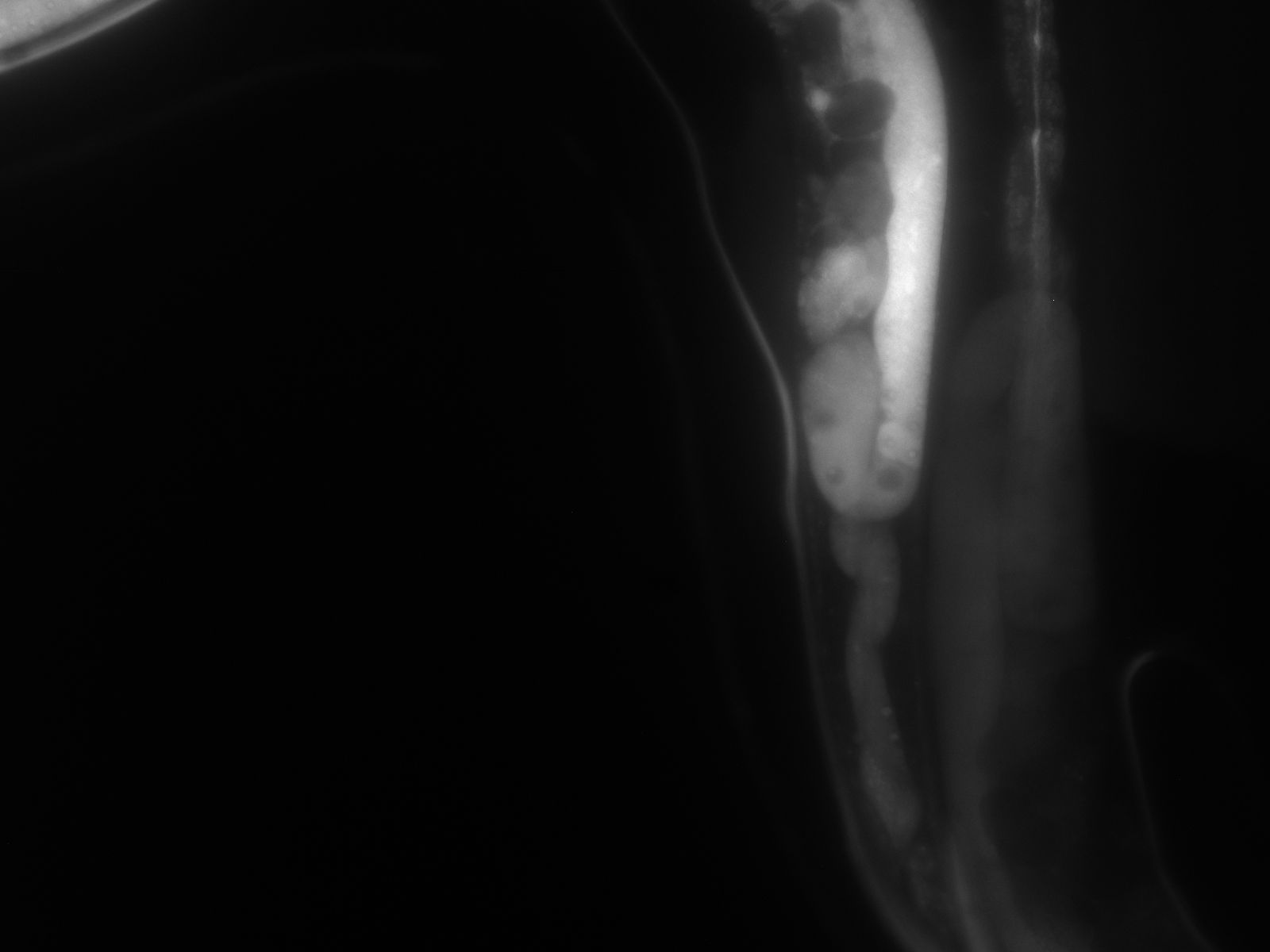

Supplement: S2 File — (ZIP) [file pgen.1011061.s002.zip › Fig.2A - Original files/Fig.2A RAW data and photos JPEG/syto12 staining - fig 2A - 1_rep - 14.5.23 jpeg/xbp-1_ire-1+pad12173.jpg]

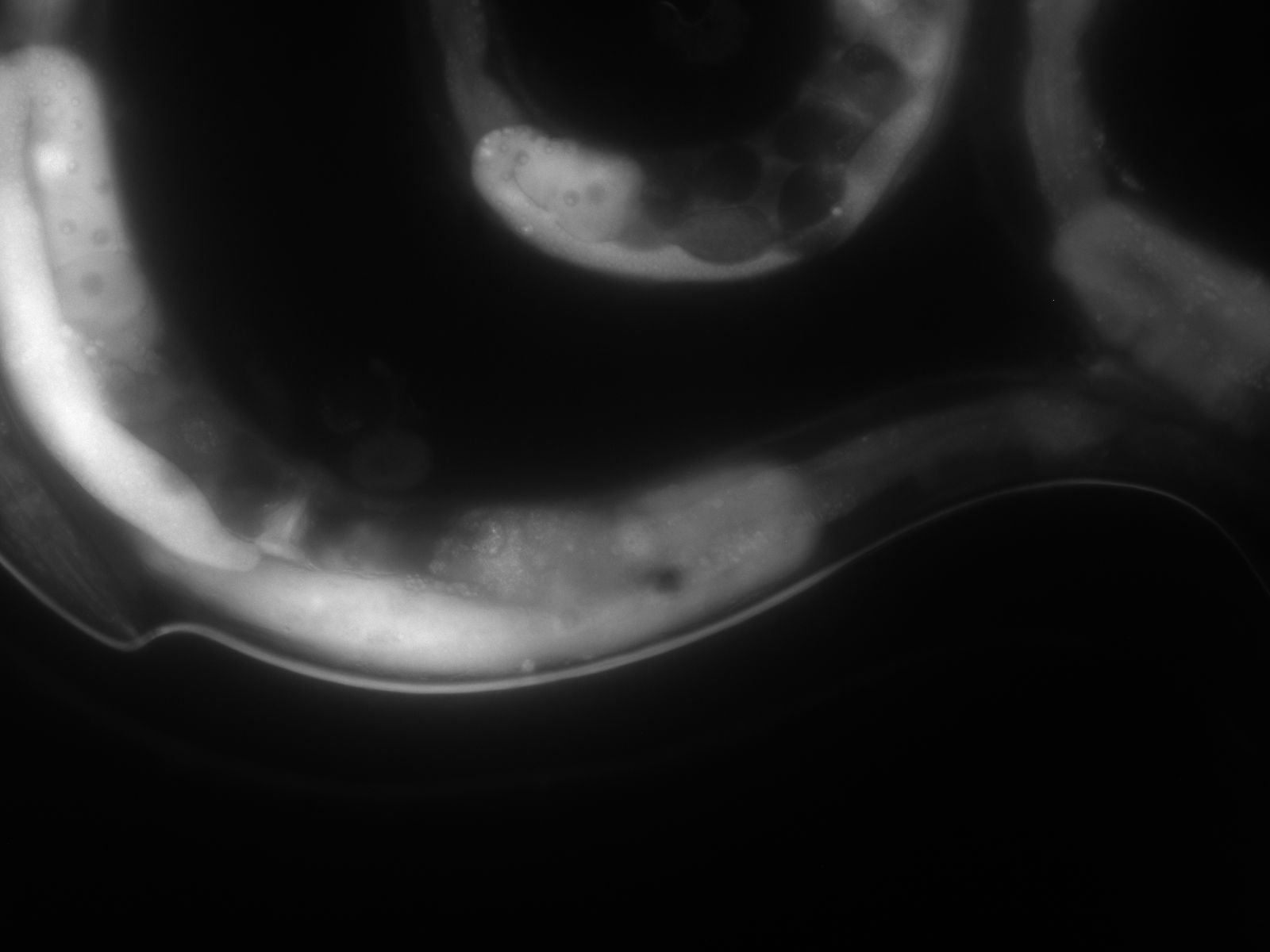

Supplement: S2 File — (ZIP) [file pgen.1011061.s002.zip › Fig.2A - Original files/Fig.2A RAW data and photos JPEG/syto12 staining - fig 2A - 1_rep - 14.5.23 jpeg/xbp-1_ire-1+pad12174.jpg]

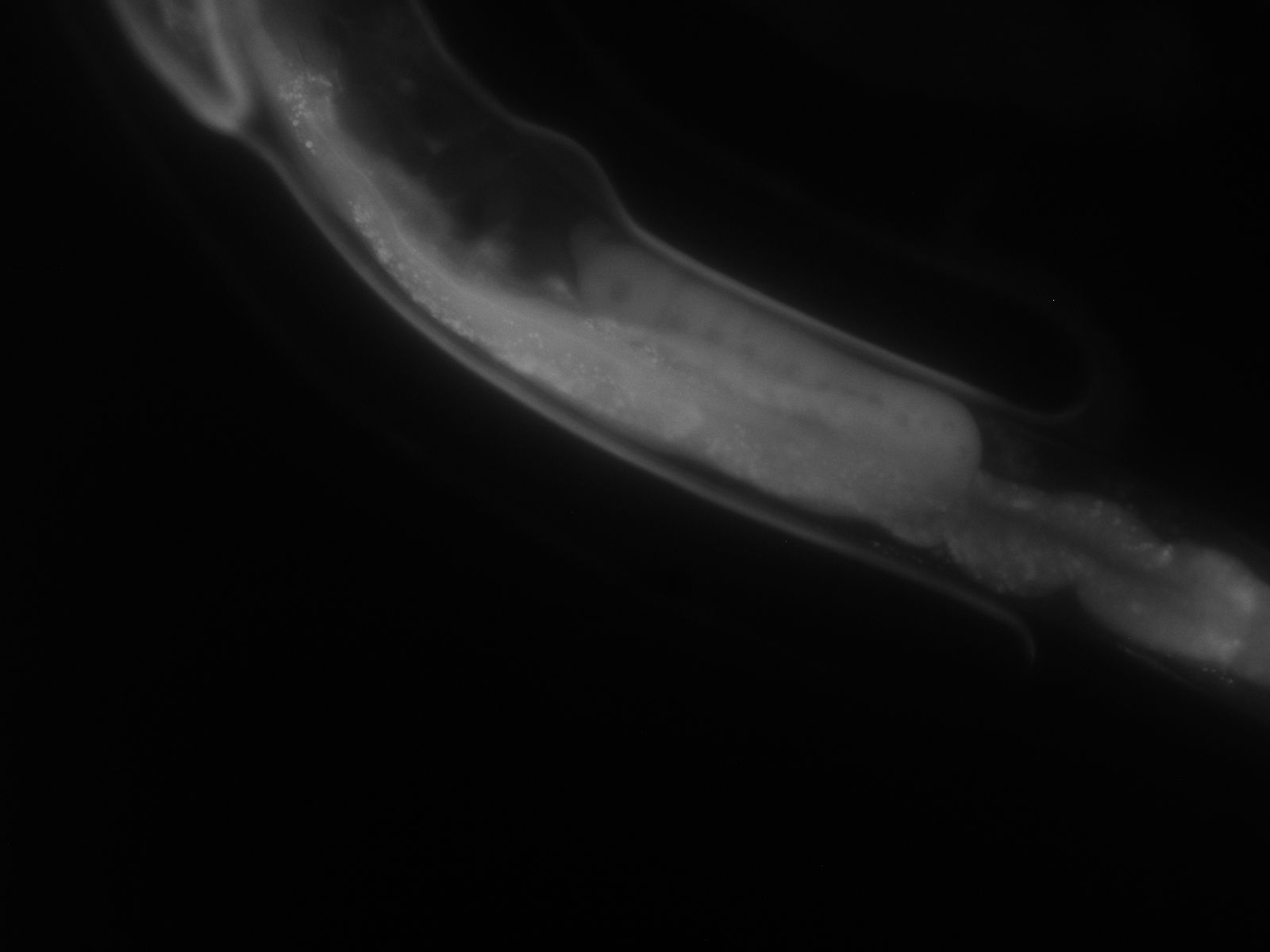

Supplement: S2 File — (ZIP) [file pgen.1011061.s002.zip › Fig.2A - Original files/Fig.2A RAW data and photos JPEG/syto12 staining - fig 2A - 1_rep - 14.5.23 jpeg/xbp-1_ire-1+pad12175.jpg]

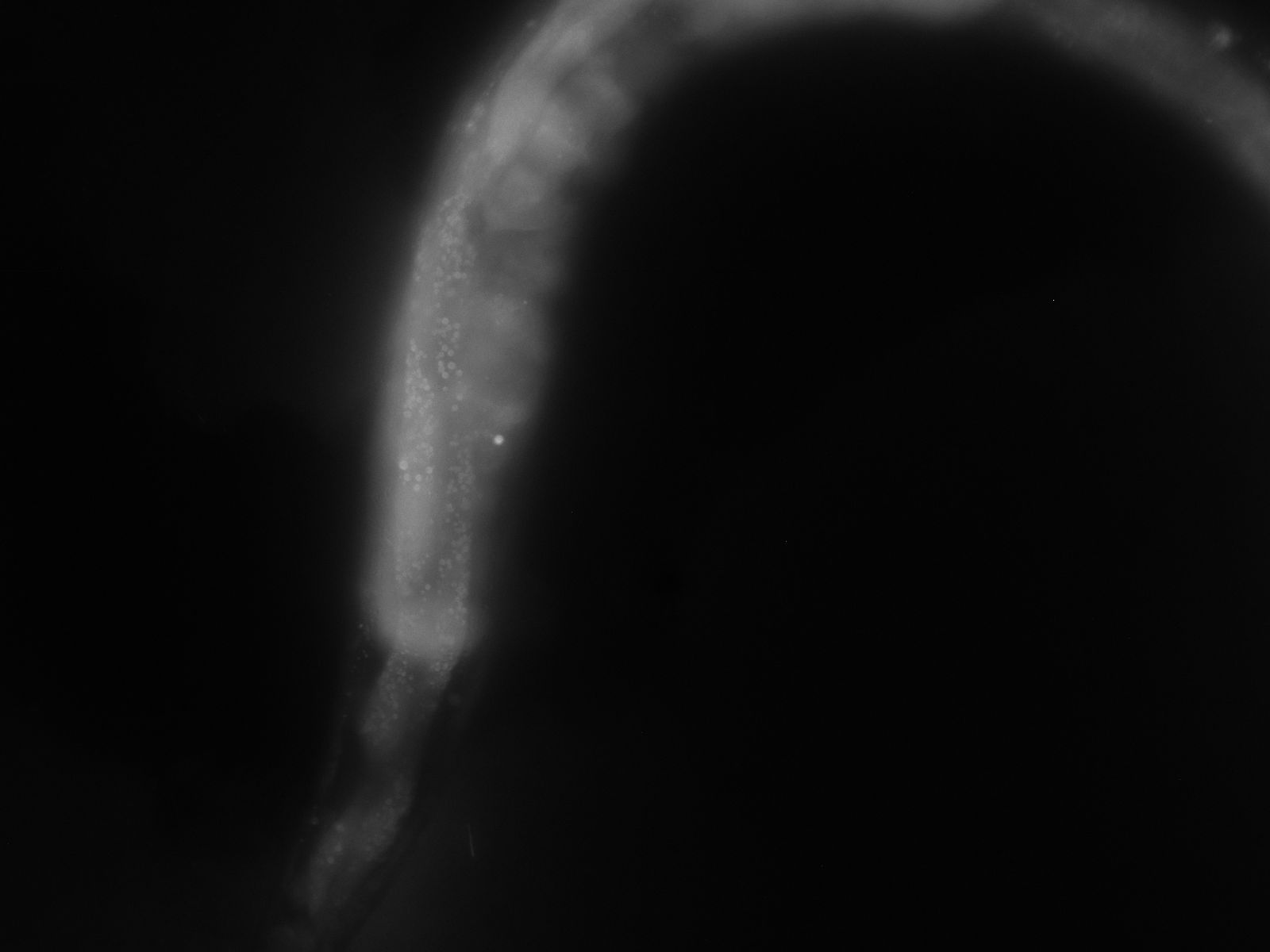

Supplement: S2 File — (ZIP) [file pgen.1011061.s002.zip › Fig.2A - Original files/Fig.2A RAW data and photos JPEG/syto12 staining - fig 2A - 1_rep - 14.5.23 jpeg/xbp-1_ire-1+pad12176.jpg]

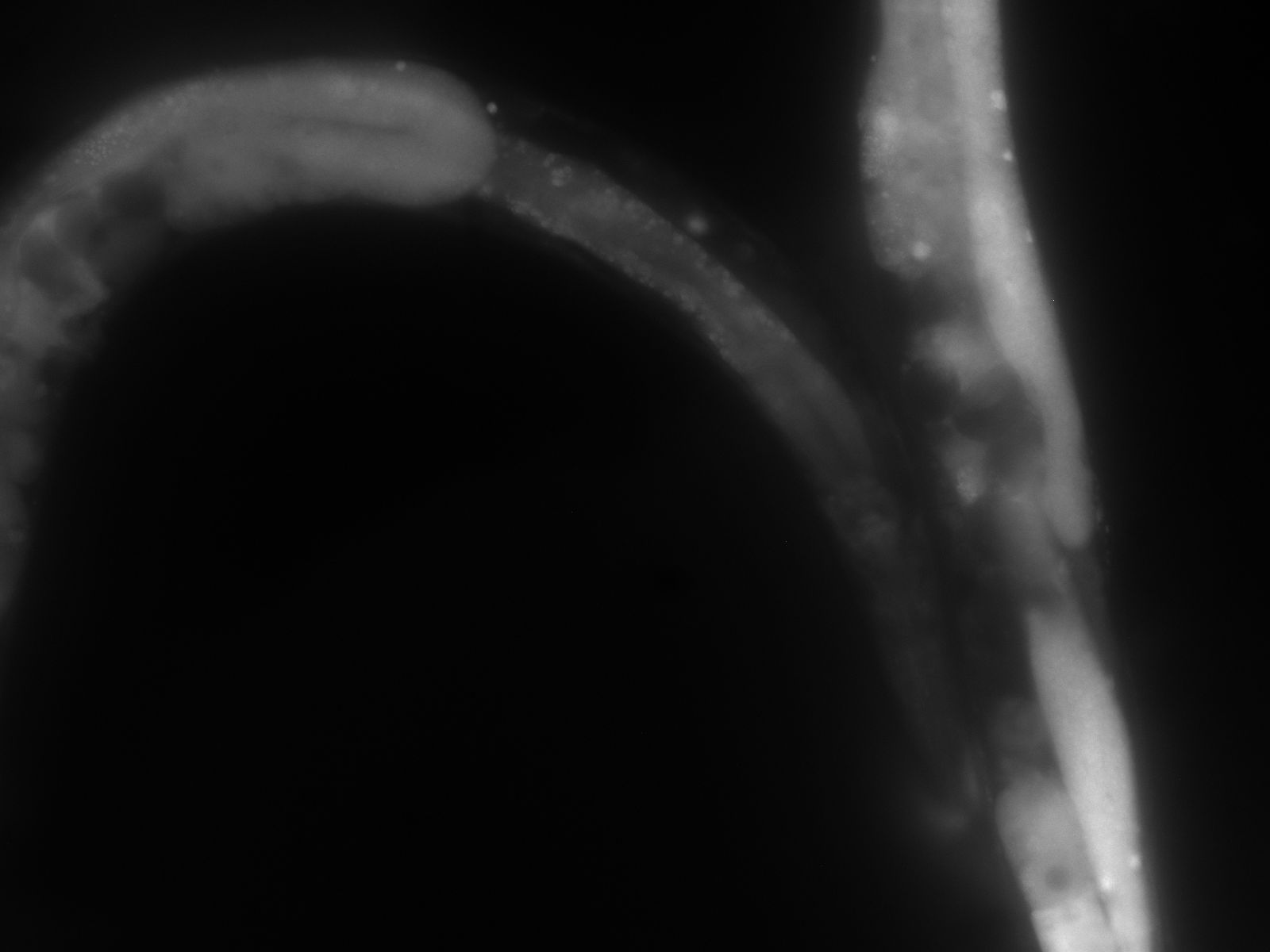

Supplement: S2 File — (ZIP) [file pgen.1011061.s002.zip › Fig.2A - Original files/Fig.2A RAW data and photos JPEG/syto12 staining - fig 2A - 1_rep - 14.5.23 jpeg/xbp-1_ire-1+pad12177.jpg]

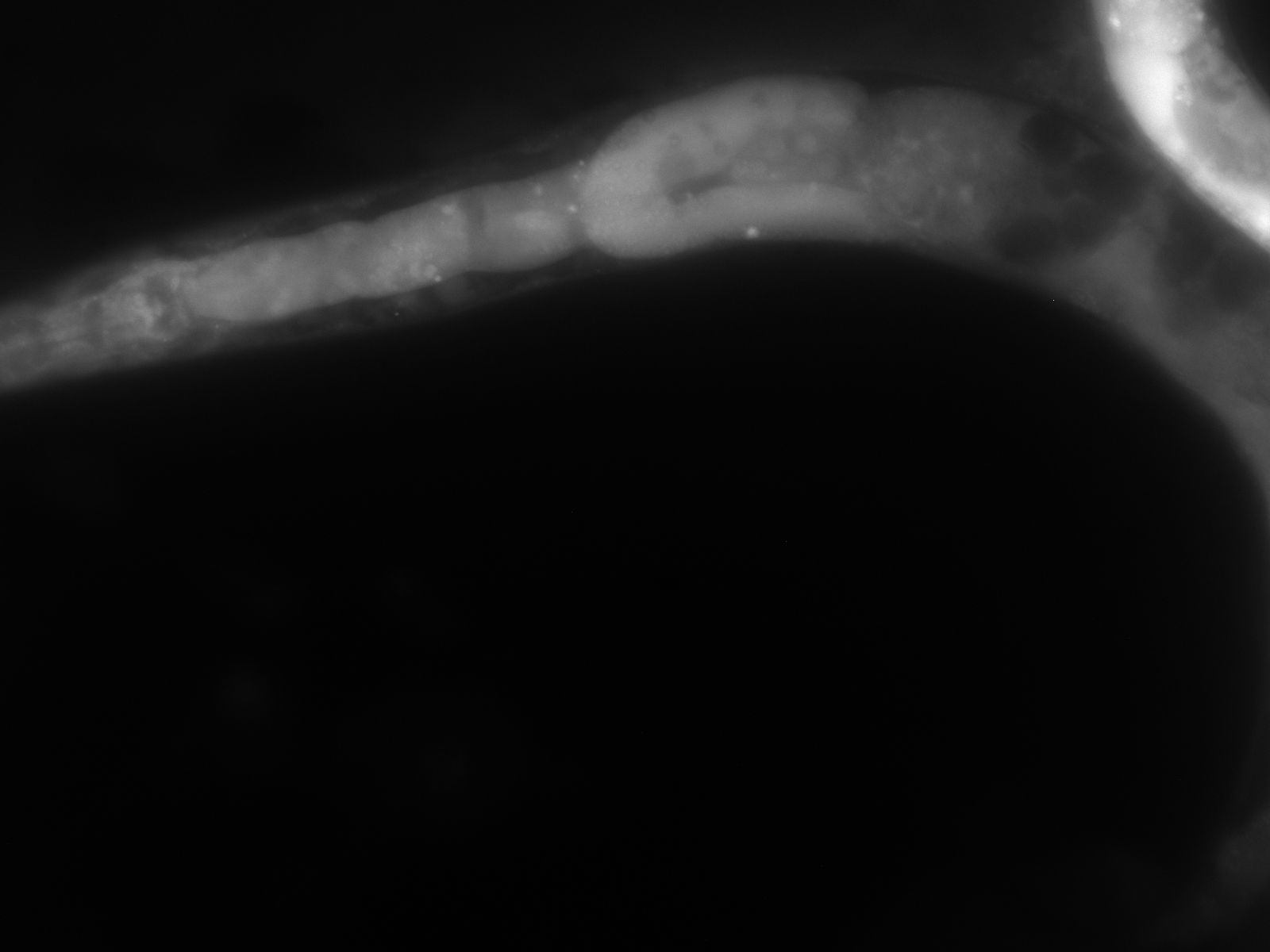

Supplement: S2 File — (ZIP) [file pgen.1011061.s002.zip › Fig.2A - Original files/Fig.2A RAW data and photos JPEG/syto12 staining - fig 2A - 1_rep - 14.5.23 jpeg/xbp-1_ire-1+pad12178.jpg]

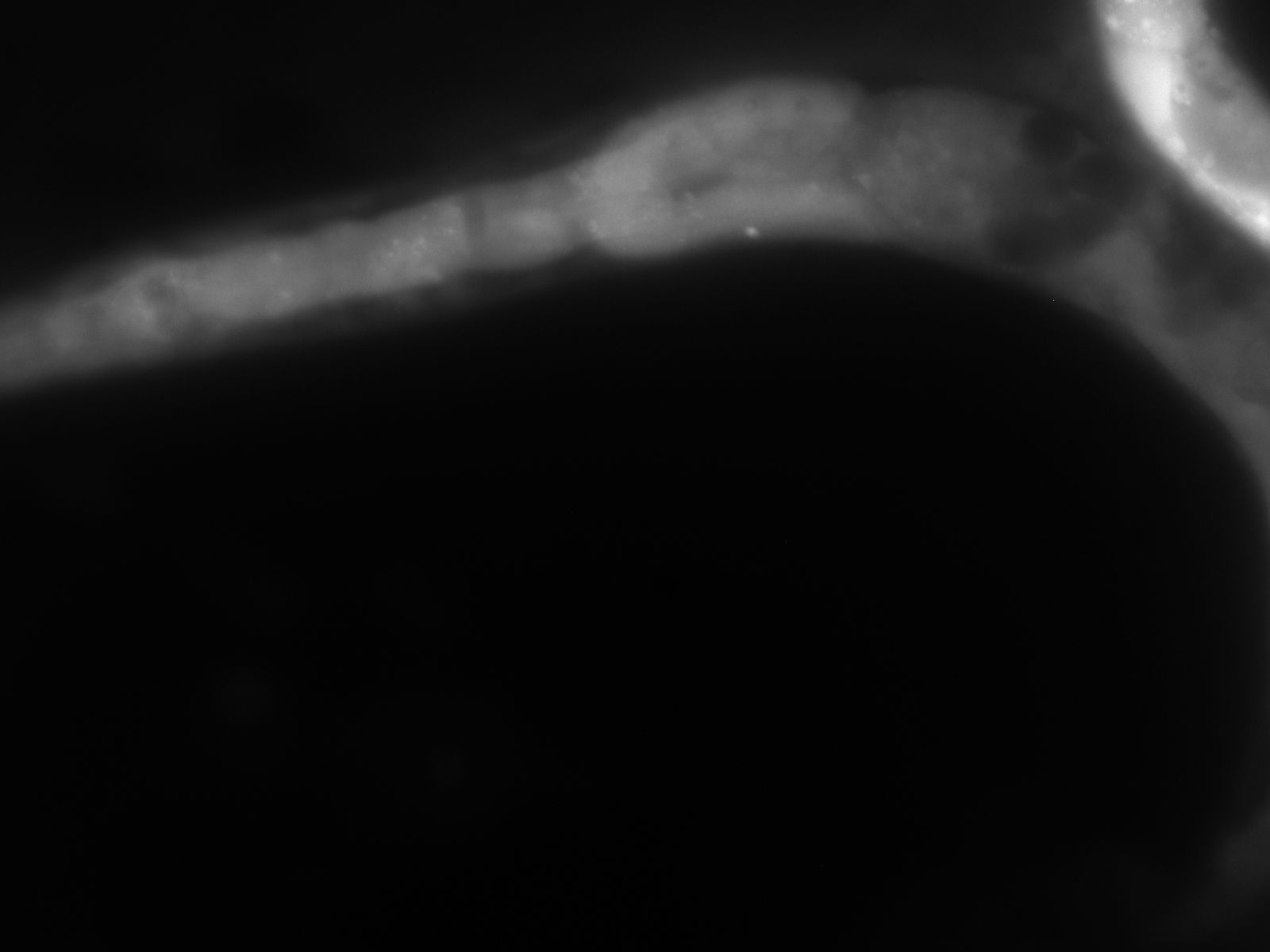

Supplement: S2 File — (ZIP) [file pgen.1011061.s002.zip › Fig.2A - Original files/Fig.2A RAW data and photos JPEG/syto12 staining - fig 2A - 1_rep - 14.5.23 jpeg/xbp-1_ire-1+pad12179.jpg]

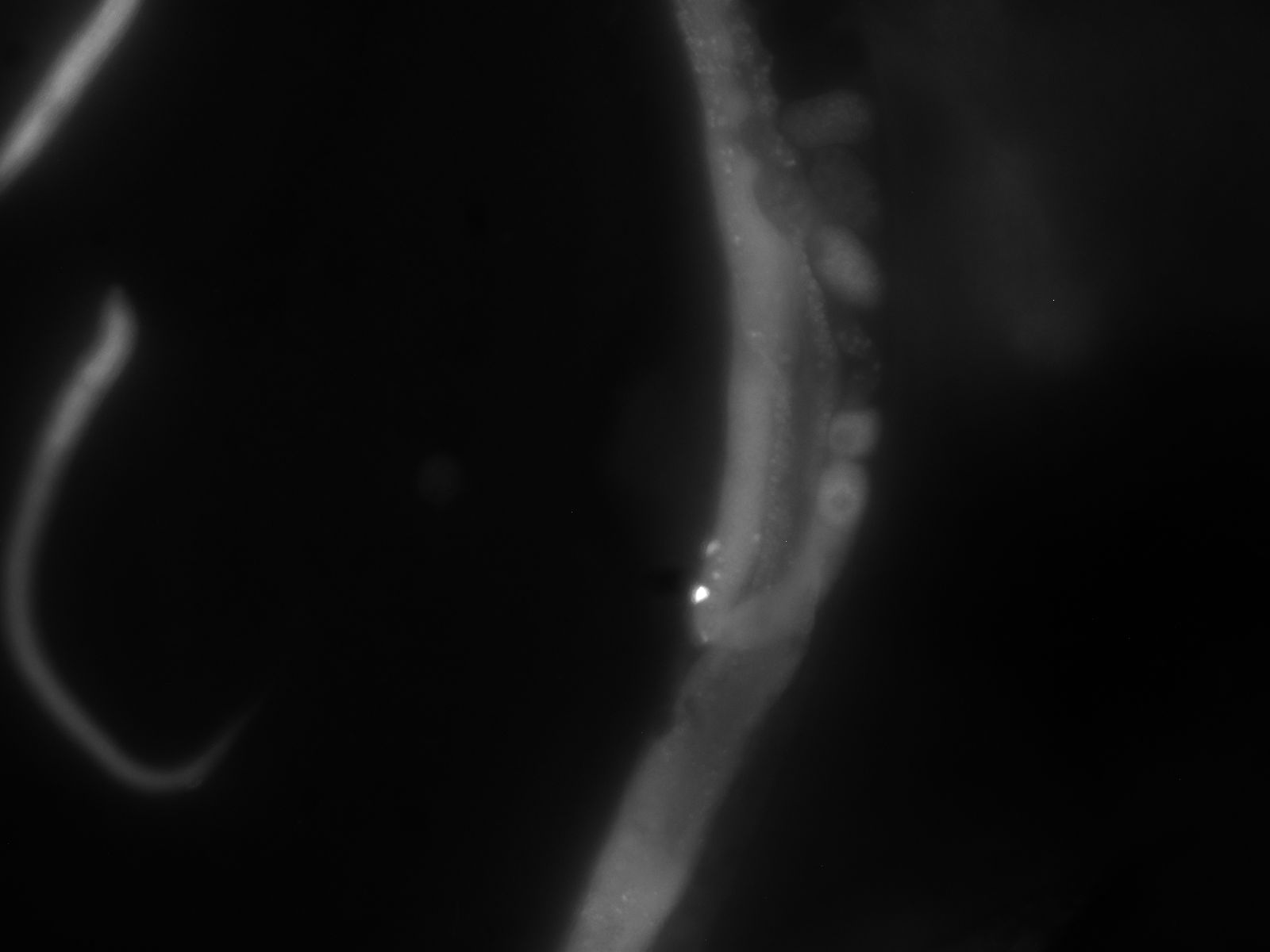

Supplement: S2 File — (ZIP) [file pgen.1011061.s002.zip › Fig.2A - Original files/Fig.2A RAW data and photos JPEG/syto12 staining - fig 2A - 1_rep - 14.5.23 jpeg/xbp-1+pad12100.jpg]
