# Supplementary figures and images for: Correction: It’s All in Your Mind: Determining Germ Cell Fate by Neuronal IRE-1 in C. elegans (part 7 of 7)
Source: PLoS Genet. 2023 Nov 30;19(11):e1011061. doi: 10.1371/journal.pgen.1011061 (PMC10688620; doi:10.1371/journal.pgen.1011061)

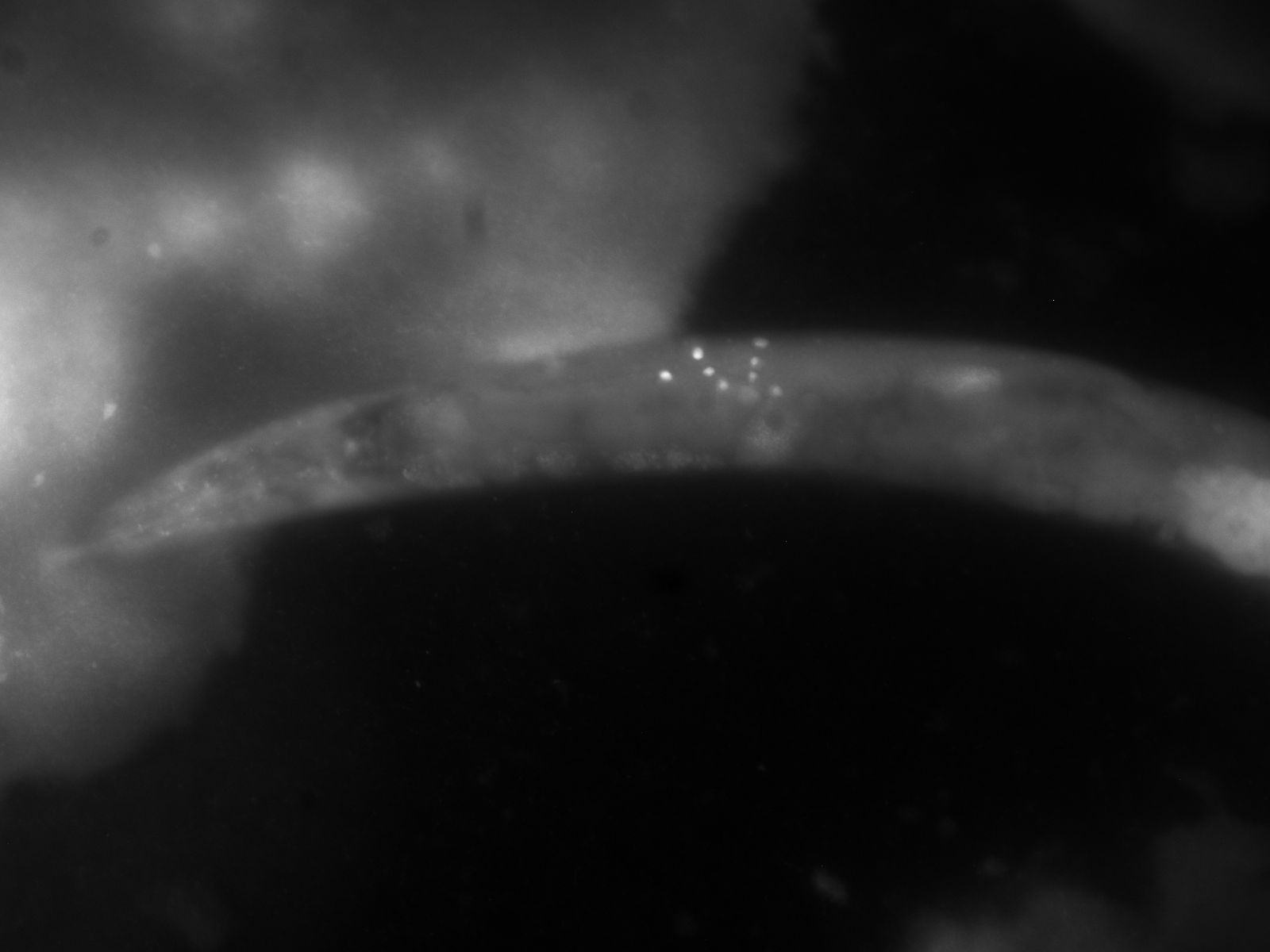

Supplement: S6 File — (ZIP) [file pgen.1011061.s006.zip › Fig.S4B+C - Original files/Fig.S4 RAW data and photos - JPEG/syto12 staining - FigS4bc - 3_rep - 22.5.23/eat-4+tfg-156.jpg]

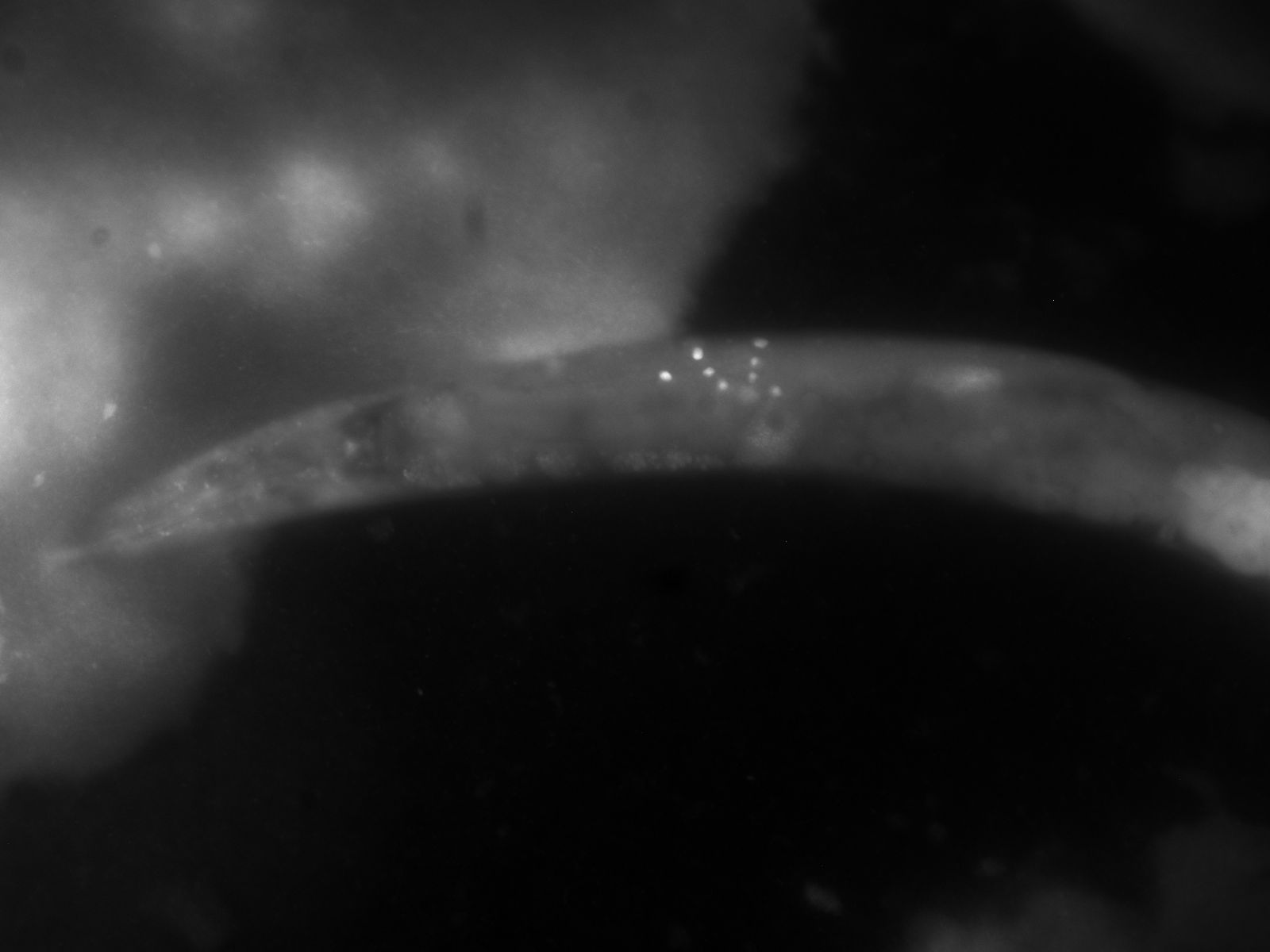

Supplement: S6 File — (ZIP) [file pgen.1011061.s006.zip › Fig.S4B+C - Original files/Fig.S4 RAW data and photos - JPEG/syto12 staining - FigS4bc - 3_rep - 22.5.23/eat-4+tfg-157.jpg]

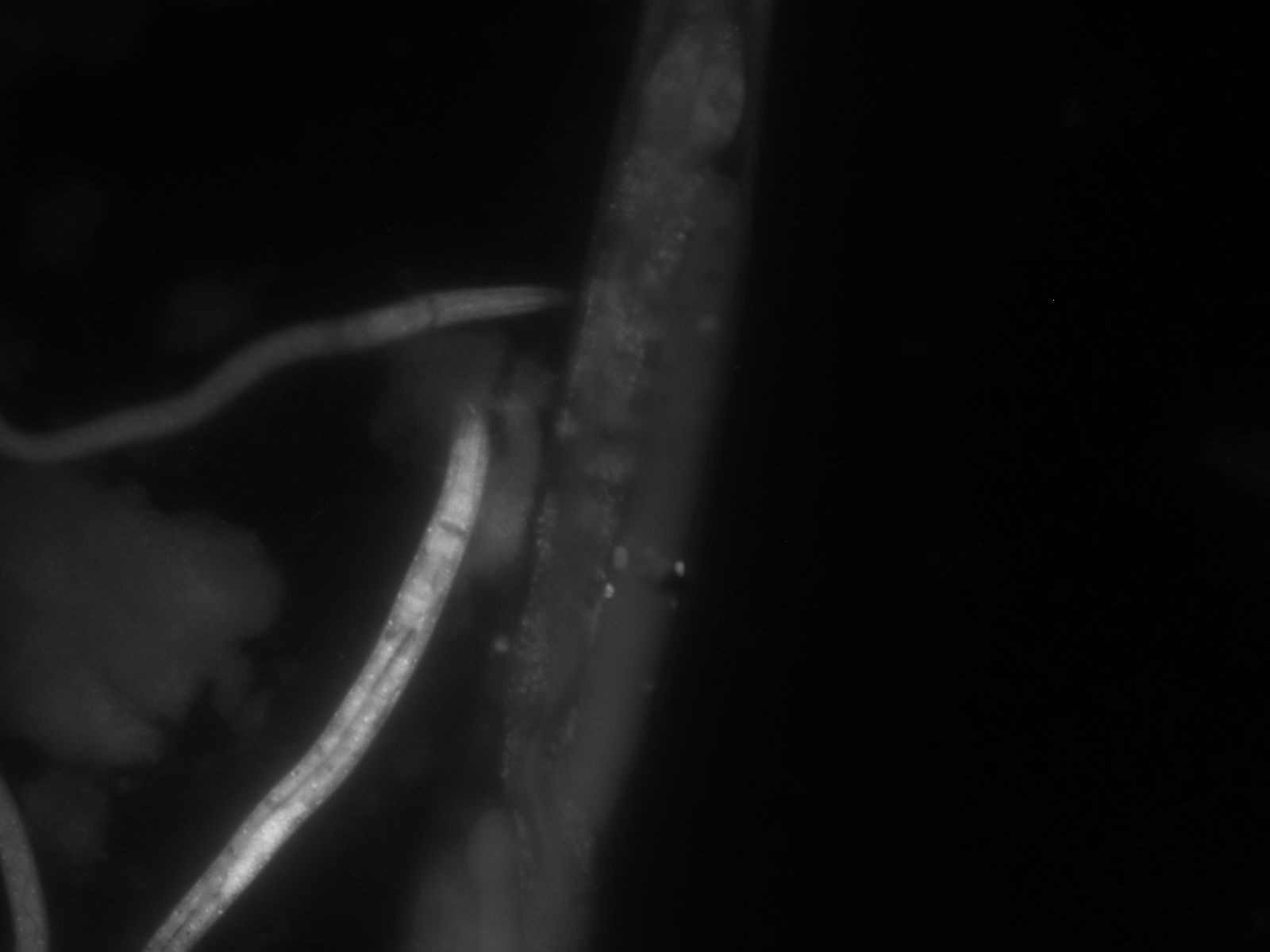

Supplement: S6 File — (ZIP) [file pgen.1011061.s006.zip › Fig.S4B+C - Original files/Fig.S4 RAW data and photos - JPEG/syto12 staining - FigS4bc - 3_rep - 22.5.23/eat-4+tfg-158.jpg]

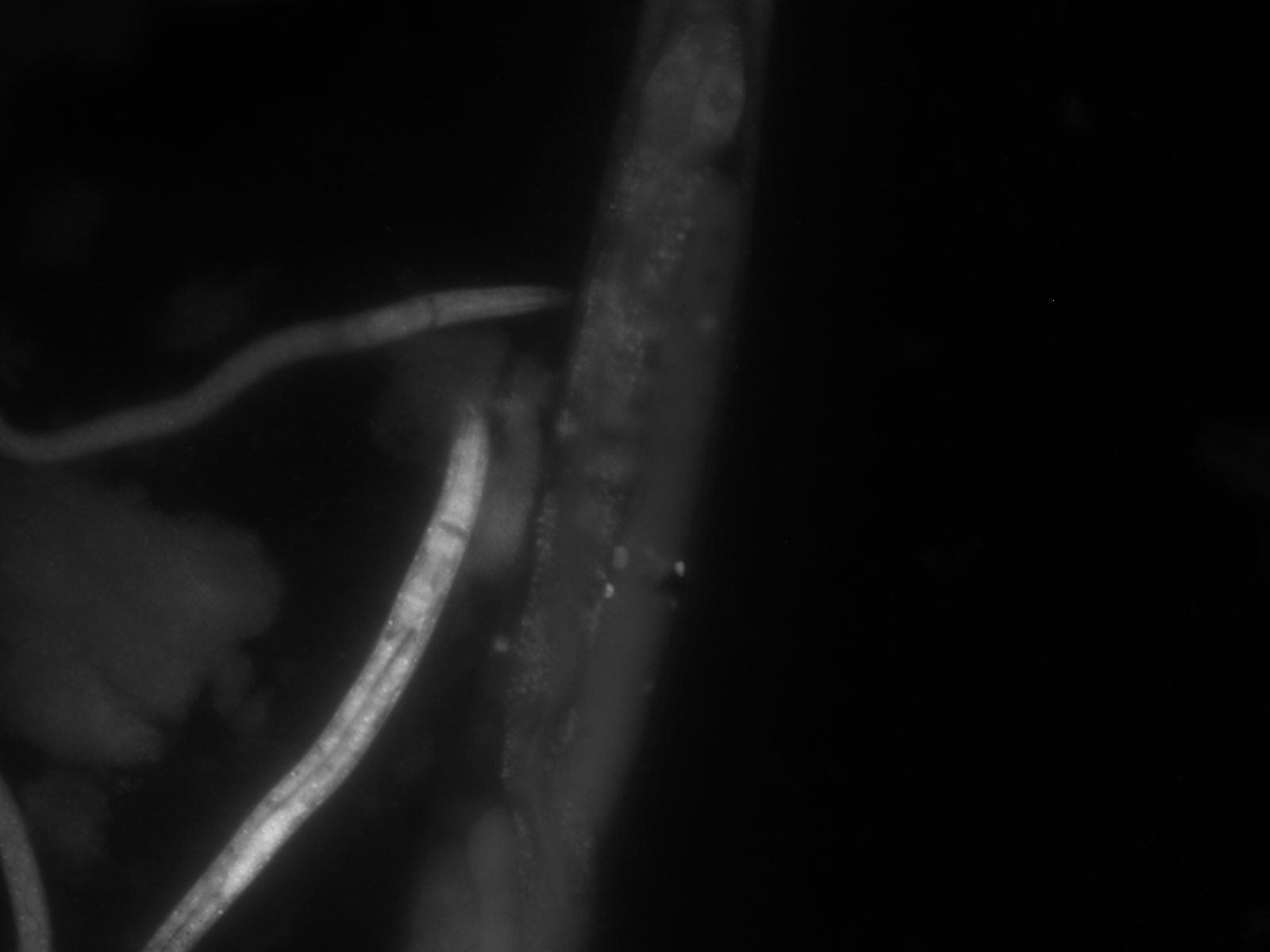

Supplement: S6 File — (ZIP) [file pgen.1011061.s006.zip › Fig.S4B+C - Original files/Fig.S4 RAW data and photos - JPEG/syto12 staining - FigS4bc - 3_rep - 22.5.23/eat-4+tfg-159.jpg]

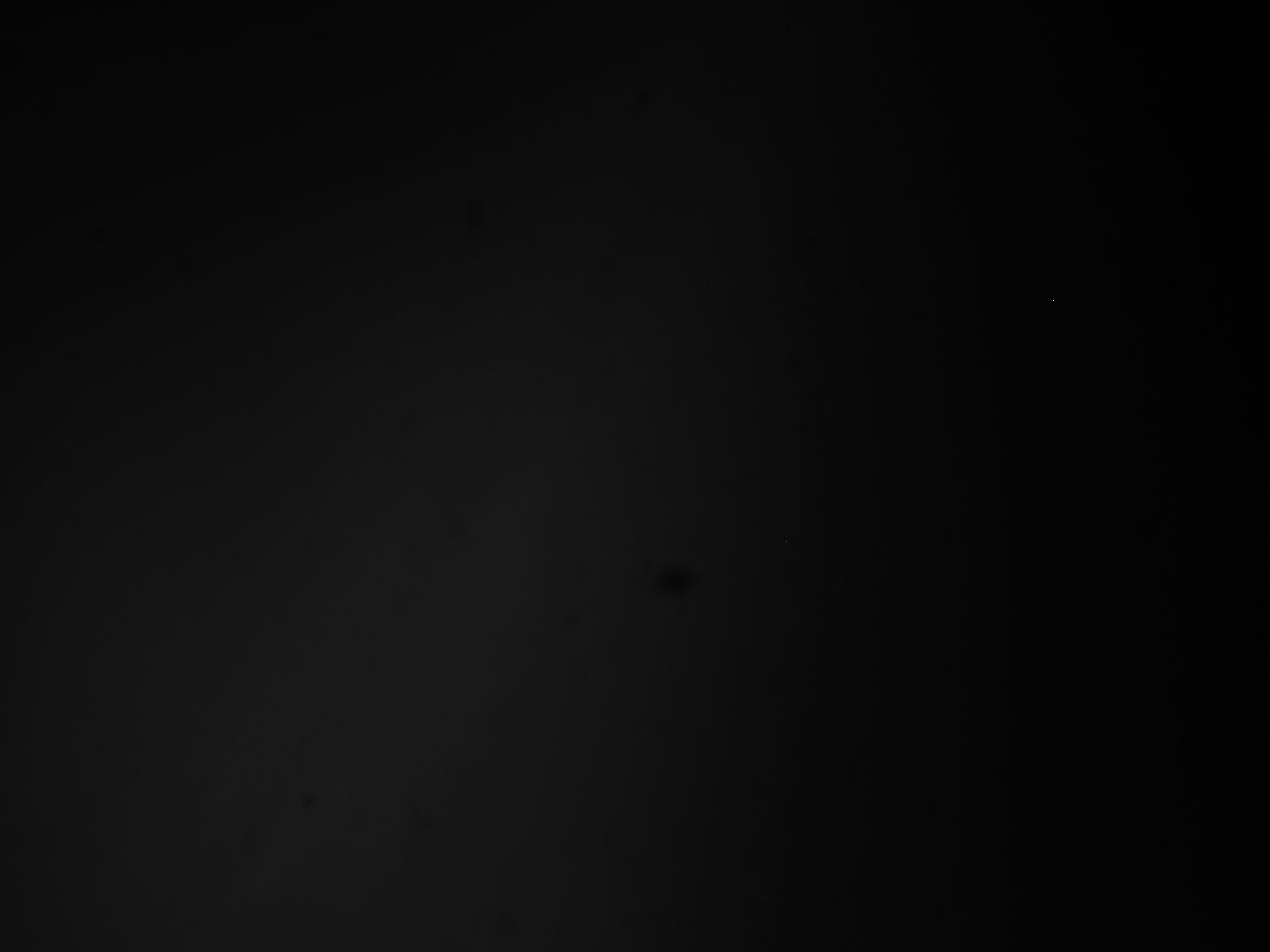

Supplement: S6 File — (ZIP) [file pgen.1011061.s006.zip › Fig.S4B+C - Original files/Fig.S4 RAW data and photos - JPEG/syto12 staining - FigS4bc - 3_rep - 22.5.23/eat-4+tfg-160.jpg]

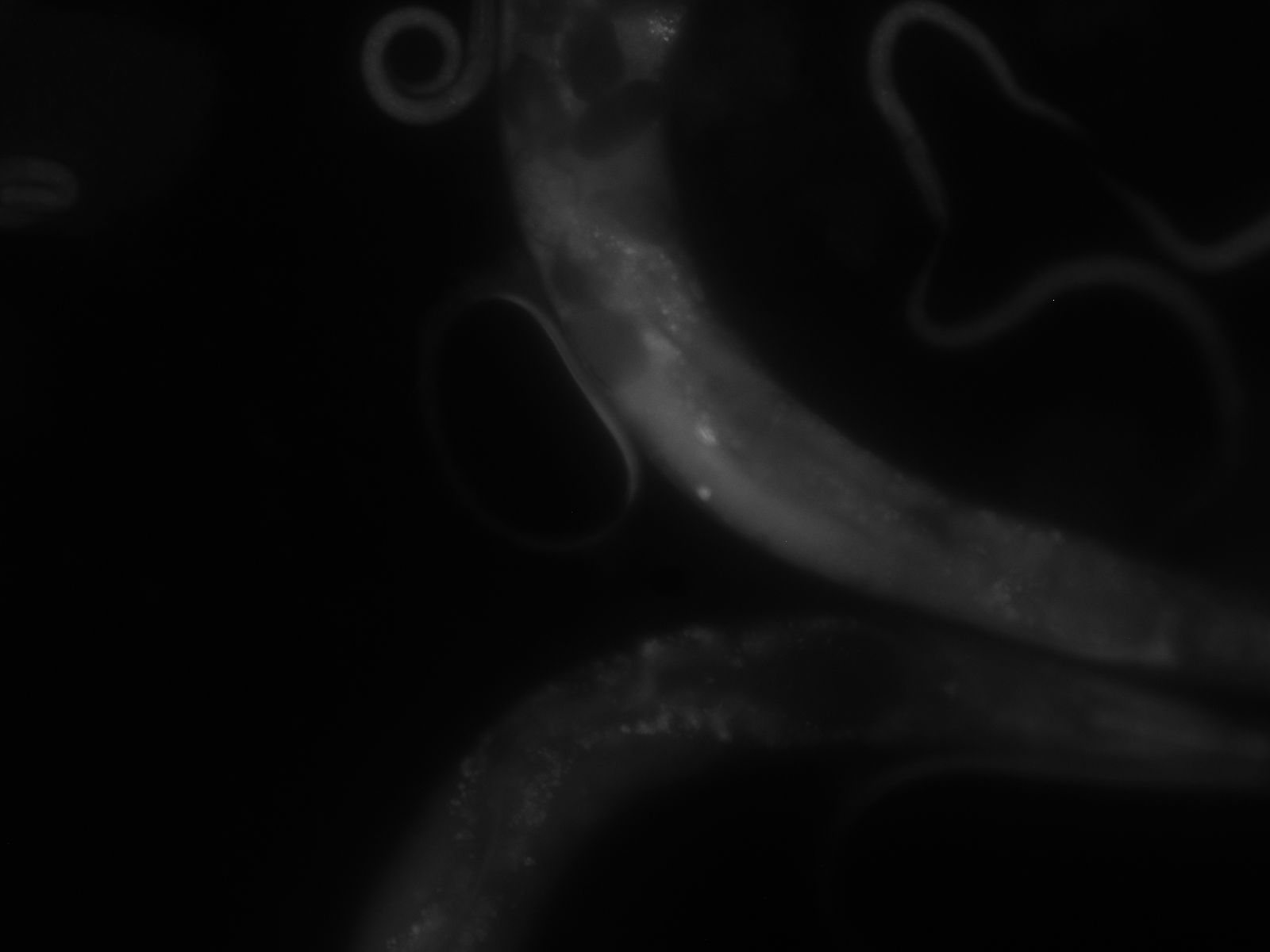

Supplement: S6 File — (ZIP) [file pgen.1011061.s006.zip › Fig.S4B+C - Original files/Fig.S4 RAW data and photos - JPEG/syto12 staining - FigS4bc - 3_rep - 22.5.23/unc-13+pad1261.jpg]

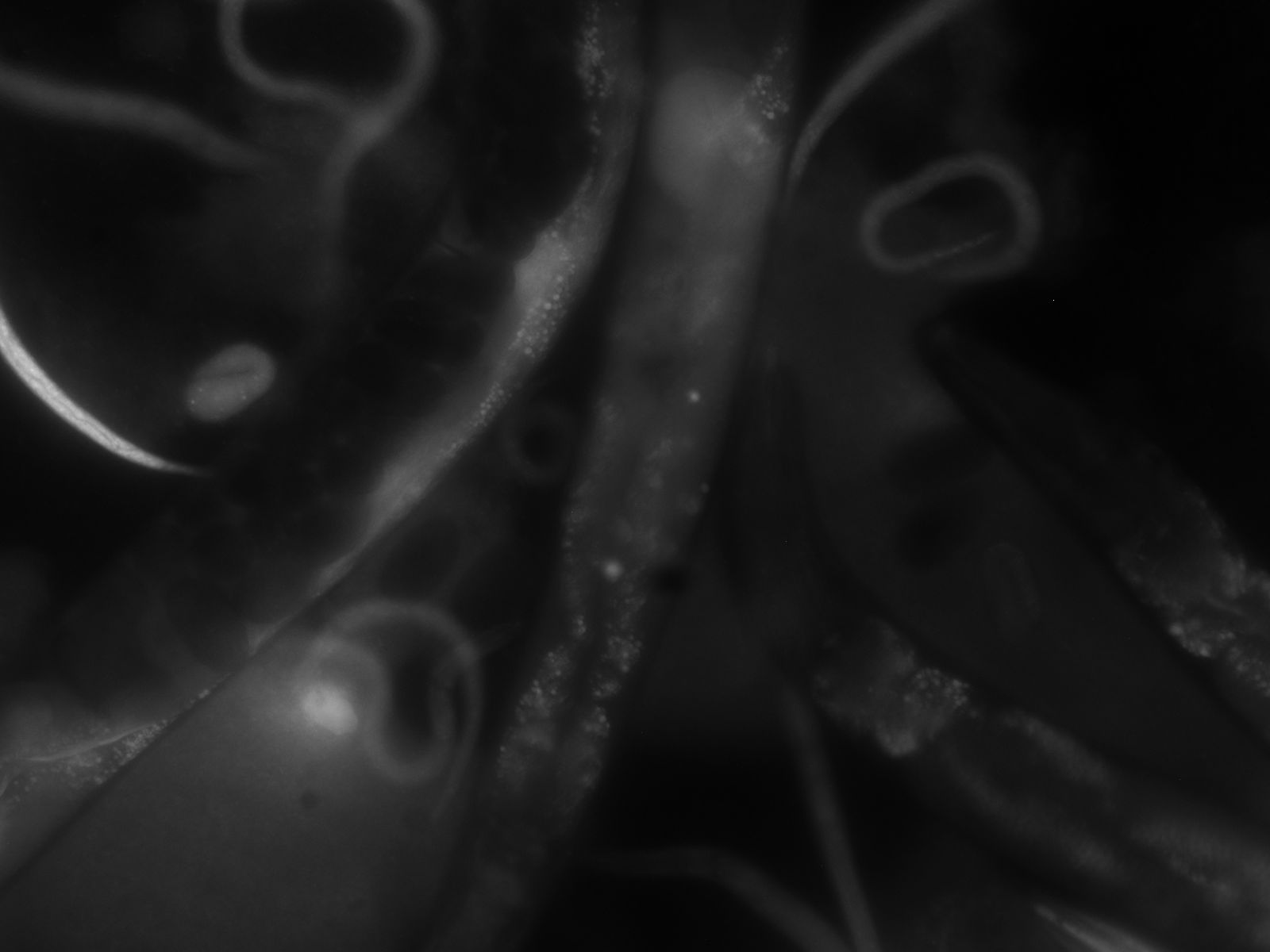

Supplement: S6 File — (ZIP) [file pgen.1011061.s006.zip › Fig.S4B+C - Original files/Fig.S4 RAW data and photos - JPEG/syto12 staining - FigS4bc - 3_rep - 22.5.23/unc-13+pad1262.jpg]

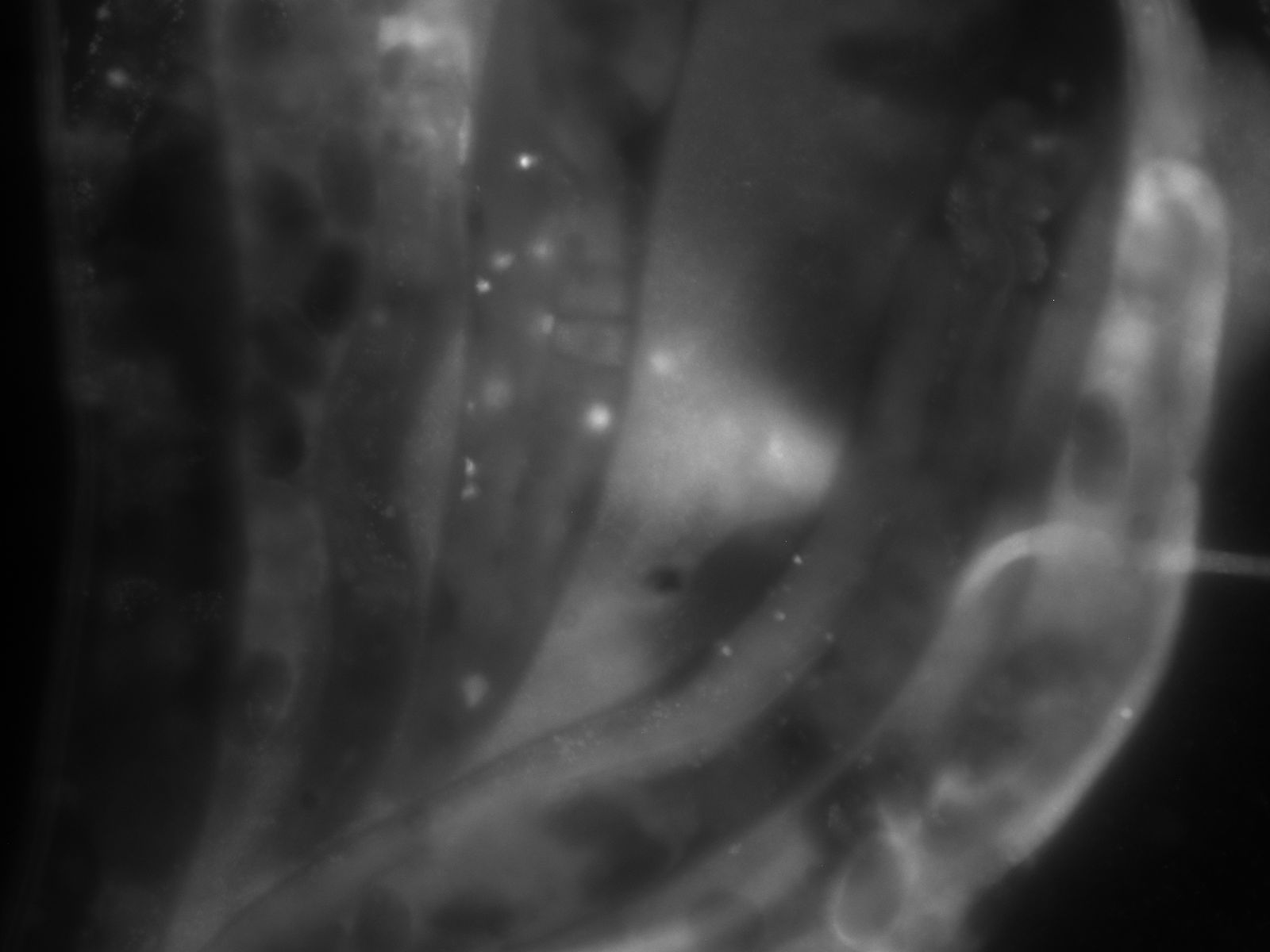

Supplement: S6 File — (ZIP) [file pgen.1011061.s006.zip › Fig.S4B+C - Original files/Fig.S4 RAW data and photos - JPEG/syto12 staining - FigS4bc - 3_rep - 22.5.23/unc-13+tfg-163.jpg]

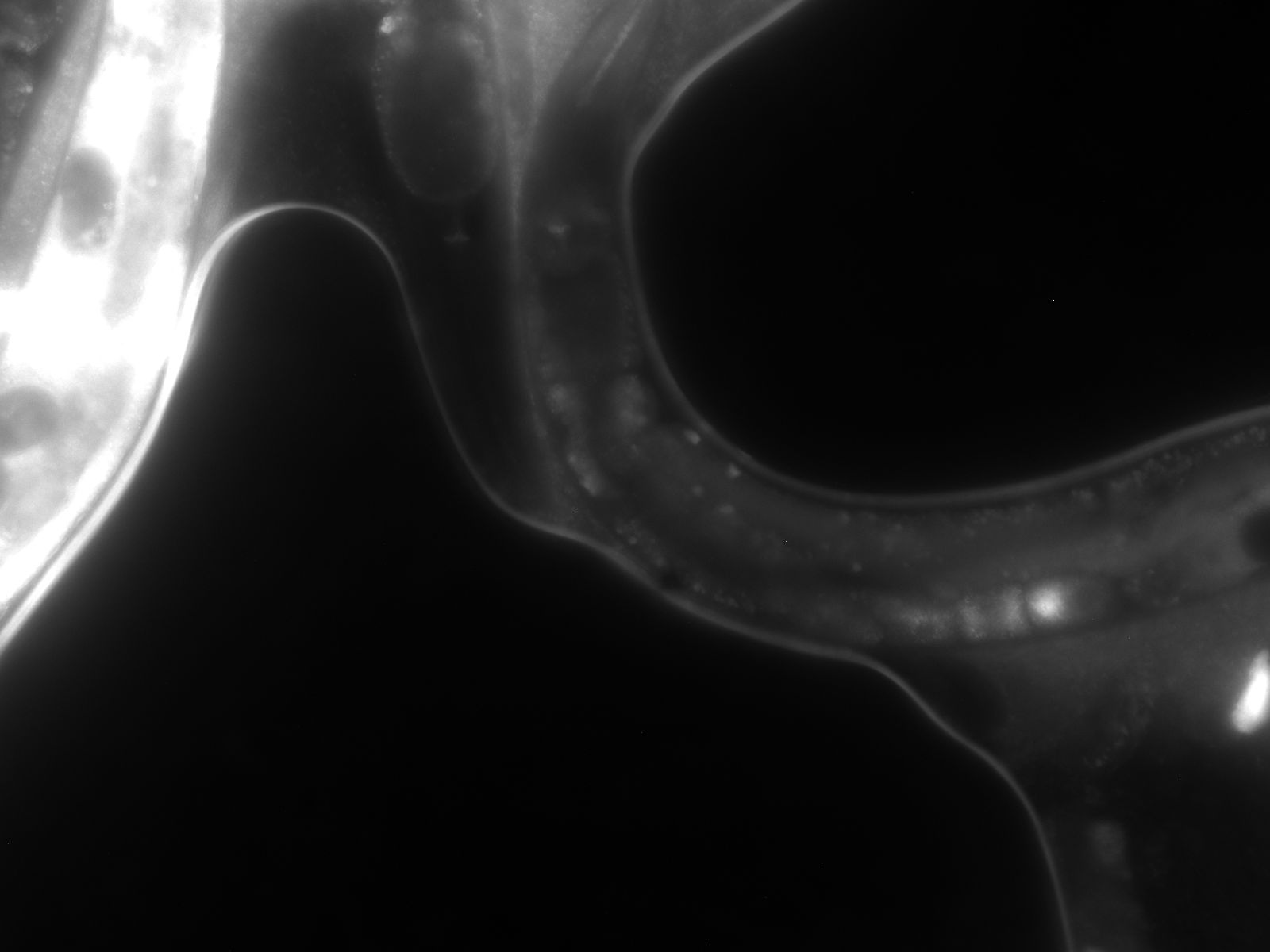

Supplement: S6 File — (ZIP) [file pgen.1011061.s006.zip › Fig.S4B+C - Original files/Fig.S4 RAW data and photos - JPEG/syto12 staining - FigS4bc - 3_rep - 22.5.23/unc-13+tfg-164.jpg]

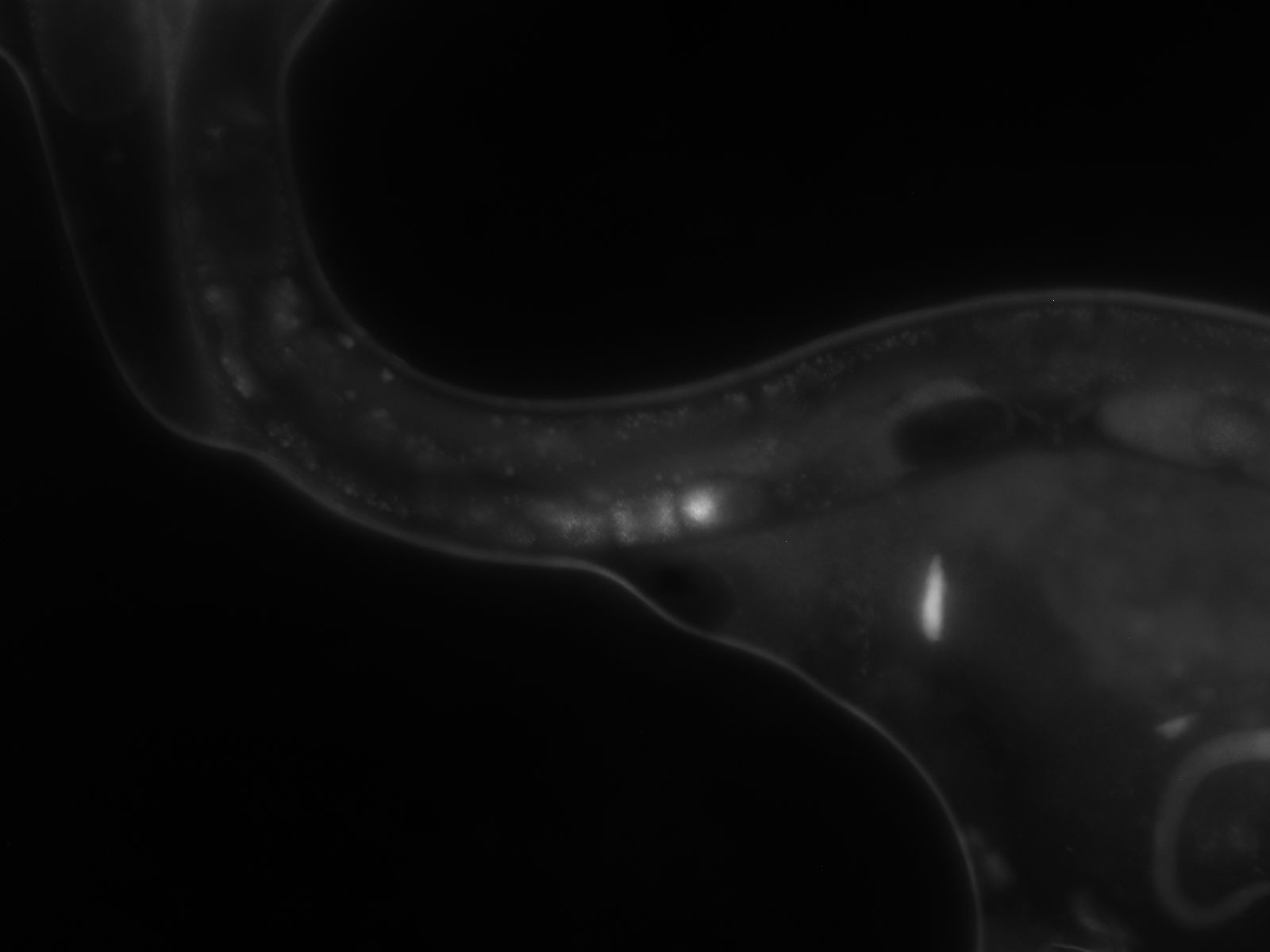

Supplement: S6 File — (ZIP) [file pgen.1011061.s006.zip › Fig.S4B+C - Original files/Fig.S4 RAW data and photos - JPEG/syto12 staining - FigS4bc - 3_rep - 22.5.23/unc-13+tfg-165.jpg]

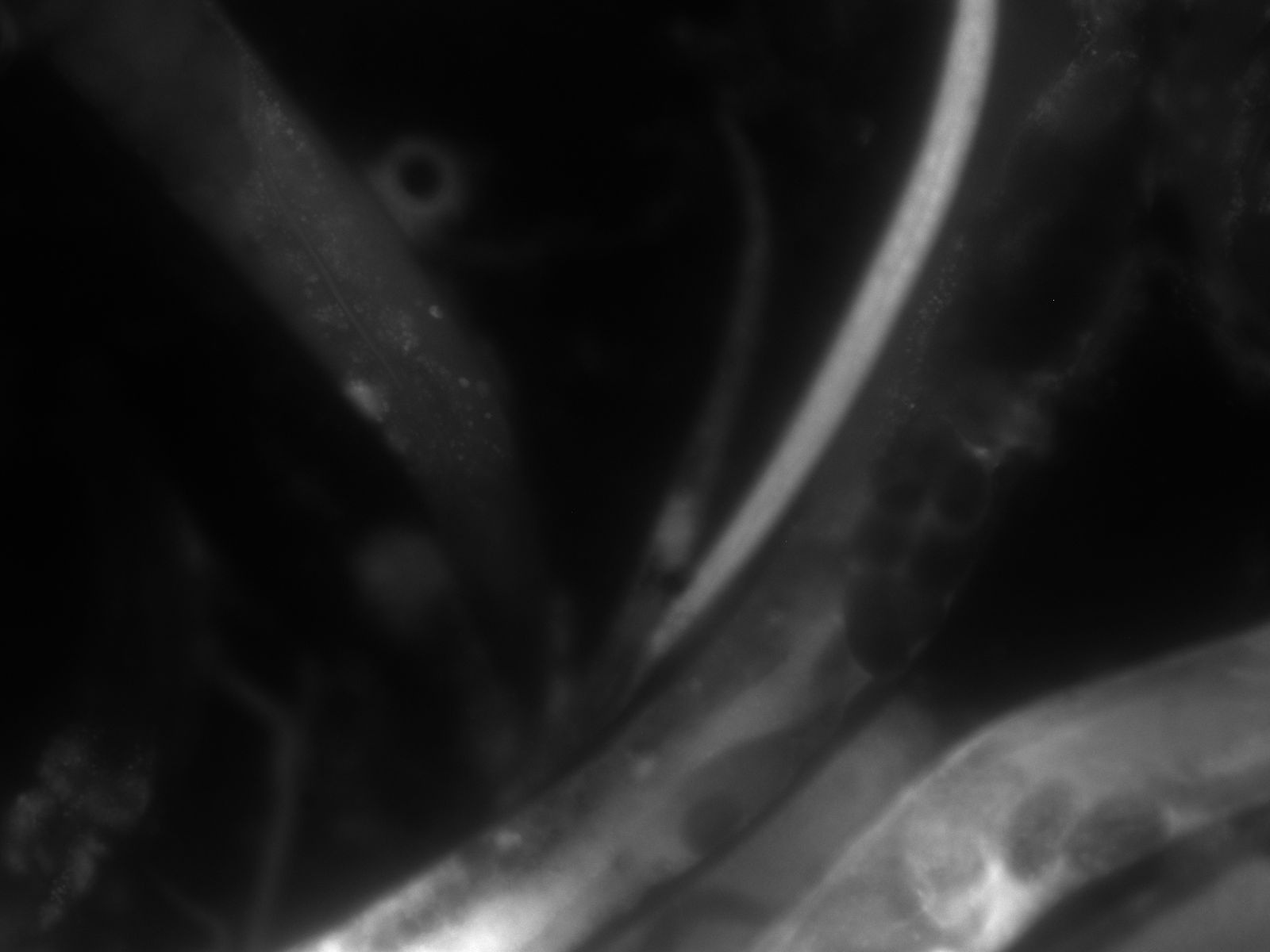

Supplement: S6 File — (ZIP) [file pgen.1011061.s006.zip › Fig.S4B+C - Original files/Fig.S4 RAW data and photos - JPEG/syto12 staining - FigS4bc - 3_rep - 22.5.23/unc-31_unc-64+pad1266.jpg]

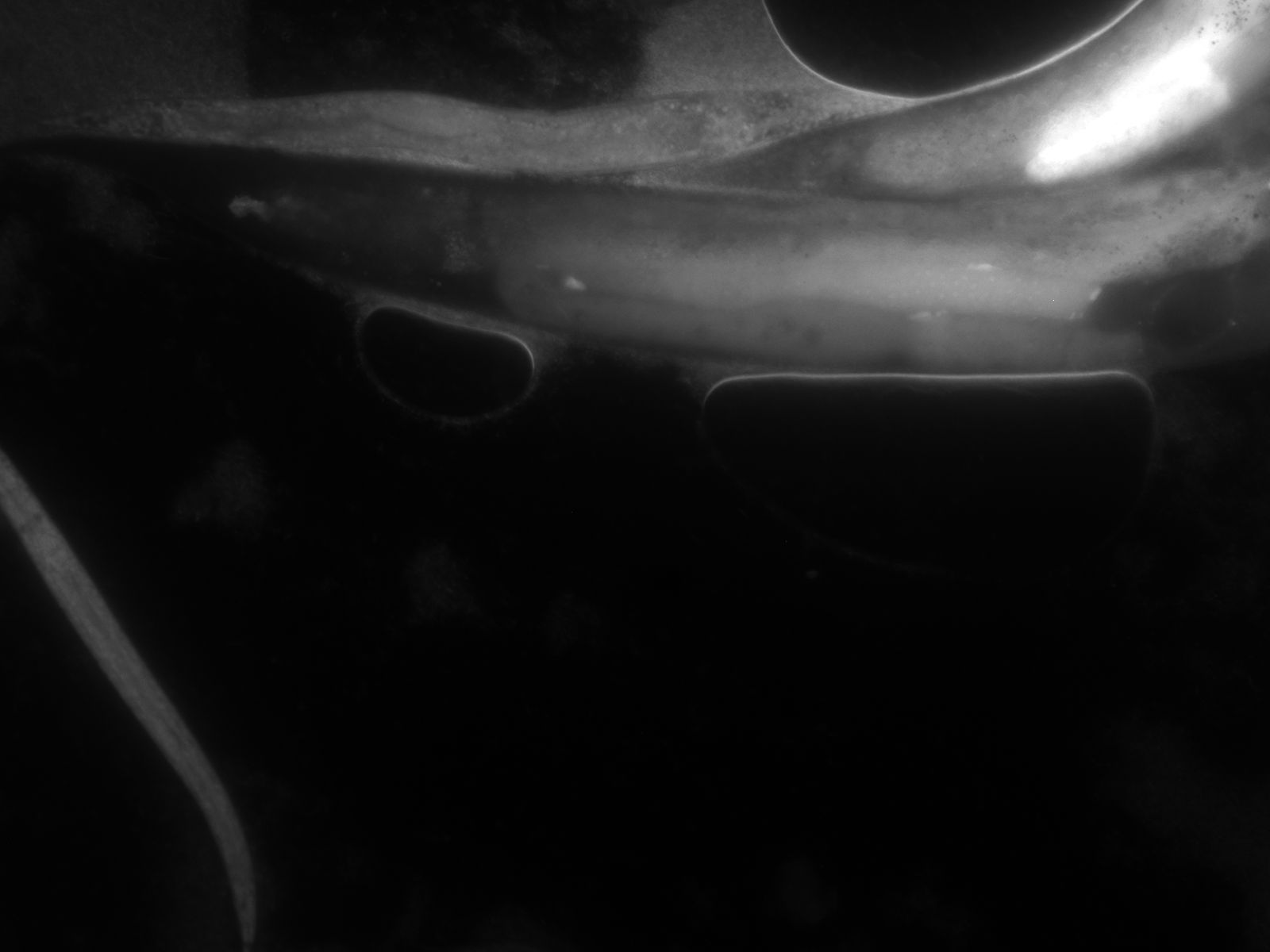

Supplement: S6 File — (ZIP) [file pgen.1011061.s006.zip › Fig.S4B+C - Original files/Fig.S4 RAW data and photos - JPEG/syto12 staining - FigS4bc - 3_rep - 22.5.23/unc-31_unc-64+pad1267.jpg]

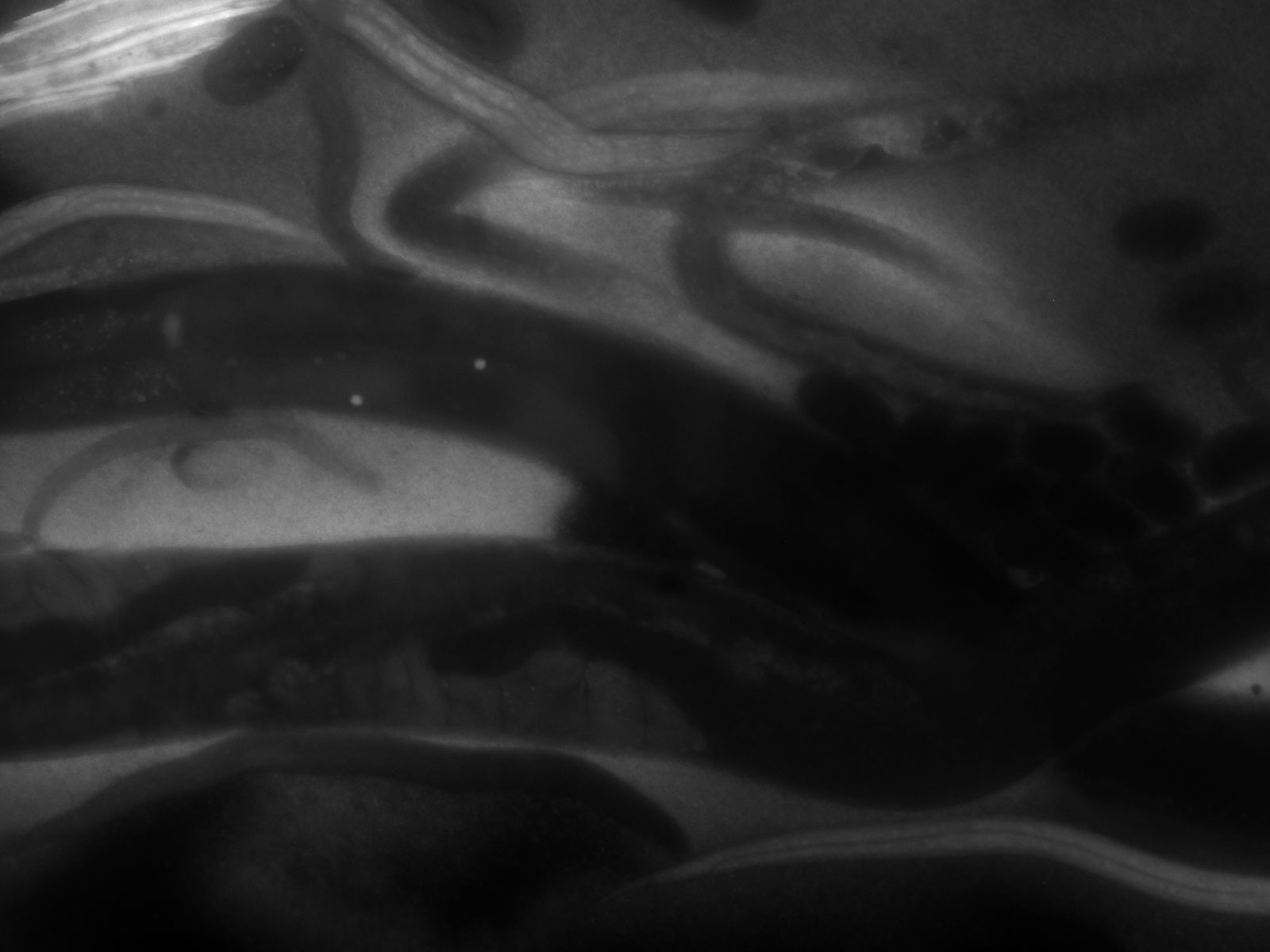

Supplement: S6 File — (ZIP) [file pgen.1011061.s006.zip › Fig.S4B+C - Original files/Fig.S4 RAW data and photos - JPEG/syto12 staining - FigS4bc - 3_rep - 22.5.23/unc-31_unc-64+pad1268.jpg]

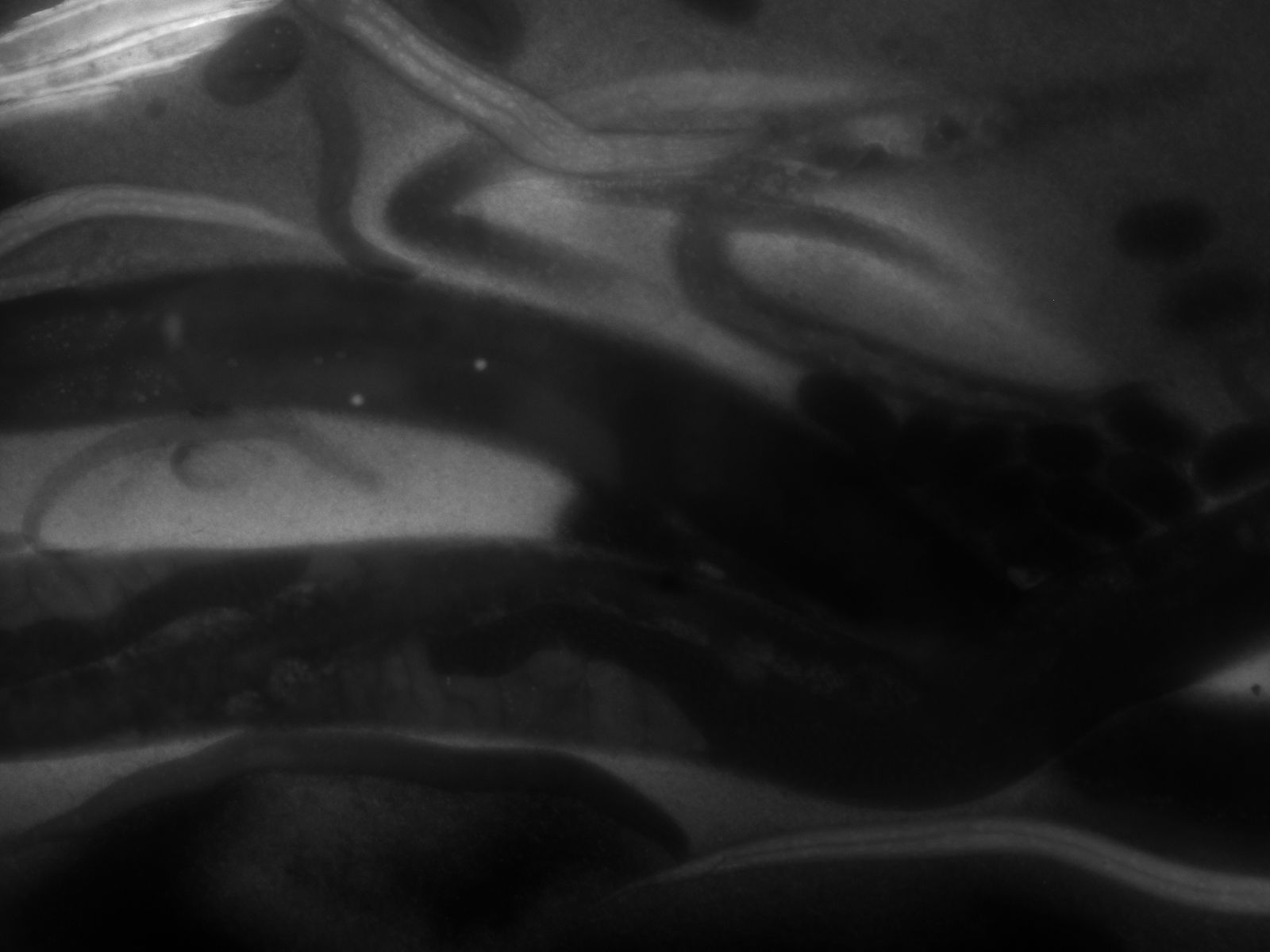

Supplement: S6 File — (ZIP) [file pgen.1011061.s006.zip › Fig.S4B+C - Original files/Fig.S4 RAW data and photos - JPEG/syto12 staining - FigS4bc - 3_rep - 22.5.23/unc-31_unc-64+pad1269.jpg]

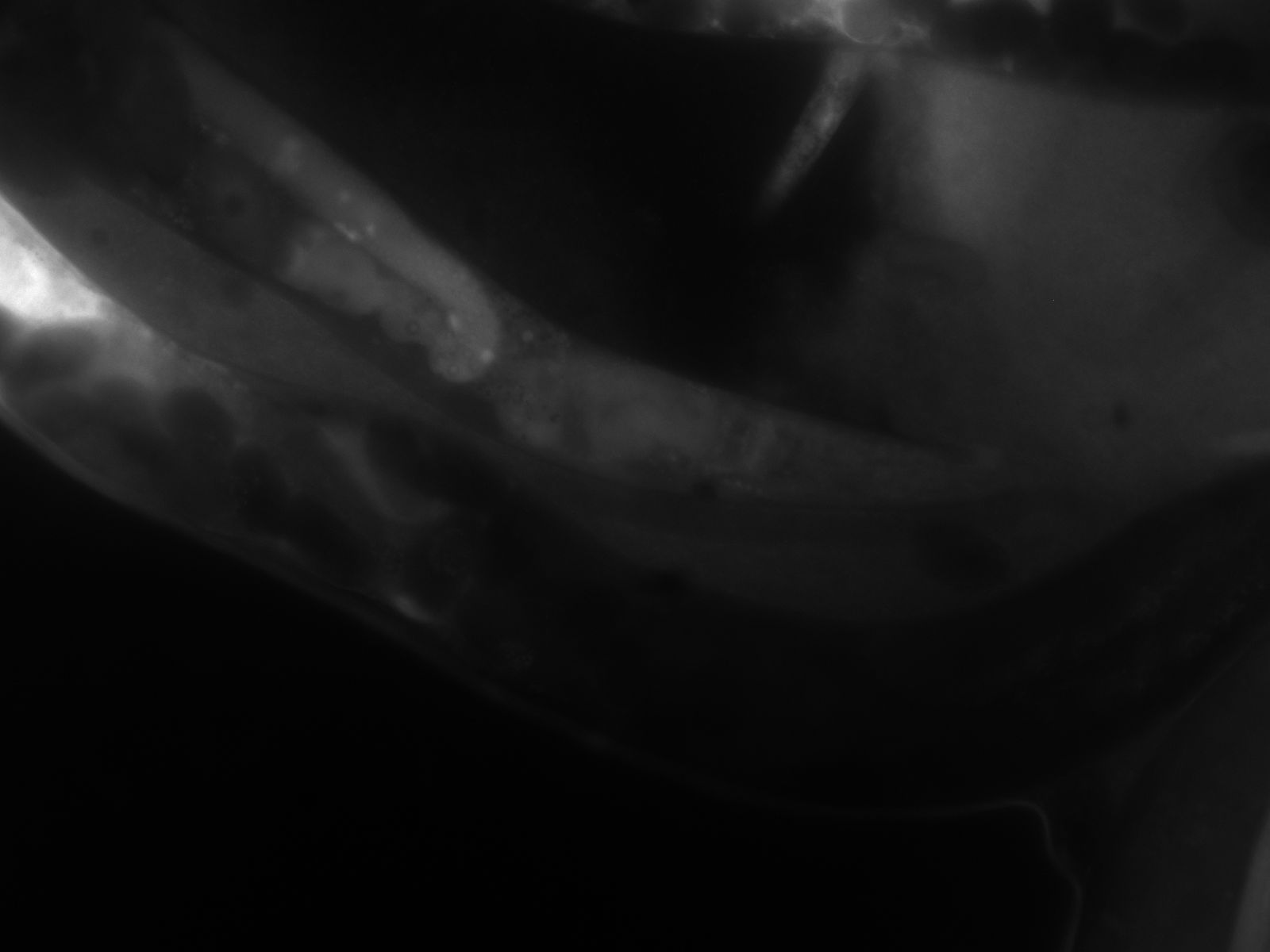

Supplement: S6 File — (ZIP) [file pgen.1011061.s006.zip › Fig.S4B+C - Original files/Fig.S4 RAW data and photos - JPEG/syto12 staining - FigS4bc - 3_rep - 22.5.23/unc-31_unc-64+tfg-170.jpg]

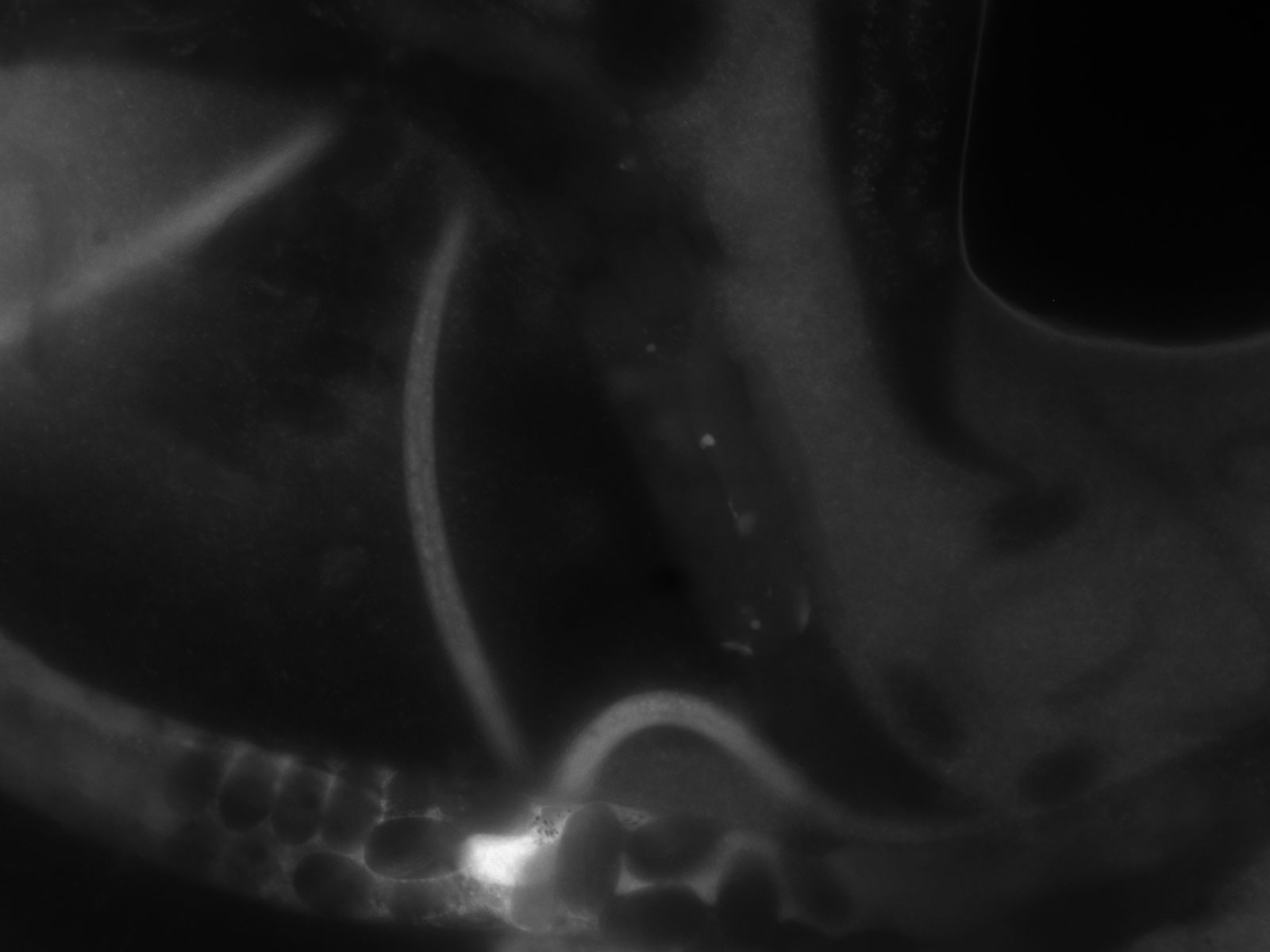

Supplement: S6 File — (ZIP) [file pgen.1011061.s006.zip › Fig.S4B+C - Original files/Fig.S4 RAW data and photos - JPEG/syto12 staining - FigS4bc - 3_rep - 22.5.23/unc-31_unc-64+tfg-171.jpg]

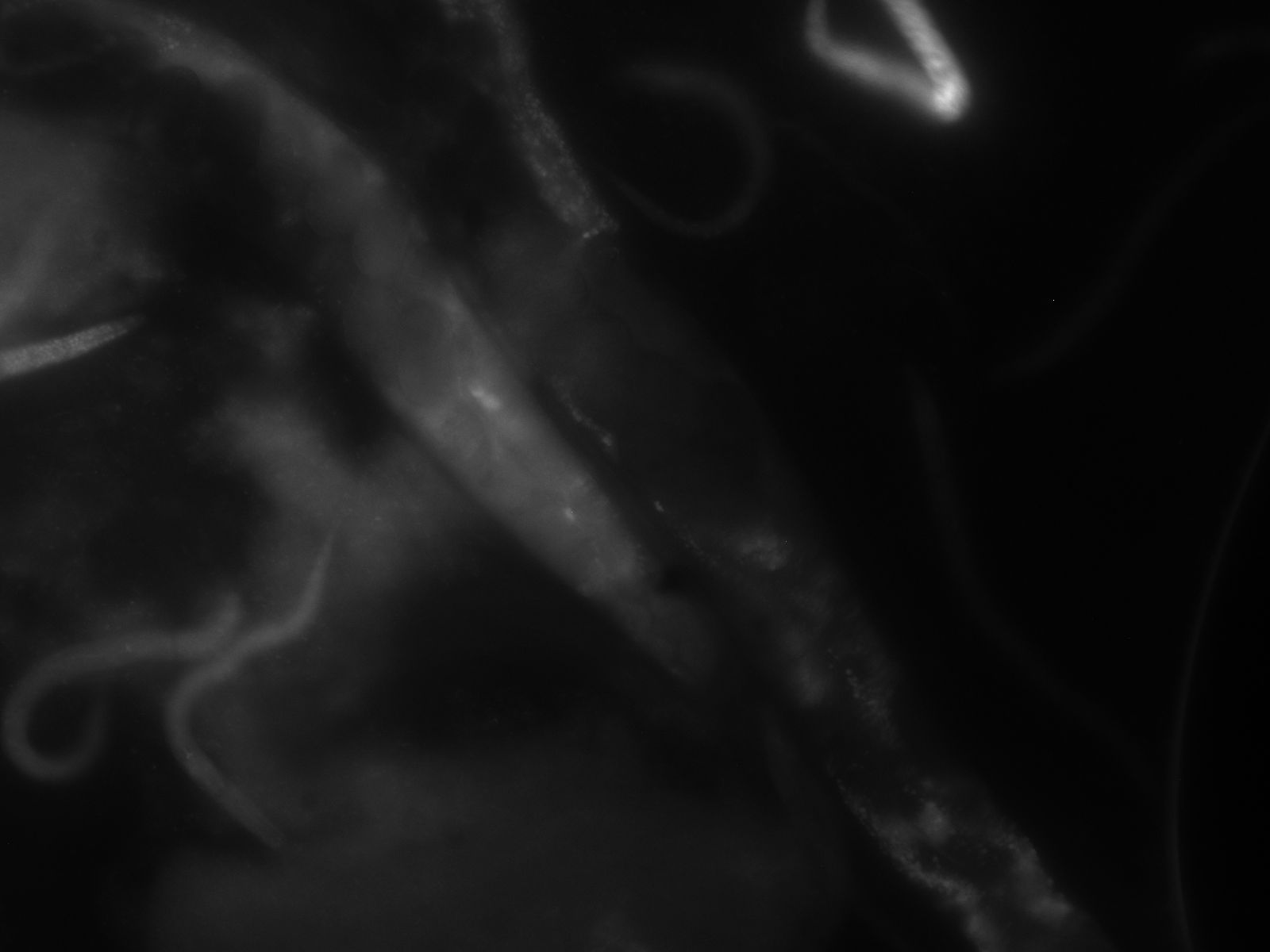

Supplement: S6 File — (ZIP) [file pgen.1011061.s006.zip › Fig.S4B+C - Original files/Fig.S4 RAW data and photos - JPEG/syto12 staining - FigS4bc - 1_rep - 14.5.23/unc-13+pad12280.jpg]

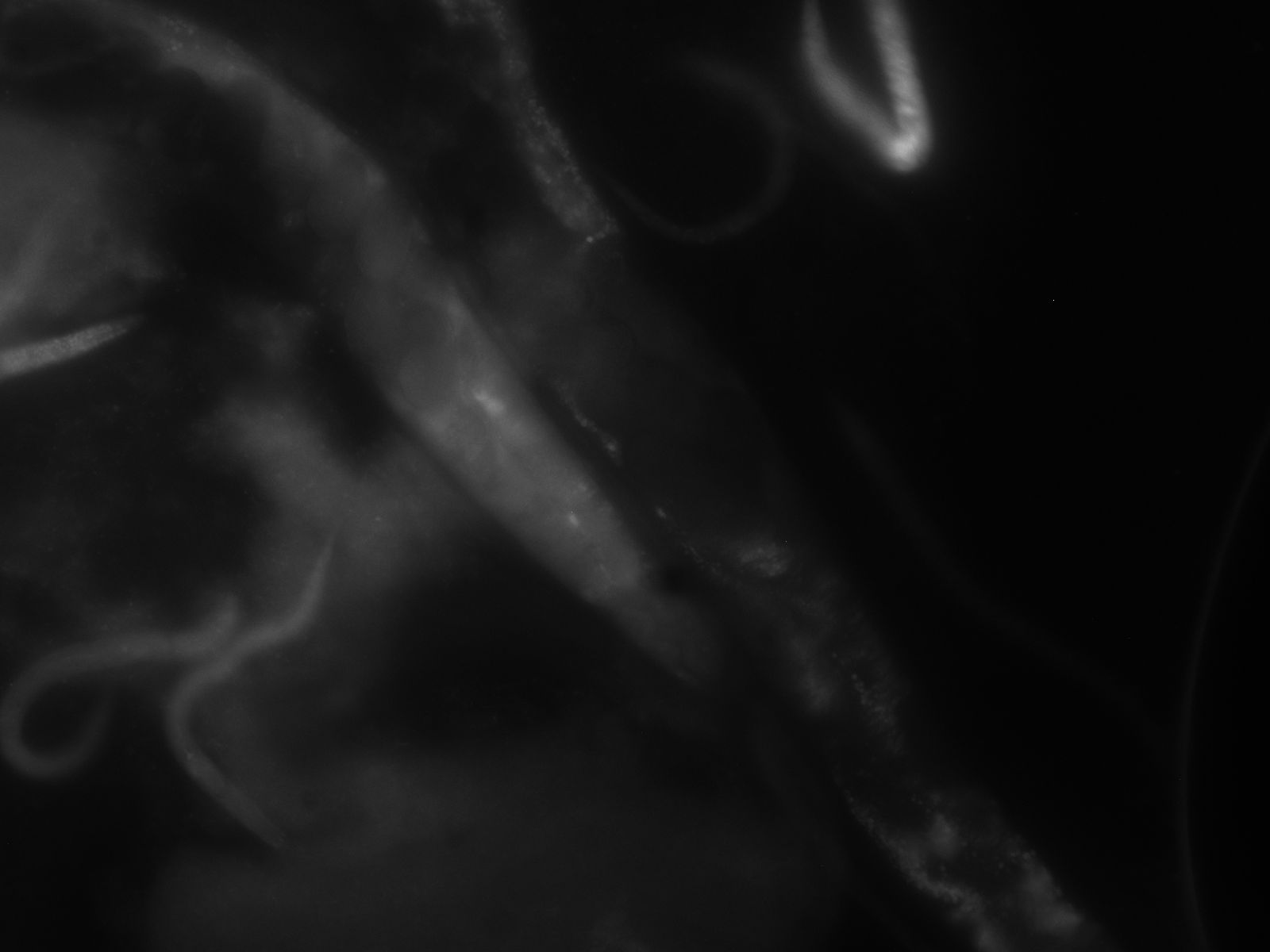

Supplement: S6 File — (ZIP) [file pgen.1011061.s006.zip › Fig.S4B+C - Original files/Fig.S4 RAW data and photos - JPEG/syto12 staining - FigS4bc - 1_rep - 14.5.23/unc-13+pad12281.jpg]

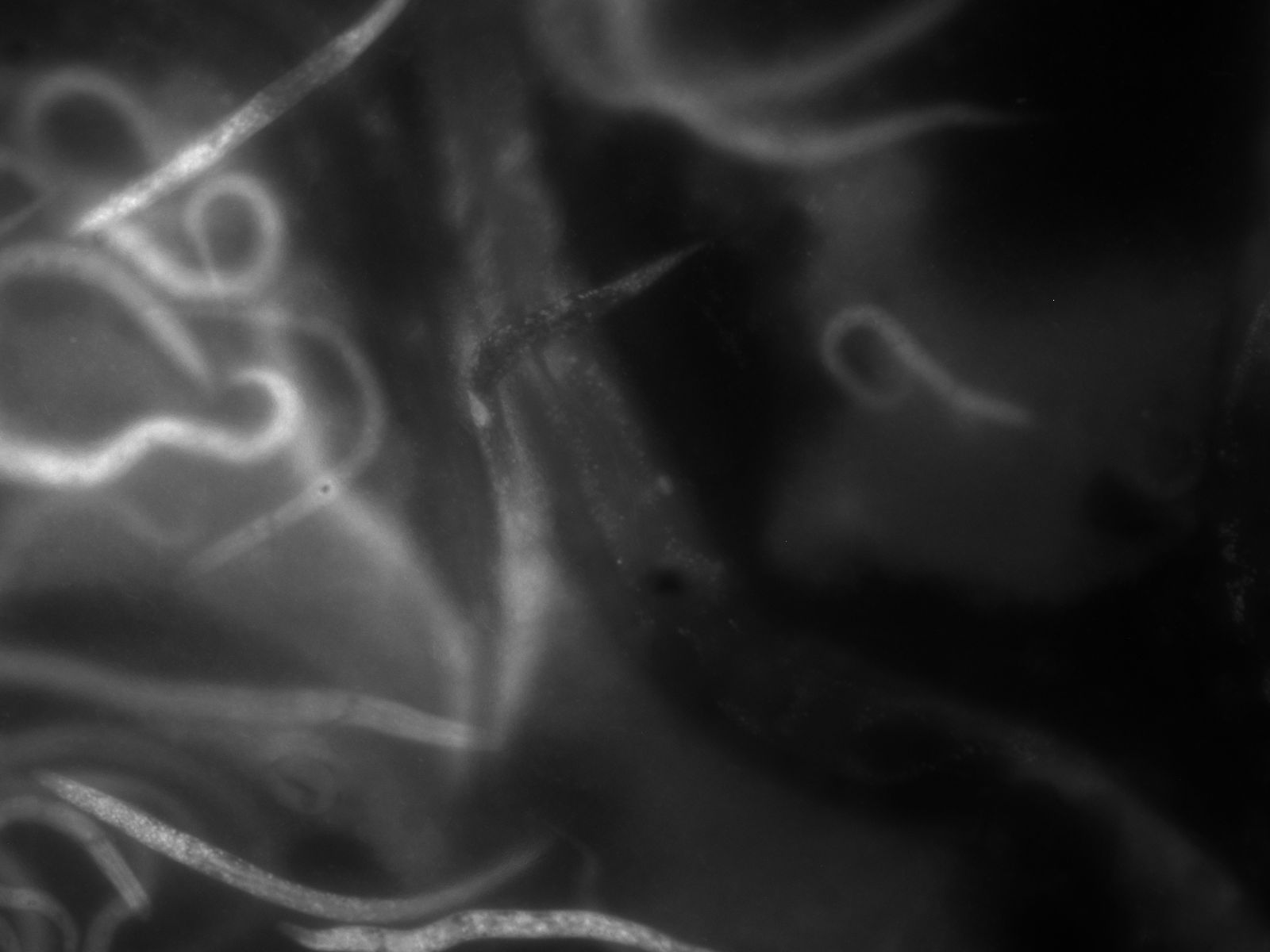

Supplement: S6 File — (ZIP) [file pgen.1011061.s006.zip › Fig.S4B+C - Original files/Fig.S4 RAW data and photos - JPEG/syto12 staining - FigS4bc - 1_rep - 14.5.23/unc-13+pad12282.jpg]

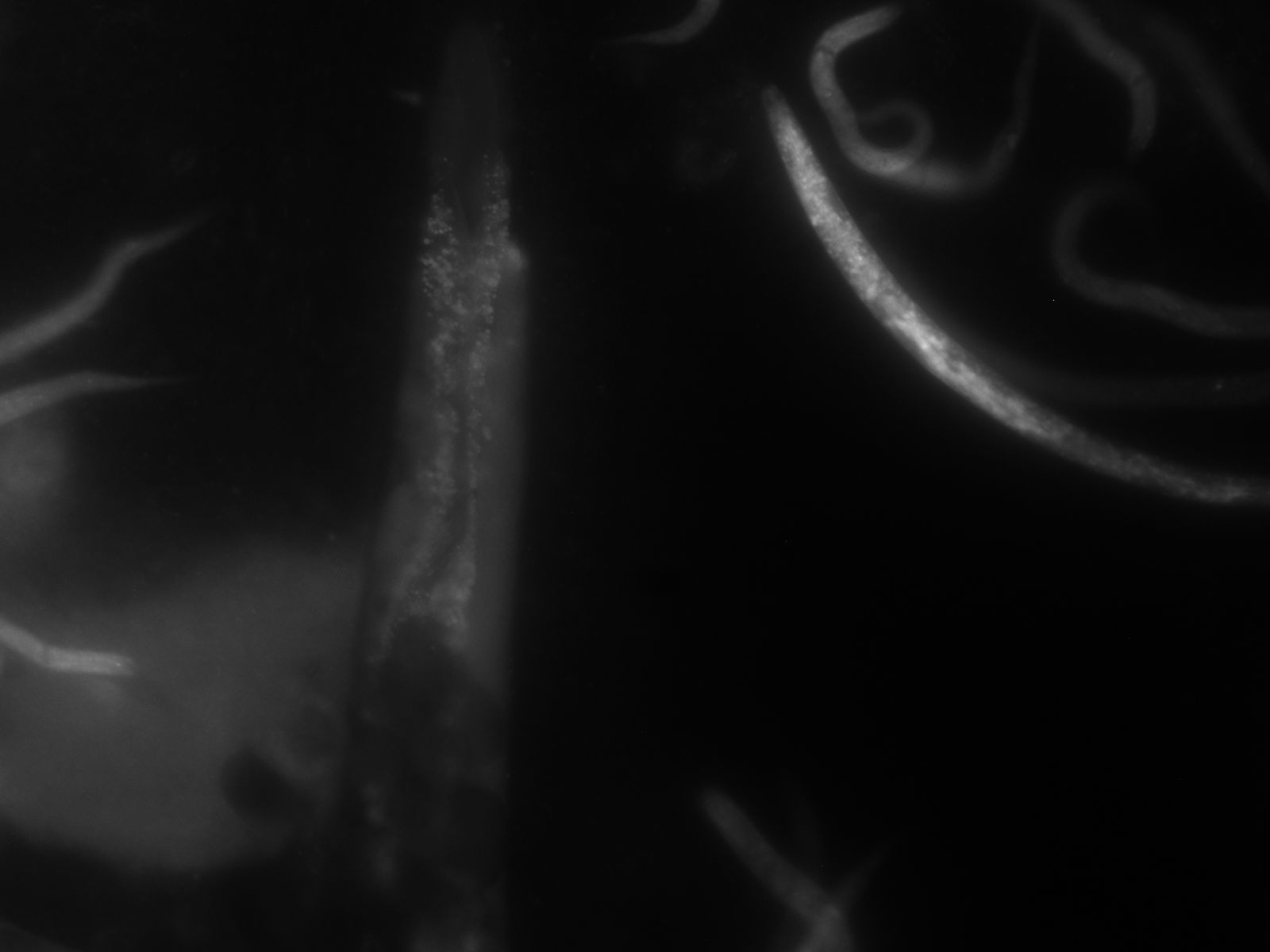

Supplement: S6 File — (ZIP) [file pgen.1011061.s006.zip › Fig.S4B+C - Original files/Fig.S4 RAW data and photos - JPEG/syto12 staining - FigS4bc - 1_rep - 14.5.23/unc-13+pad12283.jpg]

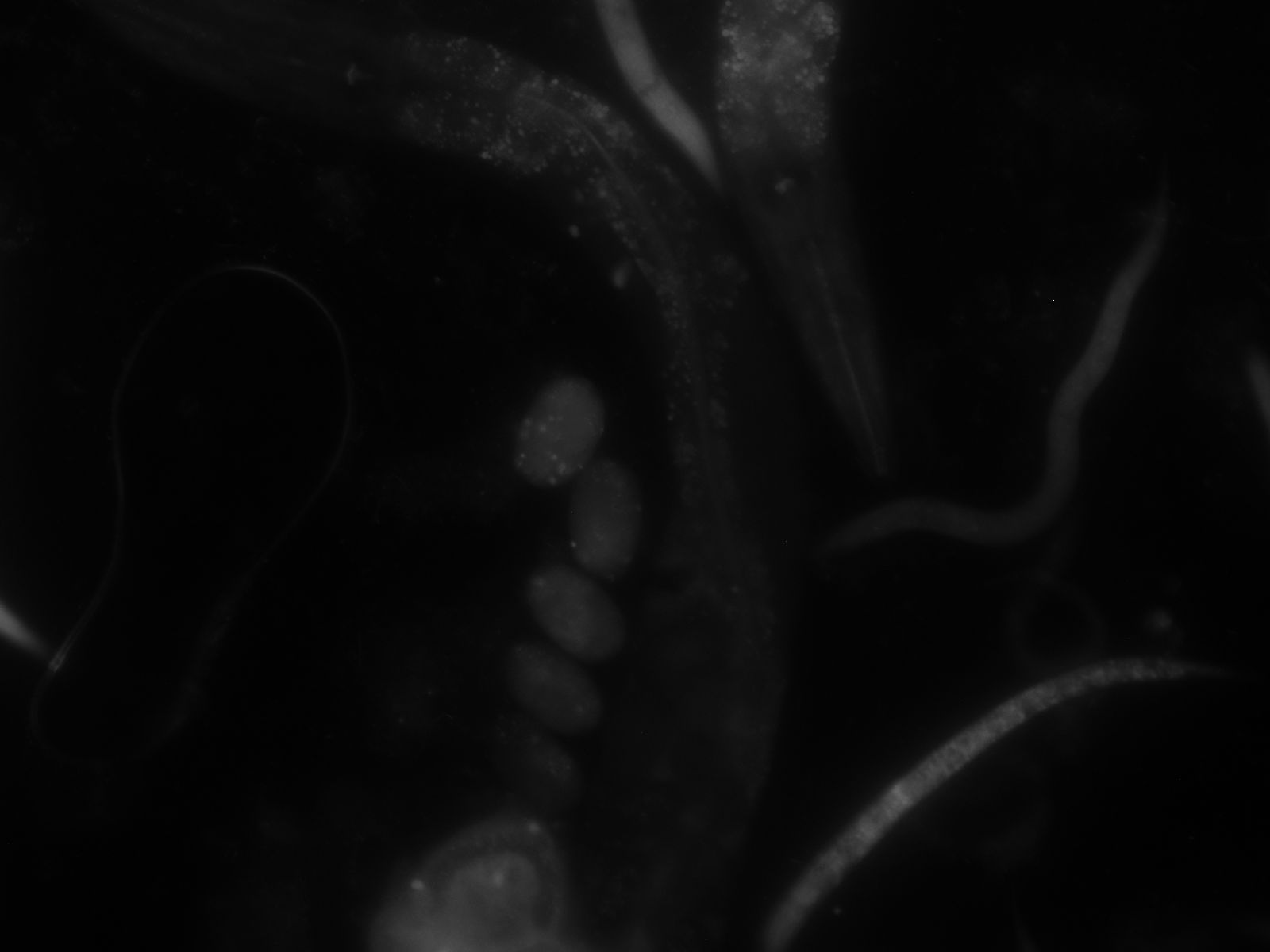

Supplement: S6 File — (ZIP) [file pgen.1011061.s006.zip › Fig.S4B+C - Original files/Fig.S4 RAW data and photos - JPEG/syto12 staining - FigS4bc - 1_rep - 14.5.23/unc-13+pad12284.jpg]

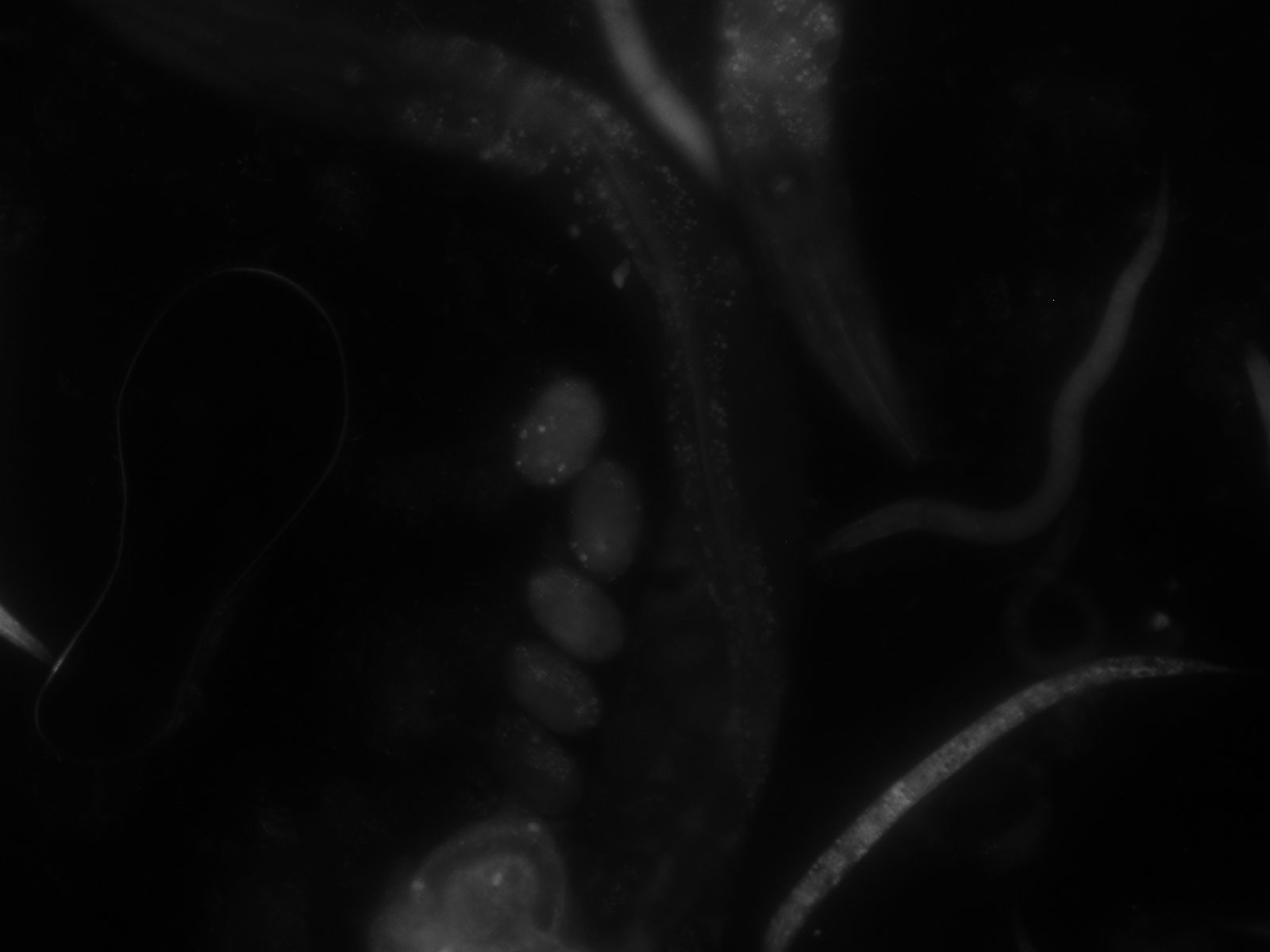

Supplement: S6 File — (ZIP) [file pgen.1011061.s006.zip › Fig.S4B+C - Original files/Fig.S4 RAW data and photos - JPEG/syto12 staining - FigS4bc - 1_rep - 14.5.23/unc-13+pad12285.jpg]

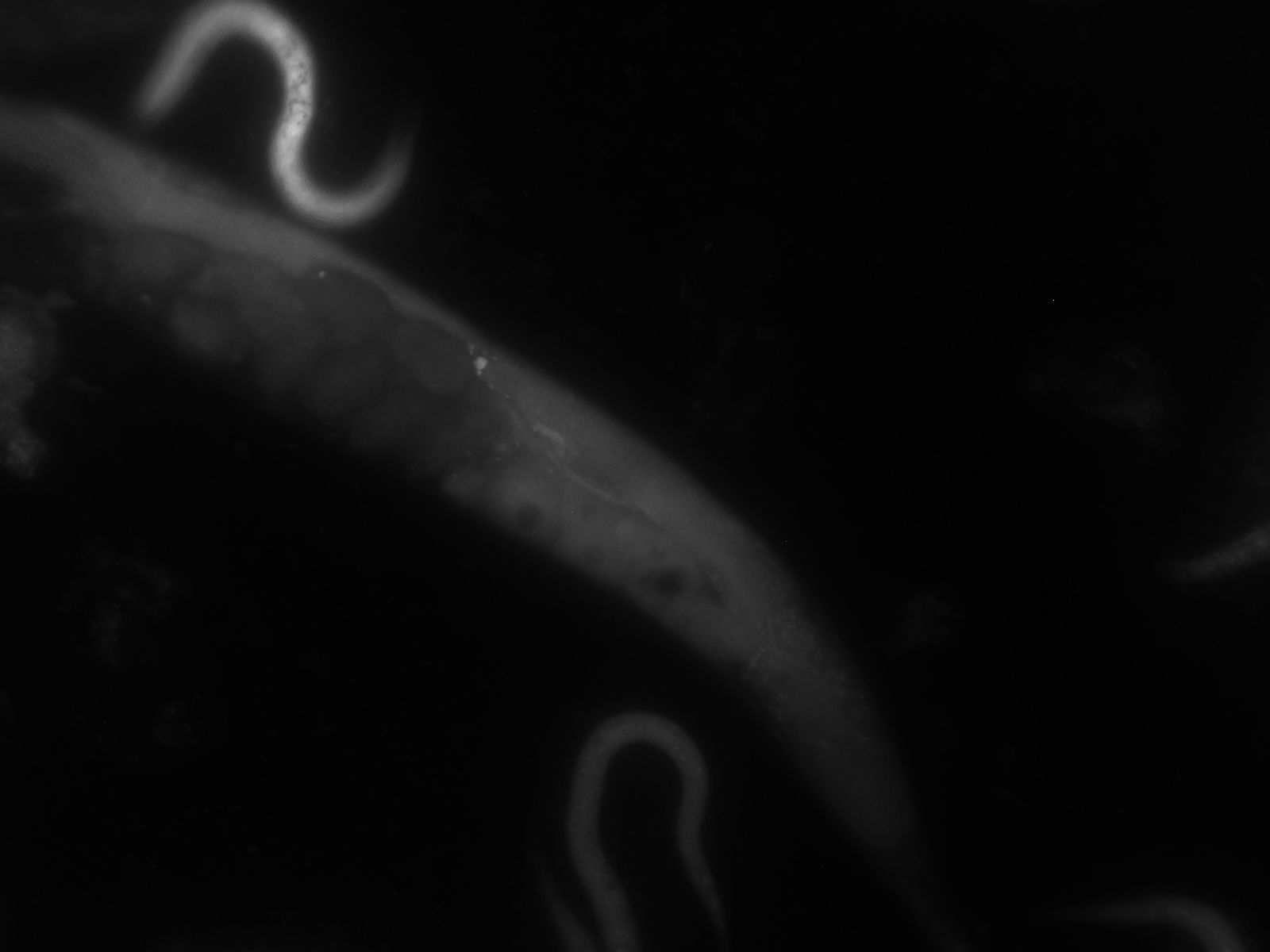

Supplement: S6 File — (ZIP) [file pgen.1011061.s006.zip › Fig.S4B+C - Original files/Fig.S4 RAW data and photos - JPEG/syto12 staining - FigS4bc - 1_rep - 14.5.23/unc-13+pad12286.jpg]

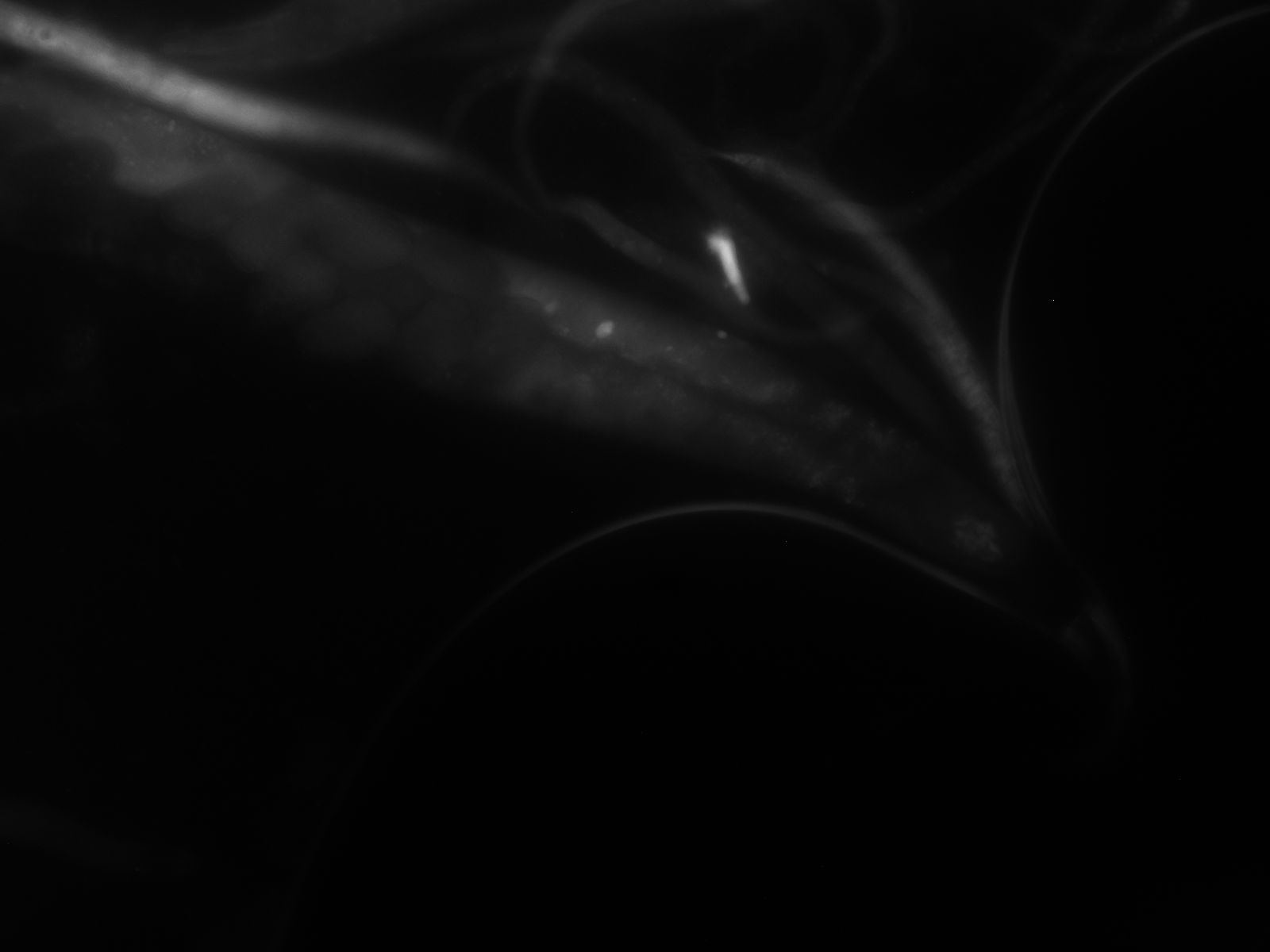

Supplement: S6 File — (ZIP) [file pgen.1011061.s006.zip › Fig.S4B+C - Original files/Fig.S4 RAW data and photos - JPEG/syto12 staining - FigS4bc - 1_rep - 14.5.23/unc-13+pad12287.jpg]

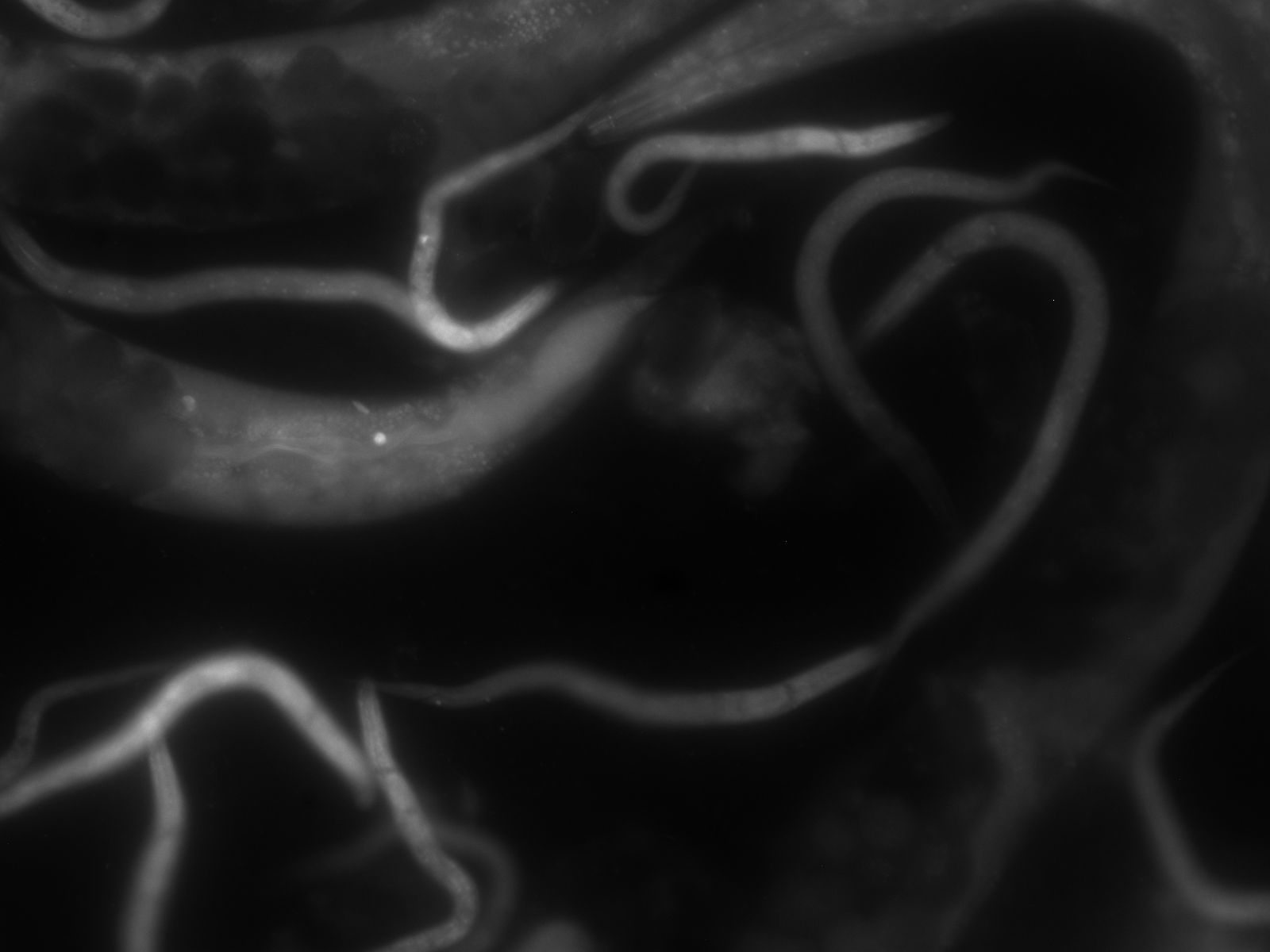

Supplement: S6 File — (ZIP) [file pgen.1011061.s006.zip › Fig.S4B+C - Original files/Fig.S4 RAW data and photos - JPEG/syto12 staining - FigS4bc - 1_rep - 14.5.23/unc-13+pad12288.jpg]

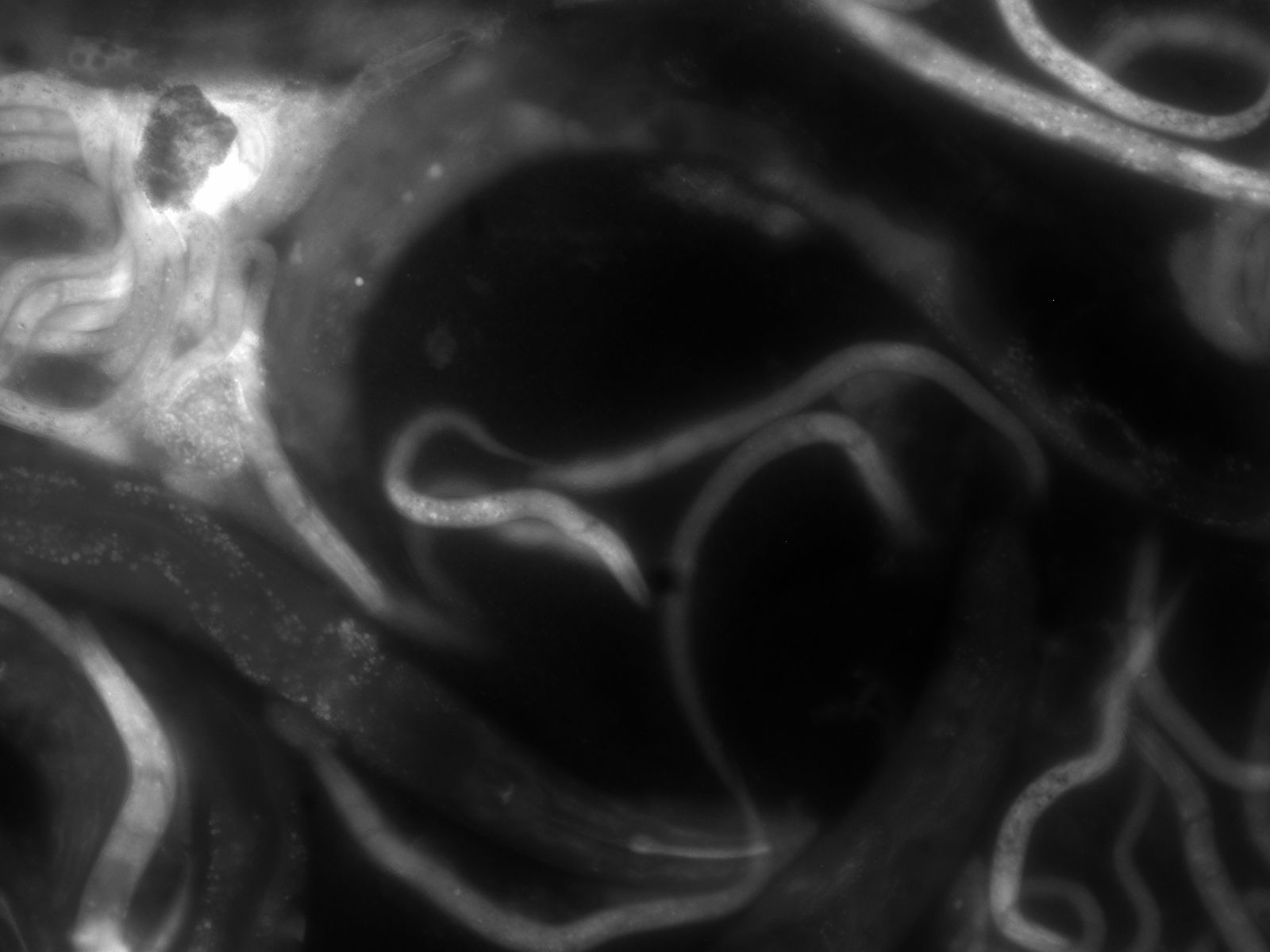

Supplement: S6 File — (ZIP) [file pgen.1011061.s006.zip › Fig.S4B+C - Original files/Fig.S4 RAW data and photos - JPEG/syto12 staining - FigS4bc - 1_rep - 14.5.23/unc-13+pad12289.jpg]

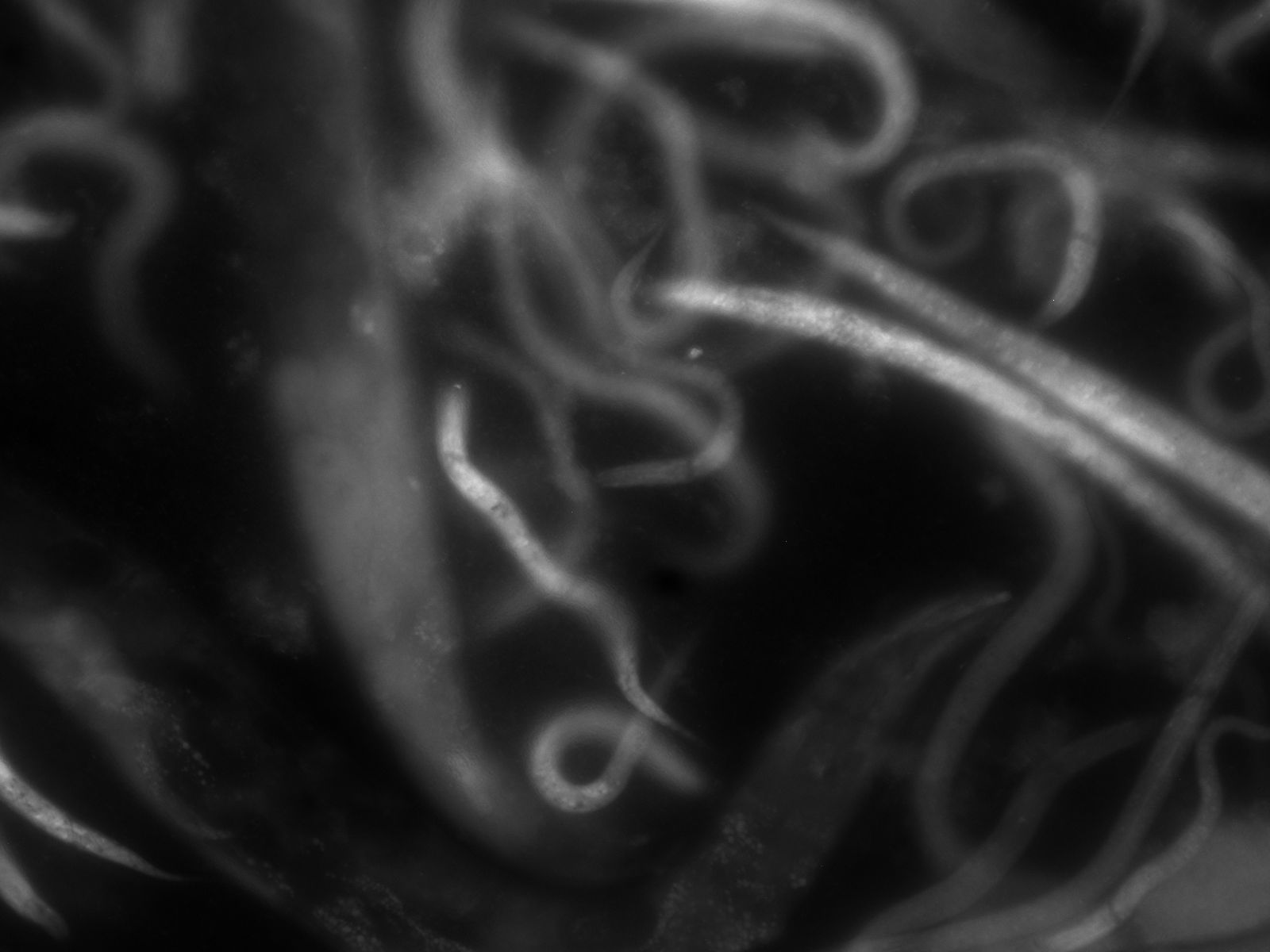

Supplement: S6 File — (ZIP) [file pgen.1011061.s006.zip › Fig.S4B+C - Original files/Fig.S4 RAW data and photos - JPEG/syto12 staining - FigS4bc - 1_rep - 14.5.23/unc-13+pad12290.jpg]

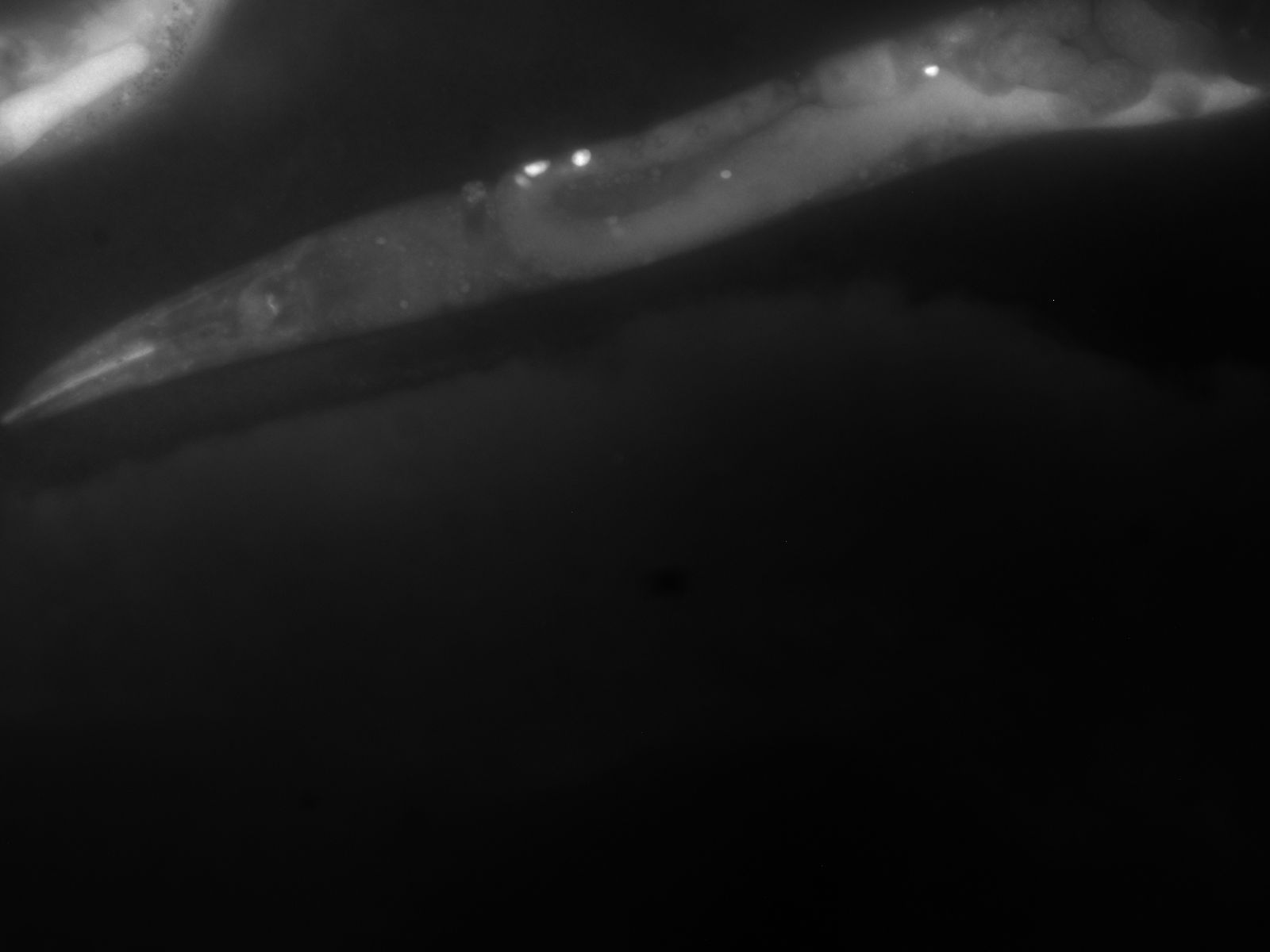

Supplement: S6 File — (ZIP) [file pgen.1011061.s006.zip › Fig.S4B+C - Original files/Fig.S4 RAW data and photos - JPEG/syto12 staining - FigS4bc - 1_rep - 14.5.23/unc-13+tfg-1291.jpg]

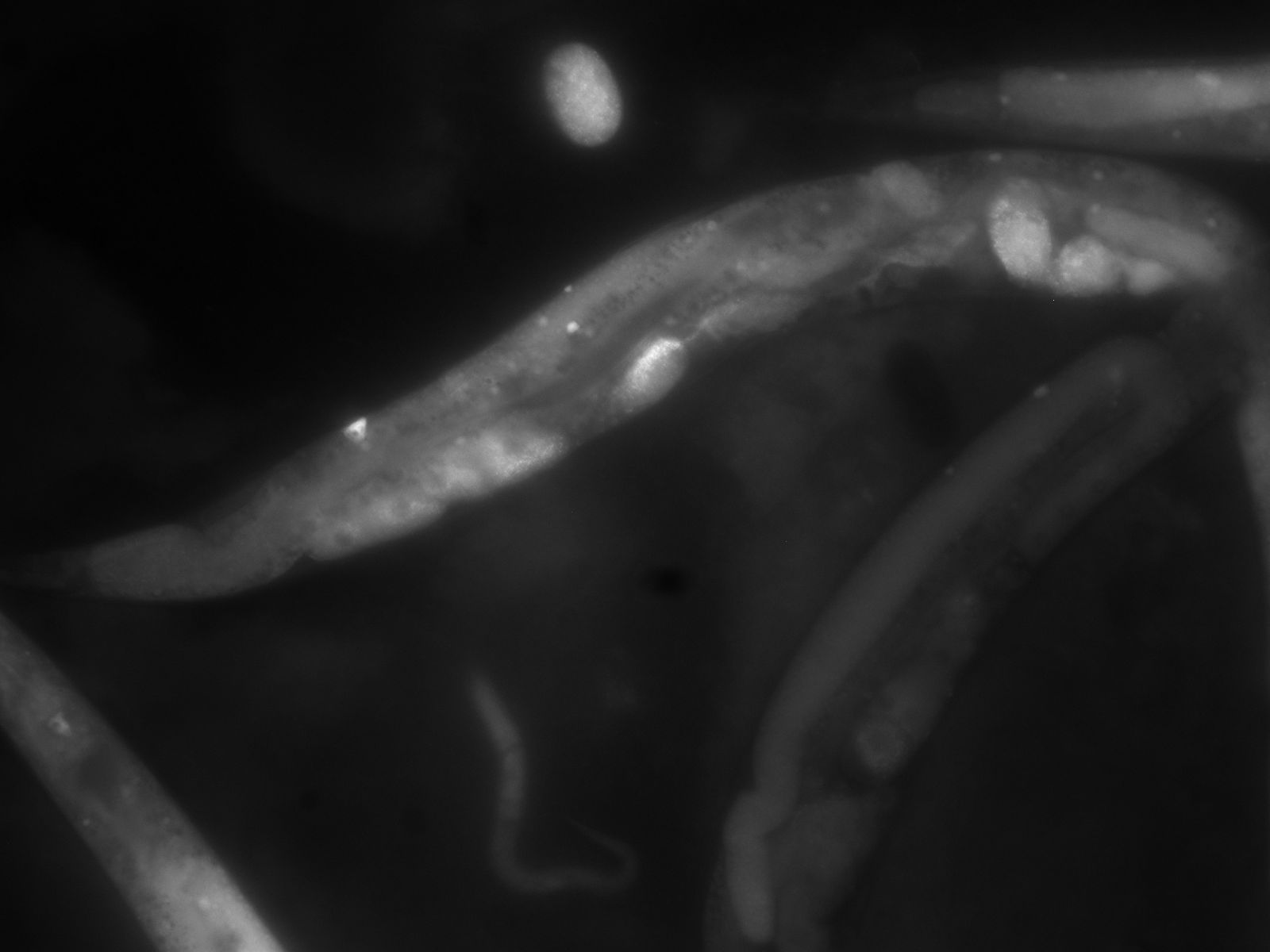

Supplement: S6 File — (ZIP) [file pgen.1011061.s006.zip › Fig.S4B+C - Original files/Fig.S4 RAW data and photos - JPEG/syto12 staining - FigS4bc - 1_rep - 14.5.23/unc-13+tfg-1292.jpg]

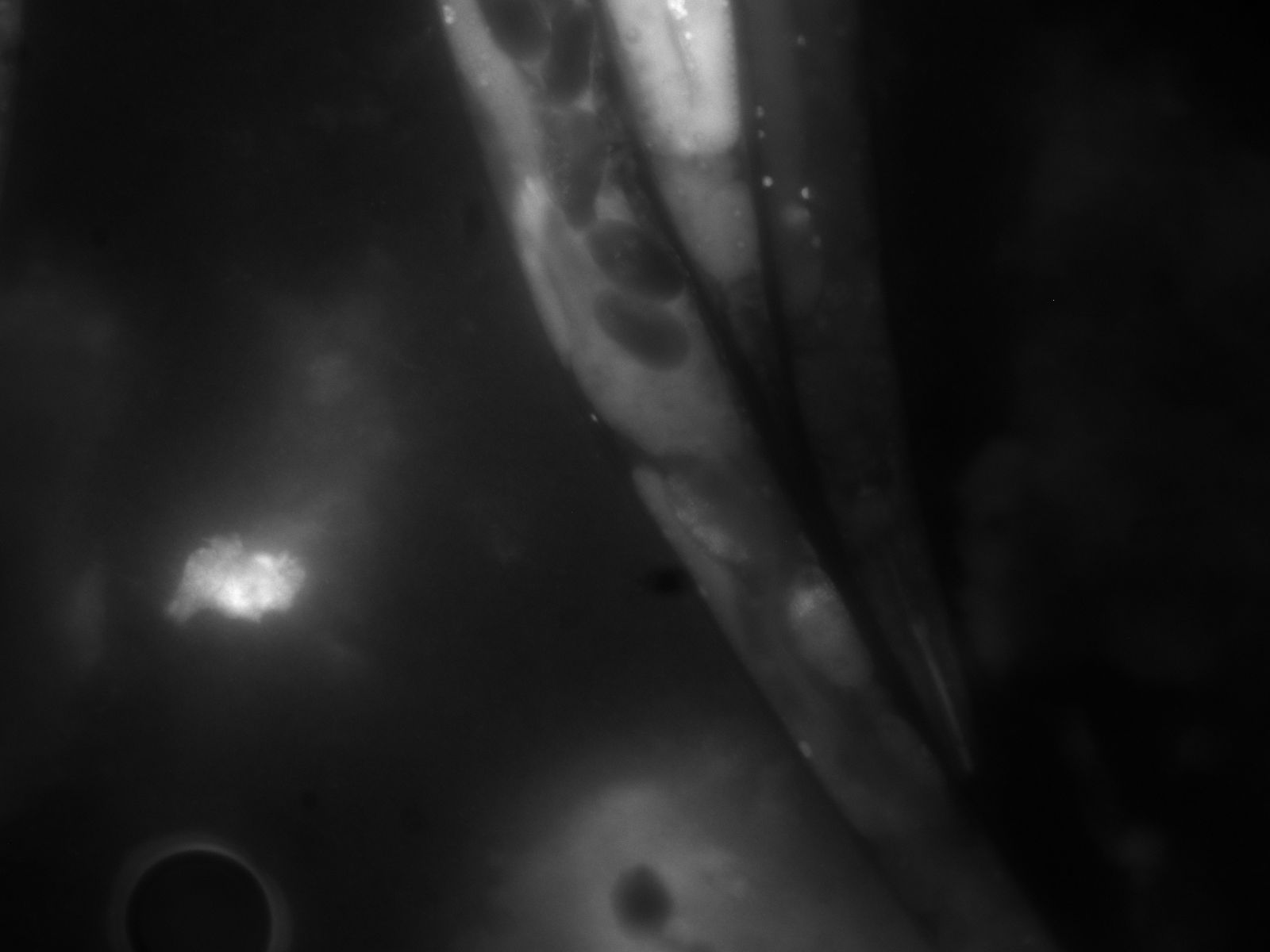

Supplement: S6 File — (ZIP) [file pgen.1011061.s006.zip › Fig.S4B+C - Original files/Fig.S4 RAW data and photos - JPEG/syto12 staining - FigS4bc - 1_rep - 14.5.23/unc-13+tfg-1293.jpg]

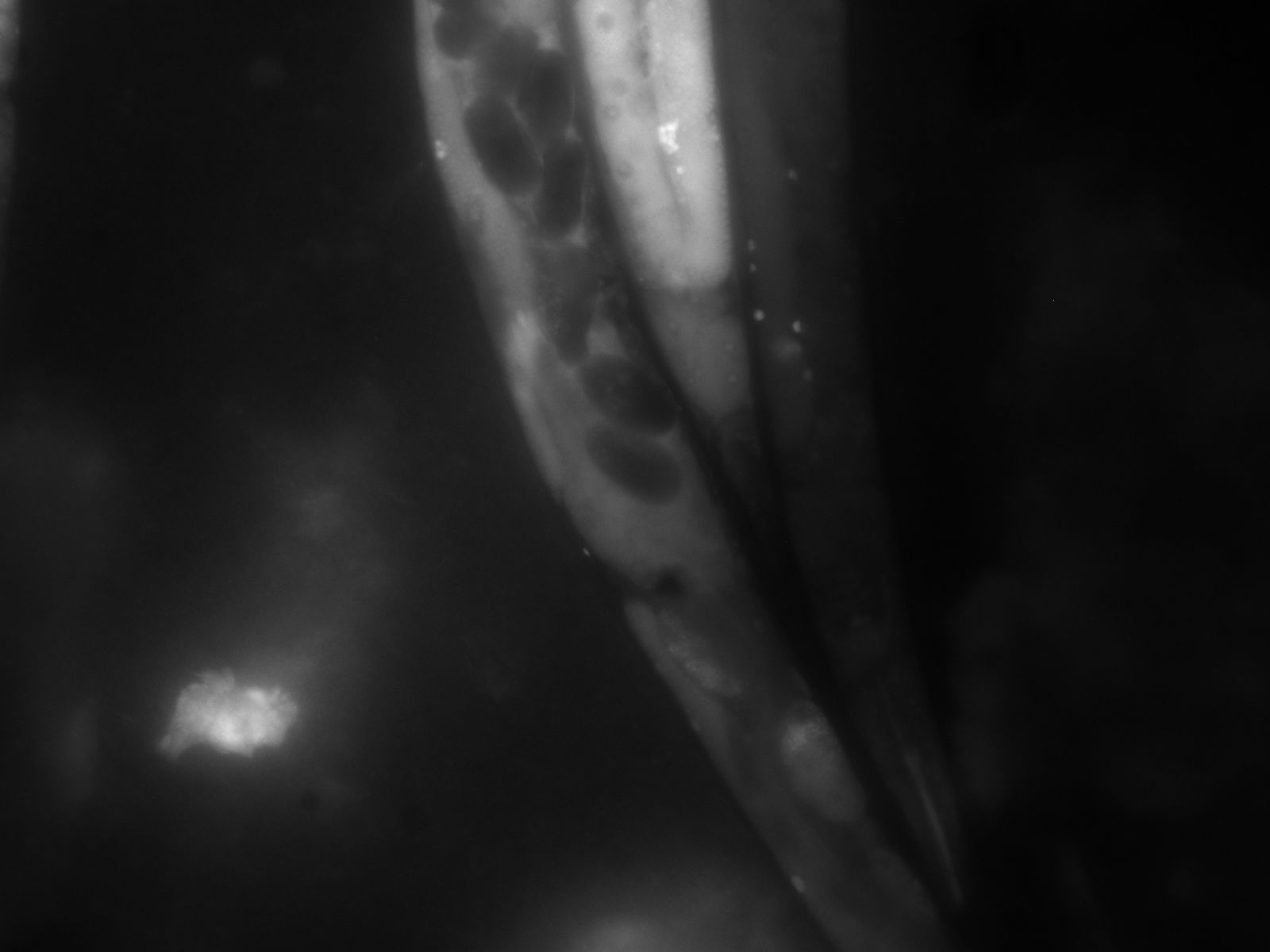

Supplement: S6 File — (ZIP) [file pgen.1011061.s006.zip › Fig.S4B+C - Original files/Fig.S4 RAW data and photos - JPEG/syto12 staining - FigS4bc - 1_rep - 14.5.23/unc-13+tfg-1294.jpg]

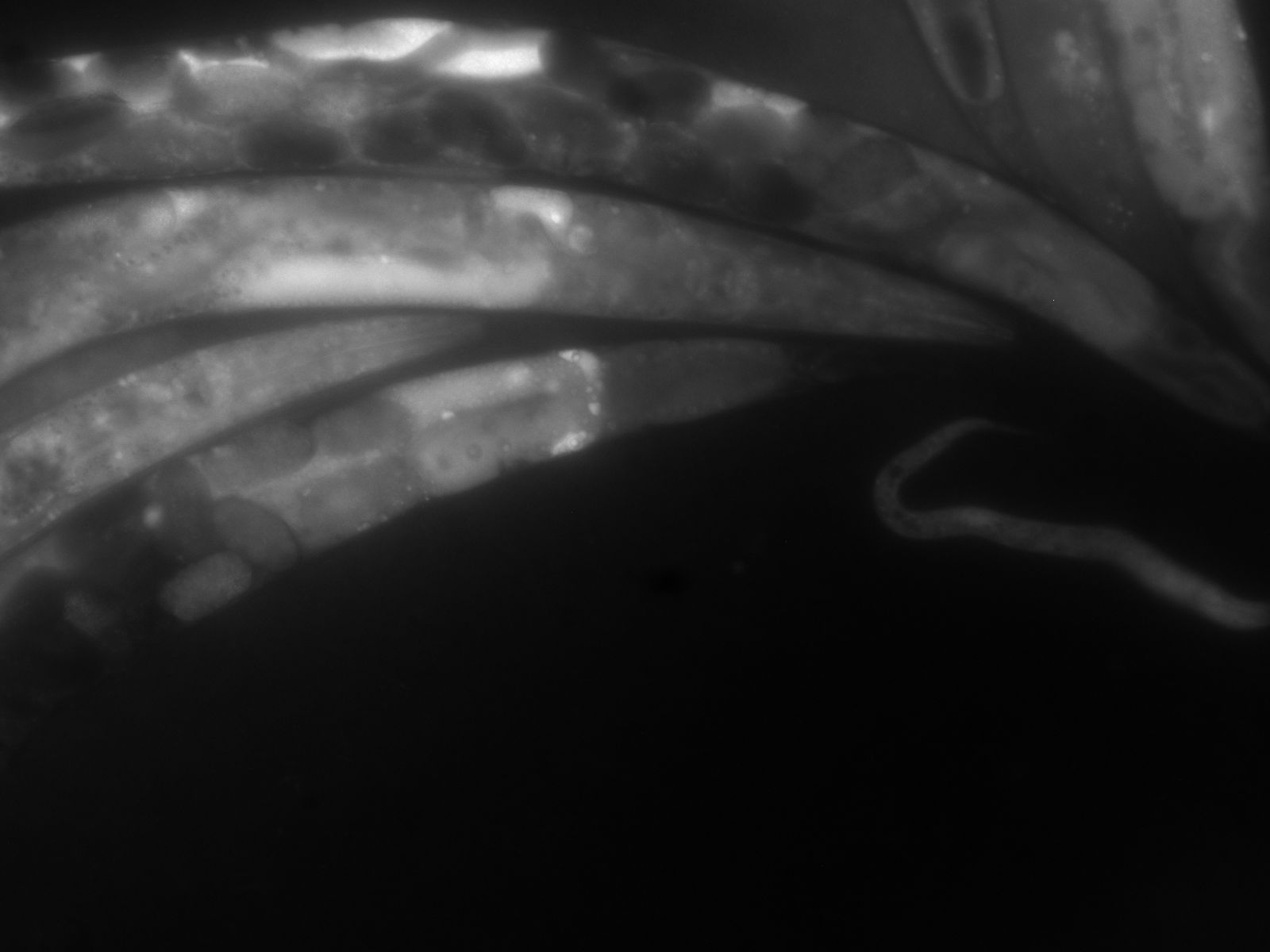

Supplement: S6 File — (ZIP) [file pgen.1011061.s006.zip › Fig.S4B+C - Original files/Fig.S4 RAW data and photos - JPEG/syto12 staining - FigS4bc - 1_rep - 14.5.23/unc-13+tfg-1295.jpg]

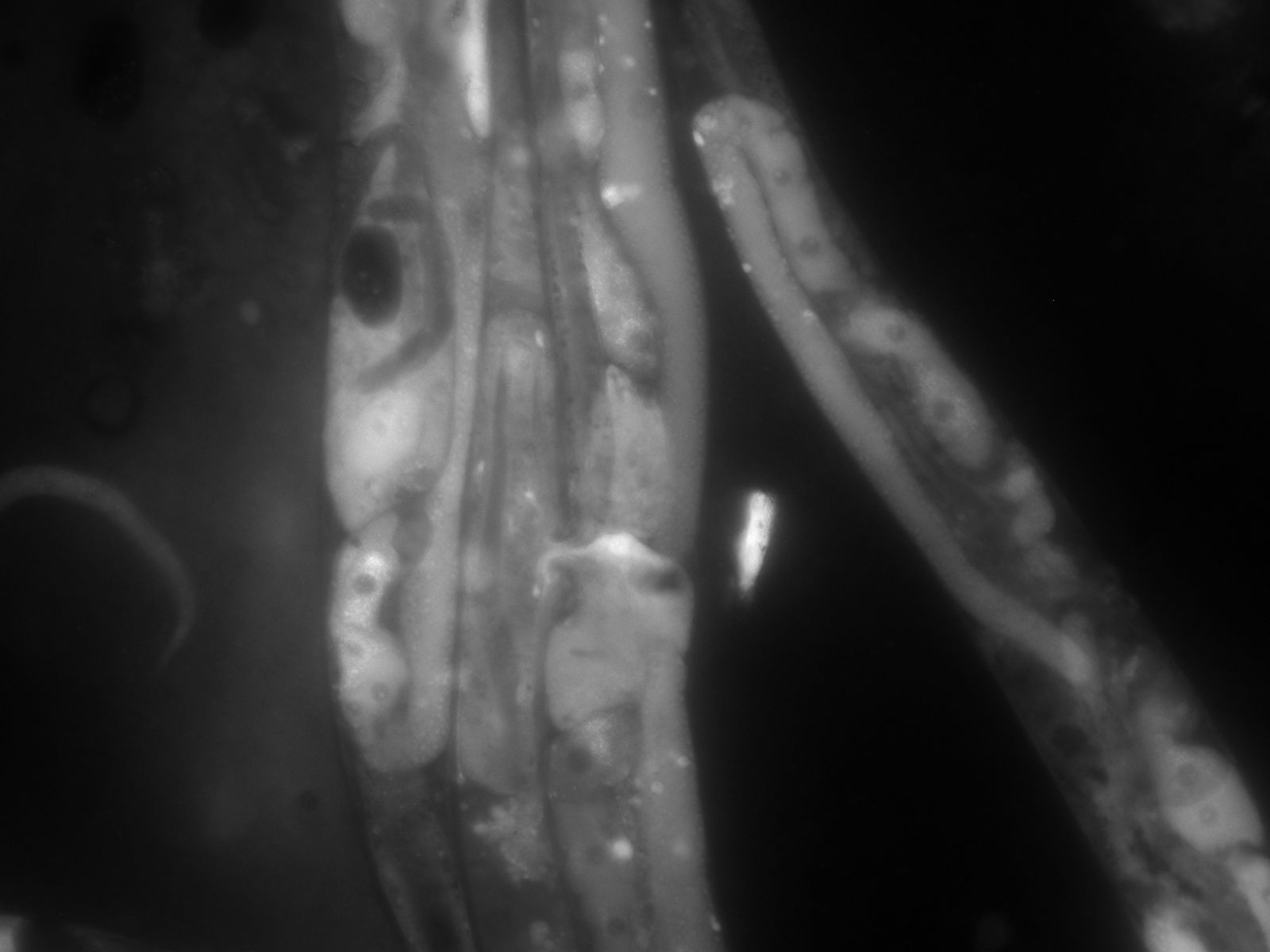

Supplement: S6 File — (ZIP) [file pgen.1011061.s006.zip › Fig.S4B+C - Original files/Fig.S4 RAW data and photos - JPEG/syto12 staining - FigS4bc - 1_rep - 14.5.23/unc-13+tfg-1296.jpg]

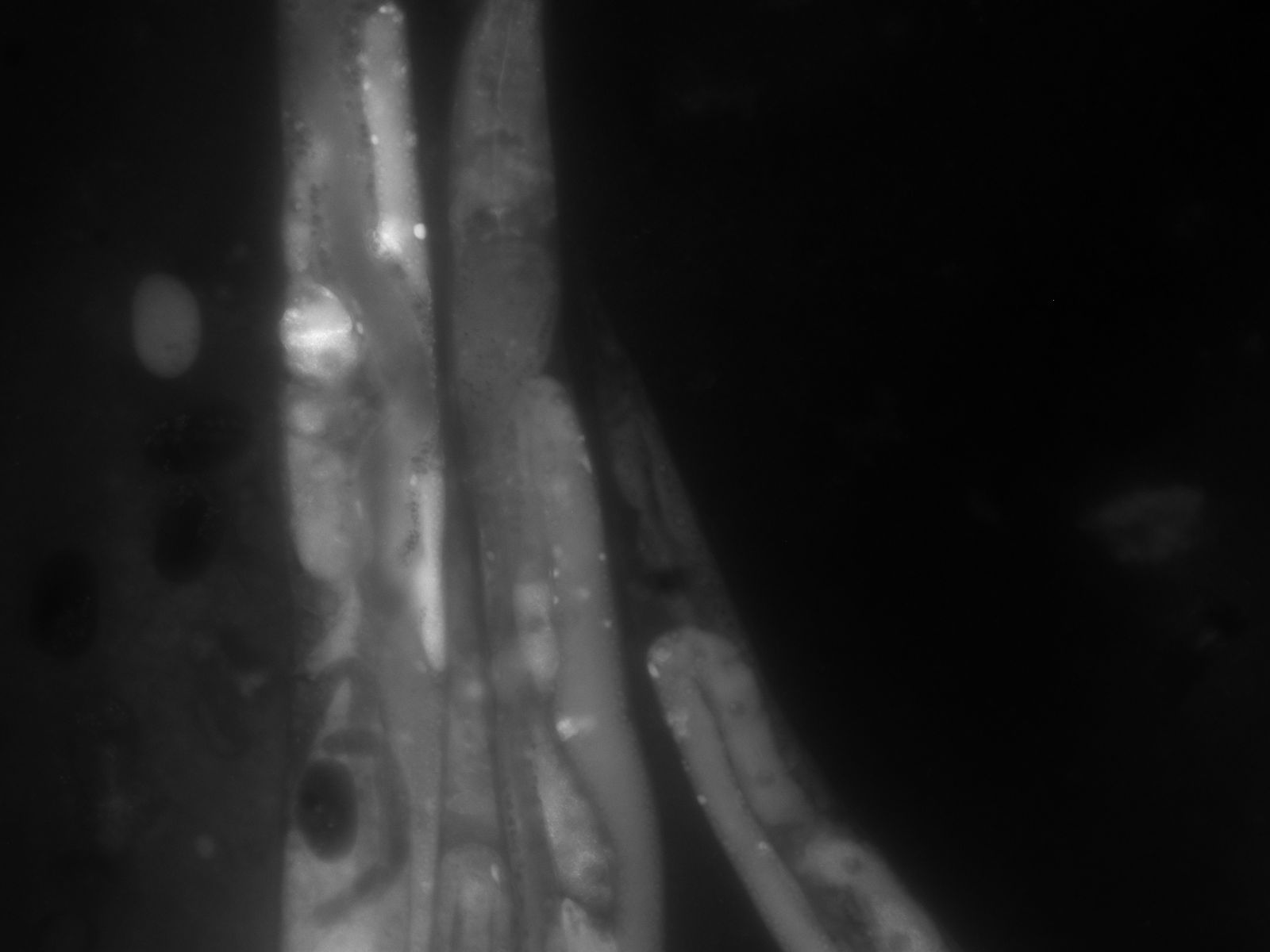

Supplement: S6 File — (ZIP) [file pgen.1011061.s006.zip › Fig.S4B+C - Original files/Fig.S4 RAW data and photos - JPEG/syto12 staining - FigS4bc - 1_rep - 14.5.23/unc-13+tfg-1297.jpg]

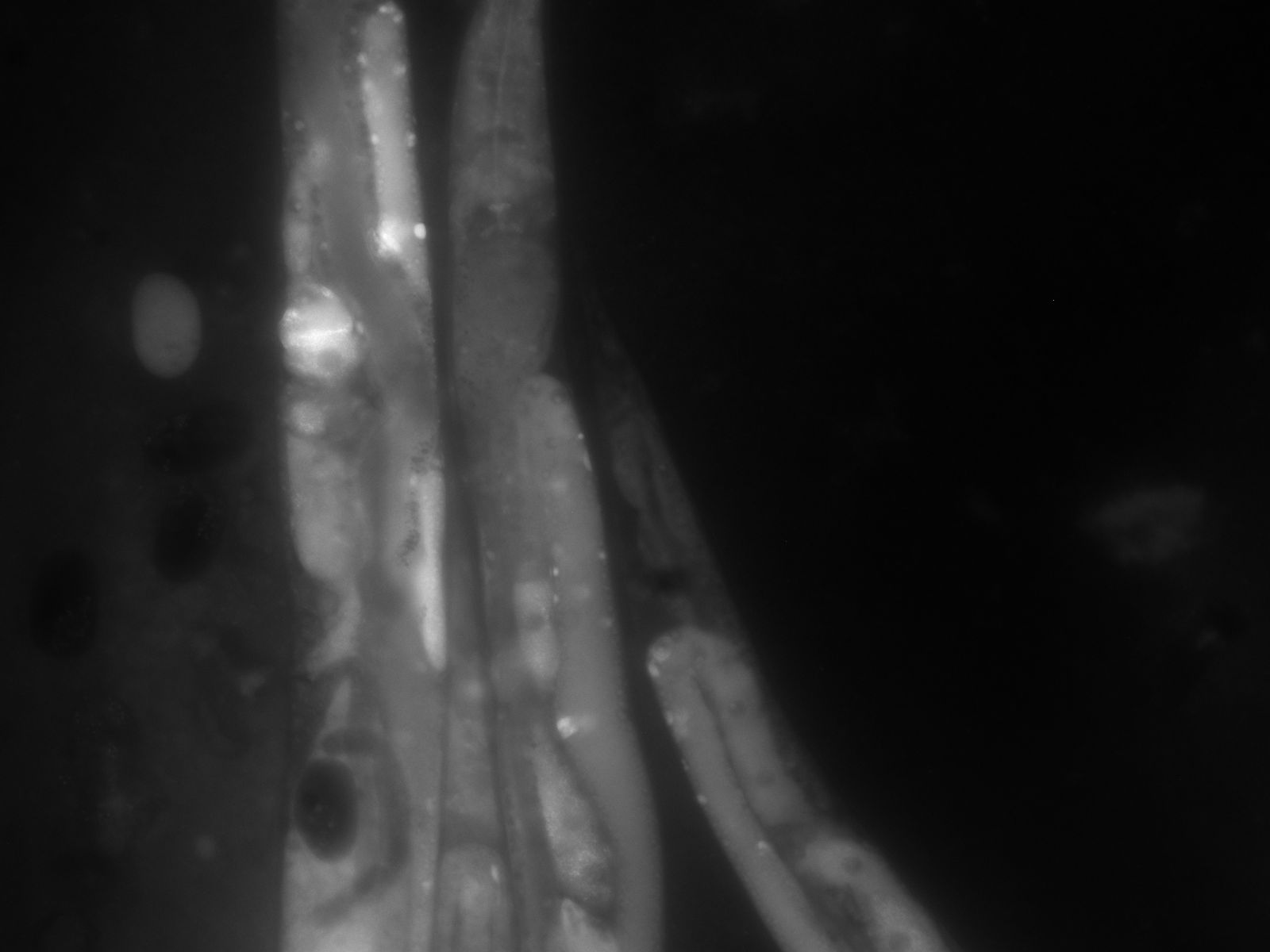

Supplement: S6 File — (ZIP) [file pgen.1011061.s006.zip › Fig.S4B+C - Original files/Fig.S4 RAW data and photos - JPEG/syto12 staining - FigS4bc - 1_rep - 14.5.23/unc-13+tfg-1298.jpg]

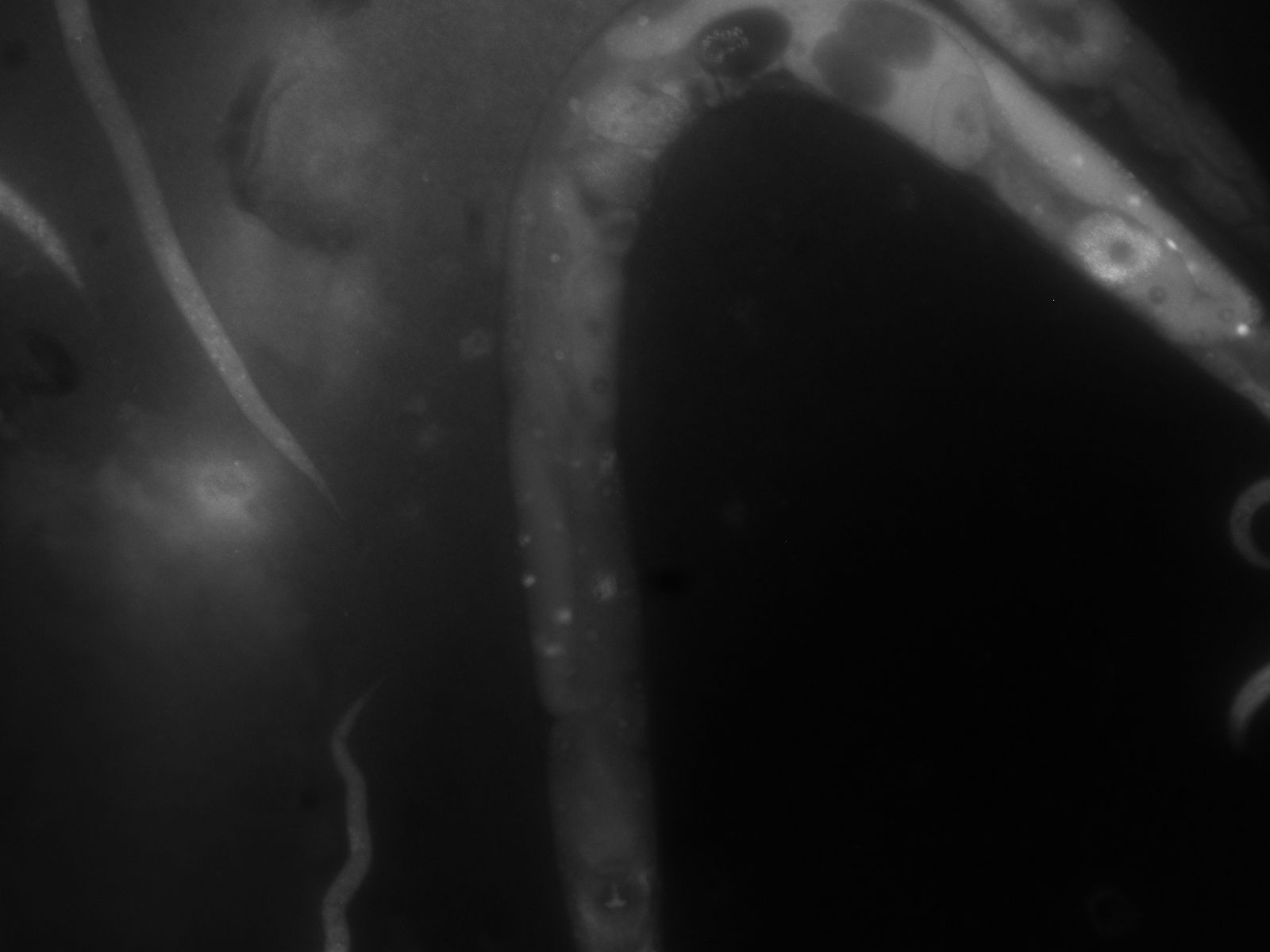

Supplement: S6 File — (ZIP) [file pgen.1011061.s006.zip › Fig.S4B+C - Original files/Fig.S4 RAW data and photos - JPEG/syto12 staining - FigS4bc - 1_rep - 14.5.23/unc-13+tfg-1299.jpg]

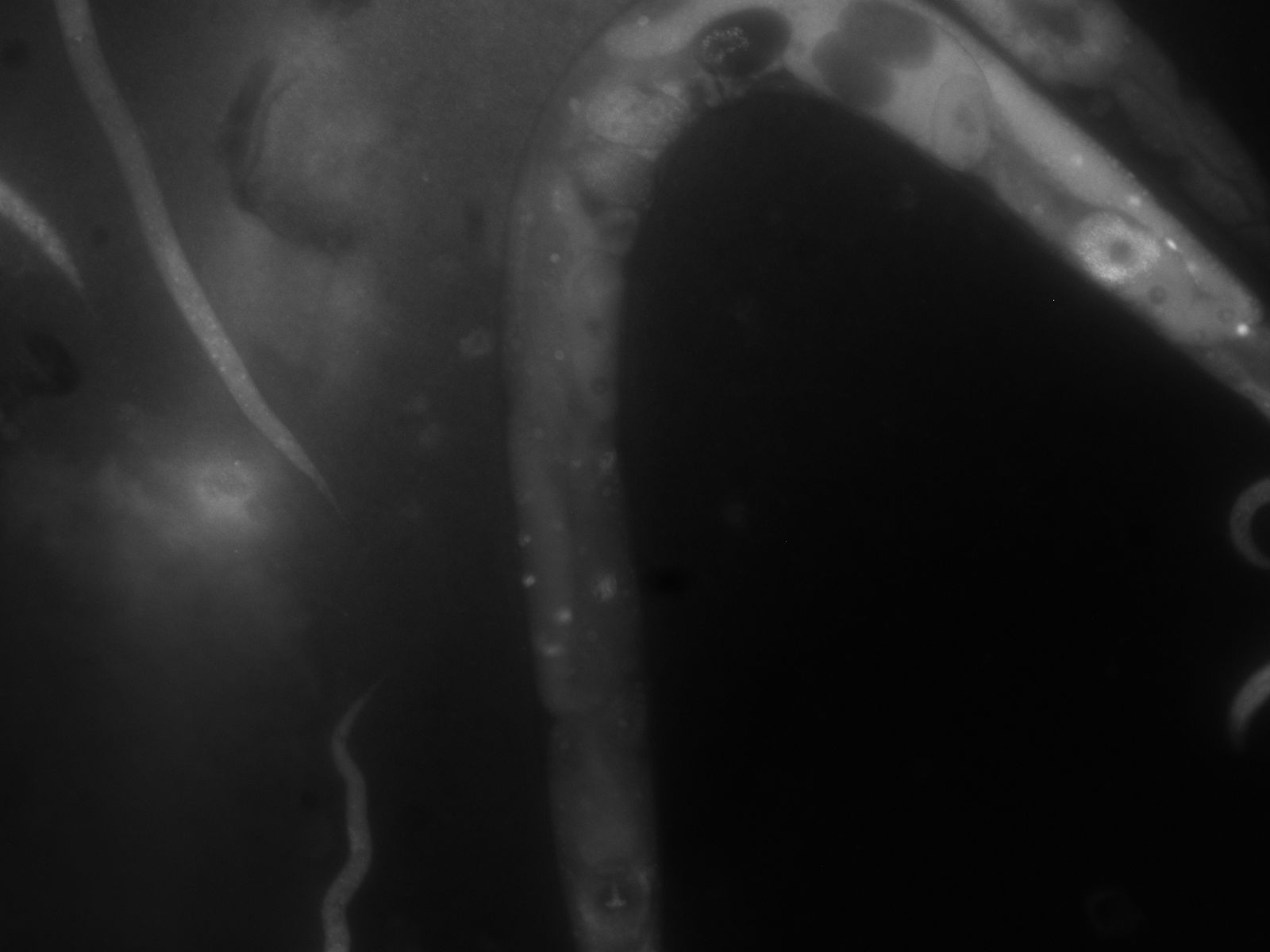

Supplement: S6 File — (ZIP) [file pgen.1011061.s006.zip › Fig.S4B+C - Original files/Fig.S4 RAW data and photos - JPEG/syto12 staining - FigS4bc - 1_rep - 14.5.23/unc-13+tfg-1300.jpg]

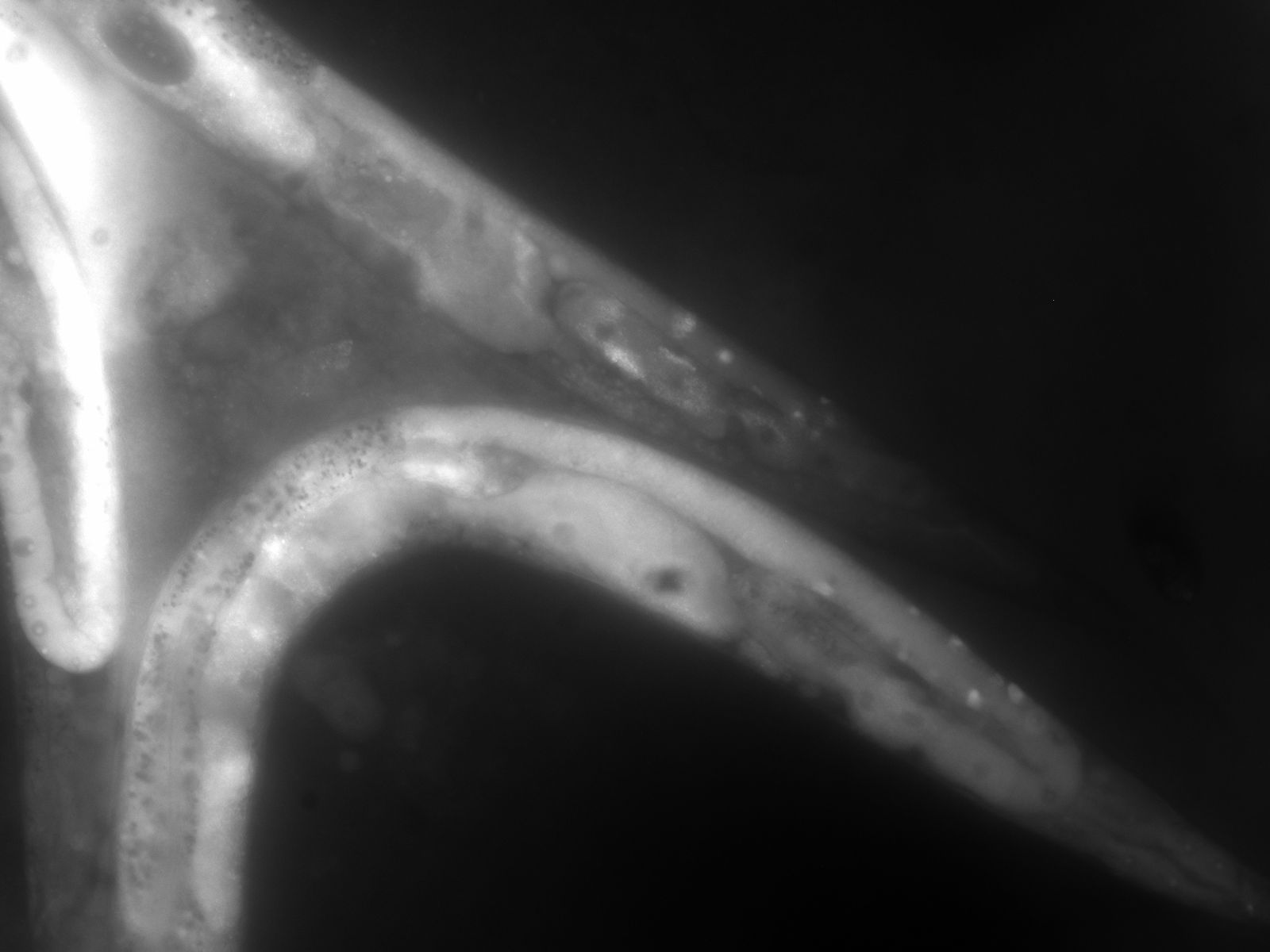

Supplement: S6 File — (ZIP) [file pgen.1011061.s006.zip › Fig.S4B+C - Original files/Fig.S4 RAW data and photos - JPEG/syto12 staining - FigS4bc - 1_rep - 14.5.23/unc-13+tfg-1301.jpg]

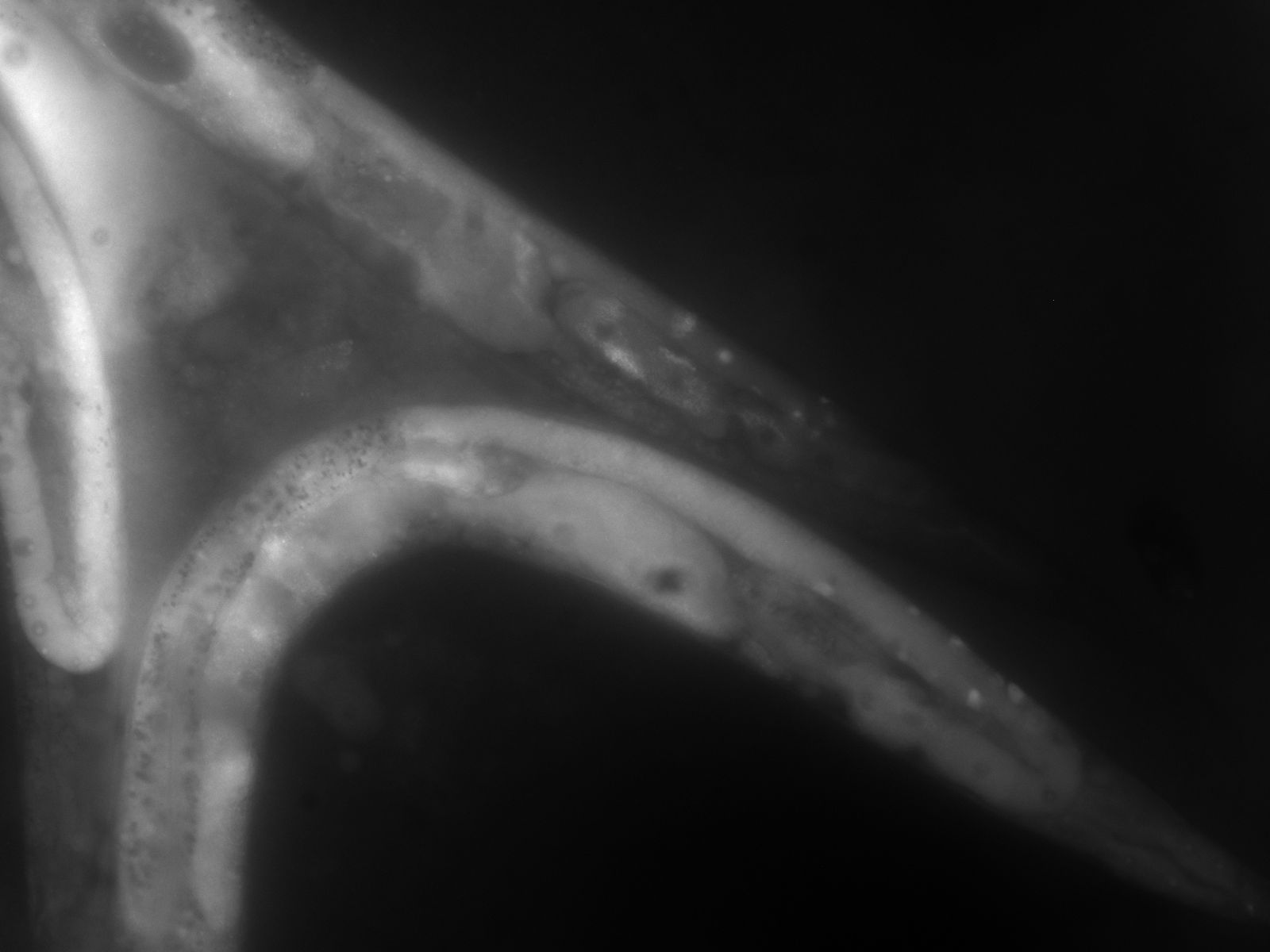

Supplement: S6 File — (ZIP) [file pgen.1011061.s006.zip › Fig.S4B+C - Original files/Fig.S4 RAW data and photos - JPEG/syto12 staining - FigS4bc - 1_rep - 14.5.23/unc-13+tfg-1302.jpg]

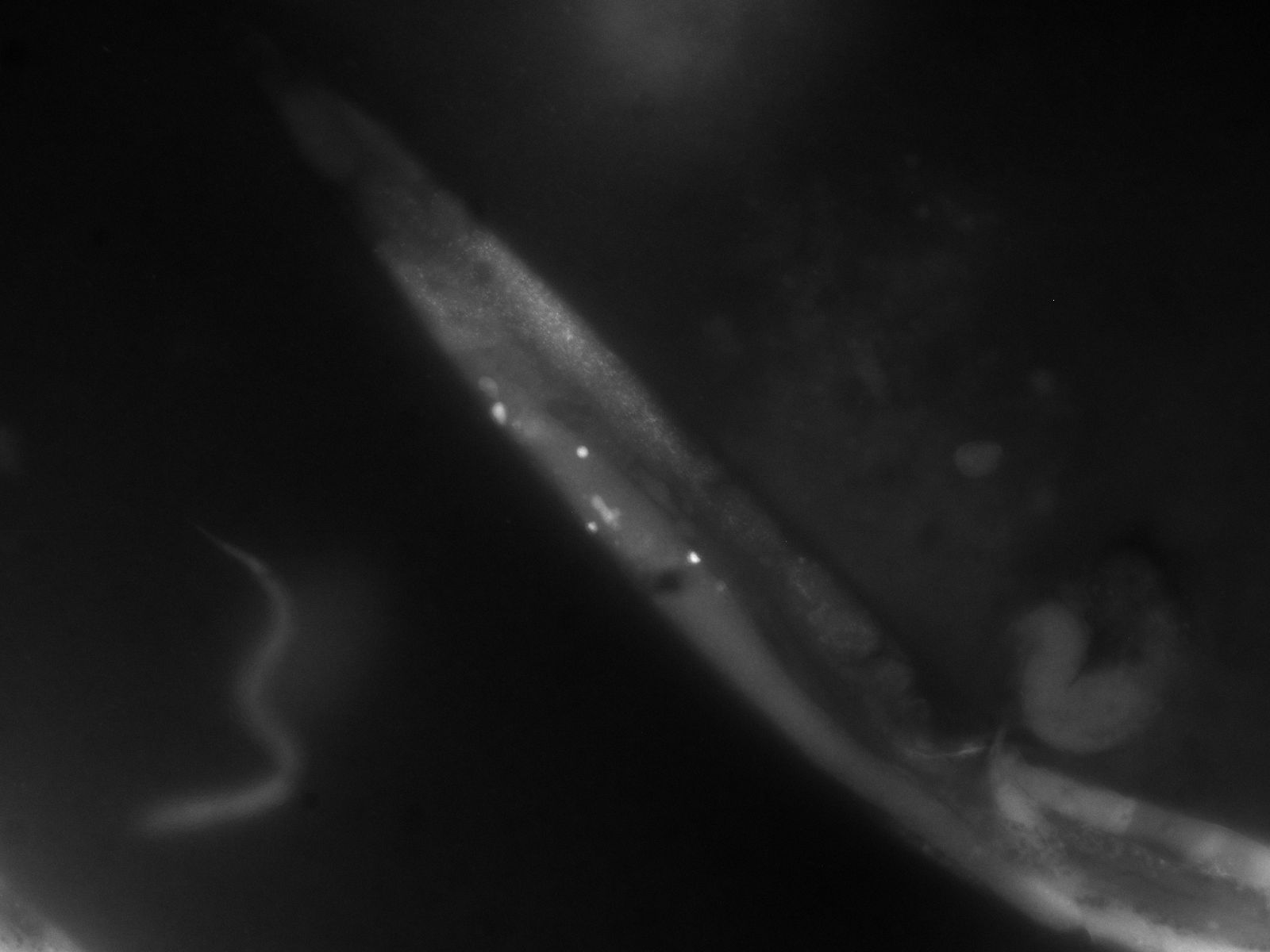

Supplement: S6 File — (ZIP) [file pgen.1011061.s006.zip › Fig.S4B+C - Original files/Fig.S4 RAW data and photos - JPEG/syto12 staining - FigS4bc - 1_rep - 14.5.23/unc-13+tfg-1303.jpg]

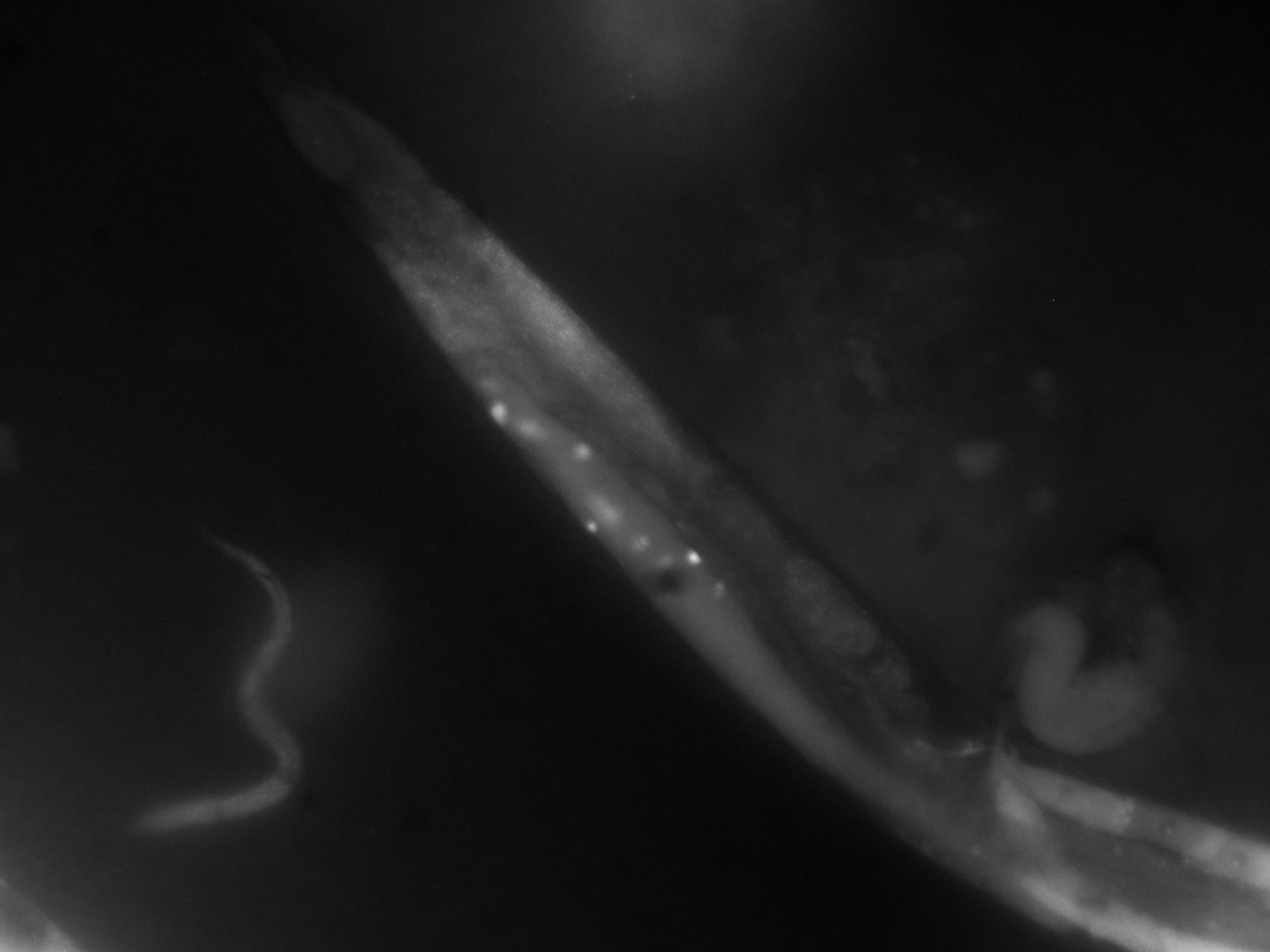

Supplement: S6 File — (ZIP) [file pgen.1011061.s006.zip › Fig.S4B+C - Original files/Fig.S4 RAW data and photos - JPEG/syto12 staining - FigS4bc - 1_rep - 14.5.23/unc-13+tfg-1304.jpg]

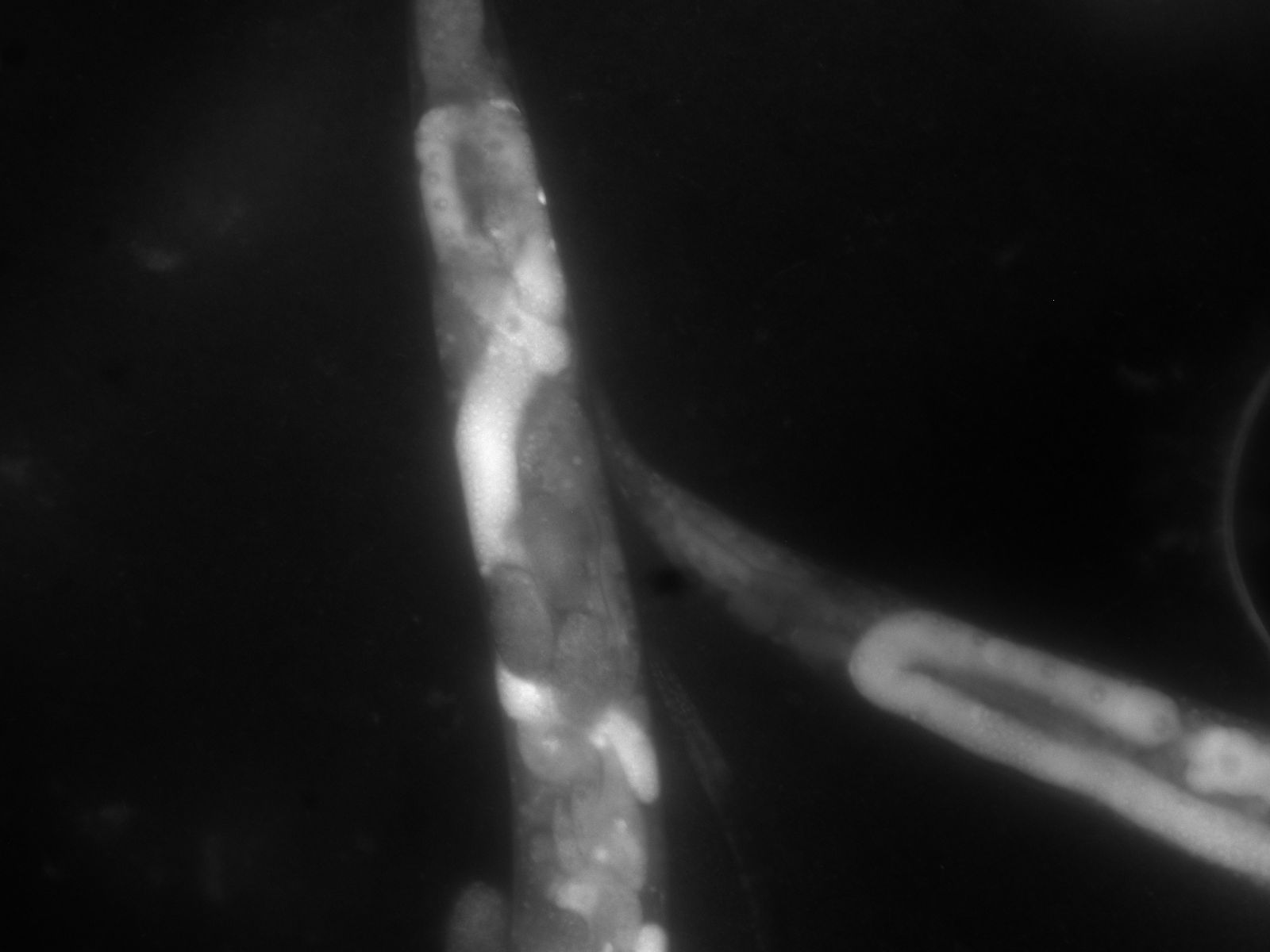

Supplement: S6 File — (ZIP) [file pgen.1011061.s006.zip › Fig.S4B+C - Original files/Fig.S4 RAW data and photos - JPEG/syto12 staining - FigS4bc - 1_rep - 14.5.23/unc-31_unc-64+pad12305.jpg]

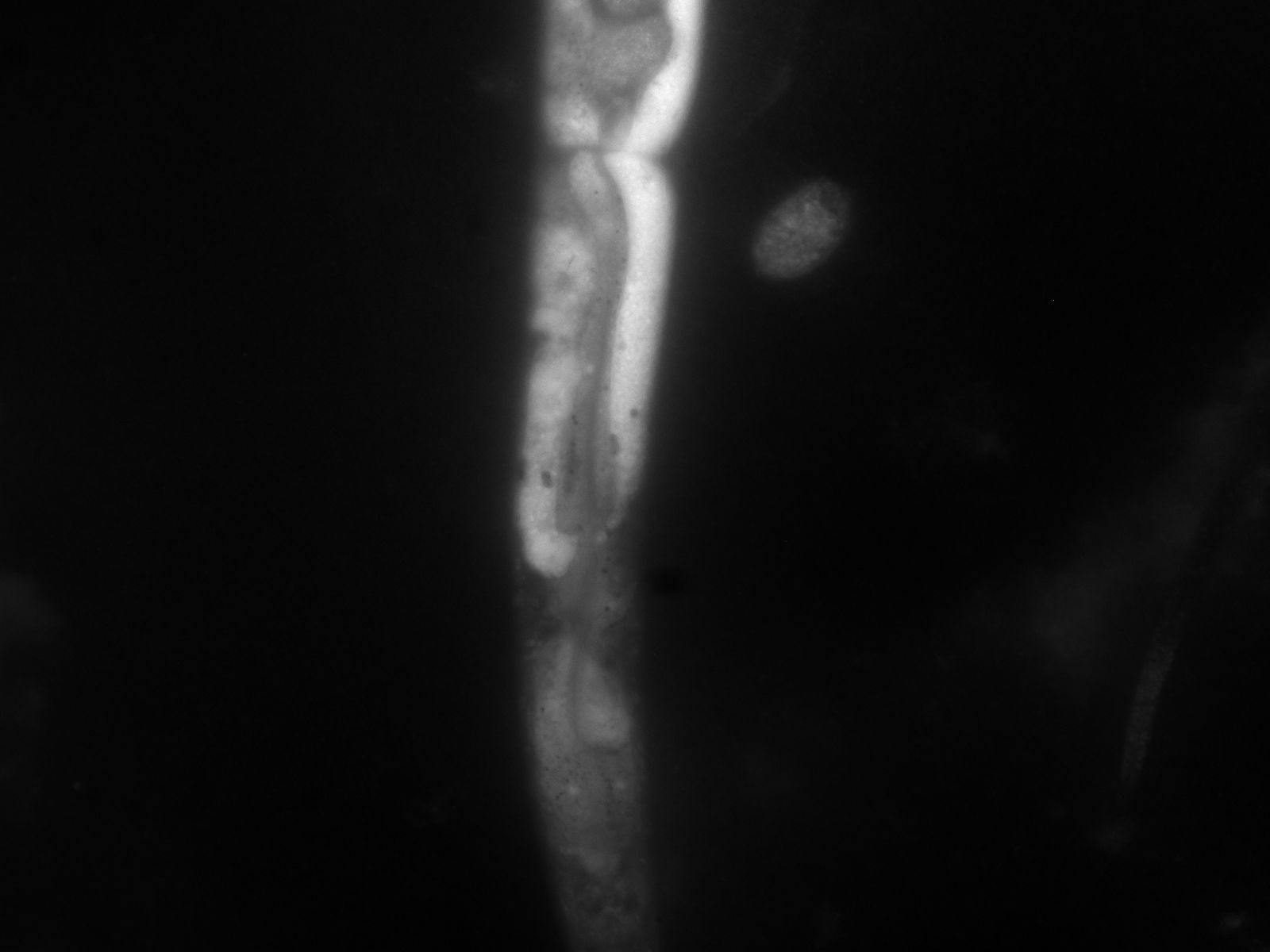

Supplement: S6 File — (ZIP) [file pgen.1011061.s006.zip › Fig.S4B+C - Original files/Fig.S4 RAW data and photos - JPEG/syto12 staining - FigS4bc - 1_rep - 14.5.23/unc-31_unc-64+pad12306.jpg]

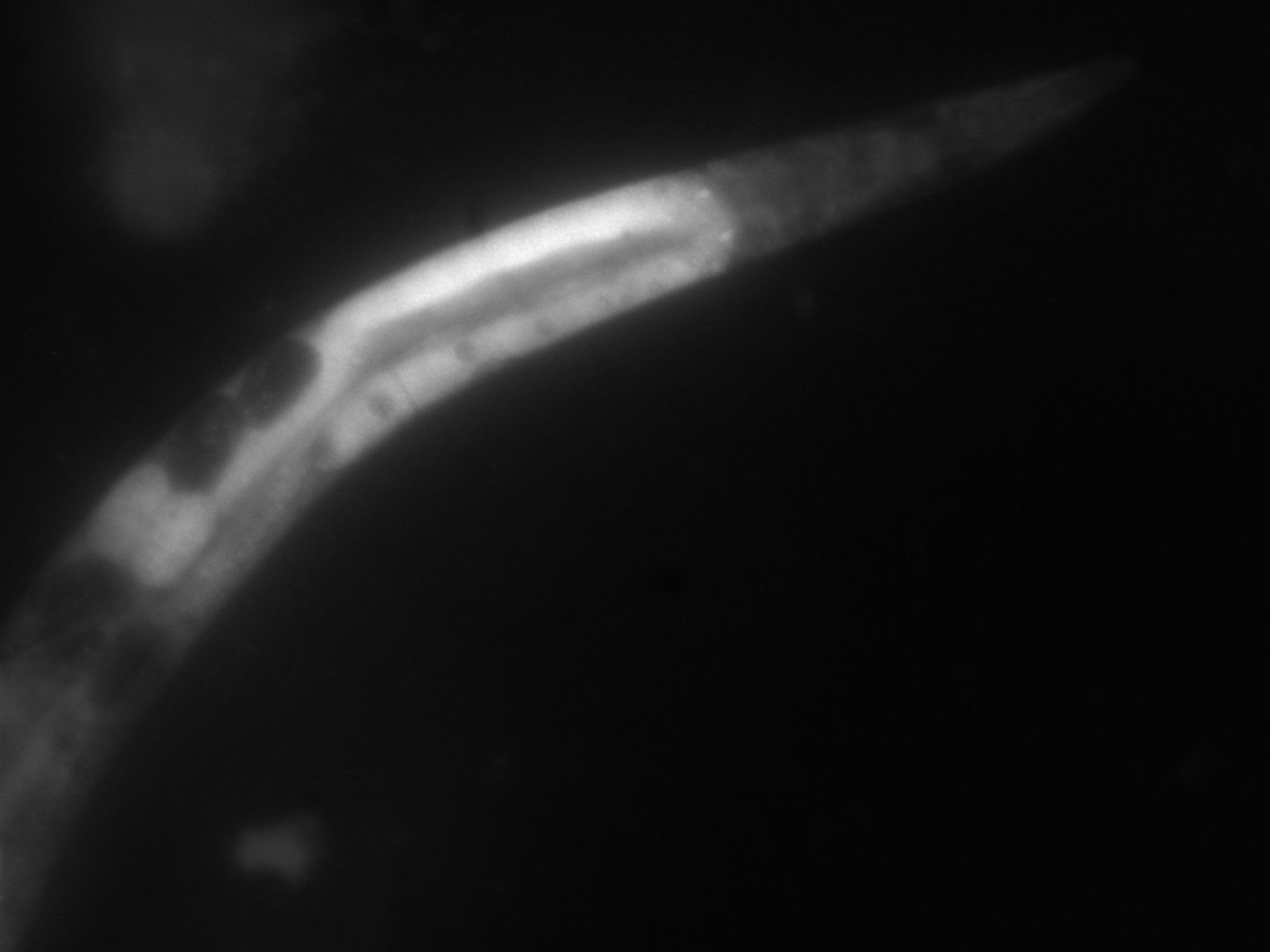

Supplement: S6 File — (ZIP) [file pgen.1011061.s006.zip › Fig.S4B+C - Original files/Fig.S4 RAW data and photos - JPEG/syto12 staining - FigS4bc - 1_rep - 14.5.23/unc-31_unc-64+pad12307.jpg]

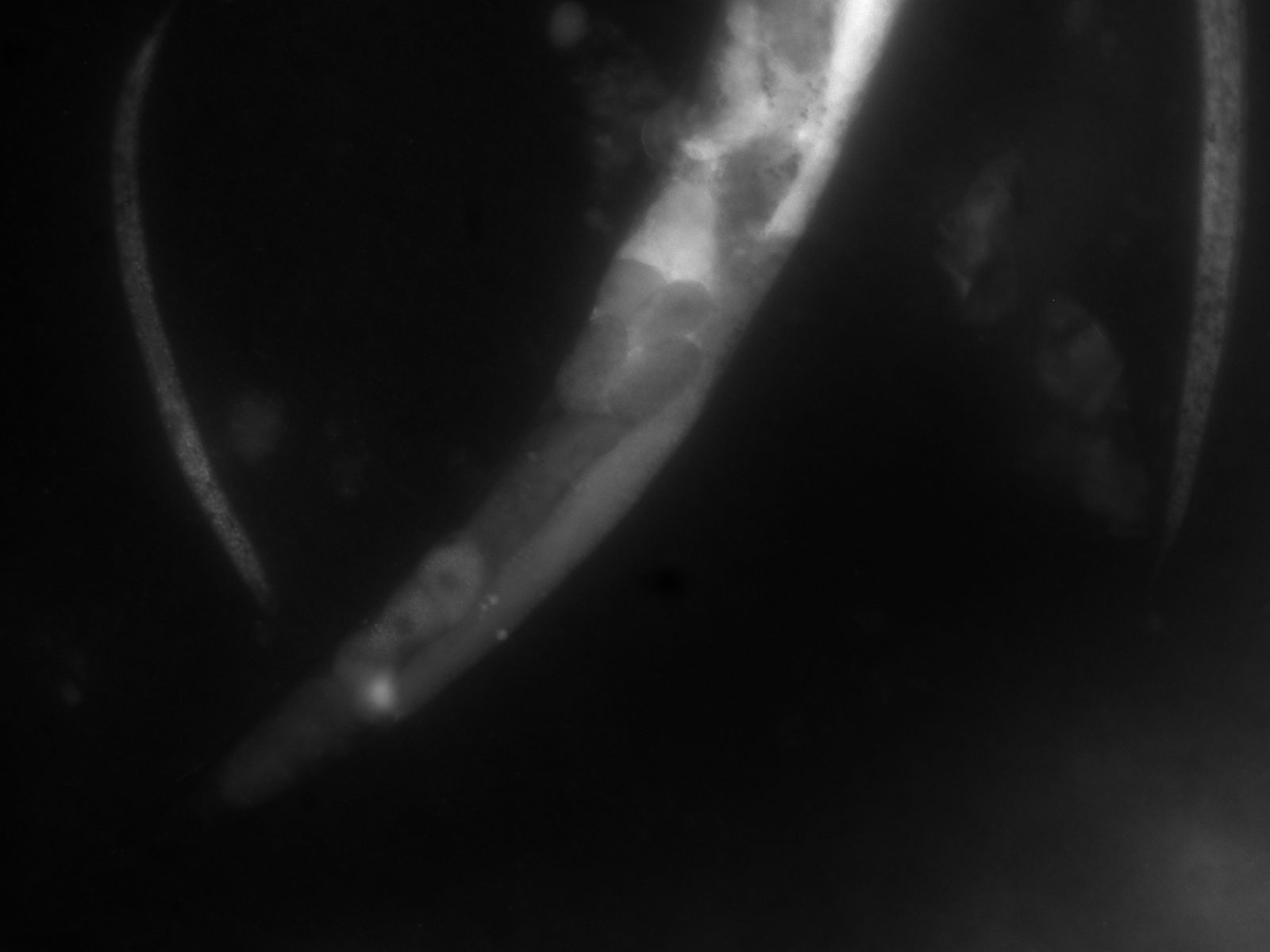

Supplement: S6 File — (ZIP) [file pgen.1011061.s006.zip › Fig.S4B+C - Original files/Fig.S4 RAW data and photos - JPEG/syto12 staining - FigS4bc - 1_rep - 14.5.23/unc-31_unc-64+pad12308.jpg]

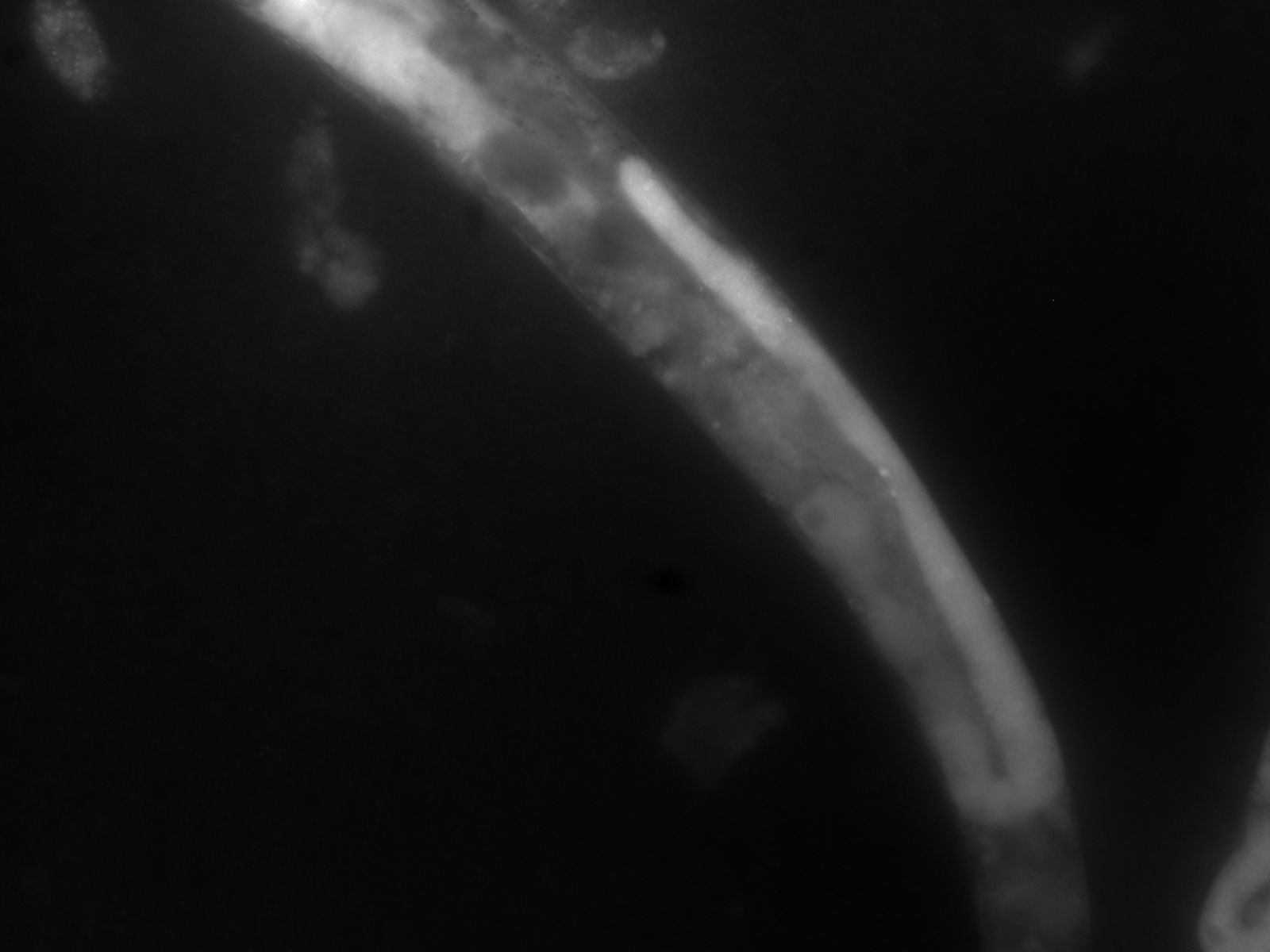

Supplement: S6 File — (ZIP) [file pgen.1011061.s006.zip › Fig.S4B+C - Original files/Fig.S4 RAW data and photos - JPEG/syto12 staining - FigS4bc - 1_rep - 14.5.23/unc-31_unc-64+pad12309.jpg]

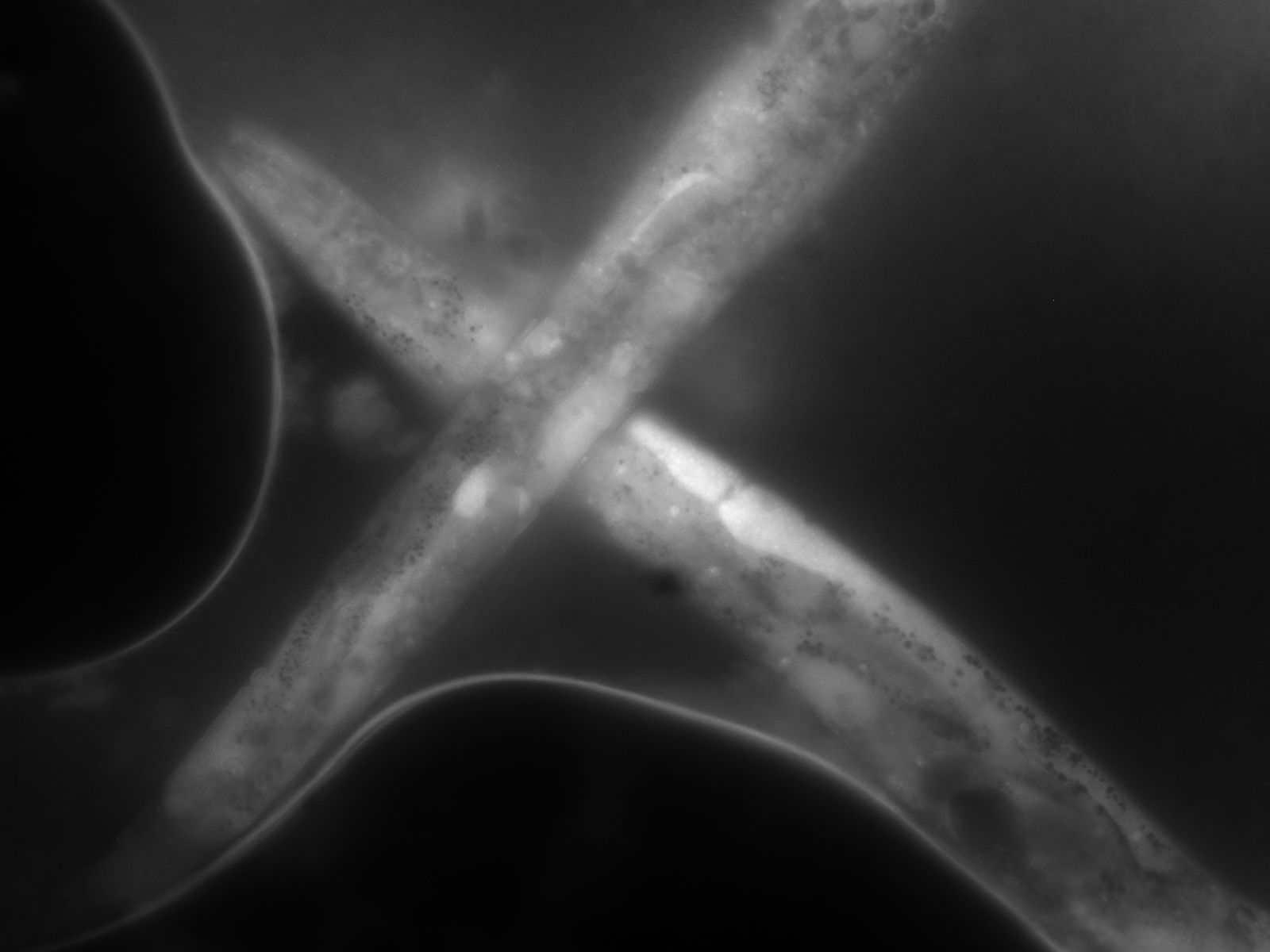

Supplement: S6 File — (ZIP) [file pgen.1011061.s006.zip › Fig.S4B+C - Original files/Fig.S4 RAW data and photos - JPEG/syto12 staining - FigS4bc - 1_rep - 14.5.23/unc-31_unc-64+pad12310.jpg]

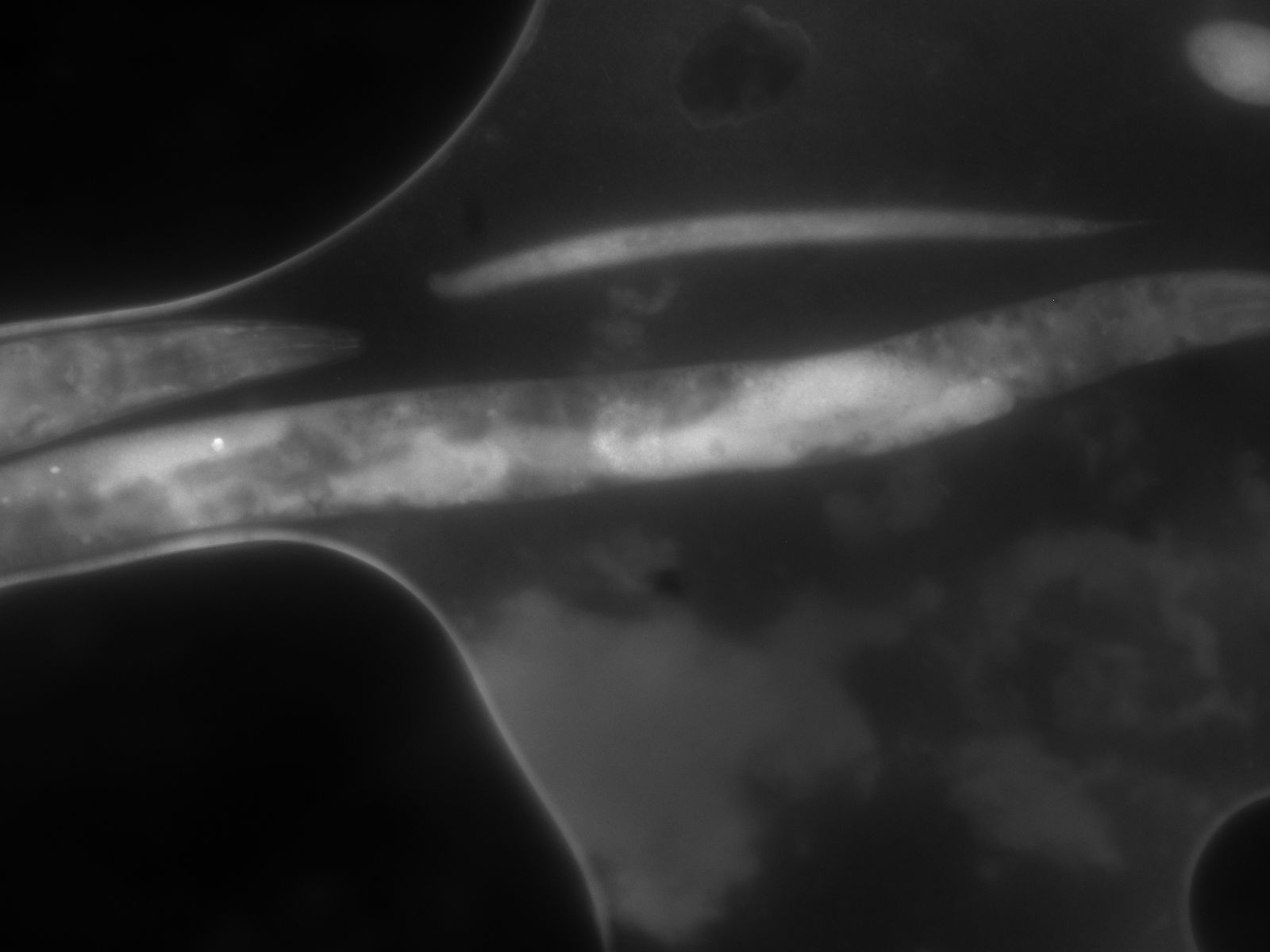

Supplement: S6 File — (ZIP) [file pgen.1011061.s006.zip › Fig.S4B+C - Original files/Fig.S4 RAW data and photos - JPEG/syto12 staining - FigS4bc - 1_rep - 14.5.23/unc-31_unc-64+pad12311.jpg]

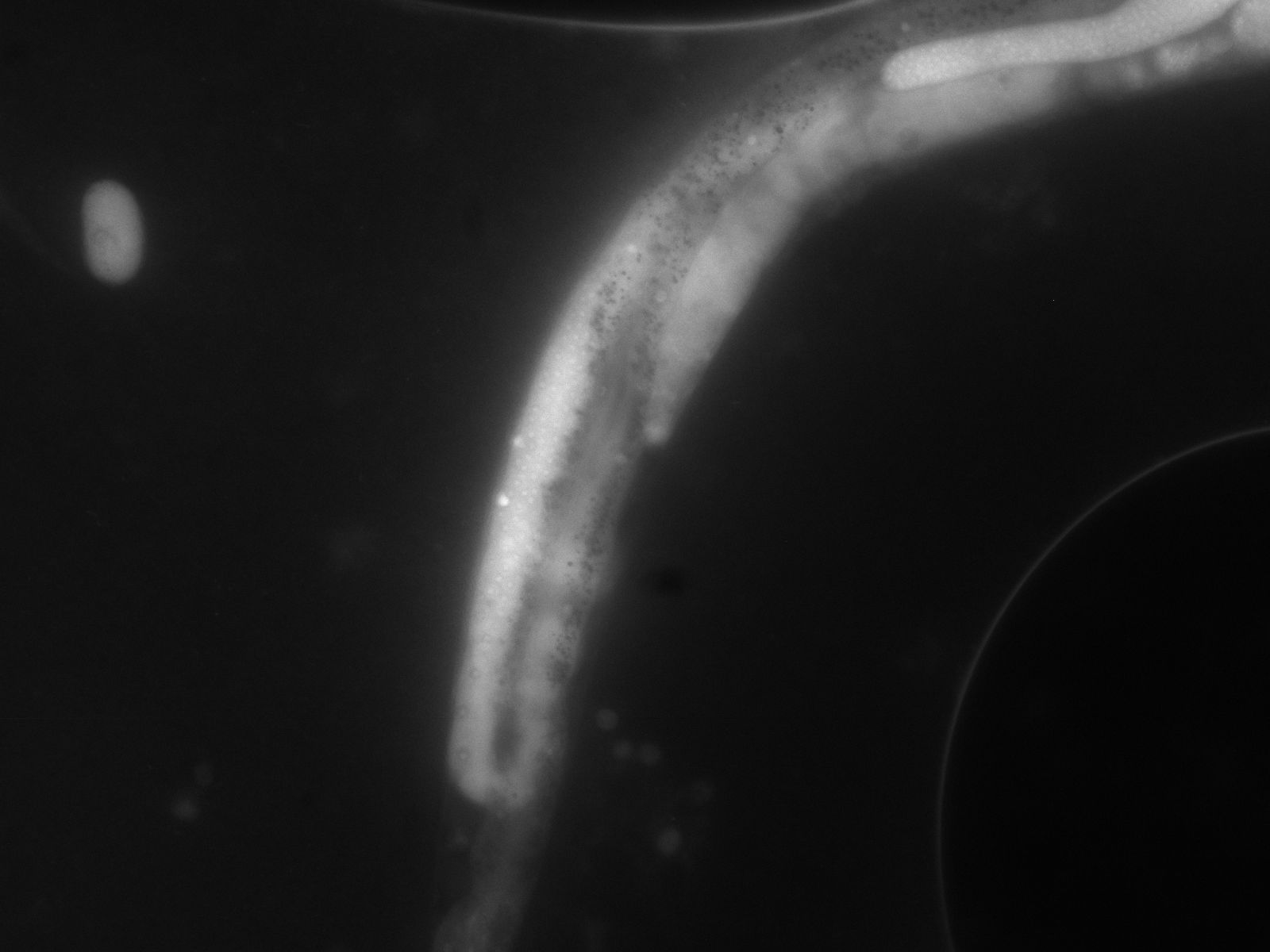

Supplement: S6 File — (ZIP) [file pgen.1011061.s006.zip › Fig.S4B+C - Original files/Fig.S4 RAW data and photos - JPEG/syto12 staining - FigS4bc - 1_rep - 14.5.23/unc-31_unc-64+pad12312.jpg]

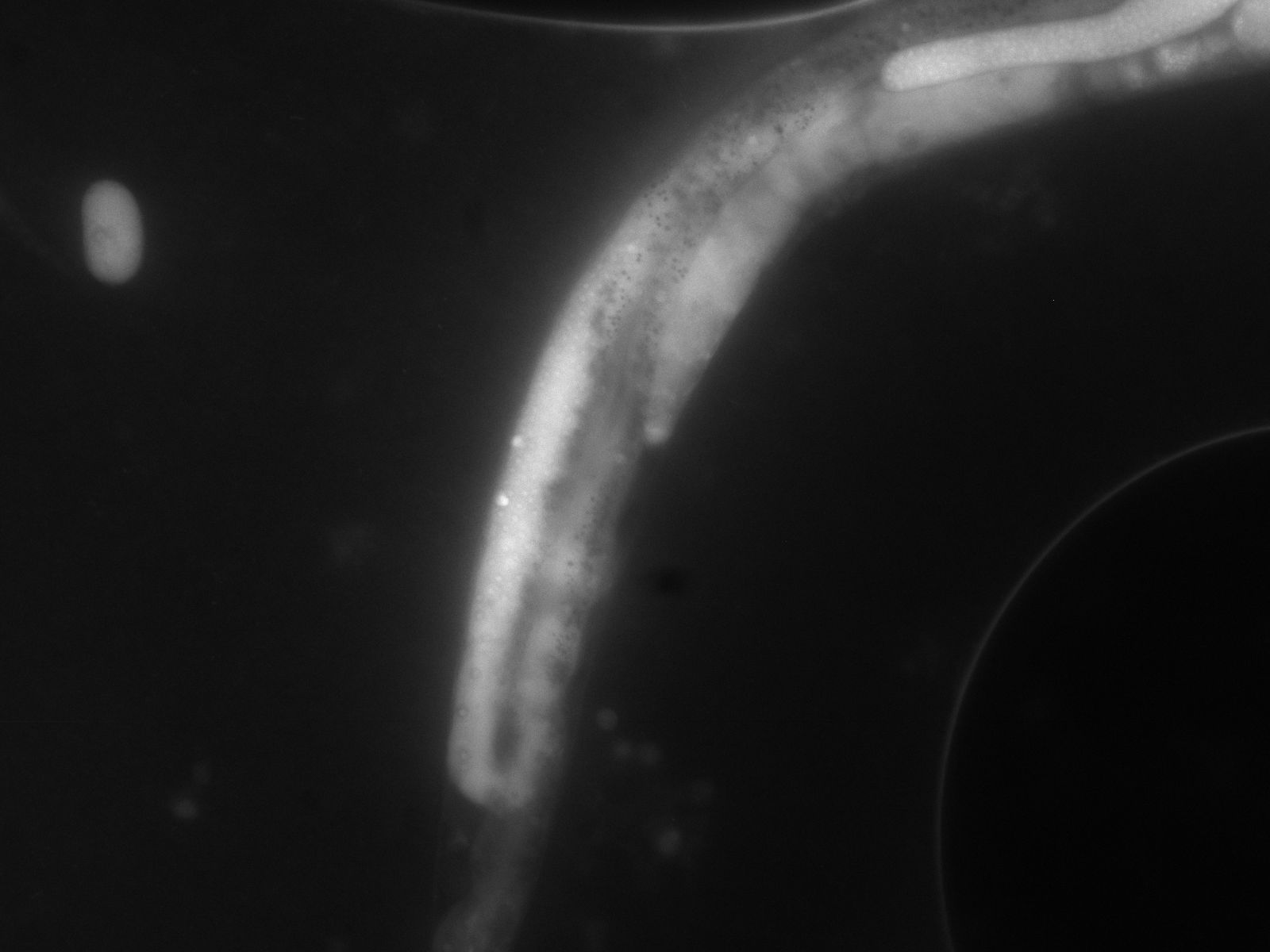

Supplement: S6 File — (ZIP) [file pgen.1011061.s006.zip › Fig.S4B+C - Original files/Fig.S4 RAW data and photos - JPEG/syto12 staining - FigS4bc - 1_rep - 14.5.23/unc-31_unc-64+pad12313.jpg]

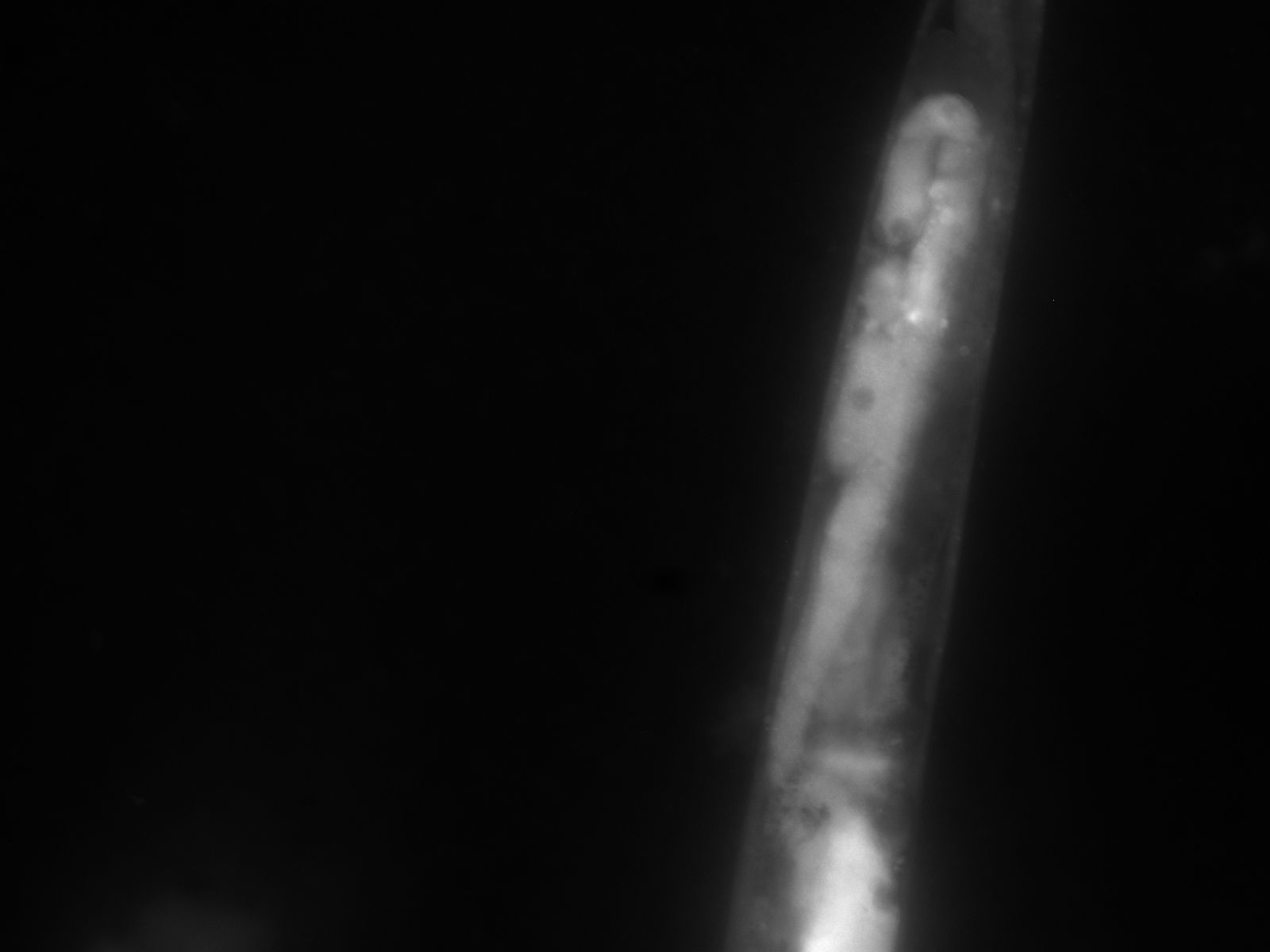

Supplement: S6 File — (ZIP) [file pgen.1011061.s006.zip › Fig.S4B+C - Original files/Fig.S4 RAW data and photos - JPEG/syto12 staining - FigS4bc - 1_rep - 14.5.23/unc-31_unc-64+pad12314.jpg]

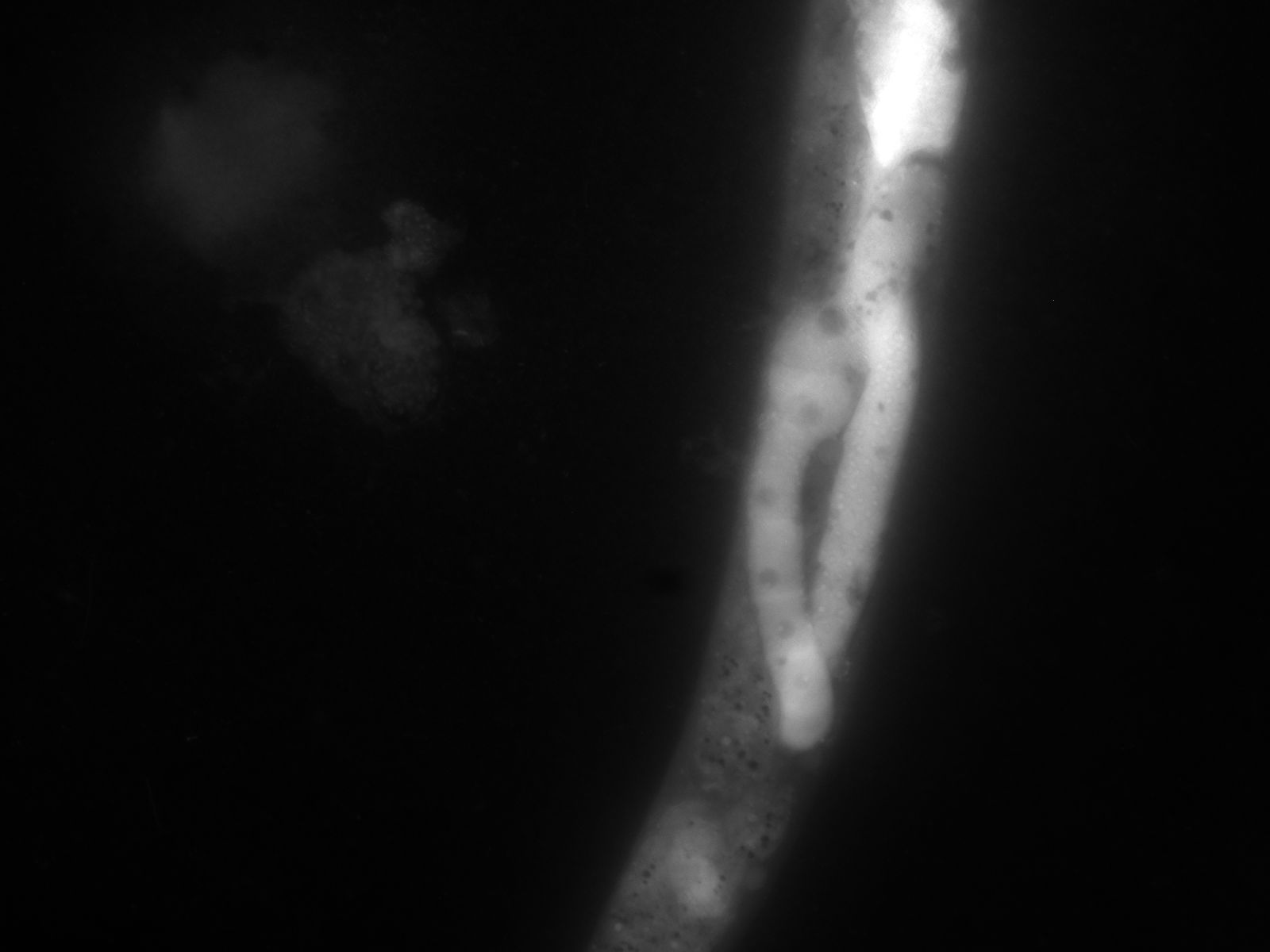

Supplement: S6 File — (ZIP) [file pgen.1011061.s006.zip › Fig.S4B+C - Original files/Fig.S4 RAW data and photos - JPEG/syto12 staining - FigS4bc - 1_rep - 14.5.23/unc-31_unc-64+pad12315.jpg]

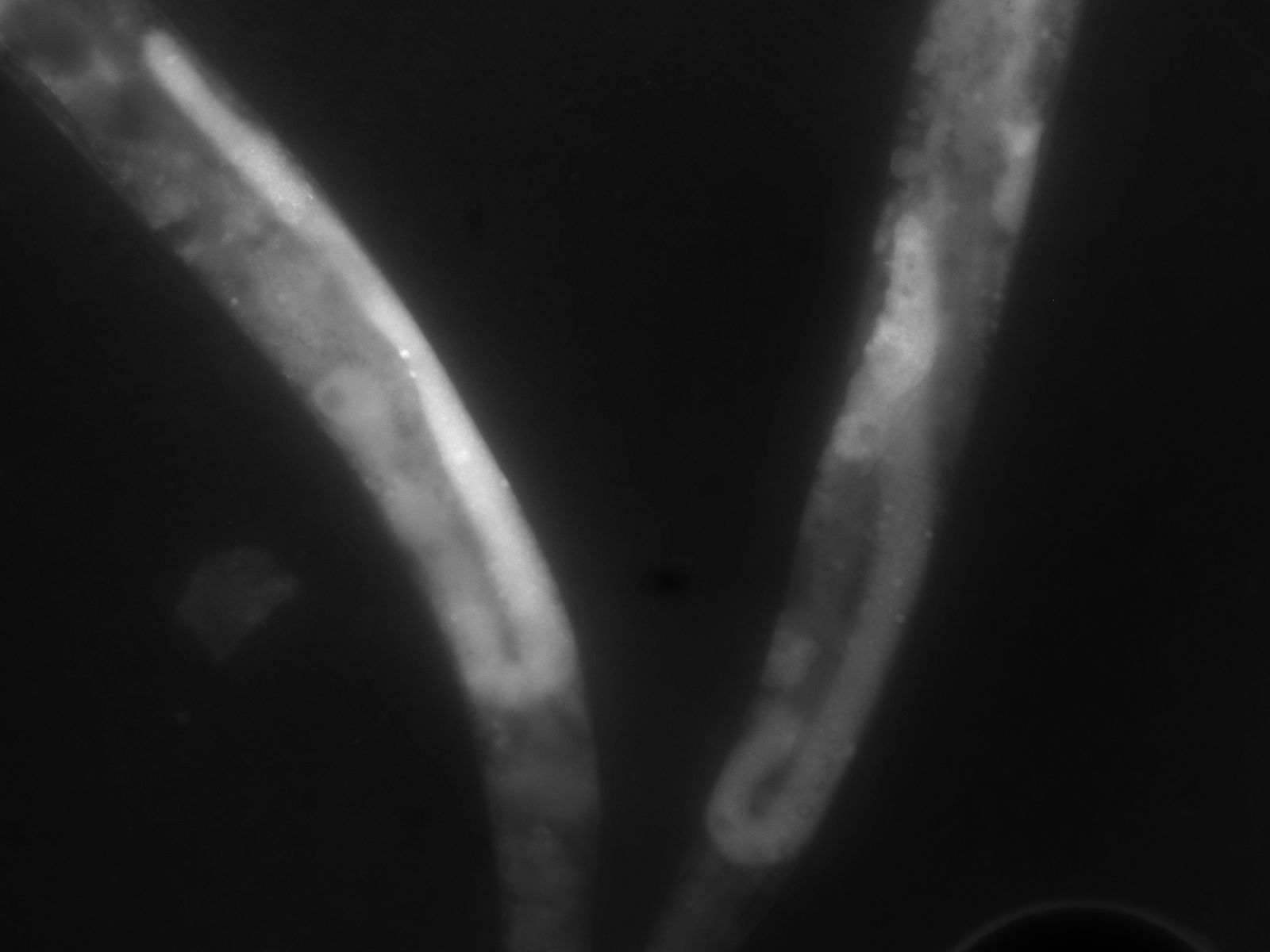

Supplement: S6 File — (ZIP) [file pgen.1011061.s006.zip › Fig.S4B+C - Original files/Fig.S4 RAW data and photos - JPEG/syto12 staining - FigS4bc - 1_rep - 14.5.23/unc-31_unc-64+pad12316.jpg]

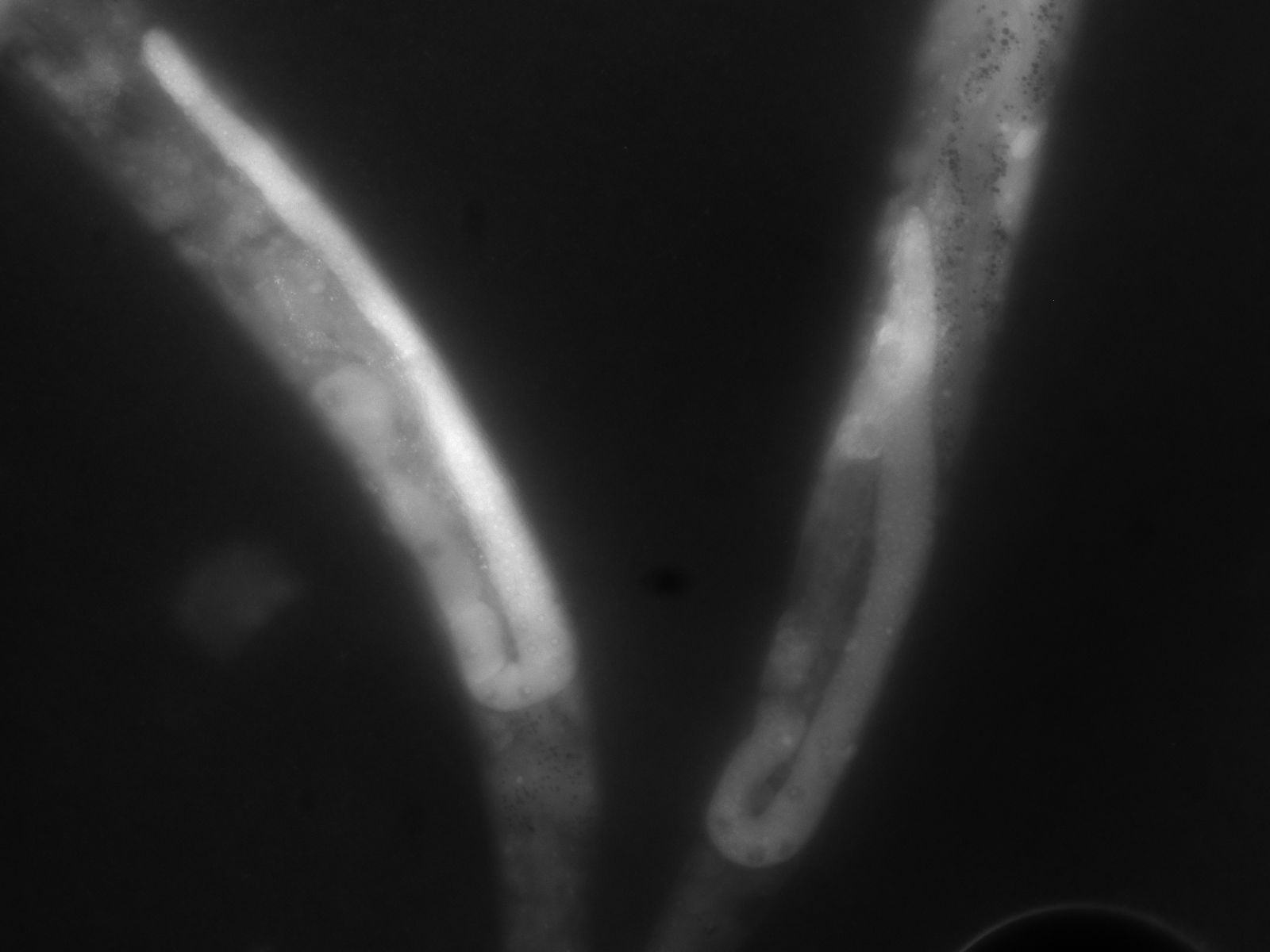

Supplement: S6 File — (ZIP) [file pgen.1011061.s006.zip › Fig.S4B+C - Original files/Fig.S4 RAW data and photos - JPEG/syto12 staining - FigS4bc - 1_rep - 14.5.23/unc-31_unc-64+pad12317.jpg]

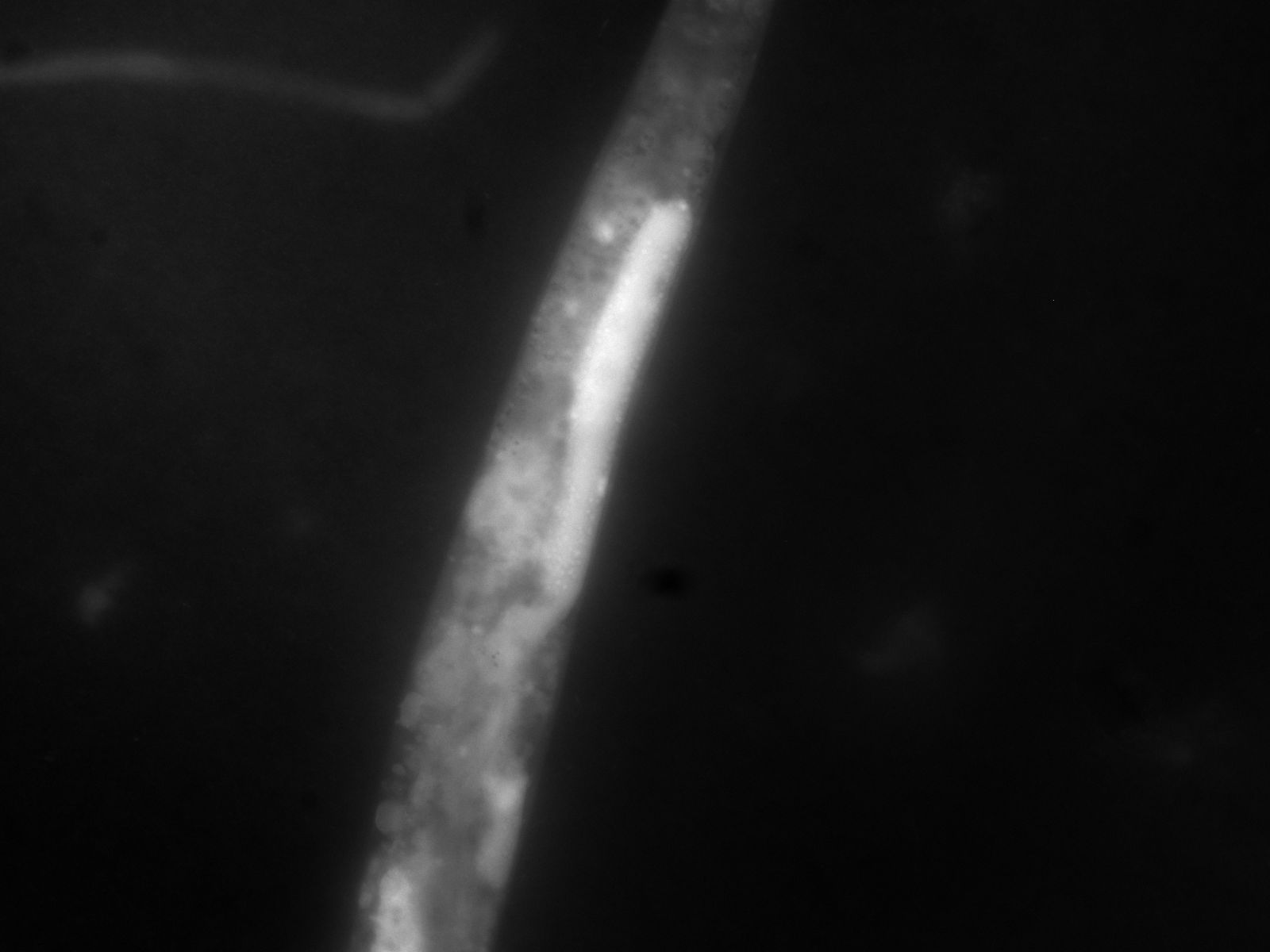

Supplement: S6 File — (ZIP) [file pgen.1011061.s006.zip › Fig.S4B+C - Original files/Fig.S4 RAW data and photos - JPEG/syto12 staining - FigS4bc - 1_rep - 14.5.23/unc-31_unc-64+pad12318.jpg]

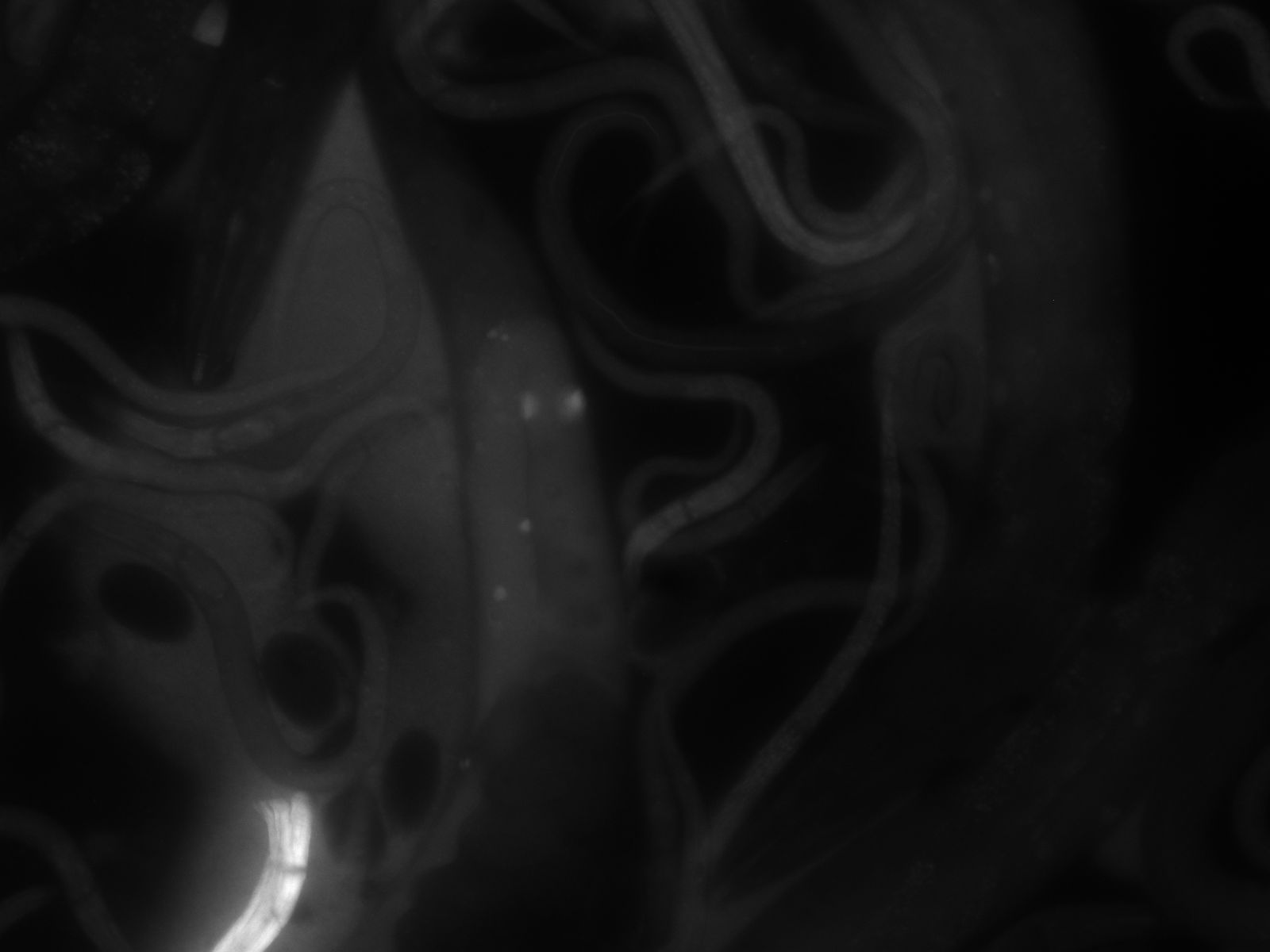

Supplement: S6 File — (ZIP) [file pgen.1011061.s006.zip › Fig.S4B+C - Original files/Fig.S4 RAW data and photos - JPEG/syto12 staining - FigS4bc - 1_rep - 14.5.23/unc-31_unc-64+tfg-1319.jpg]

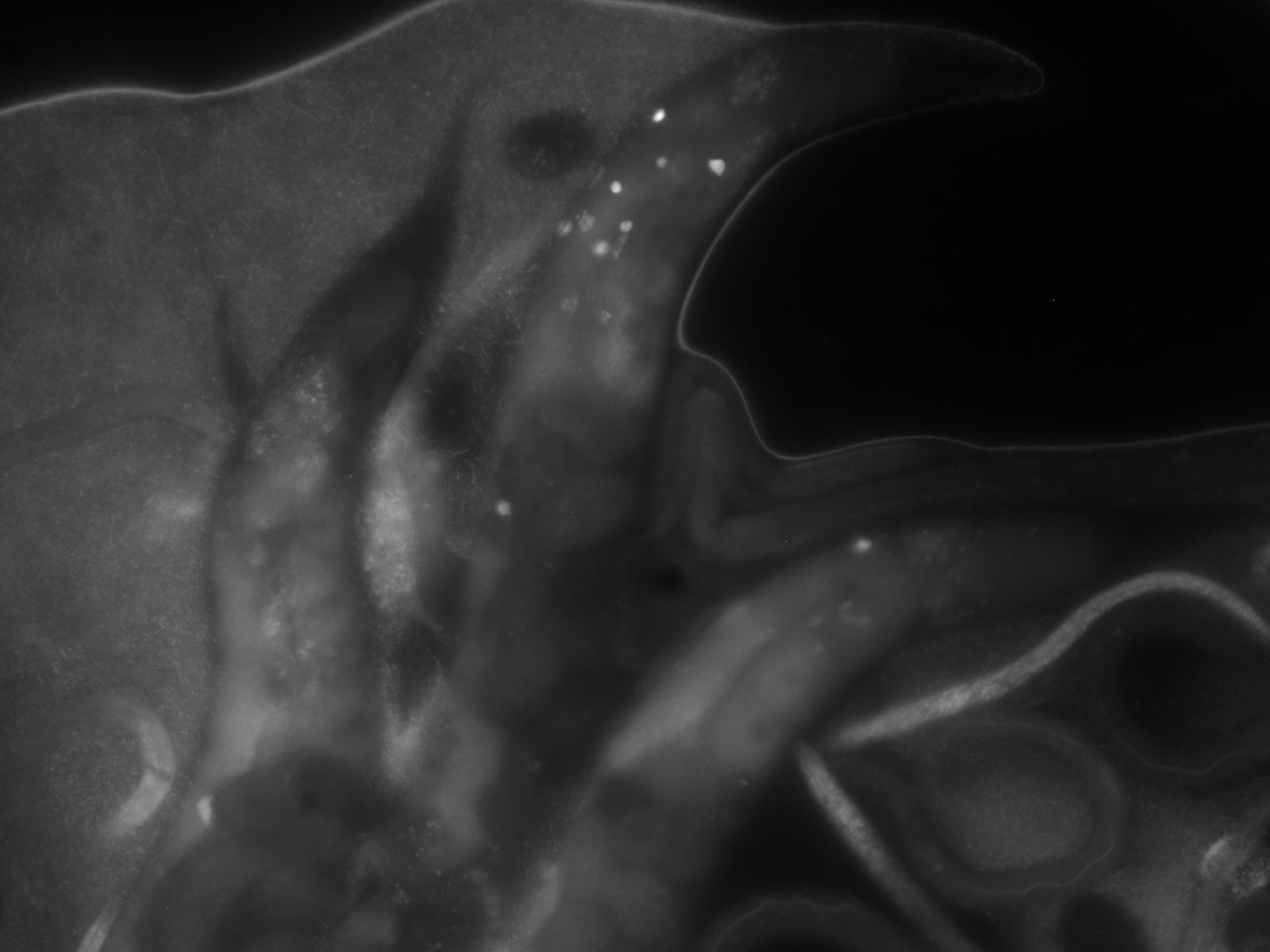

Supplement: S6 File — (ZIP) [file pgen.1011061.s006.zip › Fig.S4B+C - Original files/Fig.S4 RAW data and photos - JPEG/syto12 staining - FigS4bc - 1_rep - 14.5.23/unc-31_unc-64+tfg-1320.jpg]

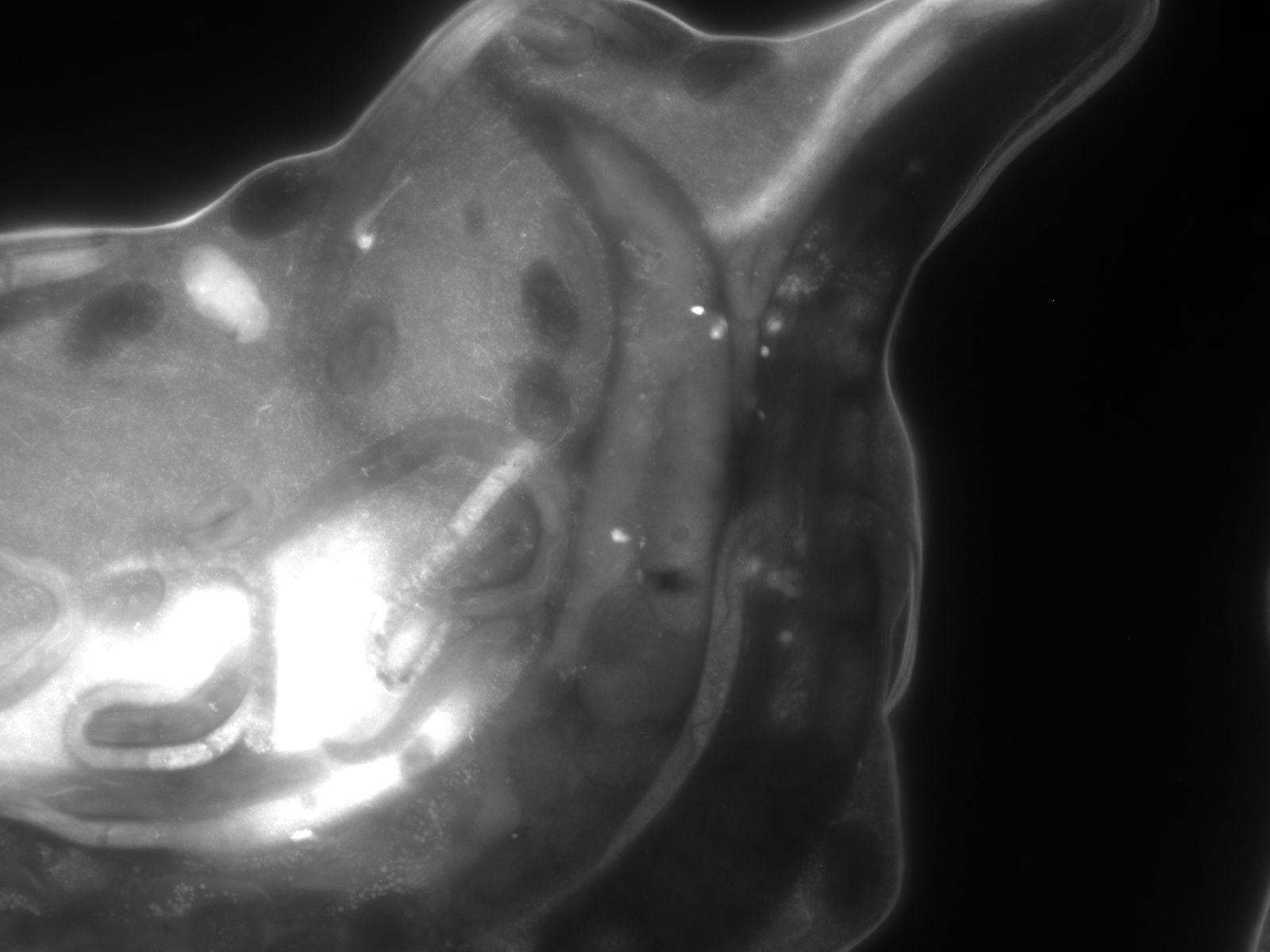

Supplement: S6 File — (ZIP) [file pgen.1011061.s006.zip › Fig.S4B+C - Original files/Fig.S4 RAW data and photos - JPEG/syto12 staining - FigS4bc - 1_rep - 14.5.23/unc-31_unc-64+tfg-1321.jpg]

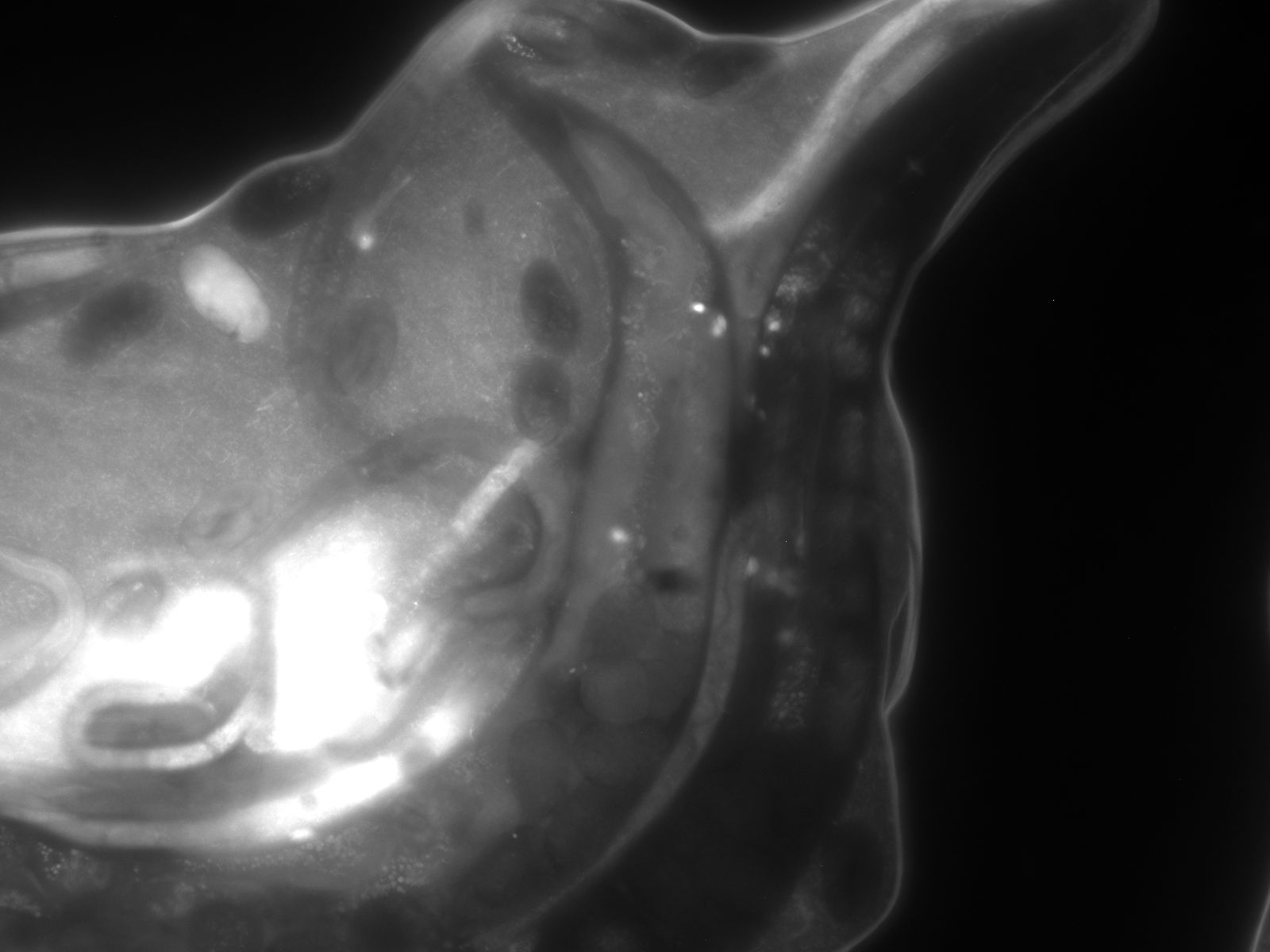

Supplement: S6 File — (ZIP) [file pgen.1011061.s006.zip › Fig.S4B+C - Original files/Fig.S4 RAW data and photos - JPEG/syto12 staining - FigS4bc - 1_rep - 14.5.23/unc-31_unc-64+tfg-1322.jpg]

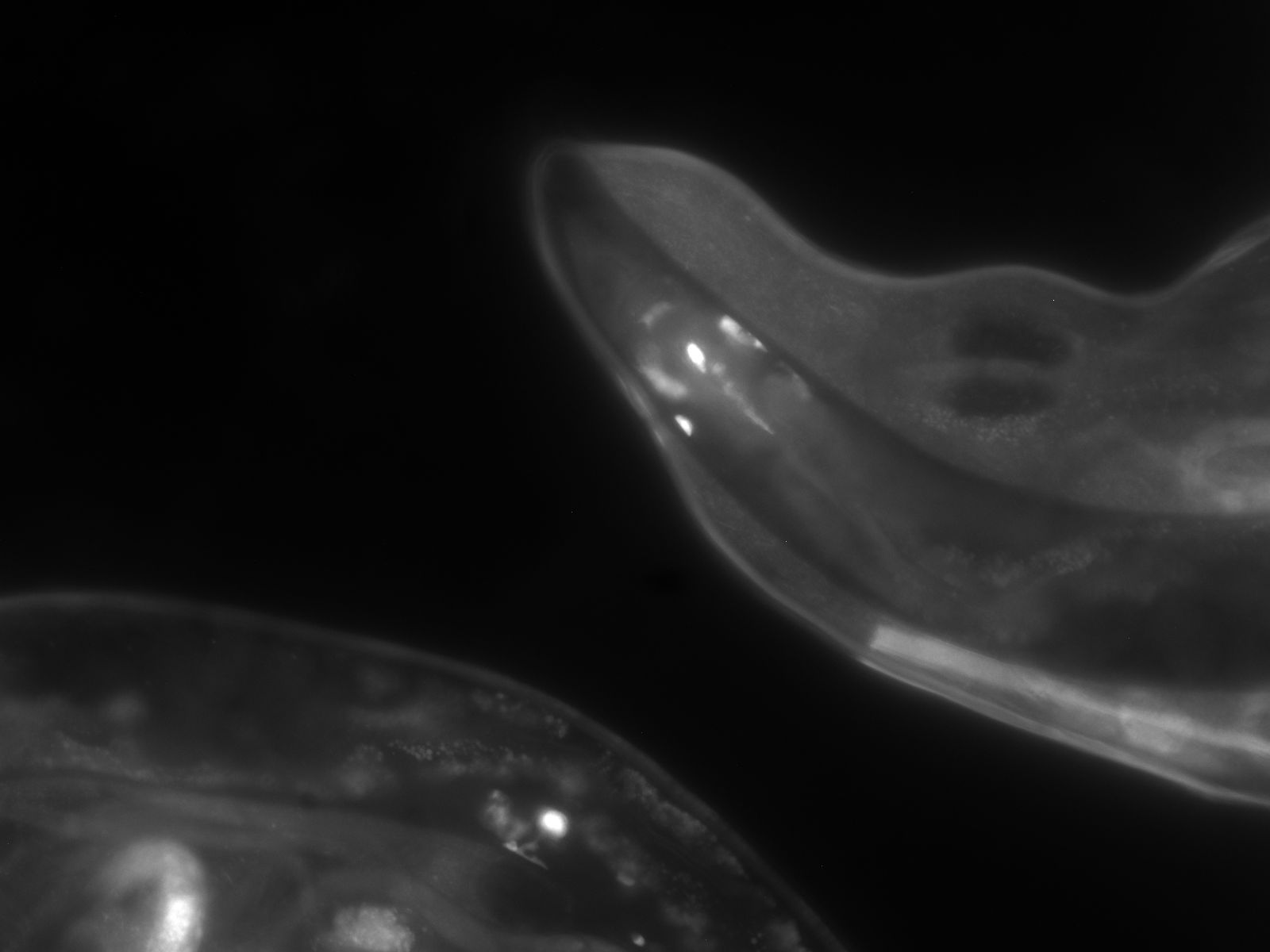

Supplement: S6 File — (ZIP) [file pgen.1011061.s006.zip › Fig.S4B+C - Original files/Fig.S4 RAW data and photos - JPEG/syto12 staining - FigS4bc - 1_rep - 14.5.23/unc-31_unc-64+tfg-1323.jpg]

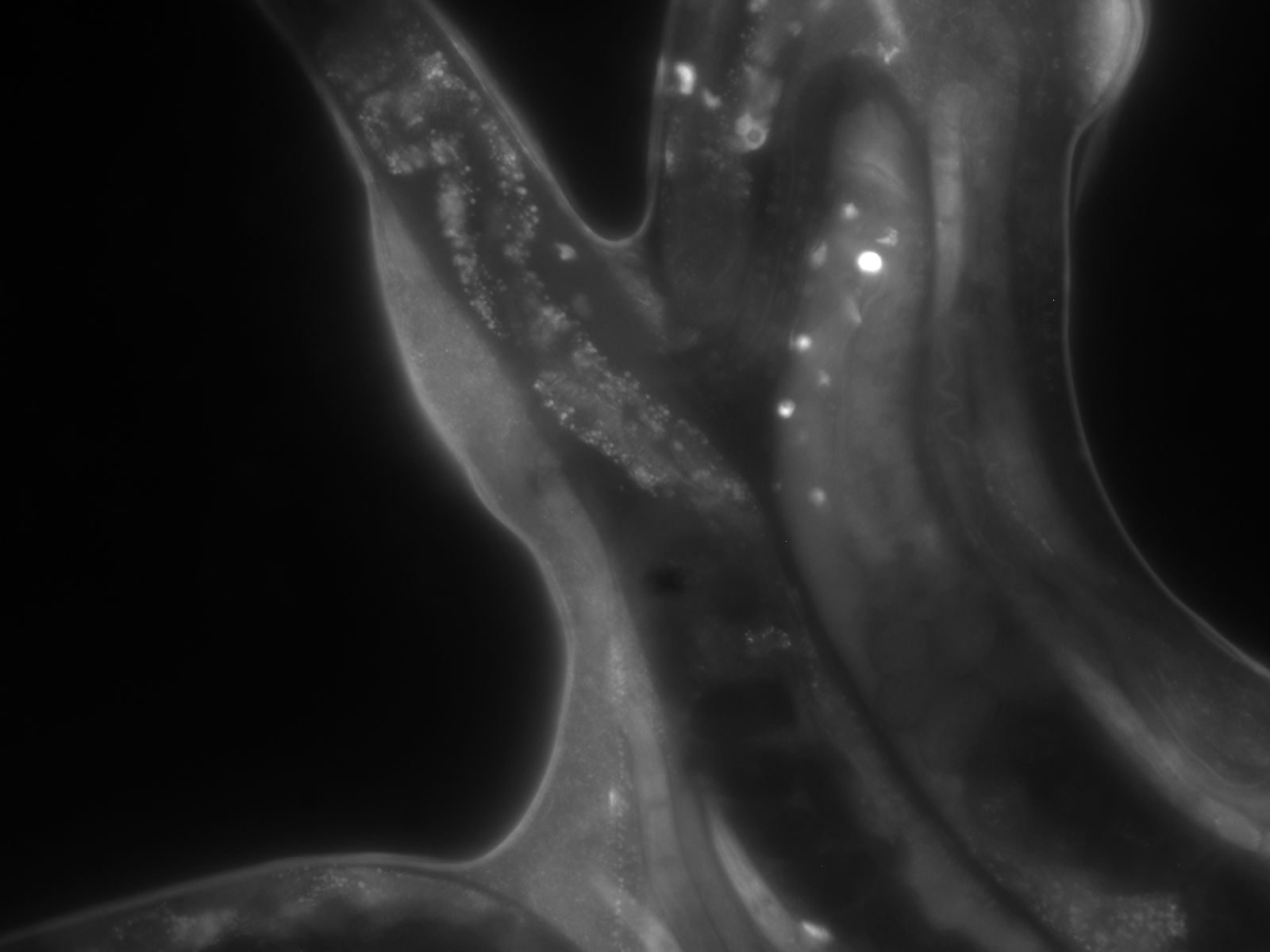

Supplement: S6 File — (ZIP) [file pgen.1011061.s006.zip › Fig.S4B+C - Original files/Fig.S4 RAW data and photos - JPEG/syto12 staining - FigS4bc - 1_rep - 14.5.23/unc-31_unc-64+tfg-1324.jpg]

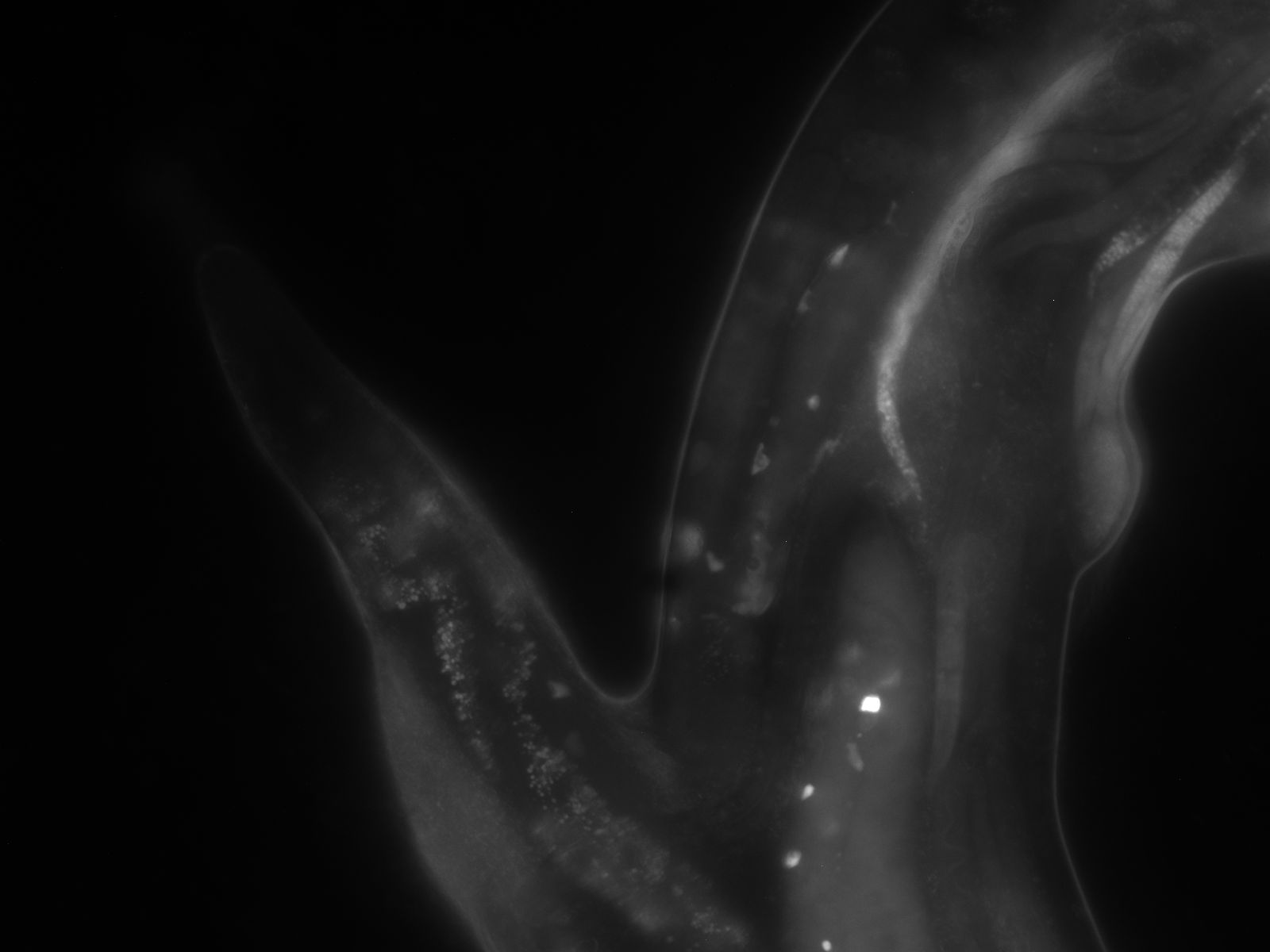

Supplement: S6 File — (ZIP) [file pgen.1011061.s006.zip › Fig.S4B+C - Original files/Fig.S4 RAW data and photos - JPEG/syto12 staining - FigS4bc - 1_rep - 14.5.23/unc-31_unc-64+tfg-1325.jpg]

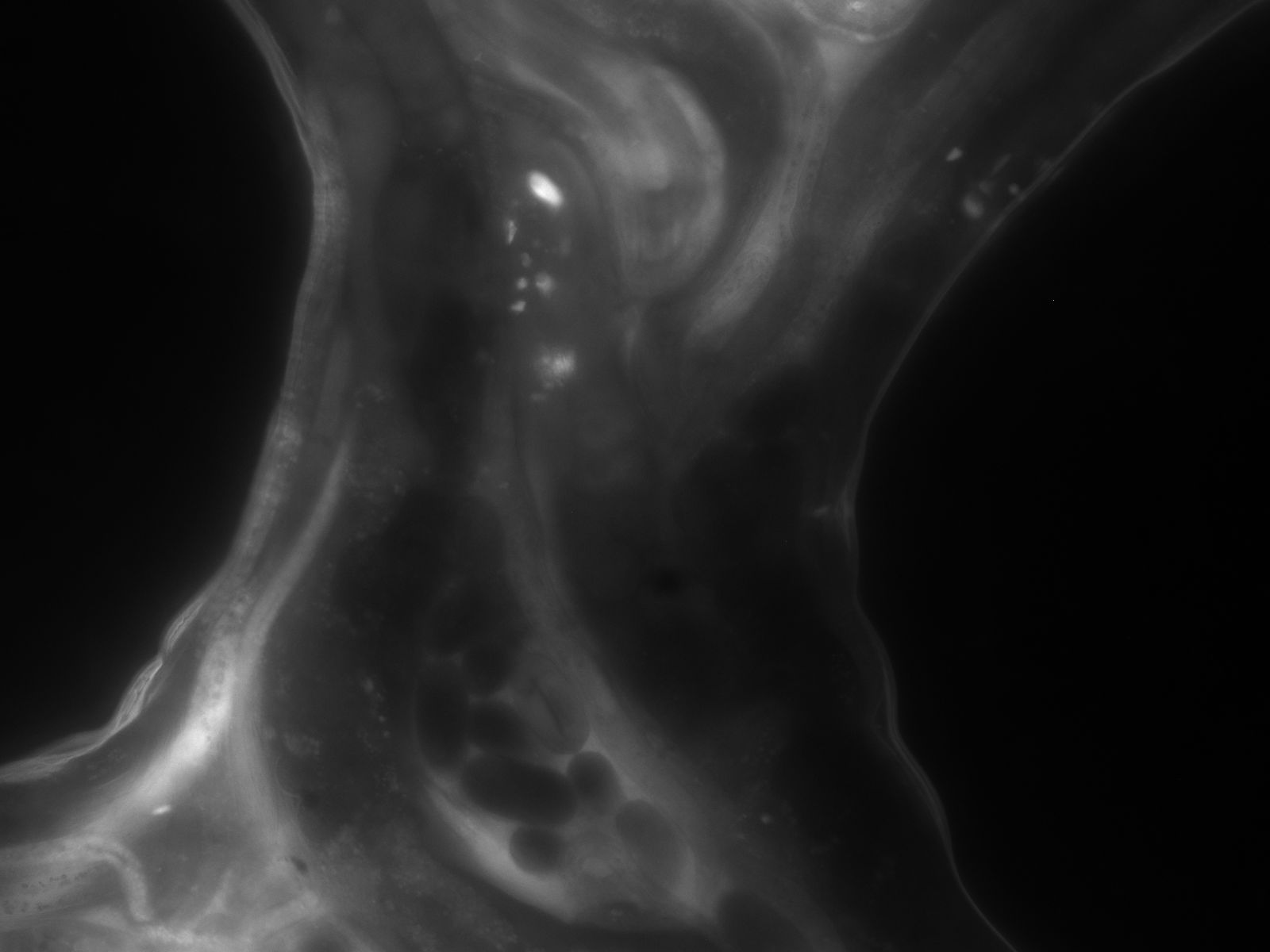

Supplement: S6 File — (ZIP) [file pgen.1011061.s006.zip › Fig.S4B+C - Original files/Fig.S4 RAW data and photos - JPEG/syto12 staining - FigS4bc - 1_rep - 14.5.23/unc-31_unc-64+tfg-1326.jpg]

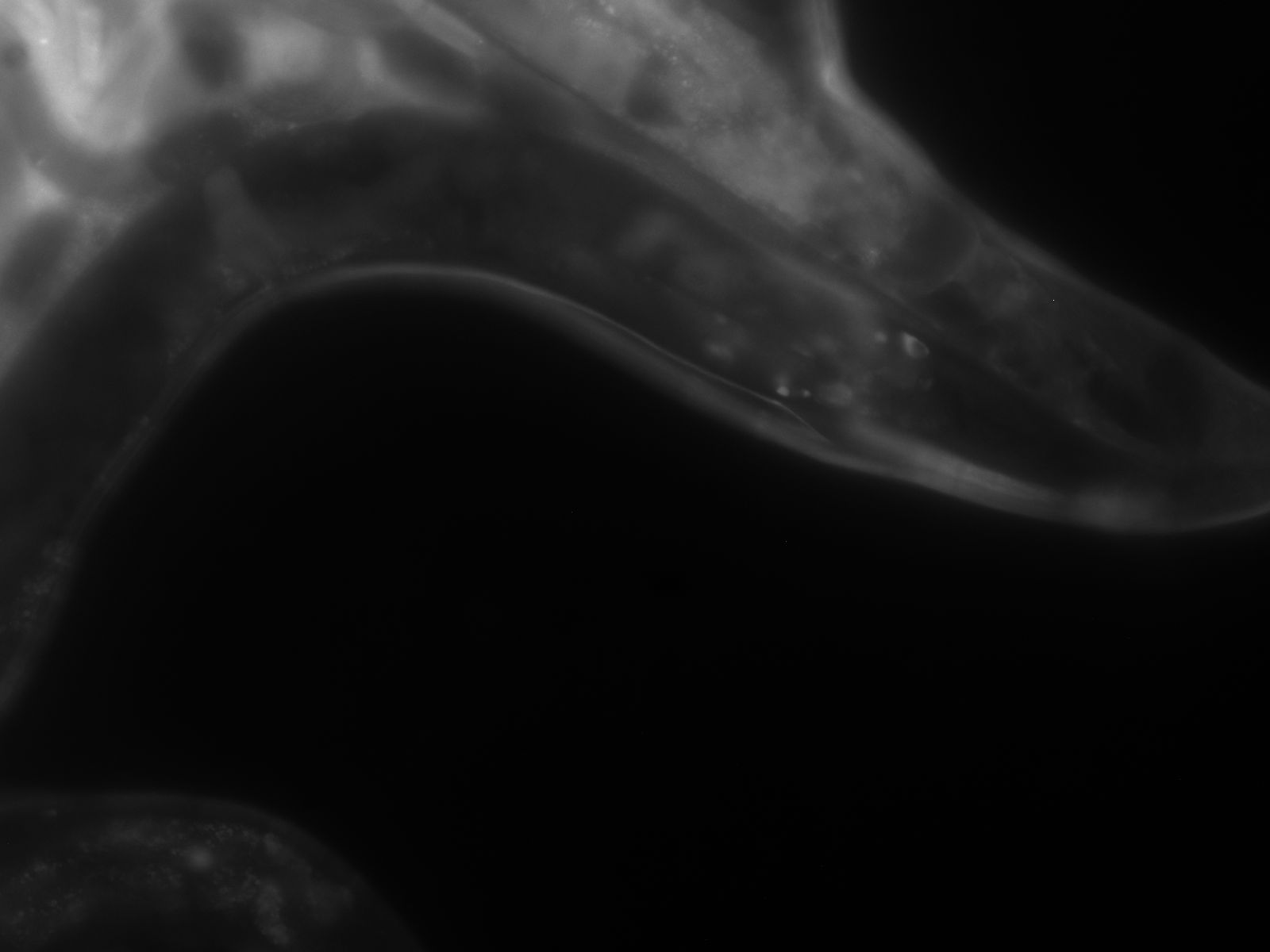

Supplement: S6 File — (ZIP) [file pgen.1011061.s006.zip › Fig.S4B+C - Original files/Fig.S4 RAW data and photos - JPEG/syto12 staining - FigS4bc - 1_rep - 14.5.23/unc-31_unc-64+tfg-1327.jpg]

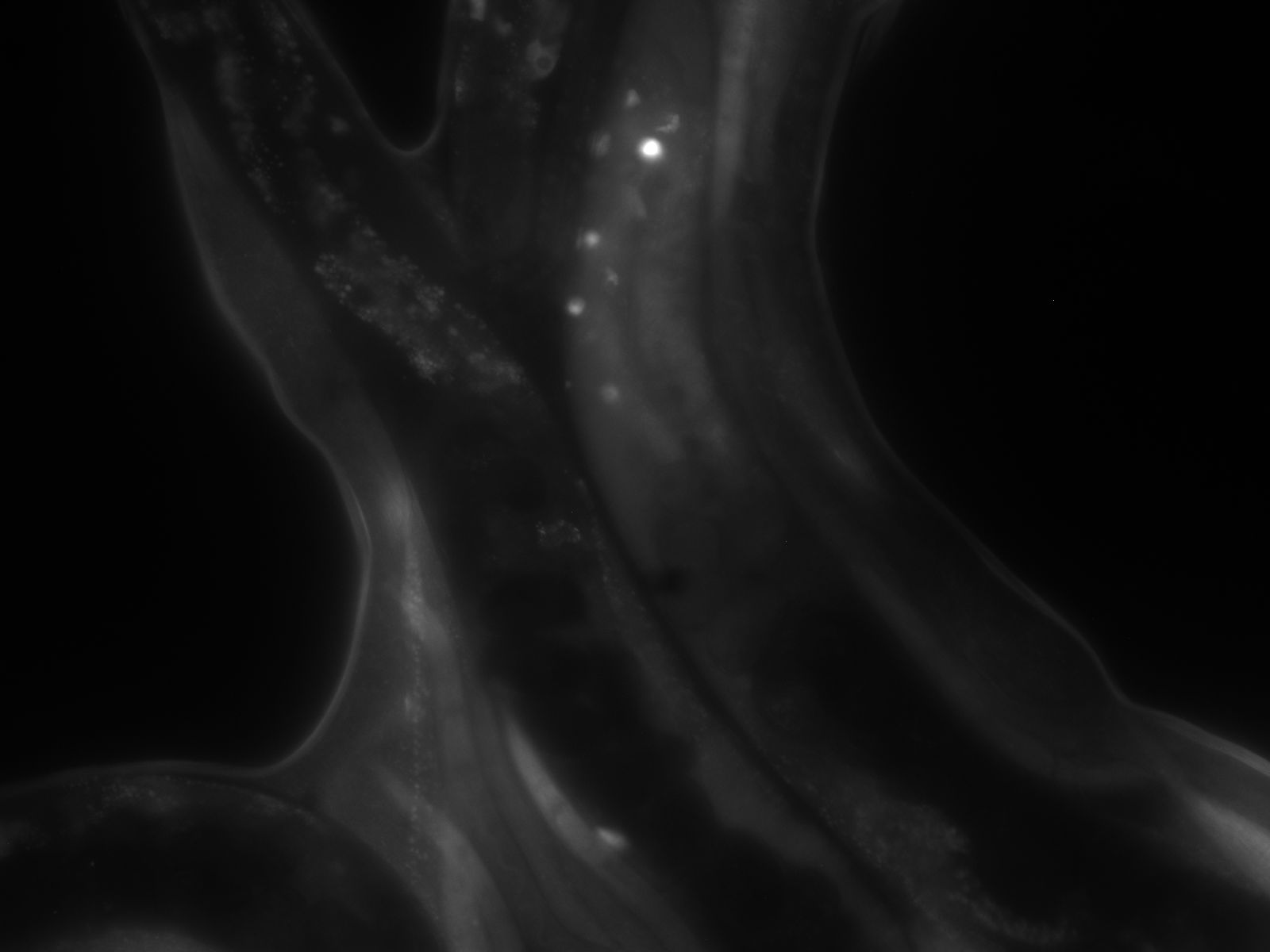

Supplement: S6 File — (ZIP) [file pgen.1011061.s006.zip › Fig.S4B+C - Original files/Fig.S4 RAW data and photos - JPEG/syto12 staining - FigS4bc - 1_rep - 14.5.23/unc-31_unc-64+tfg-1328.jpg]

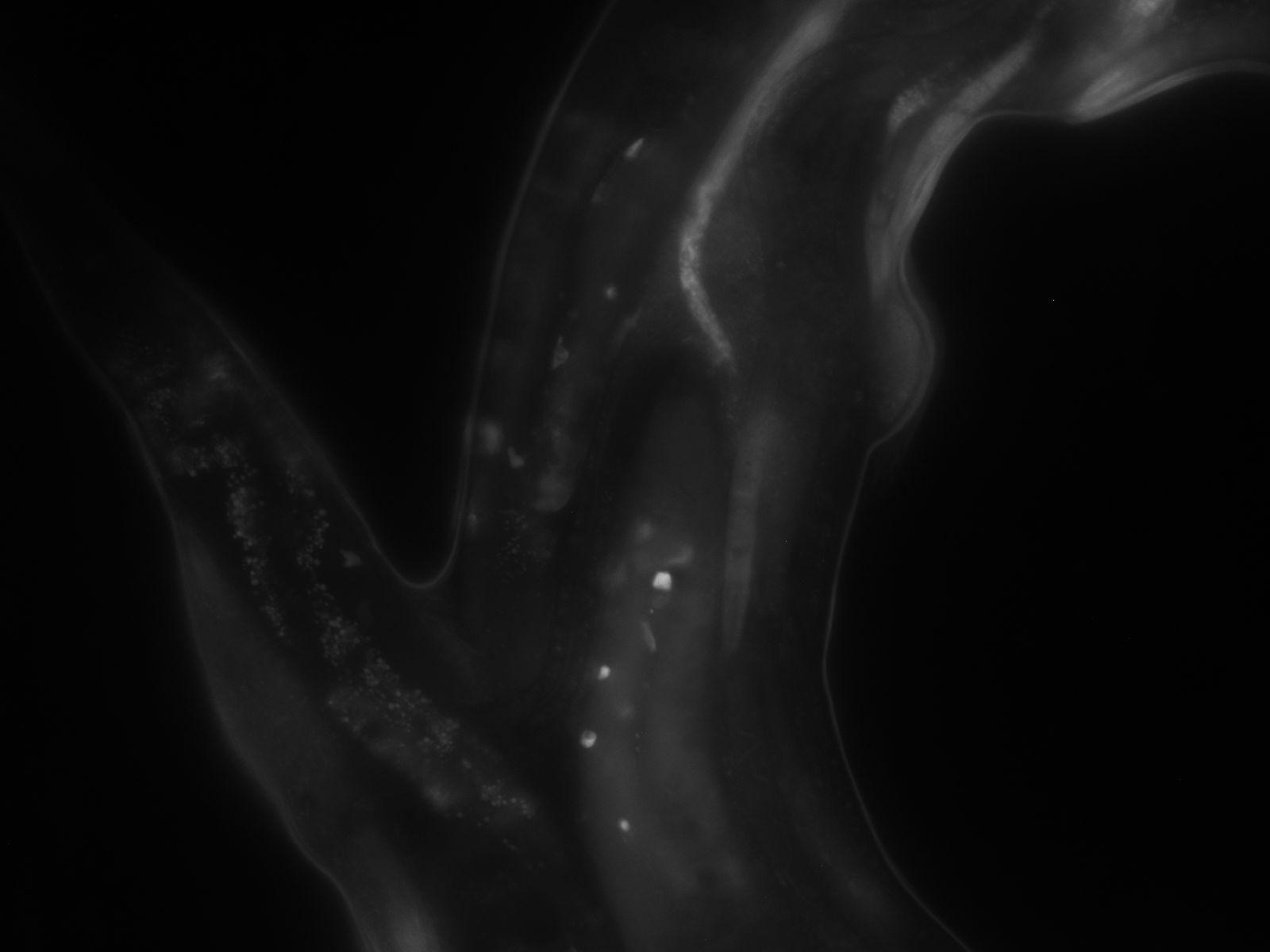

Supplement: S6 File — (ZIP) [file pgen.1011061.s006.zip › Fig.S4B+C - Original files/Fig.S4 RAW data and photos - JPEG/syto12 staining - FigS4bc - 1_rep - 14.5.23/unc-31_unc-64+tfg-1329.jpg]

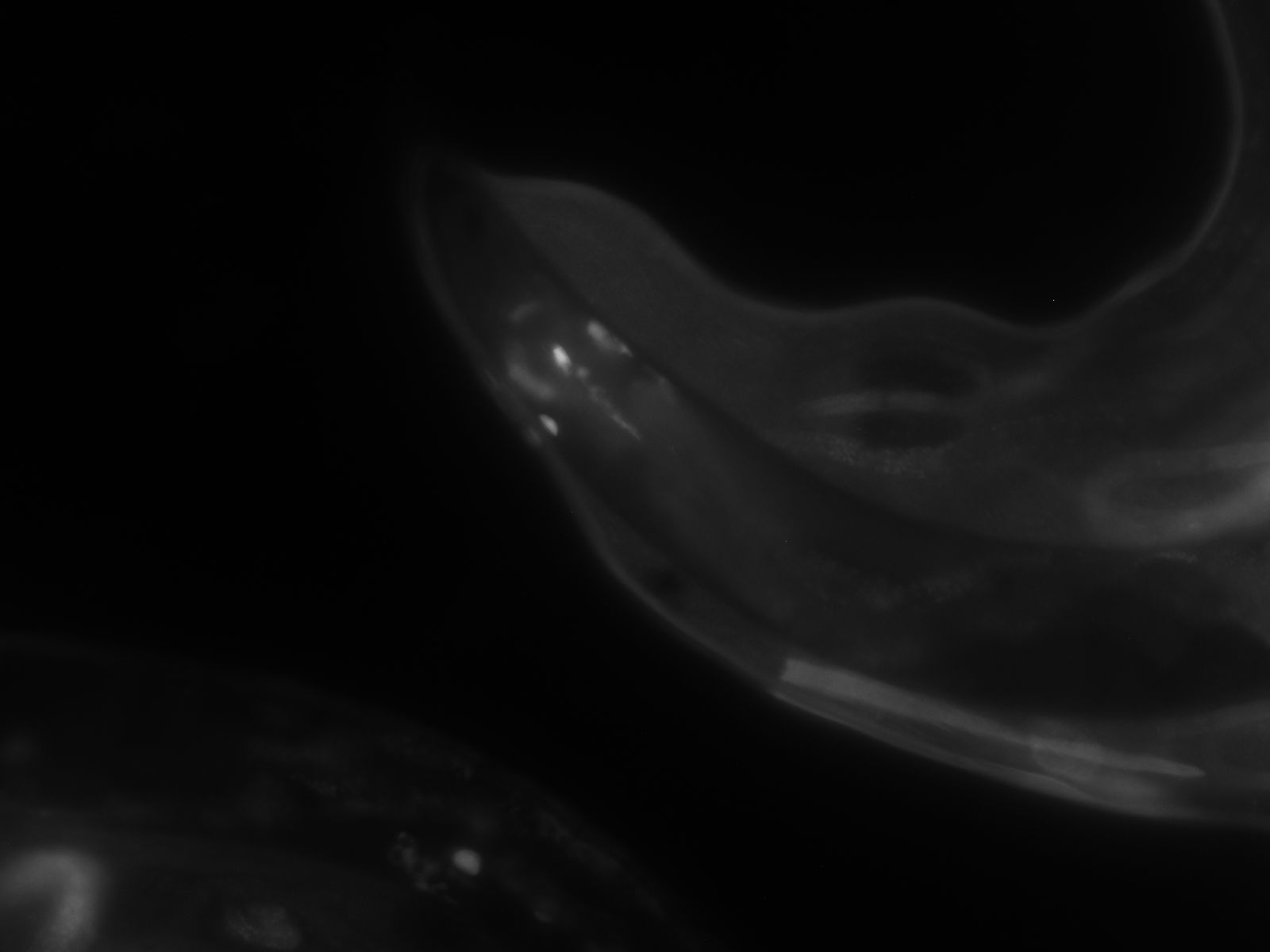

Supplement: S6 File — (ZIP) [file pgen.1011061.s006.zip › Fig.S4B+C - Original files/Fig.S4 RAW data and photos - JPEG/syto12 staining - FigS4bc - 1_rep - 14.5.23/unc-31_unc-64+tfg-1330.jpg]

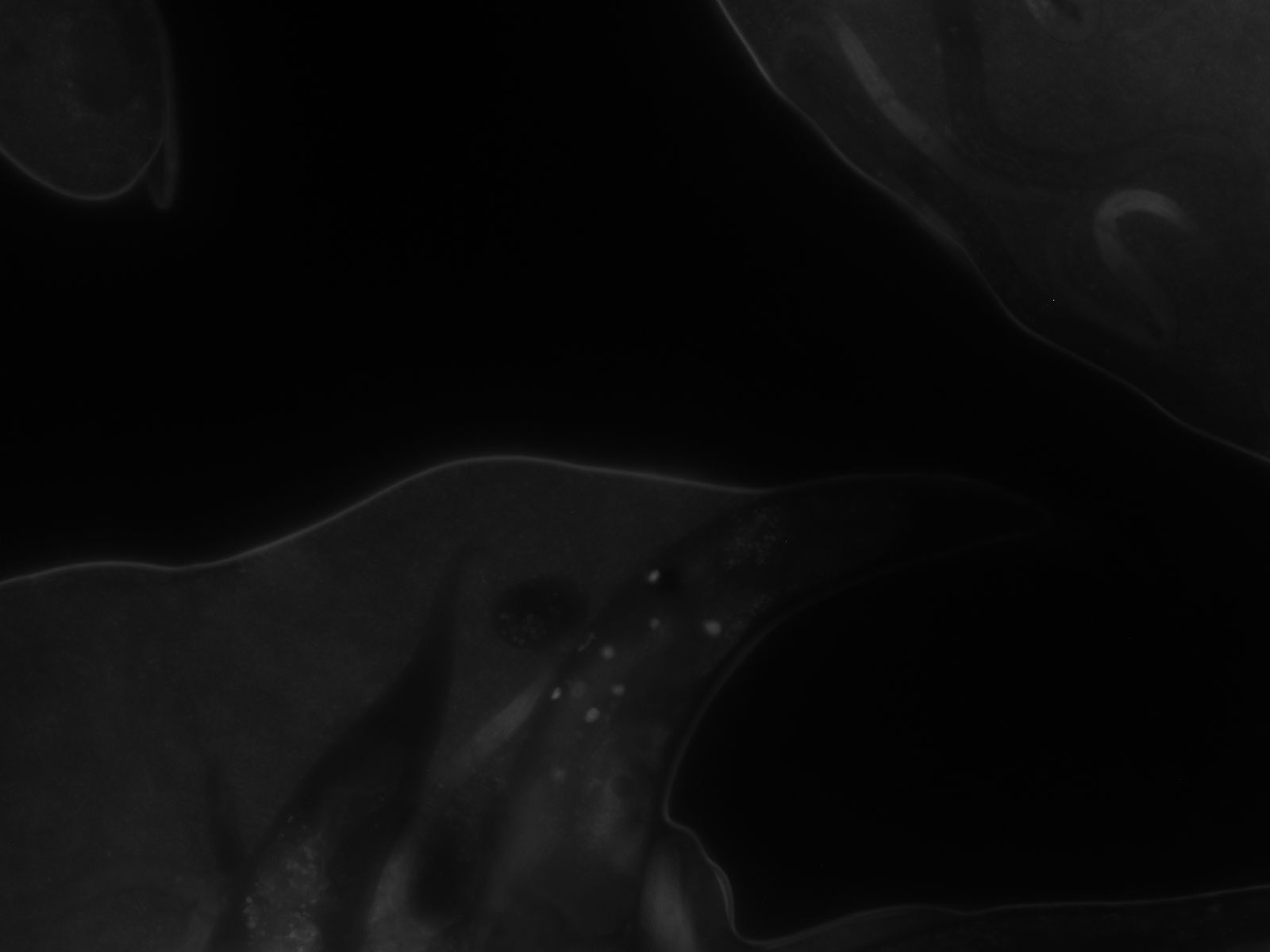

Supplement: S6 File — (ZIP) [file pgen.1011061.s006.zip › Fig.S4B+C - Original files/Fig.S4 RAW data and photos - JPEG/syto12 staining - FigS4bc - 1_rep - 14.5.23/unc-31_unc-64+tfg-1331.jpg]

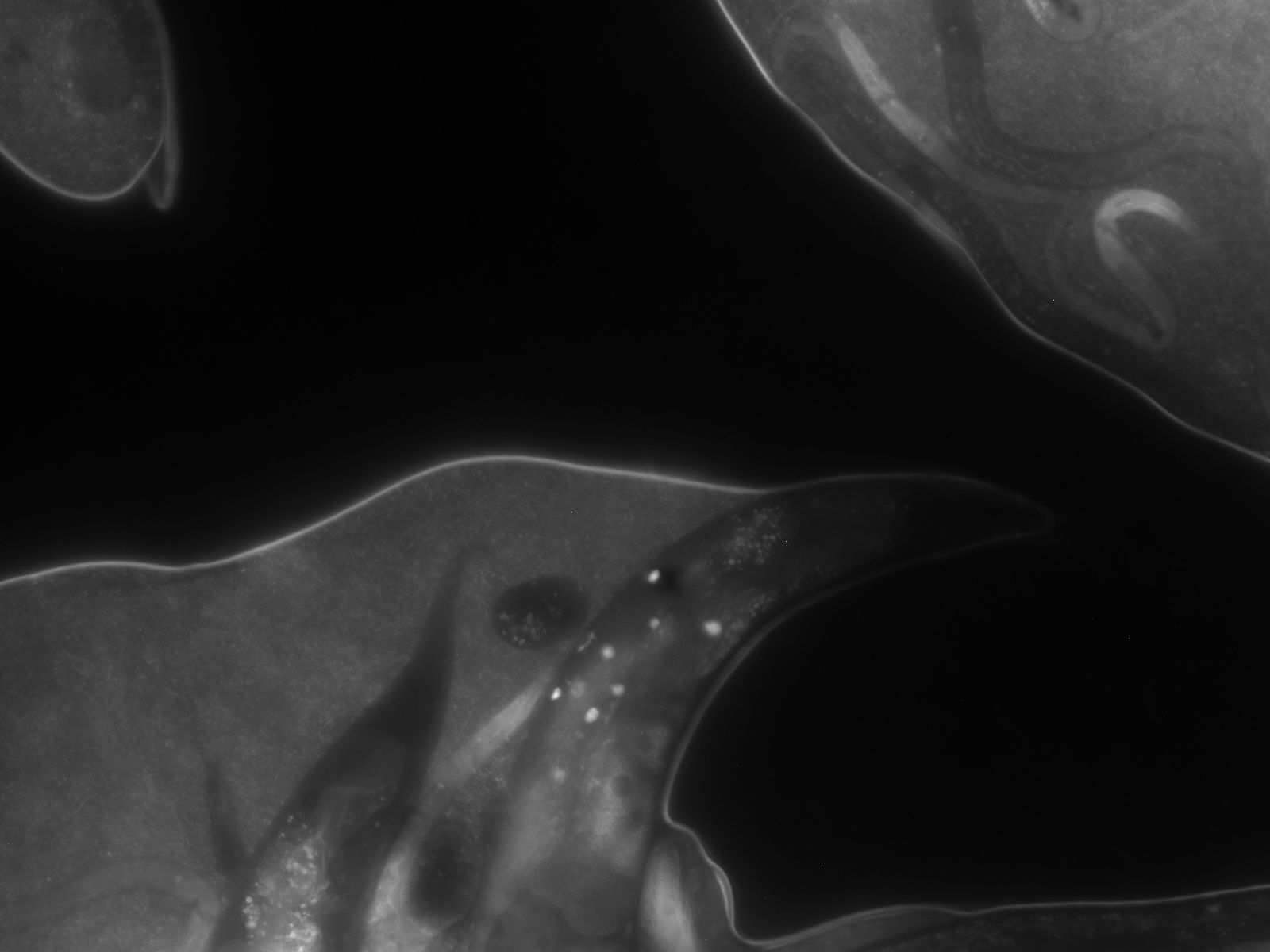

Supplement: S6 File — (ZIP) [file pgen.1011061.s006.zip › Fig.S4B+C - Original files/Fig.S4 RAW data and photos - JPEG/syto12 staining - FigS4bc - 1_rep - 14.5.23/unc-31_unc-64+tfg-1332.jpg]

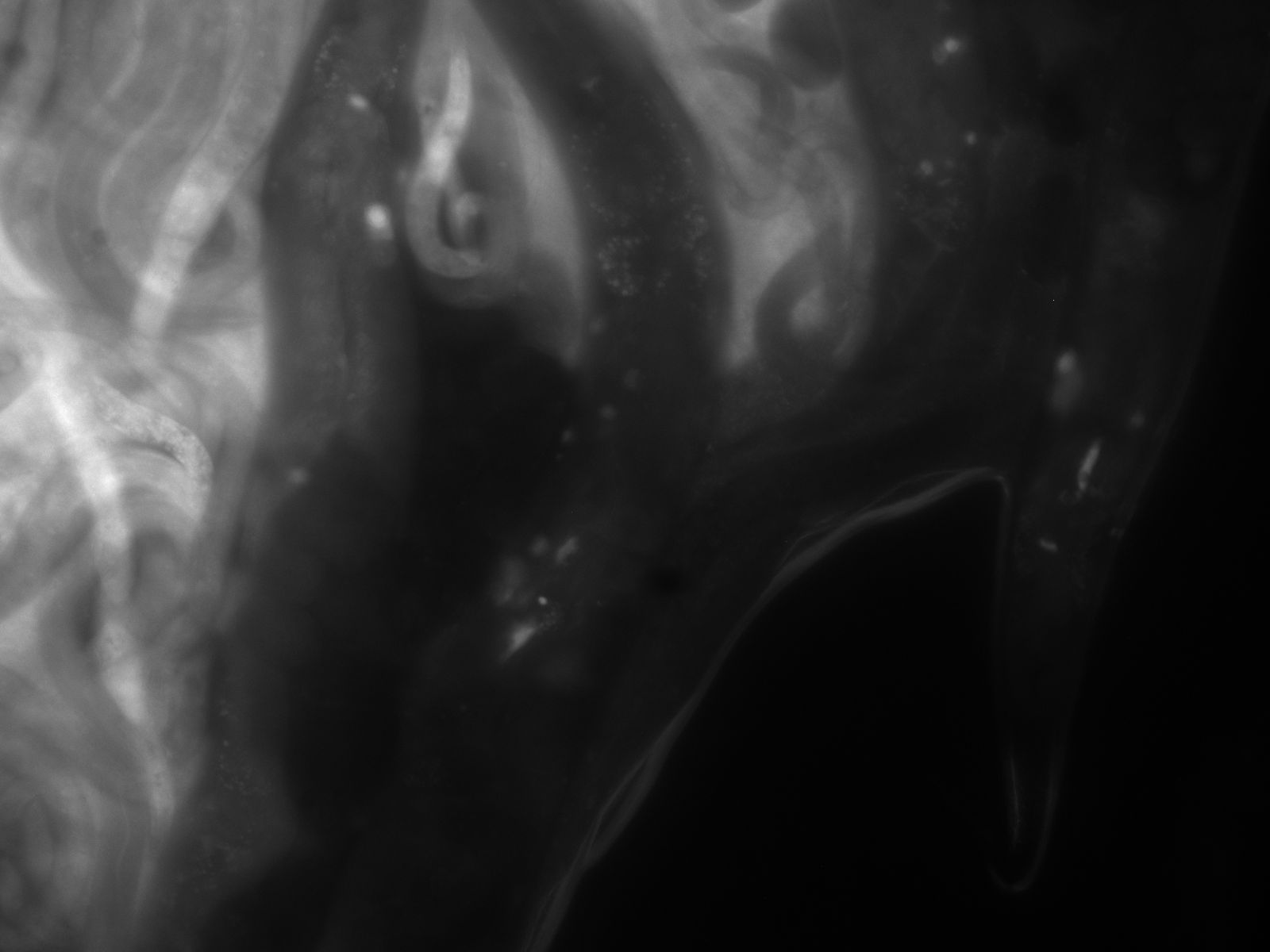

Supplement: S6 File — (ZIP) [file pgen.1011061.s006.zip › Fig.S4B+C - Original files/Fig.S4 RAW data and photos - JPEG/syto12 staining - FigS4bc - 1_rep - 14.5.23/unc-31_unc-64+tfg-1333.jpg]

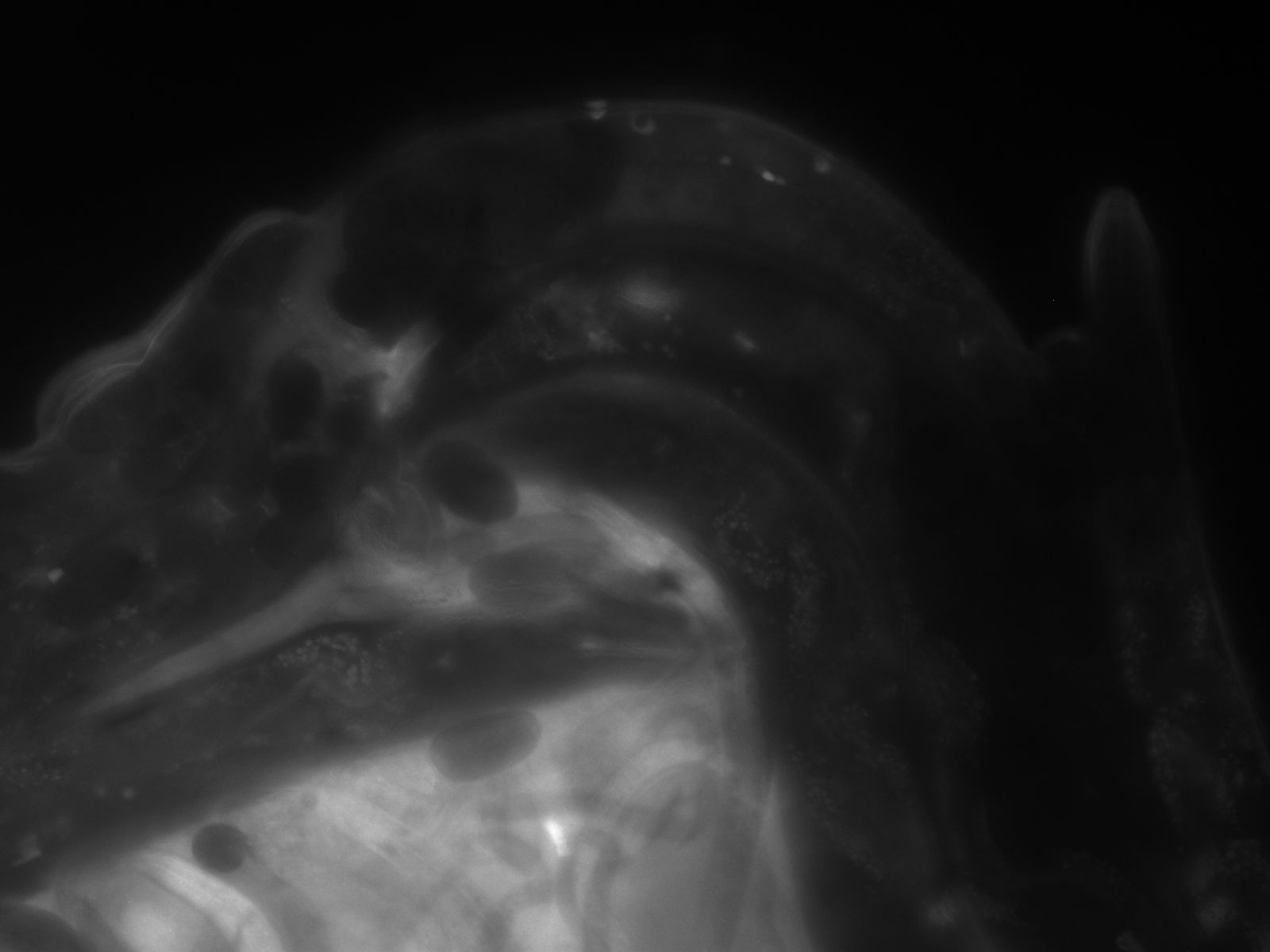

Supplement: S6 File — (ZIP) [file pgen.1011061.s006.zip › Fig.S4B+C - Original files/Fig.S4 RAW data and photos - JPEG/syto12 staining - FigS4bc - 1_rep - 14.5.23/unc-31_unc-64+tfg-1334.jpg]

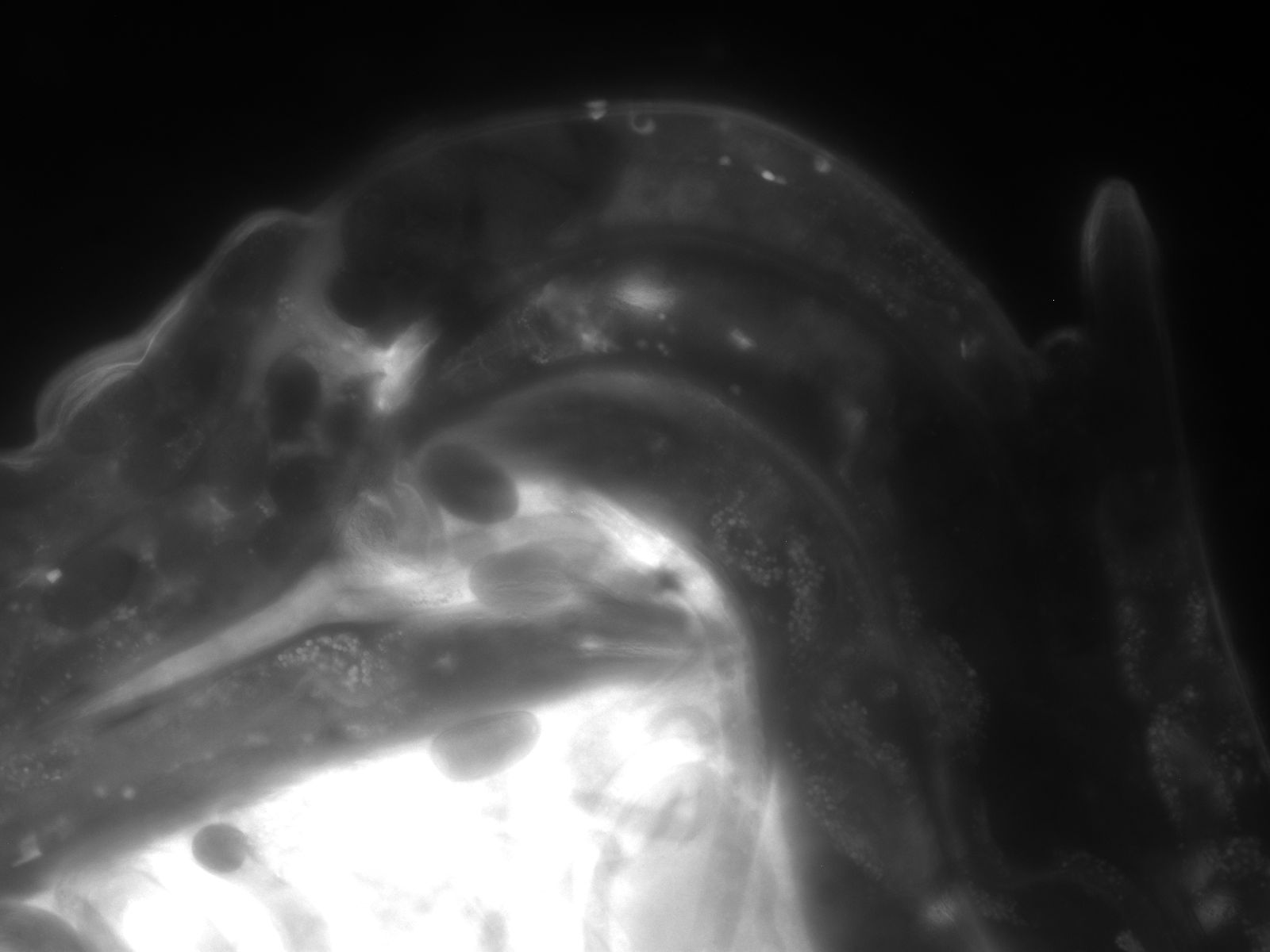

Supplement: S6 File — (ZIP) [file pgen.1011061.s006.zip › Fig.S4B+C - Original files/Fig.S4 RAW data and photos - JPEG/syto12 staining - FigS4bc - 1_rep - 14.5.23/unc-31_unc-64+tfg-1335.jpg]

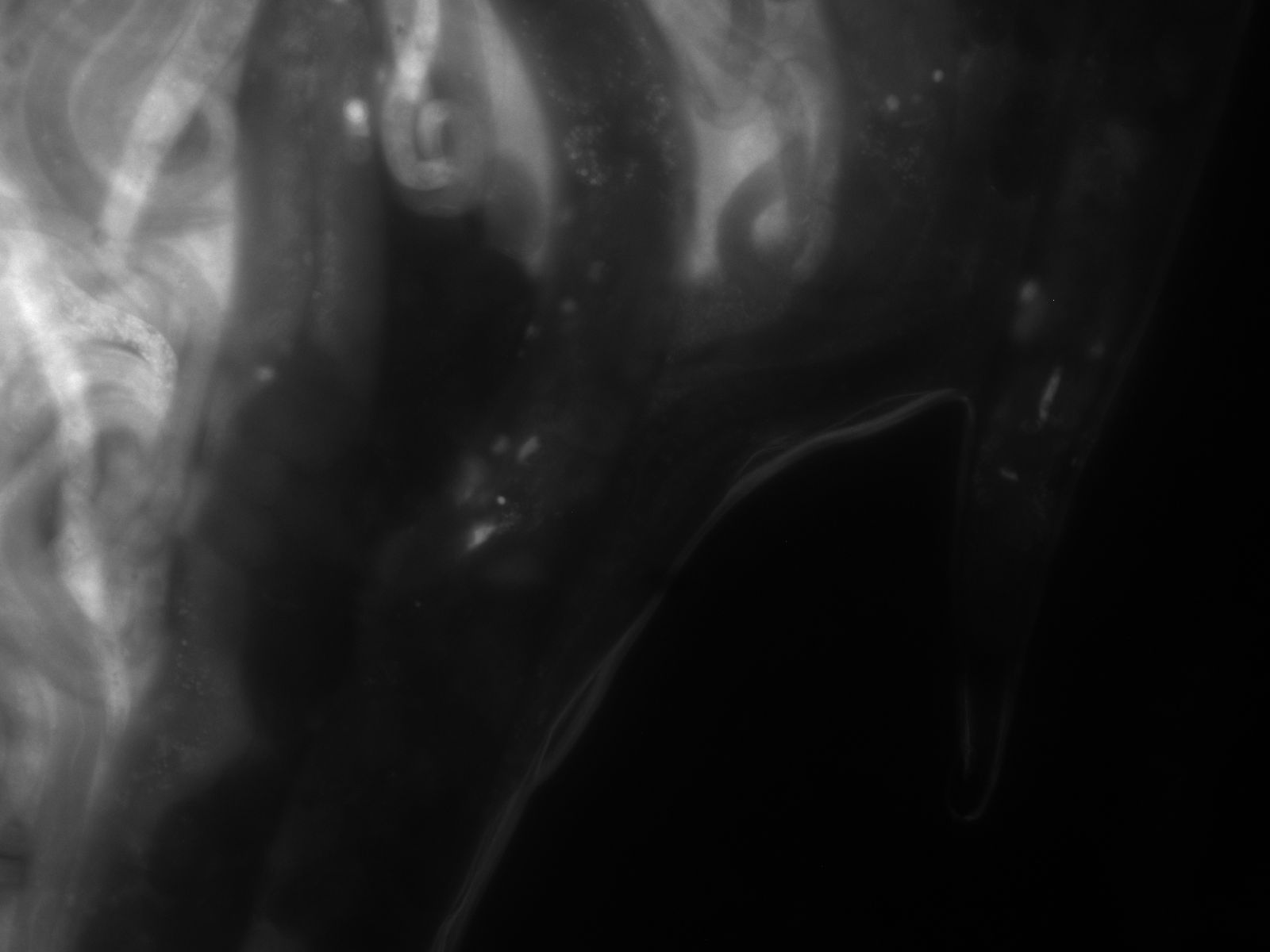

Supplement: S6 File — (ZIP) [file pgen.1011061.s006.zip › Fig.S4B+C - Original files/Fig.S4 RAW data and photos - JPEG/syto12 staining - FigS4bc - 1_rep - 14.5.23/unc-31_unc-64+tfg-1336.jpg]

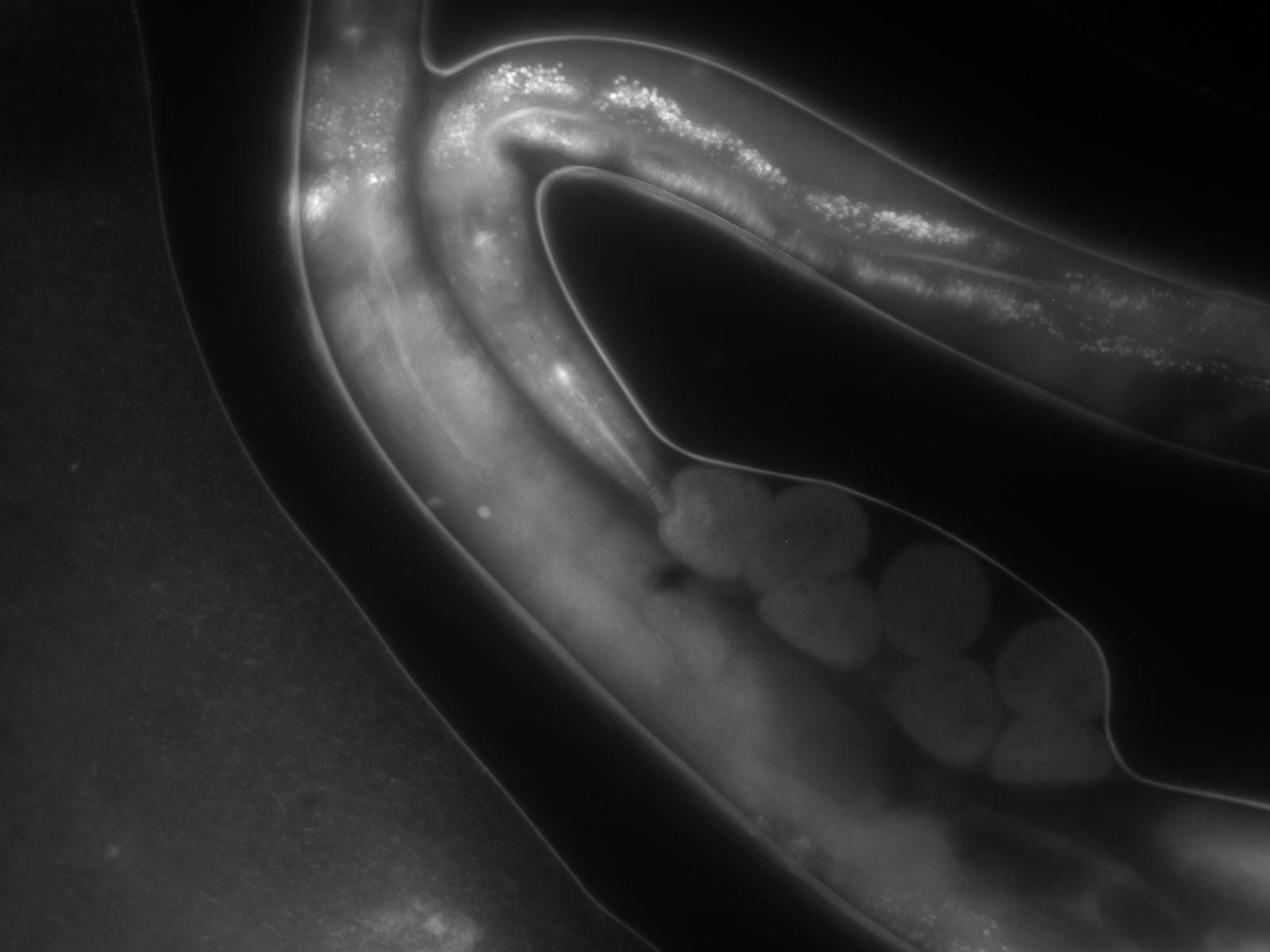

Supplement: S6 File — (ZIP) [file pgen.1011061.s006.zip › Fig.S4B+C - Original files/Fig.S4 RAW data and photos - JPEG/syto12 staining - FigS4bc - 2 rep_15.5.23/eat-4+pad1253.jpg]

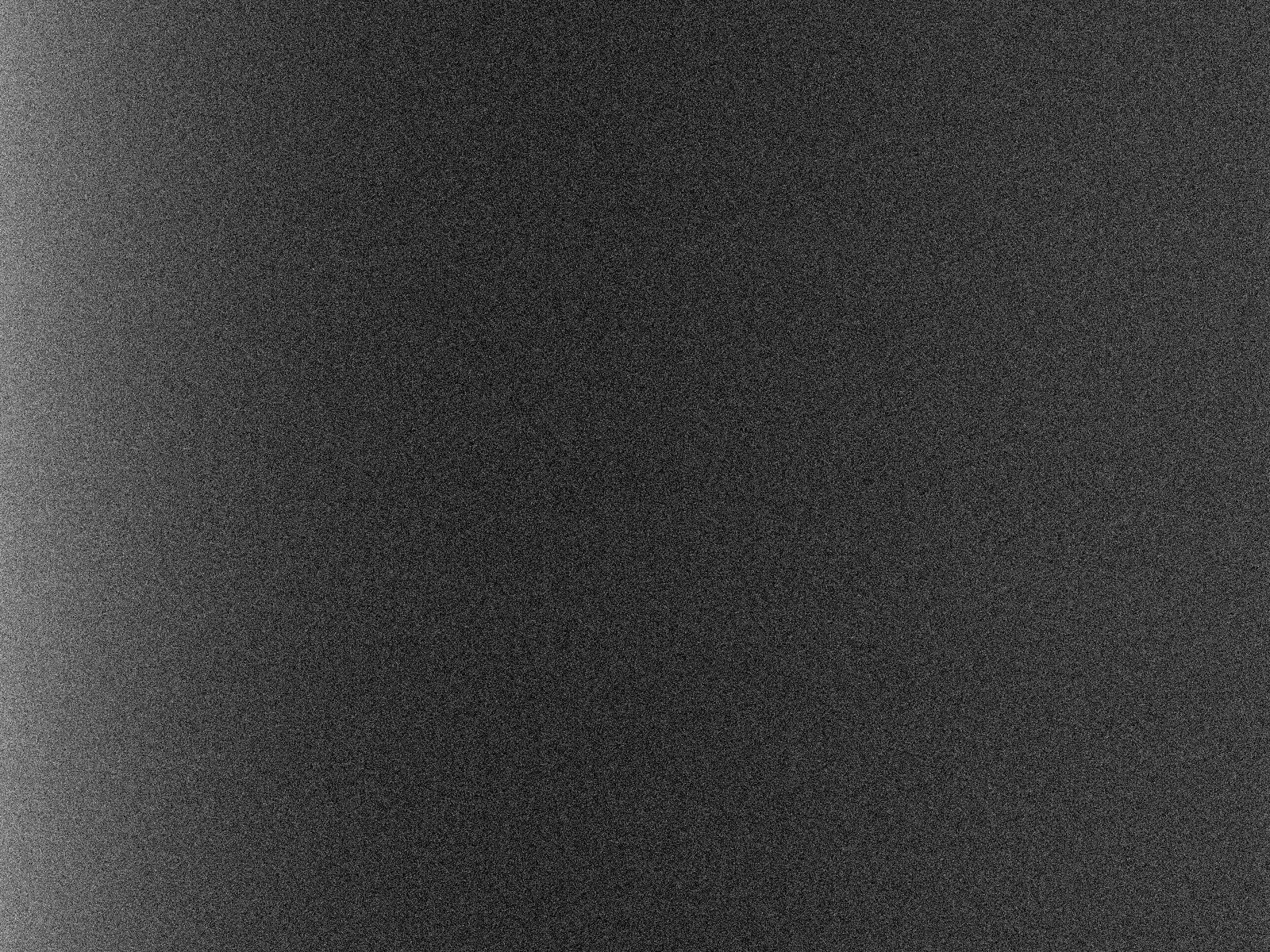

Supplement: S6 File — (ZIP) [file pgen.1011061.s006.zip › Fig.S4B+C - Original files/Fig.S4 RAW data and photos - JPEG/syto12 staining - FigS4bc - 2 rep_15.5.23/eat-4+tfg-154.jpg]

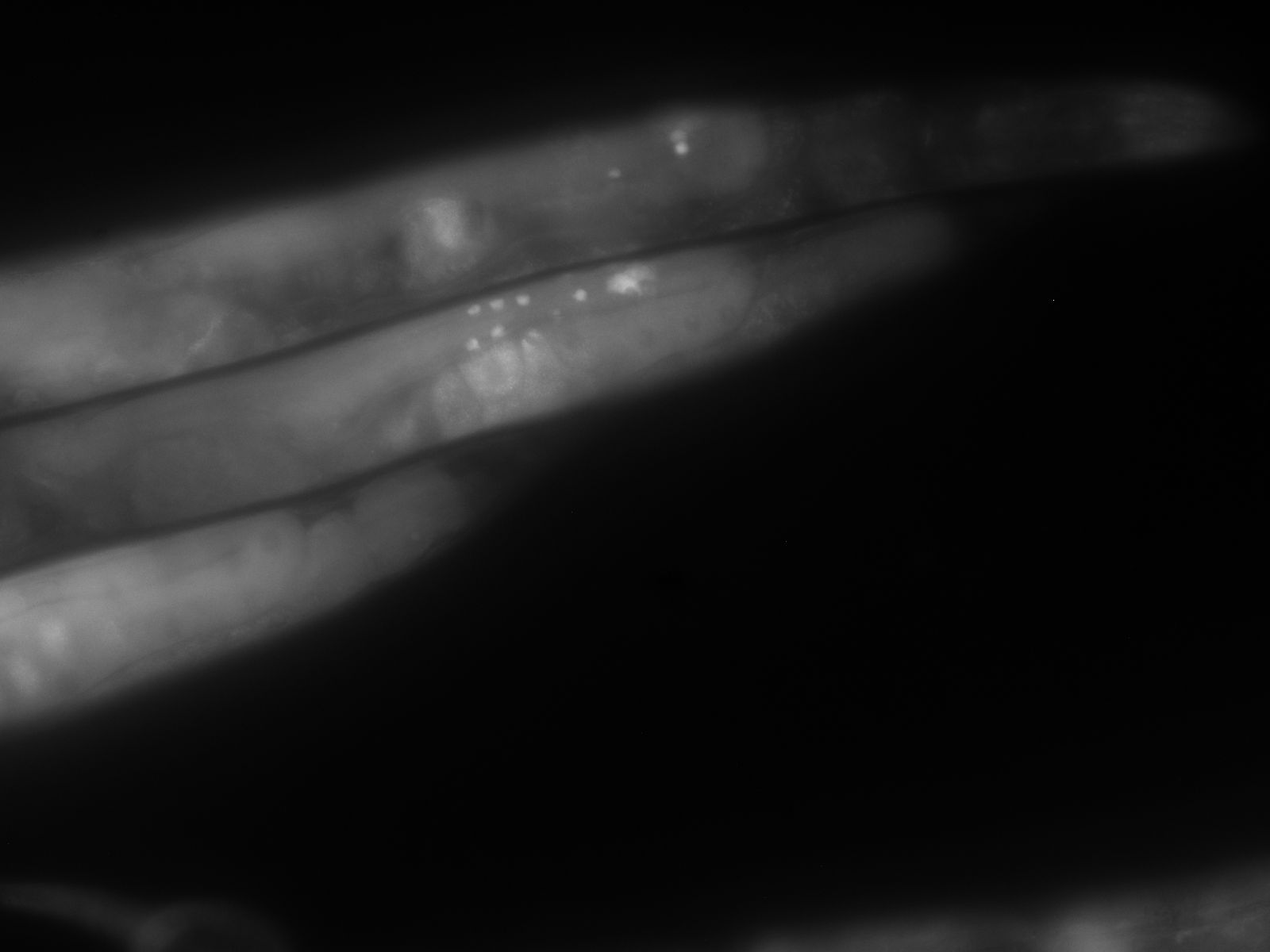

Supplement: S6 File — (ZIP) [file pgen.1011061.s006.zip › Fig.S4B+C - Original files/Fig.S4 RAW data and photos - JPEG/syto12 staining - FigS4bc - 2 rep_15.5.23/eat-4+tfg-155.jpg]

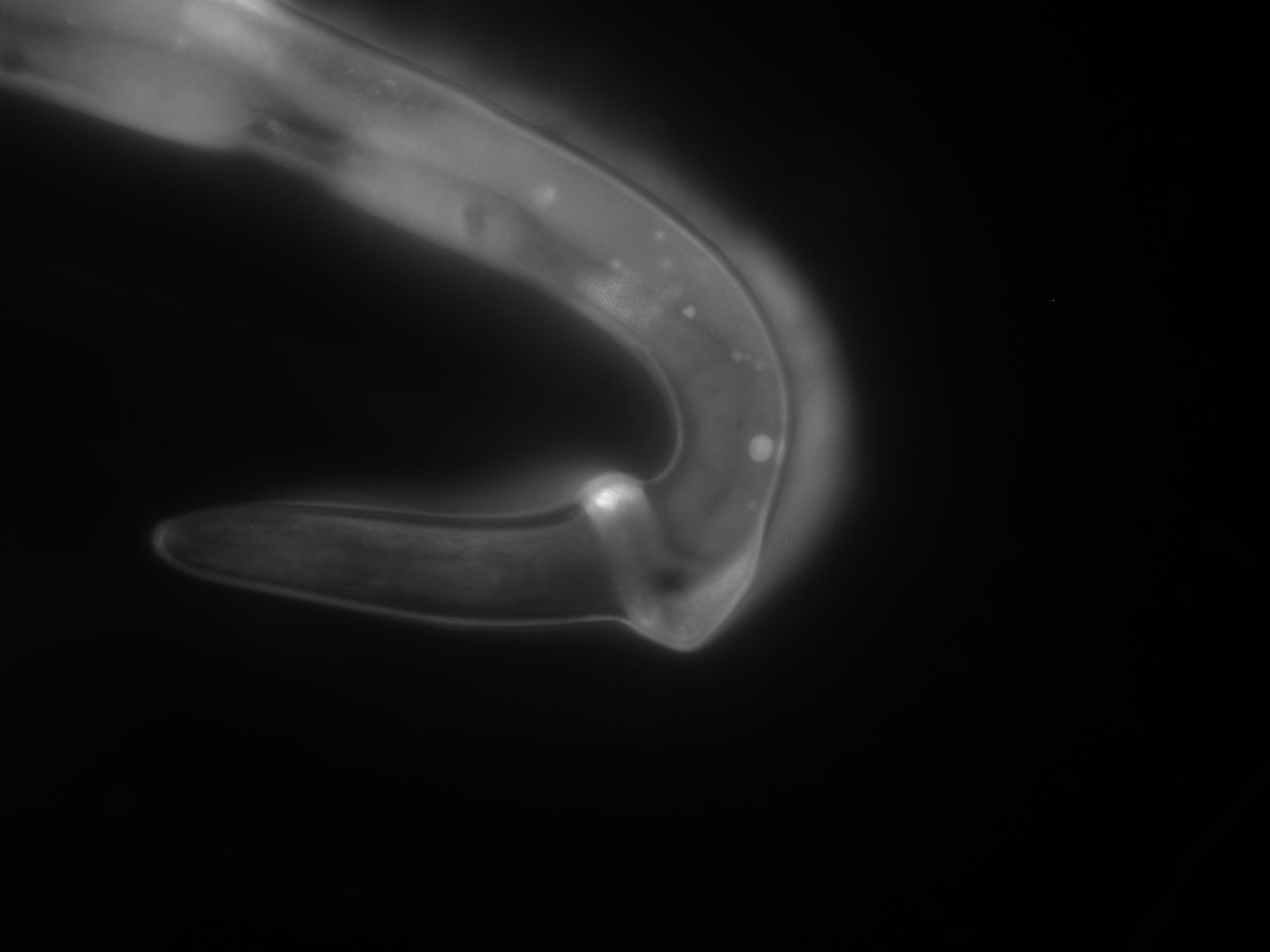

Supplement: S6 File — (ZIP) [file pgen.1011061.s006.zip › Fig.S4B+C - Original files/Fig.S4 RAW data and photos - JPEG/syto12 staining - FigS4bc - 2 rep_15.5.23/eat-4+tfg-156.jpg]

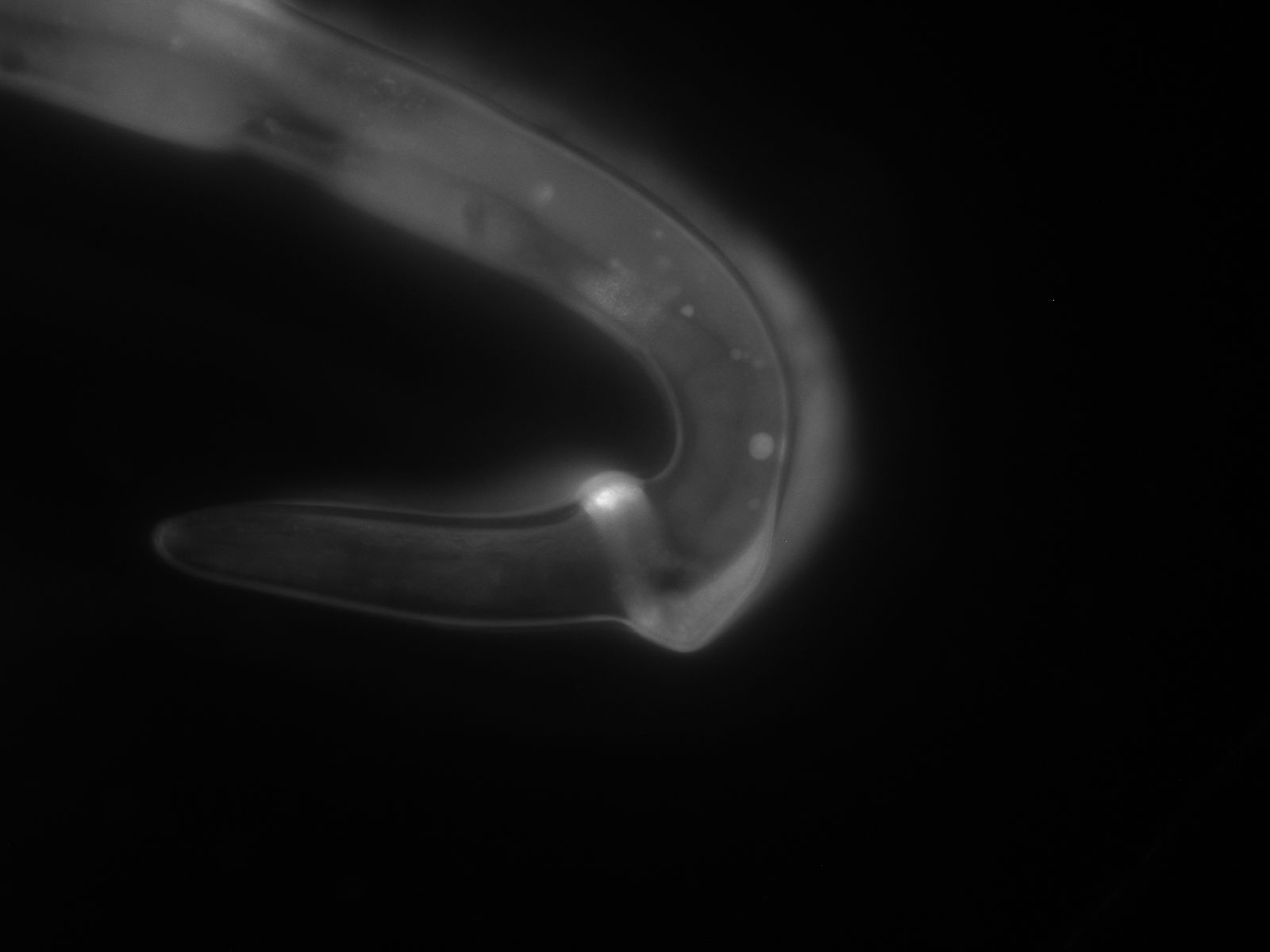

Supplement: S6 File — (ZIP) [file pgen.1011061.s006.zip › Fig.S4B+C - Original files/Fig.S4 RAW data and photos - JPEG/syto12 staining - FigS4bc - 2 rep_15.5.23/eat-4+tfg-157.jpg]

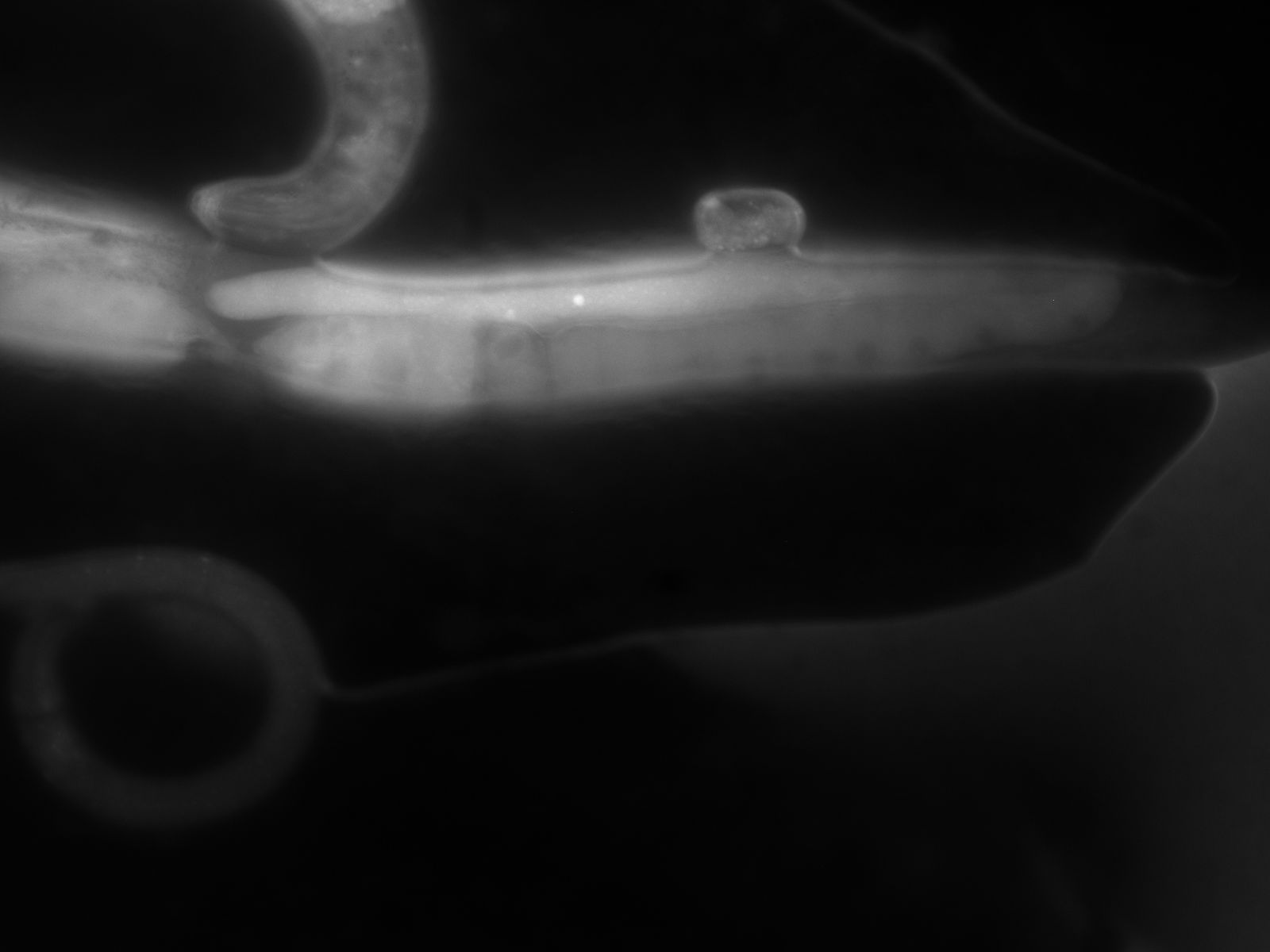

Supplement: S6 File — (ZIP) [file pgen.1011061.s006.zip › Fig.S4B+C - Original files/Fig.S4 RAW data and photos - JPEG/syto12 staining - FigS4bc - 2 rep_15.5.23/unc-13+pad1237.jpg]

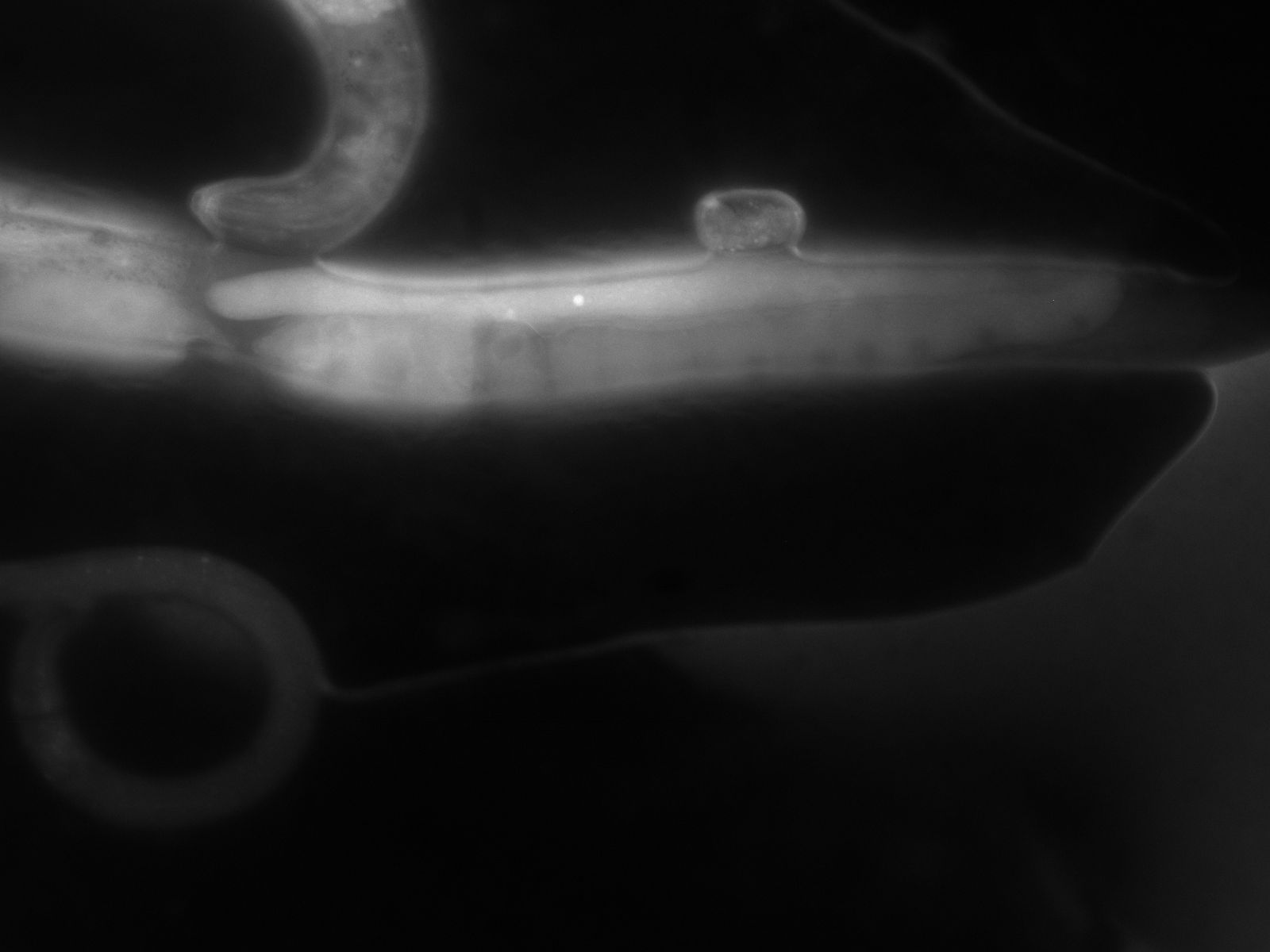

Supplement: S6 File — (ZIP) [file pgen.1011061.s006.zip › Fig.S4B+C - Original files/Fig.S4 RAW data and photos - JPEG/syto12 staining - FigS4bc - 2 rep_15.5.23/unc-13+pad1238.jpg]

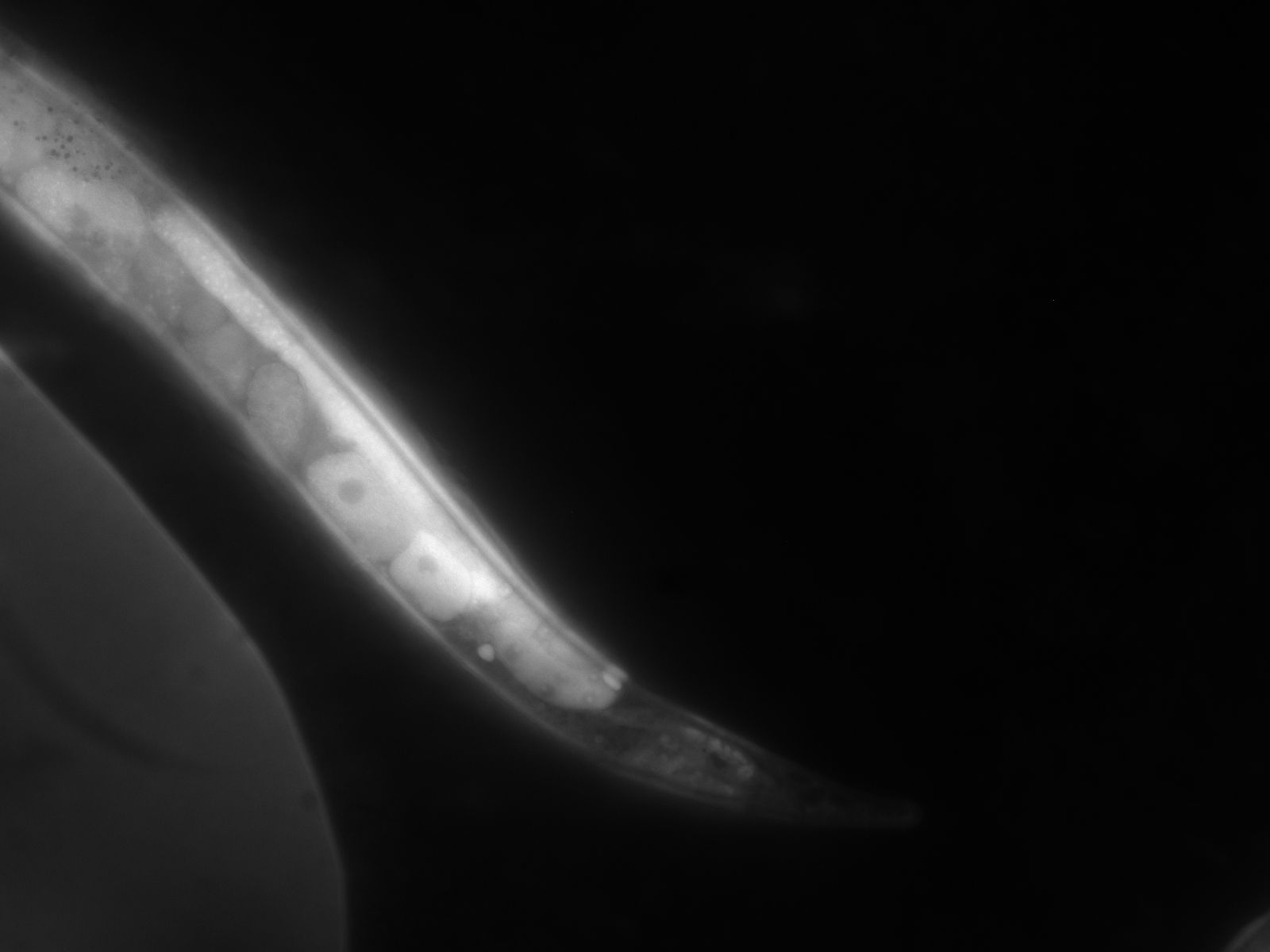

Supplement: S6 File — (ZIP) [file pgen.1011061.s006.zip › Fig.S4B+C - Original files/Fig.S4 RAW data and photos - JPEG/syto12 staining - FigS4bc - 2 rep_15.5.23/unc-13+pad1239.jpg]

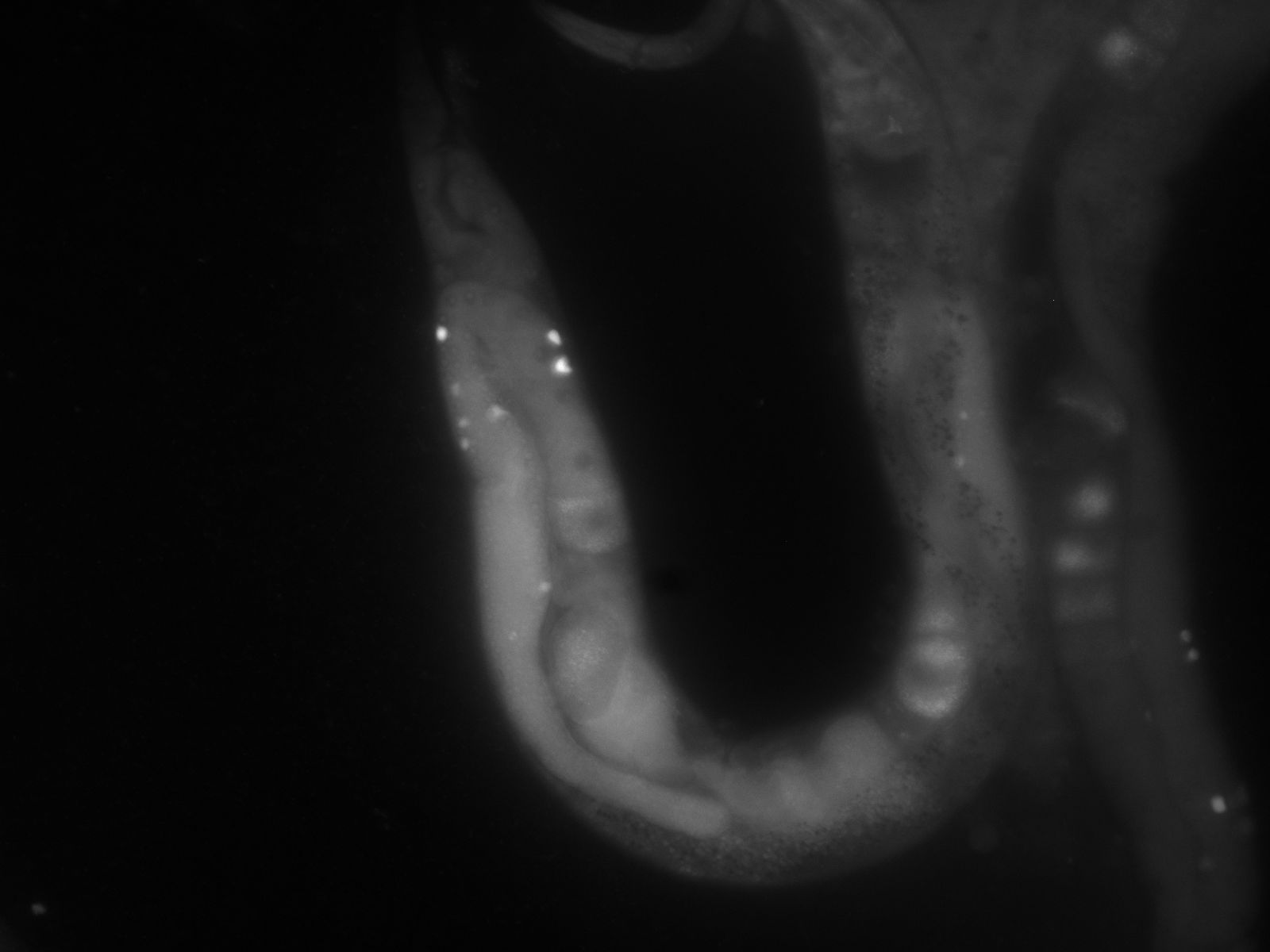

Supplement: S6 File — (ZIP) [file pgen.1011061.s006.zip › Fig.S4B+C - Original files/Fig.S4 RAW data and photos - JPEG/syto12 staining - FigS4bc - 2 rep_15.5.23/unc-13+tfg-140.jpg]

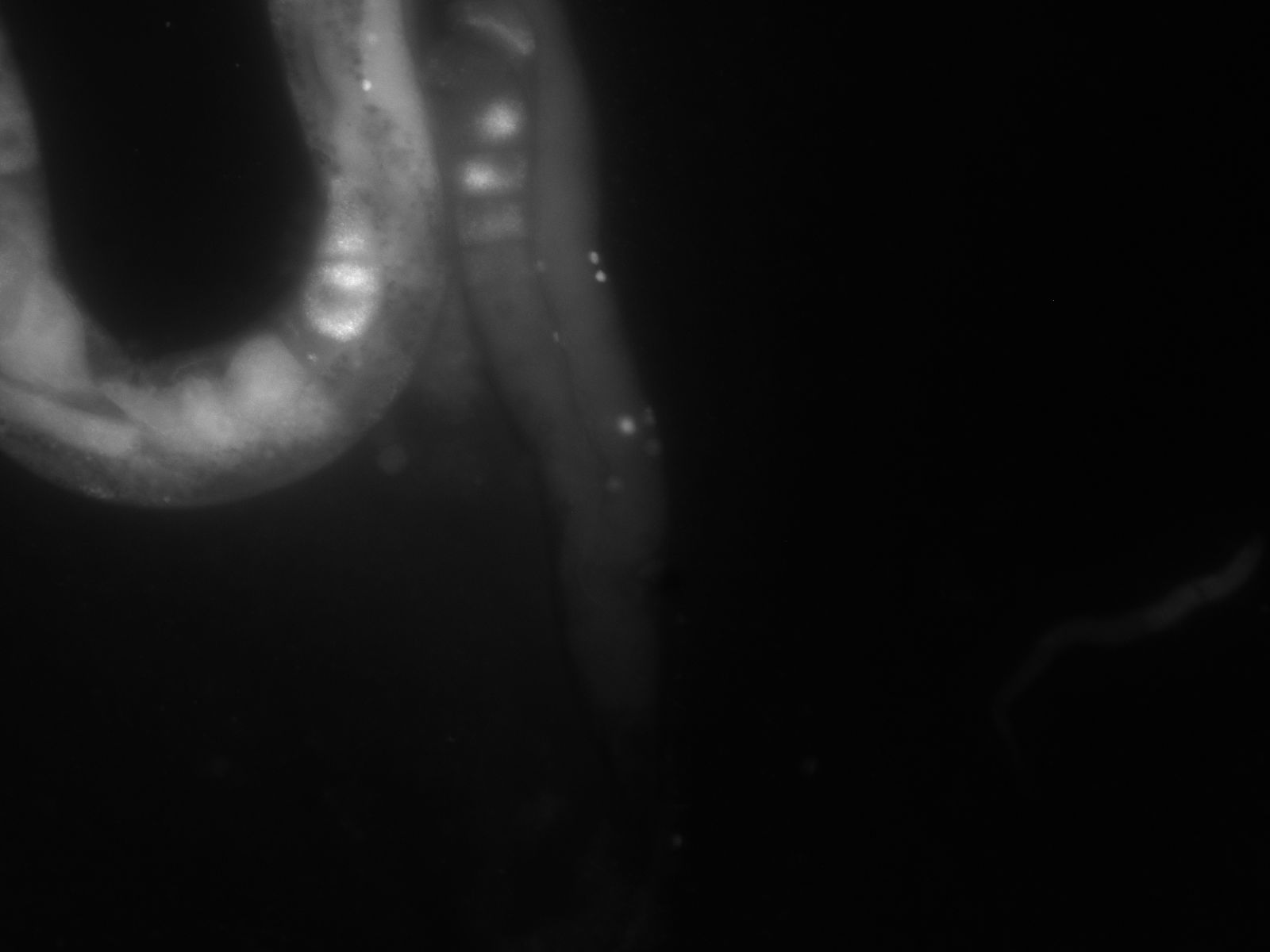

Supplement: S6 File — (ZIP) [file pgen.1011061.s006.zip › Fig.S4B+C - Original files/Fig.S4 RAW data and photos - JPEG/syto12 staining - FigS4bc - 2 rep_15.5.23/unc-13+tfg-141.jpg]

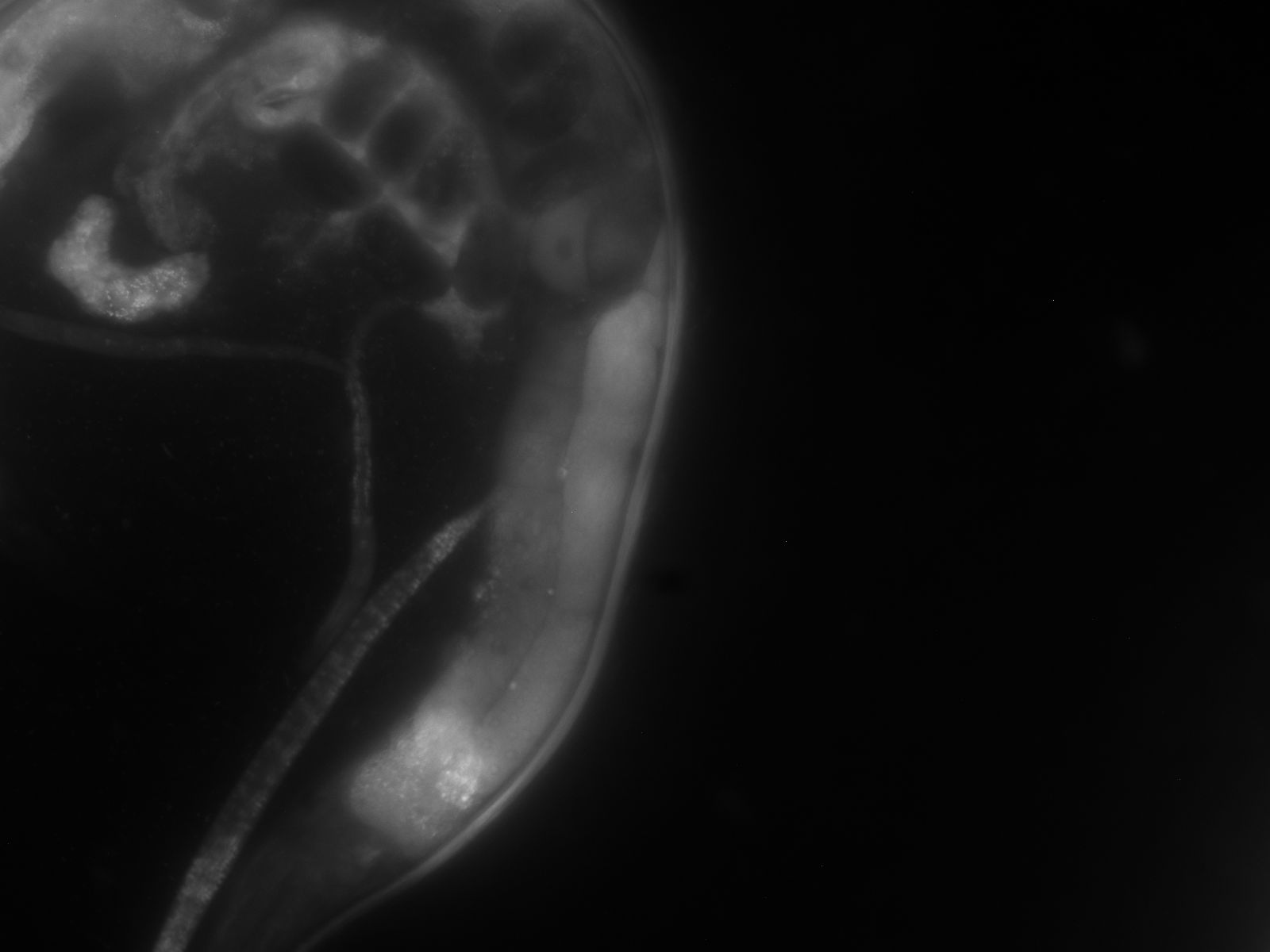

Supplement: S6 File — (ZIP) [file pgen.1011061.s006.zip › Fig.S4B+C - Original files/Fig.S4 RAW data and photos - JPEG/syto12 staining - FigS4bc - 2 rep_15.5.23/unc-31_unc-64+pad1249.jpg]

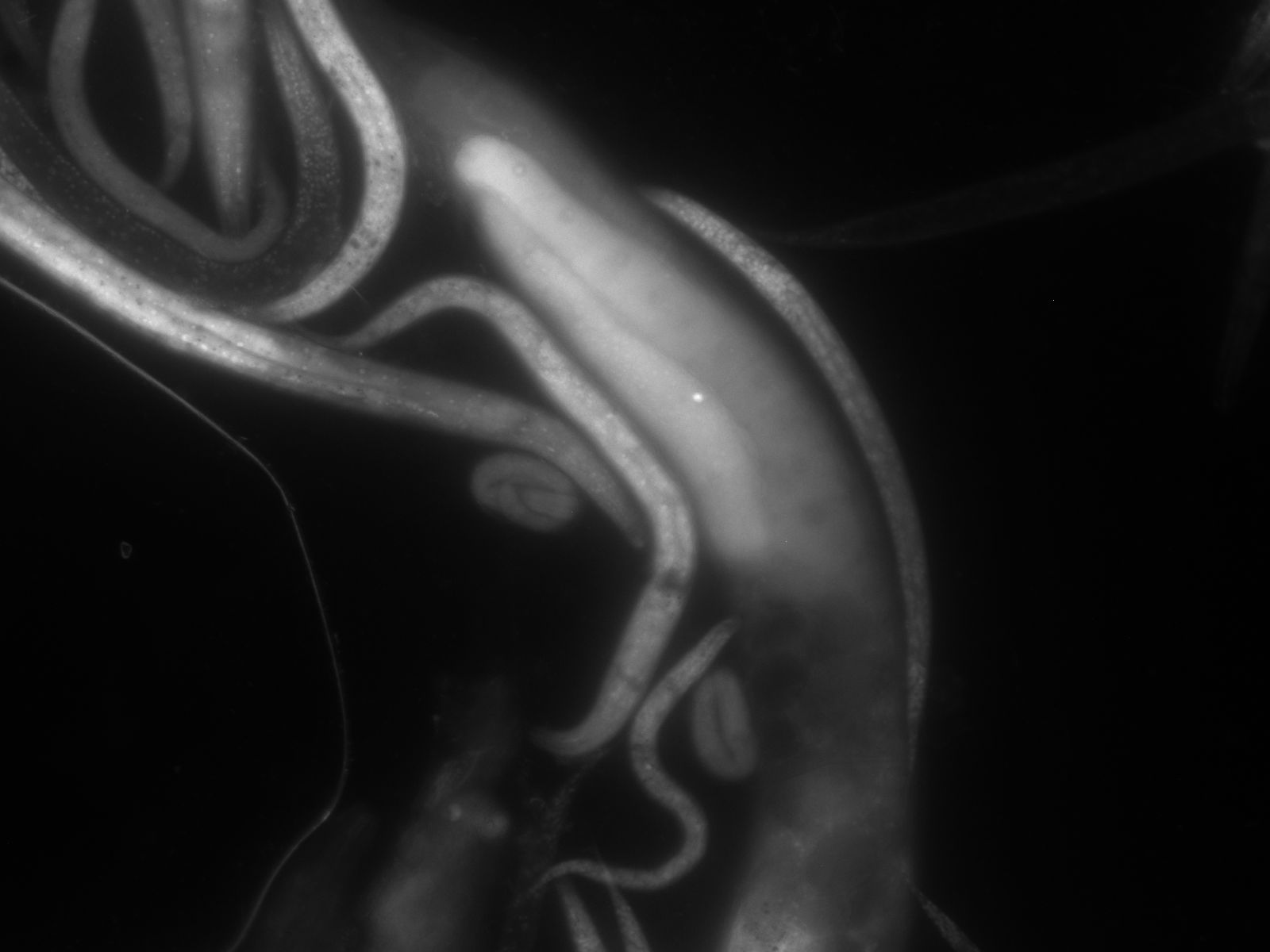

Supplement: S6 File — (ZIP) [file pgen.1011061.s006.zip › Fig.S4B+C - Original files/Fig.S4 RAW data and photos - JPEG/syto12 staining - FigS4bc - 2 rep_15.5.23/unc-31_unc-64+pad1250.jpg]

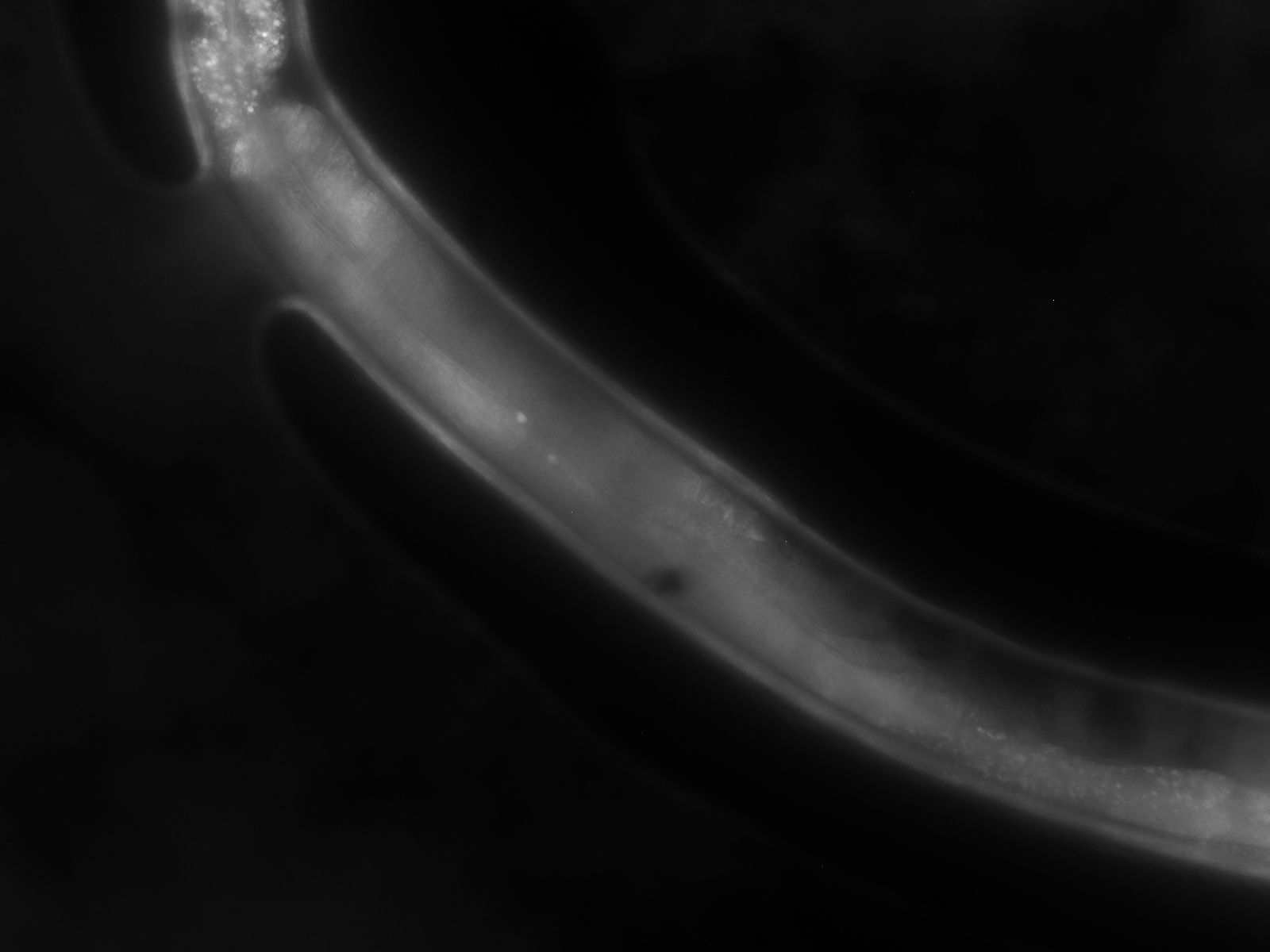

Supplement: S6 File — (ZIP) [file pgen.1011061.s006.zip › Fig.S4B+C - Original files/Fig.S4 RAW data and photos - JPEG/syto12 staining - FigS4bc - 2 rep_15.5.23/unc-31_unc-64+pad1251.jpg]

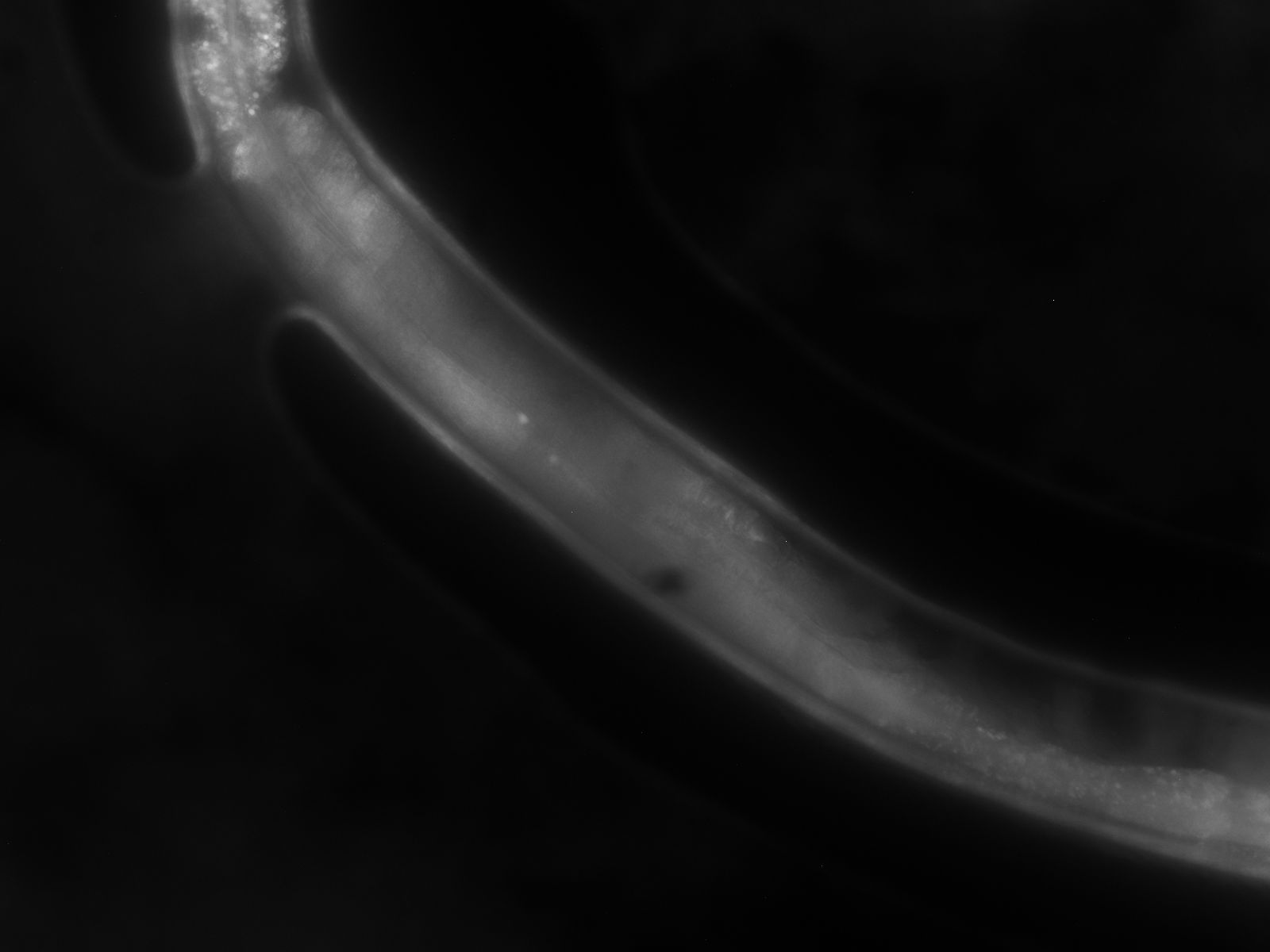

Supplement: S6 File — (ZIP) [file pgen.1011061.s006.zip › Fig.S4B+C - Original files/Fig.S4 RAW data and photos - JPEG/syto12 staining - FigS4bc - 2 rep_15.5.23/unc-31_unc-64+pad1252.jpg]

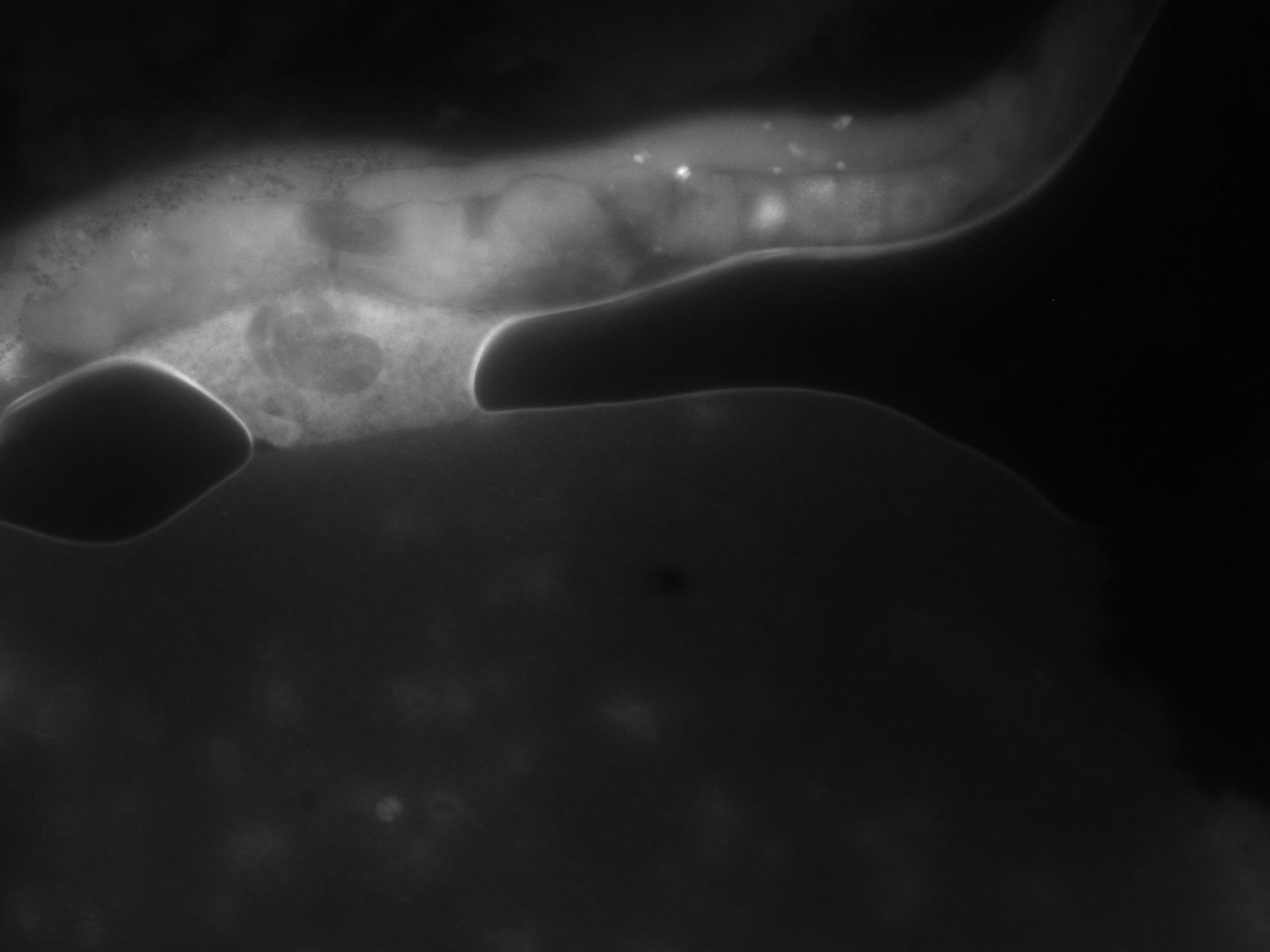

Supplement: S6 File — (ZIP) [file pgen.1011061.s006.zip › Fig.S4B+C - Original files/Fig.S4 RAW data and photos - JPEG/syto12 staining - FigS4bc - 2 rep_15.5.23/unc-31_unc-64+tfg-142.jpg]

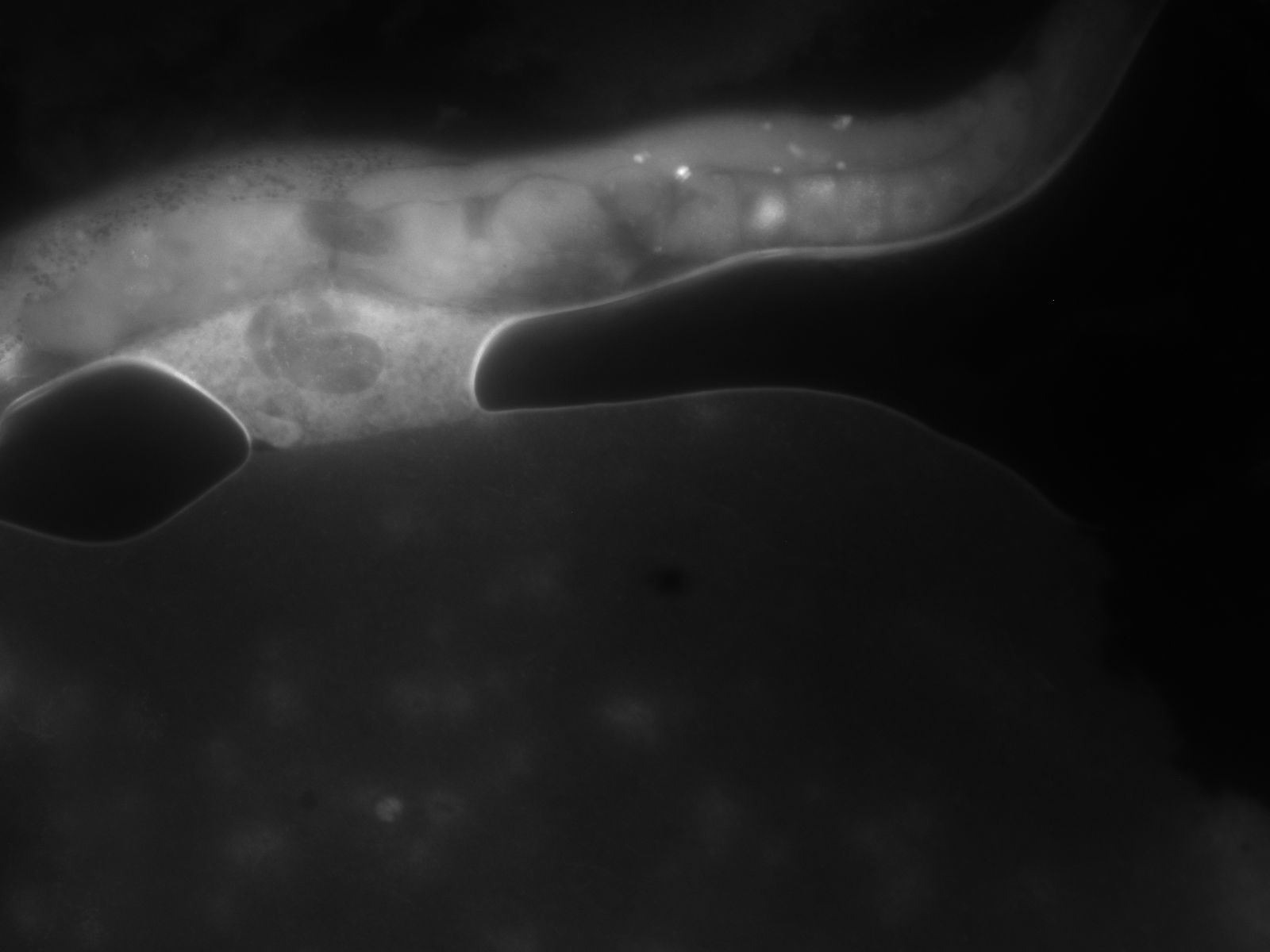

Supplement: S6 File — (ZIP) [file pgen.1011061.s006.zip › Fig.S4B+C - Original files/Fig.S4 RAW data and photos - JPEG/syto12 staining - FigS4bc - 2 rep_15.5.23/unc-31_unc-64+tfg-143.jpg]

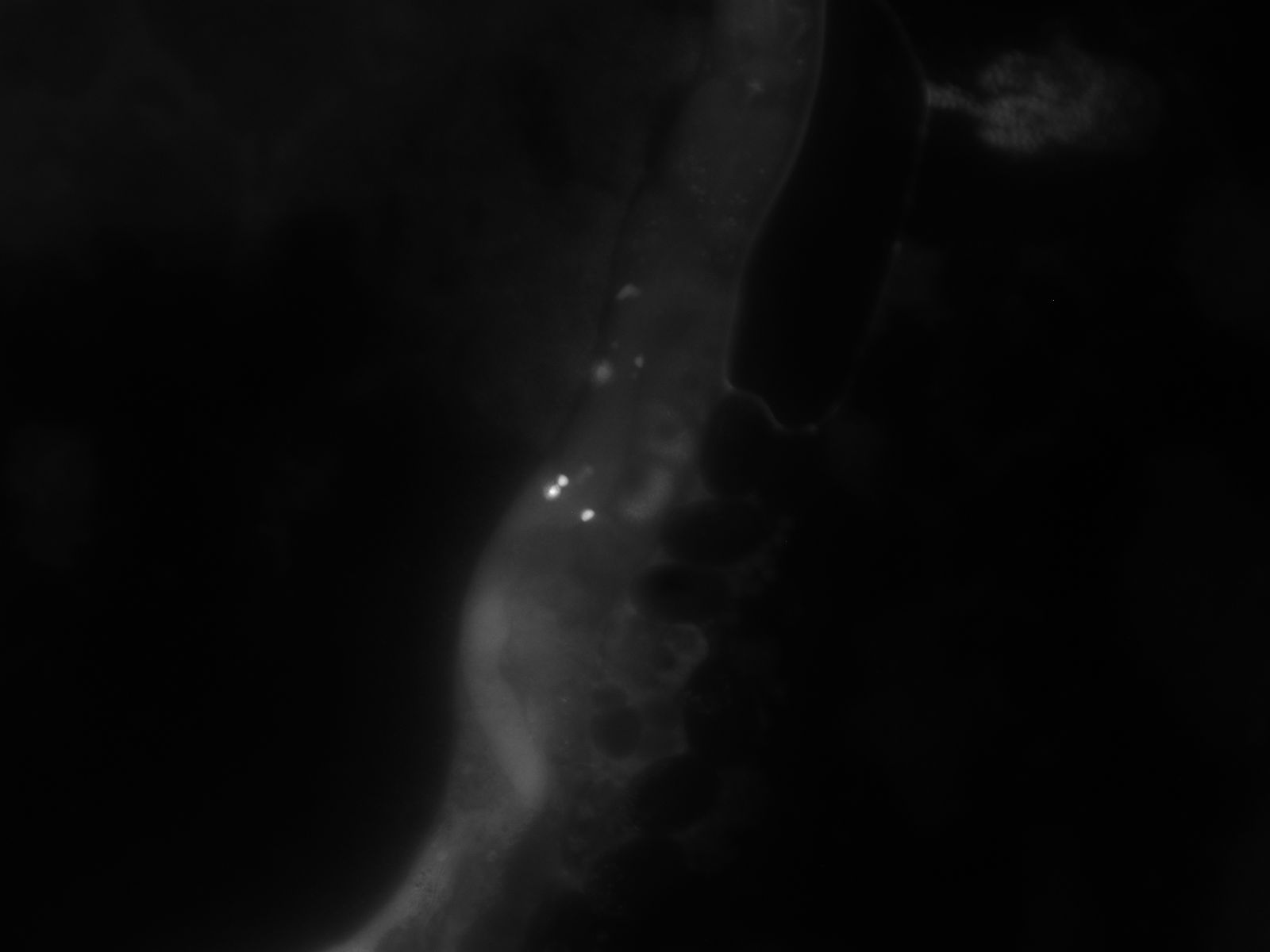

Supplement: S6 File — (ZIP) [file pgen.1011061.s006.zip › Fig.S4B+C - Original files/Fig.S4 RAW data and photos - JPEG/syto12 staining - FigS4bc - 2 rep_15.5.23/unc-31_unc-64+tfg-144.jpg]

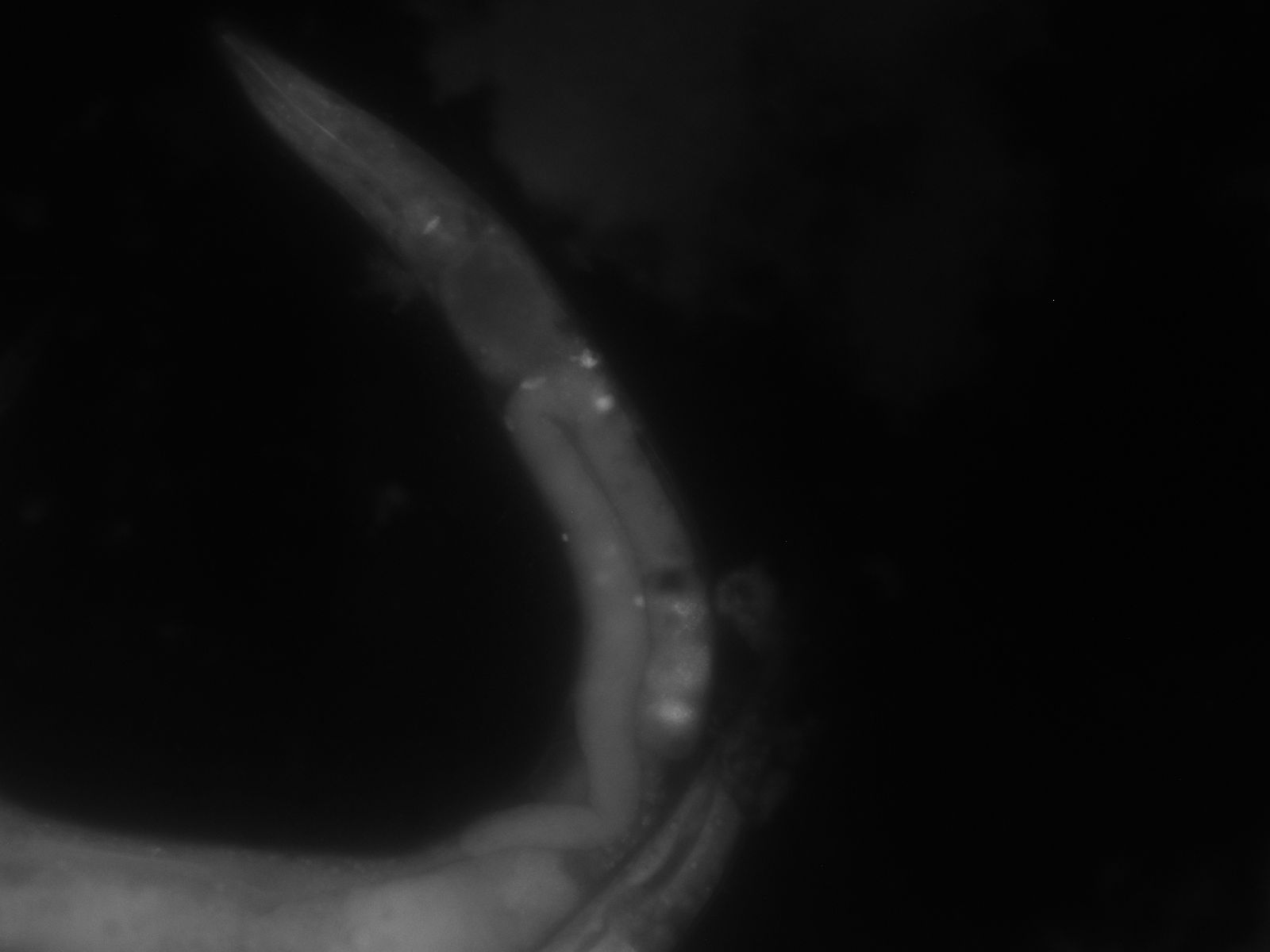

Supplement: S6 File — (ZIP) [file pgen.1011061.s006.zip › Fig.S4B+C - Original files/Fig.S4 RAW data and photos - JPEG/syto12 staining - FigS4bc - 2 rep_15.5.23/unc-31_unc-64+tfg-145.jpg]

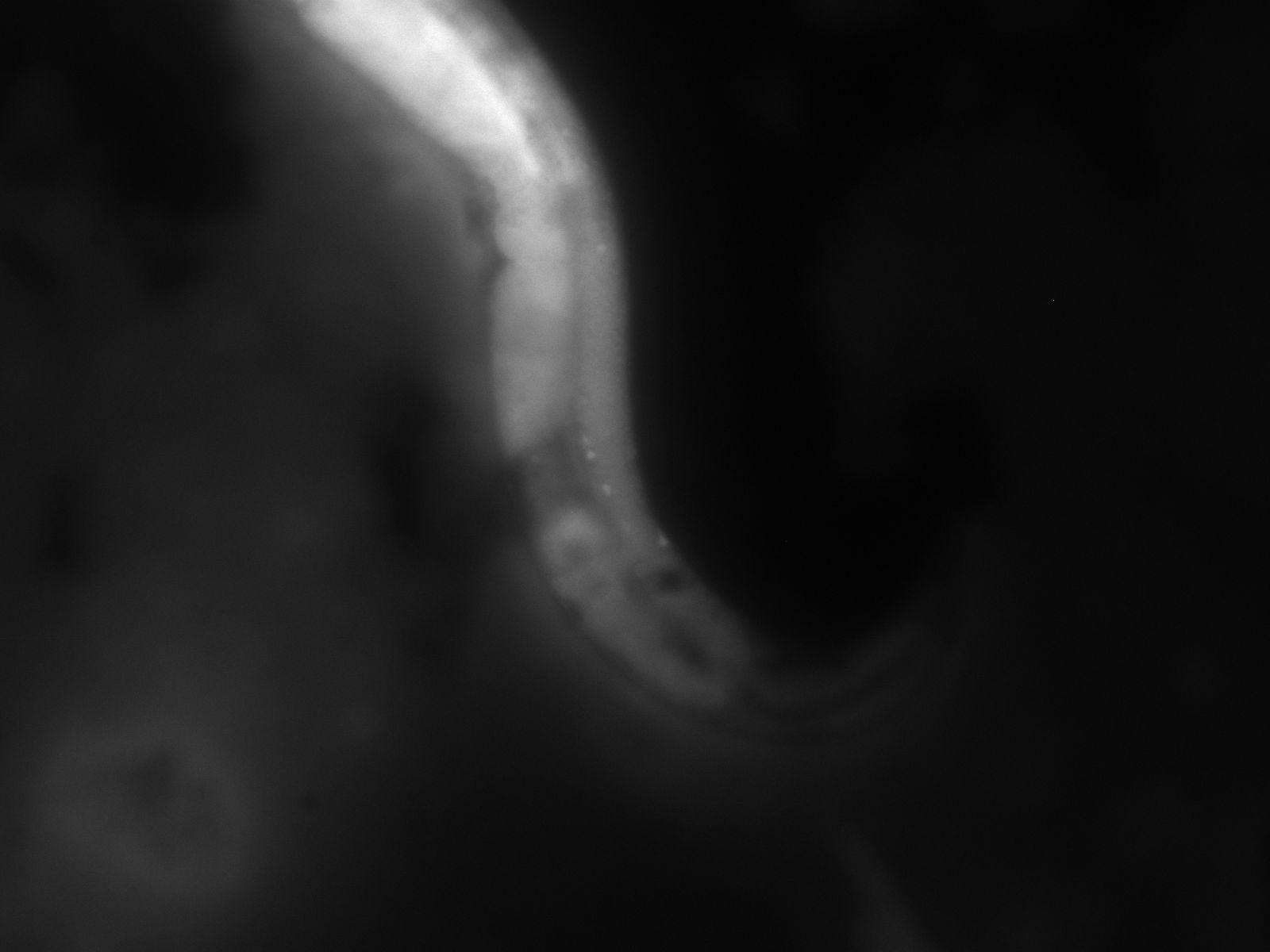

Supplement: S6 File — (ZIP) [file pgen.1011061.s006.zip › Fig.S4B+C - Original files/Fig.S4 RAW data and photos - JPEG/syto12 staining - FigS4bc - 2 rep_15.5.23/unc-31_unc-64+tfg-146.jpg]

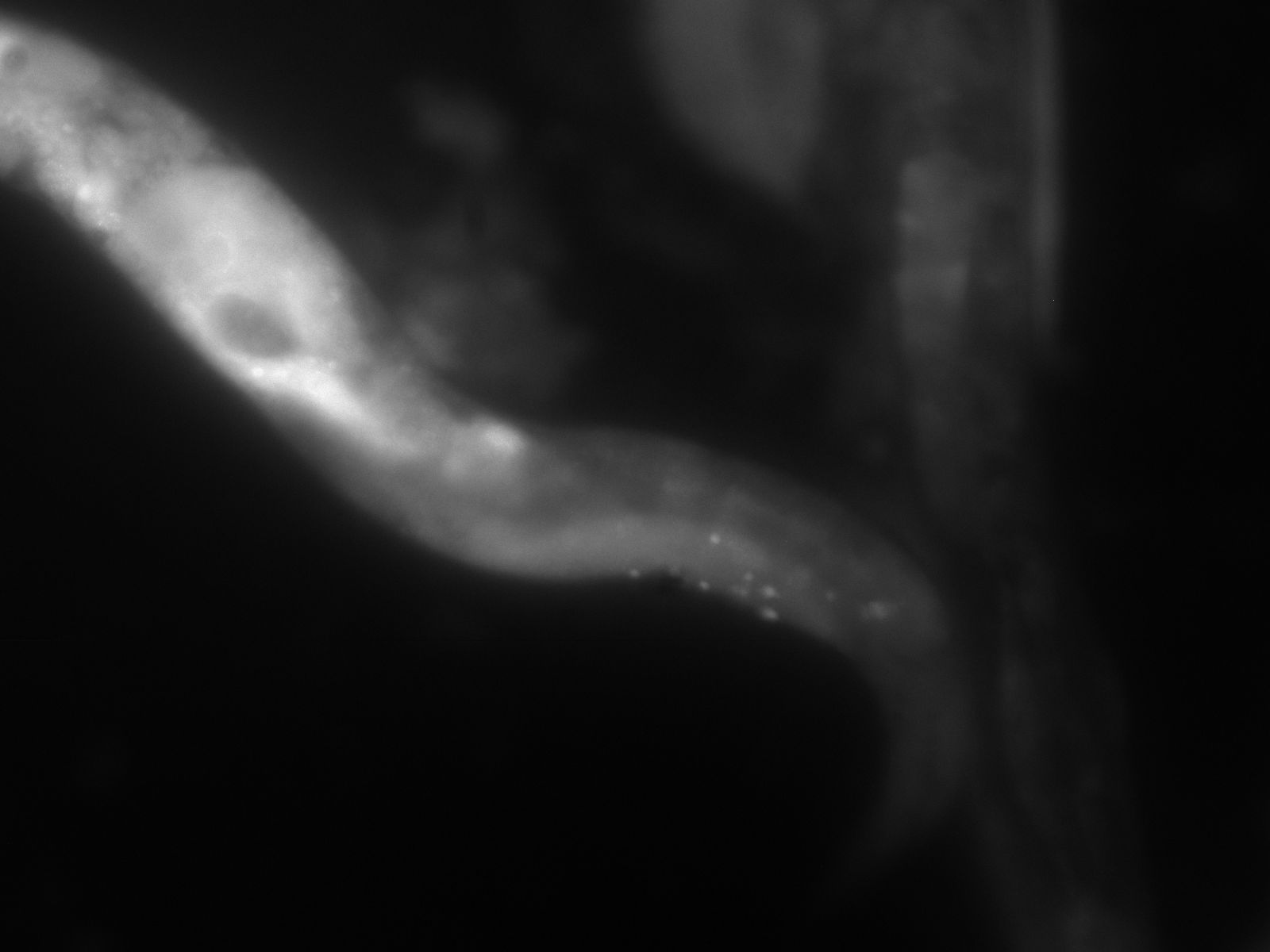

Supplement: S6 File — (ZIP) [file pgen.1011061.s006.zip › Fig.S4B+C - Original files/Fig.S4 RAW data and photos - JPEG/syto12 staining - FigS4bc - 2 rep_15.5.23/unc-31_unc-64+tfg-147.jpg]

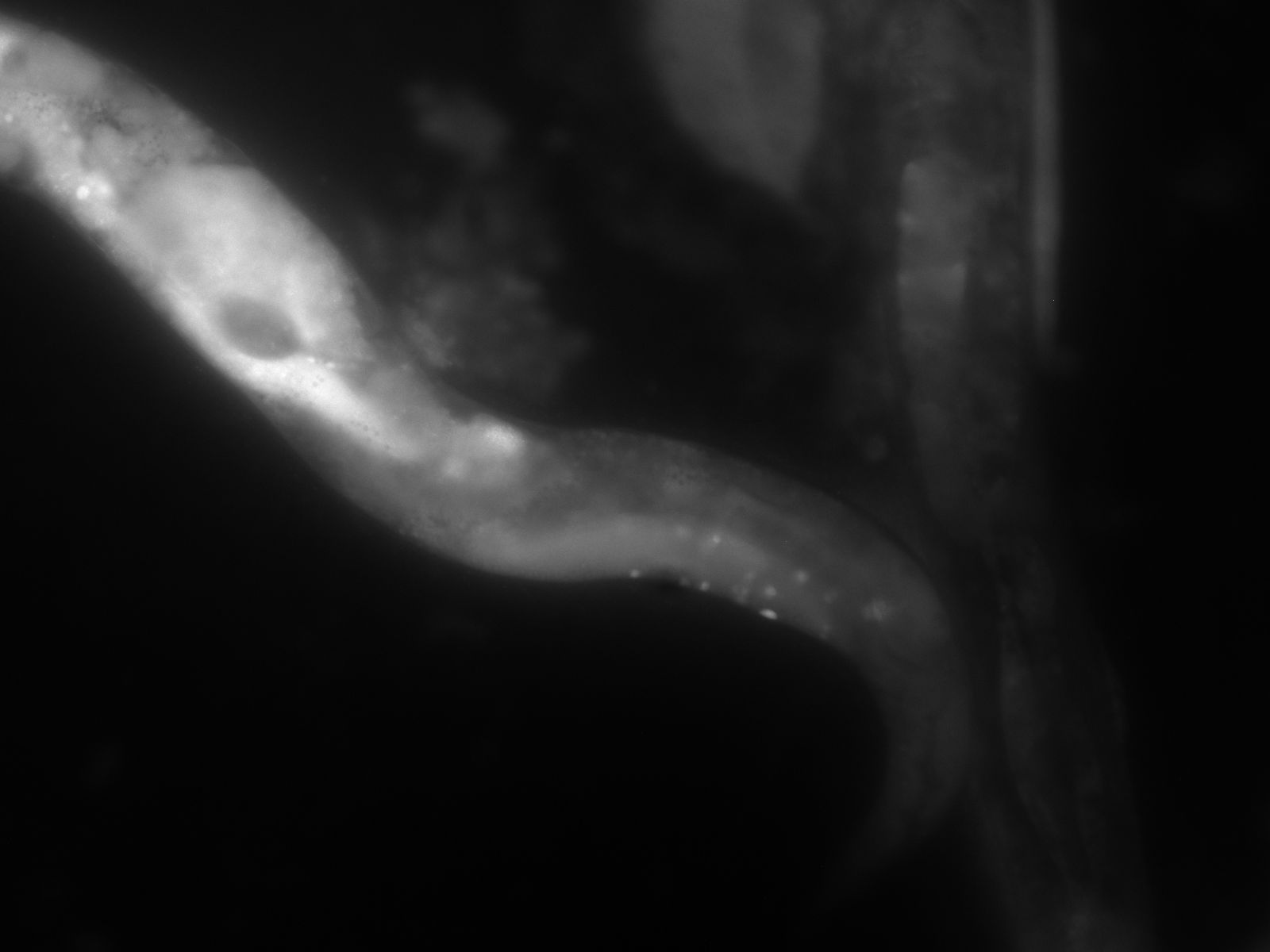

Supplement: S6 File — (ZIP) [file pgen.1011061.s006.zip › Fig.S4B+C - Original files/Fig.S4 RAW data and photos - JPEG/syto12 staining - FigS4bc - 2 rep_15.5.23/unc-31_unc-64+tfg-148.jpg]

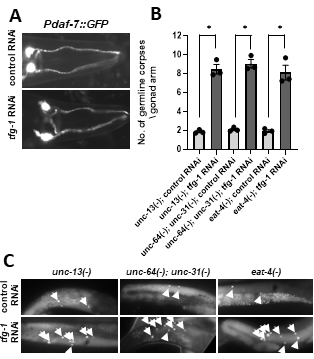

Supplement: S6 File — (ZIP) [file pgen.1011061.s006.zip › Fig.S4B+C - Original files/Updated Figure S4 - 25.6.23.tif]

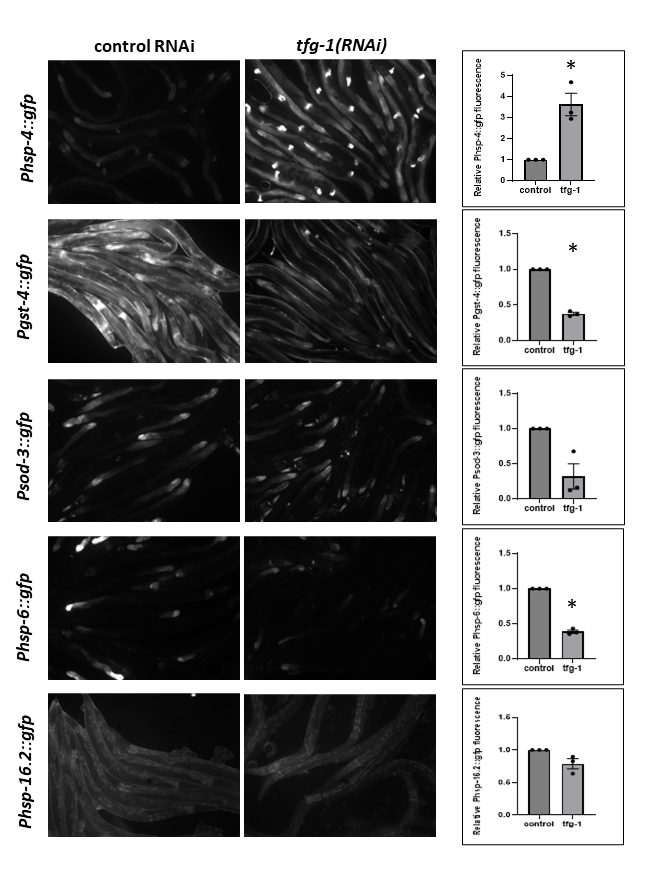

Supplement: S1 Fig — Representative fluorescence micrographs (100-fold magnification) of adult transgenic animals expressing a GFP reporter fused to promoters, whose activity is induced in response to a variety of cellular stresses. Animals were treated with control RNAi or with tfg-1 RNAi. Bar graph presents relative fluorescence +/-SEM of the indicated genotypes (n = 75 animals per genotype). Phsp-4::gfp is induced in response to ER stress, Pgst-4::gfp and Psod-3::gfp are induced in response to oxidative stress, Phsp-6::gfp is induced in response to mitochondrial stress and Phsp-16.2::gfp is induced in response to heat shock. Asterisk marks One sample-test value of P<0.05 compared to control RNAi. No increase in the levels of any of the cellular stress reporters was seen upon treatment with tfg-1 RNAi except for the Phsp-4::gfp ER stress response reporter. (TIF) [file pgen.1011061.s007.tif]

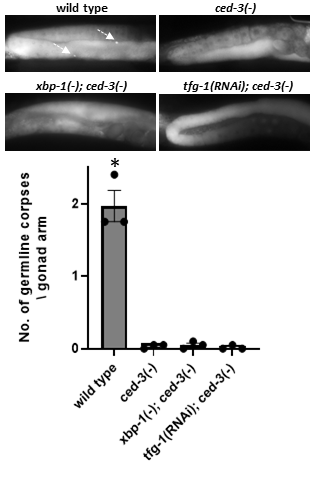

Supplement: S2 Fig — Representative fluorescence micrographs (200-fold magnification) of SYTO12-stained germ cell corpses in day-2 adults. Arrows point at SYTO12-labeled germ cell corpses. ER stress was induced by inactivation of the UPR gene xbp-1 or by blocking protein export from the ER by inactivation of tfg-1 (UPR is constitutively activated in tfg-1-deficient animals, see S1 Fig). No germ cell corpses were detected in either ced-3(n1286), xbp-1(tm2457); ced-3(n1286) or tfg-1(RNAi); ced-3(n1286) backgrounds. Bar graph shows average number +/-SEM of apoptotic corpses per gonad arm (n = 60 per genotype). Asterisk marks One-way ANOVA followed by Tukey’s multiple comparisons test values of P<0.05 compared to ced-3-deficient animals. (TIF) [file pgen.1011061.s008.tif]

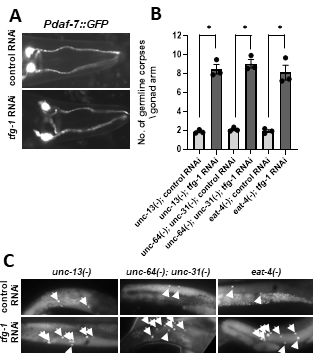

Supplement: S4 Fig — (A) Representative fluorescence micrographs (400-fold magnification) of GFP-expressing ASI neurons driven by the daf-7 promoter. The overall pattern of the ASI neurons was similar in control RNAi and the tfg-1 RNAi treated animals. (B-C) Bar graph and representative fluorescence micrographs (200-fold magnification) of germline corpses in unc-13(e51), unc-64(e246) unc-31(e928) and eat-4 (ky5) day-2 mutants are presented. The average number of apoptotic corpses per gonad arm was scored by SYTO12 staining (n = 60 animals per genotype). Asterisk marks One-way ANOVA followed by Šidák multiple comparisons test values of P<0.05. Error bars represent SEM +/-. Note that although these strains have a severely defective nervous system, they display normal basal levels of germline apoptosis, which increase in response to ER stress. These results uncouple general neuronal dysfunction and the responsiveness to ER stress-induced germline apoptosis. (TIF) [file pgen.1011061.s009.tif]
